# MTCH2 cooperates with MFN2 and lysophosphatidic acid synthesis to sustain mitochondrial fusion

**DOI:** 10.1038/s44319-023-00009-1

**Published:** 2023-12-14

**Authors:** Andres Goldman, Michael Mullokandov, Yehudit Zaltsman, Limor Regev, Smadar Levin-Zaidman, Atan Gross

**Affiliations:** 1 Montreal Neurological Institute, McGill University, Montreal, Canada; 2 Department of Immunology and Regenerative Biology, Weizmann Institute of Science, Rehovot, Israel; 3 Department of Chemical Research Support, Weizmann Institute of Science, Rehovot, Israel

**Keywords:** MTCH2, MFN2, LPA, Mitochondrial Fusion, Mitochondria-ER Communication, Membranes & Trafficking, Metabolism, Organelles

## Abstract

Fusion of the outer mitochondrial membrane (OMM) is regulated by mitofusin 1 (MFN1) and 2 (MFN2), yet the differential contribution of each of these proteins is less understood. Mitochondrial carrier homolog 2 (MTCH2) also plays a role in mitochondrial fusion, but its exact function remains unresolved. MTCH2 overexpression enforces MFN2-independent mitochondrial fusion, proposedly by modulating the phospholipid lysophosphatidic acid (LPA), which is synthesized by glycerol-phosphate acyl transferases (GPATs) in the endoplasmic reticulum (ER) and the OMM. Here we report that MTCH2 requires MFN1 to enforce mitochondrial fusion and that fragmentation caused by loss of MTCH2 can be specifically counterbalanced by overexpression of MFN2 but not MFN1, partially independent of its GTPase activity and mitochondrial localization. Pharmacological inhibition of GPATs (GPATi) or silencing ER-resident GPATs suppresses MFN2’s ability to compensate for the loss of MTCH2. Loss of either MTCH2, MFN2, or GPATi does not impair stress-induced mitochondrial fusion, whereas the combined loss of MTCH2 and GPATi or the combined loss of MTCH2 and MFN2 does. Taken together, we unmask two cooperative mechanisms that sustain mitochondrial fusion.

## Synopsis

MTCH2 and MFN2 govern two redundant and complementary pathways required to sustain normal mitochondrial architecture and plasticity.

- MTCH2 enforces mitochondrial fusion through MFN1.
- MFN2 specifically restores mitochondrial fusion in MTCH2 knockout cells, partially independent of its fusion activity.
- MFN2’s rescue ability is sensitive to loss of the pro-fusion lipid LPA.
- Loss of either MTCH2 in combination with GPAT inhibition (GPATi) or loss of both MTCH2 and MFN2 impairs mitochondrial plasticity.

## Abstract (web summary)

MTCH2 and MFN2 govern two redundant and complementary pathways required to sustain normal mitochondrial architecture and plasticity.

## Graphical abstract

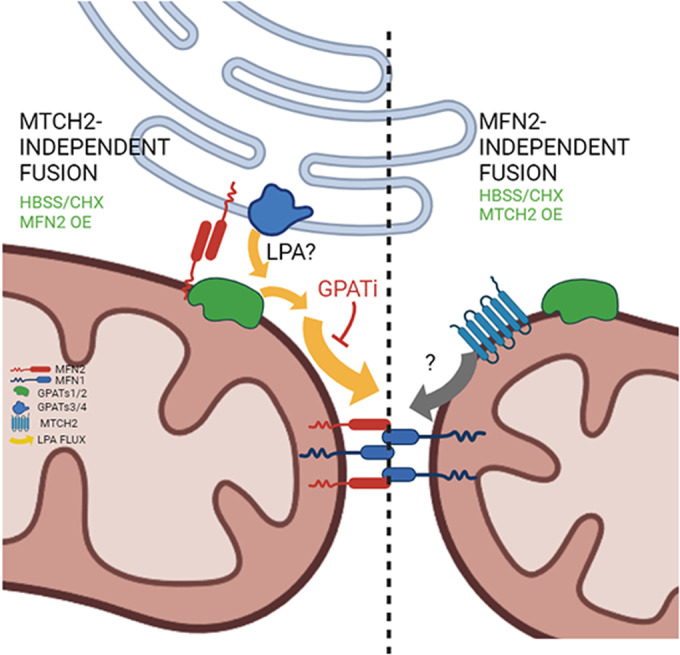

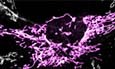

## Introduction

Mitochondria are highly dynamic organelles that undergo continuous remodeling through cycles of fusion and fission events (Bereiter-Hahn and Vöth, 1994; Nunnari et al, 1997; Sesaki and Jensen, 1999; Rowland and Voeltz, 2012). Mitochondrial fusion relies on three dynamin-related proteins (DRPs): optic atrophy protein 1 (OPA1) required for inner mitochondrial membrane (IMM) fusion (Olichon et al, 2002, 2003; Cipolat et al, 2004), and MFN1/2 required for OMM fusion (Santel and Fuller, 2001; Chen et al, 2003). MFN1 is essential for mitochondrial tethering and has higher GTPase activity compared to MFN2 (Ishihara et al, 2004).

Although in vitro mitochondrial fusion assays showed that MFN1/MFN2 heterodimers yield the highest mitochondrial fusion rate (Meeusen et al, 2004), MFN2 is dispensable for mitochondrial fusion under different stimuli, such as amino-acid deprivation, stress-induced mitochondrial hyperfusion (SIMH) and inhibition of mitochondrial fission (Cipolat et al, 2004; Tondera et al, 2009; Rambold et al, 2011; Zhao et al, 2011; Lee et al, 2014). Besides its mitochondrial fusion role, MFN2 acts as a molecular tether between mitochondria and different organelles (Merkwirth and Langer, 2008; Dorn, 2020; Huo et al, 2022; Zervopoulos et al, 2022) and localizes to the ER where it physically tethers mitochondria (de Brito and Scorrano, 2008; Sugiura et al, 2013; Casellas-Díaz et al, 2021).

MTCH2 is a non-classical OMM member of the mitochondrial carrier protein family (Robinson et al, 2012). MTCH2 regulates apoptosis (Grinberg et al, 2005; Zaltsman et al, 2010), metabolism (Maryanovich et al, 2015; Buzaglo-Azriel et al, 2016; Ruggiero et al, 2017), and was recently reported to function as an OMM insertase of tail-anchored proteins (Guna et al, 2022). MTCH2 plays an important role in lipid metabolism (Kulyté et al, 2011; Bernhard et al, 2013; Bar-Lev et al, 2016; Landgraf et al, 2016a; Rottiers et al, 2017; Labbé et al, 2021), and regulates mitochondrial fusion (Bahat et al, 2018). In line with these findings, it was proposed that MTCH2 regulates mitochondrial fusion by modulating the pro-mitochondrial fusion lipid lysophosphatidic acid (LPA) (Labbé et al, 2021).

LPA is the first product of the de-novo phospholipid biosynthesis (KENNEDY, 1961) and is formed by the addition of an acyl-CoA group to a glycerol-3 phosphate molecule, a reaction catalyzed by glycerol-phosphate acyl transferases (GPATs) (Coleman and Lee, 2004; Yamashita et al, 2014). Four different GPATs have been described: GPATs1/2, localized to the OMM (Coleman and Lee, 2004; Lewin et al, 2004; Pellon-Maison et al, 2007), and GPATs3/4, localized to the ER (Coleman and Lee, 2004; Jingsong et al, 2006). LPA synthesis is thought to occur at mitochondria-associated membranes (MAMs) (Pellon-Maison et al, 2007). Deletion of mitochondrial GPATs1 and 2 (Ohba et al, 2013), or pharmacological inhibition of GPATs activity, elicits mitochondrial fragmentation (Labbé et al, 2021).

## Results and discussion

### MTCH2 and MFN1 form a mitochondrial fusion pathway independent of MFN2

**Figure 1:**
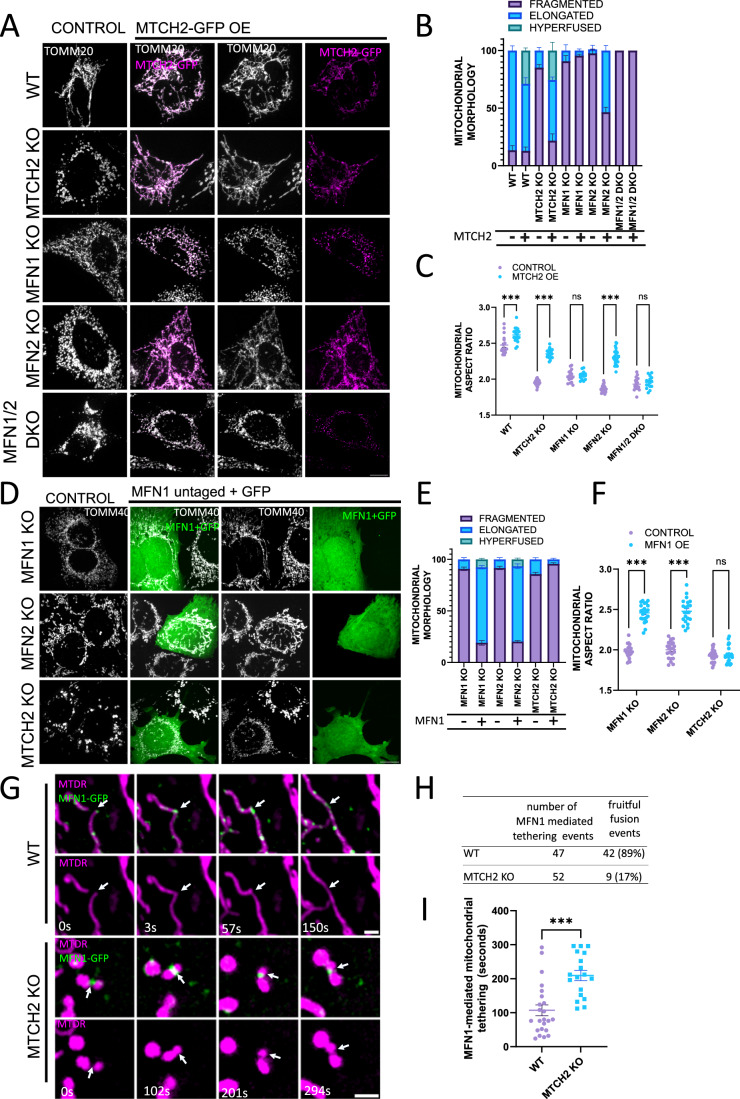
MTCH2 and MFN1 form a mitochondrial fusion pathway independent of MFN2. (**A**) Representative images of WT, MTCH2 KO, MFN1 KO, MFN2 KO, and MFN1/2 DKO MEFs either not expressing (control) or expressing MTCH2-GFP. Mitochondria were stained with αTOMM20 antibody. Scale bar 10 µm. (**B**) Classical classification of mitochondrial network morphology, showing average and SEM of three separate experiments (at least 20 cells were analyzed per condition). (**C**) Mitochondrial aspect ratio analysis (at least 20 control and 20 transfected cells were analyzed per condition). One-way ANOVA statistical analysis and SEM are shown. (**D**) Representative images of MFN1 KO, MFN2 KO, and MTCH2 KO MEFs either not expressing (control) or expressing untagged MFN1 and co-transfected with GFP. Mitochondria were stained with αTOMM40 antibody. Scale bar 10 µm. (**E**) Classical classification of mitochondrial network morphology of cells, showing average and SEM of three separate experiments (at least 20 cells were analyzed per condition). (**F**) Mitochondrial aspect ratio analysis (at least 20 control and 20 transfected cells were analyzed per condition). One-way ANOVA statistical analysis and SEM are shown. (**G**) Representative time-lapse sequences of MFN1-mediated tethering events between mitochondria in WT or MTCH2 KO MEFs. Cells were transfected with MFN1-GFP and mitochondria were stained with mitotracker deep red (MTDR). Time-lapse experiments were acquired for 5 min, capturing both channels every 3 s. Arrowheads follow a fruitful fusion event in WT cells, and arrowheads in MTCH2 KO cells depict an unfruitful fusion event, associated with long tethering time. Scale bar 2 µm. (**H**) Number of MFN1-GFP-mediated tethering events monitored, and number of fruitful fusion events detected in WT and MTCH2 KO MEFs. (**I**) Quantification of MFN1-mediated mitochondrial tethering time in WT and MTCH2 KO MEFs. Approximately 20 MFN1-GFP-mediated tethering events obtained from at least 10 different transfected cells were followed in WT and MTCH2 KO MEFs, according to the conditions described in (**G**). The duration of the MFN1-GFP-mediated tethering event was determined by the time between the initial tethering and the lapse until mitochondria separated or fused with each other. Only tethering events that lasted more than 15 s were considered for this analysis to separate them from mitochondria “kiss and run” events. The data is presented as average tethering time and SEM, and statistical analysis was performed by t-test. Data information: (**C**,**F**,**I**) ns, not significant, ***P < 0.001. Source data are available online for this figure.

MTCH2 overexpression enforces mitochondria elongation in MFN2 knockout (KO) MEFs (Bahat et al, 2018), and in wild-type (WT) HCT116 cells (Labbé et al, 2021). Since MTCH2 lacks GTPase activity, its ability to induce mitochondrial fusion depends on the presence of at least one of the MFNs. To test this, we overexpressed MTCH2-GFP in WT, MTCH2 KO, MFN1 KO, MFN2 KO, and MFN1/MFN2 double KO (DKO) MEFs, and assessed mitochondrial network morphology by classical morphological classification, and quantified mitochondrial shape using the geometrical descriptor aspect ratio (AR). Overexpression of MTCH2-GFP rescued mitochondrial elongation in MTCH2 KO MEFs, and elicited mitochondrial hyperfusion in ~20% of WT and MTCH2 KO cells (Fig. 1A–C). In addition, MTCH2-GFP overexpression enforced mitochondrial elongation in MFN2 KO but not in MFN1 KO or in MFN1/2 DKO MEFs (Fig. 1A,B). AR analysis recapitulates the effect of MTCH2 overexpression by showing a significant increase in the ratio between mitochondrial length and width (Fig. 1C). Thus, MTCH2-enforced mitochondrial elongation requires the expression of MFN1 but not of MFN2.

Overexpression of MFN2 can restore mitochondrial elongation in MTCH2 KO cells (Bahat et al, 2018). Since MFN1 was reported to possess higher GTPase activity than MFN2 (Ishihara et al, 2004), we tested whether overexpression of MFN1 could enforce mitochondrial fusion in MTCH2 KO cells. To address this question, we overexpressed an untagged version of MFN1 together with GFP to identify transfected cells in MFN1 KO, MTCH2 KO, and MFN2 KO MEFs. Overexpression of MFN1 elicited mitochondrial elongation in both MFN1 KO and MFN2 KO cells, but not in MTCH2 KO MEFs (Fig. 1D–F). Thus, MFN1 overexpression requires MTCH2 and vice-versa, to enforce mitochondrial elongation.

To explore the defect in mitochondrial fusion caused by MTCH2 KO deletion, we generated an MFN1-GFP that behaves the same way as the untagged version (Fig. EV1A–C) and performed live cell imaging of WT and MTCH2 KO MEFs expressing MFN1-GFP in the search for mitochondrial fusion events. In both WT and MTCH2 KO cells, MFN1-GFP was localized to the mitochondria forming discrete foci (Fig. 1G). WT cells showed mitochondrial fusion events associated with MFN1-GFP foci (Fig. 1G, top timeline), while these events were rare in MTCH2 KO cells (Fig. 1H). Instead, in the absence of MTCH2, MFN1-GFP foci marked tethering events between mitochondria, but in most cases with no clear resolution of the fusion intermediates (Fig. 1G, bottom timeline and H). We quantified MFN1-GFP associated tethering time between mitochondria by measuring the duration of MFN1-GFP mediated tethering events that lasted for more than 15 s, and found that MTCH2 deletion elicited close to a two-fold increase in the time mitochondria spent tethered (Fig. 1I), suggesting a possible defect in mitochondrial fusion downstream of MFN1 tethering.

**Figure EV1:**

1 MTCH2 and MFN1 form a mitochondrial fusion pathway independent of MFN2. (**A**) Representative images of MFN1 KO and MTCH2 KO MEFs either not expressing (control) or expressing MFN1-GFP. Mitochondria were stained with αTOMM20 antibody. Scale bar 10 µm. (**B**) Classical classification of mitochondrial network morphology of cells, showing average and SEM of three separate experiments (at least 20 cells were analyzed per condition). (**C**) Mitochondrial aspect ratio analysis. At least 20 control and 20 transfected cells were analyzed per condition. One-way ANOVA statistical analysis and SEM are shown. (**D**) Western blot analysis of mitochondrial fusion/fission machinery in heavy membrane (HM) fractions of WT and MTCH2 KO MEFs. (**E**) BN-GE of WT and MTCH2 KO HM fractions blotted with anti-MFN1 and MFN2 antibodies. SDHA serves as control. (**F**) qRT-PCR of MFN1 expression levels in WT MEFs treated with NT-siRNA or with MTCH1-siRNA. (**G**) Representative images of WT MEFs treated with NT-siRNA or with MTCH1-siRNA for 3 days. Mitochondria were stained with αTOMM40 antibody. Scale bar 10 µm. (**H**) Classical classification of mitochondrial network morphology of cells, showing average and SEM of three separate experiments (at least 20 cells were analyzed per condition). (**I**) Mitochondrial aspect ratio analysis. At least 20 cells were analyzed per condition. T-test statistical analysis and SEM are shown. (**J**) Representative images of MTCH2 KO MEFs expressing WT MTCH2-GFP, MTCH2-K25E GFP, or MTCH2-D189R GFP. Mitochondria were stained with αTOMM40 antibody. Scale bar 10 µm. (**K**) Classical classification of mitochondrial network morphology of cells, showing average and SEM of three separate experiments (at least 20 cells were analyzed per condition). (**L**) Mitochondrial aspect ratio analysis. At least 20 cells were analyzed per condition. One-way ANOVA statistical analysis and SEM are shown. Data information: (**C**,**I**,**L**) ns, not significant, ***P < 0.001. Source data are available online for this figure.

MTCH2 was recently reported to function as an OMM insertase (Guna et al, 2022), and thus we assessed whether MTCH2 KO mitochondrial fragmentation is possibly the outcome of reduced levels of mitochondrial dynamics-related proteins. Western blot analysis performed on heavy membrane (HM) fractions showed that MTCH2 deletion did not affect the expression levels of MFN1, MFN2, nor the mitochondrial fission proteins DRP1, MFF, FIS1, or MID49 (Fig. EV1D). Also, OPA1 expression levels and isoform were unaffected by MTCH2 deletion (Fig. EV1D), suggesting that OPA1 cleavage into its short isoforms does not contribute to MTCH2 KO-dependent mitochondrial fragmentation. MFN1/MFN2 oligomerization is required for mitochondrial fusion (Ishihara et al, 2004). Using blue native gel electrophoresis we evaluated if MTCH2 deletion impacted MFN1/2 complex formation, and found no differences in the levels or composition of MFN1 and MFN2 native complexes (Fig. EV1E).

Guna et al also reported that MTCH1 acts as an OMM insertase working in a synergistic fashion with MTCH2 (Guna et al, 2022), and therefore its deletion might also elicit mitochondrial fragmentation. To test this, we silenced MTCH1 in WT MEFs, confirmed its downregulation (Fig. EV1F), and found no effect on the mitochondrial network morphology (Fig. EV1G–I). We also assessed if MTCH2’s insertase activity is required for mitochondrial fusion. To test this possibility, we overexpressed a MTCH2-GFP hyperactive mutant (K25E) and a MTCH2-GFP inactive mutant (D189R) in MTCH2 KO cells and found that both mutants could efficiently rescue mitochondrial elongation (Fig. EV1J–L). However, it should be noted that MTCH2 KO cells transfected with the MTCH2-GFP inactive mutant (D189R) seemed to demonstrate more cells with fragmented mitochondria as compared to MTCH2 KO cells transfected with WT MTCH2-GFP (Fig. EV1K). Altogether, these results suggest that MTCH2 may require its insertase activity in a partial manner to regulate mitochondrial dynamics.

### MFN2’s ability to enforce mitochondrial fusion in MTCH2 KO cells is partially disconnected from its fusion activity

**Figure 2:**
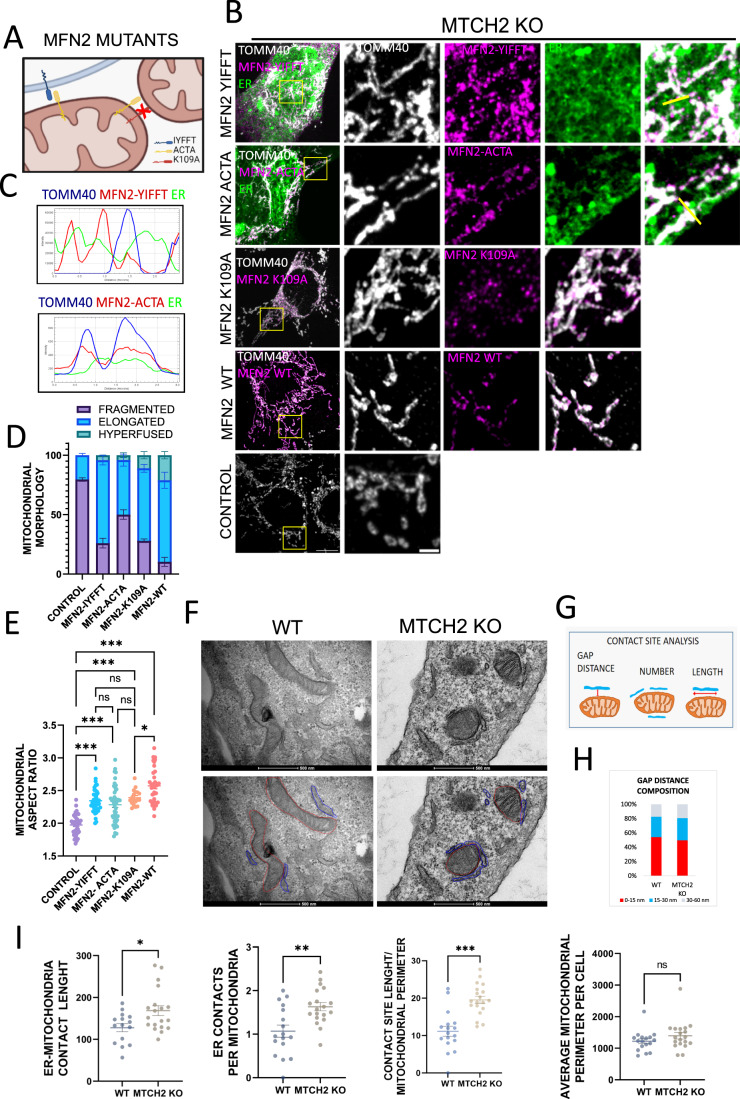
MFN2’s ability to enforce mitochondrial fusion in MTCH2 KO cells is partially disconnected from its fusion activity. (**A**) Schematic representation of MFN2 mutants utilized in this study. MFN2-IYFFT, a construct targeted to the ER compartment, MFN2-ACTA, a construct which is artificially targeted only to the mitochondria, and MFN2-K109A GTPase, a mutant that does not hydrolyze GTP and cannot mediate mitochondrial fusion. (**B**) Representatives images of MTCH2 KO MEFs control or transfected with MFN2-IYFFT-FLAG, MFN2-ACTA-FLAG, MFN2-K109A-FLAG, or WT MFN2-MYC. MFN2-IYFFT and MFN2-ACTA mutants were co-transfected with the ER marker Sec61-GFP to ensure the correct subcellular localization. Mitochondria were stained with αTOMM40 antibody. Scale bar 10 µm; inset scale bar 2 µm. (**C**) Line fluorescence intensity plot of yellow lines shown in (**B**). Mitochondria (blue), ER (green), and either MFN2-IYFFT (red upper plot) or MFN2-ACTA (red bottom plot) are shown. (**D**) Classical classification of mitochondrial network morphology showing average and SEM of three separate experiments (at least 20 cells were analyzed in per condition). (**E**) Mitochondrial aspect ratio analysis. At least 25 control and 25 transfected cells were analyzed per condition. One-way ANOVA statistical analysis and SEM are shown. (**F**) Top panel: Electron micrographs showing representative areas depicting mitochondria-ER contact regions in WT and MTCH2 KO cells. Bottom panel: freehand modeling of ER and mitochondria. ER regions that were in a distance of 60 nm or less were considered as contact regions and freehand delineated in blue. Mitochondrial perimeter was delineated in red. Scale bar 500 nm. (**G**) Schematic representation of ER-mitochondria contact sites analysis. For every ER-mitochondria contact GAP distance and length were quantified. Also, the number of ER-mitochondria contact sites per cell was analyzed and normalized to the total number of mitochondria per cell. Finally, using the total mitochondrial perimeter per cell, and the total length of the ER-mitochondria contacting regions, the average of the percentage of mitochondrial perimeter covered by contacts with the ER per cell was calculated. (**H**) Quantification of ER-mitochondria contact sites GAP distance composition in WT and MTCH2 KO MEFs. (**I**) Quantification of ER-mitochondria contact sites: average number of ER-mitochondria contact sites per mitochondria; average length of ER-mitochondria contacts sites; average mitochondrial perimeter and percentage of mitochondrial perimeter covered by ER contact sites. At least 25 WT and 25 MTCH2 KO cells were analyzed, statistical analysis was performed by t-test and SEM is shown. Data information: (**E**,**I**) ns, not significant, *P < 0.05, **P < 0.01, ***P < 0.001. Source data are available online for this figure.

To further investigate the functional basis underlying the specificity of MFN2 over MFN1 to enforce MTCH2-independent mitochondrial fusion, we tested the rescue capacity of three different mutants: MFN2-mito-targeted (MFN2-ACTA (Annis et al, 2001)), the MFN2-ER-targeted (MFN2-IYFFT (Rojo et al, 2002)), and a GTPase mutant unable to hydrolyze GTP (K109A (Yan et al, 2018)) (Fig. 2A). First, we investigated the functionality of these mutants in MFN2 KO MEFs, and found that expression of MFN2-K109A could not rescue mitochondrial fragmentation, expression of either MFN2-ACTA or MFN2-IYFFT resulted in a small increase in mitochondrial length, and co-expression of both MFN2-ACTA and MFN2-IYFFT fully restored mitochondrial elongation (Fig. EV2A,B).

**Figure EV2:**

MFN2’s ability to enforce mitochondrial fusion in MTCH2 KO cells is partially disconnected from its fusion activity. (**A**) Representatives images of MFN2 KO MEFs control or transfected with MFN2-K109A-FLAG, MFN2-IYFFT-MYC, MFN2-ACTA-V5, WT MFN2-MYC, or co-transfected with MFN2-IYFFT-MYC and MFN2-ACTA-V5. Mitochondria were stained with αTOMM20 antibody. Scale bar 10 µm; inset scale bar 5 µm. (**B**) Mitochondrial aspect ratio analysis (at least 25 control and 25 transfected cells were analyzed per condition). One-way ANOVA statistical analysis and SEM are shown. (**C**) Representative images of WT and MTCH2 KO cells transfected with the ER marker Sec61b-GFP and stained with αTOMM20 antibody. Arrowheads indicate areas of close proximity between ER and mitochondria. Scale bar 10 µm; inset scale bar 2 µm. (**D**) Representatives images of WT and MTCH2 KO MEFs co-transfected with the ER-mitochondria linker construct AKAP-mtagBFP2-UBC6 and the ER marker Sec61b-GFP. Mitochondria were labeled with MTDR. Scale bar 10 µm; Scale bar inset 5 µm. (**E**) Mitochondrial aspect ratio analysis. At least 15 control and 15 transfected cells were analyzed per condition. One-way ANOVA statistical analysis and SEM are shown. Data information: (**B**,**E**) ns, not significant, ***P < 0.001. Source data are available online for this figure.

In MTCH2 KO cells, overexpression of WT MFN2 rescued mitochondrial fragmentation, whereas overexpression of MFN2-ACTA and MFN2-IYFFT and the MFN2-GTPase mutant K109A partially rescued MTCH2 KO mitochondrial fragmentation, changing mitochondrial shape from round to more tubular/elongated (Fig. 2B,D,E). We confirmed the correct subcellular localization of the MFN2-ACTA and MFN2-IYFFT by co-transfecting cells with the ER marker Sec61b-GFP and analyzing their localization using line fluorescence plot (Fig. 2C). Altogether these results indicate that MFN2 fusion activity and mitochondrial localization are required to fully restore mitochondrial elongation in MTCH2 KO cells, but also suggest that a mitochondrial fusion unrelated role of MFN2, which is absent in MFN1, might contribute to enforce MTCH2-independent changes in mitochondrial morphology.

MFN2 is an important ER-mitochondria tethering factor, and because overexpression of ER-localized MFN2-IYFFT partially rescued MTCH2 KO mitochondria fragmentation, we investigated whether MTCH2 deletion leads to a defect in mitochondria-ER tethering. Using transmission electron microscopy (TEM) we analyzed GAP distance between mitochondria and ER, number of ER contacts per mitochondria, average length of contacts, and the percentage of mitochondrial perimeter covered by contacts (Fig. 2G). MTCH2 deletion had no effect on the distance that separates both organelles (Fig. 2F–H), but it did elicit an increase in the number and length of contact sites (Fig. 2F,G,I). These results translate into an overall increase in the total contact surface between the two organelles with no difference in the proximity.

Next, we evaluated the macro-morphology of the ER and mitochondrial networks at the light microscopy level, by transfecting WT and MTCH2 KO MEFs with the ER marker Sec61b-GFP. ER morphology appeared normal in MTCH2 KO MEFs, and in agreement with our TEM data, we observed large overlapping areas between ER and mitochondria (Fig. EV2C). Next, we investigated if MFN2’s rescue ability could be phenocopied by overexpressing the artificial ER-mitochondria tether AKAP-mtagBFP2-UBC6 (Hirabayashi et al, 2017), and found that it did not (Fig. EV2D,E). Altogether, these results suggest that MFN2-YIFFT’s and MFN2-K109A’s abilities to counterbalance MTCH2 KO mitochondrial fragmentation is most likely due to a function that is different from enforcing the tethering between ER and mitochondria.

### MTCH2 deletion uncovers the requirement of LPA synthesis to sustain mitochondrial fusion enforced by MFN2 overexpression

**Figure 3:**
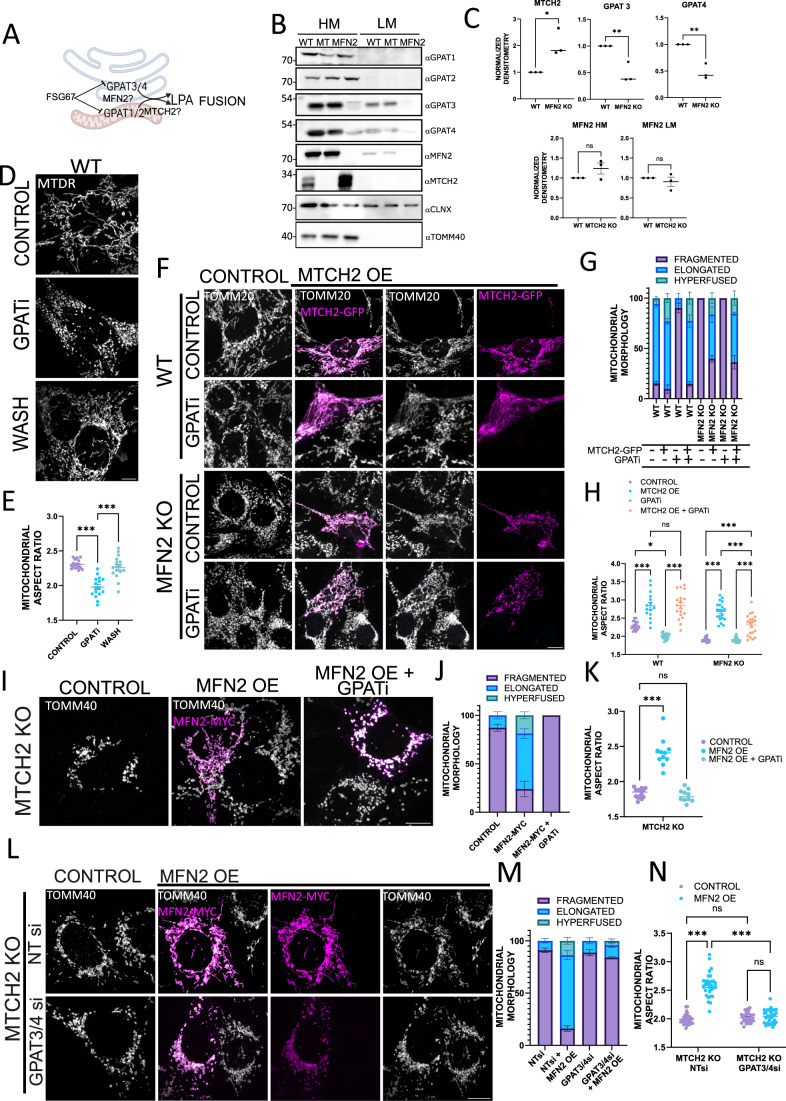
MTCH2 deletion uncovers the requirement of LPA synthesis to sustain mitochondrial fusion enforced by MFN2 overexpression. (**A**) Schematic representation of the LPA synthesis machinery. GPATs1/2 synthesize mitochondrial LPA required for phospholipid biosynthesis and for mitochondrial fusion as well. GPATs3/4 synthesize LPA in the ER compartment while LPA synthesis was shown to take place at MAMs. GPATs inhibitor (GPATi) FSG67 inhibits the activity of all of the GPATs and resulting in mitochondrial fragmentation. (**B**) Western blot analysis of GPATs1-4 in HM and light membrane (LM) fractions of WT, MTCH2 KO (MT), and MFN2 KO MEFs. TOMM40 serves as a HM loading control and calnexin (CLNX) serves as a LM loading control. (**C**) Upper plots: Densitometry analysis of MTCH2 and GPATs3/4 levels in HM fractions of WT, MFN2 KO MEFs; Lower plots: Densitometry analysis of MFN2 levels in HM and LM fractions of WT and MTCH2 KO MEFs. Band intensities were normalized to loading controls and the averages of three separate measurements are shown. One-way ANOVA statistical analysis and SEM are shown. (**D**) Representatives images of WT MEFs non-treated (control) or treated for 16 h with 100 μM GPATi −/+ wash, and imaged after 4 h. Mitochondria were labeled with MTDR. Scale bar 10 µm. (**E**) Mitochondrial aspect ratio analysis. At least 30 fields were analyzed per condition. One-way ANOVA statistical analysis and SEM are shown. (**F**) Representative images of WT and MFN2 KO MEFs control or overexpressing MTCH2-GFP in non-treated or treated for 16 h with GPATi. Mitochondria were stained with αTOMM20 antibody. Scale bar 10 µm. (**G**) Classical classification of mitochondrial network morphology. Results are presented as average and SEM of three separate experiments; at least 20 cells were analyzed per condition. (**H**) Mitochondrial aspect ratio analysis. At least 25 control and 25 transfected cells were analyzed per condition. One-way ANOVA statistical analysis and SEM are shown. (**I**) Representatives images of MTCH2 KO MEFs control or overexpressing MFN2-MYC in non-treated or treated for 16 h with GPATi. Mitochondria were stained using αTOMM40 antibody. Scale bar 10 µm. (**J**) Classical classification of mitochondrial network morphology. Results are presented as average and SEM of three separate experiments; at least 20 cells were analyzed per condition. (**K**) Mitochondrial aspect ratio analysis. At least 25 control and 25 transfected cells were analyzed per condition. One-way ANOVA statistical analysis and SEM are shown. (**L**) Representatives images of MTCH2 KO MEFs treated with non-targeting (NT) siRNA or with a combination of GPAT3 and GPAT4 siRNAs in control conditions or overexpressing MFN2-MYC. Mitochondria were stained using αTOMM40 antibody. Scale bar 10 µm. (**M**) Classical classification of mitochondrial network morphology. Results presented as average and SEM of three separate experiments; at least 20 cells were analyzed per condition. (**N**) Mitochondrial aspect ratio analysis. At least 25 control and 25 transfected cells were analyzed per condition. One-way ANOVA statistical analysis and SEM are shown. Data information: (**C**,**E**,**H**,**K**,**N**) ns, not significant, *P < 0.05, **P < 0.01, ***P < 0.001. Source data are available online for this figure.

The ability of MFN2 of the MFN2-IYFFT and the MFN2-GTPase mutants to partially enforce MTCH2-independent mitochondrial fusion suggested the involvement of a different functional role of MFN2. Several studies have highlighted the requirement of MTCH2 and MFN2 for mitochondrial lipid biosynthesis (Kulyté et al, 2011; Bernhard et al, 2013; Bar-Lev et al, 2016; Landgraf et al, 2016b; Rottiers et al, 2017; Chung et al, 2019), and MFN2 was shown to be necessary for phospholipid transfer to the mitochondria (Area-Gomez et al, 2012; Hernández-Alvarez et al, 2019). Since MTCH2 overexpression can rescue mitochondrial elongation in MFN2 KO cells and vice-versa, we reasoned that lipid homeostasis, and in particular LPA synthesis and/or transfer, may offer clues to uncovering the complementary roles of MTCH2 and MFN2 in mitochondrial fusion (Fig. 3A).

**Figure EV3:**
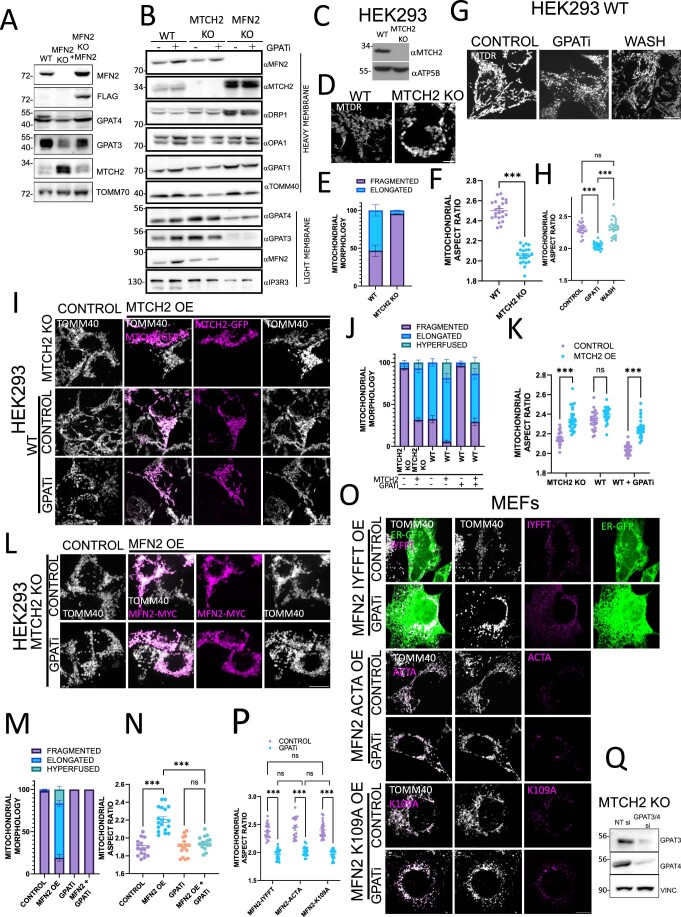
MTCH2 deletion uncovers the requirement of LPA synthesis to sustain mitochondrial fusion enforced by MFN2 overexpression. (**A**) Western blot analysis in whole-cell lysates of GPAT3/4 and MTCH2 in WT, MFN2 KO, and MFN2 KO MEFs rescued with FLAG-MFN2; TOMM70 serves as loading control. (**B**) Western blot analysis of mitochondrial fusion/fission machinery and GPATs in HM and LM fractions of WT, MTCH2 KO, and MFN2 KO MEFs control or treated for 16 h with GPATi. TOMM40 serves as a HM loading control, and IP3R3 serve as a LM loading control. (**C**) Western blot analysis of MTCH2 expression in WT and MTCH2 KO HEK293T cells; ATPVb (complex V) serves as a loading control. (**D**) Representatives images of mitochondria of WT and MTCH2 KO HEK293T cells. Mitochondria were labeled with MTDR. Scale bar 5 µm. (**E**) Classical classification of mitochondrial network morphology. Results are presented as average and SEM of three separate experiments; at least 20 fields were analyzed per condition. (**F**) Mitochondrial aspect ratio analysis. At least 25 fields were analyzed per condition. One-way ANOVA statistical analysis and SEM are shown. (**G**) Representatives images of WT HEK293T cells either non-treated (control) or treated for 16 h with GPATi −/+ wash and imaged after 4 h. Mitochondria were labeled with MTDR. Scale bar 10 µm. (**H**) Mitochondrial aspect ratio analysis. At least 25 fields were analyzed per condition. One-way ANOVA statistical analysis and SEM are shown. (**I**) Representatives images of WT and MTCH2 KO HEK293T cells overexpressing MTCH2-GFP in control conditions, and of WT cells treated for 16 h with GPATi and overexpressing MTCH2-GFP. Mitochondria were stained using αTOMM40 antibody. Scale bar 10 µm. (**J**) Classical classification of mitochondrial network morphology. Results are presented as average and SEM of three separate experiments; at least 20 cells were analyzed per condition. (**K**) Mitochondrial aspect ratio analysis. At least 25 control and 25 transfected cells were analyzed per condition. One-way ANOVA statistical analysis and SEM are shown. (**L**) Representatives images of MTCH2 KO HEK293T cells control or overexpressing MFN2-MYC non-treated or treated for 16 h with GPATi. Mitochondria were stained using αTOMM40 antibody. Scale bar 10 µm. (**M**) Classical classification of mitochondrial network morphology. Results are presented as average and SEM of three separate experiments; at least 20 cells were analyzed per condition. (**N**) Mitochondrial aspect ratio analysis of cells presented in L. At least 25 control and 25 transfected cells were analyzed per condition. One-way ANOVA statistical analysis and SEM are shown. (**O**) Representatives images of MTCH2 KO MEFs transfected with MFN2-IYFFT-FLAG, MFN2-ACTA-FLAG, or MFN2-K109A-FLAG non-treated or treated for 16 h with GPATi. MFN2-IYFFT and MFN2-ACTA mutants were co-transfected with the ER marker Sec61-GFP to ensure the correct subcellular localization. Mitochondria were stained with αTOMM40 antibody. Scale bar 10 µm. (**P**) Mitochondrial aspect ratio analysis of cells presented in (**O**). At least 20 transfected cells were analyzed per condition. One-way ANOVA statistical analysis and SEM are shown. (**Q**) Western blot analysis in whole-cell lysates of GPAT3/4 expression in MTCH2 KO MEFs cells treated with NT-siRNA or with a combination of GPAT3 and 4 siRNAs; vinculin (VINC) serves as loading control. Data information: (**F**,**H**,**K**,**N**,**P**) ns, not significant, ***P < 0.001. Source data are available online for this figure.

First, we evaluated GPATs levels in heavy membranes (HM) and in ER-enriched light membrane (LM) fractions of WT, MTCH2 KO, and MFN2 KO MEFs. Western blot analysis showed that MTCH2 deletion had no effect on the expression levels and subcellular localizations of all four GPATs (Fig. 3B,C). In contrast, MFN2 deletion led to a large reduction in the levels of the ER-residents GPAT3/4 (Fig. 3B,C). Moreover, MFN2 KO MEFs consistently showed increased MTCH2 expression levels in comparison to WT MEFs (Fig. 3B,C). Both changes in GPATs3/4 and MTCH2 expression levels were rescued upon stable reintroduction of WT MFN2 (Fig. EV3A). As previously reported (de Brito and Scorrano, 2008; Casellas-Díaz et al, 2021), we detected MFN2 expression in the LM fraction of WT and MTCH2 KO cells, which was devoid from other OMM proteins such as TOMM40, MTCH2, and GPAT1/2 (Fig. 3B,C); nevertheless MTCH2 deletion had no effect on the levels or subcellular localization of MFN2.

Next, we investigated how LPA synthesis impacts mitochondrial fusion enforced by MTCH2 or by MFN2. To inhibit LPA synthesis, we used FSG67 (GPATi (Wydysh et al, 2009; Kuhajda et al, 2011)), which was designed to inhibit GPAT1 activity but was also shown to inhibit GPAT2 and GPAT3 (Kuhajda et al, 2011; Clemens et al, 2019). WT MEFs exposed to GPATi (at a concentration that does not affect cellular viability) (McFadden et al, 2014), elicited mitochondrial fragmentation with complete penetrance, which was reversible after washing the inhibitor (Fig. 3D,E). To assess if mitochondrial fragmentation caused by GPATi treatment was due to a decrease in LPA and not to a collateral stress affecting the mitochondrial dynamics machinery, we investigated the expression levels of several of these proteins in WT, MTCH2 KO, and MFN2 KO MEFs, and found that GPATi treatment did not affect the levels or the localization of MTCH2, DRP1, OPA1, and GPATs, but did elicit a small increase in the levels of MFN2 (Fig. EV3B). Overall, these results suggest that the mitochondrial fragmentation we observed was most likely due to inhibition of GPATs activity and not to an imbalance in mitochondrial dynamics proteins.

If MTCH2 modulates LPA to enforce mitochondrial fusion (Labbé et al, 2021), then inhibiting GPATs activity would likely impair this effect. To evaluate this, we overexpressed MTCH2-GFP in WT and MFN2 KO cells treated with GPATi. Unexpectedly, MTCH2-GFP overexpression elicited mitochondrial elongation in the presence of GPATi (Fig. 3F–H). To confirm these results orthogonally, we generated MTCH2 KO HEK293T human cells (Fig. EV3C), that displayed extensive mitochondrial fragmentation (Fig. EV3D–F). Also, treatment of WT HEK293T cells with GPATi elicited mitochondrial fragmentation, which was reversible upon washing the inhibitor (Fig. EV3G,H). Similar to the results obtained in MEFs, MTCH2-GFP overexpression elicited mitochondrial elongation in WT HEK293T cells treated with GPATi (Fig. EV3I–K). These results confirm that inhibition of GPATs activity is insufficient to block MTCH2’s ability to enforce mitochondrial fusion, and suggest that if MTCH2 funnels LPA it might not require high levels of this lipid to perform this function.

Next, we evaluated whether inhibiting LPA synthesis would have an effect on MTCH2-independent mitochondrial fusion. For this purpose, we overexpressed MFN2 in MTCH2 KO MEFs, control, or treated with GPATi. Inhibition of LPA synthesis completely impaired MFN2’s ability to enforce mitochondrial elongation in MTCH2 KO MEFs and HEK293T (Figs. 3I–K and EV3L–N). In line with these results, all three MFN2 mutants also lost their ability to enforce MTCH2-independent mitochondrial fusion in the presence of GPATi (Fig. EV3O,P). Previous evidence shows that mitochondrial GPATs and not ER-GPATs are required to sustain mitochondrial fusion (Ohba et al, 2013). Nevertheless, the large decrease in ER-GPATs levels in MFN2 KO, and the ability of MTCH2 to specifically enforce mitochondrial fusion in MFN2 KO cells suggested that ER-synthetized LPA might contribute to mitochondrial dynamics and cross-talk between MFN2 and MTCH2. To test this hypothesis, we silenced GPAT3 and 4 in MTCH2 KO MEFs (Fig. EV3Q) and overexpressed MFN2. Similar to the results obtained with GPATi, reducing GPATs levels in the ER compartment impaired MFN2’s ability to enforce mitochondrial fusion in MTCH2 KO MEFs (Fig. 3L–N). In summary, our results are consistent with the idea that in the absence of MTCH2, silencing ER-GPATs or pharmacological inhibition of LPA synthesis is epistatic to fusion enforced by MFN2 overexpression.

### MTCH2 cooperates with MFN2 and LPA synthesis to sustain mitochondrial fusion under cellular stress conditions

**Figure 4:**
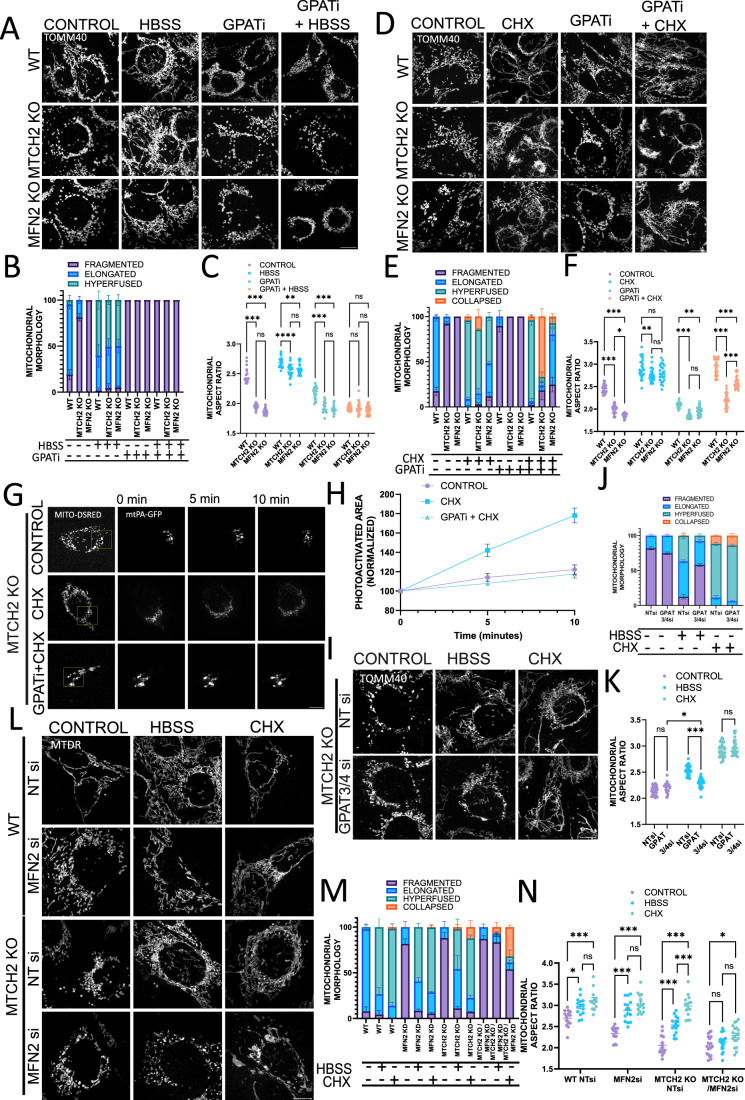
MTCH2 cooperates with MFN2 and LPA synthesis to sustain mitochondrial fusion under cellular stress conditions. (**A**) Representatives images of WT, MTCH2 KO, and MFN2 KO MEFs either non-treated (control), HBSS treated for 4 h, treated with GPATi for 16 h, or pretreated for 16 h with GPATi and then treated with HBSS for 4 h in the presence of the inhibitor. Mitochondria were stained with αTOMM40 antibody. Scale bar 10 µm. (**B**) Classical classification of mitochondrial network morphology. Results are presented as average and SEM of three separate experiments; at least 20 cells were analyzed per condition. (**C**) Mitochondrial aspect ratio analysis. At least 25 fields were analyzed per condition. One-way ANOVA statistical analysis and SEM are shown. (**D**) Representatives images of WT, MTCH2 KO, and MFN2 KO MEFs either non-treated (control), treated with 10 μM CHX for 4 h, treated with GPATi for 16 h, or pretreated for 16 h with GPATi and then treated with 10 μM CHX for 4 h in the presence of the inhibitor. Mitochondria were stained with αTOMM40 antibody. Scale bar 10 µm. (**E**) Classical classification of mitochondrial network morphology. Results are presented as average and SEM of three separate experiments; at least 20 cells were analyzed per condition. (**F**) Mitochondrial aspect ratio analysis. At least 25 fields were analyzed per condition. One-way ANOVA statistical analysis and SEM are shown. (**G**) Mitochondrial fusion assay. MTCH2 KO MEFs were co-transfected with mt-PA-GFP and mito-dsRed (both constructs are targeted to the mitochondrial matrix). After transfection cells were either left untreated, treated with 10 μM CHX for 4 h, or pretreated for 16 h with GPATi and then treated with 10 μM CHX for 4 h in the presence of the inhibitor. Yellow box indicates the initially photoactivated area. After photoactivation, the GFP signal was followed for 10 min. (**H**) Quantification of mitochondrial fusion. Eight cells were analyzed in each condition, the GFP fluorescent area was calculated on time 0, 5, and 10 min after photoactivation, and the area was then normalized to the initially photoactivated area. Each data point shows average and SEM. (**I**) Representatives images of MTCH2 KO MEFs treated with non-targeting (NT) siRNA or a combination of GPAT3 and GPAT4 siRNAs, control or treated with HBSS for 4 h, or treated with 10 μM CHX for 4 h. Mitochondria were stained with αTOMM40 antibody. Scale bar 10 µm. (**J**) Classical classification of mitochondrial network morphology. Results are presented as average and SEM of three different experiments, and at least 30 cells were analyzed per condition. (**K**) Mitochondrial aspect ratio analysis. At least 25 fields cells were analyzed per condition. One-way ANOVA statistical analysis and SEM are shown. (**L**) Representative images of WT and MTCH2 KO MEFs treated with non-targeting (NT) siRNA or with MFN2 siRNA, in control conditions, treated with HBSS for 4 h or treated with 10 μM CHX for 4 h. Mitochondria were labeled with MTDR. Scale bar 10 µm. (**M**) Classical classification of mitochondrial network morphology. Results are presented as average and SEM of three different experiments, and at least 30 cells were analyzed per condition. (**N**) Mitochondrial aspect ratio analysis. At least 25 fields were analyzed per condition. One-way ANOVA statistical analysis and SEM are shown. Data information: (**C**,**F**,**K**,**N**) ns, not significant, *P < 0.05, **P < 0.01, ***P < 0.001. Source data are available online for this figure.

To further understand the interplay between MTCH2, MFN2, and LPA synthesis in mitochondrial fusion, we choose to enforce mitochondrial hyperfusion using two different stresses: (1) inducing amino-acid deprivation by incubating cells in HBSS media, and (2) blocking protein translation using cycloheximide (CHX) (Tondera et al, 2009; Gomes et al, 2011; Rambold et al, 2011). A previous report indicated that MTCH2 is required for mitochondrial fusion in response to amino-acid deprivation (Labbé et al, 2021). In contrast, we now demonstrate that MTCH2 KO MEFs and MFN2 KO MEFs elongate their mitochondria following amino-acid deprivation induced by HBSS, yet both knockout cell lines showed a slightly reduced degree of elongation and hyperfusion as compared to WT MEFs (Fig. 4A–C).

**Figure EV4:**
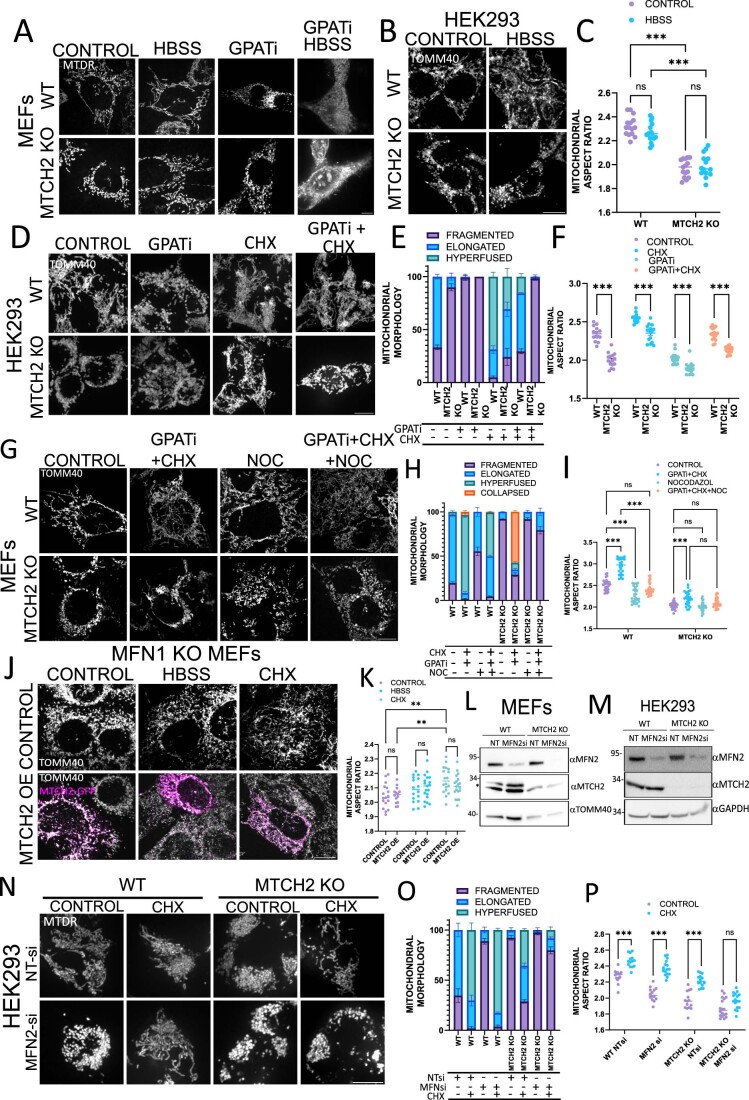
MTCH2 cooperates with MFN2 and LPA synthesis to sustain mitochondrial fusion under cellular stress conditions. (**A**) Representatives images of WT and MTCH2 KO MEFs either non-treated (control), treated with HBSS for 4 h, treated with GPATi for 16 h, or pretreated for 16 h with GPATi and then treated with HBSS for 4 h in the presence of the inhibitor. Mitochondria were labeled with MTDR. Scale bar 10 µm. (**B**) Representatives images of WT and MTCH2 KO HEK293T cells non-treated (control) or treated with HBSS for 4 h. Mitochondria were stained with αTOMM40 antibody. Scale bar 10 µm. (**C**) Mitochondrial aspect ratio analysis. At least 20 fields were analyzed per condition. One-way ANOVA statistical analysis. (**D**) Representatives images of WT and MTCH2 KO HEK293T cells either non-treated (control), treated with GPATi for 16 h, treated with 10 µM CHX for 4 h or pretreated for 16 h with GPATi and then treated with 10 µM CHX for 4 h in the presence of the inhibitor. Scale bar 10 µm. (**E)** Classical classification of mitochondrial network morphology. Results are presented as average and SEM of three different experiments, and at least 30 cells analyzed per condition. (**F**) Mitochondrial aspect ratio analysis. At least 20 fields were analyzed per condition. One-way ANOVA statistical analysis and SEM are shown. (**G**) Representatives images of WT and MTCH2 KO MEFs either non-treated (control), pretreated for 16 h with GPATi and then treated with 10 µM CHX for 4 h in the presence of the inhibitor, treated with 5 µM nocodazole (NOC) for 4 h or pretreated for 16 h with GPATi and then treated with 5 µM NOC and 10 µM CHX for 4 h in the presence of the inhibitor. Mitochondria were labeled with αTOMM40 antibody. Scale bar 10 µm. (**H**) Classical classification of mitochondrial network morphology. Results are presented as average and SEM of three different experiments, and at least 30 cells analyzed per condition. (**I**) Mitochondrial aspect ratio analysis. At least 25 fields were analyzed per condition. One-way ANOVA statistical analysis and SEM are shown. (**J**) Representatives images of MFN1 KO MEFs control or overexpressing MTCH2-GFP in control conditions or treated for 4 h with HBSS or CHX. Mitochondria were stained with αTOMM40 antibody. Scale bar 10 µm. (**K**) Mitochondrial aspect ratio analysis. At least 20 cells were analyzed per condition. One-way ANOVA statistical analysis and SEM are shown. (**L**) Western blot analysis of MFN2 in WT and MTCH2 KO MEFs treated with NT-siRNA or with siRNA targeting MFN2 expression, and TOMM40 serves as loading control. *Depicts a non-specific band. (**M**) Western blot analysis of MFN2 in WT and MTCH2 KO HEK293T cells treated with non-targeting (NT) siRNA or with MFN2 siRNA. GAPDH serves as a loading control. (**N**) Representative images of WT and MTCH2 KO HEK293T cells treated with non-targeting (NT) siRNA or with MFN2 siRNA non-treated or treated with CHX for 4 h. Mitochondria were labeled with MTDR. Scale bar 10 µm. (**O**) Classical classification of mitochondrial network morphology. Results are presented as average and SEM of three different experiments, and at least 30 cells analyzed per condition. (**P**) Mitochondrial aspect ratio. At least 20 fields were analyzed per condition. One-way ANOVA statistical analysis and SEM are shown. Data information: (**C**,**F**,**I**,**K**,**P**) ns, not significant, **P < 0.01, ***P < 0.001. Source data are available online for this figure.

In line with previous evidence (Labbé et al, 2021), we found that none of the three cell lines were able to elongate their mitochondria in response to HBSS in the presence of GPATi (Fig. 4A–C). Moreover, mitochondria of WT and MTCH2 KO MEFs treated with a combination of HBSS and GPATi did not uptake mitotracker deep red, evidencing loss of mitochondrial membrane potential (Fig. EV4A). On the other hand, HBSS treatment had no effect on mitochondrial morphology in WT and MTCH2 KO HEK293T cells (Fig. EV4B,C), suggesting that the mitochondrial response to this stress and the requirement for MTCH2 may vary among different cell lines. Overall, our data suggest that MTCH2 is not essential for HBSS-induced mitochondrial fusion in MEFs, while mitochondrial fusion induced by HBSS requires LPA synthesis.

Since LPA synthesis is essential for HBSS-induced mitochondrial fusion, we could not use this stimulus to discriminate a possible differential contribution of LPA, MTCH2, and MFN2 to mitochondrial plasticity. Thus, we turned to SIMH induced by CHX treatment (Tondera et al, 2009), and found, in agreement with previous studies (Tondera et al, 2009; Labbé et al, 2021), that WT, MTCH2 KO, and MFN2 KO respond to this stress. WT and MFN2 KO MEFs showed mitochondria hyperfusion/elongation in response to CHX in the presence of GPATi (Fig. 4D–F). In contrast, GPATi treatment largely impaired the response of MTCH2 KO MEFs to CHX, eliciting clumping and collapse of the mitochondrial network (Fig. 4D–F), with similar results obtained in MTCH2 KO HEK293T cells (Fig. EV4D–F).

To understand if the collapsed mitochondrial network observed upon treatment of MTCH2 KO MEFs with CHX and GPATi is composed of interconnected or fragmented mitochondria we utilized two different approaches: the mitochondrial fusion assay and relaxation of the mitochondrial network with nocodazole (Smirnova et al, 2001). Mitochondrial fusion assay was performed using a photoactivatable-GFP targeted to the mitochondrial matrix (mt-PA-GFP). MTCH2 KO MEFs were treated with either CHX or a combination of CHX and GPATi and their mitochondrial fusion activity was compared to non-treated cells. In CHX-treated MTCH2 KO cells, 10 min after photoactivation the GFP signal was spread throughout most of the mitochondrial network, while non-treated MTCH2 KO MEFs as well as treated with GPATi and CHX showed almost no spreading of the photoactivated GFP (Fig. 4G,H).

Next, MTCH2 KO MEFs exposed to GPATi were treated with the microtubule polymerization inhibitor nocodazole before inducing SIMH. Interestingly, pretreatment of MTCH2 KO MEFs with nocodazole abolished the mitochondrial clumping caused by the combination of CHX and GPATi, and revealed a fragmented network composed mostly of short-tubulated mitochondria (Fig. EV4G–I). These results suggest that part of the stress experienced by MTCH2 KO mitochondria exposed to CHX and GPATi can be alleviated by blocking microtubule polymerization. Importantly, even though the network is still highly fragmented, mitochondrial morphology has changed from large rounded and fragmented to short and tubulated mitochondria, indicating that loss of both MTCH2 and LPA synthesis only impairs part of the mitochondrial architectural changes induced by SIMH. Altogether, these results suggest that WT and MFN2 KO cells do not require full GPAT activity for SIMH, whereas MTCH2 KO cells are sensitized to CHX treatment in the presence of GPATi.

We showed above that silencing ER-GPATs in MTCH2 KO blocks mitochondrial fusion enforced by MFN2 overexpression, thus, we evaluated if silencing ER-resident GPATs would similarly impact MTCH2 KO response to stress. To investigate this, we enforced mitochondrial fusion with HBSS or CHX in MTCH2 KO GPAT3/4 KD cells, and found a significant defect in the response to HBSS-induced mitochondria elongation, and no impact on the response to CHX (Fig. 4I–K). These results demonstrate that in MTCH2 KO cells, ER-GPATs contribute to HBSS-induced mitochondrial fusion and suggest a more important role for GPATs1 and 2 in the synthesis of LPA required for MTCH2-independent mitochondrial fusion induced by SIMH.

We showed that MTCH2 requires MFN1 to enforce mitochondrial fusion in steady state conditions, but it could perhaps lose this requirement under conditions of stress-induced mitochondrial fusion. To test this idea, we evaluated if overexpression of MTCH2 in combination with HBSS or CHX treatment could by-pass the requirement of MFN1 to enforce mitochondrial elongation. MFN1 KO MEFs treated with HBSS showed no significant response to this stress and remained fragmented, while CHX caused a small increase in the aspect ratio analysis (Fig. EV4J,K), representative of short-tubulated mitochondria. MTCH2 overexpression did not improve mitochondrial elongation in combination with either HBSS or CHX treatments, further confirming the requirement of MFN1 expression for stress- induced mitochondrial fusion and for MTCH2-enforced mitochondrial fusion.

Our results suggest the existence of two separate pathways: one independent of MTCH2, which can be compensated by MFN2 overexpression and requires LPA synthesis to sustain its plasticity, and the second pathway independent of MFN2, which can be compensated by MTCH2 overexpression and does not require full GPAT activity to sustain mitochondrial potential to fuse. If these two pathways cooperate, then loss of both pathways should largely impair mitochondrial fusion. To test this idea, MFN2 was silenced in WT MEFs and MTCH2 KO MEFs (Fig. EV4L), and mitochondrial fusion was enforced by HBSS or CHX. Similar to MFN2 KO MEFs, MFN2 silencing in WT MEFs resulted in mitochondrial fragmentation and did not impair mitochondrial fusion in response to either HBSS or CHX treatment (Fig. 4L–N). Importantly, both MTCH2 KO/MFN2 KD MEFs and MTCH2 KO/MFN2 KD HEK293T showed aberrant or no mitochondrial plasticity post HBSS or CHX treatment (Figs. 4L–N and EV4M–P, respectively). Overall, our results suggest that expression of either MTCH2 or MFN2 is required to sustain mitochondrial plasticity under stress conditions, while loss of both proteins impairs stress-induced mitochondrial fusion.

How does MTCH2 compensate for MFN2’s absence? Previous work reported that MTCH2 plays a role in modulating the pro-fusion lipid LPA (Labbé et al, 2021). In this context, we show that: (1) based on the response to CHX, in cells expressing endogenous MTCH2 (WT and MFN2 KO cells), inhibiting GPATs activity does not impair mitochondrial plasticity; (2) mitochondria of WT and MFN2 KO cells overexpressing MTCH2 resist fragmentation induced by GPATi, and (3) based on the response to CHX and to MFN2 overexpression, loss of MTCH2 unmasks the requirement of LPA synthesis for mitochondria to retain plasticity. These results suggest a role for MTCH2 in modulating mitochondrial dynamics at the lipidic level. Recently it was postulated that MTCH2 works as an OMM lipid scramblase (preprint: Bartoš et al, 2023, preprint: Li et al, 2023). Thus, it is tempting to speculate that MTCH2 regulates the OMM lipid composition by scrambling lipids between the outer and inner leaflets.

How are these pathways intertwined and how do they cooperate under steady state conditions? If MTCH2 deletion elicits mitochondria to be more susceptible to a decrease in LPA, then specifically MFN2, and not MFN1 overexpression, might enforce mitochondrial fusion in part by compensating for this susceptibility. It is possible that this alternative or secondary role of MFN2 is connected to sustaining mitochondrial lipid homeostasis, by transferring lipids between ER and mitochondria, which affect or are directly involved in mitochondrial dynamics. In line with this idea, previous reports show that MFN2 deletion affects phospholipid synthesis and their transfer to the mitochondria (Chung et al, 2019; Hernández-Alvarez et al, 2019). In addition, a most recent publication reports the existence of two novel splicing variants of MFN2 located in the ER compartment that regulate its morphology and modulate lipid transfer as well as tethering (Naón et al, 2023).

Here we show that MFN2 deletion elicits a significant decrease in the levels of ER-resident GPAT3 and 4, an effect that could possibly be explained by either the loss of ER integrity or that of ER-mitochondrial contact sites caused by the absence of these ER-resident isoforms of MFN2 (Naón et al, 2023). Interestingly, MFN2 KO MEFs showed an increase in MTCH2 expression levels, which is not sufficient to induce mitochondrial elongation in steady state conditions, but it might reflect a cross-talk between the two pathways. It is conceivable that the increase in MTCH2 protein levels represents an attempt to compensate for the poor ER-GPATs activity, or inadequate lipid synthesis/flux moving from the ER to the mitochondria. Thus, MTCH2 could act as both as a sensor and a modulator of OMM lipid composition, which possesses the ability to alter the morphology and dynamic behavior of mitochondria and steady-state and stress (e.g., apoptosis) conditions.

## Methods

### Cell lines and culture conditions

MFN1 KO, MFN2 KO, and MFN1/2 DKO MEFs, originally published by Prof. David Chan (Chen et al, 2003), were a gift from Prof. Gyorgy Hajnoczky. MTCH2 KO MEFs were generated from *MTCH2*^*F/F*^ primary MEFs, which were prepared from 11- to 13-day-old embryos and transformed with SV40 (Zaltsman et al, 2010). *MTCH2*^*F/F*^ MEFs were transfected with Cre-GFP, and several MTCH2 KO clones were obtained, showing similar mitochondrial fragmentation, and one of these clones was used for this study. The MTCH2 CRISPR KO cell line was generated in HEK293T cells. Guides were designed using CHOP web application (https://chopchop.cbu.uib.no/). The MTCH2 guides-RNAs were: F-CACCAGCACTTTCACGTACATGAGGT and R-TAAAACCTCATGTACGTGAAAGTGCT. Cells were transfected with guides and selected with puromycin; several MTCH2 KO clones were obtained, showing similar mitochondrial fragmentation, and one of these clones was used for this study. Cells were grown in high glucose DMEM (cat. # 41965, Thermo Fisher), 10% FBS (cat. # 12657, Gibco), supplemented with 2 mM L-glutamine (cat. # 03-020, Biological Industries), 1 mM sodium pyruvate (cat. # 03-042, Biological Industries) and antibiotics (cat. # 03-031, Biological Industries). For HBSS starvation, cells were washed 3 times with DMEM (cat. # A14430, Thermo Fisher), and then incubated for 4 h with HBSS (cat. # 02-015, Biological Industries). Additional reagents used in this study: CHX (10 µM; cat. # 01810, Millipore-Sigma), Nocodazol (5 µM; cat. # SML1665, Millipore-Sigma), and FSG67 (100 µM; cat. # 1158383-34-6, Focus Biomolecules). All cell lines were regularly tested for Mycoplasma contamination using the MycoAlert® PLUS Mycoplasma Detection Kit.

### Antibodies

Mouse monoclonal antibodies were used for: MYC (SC-40, Santa Cruz), MFN2 (clone XX-1, Santa Cruz), OPA1 (BD612606, BD Transduction Laboratories), Complex III (UQCRC2) (Ab14745, Abcam), V5 tag (R960-25, Invitrogen), GAPDH (sc-47724, Santa Cruz), Tubulin (sc-5286, Santa Cruz), IP3R3 (Clone 2, BD Transduction Laboratories), FLAG (M2, Sigma), Vinculin (V4505, Sigma) and Complex V (ATP5bMS-503, MitoScience). Rabbit antibodies were used for: human MTCH2 (Ab174921, Abcam), MFN1 (Ab104274, Abcam), DRP1 (clone D8H5 CS5391, Cell Signaling), MFF (17090-AP, Proteintech), MID49 (16413-AP, Proteintech), TOMM20 (SC-11418, Santa Cruz), TOMM40 (18409-AP, Proteintech), TOMM70 (14528-1-AP, Proteintech), GPAT1 (GPAM) (HPA06009790, Sigma), GPAT2 (HPA036841, Sigma), GPAT3 (AGPAT9) (20603-AP, Proteintech), GPAT4 (AGPAT6) (16762-AP, Proteintech), FLAG (F7425.2MG, Sigma), and MYC (2278, Cell Signaling). The MFN1 polyclonal antibody used for BN-GE was a kind gift from Prof. Richard Youle. Anti-MTCH2 polyclonal antibodies that recognize the mouse protein were generated in-house (Grinberg et al, 2005). The calnexin antibody (ab140818, Abcam) raised in chicken was also used in this study. Secondary antibodies, donkey anti-mouse or donkey anti-rabbit coupled to Cy2, Cy3, or Cy5 dyes were purchased from Molecular Probes, In., and secondary antibodies, HRP-conjugated donkey anti-mouse, donkey anti-rat, and donkey anti-rabbit were purchased from Amersham GE.

### Expression plasmids, transfections, and gene silencing

The MTCH2-GFP plasmid was generated in our laboratory and was previously described (Grinberg et al, 2005). MTCH2-K25E-GFP and MTCH2-D189R-GFP were generated by site directed mutagenesis using the QuickChange Lightning kit (210518, Agilent), using the following primers (MTCH2-K25E F-CCGCTCATGTACGTGGAAGTGCTCATCCAGG, MTCH2-D189R F-CCTCGCCTTCTAGGTCGCATCCTTTCTTTGTGGC. N-terminally EGFP-tagged MFN1 plasmid (MFN1-GFP) was generated by cloning the full-length ORF of mouse MFN1 (accession number AY174062) into a pEGFP-C1 expression vector (Clonetech). Untagged MFN1 isoform 1 (accession number NM_033540) was expressed using as a backbone the pCDNA5.1 vector and co-transfected with a cytosolic GFP construct. MFN2-MYC (23213), AKAP-mtagBFP2-UBC6 (105007), and Sec61b-GFP (121159) were purchased from Addgene. MFN2-IYFFT-FLAG, MFN2-ACTA-FLAG, and MFN2-K109A were obtained from Prof. Heidi McBride, and MFN2-ACTA-V5 was obtained from Prof. Luca Scorrano. mt-PA-GFP as well as mito-DSRED were provided by Prof. Gyorgy Hajnoczky. For MEFs transfection, Lipofectamine 3000 (Thermo Fisher Scientific) was used, and for HEK293 transfection polyJET (MTI; Global Systems) was used according to the manufacturer’s instructions. Most experiments were carried out either 24 or 48 h post transfection. On-TARGET plus Smart Pool (Dharmacon) siRNAs were used: MTCH1 mouse (L-050263-01), MFN2 human (L-012961-00), MFN2 mouse (L-046303-00), GPAT3 mouse (L-052255-01), GPAT4 mouse (L-058095-01) and non-targeting (NT) control (D-001810-10). Gene silencing was performed by transfecting cells using Lipofectamine RNAi MAX (Invitrogen, 13778150), according to the manufacturer’s instructions, and 72 h post transfection gene silencing was confirmed by Western blot analysis or by qRT-PCR.

### Sample preparation and Western blot analysis

Whole-cell lysates were obtained by lysing cell pellets with RIPA buffer for 15 min on ice. Then samples were centrifugated, and protein concentration was estimated. Subcellular fractionation was performed as described previously (Zaltsman et al, 2010). Briefly, cells were collected by scraping, centrifuged, and washed with ice-cold PBS. For heavy membrane purification, cells were resuspended in mitochondrial buffer (MB, 200 mM mannitol, 70 mM sucrose, 1 mM EGTA, 10 mM HEPES pH 7.5) and homogenized on ice with a rotating Dounce Teflon homogenizer at 2100 rpm with 40 strokes, and cell debris and unbroken cells were pelleted at low-speed centrifugation (900 × *g*, 10 min, 4 °C). The supernatant was then centrifuged at 8000 × *g*, 10 min, 4 °C to obtain in the pellet the heavy membrane (HM) fraction enriched in mitochondria. To purify the light membrane (LM) fraction, the supernatant of the HM fraction was centrifuged at 20,000 × *g*, 30 min 4 °C, and the supernatant was centrifuged again at 100,000 × *g*, 1 h 4 °C and the pellet was resuspended in mitochondrial buffer. Protein concentration was assessed by Bradford, and equal amounts of protein were separated by SDS-PAGE. Size-separated denaturized samples were then transferred to either PVDF or nitrocellulose membranes, blocked in milk, incubated with different primary antibodies followed by peroxidase-conjugated anti-rat, anti-mouse, or anti-rabbit, followed by ECL treatment (Amersham).

### Blue native gel electrophoresis (BN-GE)

For isolation of native mitochondrial protein complexes we proceeded as previously described (Gomes et al, 2011), with small modifications. Briefly, 200 μg of mitochondria were resuspended in 50 μl of native loading buffer (Invitrogen) containing 4% digitonin (Sigma) and protease-inhibitor cocktail (Sigma). After incubating 10 min at 4 °C, the lysate was cleared by centrifugation at 22,000 × *g* for 30 min at 4 °C. 2.5 μl of native additive G250 5% (Invitrogen) was added to the supernatant and 100 μg of protein was loaded onto a 3–12% native gel (NativePAGE™ (Invitrogen)). Native complexes were size-separated for 30 min at 150 V with Dark Blue cathode buffer and 90 min at 250 V with Light Blue cathode buffer (Invitrogen). Finally, the native protein complexes were transferred to a PVDF membrane using Native Transfer (Novex) and Western blotted with different antibodies.

### Quantitative real-time reverse transcript PCR

To confirm silencing of MTCH1 by qPCR total RNA was extracted using NucleoSpin RNA kit (Macherey-Nagel #740955) and retrotranscribed to cDNA using SuperScript IV VILO (Thermo Fisher). qRT-PCR was performed using the next set of primers: Mtch1-f—GCTGCTGGGATTCTTCGTTG, Mtch1-r—ACAGCCCCACAAGAAAACCA, Gapdh-f—TGTCCGTCGTGGATCTGAC, Gapdh-r—CCTGCTTCACCACCTTCTTG, Hprt-f—TCCTCCTCAGACCGCTTTT, Hprt-r—CCTGGTTCATCATCGCTAATC. The amplification reaction was quantified by calculating Δ and 2^ΔCT^ values. GAPDH and HPRT were used as housekeeping controls.

### Microscopy studies

Confocal images were acquired in a Nikon ECLIPSE Ti2-E inverted microscope with CSW-1 spinning disc system (Yokogawa), with a x100 CFI Plan Apo100x oil (na 1.45 wd 0.13 mm), equipped with temperature and CO_2_ control. Images were acquired using a Photometrics prime 95b camera and Nikon Elements software. Lasers 405/488/561/638 nm were used for DAPI/BFP, GFP, Cy3 and Cy5/MTDR, respectively. Live imaging time-lapse experiments were acquired in a wide field Olympus IX83 microscope (Olympus, Japan) equipped with ×100 oil immersion objective Plan Apo100x oil (na 1.45 wd 0.13 mm), Orca Flash 4.0 camera (Hamamatsu Photonics, Japan) and a LED light source (CoolLED, UK). Images were acquired using cellSense software and preprocessed using the online deblur tool.

### Immunofluorescence

For immunofluorescence studies, cells were seeded 24 h before the experiment in 13 mm sterile glass coverslips (Thermo Scientific). Cells were then fixed with 6% PFA for 15 min at 37 °C. PFA was quenched by incubating the samples for 10 min in 50 mM NH_4_Cl_2_ in PBS. After washing, cells were permeabilized with 0.1% triton X-100 in PBS for 20 min and then blocked for 30 min at room temperature with 5% BSA, 0.1% triton X-100 in PBS. Cells were then probed with primary antibodies, washed, and stained with secondary antibodies conjugated to Cy2, Cy3, or Cy5 (Molecular Probes, Inc.). After three additional washes with DDW, coverslips were mounted on slides using SlowFade Light Antifade kit (Molecular Probes, Inc.).

### Live imaging

For live cell imaging experiments, cells were seeded 24 h before the experiment. Cells were pre-incubated with 100 nM Mitotracker Deep Red (MTDR) (Thermo Fisher M22426) for 30 min. Prior to imaging, cells were washed and media was changed to imaging media (DMEM without phenol red, 10% FBS, 2 mM glutamine, 1 mM sodium pyruvate, and 20 mM HEPEs) and stabilized for an additional 30 min at 37 °C and 5% CO_2_. Cells were then imaged under temperature and CO_2_-controlled conditions.

### Classical classification of mitochondrial network morphology

Cells were classified unblinded according to the morphology of the mitochondrial network, into “Elongated” where >90% of mitochondria had tubular shape, “Fragmented” with >90% round mitochondria, “Hyperfused” for mitochondrial network highly reticular and interconnected, and “Collapsed” for mitochondrial network that shows clumping surrounding the perinuclear region of the cell. Data is presented as percentage of cells in each category, and standard error of the mean of three different experiments is calculated.

### Mitochondrial aspect ratio (AR)

To perform mitochondria AR analysis, images acquired under the same conditions were processed using background subtraction, thresholded (binarized), and then segmented using the Analyze Particles tool from FIJI (Schindelin et al, 2012). One representative experiment out of three replicas was analyzed by calculating average mitochondrial AR per cell or average mitochondrial AR per field of view. At least 15 cells or fields were analyzed in each condition. Statistical analysis was performed by one-way ANOVA with multiple comparisons, unless otherwise mentioned.

### MFN1-mediated mitochondrial tethering time

WT and MTCH2 KO cells were transfected with MFN1-GFP, stained with MTDR, and several cells (~30 fields) were imaged for a period of 5 min, taking one image every 3 s. We located and isolated 23 WT and 18 MTCH2 KO different MFN1-GFP tethering events, which we defined as those events where mitochondria remained visibly tethered through an MFN1-GFP foci for more than 15 s, trying to avoid “kiss and run” type of interactions (Liu et al, 2009). We utilized the Particle Tracking of the MOSAIC plugin on FIJI to track MFN1-GFP foci during the fusion attempts, and measured the time that the MFN1 foci spent in these tethering points.

### EM sample preparation and contact site analysis

Transmission electron microscopy. Cells were fixed with 4% paraformaldehyde, 2% glutaraldehyde in 0.1 M cacodylate buffer containing 5 mM CaCl_2_ (pH 7.4), then postfixed with 1% osmium tetroxide supplemented with 0.5% potassium hexacyanoferrate tryhidrate and potassium dichromate in 0.1 M cacodylate (1 h), stained with 2% uranyl acetate in water (1 h), dehydrated in graded ethanol solutions and embedded in Agar 100 epoxy resin (Agar Scientific Ltd., Stansted, UK). Ultrathin sections (70–90 nm) were viewed and photographed with a FEI Tecnai SPIRIT (FEI, Eindhoven, Netherlands) transmission electron microscope operated at 120 kV and equipped with a OneView Gatan Camera. To analyze ER-mitochondria contact sites by EM, we used the freehand drawing tool from FIJI software. We delimited only ER tubules within a 60 nm distance from the nearest mitochondrial surface, and delimited the mitochondrial perimeter. Then, we analyzed length and GAP of each contact site. We separated the contact site according to their GAP into three groups: from 0–15 nm, 15–30 nm, and 30–60 nm. Finally, we calculated the number of contacts per mitochondria, the average contact length, mitochondrial perimeter, total mitochondrial number, and ER-mitochondria contact coverage.

### Reconstitution of MFN2 in MFN2 KO MEFs

For stable reconstitution of WT MFN2 (NM_014874) we used FLAG N-terminal labeled human MFN2 in the retroviral backbone pBABE-GTW. MFN2 KO MEFs were transduced with retroviral particles generated in the packaging line HEK293 phoenix. An MFN2 KO polyclonal population expressing FLAG-MFN2 was obtained by puromycin selection. Expression of the rescue protein was confirmed by Western blot analysis.

### Mitochondrial fusion assay

MTCH2 KO MEFs were co-transfected with mt-PA-GFP construct and a mitochondrial matrix targeted DsRed fluorescent protein to identify transfected cells. 24 h post transfection cells were seeded in imaging plates and exposed to the different treatments. Transfected cells were photoactivated with 405 laser, and cells were imaged during a lapse of 10 min. The GFP positive area was measured at 0-, 5-, and 10-min post photoactivation, and normalized to time 0.

### Statistical analysis

*P* values were calculated by one-way ANOVA test, unless otherwise specified, with GraphPad prism statistical software.

## Supplementary information

Source Data Fig. 1

Source Data Fig. 2

Source Data Fig. 3

Source Data Fig. 4

Source Data EV Fig. 1

Source Data EV Fig. 2

Source Data EV Fig. 3

Source Data EV Fig. 4

Peer Review File

Expanded View Figures

## Data Availability

This study includes no data deposited in external repositories.
