# Supplementary material for: MTCH2 cooperates with MFN2 and lysophosphatidic acid synthesis to sustain mitochondrial fusion
Source: EMBO Rep. 2023 Dec 14;25(1):8. doi: 10.1038/s44319-023-00009-1 (PMC10897490; doi:10.1038/s44319-023-00009-1)
Supplement: Supplementary file 1 — Source Data Fig. 1 [file 44319_2023_9_MOESM1_ESM.pdf]

EMBO reports

MTCH2 cooperates with MFN2 and lysophosphatidic acid synthesis to sustain mitochondrial fusion

Andres Goldman, Michael Mullokandov, Yehudit Zaltsman, Limor Regev, Smadar Levin-Zaidman, and Atan Gross
DOI: 10.15252/embr.202357575

Corresponding author(s): Atan Gross (atan.gross@weizmann.ac.il) , Andres Goldman (andres.goldman@mcgill.ca)

Review Timeline:

Transfer from Review Commons:	1st Jun 23
Editorial Decision:	2nd Jun 23
Revision Received:	3rd Oct 23
Editorial Decision:	24th Oct 23
Revision Received:	27th Oct 23
Accepted:	7th Nov 23

Editor: Martina Rembold

Transaction Report: This manuscript was transferred to EMBO reports following peer review at Review Commons and The EMBO Journal.

Review #1

1. Evidence, reproducibility and clarity:

Evidence, reproducibility and clarity (Required)

In this manuscript, authors investigate the role of MTCH2 in mitochondrial morphology, in several conditions.
The authors showed compensatory effect of MFN2 and MTCH2 on stress induced mitochondria hyperfusion (SIMH) in HBSS or CHX treated condition. Since mitochondria hyperfusion upon CHX treatment is impaired in MTCH2 KO cells treated with a GPAT inhibitor, but not in MFN2 KO cells, authors suggest two modes of SIMH, one MTCH2 dependent, the second MFN2/LPA dependent.

This effect seems to be phenocopied in unstressed condition using overexpression system. Mitochondrial fragmentation in MFN2 KO cells can be recovered by MTCH2 overexpression, and vice versa. The fragmentation of mitochondria in MTCH2 KO MEF is reversed also by an ER-targeted MFN2, suggesting the importance of MFN2 ER localization. The authors also point out that MTCH2 KO have increased mitochondria-ER contacts.

Major comments

1. Each of the finding is interesting, but the results are not well discussed and logical links leading to the key conclusions are sometimes missing.

1-1) A previous report (Bahat et al., 2018) and this manuscript show that MFN2 OE can restore mitochondrial elongation in MTCH2 KO cells and MTCH2 OE can do the same in MFN2 KO cells. Based on these data, authors conclude that "MFN2 and MTCH2 compensate for each other's absence", but the compensation works only when they are overexpressed. On the other hand, upon HBSS or CHX treatment, MFN2 KO or MTCH2 KO cells have elongated/hyperfused mitochondria, but this is not observed in double deficient cells. In this case, MFN2 and MTCH2 show compensatory effects on mitochondria elongation. The authors believe the two conditions, unstressed&OE and stressed conditions, activating same molecular machineries, but it is not fully supported by the data. For example, in unstressed condition, MTCH2 OE can recover MFN2 KO but not of MFN1 KO, suggesting MTCH2-dependent mitochondria fusion requires MFN1, but it is not tested for stressed condition.
Furthermore, even if the machineries are common among stressed and unstressed

conditions, authors should discuss why the endogenous MFN2/MTCH2 expression is not enough to activate mitochondria fusion in MTCH2/MFN2 KO cells, respectively, and how the compensatory effects are activated upon HBSS or CHX treatment.

1-2) Authors found out that ER-targeted MFN2 can rescue the mitochondria fragmentation in MTCH2 KO MEF cells, but mitochondria-targeted MFN2 has a lower effect than Wt MFN2. (Fig 2A&B). This finding suggests that MTCH2 loss might impair MFN2 localization at the ER. The authors should investigate endogenous MFN2 localization in MTCH2 KO MEFs.

1-3) The analysis to test whether recovery of mitochondrial morphology by ER-targeted MFN2 in MTCH2 KO depends on LPA synthesis or not is missing (Fig 3G). Authors should examine whether mitochondrial elongation induced by ER-localized MFN2 in MTCH2 KO cells is impaired by the GPAT inhibitor.

1-4) In the discussion section, authors suggest that mitochondrial LPA would be a crucial factor for MFN2 dependent mitochondrial fusion. To test this hypothesis, authors should overexpress mitochondrial GPAT and evaluate its effect on mitochondrial morphology.

1-5) In the discussion section, authors indicated that ER-targeted MFN2 could recover mito-ER contacts leading to LPA flux from ER to mitochondria and mitochondria elongation in MTCH2 KO. However, MTCH2 KO itself already have more mito-ER contacts (Fig 2D-H), and an artificial linker fails to recover mitochondria fragmentation in MTCH2 KO cells (Fig S2C, D). Thus, increased number of contacts appears not sufficient to recover the phenotype. The authors should consider this point in the discussion.
2. Certain methods are not appropriate to support the stated conclusions.

2-1) Authors assess "mitochondria fusion" by evaluating mitochondrial morphology. The authors also describe mitochondrial clumping as a fusion-impaired phonotype (Fig 4A&B). Mitochondrial fusion should be evaluated using a PEG assay or a mtPA-GFP analysis.

2-2) In figure 2D-G, authors show that MTCH2 KO cells have more and longer mitochondria-ER contacts. The correct experiment is not to compare these cells to WT, but to KO reconstituted with MTCH2.

2-3) Since staining of MitoTracker depends on mitochondrial membrane potential, mitochondria with low potential would be invisible and excluded from the analysis. Authors should investigate mitochondrial morphology by immunostaining also in Fig

4D, S4A, D, and K.
3. Other points

3-1) There are some discrepancies with the previous Labbé et al article.:
Labbé et al. suggest that MTCH2 activity on mitochondria (from HCT116 cells) fusion is dependent on LPA, based on in vitro fusion assay. On the other hand, this manuscript shows that inhibition of LPA synthesis could not block MTCH2-induced mitochondria elongation in WT MEF and HEK293T cells.
MTCH2 KO HCT116 cells are resistant to HBSS- but sensitive to CHX-induced mitochondria elongation. In this manuscript, MTCH2 KO MEF cells are sensitive to both stimuli, and only when MTCH2- and MFN2-deficient MEF cells are resistant to both stimuli.
These discrepancies would be caused by difference of assay system or cell lines, but it is not clearly addressed.

Minor comments

1. Since authors use FSG67, an inhibitor against GPAT1, 2 and 3, knocking down of each of GPATs will improve the significance of this work.
2. Recently, a paper about MTCH2 is published (Guna et al., Science), which shows its insertase activity on tail anchored proteins. Authors should include this point of view in the discussion.

2. Significance:

Significance (Required)

Mitochondrial carrier homologue 2 (MTCH2/MIMP/SLC25A50) was found as a mitochondrial solute carrier family member but the substrates are unknown. MTCH2 has roles on apoptosis with Bid (Zaltsman et al., 2010, the authors' group), lipid homeostasis (Rottiers et al., 2017), and mitochondrial morphology (Bahat et al., 2018, the authors' group, and Labbé et al., 2021).

Stress induced mitochondria hyperfusion (SIMH) was reported in 2009 by Tondera et al.. Under stress condition, such as UV-C, cycloheximide (CHX), or actinomycin D treatment, hyperfused mitochondria were observed and the event was named as SIMH. They also showed that SIMH is dependent on L-Opa1, MFN1 and SLP-2, which is later found as Opa1 regulator, but not on MFN2, BAX/BAK.

In this manuscript, authors show compensatory effect of MFN2 and MTCH2 on SIMH in HBSS or CHX treated condition. This compensatory effect seems to be

reproduced in unstressed condition: mitochondrial fragmentation in MFN2 KO cells can be recovered with MTCH2 overexpression, and vice versa. Authors indicate that LPA synthesis and its mitochondrial localization would be crucial for MFN2 dependent fusion.
The compensatory effect of MFN2 and MTCH2 is potentially interesting for a large audience in multiple cell biology fields (mitochondrial biology, ER-mitochondria contact sites, lipid biology).

Expertise: Mitochondria; fusion/fission; ER-mitochondria contact sites

3. How much time do you estimate the authors will need to complete the suggested revisions:

Estimated time to Complete Revisions (Required)

(Decision Recommendation)

Between 3 and 6 months

4. *Review Commons* values the work of reviewers and encourages them to get credit for their work. Select 'Yes' below to register your reviewing activity at Web of Science Reviewer Recognition Service (formerly Publons); note that the content of your review will not be visible on Web of Science.

Web of Science Reviewer Recognition

Yes

Review #2

1. Evidence, reproducibility and clarity:

Evidence, reproducibility and clarity (Required)

In this manuscript, Goldman et al examine the role of the mitochondrial protein MTCH2 in mitochondrial fusion. Using a combination of overexpression of MTCH2, MFN1, and MFN2 in respective knockout cells, pharmacological inhibition of GPATs, and stress-induced mitochondrial hyperfusion, the authors explore the relationship between MTCH2, the mitofusins, and LPA levels. These experiments are particularly interesting in light of recent work by Labbe et al which propose that MTCH2 coordinates with LPA to promote mitochondrial fusion. The authors find a key difference from the work of Labbe et al, presenting data suggesting that the role of MTCH2 in starvation-induced mitochondrial hyperfusion varies depending on cell line. They also make the interesting observation that overexpression of MTCH2 is able to bypass GPATi treatment and promote mitochondrial fusion. The authors instead utilize differentially targeted MFN2 to argue that ER-localized MFN2 promotes mitochondrial fusion cooperatively with LPA synthesis. Based on these and other results, they conclude that MTCH2 works with MFN1 to stimulate fusion in a pathway parallel to MFN2 and LPA.

While the assays are mostly well performed and the findings will be of interest to those in the mitochondrial dynamics field, many of the conclusions drawn by the authors are not supported by the experiments shown. This in turn causes the the text to suffer from a lack of clarity and made the logic of the manuscript hard to follow. Additional controls are also needed to draw robust conclusions from MFN2 targeting and MFN1 overexpression experiments. Finally, the rigor of quantification should be clarified and expanded to all assays in the manuscript.

Specific points

1. The authors should comment on recently published work in Science that MTCH1 and likely MTCH2 are outer membrane insertases. The data in the manuscript seem consistent with the model that an undetermined protein may be poorly inserted in a MTCH2 knockout leading to reduced fusion activity mediated by MFN1. Could this explain how MTCH2 overexpression selectively restores fusion to MFN2 KO cells?
2. The authors make the claim that "MFN2 and MTCH2 compensate for each other's absence" though this is not supported by their data. For example, MFN2 expression is not affected in an MTCH2 KO but cannot compensate to promote fusion. Rather, the authors find that MTCH2 and MFN2 are capable of promoting fusion when overexpressed in the absence of the other.
3. The authors rely heavily on claims that tagged MFN1 and MFN2 are fully functional, but is it possible that MFN1-GFP is only partially functional? Does untagged MFN1 overexpression cause mitochondrial fusion in a MTCH2 KO? The quantification of this is done only with aspect ratio, and not by categorization of mitochondrial morphology (Fig. S1). The authors should present both analyses in this

and all other experiments in the manuscript, particularly since aspect ratio is only performed on 15 cells per condition and not on experimental replicates. The authors should clarify if sample identity was blinded prior to analysis.
4. The constructs that form the basis of conclusions of ER versus mitochondrial-targeted MFN2 require additional controls to support robust conclusions. The immunofluorescence data suggests that MFN2-ACTA to some degree targets outside of mitochondria (Fig. 2A), and also that MFN2-YIFFT seems to localize to some degree to mitochondria (Fig. 2A). The YIFFT construct may also localize more prevalently to mitochondria in the presence of ACTA (Fig. S2A). Mistargeting would make interpretation of these experiments not possible, as these constructs must exclusively localize to their intended organelle to make strong conclusions. Triple labeling with ER and mitochondrial markers would be helpful, as well as western blots to confirm consistent expression levels and protein stability of each construct. The ACTA MFN2 also appears to promote fusion in a MTCH2 KO. How is this reconciled with the conclusion that ER-targeting of MFN2 is required?
5. The authors conclude that "MFN2-mediated fusion requires LPA synthesis", but what is shown instead is that GPATi is epistatic to fusion caused by overexpression of MFN2. The authors should be careful about drawing strong conclusions from their overexpression studies. While MTCH2 overexpression causes hyperfusion in the presence of GPATi, this does not mean that LPA doesn't promote MTCH2-dependent fusion, merely that GPATi does not block hyperfusion caused by MTCH2 overexpression.
6. The collapse of the mitochondrial network in MTCH2 KO cells treated with cycloheximide + GPATi does not indicate the cells "require newly-synthesized LPA to respond to SIMH". Instead, it suggests that mitochondria in MTCH2 KO cells are sensitized to combined GPATi/cycloheximide treatment. Could this collapse be fusion-independent? If mitochondria are treated with nocodazole to relax the mitochondrial network (as in Smirnova et al, MBoC, 2001 or Yang et al, Nat Comm, 2022), does the mitochondrial network appear hyperfused?
7. The fact that FSG67 kills starved cells in the absence of MTCH2 does not mean LPA is not required for starvation induced fusion as concluded by the authors (p.12, first paragraph).

2. Significance:

Significance (Required)

see above

3. How much time do you estimate the authors will need to complete the suggested revisions:

Estimated time to Complete Revisions (Required)

(Decision Recommendation)

Between 1 and 3 months

4. *Review Commons* values the work of reviewers and encourages them to get credit for their work. Select 'Yes' below to register your reviewing activity at Web of Science Reviewer Recognition Service (formerly Publons); note that the content of your review will not be visible on Web of Science.

Web of Science Reviewer Recognition

Yes

Review #3

1. Evidence, reproducibility and clarity:

Evidence, reproducibility and clarity (Required)

Summary:

In this manuscript, Goldman et al. report the role of mitochondrial carrier homolog 2 (MTCH2), a mammalian atypical transporter, in regulating mitochondrial shape. Although numerous studies have previously suggested that MTCH2 localizes to the outer membrane of mitochondria (OMM) and acts in a myriad of processes including apoptosis, energy production, mitochondrial dynamics, lipid metabolism, and calcium signaling, its primary function remains uncertain. Using extensive fluorescence imaging, the authors reveal a functional relationship between MTCH2 and MFN1/2, large GTPases regulating mitochondrial fusion. Overexpression of MTCH2 restored mitochondrial elongation in MFN2 KO cells, but not MFN1 KO or MFN1/2 DKO cells. Overexpression of MFN2, but not MFN1, recovered mitochondrial elongation in MTCH2 KO cells. These results suggest that MTCH2 and MFN1 cooperatively act in

mitochondrial elongation, and that MFN2 promotes mitochondrial fusion independently of MTCH2 and MFN1. Strikingly, ER-anchored MFN2 can rescue mitochondrial shaping defects in MTCH2 KO cells. The authors also investigated the role of lysophosphatidic acid (LPA), a mitochondrial fusion-promoting lipid, in MTCH2- and MFN2-mediated processes, and found that inhibition of LPA synthesis led to suppression of mitochondrial elongation in MTCH2 KO cells overexpressing MFN2, but not MFN2 KO cells overexpressing MTCH2. Collectively, they propose that MFN1 and MFN2 mediates mitochondrial fusion via two distinct mechanisms: one in the OMM depending on MTCH2 and MFN1, and the other in the ER depending on MFN2 and LPA synthesis.

Major comments:

1. As FSG67, an inhibitor of glycerol-phosphate acyl transferase (GPAT) for LPA synthesis, blocks GPAT1/2 in the OMM and GPAT3/4 in the ER, it remains possible that OMM-anchored MFN2 cooperates with GPAT1/2 for concentrating LPA at the mitochondrial fusion site. Thus, the authors should test if loss of GPAT3/4, but not GPAT1/2, suppresses mitochondrial elongation in MTCH2 KO cells overexpressing MFN2.
2. It seems conceivable that FSG67 treatment causes a decrease in the protein levels of MFN2 and/or MTCH2, thereby leading to mitochondrial fragmentation. The authors should clarify this point by western blotting.

Minor comments:

1. It would be interesting to investigate whether an ER-anchored MFN2 variant defective in GTP hydrolysis can restore mitochondrial elongation in MTCH2 KO cells.
2. The authors should add single-color fluorescent images into Figs. 1A, 1C, 1F, S1A, 2H, S2A, S2C, 3C, 3F, S3F, and S3H.

2. Significance:

Significance (Required)

General assessment:

The findings in this study are potentially interesting and could provide new insights into the molecular mechanisms of mitochondrial fusion in mammals. The fluorescence imaging data are of high quality with quantification and statistical evaluation, mostly supporting the conclusion. There are, however, some missing

points regarding the relationships among MTCH2, MFN1, MFN2, and GPAT1/2/3/4 in more detail. For example, does ER-anchored MFN2 interact with GPAT3/4? Does MTCH2 interact with MFN1 to promote mitochondrial elongation? Is LPA required for MFN1-mediated mitochondrial fusion? Nevertheless, this study would significantly be strengthened if the authors clarify the major and minor comments.

Advance:

This study raises the possibilities that ER-anchored MFN2 may act in transport of LPA from the ER to mitochondria in cooperation with GPAT3/4, and that MTCH2 may promote MFN1-mediated mitochondrial fusion independently of LPA.

Audience:

Given the findings that ER-anchored MFN2 and LPS synthesis cooperatively acts in promoting mitochondrial fusion, it will attract a broad range of researchers who study mitochondria, ER, membrane fusion, lipids, and interorganellar communication.

3. How much time do you estimate the authors will need to complete the suggested revisions:

Estimated time to Complete Revisions (Required)

(Decision Recommendation)

Between 1 and 3 months

4. *Review Commons* values the work of reviewers and encourages them to get credit for their work. Select 'Yes' below to register your reviewing activity at Web of Science Reviewer Recognition Service (formerly Publons); note that the content of your review will not be visible on Web of Science.

Web of Science Reviewer Recognition

Yes

Full Revision

Manuscript number: RC-2022-01705R

Corresponding author(s): Prof. Atan Gross, Dr. Andres Goldman

1. General Statements

In this paper we studied the contribution of MTCH2, MFN2 and lysophosphatidic acid (LPA) synthesis to mitochondrial fusion. We found that MTCH2 overexpression enforces MFN1-dependent mitochondrial fusion, which is independent of MFN2 expression and insensitive to inhibition of LPA synthesis. Inversely, MFN2 overexpression enforces mitochondrial fusion independent of MTCH2 expression but is sensitive to LPA synthesis inhibition. Using MFN2 mutants we demonstrate that MFN2's ability to enforce fusion is connected to its tethering activity, and probably coupled to LPA transfer from the ER to the mitochondria. We also used two cellular stress conditions to explore the effect of loss of either MTCH2 or MFN2 on mitochondrial plasticity in relation to LPA availability. We revealed that loss of MTCH2 in combination with loss of MFN2/or in combination with LPA synthesis inhibition, largely impairs mitochondrial fusion. Our data suggests a model in which two separate and cooperative pathways are required to sustain mitochondrial plasticity and highlight the importance of the contribution of ER-mitochondrial communication to mitochondrial fusion.

We found the reviewers comments insightful and fair, and we addressed the majority of them. Importantly our manuscript lacked a functional mechanism for MTCH2, and most recently an elegant paper published in Science described that MTCH2 functions as an OMM insertase. This recent finding might provide clues to understanding some of the findings reported in our manuscript. Thus, in accordance with the reviewers' recommendations, we performed additional experiments related to the insertase activity and discussed its wider implications.

2. Point-by-point description of the revisions

Reviewer #1

1-1) Authors conclude that "MFN2 and MTCH2 compensate for each other's absence", but the compensation works only when they are overexpressed. On the other hand, upon HBSS or CHX treatment, MFN2 KO or MTCH2 KO cells have elongated/hyperfused mitochondria, but this is not observed in double deficient cells. In this case, MFN2 and MTCH2 show compensatory effects

on mitochondria elongation. The authors believe the two conditions, unstressed & OE and stressed conditions, activating same molecular machineries, but it is not fully supported by the data. For example, in unstressed condition, MTCH2 OE can recover MFN2 KO but not of MFN1 KO, suggesting MTCH2-dependent mitochondria fusion requires MFN1, but it is not tested for stressed condition.

Furthermore, even if the machineries are common among stressed and unstressed conditions, authors should discuss why the endogenous MFN2/MTCH2 expression is not enough to activate mitochondria fusion in MTCH2/MFN2 KO cells, respectively, and how the compensatory effects are activated upon HBSS or CHX treatment.

R: We thank the reviewer for this important comment.
1) The reviewer's comment is correct; in resting conditions the endogenous expression of MFN2 in MTCH2 KO and vice-versa are not sufficient to compensate for each other's absence, since in steady state they both remain largely fragmented. Thus, we corrected the text accordingly by removing this concept. Interestingly, in the revised MS we show that MFN2 KO results in an increase in MTCH2 expression levels in the mitochondria and to a strong decrease in the GPAT3 and 4 expression levels in the ER (Rev Fig. 3B, C). These results suggest that: 1) Expression of MFN2 is important for the stabilization of GPAT3 and 4 (perhaps they form a functional complex together); 2) the expression levels of <u>endogenous</u> MTCH2 are possibly elevated to compensate for the decreased biosynthesis of LPA and increased demand of LPA funneling for mitochondrial fusion. This hypothesis was added to the discussion.

2) In the revised MS, we tested the possibility that MTCH2 overexpression together with mitochondrial fusion stress could enforce mitochondrial fusion in MFN1 KO cells, and found that it could not (Rev Supp Fig. 4 J-K).

3) We have also discussed what might be the compensatory effects of MTCH2 and MFN2 when activated upon enforced mitochondrial fusion induced by protein overexpression and stress (page 20 last paragraph).

1-2) Authors found out that ER-targeted MFN2 can rescue the mitochondria fragmentation in MTCH2 KO MEF cells, but mitochondria-targeted MFN2 has a lower effect than Wt MFN2. (Fig 2A&B). This finding suggests that MTCH2 loss might impair MFN2 localization at the ER. The authors should investigate endogenous MFN2 localization in MTCH2 KO MEFs.

R: We thank the reviewer for this insightful comment. We addressed this point and show that MTCH2 deletion does not change the expression levels or the subcellular localization of endogenous MFN2 (Rev Fig. 3B).

1-3) The analysis to test whether recovery of mitochondrial morphology by ER-targeted MFN2 in MTCH2 KO depends on LPA synthesis or not is missing (Fig 3G). Authors should examine whether mitochondrial elongation induced by ER-localized MFN2 in MTCH2 KO cells is impaired by the GPAT inhibitor.

R: This analysis was performed (we also included the other mutants), and presented the new data in the revised MS (Rev Supp Fig. 3N, O).

1-4) In the discussion section, authors suggest that mitochondrial LPA would be a crucial factor for MFN2 dependent mitochondrial fusion. To test this hypothesis, authors should overexpress mitochondrial GPAT and evaluate its effect on mitochondrial morphology.

R: We thank the reviewer for this important suggestion. We ordered two commercially available plasmids encoding GPAT1 to address this point (DNASU HsCD00082324 and HsCD00082324) but unfortunately the proteins were not expressed in our cells. In addition, we revised the text and changed the angle of the interpretation of these results, and clarified that inhibiting LPA impairs MTCH2-independent mitochondrial plasticity in response to MFN2 overexpression, rather than MFN2-dependent mitochondrial fusion.

1-5) In the discussion section, authors indicated that ER-targeted MFN2 could recover mito-ER contacts leading to LPA flux from ER to mitochondria and mitochondria elongation in MTCH2 KO. However, MTCH2 KO itself already have more mito-ER contacts (Fig 2D-H), and an artificial linker fails to recover mitochondria fragmentation in MTCH2 KO cells (Fig S2C, D). Thus, increased number of contacts appears not sufficient to recover the phenotype. The authors should consider this point in the discussion.

R: We added a comment on this important point in the results section (page 10, line 9)

2) Certain methods are not appropriate to support the stated conclusions.

2-1) Authors assess "mitochondria fusion" by evaluating mitochondrial morphology. The authors also describe mitochondrial clumping as a fusion-impaired phonotype (Fig 4A&B). Mitochondrial fusion should be evaluated using a PEG assay or a mtPA-GFP analysis.

R: We now provide in the revised MS results of a mtPA-GFP analysis done for MTCH2 KO MEFs exposed to GPATs inhibitor and treated with CHX (Rev Fig. 4G, H). This experiment supports the notion that loss of MTCH2 along with LPA synthesis inhibition largely impairs mitochondrial fusion in response to CHX.

2-2) In figure 2D-G, authors show that MTCH2 KO cells have more and longer mitochondria-ER contacts. The correct experiment is not to compare these cells to WT, but to KO reconstituted with MTCH2.

R: The reviewer is correct however it will take us many more months to generate a stable MTCH2 rescue cell line and to perform EM analysis of these cells, which would significantly slow down the revision of our MS.

2-3) Since staining of MitoTracker depends on mitochondrial membrane potential, mitochondria with low potential would be invisible and excluded from the analysis. Authors should investigate mitochondrial morphology by immunostaining also in Fig 4D, S4A, D, and K.

R: We agree with the reviewer's comment, but re-doing all these experiments will be too labor-intensive and time consuming. We therefore focused on Supp Fig. 4A, in which the combination of FSG67 (GPATi in the revised MS) and HBSS treatment impaired mitochondrial membrane potential and mitochondria did not uptake the MitoTracker dye. We repeated this experiment using immunofluorescence, performed new quantifications, and incorporated the new data into the MS (Rev Fig. 4A). We did not remove Supp Fig. 4A since we wanted to emphasize the point that the combination of LPA synthesis inhibition and amino-acid deprivation results in loss of mitochondrial membrane potential.

Minor comments

1) Since authors use FSG67, an inhibitor against GPAT1, 2 and 3, knocking down of each of GPATs will improve the significance of this work.

R: We thank the reviewer for suggesting these experiments. Since the contribution of mitochondrial GPATs to mitochondrial fusion was already established, we complemented our studies by silencing ER GPATs 3 and 4. We tested the contribution of ER-GPATs to MTCH2-independent mitochondrial fusion elicited by MFN2 overexpression (Rev Fig. 3L-N) and induced by either HBSS or CHX (Rev Fig. 4I-K).

2) Recently, a paper about MTCH2 is published (Guna et al., Science), which shows its insertase activity on tail anchored proteins. Authors should include this point of view in the discussion.

R: We performed new experiments to address this important point. We evaluated the effect of MTCH2 deletion on the expression and localization of the fusion and fission proteins and on the LPA synthesis proteins, and found minor changes (Rev Supp Fig. 1D and Rev Fig. 3B, C). Thus, the effects we are seeing in the MTCH2 KO cells do not seem to be directly related to its insertase activity.

3-1) There are some discrepancies with the previous Labbé et al article.: Labbé et al. suggest that MTCH2 activity on mitochondria (from HCT116 cells) fusion is dependent on LPA, based on in vitro fusion assay. On the other hand, this manuscript shows that inhibition of LPA synthesis could not block MTCH2-induced mitochondria elongation in WT MEF and HEK293T cells. MTCH2 KO HCT116 cells are resistant to HBSS- but sensitive to CHX-induced mitochondria elongation. In this manuscript, MTCH2 KO MEF cells are sensitive to both stimuli, and only when MTCH2- and MFN2-deficient MEF cells are resistant to both stimuli. These discrepancies would be caused by difference of assay system or cell lines, but it is not clearly addressed.

R: We thank the reviewer for raising these discrepancies. Despite the discrepancies in the response of MTCH2 KO MEFs to HBSS, both manuscripts largely support the model that MTCH2 funnels LPA towards mitochondrial fusion sites. Our data suggests that loss of MTCH2 unmasks the requirement of sufficient LPA synthesis to sustain mitochondrial fusion, and we also show that MTCH2 overexpression can enforce mitochondrial fusion in

the presence of GPATs inhibitor. These two results can be conciliated by the model that MTCH2 is able to optimize or funnel the levels of LPA towards the mitochondrial fusion sites, and when MTCH2 is absent and not able to catalyze this process, mitochondria rely on LPA levels to stay elevated to enable mitochondrial fusion but in a less efficient way. Interestingly, since we show that mitochondrial fusion enforced by HBSS shows full dependency on LPA synthesis, it is expected that loss of MTCH2 will have a stronger impact on HBSS-mediated mitochondrial fusion than on CHX. Nevertheless, MEFs clearly and consistently are sensitive to HBSS, yet this does not exclude that MTCH2 deletion in combination with HBSS treatment may be synergistically detrimental for the cell in other aspects of its well-functioning.

Reviewer #2

Specific points

1. The authors should comment on recently published work in Science that MTCH1 and likely MTCH2 are outer membrane insertases. The data in the manuscript seem consistent with the model that an undetermined protein may be poorly inserted in a MTCH2 knockout leading to reduced fusion activity mediated by MFN1. Could this explain how MTCH2 overexpression selectively restores fusion to MFN2 KO cells?

R: We thank the reviewer for raising this important point, and we have now performed new experiments to address it. We evaluated the effect of MTCH2 deletion on the expression and localization of the fusion and fission proteins and on the LPA synthesis proteins, and found minor changes (Rev Supp Fig. 1D and Rev Fig. 3B, C). Thus, the effects we are seeing in the MTCH2 KO cells do not seem to be directly related to its insertase activity.

2. The authors make the claim that "MFN2 and MTCH2 compensate for each other's absence" though this is not supported by their data. For example, MFN2 expression is not affected in an MTCH2 KO but cannot compensate to promote fusion. Rather, the authors find that MTCH2 and MFN2 are capable of promoting fusion when overexpressed in the absence of the other.

R: We thank the reviewer for this important comment.
The reviewer's comment is correct, and in resting conditions the endogenous expression of MFN2 in MTCH2 KO cells and vice-versa are not sufficient to compensate for each other's absence, since in steady state they both remain largely fragmented. Thus, we corrected the text accordingly by removing this concept. Interestingly, in the revised MS we show that MFN2 KO results in an increase in MTCH2 expression levels in the mitochondria and to a strong decrease in the GPAT3 and 4 expression levels in the ER (Rev Fig. 3B, C). These results suggest that: 1) Expression of MFN2 is important for the stabilization of GPAT3 and 4 (perhaps they form a functional complex together); 2) the expression levels of <u>endogenous</u> MTCH2 are possibly elevated to compensate for the decreased biosynthesis of LPA and increased demand of LPA funneling for mitochondrial fusion. This hypothesis was added to the discussion.

3. The authors rely heavily on claims that tagged MFN1 and MFN2 are fully functional, but is it possible that MFN1-GFP is only partially functional? Does untagged MFN1 overexpression cause mitochondrial fusion in a MTCH2 KO? The quantification of this is done only with aspect ratio, and not by categorization of mitochondrial morphology (Fig. S1). The authors should present both analyses in this and all other experiments in the manuscript, particularly since aspect ratio is only performed on 15 cells per condition and not on experimental replicates. The authors should clarify if sample identity was blinded prior to analysis.

R: As suggested by the reviewer, we repeated all the MFN1 overexpression experiments using an untagged version of the protein, which was overexpressed in MFN1 KO, MTCH2 KO and MFN2 KO MEFs. The new data was included in Rev Fig. 1C-E. The data is consistent with our previous observations using MFN1-GFP. Also, mitochondrial morphology classification was added to the majority of the experiments presented in our MS, which represents quantification of three separate technical repetitions of the experiments (this analysis was not performed blinded).

4. The constructs that form the basis of conclusions of ER versus mitochondrial-targeted MFN2 require additional controls to support robust conclusions. The immunofluorescence data suggests that MFN2-ACTA to some degree targets outside of mitochondria (Fig. 2A), and also that MFN2-YIFFT seems to localize to some degree to mitochondria (Fig. 2A). The YIFFT construct may also localize more prevalently to mitochondria in the presence of ACTA (Fig. S2A). Mistargeting would make interpretation of these experiments not possible, as these constructs must exclusively localize to their intended organelle to make strong conclusions. Triple labeling with ER and mitochondrial markers would be helpful, as well as western blots to confirm consistent expression levels and protein stability of each construct. The ACTA MFN2 also appears to promote fusion in a MTCH2 KO. How is this reconciled with the conclusion that ER-targeting of MFN2 is required?

R: We thank the reviewer for these important concerns. We repeated all the MFN2 mutant experiments, including the GTPase mutant MFN2 K109A, and included ER labelling to the MFN2-IYFFT and MFN2-ACTA transfections (Rev Fig. 2B; Supp Fig. 2A, C, D). We also included line fluorescence plot analysis to show localization of MFN2 mutants along with ER or mitochondrial markers (Rev Fig. 2C). Because these studies are cell-based analysis, which were performed using transient transfection, and the efficiency of expression of some of these constructs was very low, it would be technically very difficult to detect their expression using subcellular fractionation. Nevertheless, the constructs MFN2-IYFFT-FLAG and MFN2-ACTA used in our MS were previously reported and their subcellular localization was confirmed (Sugiura, A. *et al.* (2013) 'MITOL Regulates Endoplasmic Reticulum-Mitochondria Contacts via Mitofusin2', *Molecular Cell*, 51(1), pp. 20–34. doi:https://doi.org/10.1016/j.molcel.2013.04.023).

5. The authors conclude that "MFN2-mediated fusion requires LPA synthesis", but what is shown instead is that GPATi is epistatic to fusion caused by overexpression of MFN2. The authors should

be careful about drawing strong conclusions from their overexpression studies. While MTCH2 overexpression causes hyperfusion in the presence of GPATi, this does not mean that LPA doesn't promote MTCH2-dependent fusion, merely that GPATi does not block hyperfusion caused by MTCH2 overexpression.

R: We thank the reviewer for pointing out these issues. We have revised the text accordingly, and instead of "MFN2-mediated fusion requires LPA synthesis" we wrote "GPATi is epistatic to fusion caused by overexpression of MFN2" (page 13, line 14). We also added "reducing LPA levels by inhibiting GPAT activity is insufficient to impair mitochondrial fusion enforced by MTCH2-overxpression" (page 13, line 9).

6. The collapse of the mitochondrial network in MTCH2 KO cells treated with cycloheximide + GPATi does not indicate the cells "require newly-synthesized LPA to respond to SIMH". Instead, it suggests that mitochondria in MTCH2 KO cells are sensitized to combined GPATi/cycloheximide treatment. Could this collapse be fusion-independent? If mitochondria are treated with nocodazole to relax the mitochondrial network (as in Smirnova et al, MBoC, 2001 or Yang et al, Nat Comm, 2022), does the mitochondrial network appear hyperfused?

R: Thank you and as the reviewer suggested we rephrased to "mitochondria in MTCH2 KO cells are sensitized to combined GPATi/CHX treatment." (page 16, line 4). In addition, we performed the nocodazol experiment suggested by the reviewer and included the results in the MS (Rev Supp Fig. 4G-I). The results suggest that MTCH2 KO/GPATi generate clumping and collapse of the mitochondrial network that can be relaxed by blocking microtubule polymerization. Importantly eventhough the network is still highly fragmented, mitochondrial morphology has changed from large rounded and fragmented to short and tubulated mitochondria, suggesting that MTCH2 KO/GPATi does not impair other mitochondrial architectural changes induced by CHX.

7. The fact that FSG67 kills starved cells in the absence of MTCH2 does not mean LPA is not required for starvation induced fusion as concluded by the authors (p.12, first paragraph).

R: Thank you and we corrected this part of the MS (page 14, line 19).

Reviewer #3

Major comments:

1. As FSG67, an inhibitor of glycerol-phosphate acyl transferase (GPAT) for LPA synthesis, blocks GPAT1/2 in the OMM and GPAT3/4 in the ER, it remains possible that OMM-anchored MFN2 cooperates with GPAT1/2 for concentrating LPA at the mitochondrial fusion site. Thus,

the authors should test if loss of GPAT3/4, but not GPAT1/2, suppresses mitochondrial elongation in MTCH2 KO cells overexpressing MFN2.

R: We appreciate the reviewer's comment and value this observation. We now included in the revised MS two different sets of experiments: silencing ER resident GPATs in MTCH2 KO MEFs, and enforcing mitochondrial fusion either by MFN2 overexpression (Rev Fig. 3L-N) or by HBSS/CHX stress (Rev Fig. 4I-K). We found that ER GPATs do have a contribution to MTCH2-independent mitochondrial fusion enforced by MFN2 overexpression, and to HBSS-induced mitochondrial fusion. These new results suggest that in the absence of MTCH2, LPA synthetized at the ER, is utilized for mitochondrial fusion. Unfortunately, we were unsuccessful in silencing GPAT1 (we tried two different sets of siRNAs, each composed of four different oligos).

2. It seems conceivable that FSG67 treatment causes a decrease in the protein levels of MFN2 and/or MTCH2, thereby leading to mitochondrial fragmentation. The authors should clarify this point by western blotting.

R: We thank the reviewer for this insightful comment. We performed these experiments and the results appear in the MS (Rev Supp Fig. 3A). We analyzed by Western blot the effect of FSG67 (named GPATi in the revised MS) on the expression levels of MTCH2, MFN2, fusion/fission proteins, GPATs, and a few other proteins. Interestingly, GPATi actually resulted in a small increase in MFN2 expression levels but had no effect on MTCH2 expression levels. Moreover, thanks to the reviewer's suggestion, we also revealed that in non-treated and GPATi-treated MFN2 KO cells, MTCH2 expression levels were increased and GPATs3/4 expression levels were largely decreased (Rev Fig. 3B, C). These results suggest that: 1) Expression of MFN2 is important for the stabilization of GPAT3 and 4 (perhaps they form a functional complex together); 2) the expression levels of <u>endogenous</u> MTCH2 are possibly elevated to compensate for the decreased biosynthesis of LPA and increased demand of LPA funneling for mitochondrial fusion. This hypothesis was added to the discussion.

Minor comments:

1. It would be interesting to investigate whether an ER-anchored MFN2 variant defective in GTP hydrolysis can restore mitochondrial elongation in MTCH2 KO cells.

R: We thank the reviewer for this suggestion. We generated a MFN2-IYYFT K109A mutant, but unfortunately it was expressed at very low levels, and in the expressed cells it elicited mitochondrial aggregation (see below).

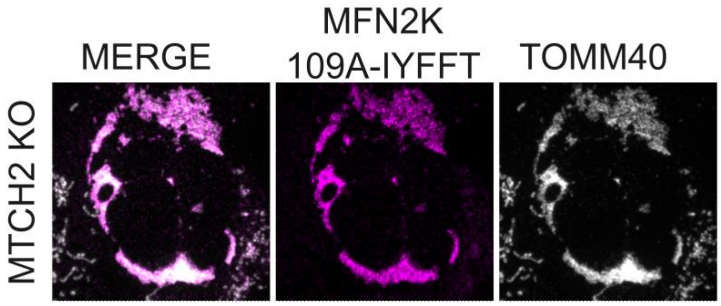

2. The authors should add single-color fluorescent images into Figs. 1A, 1C, 1F, S1A, 2H, S2A, S2C, 3C, 3F, S3F, and S3H.

R: We thank the reviewer and added single-color fluorescent images into all the Figures requested.

Referee #1:

The authors have addressed our concerns. The paper is now a strong candidate for publication

Referee #2:

In their revised manuscript, Goldman et al perform several key control assays that improve the overall rigor of the work. While the manuscript contains a wealth of data, including an evaluation of steady-state mitochondrial morphology, morphology after stress induced fusion, and the effect of GPAT inhibition of each, a flaw of the manuscript is that the data are often confounding and the narrative remains hard to follow. The authors also performed additional experiments in the revision that muddle their original conclusions. The findings will be of interest to experts who study mitochondrial fusion and certainly warrant publication, but a limitation of the work is that they do not lend themselves to clear conclusions of the role of MTCH2 in mitochondrial fusion and lack accessibility to a broader audience.

The authors now provide data that MTCH2 expression is increased in MFN2 KO cells. If this is the case, why is the abundant MTCH2 insufficient to promote MFN1-dependent mitochondrial fusion without further overexpressing the protein? The manuscript now also includes data that expression of a fusion-defective MFN2 can rescue mitochondrial morphology defects of MTCH2. This implies that MTCH2 is not required for MFN1-dependent fusion per se, but that MFN1 is incapable of supporting normal morphology without MTCH2 and some activity of MFN2. Could this be explained by differences in the relative rates of fission in the cell lines or other indirect effects? Most assays in the manuscript are performed by examining steady-state morphology, but perhaps stronger conclusions could be made with assays of fusion using photoactivatable GFP (as done in CHX treatments in new Fig. 4G). Finally, the different responses to MTCH2 KO with and without GPATi during induced-mitochondrial hyperfusion are interesting, though difficult to interpret. These results are presented in masse without clear conclusions and it is not clear how these data fit into their model.

A key limitation of the work is that there remains a strong possibility that MTCH2 indirectly promotes fusion by reduced targeting of a subset of proteins to the mitochondrial outer membrane, perhaps even ones without established roles in maintaining mitochondrial morphology. The authors addressed this point in the revision by western blotting a few key proteins. A more rigorous test of their model could be to determine if MTCH1 knockdown similarly affects mitochondrial morphology, as both proteins are suggested to influence protein targeting to the mitochondrial outer membrane in the work by Guna et al. If the role of MTCH2 is specific, knockdown of MTCH1 would be predicted to have no effect on mitochondrial

morphology. Barring this experiment, the authors should more explicitly acknowledge this caveat of their work in the discussion.

Referee #3:

In this revised manuscript, Goldman et al. provided additional data and descriptions to address most of the points raised by the referees. In particular, overexpression of MFN2(K109A), a GTPase-inactive variant, can partially rescue mitochondrial elongation in MTCH2 KO cells (Fig. 2B-E), suggesting that MFN2 could function in mitochondrial shaping independently of its GTPase activity and MTCH2. Interestingly, loss of MFN2 leads to a strong increase in the MTCH2 levels and a decrease in the GPAT3/4 levels (Fig. 3B and C). Moreover, MTCH2 KO cells overexpressing MFN2 requires GPAT3/4 to harbor elongated mitochondria (Fig. 3L-N). These findings further support the idea that ER-localized MFN2 acts in GPAT3/4-dependent lysophosphatidic acid synthesis, thereby contributing to mitochondrial elongation in a GTP-independent manner. There are, however, still several concerns that need to be clarified to substantiate their conclusions.

Specific points:
1. It would be necessary to unveil the relationship between MFN2 and GPAT3/4 in more detail. To this end, the authors should examine the protein levels of GPAT3/4 in MFN2 KO cells overexpressing MFN2-WT, MFN2-K109A, MFN2-YIFFT, MFN2-ACTA, MFN2-YIFFT/ACTA, and negative control as shown in Supp Fig. 2A.

2. Based on the recent report that MTCH2 is a membrane protein insertase (Guna et al., Science 378: 317-322 [2022]), it seems conceivable that unknown MTCH2 substrate(s) may act in MFN2-independent mitochondrial elongation. It also remains possible that MTCH2 is a bifunctional (or even multifunctional) protein promoting fusion of mitochondria independently of its insertase activity. Accordingly, as shown in Fig. 1A-C, the authors should try to investigate mitochondrial morphology in MFN2 KO cells overexpressing MTCH2-D189R (insertase-inactive) or MTCH2-K25E (insertase-hyperactive) variants.

3. Loss of MFN2 leads to a strong increase in the MTCH2 levels as well as mitochondrial fragmentation (Fig. 3B and C), which is somewhat inconsistent with the observation that overexpression of MTCH2 recover elongated mitochondria in MFN2 KO cells (Fig. 1A and B). Thus, the authors should explain about this issue a bit in depth.

==========================

Rev_Com_number: RC-2022-01705
New_manu_number: EMBOJ-2023-114368
Corr_author: Gross
Title: MTCH2 cooperates with MFN2 and lysophosphatidic acid synthesis to sustain mitochondrial fusion

1st Editorial Decision 2nd Jun 2023

Dear Atan,

Thank you for the transfer of your research manuscript to EMBO reports. It has been reviewed at our sister journal The EMBO Journal, who could not offer publication and we have invited you to revise your study for potential publication in EMBO reports.

As discussed, please address all suggestions from referee #2 in the text.
Please test whether the insertase activity of MTCH2 is required by knockdown of MTCH1 (ref 2) and by testing insertase-inactive MTCH2 (ref 3).
Please experimentally address all other concerns from referee 3.

The revised study will be re-reviewed by referee 3 and acceptance of the manuscript will depend on a positive outcome of this second round of review.
Please also address all referee concerns in a complete point-by-point response.

We realize that it is difficult to revise to a specific deadline. In the interest of protecting the conceptual advance provided by the work, we recommend a revision within 3 months (September 2nd). Please discuss the revision progress ahead of this time with the editor if you require more time to complete the revisions.

I am also happy to discuss the revision further via e-mail or a video call, if you wish.

You can either publish the study as a short report or as a full article. For short reports, the revised manuscript should not exceed 27,000 characters (including spaces but excluding materials & methods and references) and 5 main plus 5 expanded view figures. The results and discussion sections must further be combined, which will help to shorten the manuscript text by eliminating some redundancy that is inevitable when discussing the same experiments twice. For a normal article there are no length limitations, but it should have more than 5 main figures and the results and discussion sections must be separate. In both cases, the entire materials and methods must be included in the main manuscript file.

*****IMPORTANT NOTE:
We perform an initial quality control of all revised manuscripts before re-review. Your manuscript will FAIL this control and the handling will be DELAYED if the following APPLIES:

1) A data availability section providing access to data deposited in public databases is missing. If you have not deposited any data, please add a sentence to the data availability section that explains that.

2) Your manuscript contains statistics and error bars based on n=2. Please use scatter blots in these cases. No statistics should be calculated if n=2.

When submitting your revised manuscript, please carefully review the instructions that follow below. Failure to include requested items will delay the evaluation of your revision.*****

When submitting your revised manuscript, we will require:

1) a .docx formatted version of the manuscript text (including legends for main figures, EV figures and tables). Please make sure that the changes are highlighted to be clearly visible.

2) individual production quality figure files as .eps, .tif, .jpg (one file per figure).
Please download our Figure Preparation Guidelines (figure preparation pdf) from our Author Guidelines pages https://www.embopress.org/page/journal/14693178/authorguide for more info on how to prepare your figures.

3) a .docx formatted letter INCLUDING the reviewers' reports and your detailed point-by-point responses to their comments. As part of the EMBO Press transparent editorial process, the point-by-point response is part of the Review Process File (RPF), which will be published alongside your paper.

4) a complete author checklist, which you can download from our author guidelines (). Please insert information in the checklist that is also reflected in the manuscript. The completed author checklist will also be part of the RPF.

5) Please note that all corresponding authors are required to supply an ORCID ID for their name upon submission of a revised manuscript (). Please find instructions on how to link your ORCID ID to your account in our manuscript tracking system in our Author guidelines
()

6) We replaced Supplementary Information with Expanded View (EV) Figures and Tables that are collapsible/expandable online. A maximum of 5 EV Figures can be typeset. EV Figures should be cited as 'Figure EV1, Figure EV2" etc... in the text and their respective legends should be included in the main text after the legends of regular figures.

- For the figures that you do NOT wish to display as Expanded View figures, they should be bundled together with their legends in a single PDF file called *Appendix*, which should start with a short Table of Content. Appendix figures should be referred to in the main text as: "Appendix Figure S1, Appendix Figure S2" etc. See detailed instructions regarding expanded view here:

- Additional Tables/Datasets should be labeled and referred to as Table EV1, Dataset EV1, etc. Legends have to be provided in a separate tab in case of .xls files. Alternatively, the legend can be supplied as a separate text file (README) and zipped together with the Table/Dataset file.

7) Please note that a Data Availability section at the end of Materials and Methods is now mandatory. In case you have no data that requires deposition in a public database, please state so instead of refereeing to the database.
See also < https://www.embopress.org/page/journal/14693178/authorguide#dataavailability>). Please note that the Data Availability Section is restricted to new primary data that are part of this study.

8) At EMBO Press we ask authors to provide source data for the main figures. Our source data coordinator will contact you to discuss which figure panels we would need source data for and will also provide you with helpful tips on how to upload and organize the files.

Additional information on source data and instruction on how to label the files are available .

9) The journal requires a statement specifying whether or not authors have competing interests (defined as all potential or actual interests that could be perceived to influence the presentation or interpretation of an article). In case of competing interests, this must be specified in your disclosure statement. Further information: https://www.embopress.org/competing-interests

10) Figure legends and data quantification:
The following points must be specified in each figure legend:

- the name of the statistical test used to generate error bars and P values,
- the number (n) of independent experiments (please specify technical or biological replicates) underlying each data point,
- the nature of the bars and error bars (s.d., s.e.m.)

- If the data are obtained from n {less than or equal to} 5, show the individual data points in addition to the SD or SEM.
- If the data are obtained from n {less than or equal to} 2, use scatter blots showing the individual data points.

Discussion of statistical methodology can be reported in the materials and methods section, but figure legends should contain a basic description of n, P and the test applied.

See also the guidelines for figure legend preparation:
https://www.embopress.org/page/journal/14693178/authorguide#figureformat

- Please also include scale bars in all microscopy images.

11) Our journal encourages inclusion of *data citations in the reference list* to directly cite datasets that were re-used and obtained from public databases. Data citations in the article text are distinct from normal bibliographical citations and should directly link to the database records from which the data can be accessed. In the main text, data citations are formatted as follows: "Data ref: Smith et al, 2001" or "Data ref: NCBI Sequence Read Archive PRJNA342805, 2017". In the Reference list, data citations must be labeled with "[DATASET]". A data reference must provide the database name, accession number/identifiers and a resolvable link to the landing page from which the data can be accessed at the end of the reference. Further instructions are available at .

12) As part of the EMBO publication's Transparent Editorial Process, EMBO reports publishes online a Review Process File to accompany accepted manuscripts. This File will be published in conjunction with your paper and will include the referee reports, your point-by-point response and all pertinent correspondence relating to the manuscript.

You are able to opt out of this by letting the editorial office know (emboreports@embo.org). If you do opt out, the Review Process File link will point to the following statement: "No Review Process File is available with this article, as the authors have chosen not to make the review process public in this case."

We would also welcome the submission of cover suggestions, or motifs to be used by our Graphics Illustrator in designing a cover.

I look forward to seeing a revised form of your manuscript when it is ready and please let me know if you have questions or comments regarding the revision.

Kind regards,

Martina

Martina Rembold, PhD
Senior Editor
EMBO reports

Dear Martina,

Please find below our point-by-point response to issues raised by the two Reviewers.

Reviewer #2:

The authors now provide data that MTCH2 expression is increased in MFN2 KO cells. If this is the case, why is the abundant MTCH2 insufficient to promote MFN1-dependent mitochondrial fusion without further overexpressing the protein?

R: This point was further addressed at the end of the Results and Discussion sections (page 17 starting at line 20, and on page 18 line 3)

A key limitation of the work is that there remains a strong possibility that MTCH2 indirectly promotes fusion by reduced targeting of a subset of proteins to the mitochondrial outer membrane, perhaps even ones without established roles in maintaining mitochondrial morphology. If the role of MTCH2 is specific, knockdown of MTCH1 would be predicted to have no effect on mitochondrial morphology. Barring this experiment, the authors should more explicitly acknowledge this caveat of their work in the discussion.

R: We addressed the point raised by the reviewer. In the revised manuscript we included silencing of MTCH1, and found that it had no effect on mitochondria morphology (EV 1F-I). We also assessed if MTCH2's insertase activity is required for mitochondrial fusion. We overexpressed a MTCH2-GFP hyperactive mutant (K25E) and a MTCH2-GFP inactive mutant (D189R) in MTCH2 KO cells and found that both mutants could rescue mitochondrial elongation as efficient as WT MTCH2 (EV 1J-L). However, it should be noted that MTCH2 KO cells transfected with the MTCH2-GFP inactive mutant (D189R) seemed to demonstrate more cells with fragmented mitochondria as compared to MTCH2 KO cells transfected with WT MTCH2-GFP (EV 1K). Altogether, these results suggest that MTCH2 may require its insertase activity in a partial manner to regulate mitochondrial dynamics.

Reviewer #3:

Specific points:
1. It would be necessary to unveil the relationship between MFN2 and GPAT3/4 in more detail. To this end, the authors should examine the protein levels of GPAT3/4 in MFN2 KO cells overexpressing MFN2-WT, MFN2-K109A, MFN2-YIFFT, MFN2-ACTA, MFN2-YIFFT/ACTA, and negative control as shown in Supp Fig. 2A.

R: We understand the importance of this point. We have tried to execute the experiments requested by the reviewer but unfortunately since MEFs are difficult to transfect we managed to obtain only very low levels of expression of the MFN2 mutants, which were insufficient to detect any effect. Nevertheless, we thought it was of critical importance to

demonstrate that the changes in ER GPATs and MTCH2 levels were a direct consequence of MFN2 deletion. Thus, we generated a stable polyclonal cell line of MFN2 KO MEFs expressing FLAG tagged WT MFN2, and the results show that GPATs3/4 and MTCH2 levels were restored (EV 3A).

2. Based on the recent report that MTCH2 is a membrane protein insertase (Guna et al., Science 378: 317-322 [2022]), it seems conceivable that unknown MTCH2 substrate(s) may act in MFN2-independent mitochondrial elongation. It also remains possible that MTCH2 is a bifunctional (or even multifunctional) protein promoting fusion of mitochondria independently of its insertase activity. Accordingly, as shown in Fig. 1A-C, the authors should try to investigate mitochondrial morphology in MFN2 KO cells overexpressing MTCH2-D189R (insertase-inactive) or MTCH2-K25E (insertase-hyperactive) variants.

R: We agree with the reviewer's comments. Unfortunately, we had difficulties expressing the MTCH2 mutants which were obtained directly from the authors of the Science paper. After several attempts we decided to generate our own mutants on the background of the MTCH2 plasmid used in this study. We managed to generate both insertase inactive (D189R) and insertase hyperactive (K25E) mutants, but because of time limitations we focused on their ability to rescue MTCH2 KO dependent mitochondrial fragmentation. We found that both mutants could rescue mitochondrial fragmentation in the MTCH2 KO as efficient as WT MTCH2 (EV 1J-L). However, it should be noted that MTCH2 KO cells transfected with the MTCH2-GFP inactive mutant (D189R) seemed to demonstrate more cells with fragmented mitochondria as compared to MTCH2 KO cells transfected with WT MTCH2-GFP (EV 1K). Altogether, these results suggest that MTCH2 may require its insertase activity in a partial manner to regulate mitochondrial dynamics.

3. Loss of MFN2 leads to a strong increase in the MTCH2 levels as well as mitochondrial fragmentation (Fig. 3B and C), which is somewhat inconsistent with the observation that overexpression of MTCH2 recover elongated mitochondria in MFN2 KO cells (Fig. 1A and B). Thus, the authors should explain about this issue a bit in depth.

R: We appreciate the reviewer comment, and we addressed this point in more depth at the end of the Results and Discussion sections (page 17 starting at line 20, and on page 18 line 3)

1st Revision - Editorial Decision 24th Oct 2023

Dear Atan,

Thank you for the submission of your revised manuscript to EMBO Reports. It has been re-reviewed by former referee #2 (report copied below) who concluded that you have adequately addressed all remaining referee concerns and supports publication.

Browsing through the manuscript myself, I noticed a few editorial things that we need before we can proceed with the official acceptance of your study.

- With four figures your manuscript will be published as Scientific Report. To comply with the Reports format, please combine the Results and Discussion section and please keep an eye on our character limit (27,000 characters including spaces but excluding materials & methods and references). It seems you have already combined these sections but the header "Results and Discussion" is missing.

- Please provide up to 5 keywords.

- Please update the 'Conflict of interest' paragraph to our new 'Disclosure and competing interests statement'. For more information see
https://www.embopress.org/page/journal/14693178/authorguide#conflictsofinterest

- Please remove the Author Contributions from the manuscript file and make sure that the author contributions in our online submission system are correct and up-to-date. The information you specified in the system will be automatically retrieved and typeset into the article. You can enter additional information in the free text box provided, if you wish.

- Reference format: please remove the DOIs from the reference list (except for the preprints). Et al needs to be used after 10 author names.
- Bartoš, L., (2023) and Li, D et al (2023) are preprints. Please cite them in the text as: (preprint: NAME1 et al, YEAR); in the reference list as: Author NAME1, Author NAME2 (YEAR) article title. bioRxiv doi [PREPRINT].

- Author Checklist: Please add the manuscript number in the header.

- Please report in the methods section whether the cell lines were recently authenticated (e.g., by STR profiling) and tested for mycoplasma contamination (as indicated in the Author Checklist, Cell materials).

- Please provide information on funding in the manuscript (Acknowledgment section). The information provided in the online submission system must match the information in the manuscript.

- Please add callouts for the EV figures in the text.

- The manuscript sections are in the wrong order. (The Figure legends need to go after References; first the main figure legends and then EV figure legends).
The correct order is like this:
Title page - Abstract - Introduction - Results and Discussion - Materials and Methods - Acknowledgements - Disclosure and competing interests statement - References - Figure legends - Tables and their legends (not EV tables) - Expanded View Figure legends

- Supplementary Figures should be renamed Expanded View Figures. Supplementary Figure 1 is Expanded View Figure 1 etc. In the text the callout is Fig EV1.

- Our production/data editors have asked you to clarify several points in the figure legends (see below). Please incorporate these changes in the manuscript and return the revised file with tracked changes with your final manuscript submission:
1. Please note that the figure legend style does not comply with the journal guidelines i.e. all the figure legends are in a run-on style. All figure panel descriptions must be separated by a line break.
2. Please provide titles for all Expanded View figures (referred as supplementary figures in the manuscript).
3. Please define the annotated p values ****/***/**/* in the legend of figure 1c, f, i; 2e, i; 3c, e, h, k, n; 4c, f, k, n; EV1c, i, l; EV2b, e; EV3f, h, k, n, p; EV4c, f, i, k, p as appropriate.
4. Please note that information related to n is missing in the legend of figure 1i.

- The images shown in Figure 1D MFN2 KO / Control and Figure EV2A MFN2 KO / CONTROL appear very similar but in Fig. 1D it states that it has been stained for TOMM40 and Fig. EV2A says TOMM20. Please double check these panels. Also, if the duplication is intended, this needs to be clearly stated in both figure legends.

- The source data folders for Figure 4 need to be grouped into one folder and uploaded as one folder for Figure 4. We realize

that the existing sub-folders are already large so please let us know in case the combined folder would exceed the size and would not allow you to upload it. The source data for the EV figures need to be grouped and uploaded as one folder.

- As a standard procedure, we edit the title and abstract of manuscripts to make them more accessible to a general readership. Please find the edited version below my signature and incorporate it in the manuscript file if you agree with it.

- On a different note, I would like to alert you that EMBO Press offers a new format for a video-synopsis of work published with us, which essentially is a short, author-generated film explaining the core findings in hand drawings, and, as we believe, can be very useful to increase visibility of the work. This has proven to offer a nice opportunity for exposure i.p. for the first author(s) of the study. Please see the following link for representative examples and their integration into the article web page:

https://www.embopress.org/video_synopses
https://www.embopress.org/doi/full/10.15252/embj.2019103932

Please let me know, should you be interested to engage in commissioning a similar video synopsis for your work. According operation instructions are available and intuitive.

We look forward to seeing a final version of your manuscript as soon as possible.

With kind regards,

Martina

Martina Rembold, PhD
Senior Editor
EMBO reports

****

Referee #1:

In the revised manuscript by Goldman et al, the authors have now addressed a primary concern of the reviewers that MTCH2 insertase activity may confound the interpretation of their results, and have made appropriate adjustments to the manuscript to tone down their conclusions. I recommend publication of the manuscript.

****

Abstract
Fusion of the outer mitochondrial membrane (OMM) is regulated by mitofusin 1 (MFN1) and 2 (MFN2), yet the differential contribution of each of these proteins is less understood. Mitochondrial carrier homolog 2 (MTCH2) also plays a role in mitochondrial fusion, but its exact function remains unresolved. MTCH2 overexpression enforces MFN2-indepdendent mitochondrial fusion, proposedly by modulating the phospholipid lysophosphatidic acid (LPA), which is synthetized OMM by glycerol-phosphate acyl transferases (GPATs) in the endoplasmic reticulum (ER) and the OMM. Here we report that MTCH2 requires MFN1 to enforce mitochondrial fusion and that fragmentation caused by loss of MTCH2 can be specifically counterbalanced by overexpression MFN2 but not MFN1, partially independent of its GTPase activity and mitochondrial localization. Pharmacological inhibition of GPATs (GPATi) or silencing ER-resident GPATs suppresses MFN2's ability to compensate for the loss of MTCH2. Loss of either MTCH2, MFN2 or GPATi does not impair stress-induced mitochondrial fusion, whereas the combined loss of MTCH2 and GPATi or the combined loss of MTCH2 and MFN2 [CORRECT?] does. Taken together, we unmask two cooperative mechanisms that sustain mitochondrial fusion.

Referee #1:

In the revised manuscript by Goldman et al, the authors have now addressed a primary concern of the reviewers that MTCH2 insertase activity may confound the interpretation of their results, and have made appropriate adjustments to the manuscript to tone down their conclusions. I recommend publication of the manuscript.

***
Rev_Com_number: RC-2022-01705
New_manu_number: EMBOR-2023-57575V2
Corr_author: Gross
Title: MTCH2 cooperates with MFN2 and lysophosphatidic acid synthesis to sustain mitochondrial fusion

All editorial and formatting issues were resolved by the authors.

2nd Revision - Editorial Decision 7th Nov 2023

Prof. Atan Gross
Weizmann Inst. of Science
Biological Regulation Department
The Weizmann Institute of Science
Candiotty Bldg, Rm 306a
Rehovot, IL-Rehovot 76100
Israel

Dear Atan,

I am very pleased to accept your manuscript for publication in the next available issue of EMBO reports. Thank you for your contribution to our journal.

Your manuscript will be processed for publication by EMBO Press. It will be copy edited and you will receive page proofs prior to publication. Please note that you will be contacted by Springer Nature Author Services to complete licensing and payment information.

You may qualify for financial assistance for your publication charges - either via a Springer Nature fully open access agreement or an EMBO initiative. Check your eligibility: https://www.embopress.org/page/journal/14693178/authorguide#chargesguide

Should you be planning a Press Release on your article, please get in contact with embo_production@springernature.com as early as possible in order to coordinate publication and release dates.

If you have any questions, please do not hesitate to contact the Editorial Office. Thank you for your contribution to EMBO Reports.

Kind regards,

Martina

Martina Rembold, PhD
Senior Editor
EMBO reports

>>> Please note that it is EMBO Reports policy for the transcript of the editorial process (containing referee reports and your response letter) to be published as an online supplement to each paper. If you do NOT want this, you will need to inform the Editorial Office via email immediately. More information is available here: https://www.embopress.org/transparent-process#Review_Process

EMBO Press Author Checklist

Corresponding Author Name: Atan Gross
Journal Submitted to: EMBO reports
Manuscript Number: EMBOR-2023-57575V2

USEFUL LINKS FOR COMPLETING THIS FORM
The EMBO Journal - Author Guidelines
EMBO Reports - Author Guidelines
Molecular Systems Biology - Author Guidelines
EMBO Molecular Medicine - Author Guidelines

Reporting Checklist for Life Science Articles (updated January
This checklist is adapted from Materials Design Analysis Reporting (MDAR) Checklist for Authors. MDAR establishes a minimum set of requirements in transparent reporting in the life sciences (see Statement of Task: 10.31222/osf.io/9sm4x). Please follow the journal's guidelines in preparing your
Please note that a copy of this checklist will be published alongside your article.

Abridged guidelines for figures
1. Data
The data shown in figures should satisfy the following conditions:
- the data were obtained and processed according to the field's best practice and are presented to reflect the results of the experiments in an accurate and unbiased manner.
- ideally, figure panels should include only measurements that are directly comparable to each other and obtained with the same assay.
- plots include clearly labeled error bars for independent experiments and sample sizes. Unless justified, error bars should not be shown for technical
- if n<5, the individual data points from each experiment should be plotted. Any statistical test employed should be justified.
- Source Data should be included to report the data underlying figures according to the guidelines set out in the authorship guidelines on Data

2. Captions
Each figure caption should contain the following information, for each panel where they are relevant:
- a specification of the experimental system investigated (eg cell line, species name).
- the assay(s) and method(s) used to carry out the reported observations and measurements.
- an explicit mention of the biological and chemical entity(ies) that are being measured.
- an explicit mention of the biological and chemical entity(ies) that are altered/varied/perturbed in a controlled manner.
- the exact sample size (n) for each experimental group/condition, given as a number, not a range;
- a description of the sample collection allowing the reader to understand whether the samples represent technical or biological replicates (including how many animals, litters, cultures, etc.).
- a statement of how many times the experiment shown was independently replicated in the laboratory.
- definitions of statistical methods and measures:
  - common tests, such as t-test (please specify whether paired vs. unpaired), simple χ2 tests, Wilcoxon and Mann-Whitney tests, can be unambiguously identified by name only, but more complex techniques should be described in the methods section;
  - are tests one-sided or two-sided?
  - are there adjustments for multiple comparisons?
  - exact statistical test results, e.g., P values = x but not P values < x;
  - definition of 'center values' as median or average;
  - definition of error bars as s.d. or s.e.m.

Please complete ALL of the questions below.
Select "Not Applicable" only when the requested information is not relevant for your study.

Materials

Newly Created Materials	Information included in the manuscript?	In which section is the information available? (Reagents and Tools Table, Materials and Methods, Figures, Data Availability Section)
New materials and reagents need to be available; do any restrictions apply?	Not Applicable	

Antibodies	Information included in the manuscript?	In which section is the information available? (Reagents and Tools Table, Materials and Methods, Figures, Data Availability Section)
For **antibodies** provide the following information:<br>- Commercial antibodies: RRID (if possible) or supplier name, catalogue number and or/clone number<br>- Non-commercial: RRID or citation	Yes	Materials and methods

DNA and RNA sequences	Information included in the manuscript?	In which section is the information available? (Reagents and Tools Table, Materials and Methods, Figures, Data Availability Section)
**Short novel DNA or RNA including primers, probes**: provide the sequences.	Yes	Materials and methods

Cell materials	Information included in the manuscript?	In which section is the information available? (Reagents and Tools Table, Materials and Methods, Figures, Data Availability Section)
**Cell lines:** Provide species information, strain. Provide accession number in repository **OR** supplier name, catalog number, clone number, and/**OR** RRID.	Yes	Materials and methods
**Primary cultures:** Provide species, strain, sex of origin, genetic modification status.	Not Applicable	
Report if the cell lines were recently **authenticated** (e.g., by STR profiling) and tested for mycoplasma contamination.	Yes	Materials and methods

Experimental animals	Information included in the manuscript?	In which section is the information available? (Reagents and Tools Table, Materials and Methods, Figures, Data Availability Section)
**Laboratory animals or Model organisms:** Provide species, strain, sex, age, genetic modification status. Provide accession number in repository **OR** supplier name, catalog number, clone number, **OR** RRID.	Not Applicable	
**Animal observed in or captured from the field:** Provide species, sex, and age where possible.	Not Applicable	
Please detail **housing and husbandry conditions**.	Not Applicable	

Plants and microbes	Information included in the manuscript?	In which section is the information available? (Reagents and Tools Table, Materials and Methods, Figures, Data Availability Section)
**Plants:** provide species and strain, ecotype and cultivar where relevant, unique accession number if available, and source (including location for collected wild specimens).	Not Applicable	
**Microbes:** provide species and strain, unique accession number if available, and source.	Not Applicable	

Human research participants	Information included in the manuscript?	In which section is the information available? (Reagents and Tools Table, Materials and Methods, Figures, Data Availability Section)
If collected and within the bounds of privacy constraints report on age, sex and gender or ethnicity for all study participants.	Not Applicable	

Core facilities	Information included in the manuscript?	In which section is the information available? (Reagents and Tools Table, Materials and Methods, Figures, Data Availability Section)
If your work benefited from core facilities, was their service mentioned in the acknowledgments section?	Not Applicable	

Design

Study protocol	Information included in the manuscript?	In which section is the information available? (Reagents and Tools Table, Materials and Methods, Figures, Data Availability Section)

If study protocol has been **pre-registered, provide DOI in the manuscript**. For clinical trials, provide the trial registration number **OR** cite DOI.	Not Applicable	
Report the **clinical trial registration number** (at ClinicalTrials.gov or equivalent), where applicable.	Not Applicable	

Laboratory protocol	Information included in the manuscript?	In which section is the information available? (Reagents and Tools Table, Materials and Methods, Figures, Data Availability Section)
Provide DOI OR other citation details if **external detailed step-by-step protocols** are available.	Not Applicable	

Experimental study design and statistics	Information included in the manuscript?	In which section is the information available? (Reagents and Tools Table, Materials and Methods, Figures, Data Availability Section)
Include a statement about **sample size** estimate even if no statistical methods were used.	Not Applicable	
Were any steps taken to minimize the effects of subjective bias when allocating animals/samples to treatment (e.g. **randomization procedure**)? If yes, have they been described?	Not Applicable	
Include a statement about **blinding** even if no blinding was done.	Yes	Materials and methods
Describe **inclusion/exclusion criteria** if samples or animals were excluded from the analysis. Were the criteria pre-established?<br><br>If sample or data points were omitted from analysis, report if this was due to attrition or intentional exclusion and provide justification.	Not Applicable	
For every figure, are **statistical tests** justified as appropriate? Do the data meet the assumptions of the tests (e.g., normal distribution)? Describe any methods used to assess it. Is there an estimate of variation within each group of data? Is the variance similar between the groups that are being statistically compared?	Yes	Materials and methods

Sample definition and in-laboratory replication	Information included in the manuscript?	In which section is the information available? (Reagents and Tools Table, Materials and Methods, Figures, Data Availability Section)
In the figure legends: state number of times the experiment was **replicated** in laboratory.	Yes	figure legends and methods
In the figure legends: define whether data describe **technical or biological replicates**.	Yes	figure legends and methods

Ethics

Ethics	Information included in the manuscript?	In which section is the information available? (Reagents and Tools Table, Materials and Methods, Figures, Data Availability Section)
Studies involving **human participants**: State details of **authority granting ethics approval** (IRB or equivalent committee(s), provide reference number for approval.	Not Applicable	
Studies involving **human participants**: Include a statement confirming that **informed consent** was obtained from all subjects and that the experiments conformed to the principles set out in the WMA Declaration of Helsinki and the Department of Health and Human Services Belmont Report.	Not Applicable	
Studies involving **human participants:** For publication of **patient photos**, include a statement confirming that consent to publish was obtained.	Not Applicable	
Studies involving experimental **animals**: State details of **authority granting ethics approval** (IRB or equivalent committee(s), provide reference number for approval. Include a statement of compliance with ethical regulations.	Not Applicable	
Studies involving **specimen and field samples:** State if relevant **permits** obtained, provide details of authority approving study; if none were required, explain why.	Not Applicable	

Dual Use Research of Concern (DURC)	Information included in the manuscript?	In which section is the information available? (Reagents and Tools Table, Materials and Methods, Figures, Data Availability Section)
Could your study fall under dual use research restrictions? Please check biosecurity documents and list of **select agents and toxins** (CDC): https://www.selectagents.gov/sat/list.htm	Not Applicable	
If you used a select agent, is the security level of the lab appropriate and reported in the manuscript?	Not Applicable	
If a study is subject to dual use research of concern regulations, is the name of the **authority granting approval and reference number** for the regulatory approval provided in the manuscript?	Not Applicable	

Reporting

The MDAR framework recommends adoption of discipline-specific guidelines, established and endorsed through community initiatives. Journals have their own policy about requiring specific guidelines and recommendations to complement MDAR.

Adherence to community standards	Information included in the manuscript?	In which section is the information available? (Reagents and Tools Table, Materials and Methods, Figures, Data Availability Section)
State if relevant guidelines or checklists (e.g., **ICMJE, MIBBI, ARRIVE, PRISMA**) have been followed or provided.	Not Applicable	
For **tumor marker prognostic studies,** we recommend that you follow the **REMARK** reporting guidelines (see link list at top right). See author guidelines, under 'Reporting Guidelines'. Please confirm you have followed these guidelines.	Not Applicable	
For **phase II and III randomized controlled trials**, please refer to the **CONSORT** flow diagram (see link list at top right) and submit the CONSORT checklist (see link list at top right) with your submission. See author guidelines, under 'Reporting Guidelines'. Please confirm you have submitted this list.	Not Applicable	

Data Availability

Data availability	Information included in the manuscript?	In which section is the information available? (Reagents and Tools Table, Materials and Methods, Figures, Data Availability Section)
Have **primary datasets** been deposited according to the journal's guidelines (see 'Data Deposition' section) and the respective accession numbers provided in the Data Availability Section?	Not Applicable	
Were **human clinical and genomic datasets** deposited in a public access-controlled repository in accordance to ethical obligations to the patients and to the applicable consent agreement?	Not Applicable	
Are **computational models** that are central and integral to a study available without restrictions in a machine-readable form? Were the relevant accession numbers or links provided?	Not Applicable	
If publicly available data were reused, provide the respective **data citations in the reference list**.	Not Applicable	
