# Supplementary figures and images for: MTCH2 cooperates with MFN2 and lysophosphatidic acid synthesis to sustain mitochondrial fusion (part 1 of 6)
Source: EMBO Rep. 2023 Dec 14;25(1):8. doi: 10.1038/s44319-023-00009-1 (PMC10897490; doi:10.1038/s44319-023-00009-1)

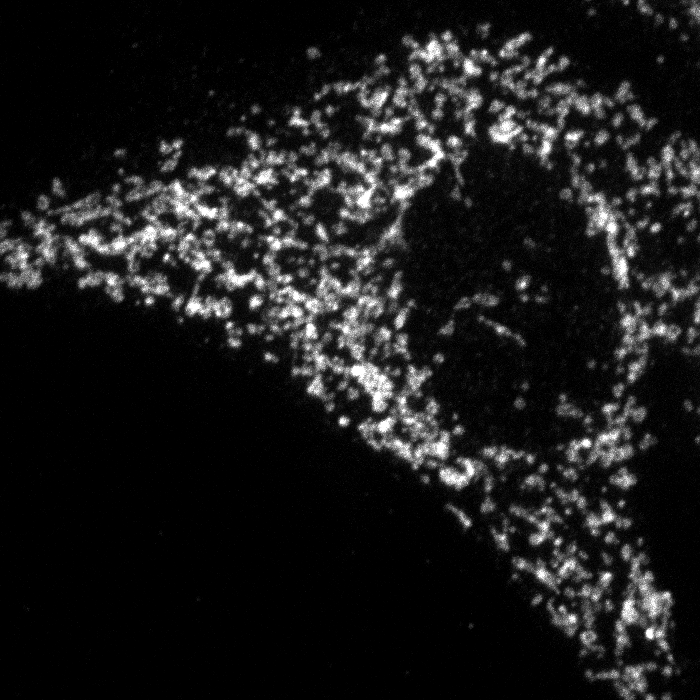

Supplement: Supplementary file 2 — Source Data Fig. 2 [file 44319_2023_9_MOESM2_ESM.zip › fig 1/a/images/mfn1 ko control.tif (RGB).tif]

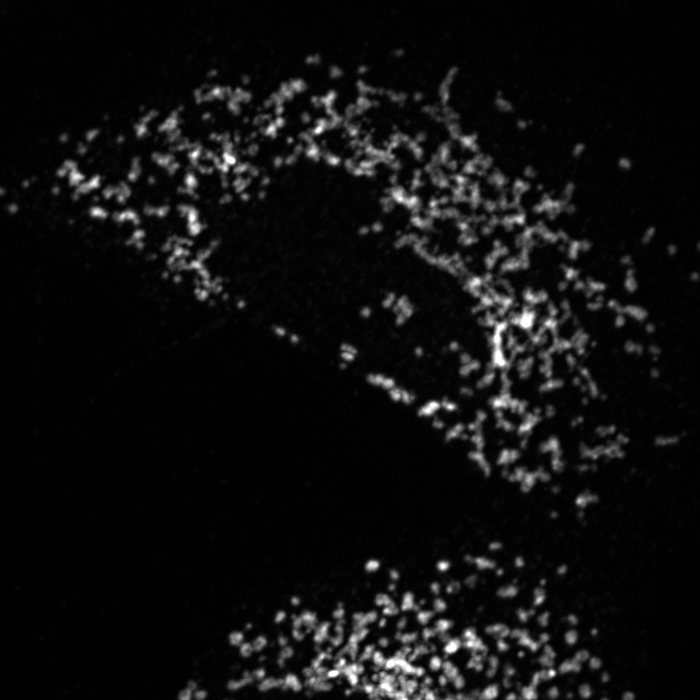

Supplement: Supplementary file 2 — Source Data Fig. 2 [file 44319_2023_9_MOESM2_ESM.zip › fig 1/a/images/MFN1 KO MTCH2 GFP.tif]

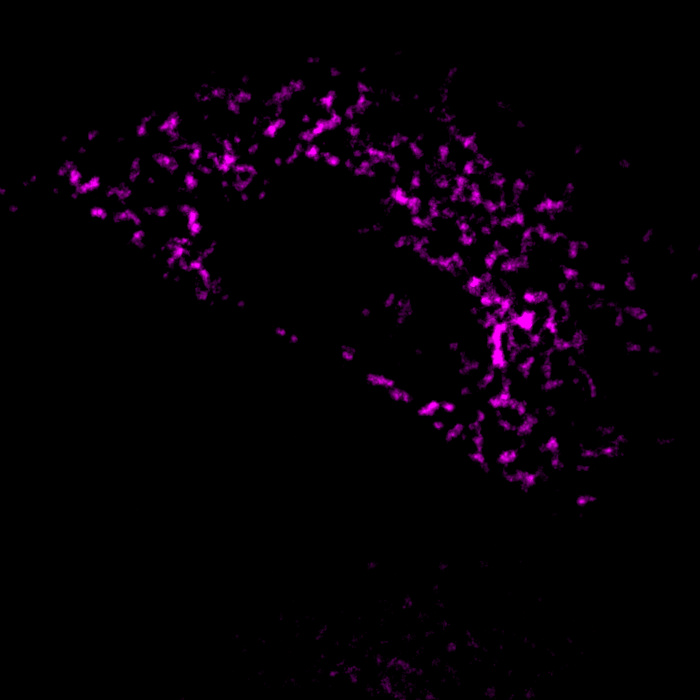

Supplement: Supplementary file 2 — Source Data Fig. 2 [file 44319_2023_9_MOESM2_ESM.zip › fig 1/a/images/MFN1 KO MTCH2 GFP.tif (RGB) mtch2.tif]

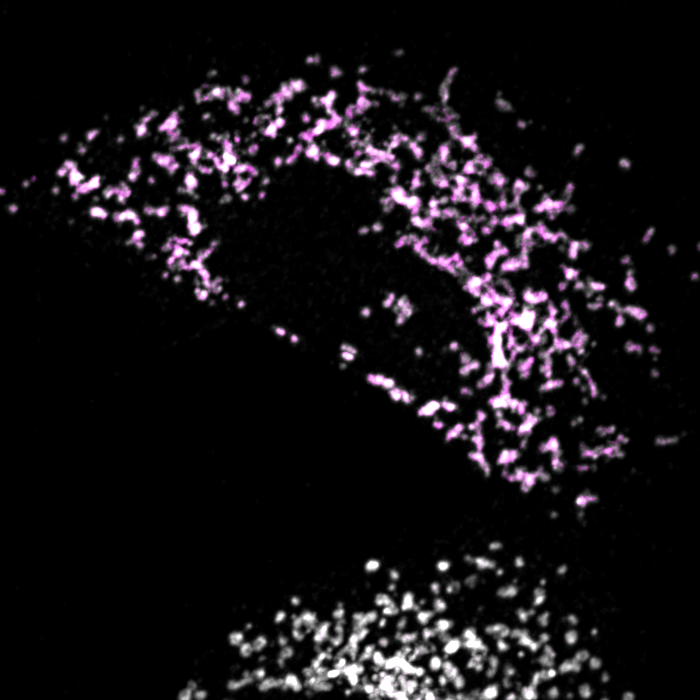

Supplement: Supplementary file 2 — Source Data Fig. 2 [file 44319_2023_9_MOESM2_ESM.zip › fig 1/a/images/MFN1 KO MTCH2 GFP.tif (RGB)composite.tif]

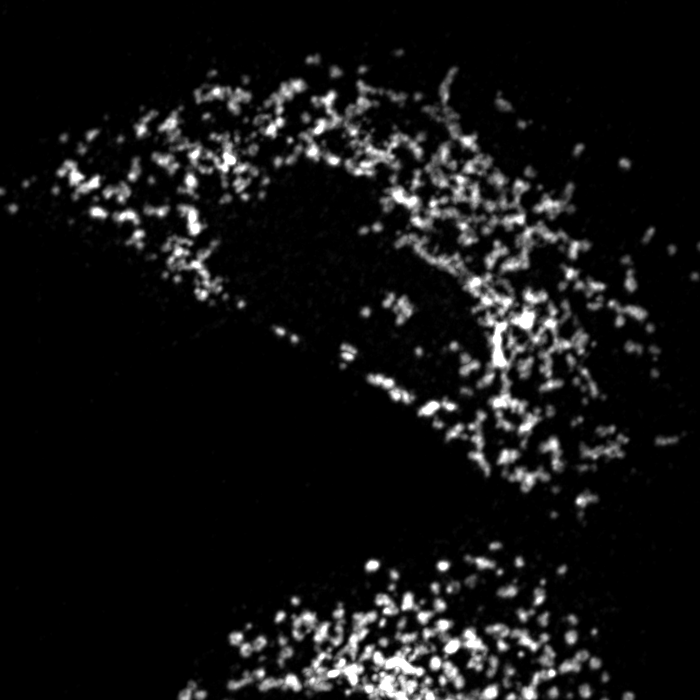

Supplement: Supplementary file 2 — Source Data Fig. 2 [file 44319_2023_9_MOESM2_ESM.zip › fig 1/a/images/MFN1 KO MTCH2 GFP.tif (RGB)tom20.tif]

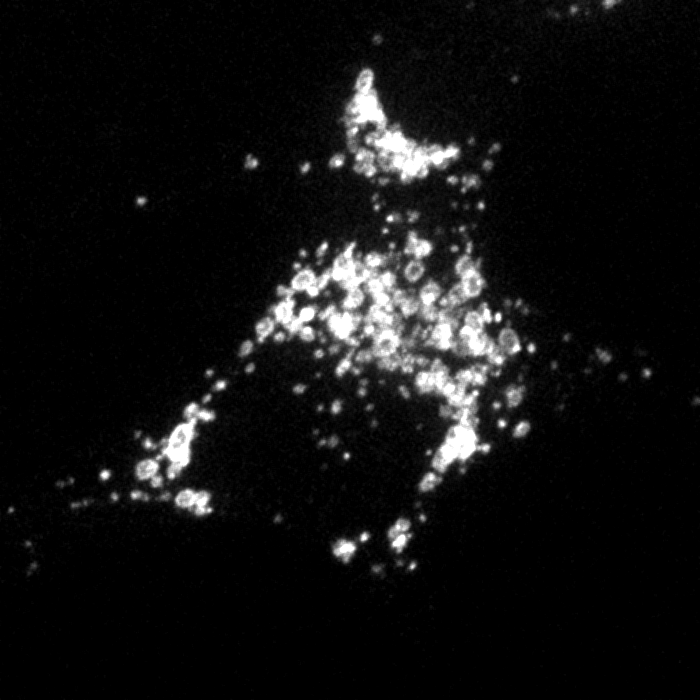

Supplement: Supplementary file 2 — Source Data Fig. 2 [file 44319_2023_9_MOESM2_ESM.zip › fig 1/a/images/mfn12 dko ctrlopt 3.tif (RGB).tif]

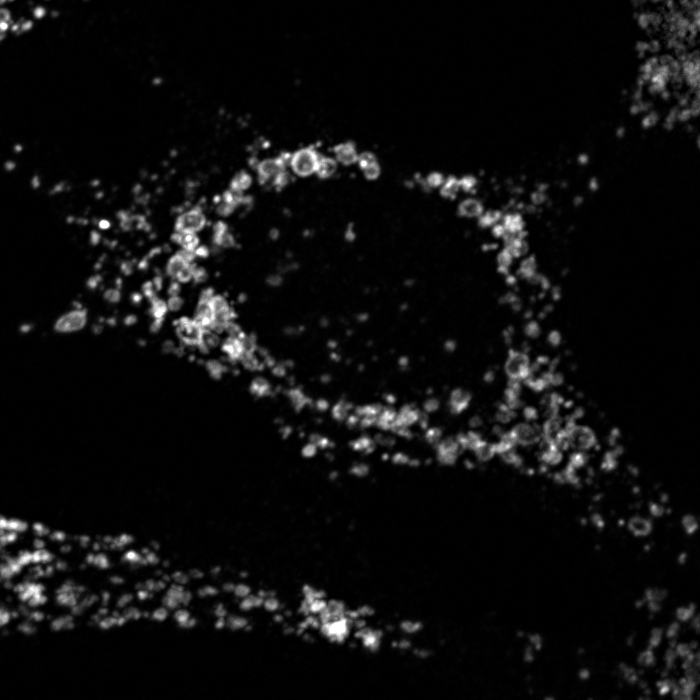

Supplement: Supplementary file 2 — Source Data Fig. 2 [file 44319_2023_9_MOESM2_ESM.zip › fig 1/a/images/MFN12 DKO MTCH2 GFP.tif]

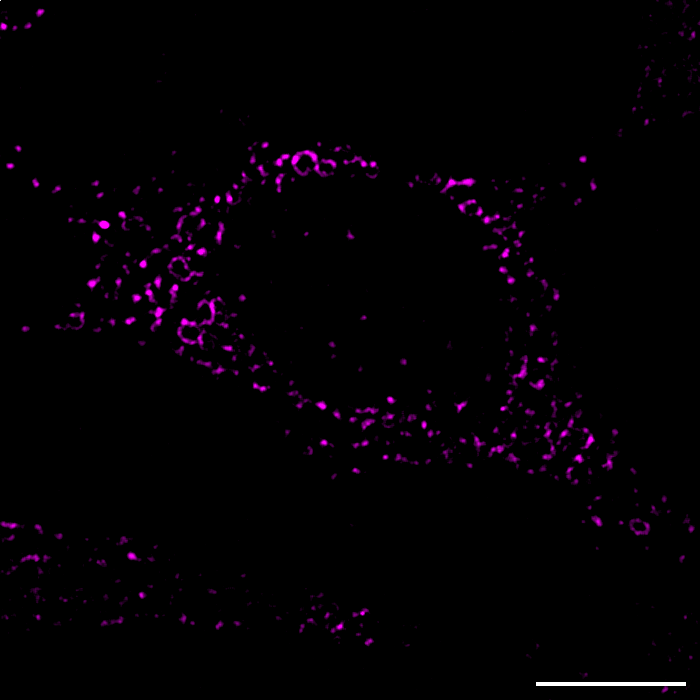

Supplement: Supplementary file 2 — Source Data Fig. 2 [file 44319_2023_9_MOESM2_ESM.zip › fig 1/a/images/MFN12 DKO MTCH2 GFP.tif (RGB) mtxg2 gfp-1scale.tif]

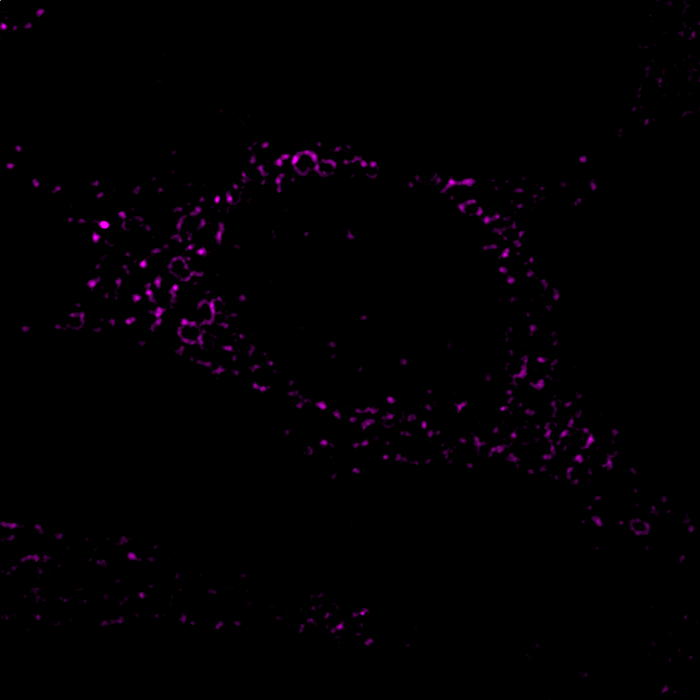

Supplement: Supplementary file 2 — Source Data Fig. 2 [file 44319_2023_9_MOESM2_ESM.zip › fig 1/a/images/MFN12 DKO MTCH2 GFP.tif (RGB) mtxg2 gfp.tif]

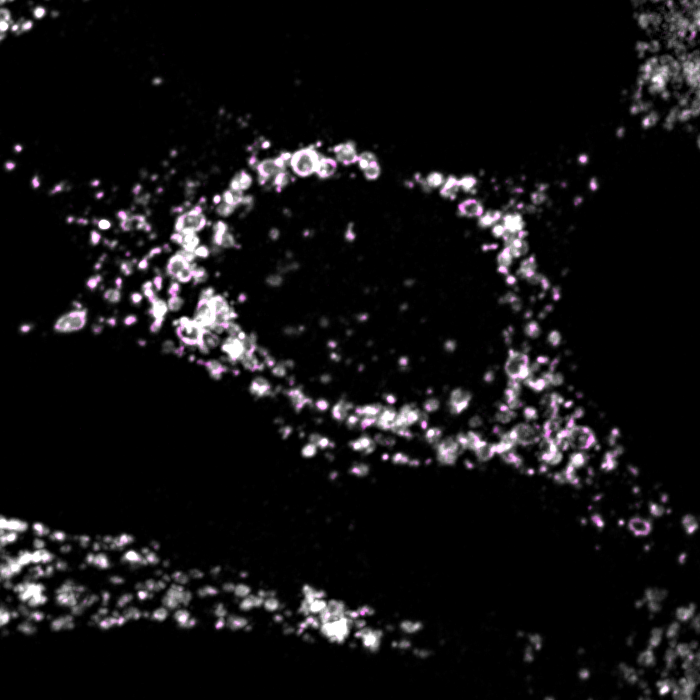

Supplement: Supplementary file 2 — Source Data Fig. 2 [file 44319_2023_9_MOESM2_ESM.zip › fig 1/a/images/MFN12 DKO MTCH2 GFP.tif (RGB)comp.tif]

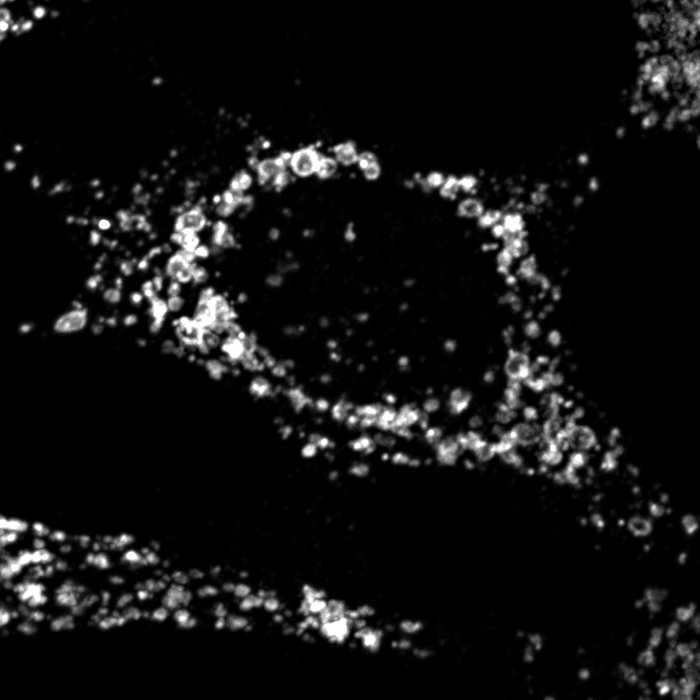

Supplement: Supplementary file 2 — Source Data Fig. 2 [file 44319_2023_9_MOESM2_ESM.zip › fig 1/a/images/MFN12 DKO MTCH2 GFP.tif (RGB)tomm20.tif]

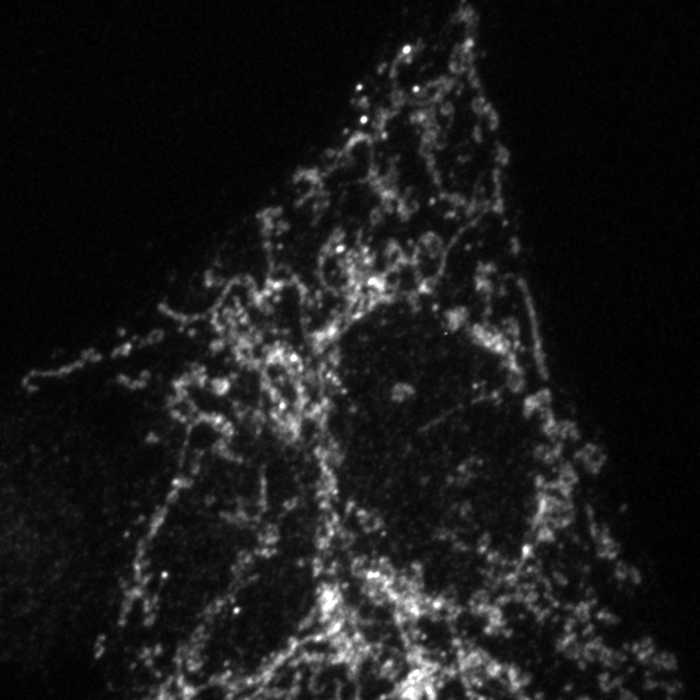

Supplement: Supplementary file 2 — Source Data Fig. 2 [file 44319_2023_9_MOESM2_ESM.zip › fig 1/a/images/mfn2 ko mtch2 op1.tif]

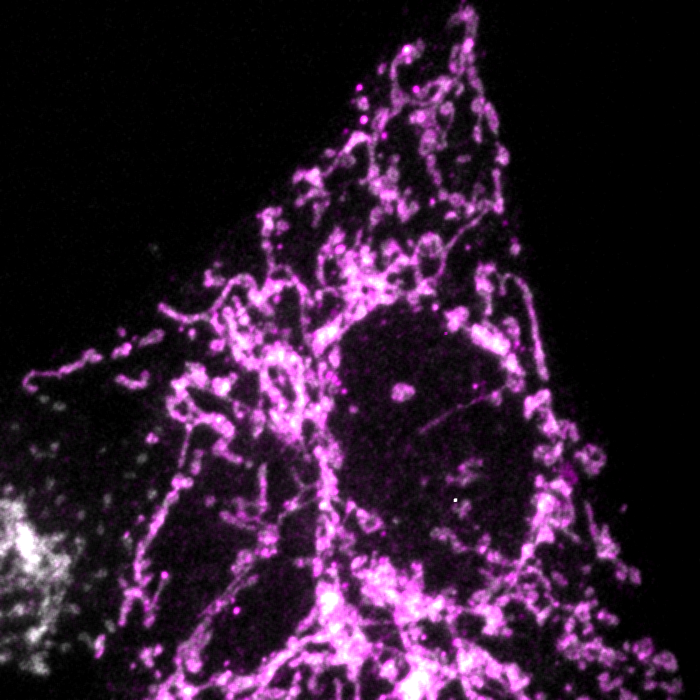

Supplement: Supplementary file 2 — Source Data Fig. 2 [file 44319_2023_9_MOESM2_ESM.zip › fig 1/a/images/mfn2 ko mtch2 op1.tif (RGB) compo.tif]

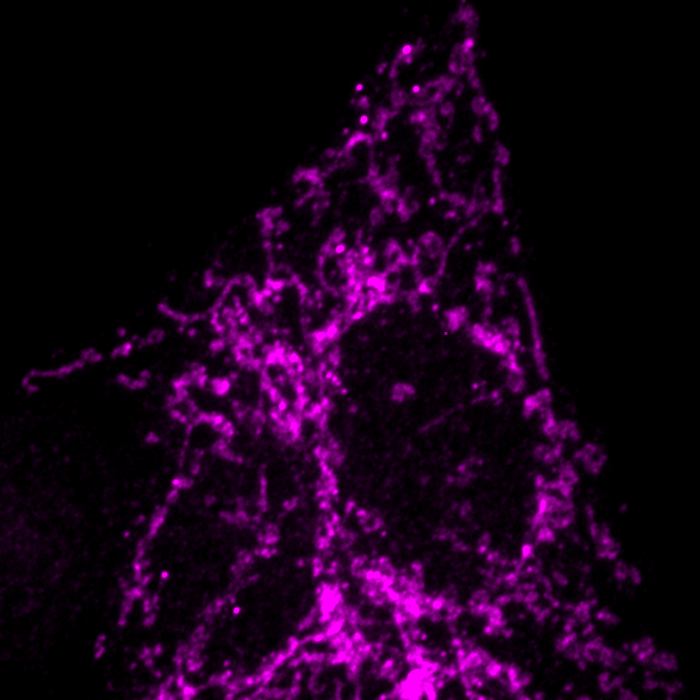

Supplement: Supplementary file 2 — Source Data Fig. 2 [file 44319_2023_9_MOESM2_ESM.zip › fig 1/a/images/mfn2 ko mtch2 op1.tif (RGB) mtch2.tif]

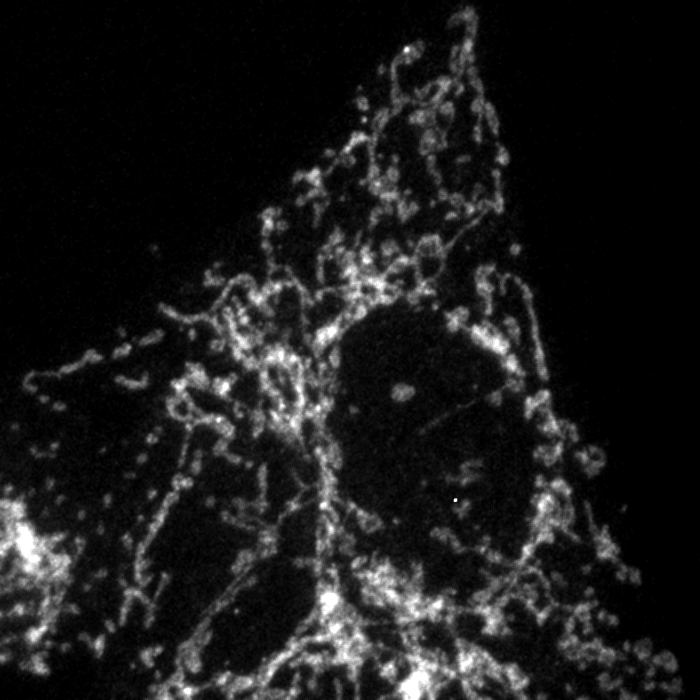

Supplement: Supplementary file 2 — Source Data Fig. 2 [file 44319_2023_9_MOESM2_ESM.zip › fig 1/a/images/mfn2 ko mtch2 op1.tif (RGB)tom20.tif]

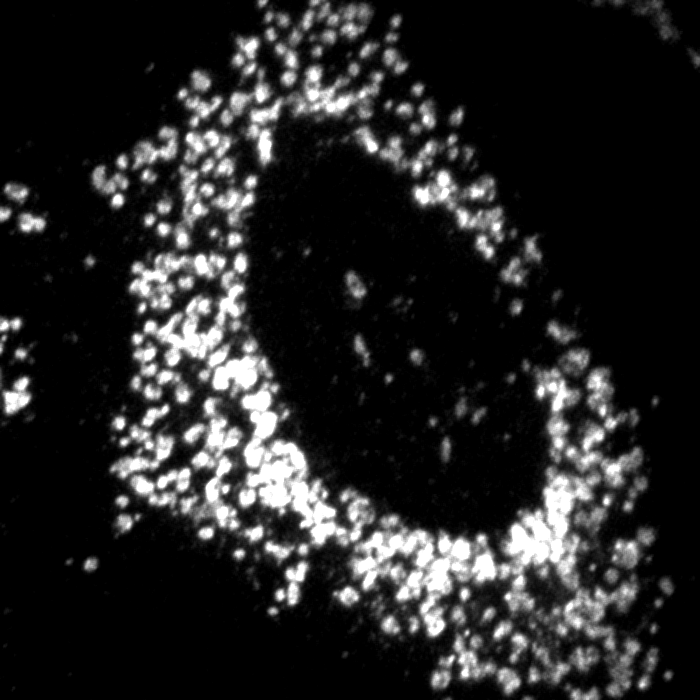

Supplement: Supplementary file 2 — Source Data Fig. 2 [file 44319_2023_9_MOESM2_ESM.zip › fig 1/a/images/mfn2 ko opt2.tif (RGB).tif]

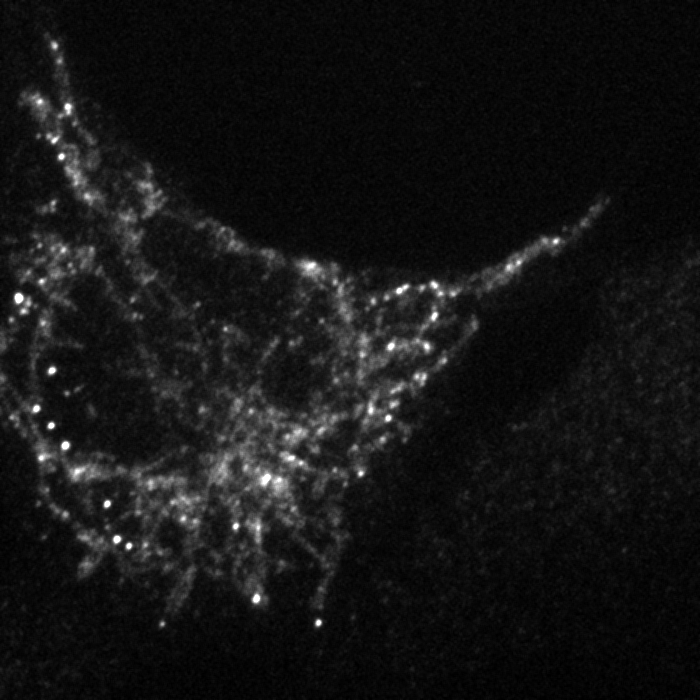

Supplement: Supplementary file 2 — Source Data Fig. 2 [file 44319_2023_9_MOESM2_ESM.zip › fig 1/a/images/mtch2 ko + mtch2 op4.tif]

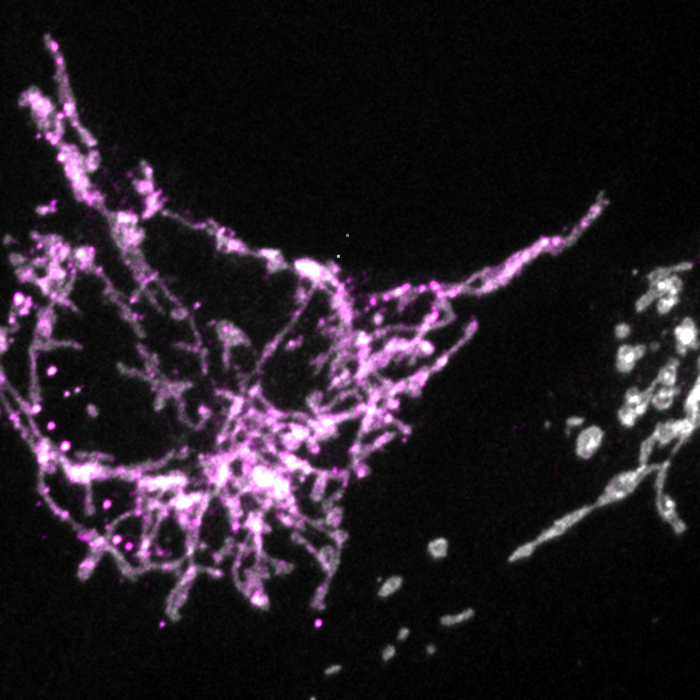

Supplement: Supplementary file 2 — Source Data Fig. 2 [file 44319_2023_9_MOESM2_ESM.zip › fig 1/a/images/mtch2 ko + mtch2 op4.tif (RGB)comp.tif]

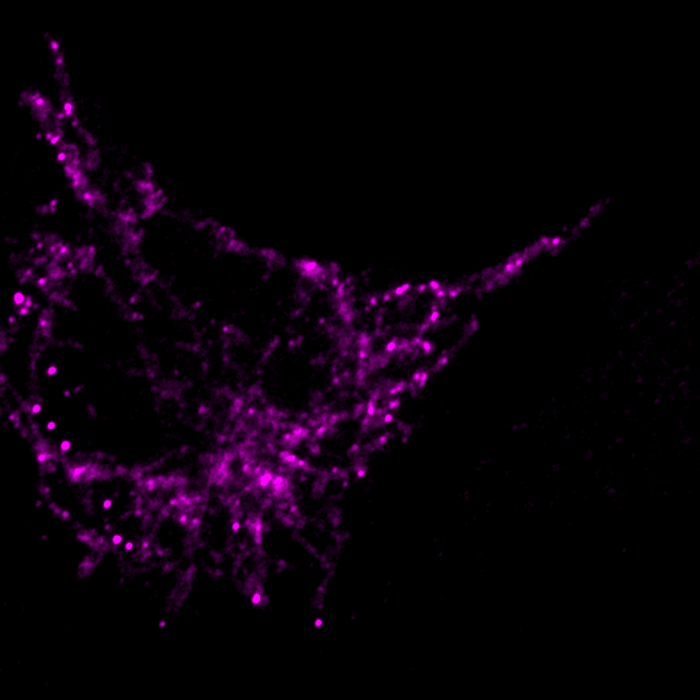

Supplement: Supplementary file 2 — Source Data Fig. 2 [file 44319_2023_9_MOESM2_ESM.zip › fig 1/a/images/mtch2 ko + mtch2 op4.tif (RGB)mtch2.tif]

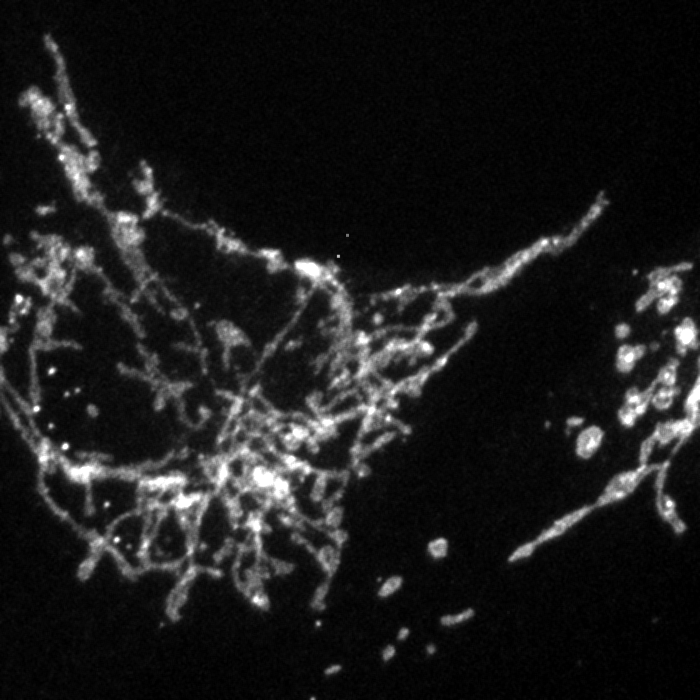

Supplement: Supplementary file 2 — Source Data Fig. 2 [file 44319_2023_9_MOESM2_ESM.zip › fig 1/a/images/mtch2 ko + mtch2 op4.tif (RGB)tom20.tif]

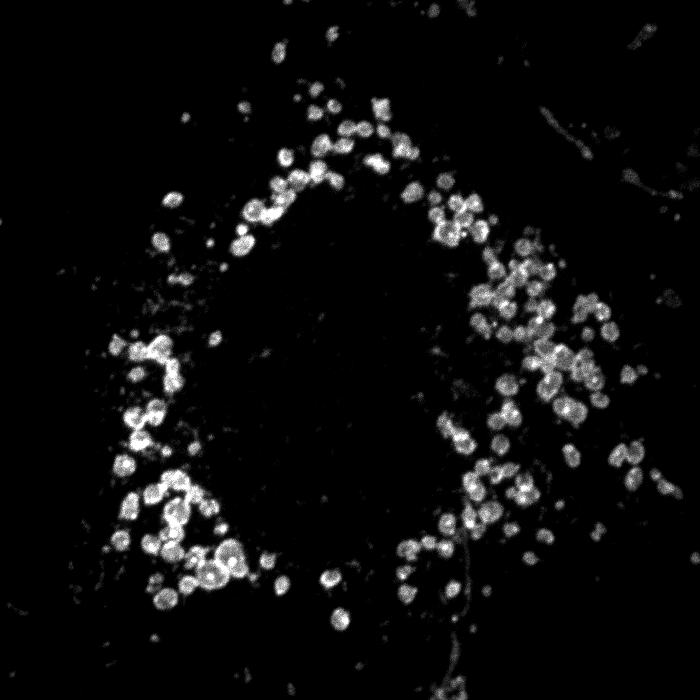

Supplement: Supplementary file 2 — Source Data Fig. 2 [file 44319_2023_9_MOESM2_ESM.zip › fig 1/a/images/mtch2 ko op4.tif (RGB).tif]

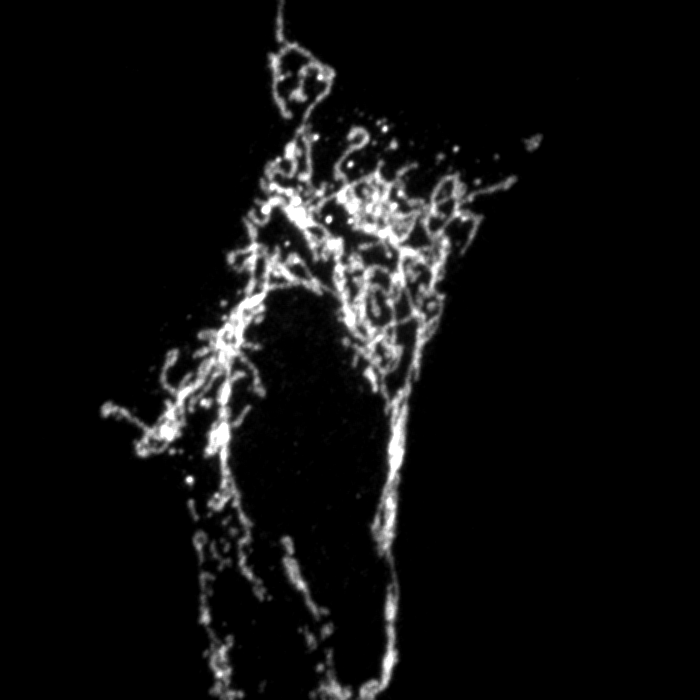

Supplement: Supplementary file 2 — Source Data Fig. 2 [file 44319_2023_9_MOESM2_ESM.zip › fig 1/a/images/wt controlop3.tif (RGB).tif]

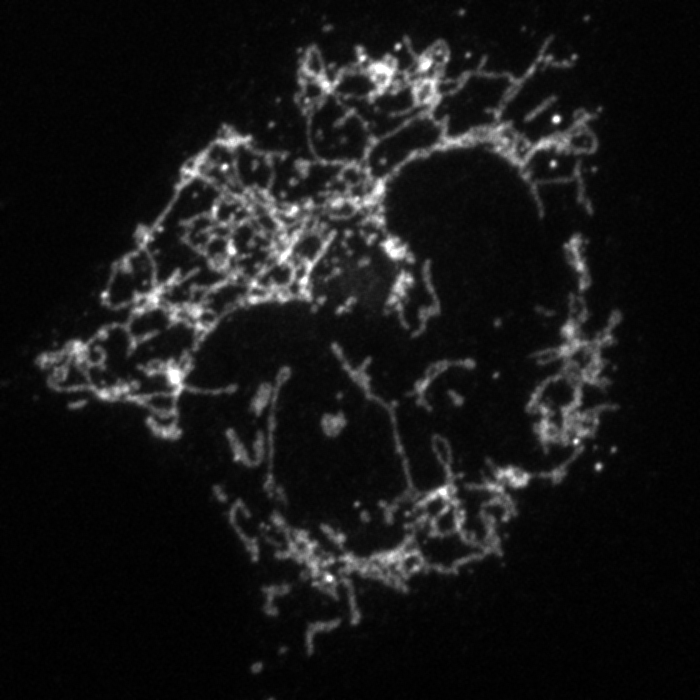

Supplement: Supplementary file 2 — Source Data Fig. 2 [file 44319_2023_9_MOESM2_ESM.zip › fig 1/a/images/wt mtch2 gfp op1.tif]

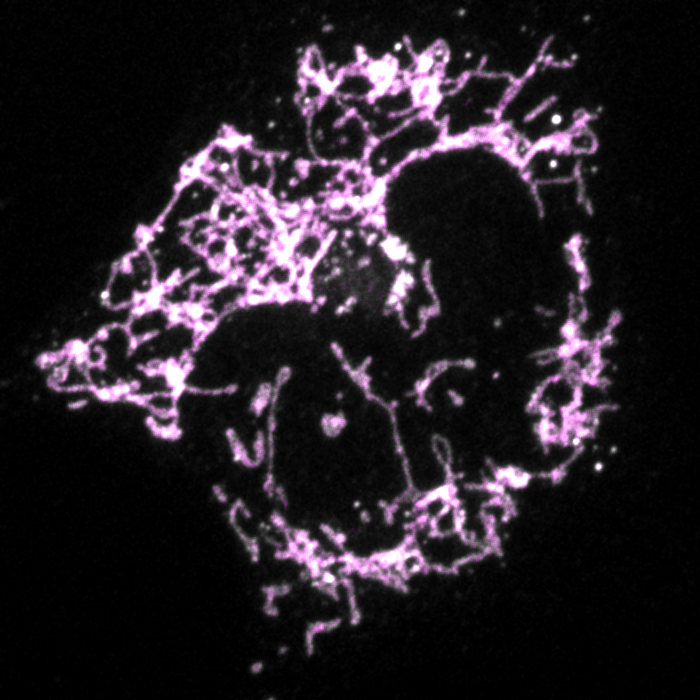

Supplement: Supplementary file 2 — Source Data Fig. 2 [file 44319_2023_9_MOESM2_ESM.zip › fig 1/a/images/wt mtch2 gfp op1.tif (RGB)comp.tif]

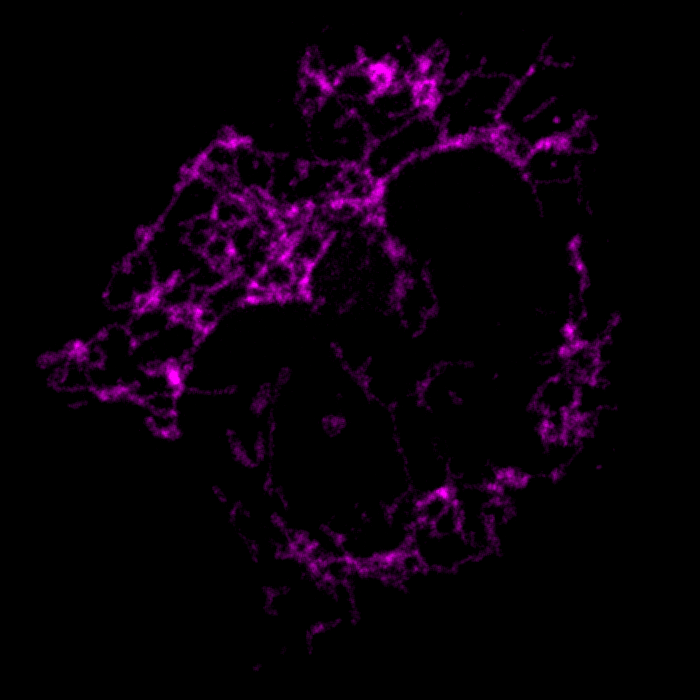

Supplement: Supplementary file 2 — Source Data Fig. 2 [file 44319_2023_9_MOESM2_ESM.zip › fig 1/a/images/wt mtch2 gfp op1.tif (RGB)mtch2.tif]

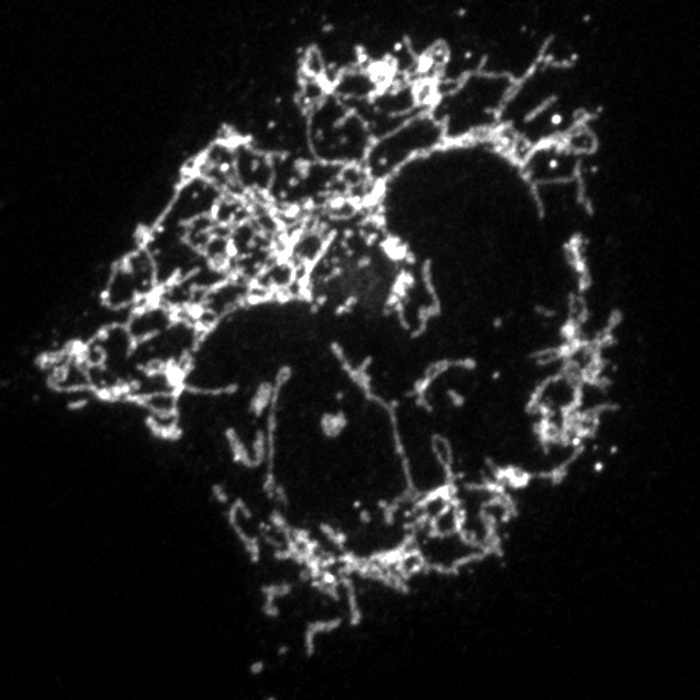

Supplement: Supplementary file 2 — Source Data Fig. 2 [file 44319_2023_9_MOESM2_ESM.zip › fig 1/a/images/wt mtch2 gfp op1.tif (RGB)tom20.tif]

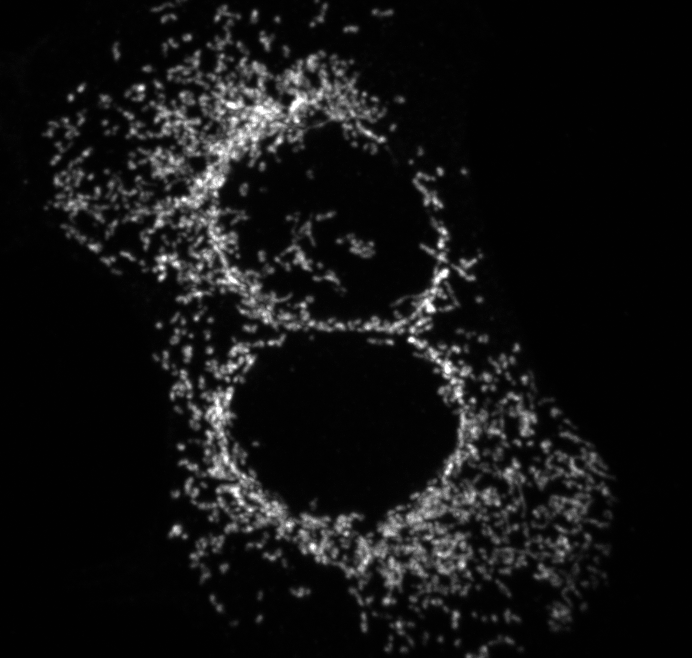

Supplement: Supplementary file 2 — Source Data Fig. 2 [file 44319_2023_9_MOESM2_ESM.zip › fig 1/d/IMAGES/MFN1 KO/control/MAX_MEFS mfn1 ko untaged mfn1 anti mfn1 gfp dapi22_thumb_w1Con-Cy5-1.tif (RGB).tif]

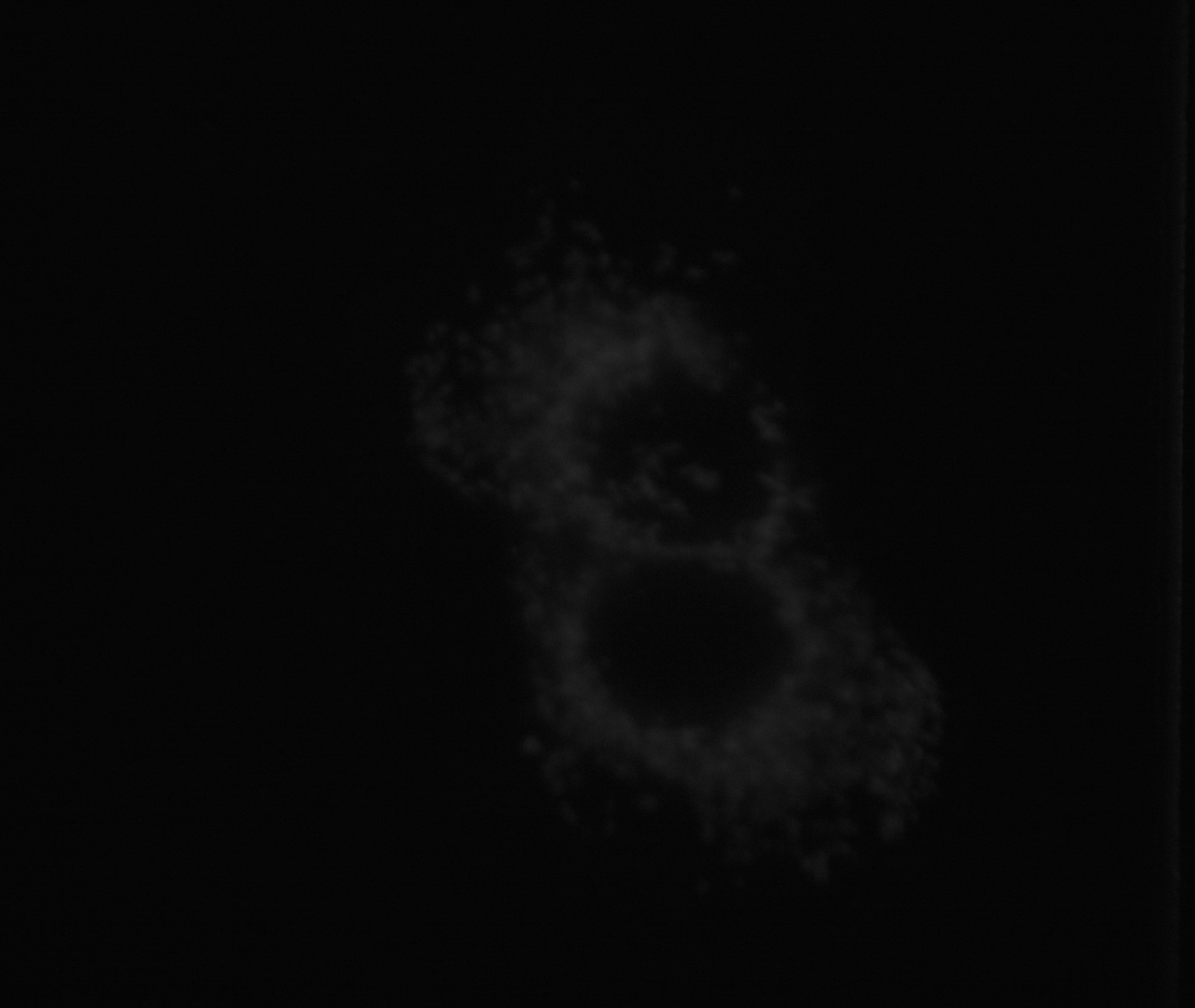

Supplement: Supplementary file 2 — Source Data Fig. 2 [file 44319_2023_9_MOESM2_ESM.zip › fig 1/d/IMAGES/MFN1 KO/control/MEFS mfn1 ko untaged mfn1 anti mfn1 gfp dapi22_thumb_w1Con-Cy5.tif]

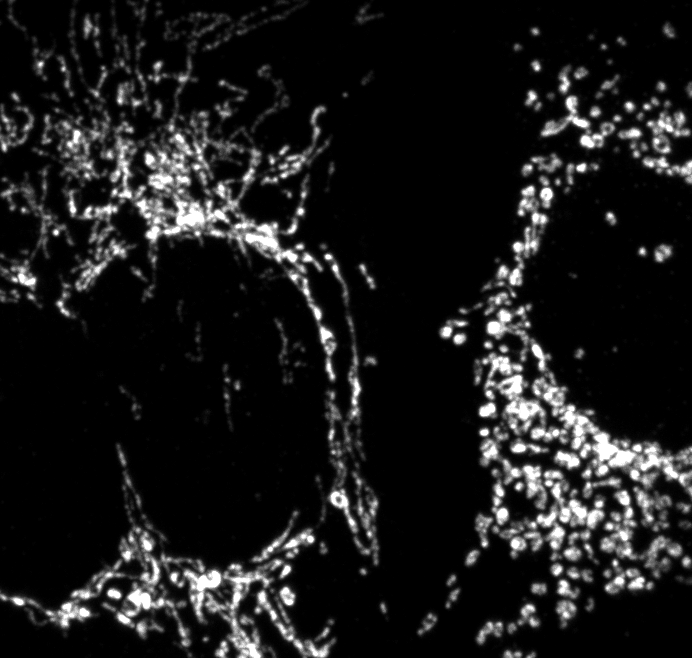

Supplement: Supplementary file 2 — Source Data Fig. 2 [file 44319_2023_9_MOESM2_ESM.zip › fig 1/d/IMAGES/MFN1 KO/transfected/MAX_MEFS mfn1 ko untaged mfn1 anti mfn1 gfp dapi5_thumb_w1Con-Cy5-1.tif (RGB MITO).tif]

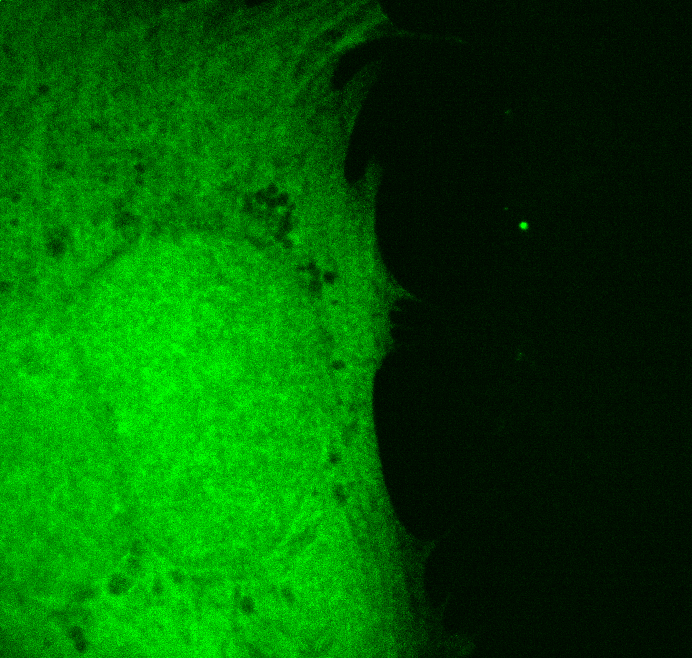

Supplement: Supplementary file 2 — Source Data Fig. 2 [file 44319_2023_9_MOESM2_ESM.zip › fig 1/d/IMAGES/MFN1 KO/transfected/MAX_MEFS mfn1 ko untaged mfn1 anti mfn1 gfp dapi5_thumb_w1Con-Cy5-1.tif (RGB) GFP.tif]

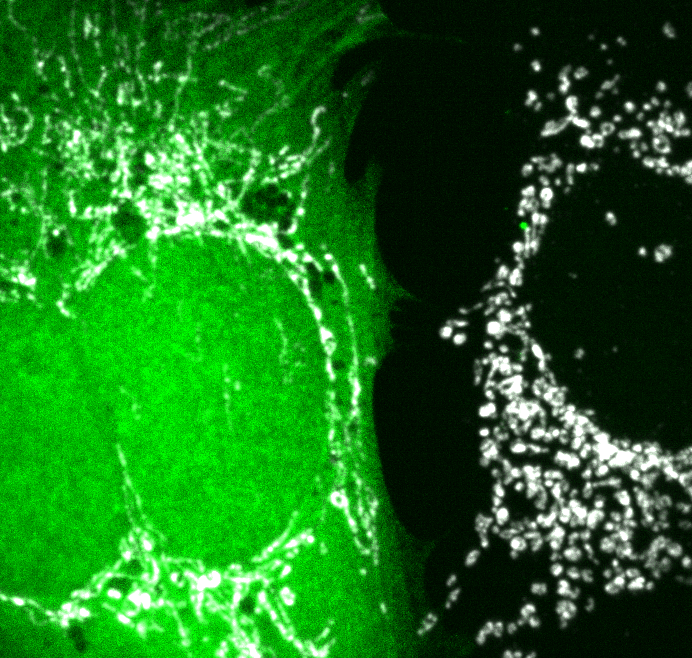

Supplement: Supplementary file 2 — Source Data Fig. 2 [file 44319_2023_9_MOESM2_ESM.zip › fig 1/d/IMAGES/MFN1 KO/transfected/MAX_MEFS mfn1 ko untaged mfn1 anti mfn1 gfp dapi5_thumb_w1Con-Cy5-1.tif (RGBCOMPOS).tif]

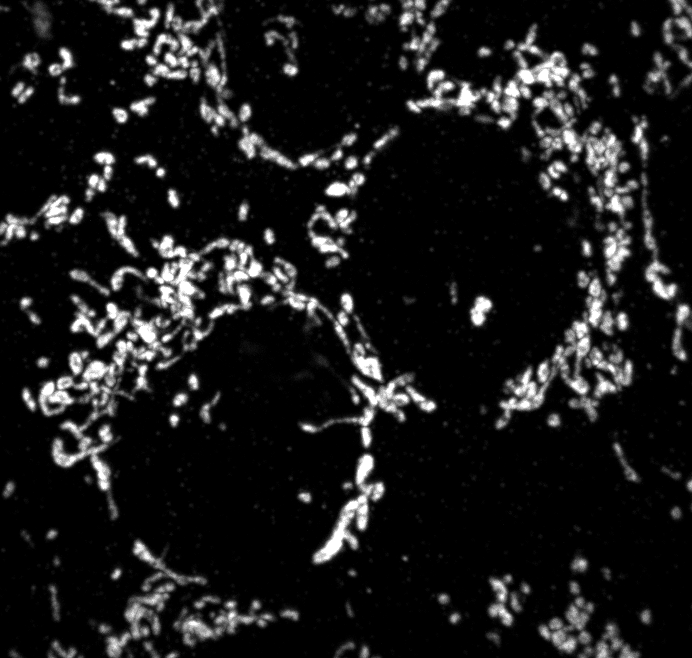

Supplement: Supplementary file 2 — Source Data Fig. 2 [file 44319_2023_9_MOESM2_ESM.zip › fig 1/d/IMAGES/MFN2 KO/CONTROL/MAX_MEFs mfn2 ko control tom598 cytc633 dapi1_thumb_w1Con-mcherry_s1.TIF - Stage11-1-1.tif]

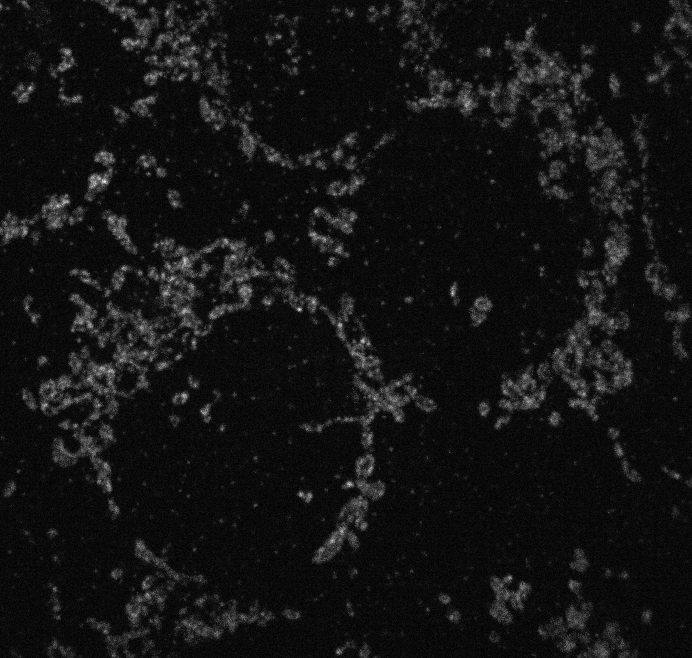

Supplement: Supplementary file 2 — Source Data Fig. 2 [file 44319_2023_9_MOESM2_ESM.zip › fig 1/d/IMAGES/MFN2 KO/CONTROL/MAX_MEFs mfn2 ko control tom598 cytc633 dapi1_thumb_w1Con-mcherry_s1.TIF - Stage11-1.tif]

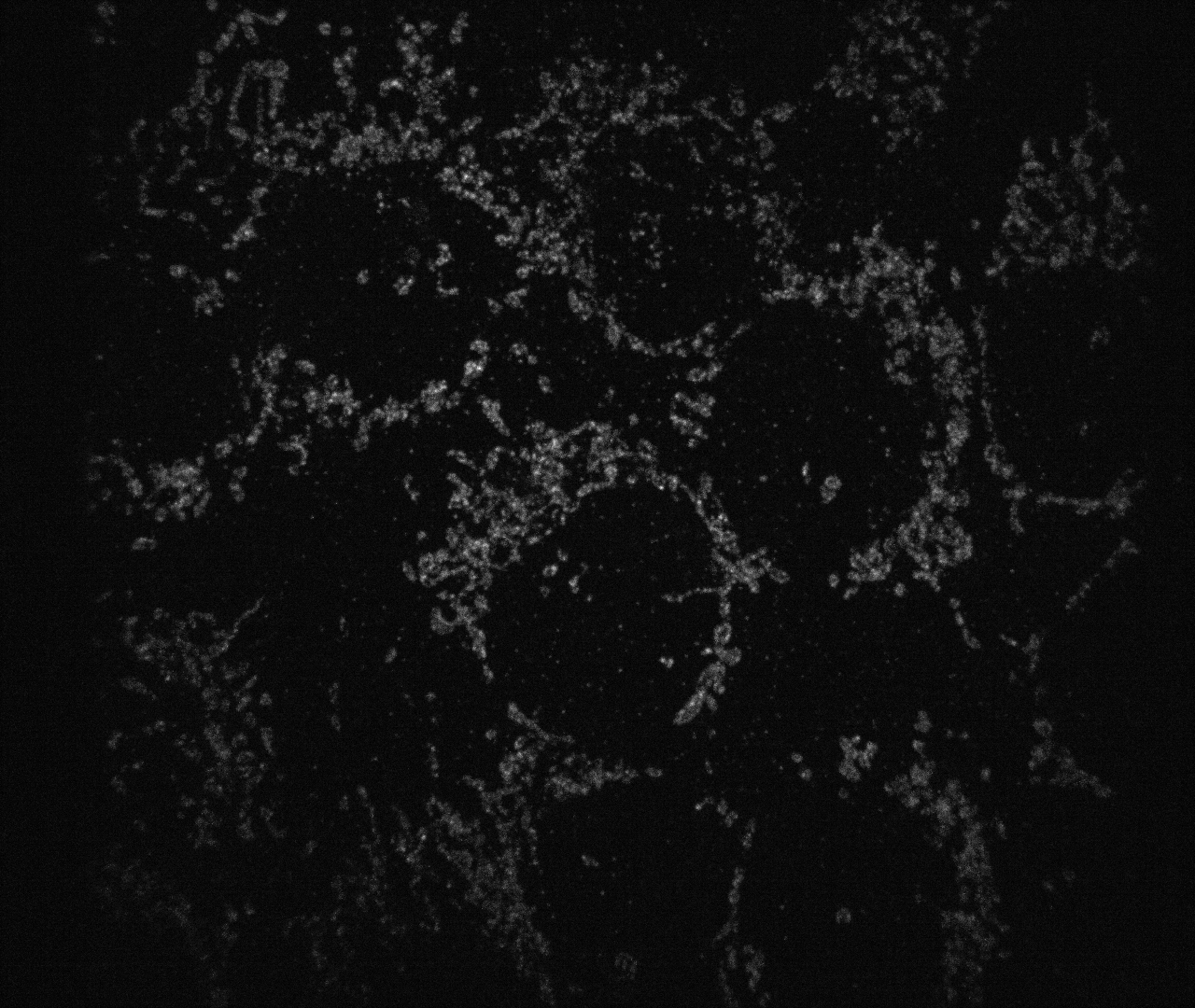

Supplement: Supplementary file 2 — Source Data Fig. 2 [file 44319_2023_9_MOESM2_ESM.zip › fig 1/d/IMAGES/MFN2 KO/CONTROL/MAX_MEFs mfn2 ko control tom598 cytc633 dapi1_thumb_w1Con-mcherry_s1.TIF - Stage11.tif]

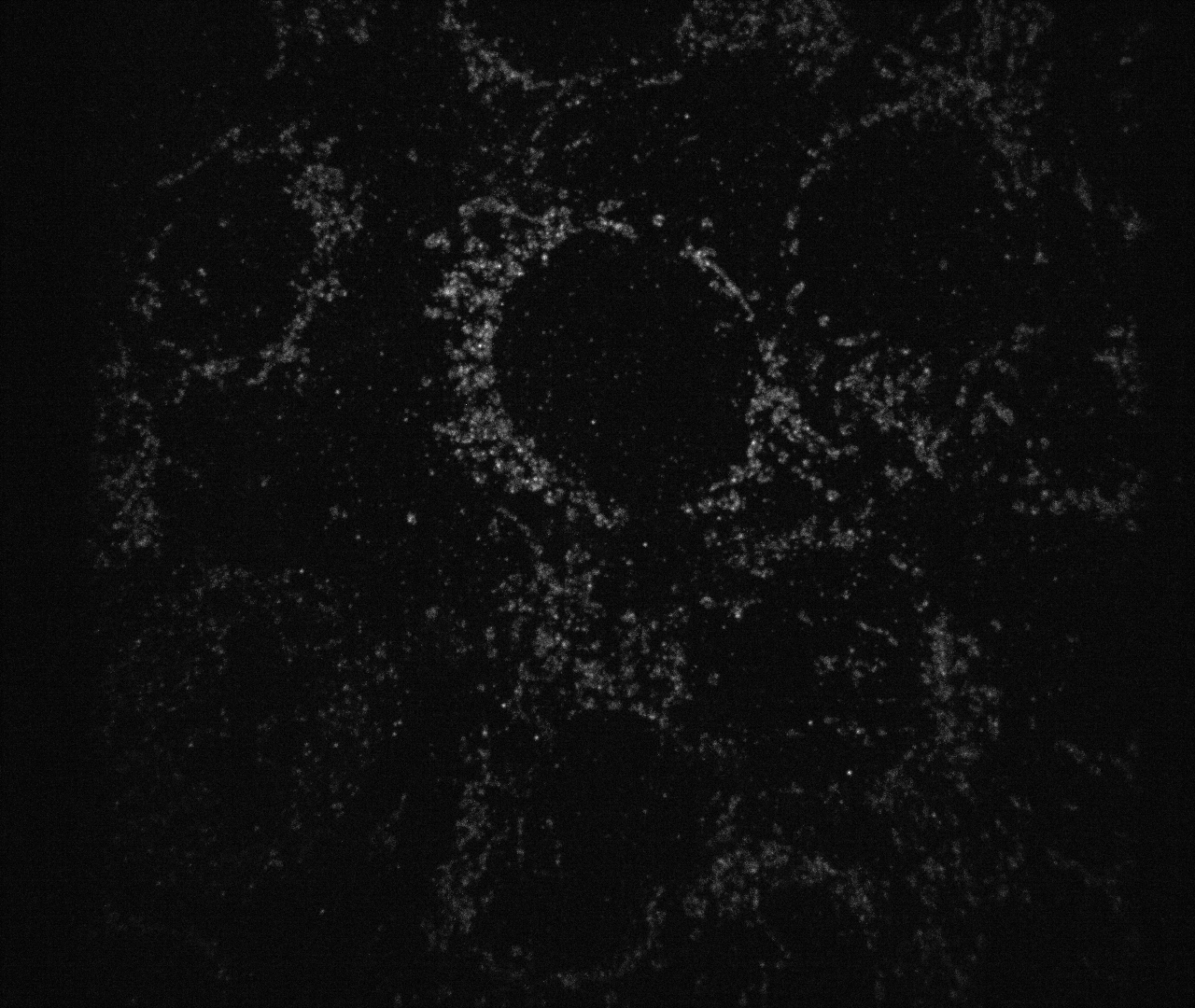

Supplement: Supplementary file 2 — Source Data Fig. 2 [file 44319_2023_9_MOESM2_ESM.zip › fig 1/d/IMAGES/MFN2 KO/CONTROL/MAX_MEFs mfn2 ko control tom598 cytc633 dapi1_thumb_w1Con-mcherry_s1.TIF - Stage12.tif]

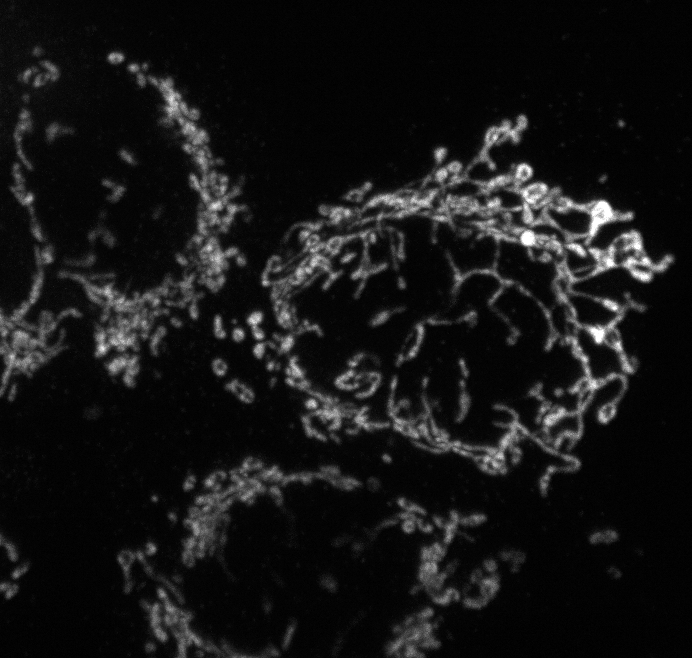

Supplement: Supplementary file 2 — Source Data Fig. 2 [file 44319_2023_9_MOESM2_ESM.zip › fig 1/d/IMAGES/MFN2 KO/TRANSF/MAX_mfn1 ko gfp mfn1 non tagged t40598 cuyt633_thumb_w1Con-Cy5-1.tif]

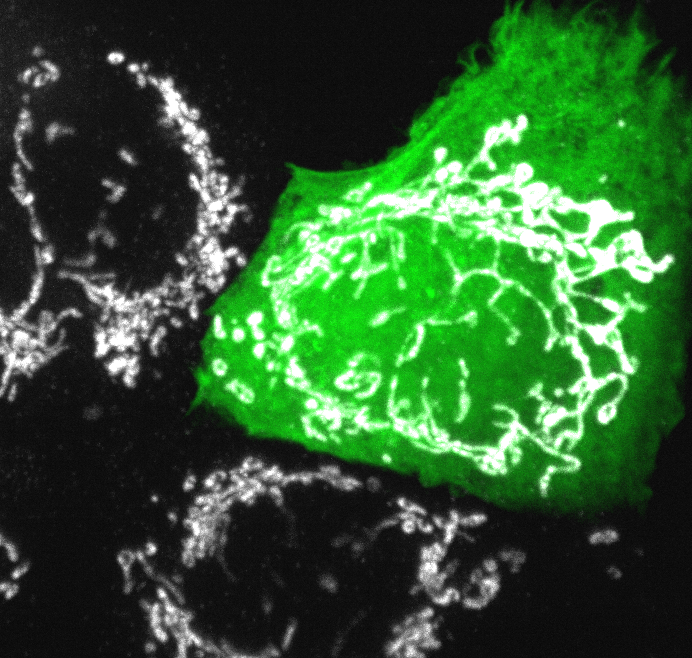

Supplement: Supplementary file 2 — Source Data Fig. 2 [file 44319_2023_9_MOESM2_ESM.zip › fig 1/d/IMAGES/MFN2 KO/TRANSF/MAX_mfn1 ko gfp mfn1 non tagged t40598 cuyt633_thumb_w1Con-Cy5-1.tif (RGB COMPO).tif]

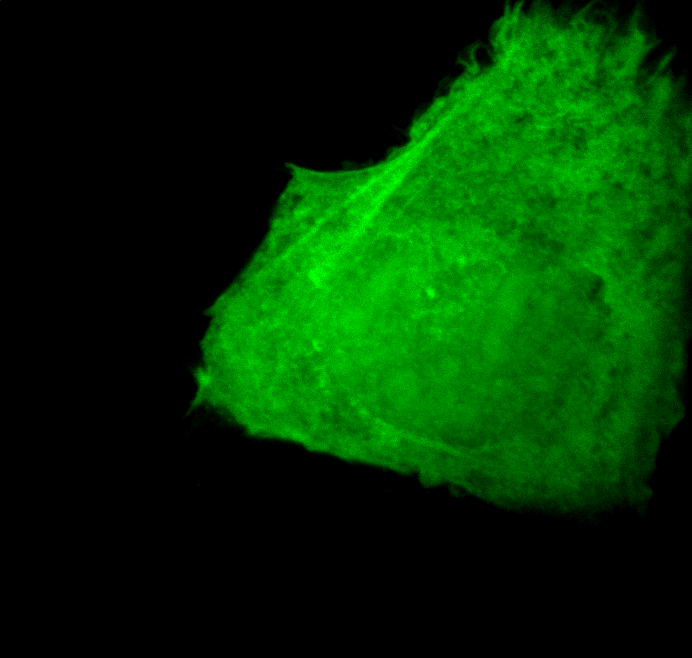

Supplement: Supplementary file 2 — Source Data Fig. 2 [file 44319_2023_9_MOESM2_ESM.zip › fig 1/d/IMAGES/MFN2 KO/TRANSF/MAX_mfn1 ko gfp mfn1 non tagged t40598 cuyt633_thumb_w1Con-Cy5-1.tif (RGB) CH1.tif]

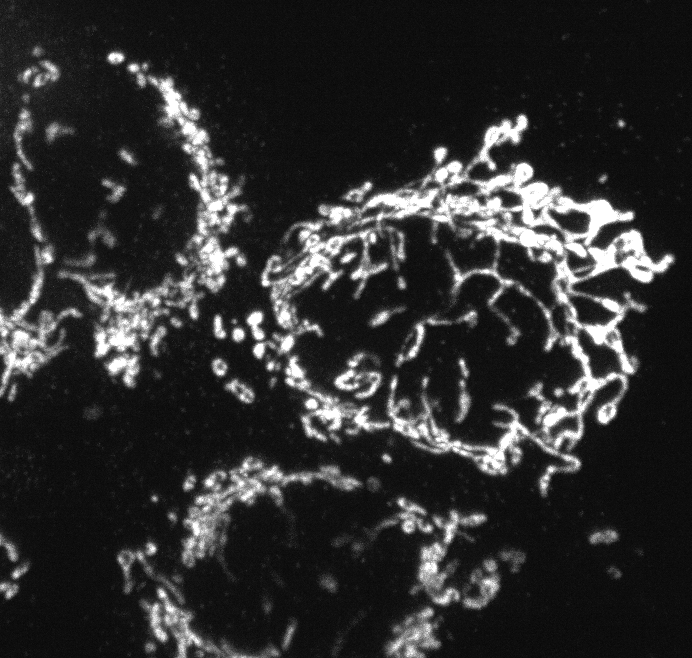

Supplement: Supplementary file 2 — Source Data Fig. 2 [file 44319_2023_9_MOESM2_ESM.zip › fig 1/d/IMAGES/MFN2 KO/TRANSF/MAX_mfn1 ko gfp mfn1 non tagged t40598 cuyt633_thumb_w1Con-Cy5-1.tif (RGB)C 2.tif]

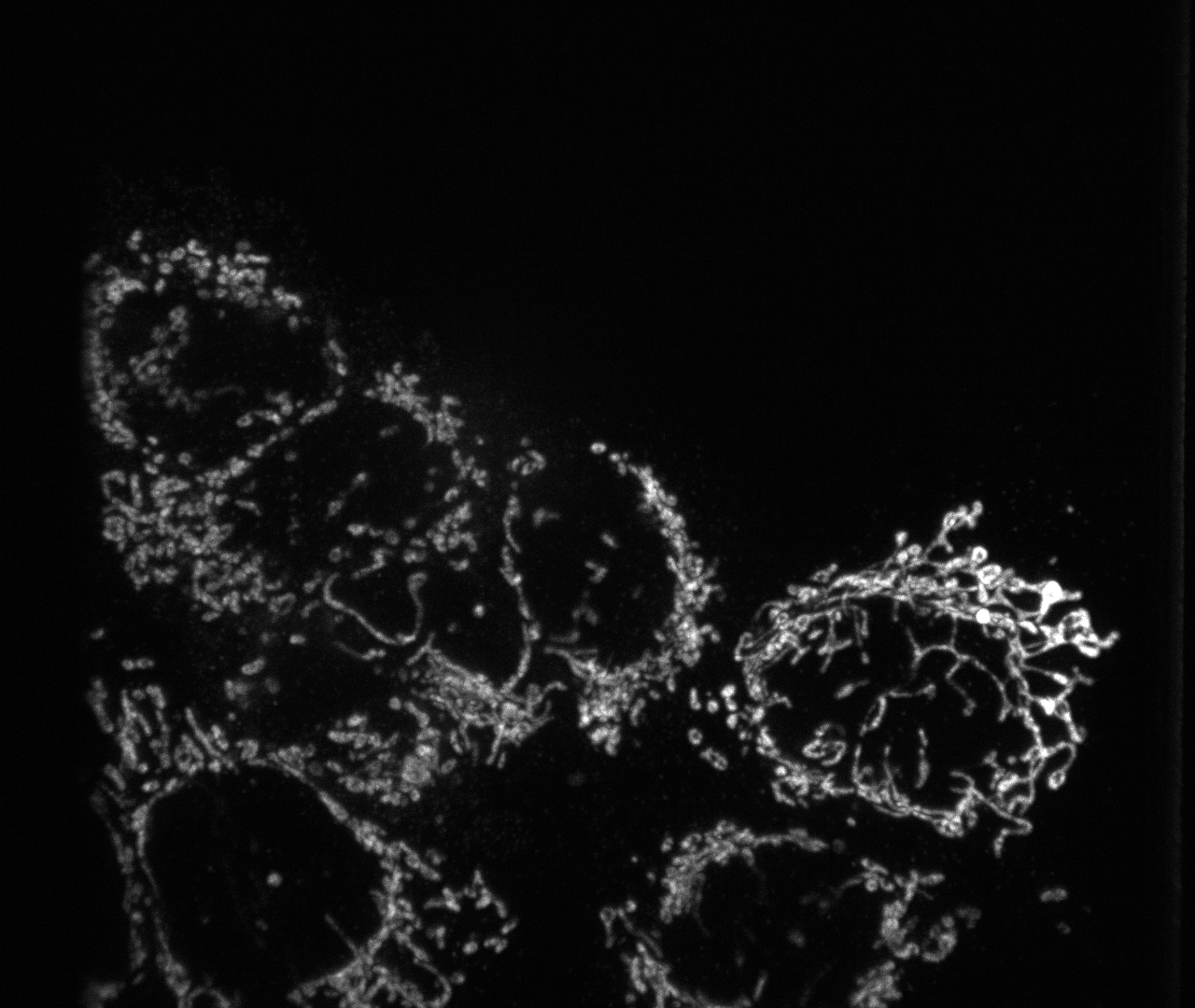

Supplement: Supplementary file 2 — Source Data Fig. 2 [file 44319_2023_9_MOESM2_ESM.zip › fig 1/d/IMAGES/MFN2 KO/TRANSF/MAX_mfn1 ko gfp mfn1 non tagged t40598 cuyt633_thumb_w1Con-Cy5.tif]

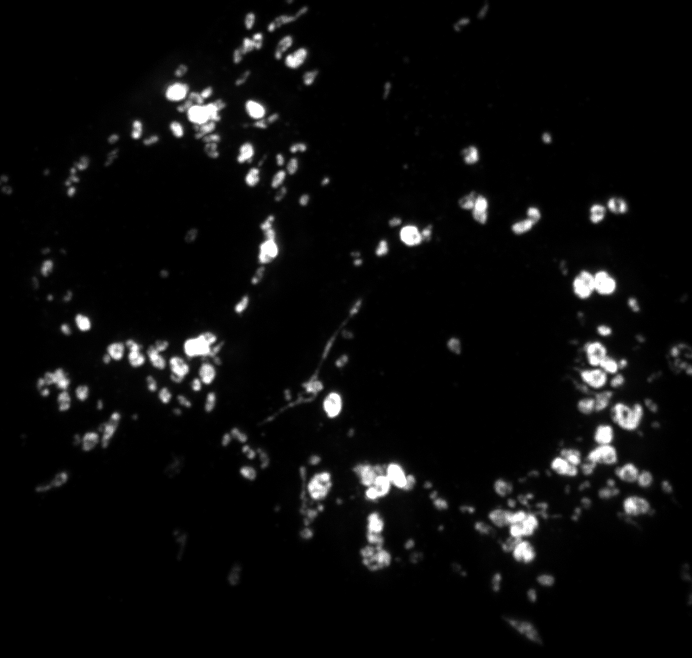

Supplement: Supplementary file 2 — Source Data Fig. 2 [file 44319_2023_9_MOESM2_ESM.zip › fig 1/d/IMAGES/MTCH2 KO/control/MAX_MEFS MTCH2 ko untaged mfn1 anti mfn1 gfp dapi52_thumb_w1Con-Cy5-1.tif (RGB).tif]

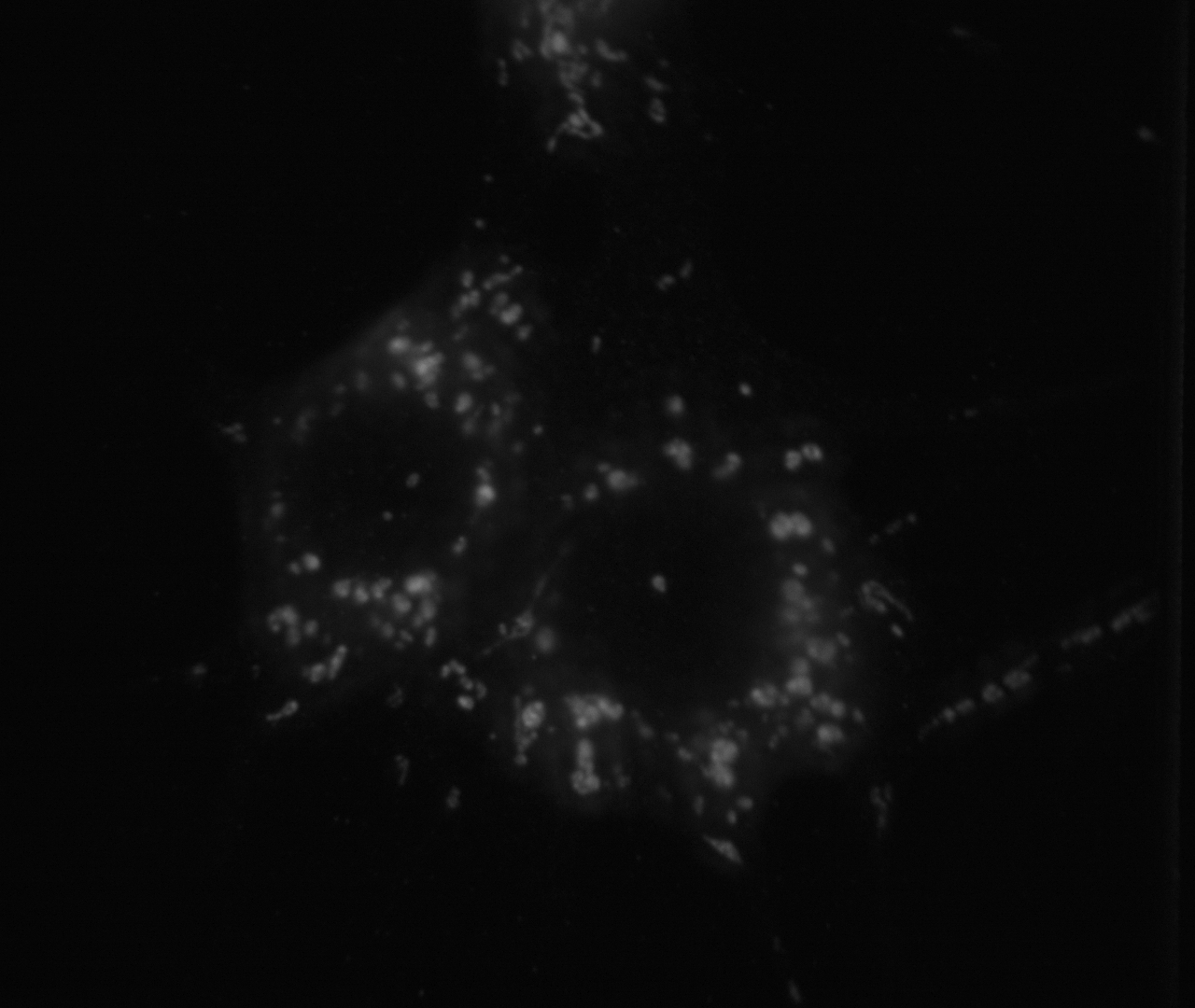

Supplement: Supplementary file 2 — Source Data Fig. 2 [file 44319_2023_9_MOESM2_ESM.zip › fig 1/d/IMAGES/MTCH2 KO/control/MEFS MTCH2 ko untaged mfn1 anti mfn1 gfp dapi52_thumb_w1Con-Cy5.tif]

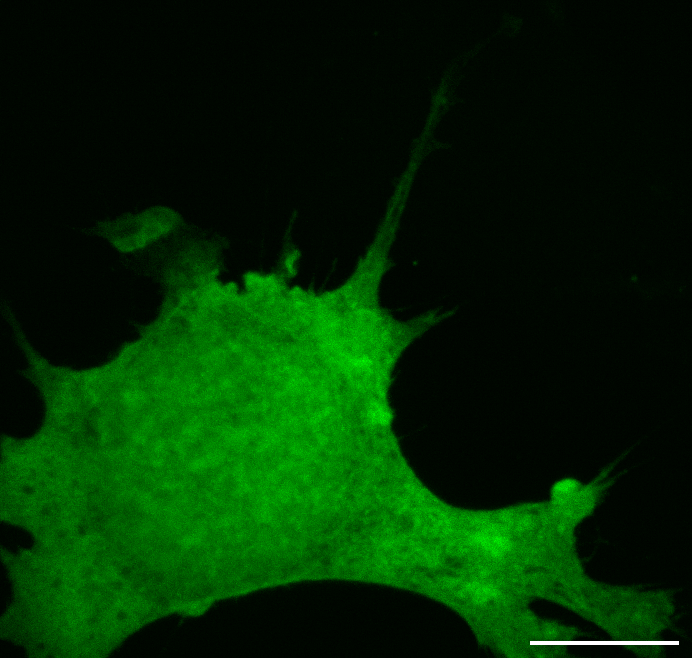

Supplement: Supplementary file 2 — Source Data Fig. 2 [file 44319_2023_9_MOESM2_ESM.zip › fig 1/d/IMAGES/MTCH2 KO/transfected/MAX_MEFS MTCH2 ko untaged mfn1 anti mfn1 gfp dapi48_thumb_w1Con-Cy5-1.tif (RGB) GREEN-1 scale bar.tif]

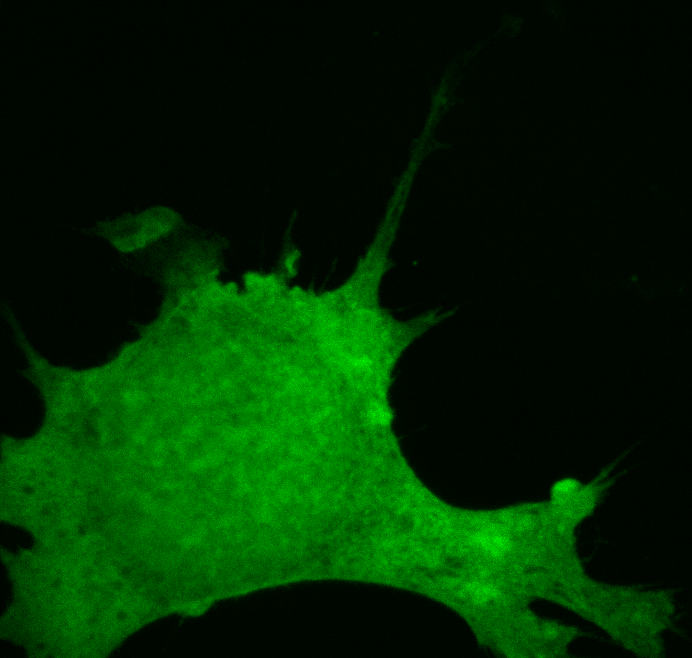

Supplement: Supplementary file 2 — Source Data Fig. 2 [file 44319_2023_9_MOESM2_ESM.zip › fig 1/d/IMAGES/MTCH2 KO/transfected/MAX_MEFS MTCH2 ko untaged mfn1 anti mfn1 gfp dapi48_thumb_w1Con-Cy5-1.tif (RGB) GREEN.tif]

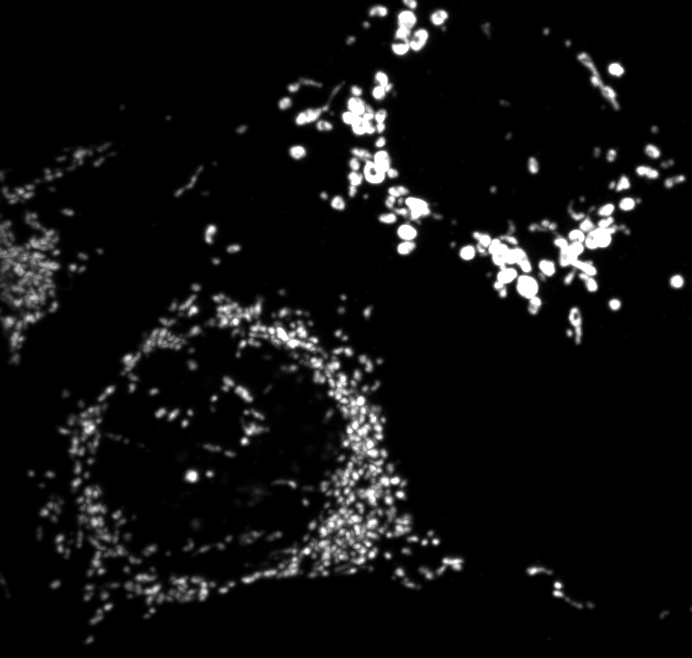

Supplement: Supplementary file 2 — Source Data Fig. 2 [file 44319_2023_9_MOESM2_ESM.zip › fig 1/d/IMAGES/MTCH2 KO/transfected/MAX_MEFS MTCH2 ko untaged mfn1 anti mfn1 gfp dapi48_thumb_w1Con-Cy5-1.tif (RGB) MITO.tif]

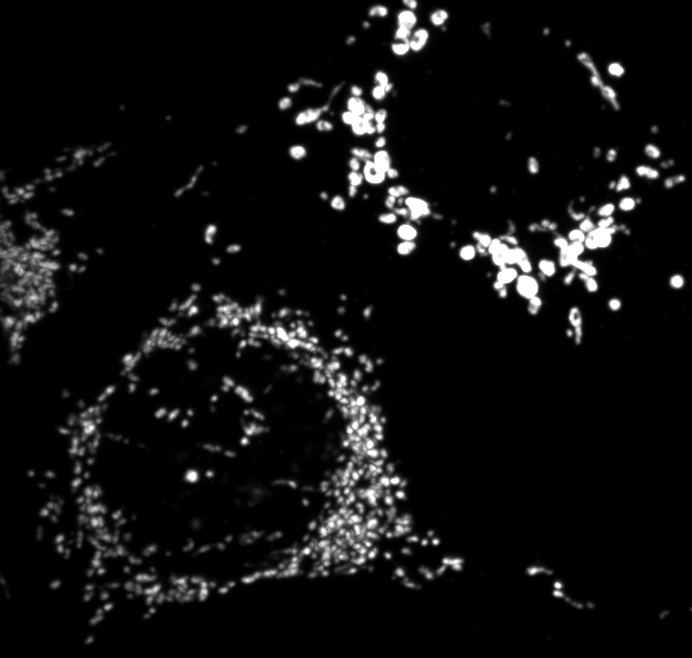

Supplement: Supplementary file 2 — Source Data Fig. 2 [file 44319_2023_9_MOESM2_ESM.zip › fig 1/d/IMAGES/MTCH2 KO/transfected/MAX_MEFS MTCH2 ko untaged mfn1 anti mfn1 gfp dapi48_thumb_w1Con-Cy5-1.tif (RGB).tif]

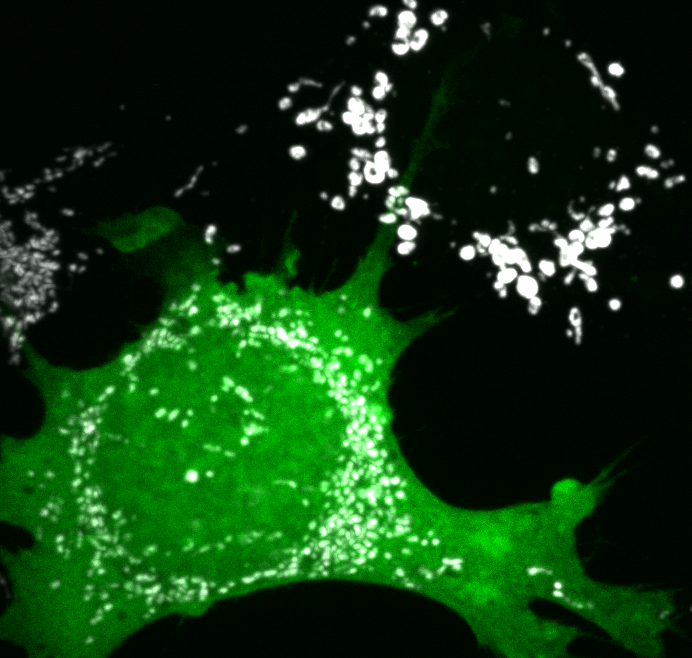

Supplement: Supplementary file 2 — Source Data Fig. 2 [file 44319_2023_9_MOESM2_ESM.zip › fig 1/d/IMAGES/MTCH2 KO/transfected/MAX_MEFS MTCH2 ko untaged mfn1 anti mfn1 gfp dapi48_thumb_w1Con-Cy5-1.tif (RGBCOM).tif]

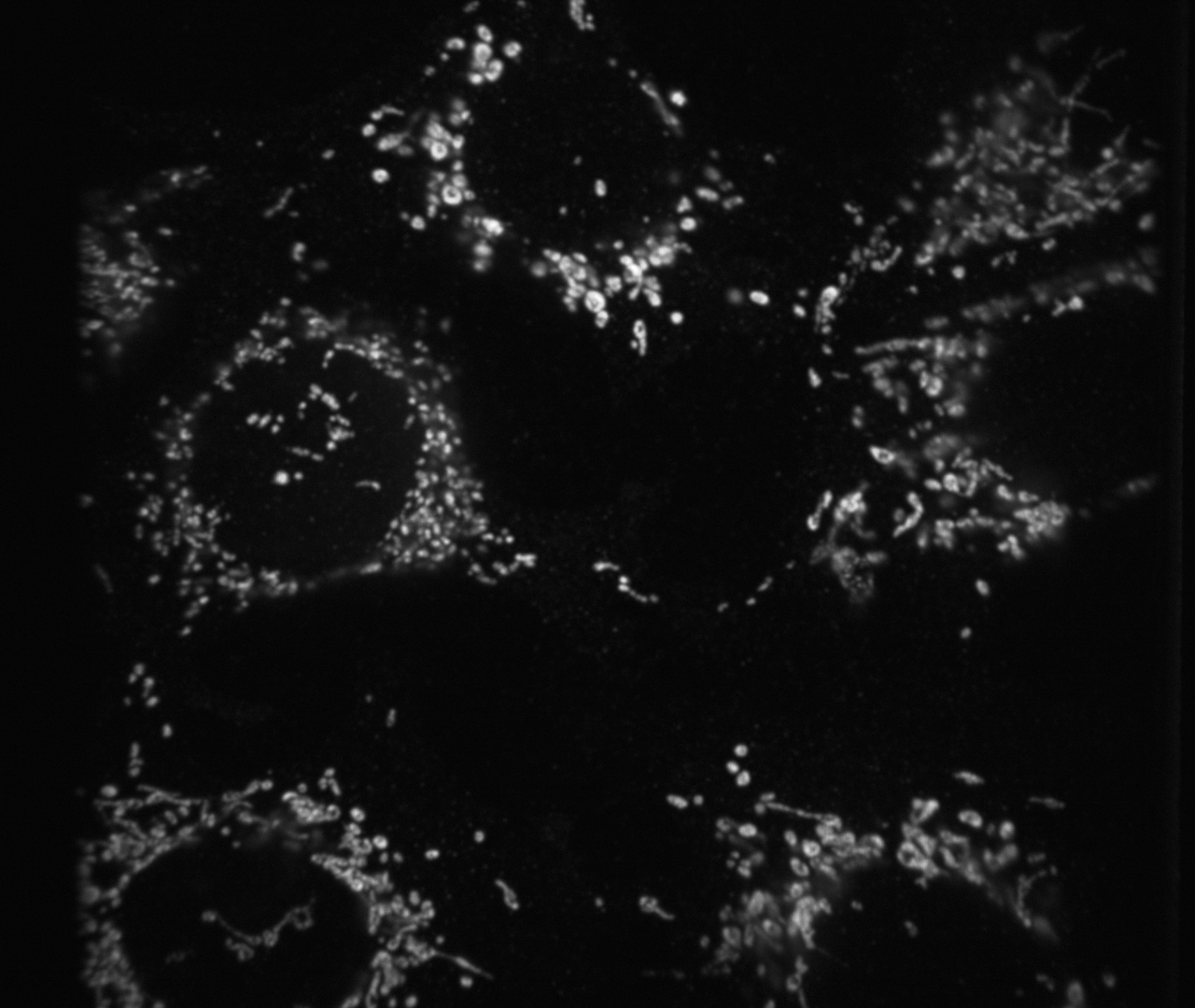

Supplement: Supplementary file 2 — Source Data Fig. 2 [file 44319_2023_9_MOESM2_ESM.zip › fig 1/d/IMAGES/MTCH2 KO/transfected/MEFS MTCH2 ko untaged mfn1 anti mfn1 gfp dapi48_thumb_w1Con-Cy5.tif]

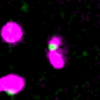

Supplement: Supplementary file 2 — Source Data Fig. 2 [file 44319_2023_9_MOESM2_ESM.zip › fig 1/g/images/MTCH2 KO/Process_17396.vsi - Cy5-Quad, GFP-Quad-1 example1-1 t10.tif]

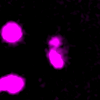

Supplement: Supplementary file 2 — Source Data Fig. 2 [file 44319_2023_9_MOESM2_ESM.zip › fig 1/g/images/MTCH2 KO/Process_17396.vsi - Cy5-Quad, GFP-Quad-1 example1-1 t104.tif]

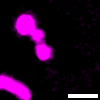

Supplement: Supplementary file 2 — Source Data Fig. 2 [file 44319_2023_9_MOESM2_ESM.zip › fig 1/g/images/MTCH2 KO/Process_17396.vsi - Cy5-Quad, GFP-Quad-1 example1-1 t135-1 2im scale bar.tif]

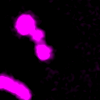

Supplement: Supplementary file 2 — Source Data Fig. 2 [file 44319_2023_9_MOESM2_ESM.zip › fig 1/g/images/MTCH2 KO/Process_17396.vsi - Cy5-Quad, GFP-Quad-1 example1-1 t135.tif]

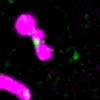

Supplement: Supplementary file 2 — Source Data Fig. 2 [file 44319_2023_9_MOESM2_ESM.zip › fig 1/g/images/MTCH2 KO/Process_17396.vsi - Cy5-Quad, GFP-Quad-1 example1-1 t135x.tif]

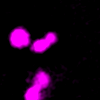

Supplement: Supplementary file 2 — Source Data Fig. 2 [file 44319_2023_9_MOESM2_ESM.zip › fig 1/g/images/MTCH2 KO/Process_17396.vsi - Cy5-Quad, GFP-Quad-1 example1-1 t71.tif]

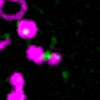

Supplement: Supplementary file 2 — Source Data Fig. 2 [file 44319_2023_9_MOESM2_ESM.zip › fig 1/g/images/MTCH2 KO/Process_17396.vsi - Cy5-Quad, GFP-Quad-1 example1-1t37.tif]

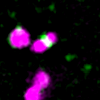

Supplement: Supplementary file 2 — Source Data Fig. 2 [file 44319_2023_9_MOESM2_ESM.zip › fig 1/g/images/MTCH2 KO/Process_17396.vsi - Cy5-Quad, GFP-Quad-1 example1-1t71c.tif]

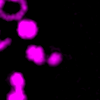

Supplement: Supplementary file 2 — Source Data Fig. 2 [file 44319_2023_9_MOESM2_ESM.zip › fig 1/g/images/MTCH2 KO/Process_17396.vsi - Cy5-Quad, GFP-Quad-1 example1-2 t37.tif]

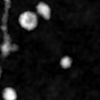

Supplement: Supplementary file 2 — Source Data Fig. 2 [file 44319_2023_9_MOESM2_ESM.zip › fig 1/g/images/MTCH2 KO/Process_17396.vsi - Cy5-Quad, GFP-Quad-1 example1.tif]

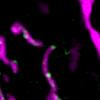

Supplement: Supplementary file 2 — Source Data Fig. 2 [file 44319_2023_9_MOESM2_ESM.zip › fig 1/g/images/WT/Process_5894.vsi - 011 .tiff-6 CROPP example1-1 t0.tif]

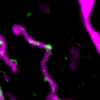

Supplement: Supplementary file 2 — Source Data Fig. 2 [file 44319_2023_9_MOESM2_ESM.zip › fig 1/g/images/WT/Process_5894.vsi - 011 .tiff-6 CROPP example1-1 t1.tif]

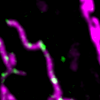

Supplement: Supplementary file 2 — Source Data Fig. 2 [file 44319_2023_9_MOESM2_ESM.zip › fig 1/g/images/WT/Process_5894.vsi - 011 .tiff-6 CROPP example1-1 t18.tif]

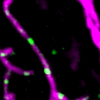

Supplement: Supplementary file 2 — Source Data Fig. 2 [file 44319_2023_9_MOESM2_ESM.zip › fig 1/g/images/WT/Process_5894.vsi - 011 .tiff-6 CROPP example1-1 t33.tif]

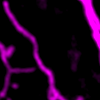

Supplement: Supplementary file 2 — Source Data Fig. 2 [file 44319_2023_9_MOESM2_ESM.zip › fig 1/g/images/WT/Process_5894.vsi - 011 .tiff-6 CROPP example1-1 t42.tif]

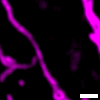

Supplement: Supplementary file 2 — Source Data Fig. 2 [file 44319_2023_9_MOESM2_ESM.zip › fig 1/g/images/WT/Process_5894.vsi - 011 .tiff-6 CROPP example1-1 t49-1 2um scale bar.tif]

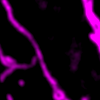

Supplement: Supplementary file 2 — Source Data Fig. 2 [file 44319_2023_9_MOESM2_ESM.zip › fig 1/g/images/WT/Process_5894.vsi - 011 .tiff-6 CROPP example1-1 t49.tif]

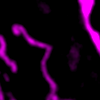

Supplement: Supplementary file 2 — Source Data Fig. 2 [file 44319_2023_9_MOESM2_ESM.zip › fig 1/g/images/WT/Process_5894.vsi - 011 .tiff-6 CROPP example1-2 t1.tif]

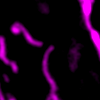

Supplement: Supplementary file 2 — Source Data Fig. 2 [file 44319_2023_9_MOESM2_ESM.zip › fig 1/g/images/WT/Process_5894.vsi - 011 .tiff-6 CROPP example1-2 t0.tif]

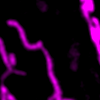

Supplement: Supplementary file 2 — Source Data Fig. 2 [file 44319_2023_9_MOESM2_ESM.zip › fig 1/g/images/WT/Process_5894.vsi - 011 .tiff-6 CROPP example1-2 t18.tif]

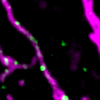

Supplement: Supplementary file 2 — Source Data Fig. 2 [file 44319_2023_9_MOESM2_ESM.zip › fig 1/g/images/WT/Process_5894.vsi - 011 .tiff-6 CROPP example1-2 t49.tif]

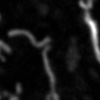

Supplement: Supplementary file 2 — Source Data Fig. 2 [file 44319_2023_9_MOESM2_ESM.zip › fig 1/g/images/WT/Process_5894.vsi - 011 .tiff-6 CROPP example1.tif]

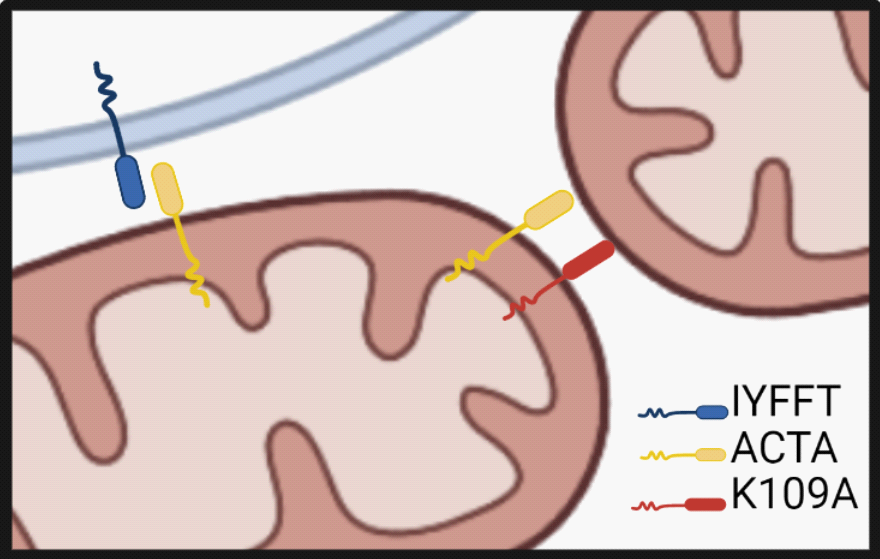

Supplement: Supplementary file 3 — Source Data Fig. 3 [file 44319_2023_9_MOESM3_ESM.zip › fig 2/a/mutants model.tif]

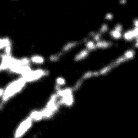

Supplement: Supplementary file 3 — Source Data Fig. 3 [file 44319_2023_9_MOESM3_ESM.zip › fig 2/b/MFN2 ACTA/Composite-1.tif (RGB) ch1.tif]

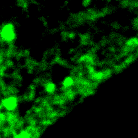

Supplement: Supplementary file 3 — Source Data Fig. 3 [file 44319_2023_9_MOESM3_ESM.zip › fig 2/b/MFN2 ACTA/Composite-1.tif (RGB) ch2.tif]

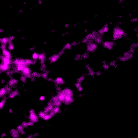

Supplement: Supplementary file 3 — Source Data Fig. 3 [file 44319_2023_9_MOESM3_ESM.zip › fig 2/b/MFN2 ACTA/Composite-1.tif (RGB) ch3.tif]

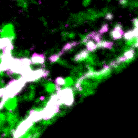

Supplement: Supplementary file 3 — Source Data Fig. 3 [file 44319_2023_9_MOESM3_ESM.zip › fig 2/b/MFN2 ACTA/Composite-1.tif (RGB) compo.tif]

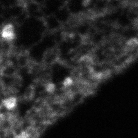

Supplement: Supplementary file 3 — Source Data Fig. 3 [file 44319_2023_9_MOESM3_ESM.zip › fig 2/b/MFN2 ACTA/MAX_MEFS MTCH2 ko acta flag598 er gfp mitobfp mrpl12 dapi59_thumb_w1Con-mcherry-1-1.tif]

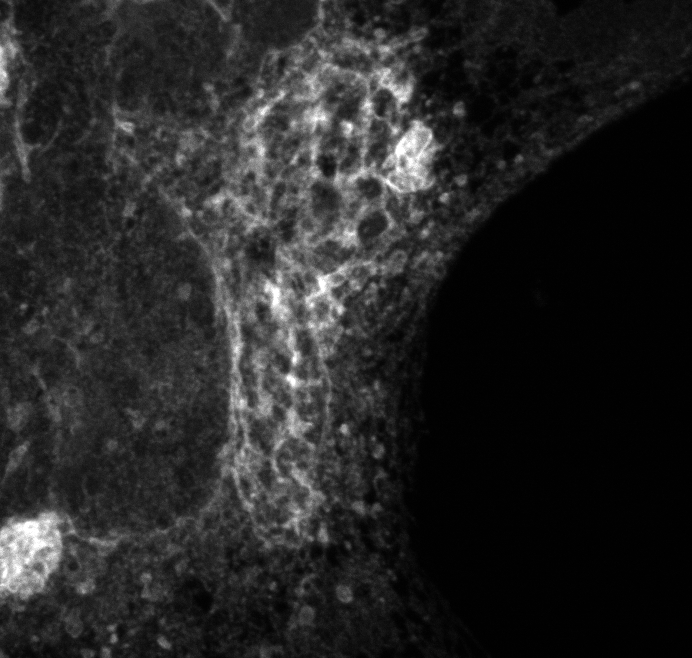

Supplement: Supplementary file 3 — Source Data Fig. 3 [file 44319_2023_9_MOESM3_ESM.zip › fig 2/b/MFN2 ACTA/MAX_MEFS MTCH2 ko acta flag598 er gfp mitobfp mrpl12 dapi59_thumb_w1Con-mcherry-1.tif]

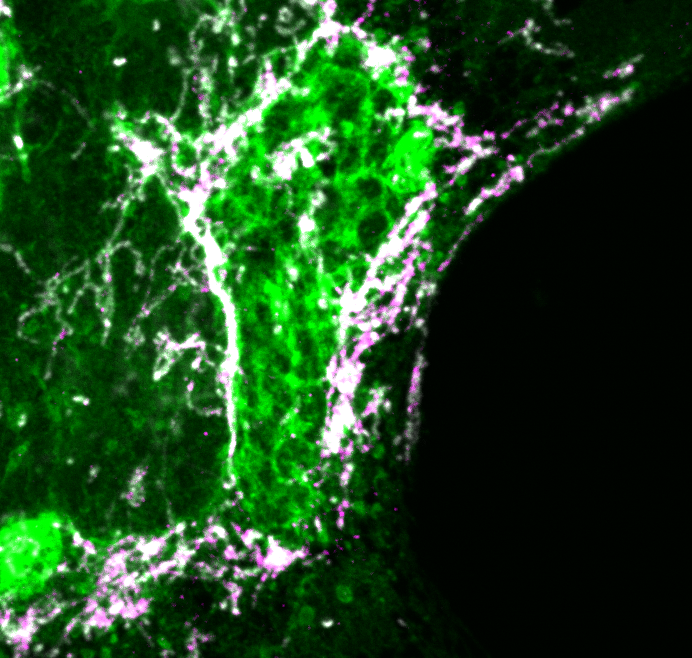

Supplement: Supplementary file 3 — Source Data Fig. 3 [file 44319_2023_9_MOESM3_ESM.zip › fig 2/b/MFN2 ACTA/MAX_MEFS MTCH2 ko acta flag598 er gfp mitobfp mrpl12 dapi59_thumb_w1Con-mcherry-1.tif (RGB).tif]

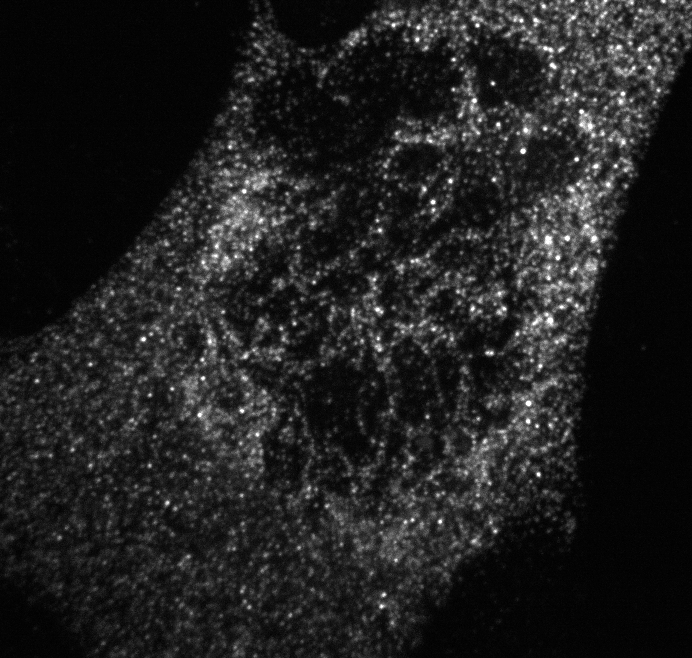

Supplement: Supplementary file 3 — Source Data Fig. 3 [file 44319_2023_9_MOESM3_ESM.zip › fig 2/b/MFN2 IYFFT/MAX_MEFS MTCH2 KO MFN2 IYFFT FLAG 598 ER GFP TOM640 DAPI15_thumb_w1Con-mcherry-1.tif]

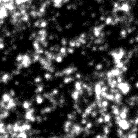

Supplement: Supplementary file 3 — Source Data Fig. 3 [file 44319_2023_9_MOESM3_ESM.zip › fig 2/b/MFN2 IYFFT/MAX_MEFS MTCH2 KO MFN2 IYFFT FLAG 598 ER GFP TOM640 DAPI15_thumb_w1Con-mcherry-13-1-1-3-1 for line fluorescence.tif]

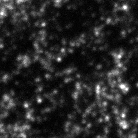

Supplement: Supplementary file 3 — Source Data Fig. 3 [file 44319_2023_9_MOESM3_ESM.zip › fig 2/b/MFN2 IYFFT/MAX_MEFS MTCH2 KO MFN2 IYFFT FLAG 598 ER GFP TOM640 DAPI15_thumb_w1Con-mcherry-13-1-1-3.tif]

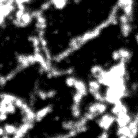

Supplement: Supplementary file 3 — Source Data Fig. 3 [file 44319_2023_9_MOESM3_ESM.zip › fig 2/b/MFN2 IYFFT/MAX_MEFS MTCH2 KO MFN2 IYFFT FLAG 598 ER GFP TOM640 DAPI15_thumb_w1Con-mcherry-13-1-1-3.tif (RGB) ch1.tif]

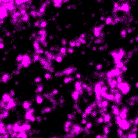

Supplement: Supplementary file 3 — Source Data Fig. 3 [file 44319_2023_9_MOESM3_ESM.zip › fig 2/b/MFN2 IYFFT/MAX_MEFS MTCH2 KO MFN2 IYFFT FLAG 598 ER GFP TOM640 DAPI15_thumb_w1Con-mcherry-13-1-1-3.tif (RGB) ch2.tif]

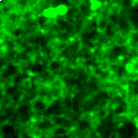

Supplement: Supplementary file 3 — Source Data Fig. 3 [file 44319_2023_9_MOESM3_ESM.zip › fig 2/b/MFN2 IYFFT/MAX_MEFS MTCH2 KO MFN2 IYFFT FLAG 598 ER GFP TOM640 DAPI15_thumb_w1Con-mcherry-13-1-1-3.tif (RGB) ch3.tif]

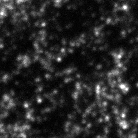

Supplement: Supplementary file 3 — Source Data Fig. 3 [file 44319_2023_9_MOESM3_ESM.zip › fig 2/b/MFN2 IYFFT/MAX_MEFS MTCH2 KO MFN2 IYFFT FLAG 598 ER GFP TOM640 DAPI15_thumb_w1Con-mcherry-13-1-1.tif]

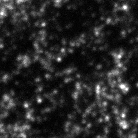

Supplement: Supplementary file 3 — Source Data Fig. 3 [file 44319_2023_9_MOESM3_ESM.zip › fig 2/b/MFN2 IYFFT/MAX_MEFS MTCH2 KO MFN2 IYFFT FLAG 598 ER GFP TOM640 DAPI15_thumb_w1Con-mcherry-13-1.tif]

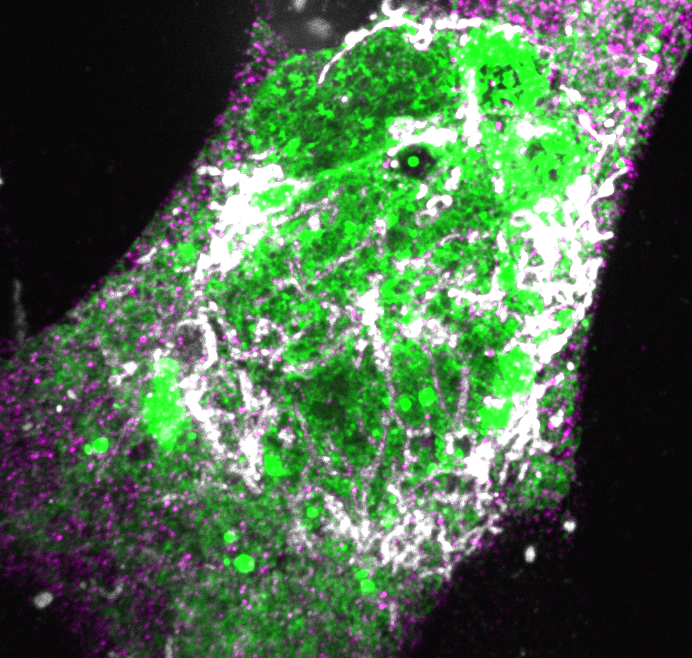

Supplement: Supplementary file 3 — Source Data Fig. 3 [file 44319_2023_9_MOESM3_ESM.zip › fig 2/b/MFN2 IYFFT/MAX_MEFS MTCH2 KO MFN2 IYFFT FLAG 598 ER GFP TOM640 DAPI15_thumb_w1Con-mcherry-13.tif (RGB).tif]

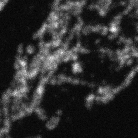

Supplement: Supplementary file 3 — Source Data Fig. 3 [file 44319_2023_9_MOESM3_ESM.zip › fig 2/b/MFN2 K209A/MAX_MEFs MTCH2 KO mfn2 k209a flag tom633 dapi3_thumb_w1Con-mcherry-1-1-1.tif]

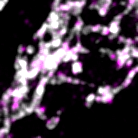

Supplement: Supplementary file 3 — Source Data Fig. 3 [file 44319_2023_9_MOESM3_ESM.zip › fig 2/b/MFN2 K209A/MAX_MEFs MTCH2 KO mfn2 k209a flag tom633 dapi3_thumb_w1Con-mcherry-1-1-1.tif (RGB) new ch1.tif]

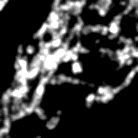

Supplement: Supplementary file 3 — Source Data Fig. 3 [file 44319_2023_9_MOESM3_ESM.zip › fig 2/b/MFN2 K209A/MAX_MEFs MTCH2 KO mfn2 k209a flag tom633 dapi3_thumb_w1Con-mcherry-1-1-1.tif (RGB) new ch2.tif]

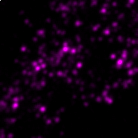

Supplement: Supplementary file 3 — Source Data Fig. 3 [file 44319_2023_9_MOESM3_ESM.zip › fig 2/b/MFN2 K209A/MAX_MEFs MTCH2 KO mfn2 k209a flag tom633 dapi3_thumb_w1Con-mcherry-1-1-1.tif (RGB) new ch3.tif]

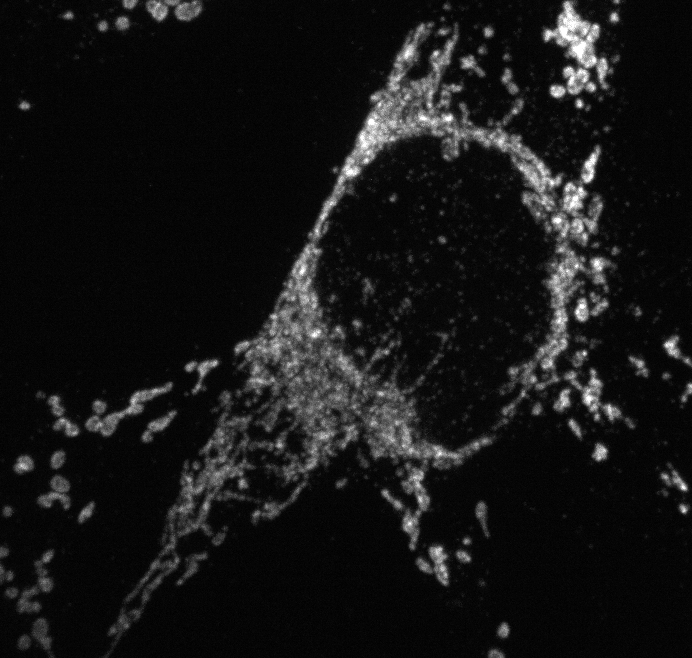

Supplement: Supplementary file 3 — Source Data Fig. 3 [file 44319_2023_9_MOESM3_ESM.zip › fig 2/b/MFN2 K209A/MAX_MEFs MTCH2 KO mfn2 k209a flag tom633 dapi3_thumb_w1Con-mcherry-1.tif]

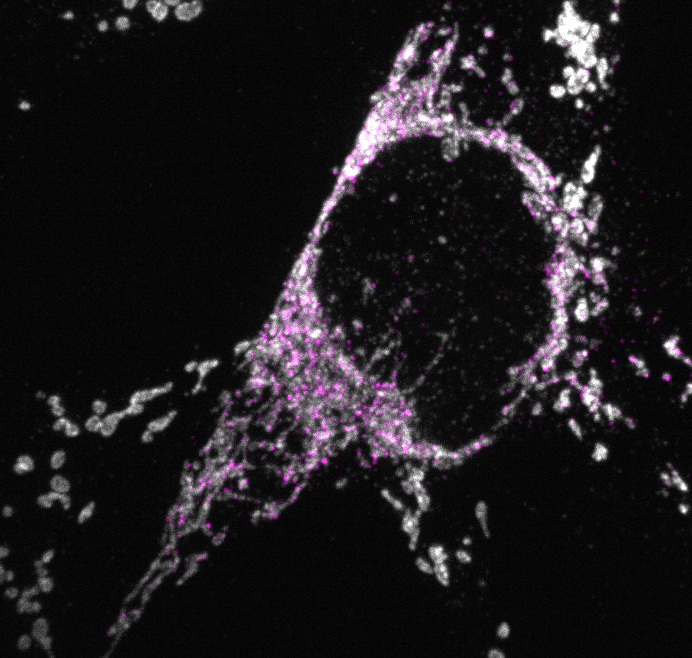

Supplement: Supplementary file 3 — Source Data Fig. 3 [file 44319_2023_9_MOESM3_ESM.zip › fig 2/b/MFN2 K209A/MAX_MEFs MTCH2 KO mfn2 k209a flag tom633 dapi3_thumb_w1Con-mcherry-1.tif (RGB) composite.tif]

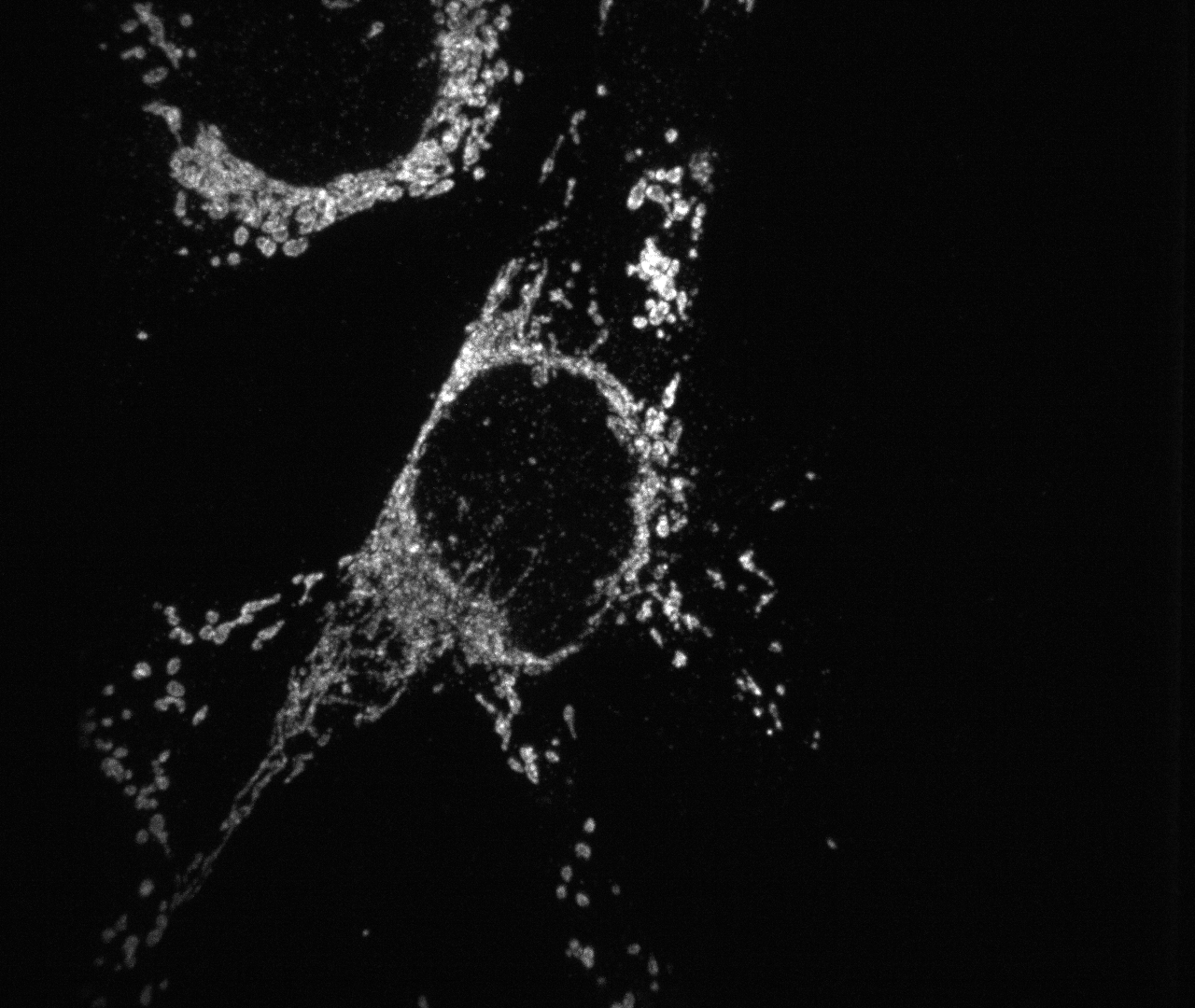

Supplement: Supplementary file 3 — Source Data Fig. 3 [file 44319_2023_9_MOESM3_ESM.zip › fig 2/b/MFN2 K209A/MAX_MEFs MTCH2 KO mfn2 k209a flag tom633 dapi3_thumb_w1Con-mcherry.tif]

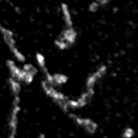

Supplement: Supplementary file 3 — Source Data Fig. 3 [file 44319_2023_9_MOESM3_ESM.zip › fig 2/b/MFN2 WT/MAX_Process_15802.vsi - GFP-Quad, mCherry-Quad-1-1.tif]

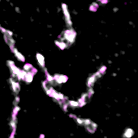

Supplement: Supplementary file 3 — Source Data Fig. 3 [file 44319_2023_9_MOESM3_ESM.zip › fig 2/b/MFN2 WT/MAX_Process_15802.vsi - GFP-Quad, mCherry-Quad-1-1.tif (RGB) n1.tif]

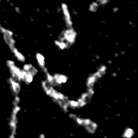

Supplement: Supplementary file 3 — Source Data Fig. 3 [file 44319_2023_9_MOESM3_ESM.zip › fig 2/b/MFN2 WT/MAX_Process_15802.vsi - GFP-Quad, mCherry-Quad-1-1.tif (RGB)n2.tif]

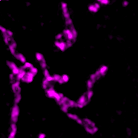

Supplement: Supplementary file 3 — Source Data Fig. 3 [file 44319_2023_9_MOESM3_ESM.zip › fig 2/b/MFN2 WT/MAX_Process_15802.vsi - GFP-Quad, mCherry-Quad-1-1.tif (RGB)op2.tif]

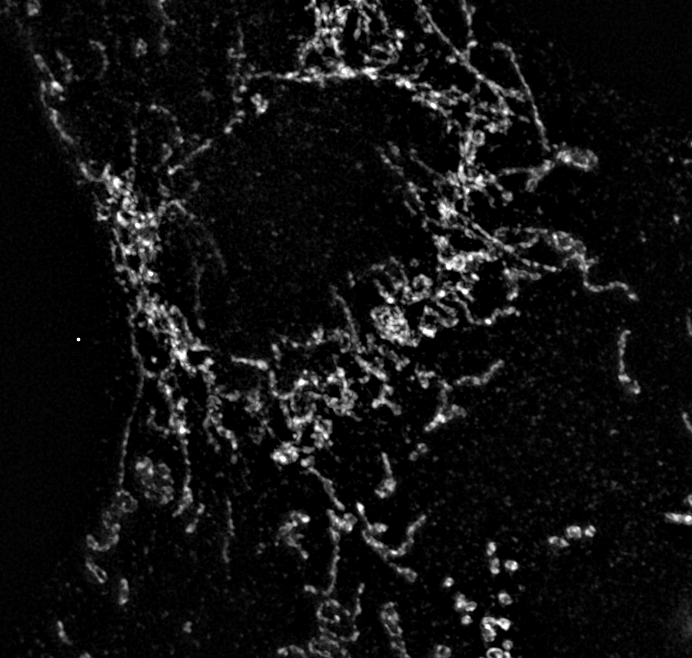

Supplement: Supplementary file 3 — Source Data Fig. 3 [file 44319_2023_9_MOESM3_ESM.zip › fig 2/b/MFN2 WT/MAX_Process_15802.vsi - GFP-Quad, mCherry-Quad-1.tif]

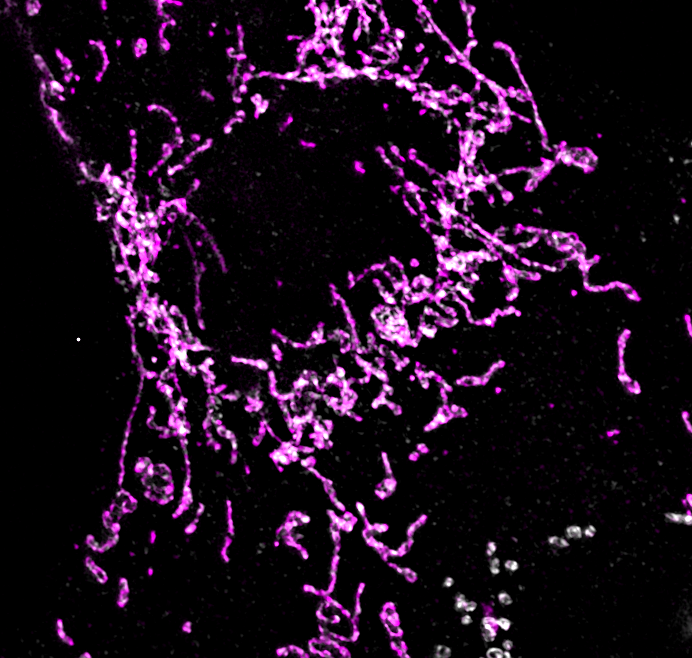

Supplement: Supplementary file 3 — Source Data Fig. 3 [file 44319_2023_9_MOESM3_ESM.zip › fig 2/b/MFN2 WT/MAX_Process_15802.vsi - GFP-Quad, mCherry-Quad-1.tif (RGB).tif]
