# Supplementary figures and images for: MTCH2 cooperates with MFN2 and lysophosphatidic acid synthesis to sustain mitochondrial fusion (part 2 of 6)
Source: EMBO Rep. 2023 Dec 14;25(1):8. doi: 10.1038/s44319-023-00009-1 (PMC10897490; doi:10.1038/s44319-023-00009-1)

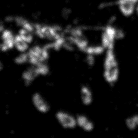

Supplement: Supplementary file 3 — Source Data Fig. 3 [file 44319_2023_9_MOESM3_ESM.zip › fig 2/b/mtch2 ko control/MAX_MEFs MTCH2 KO NT si CONTROL TOM40598 CYT633 DAPI2_thumb_w1Con-mcherry_s1.TIF - Stage20-1-1-1.tif]

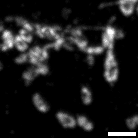

Supplement: Supplementary file 3 — Source Data Fig. 3 [file 44319_2023_9_MOESM3_ESM.zip › fig 2/b/mtch2 ko control/MAX_MEFs MTCH2 KO NT si CONTROL TOM40598 CYT633 DAPI2_thumb_w1Con-mcherry_s1.TIF - Stage20-1-1-2 scale.tif]

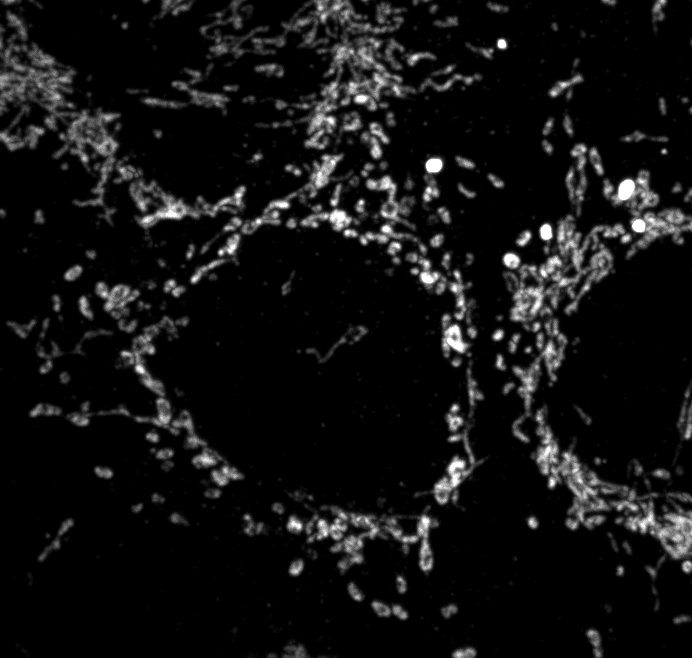

Supplement: Supplementary file 3 — Source Data Fig. 3 [file 44319_2023_9_MOESM3_ESM.zip › fig 2/b/mtch2 ko control/MAX_MEFs MTCH2 KO NT si CONTROL TOM40598 CYT633 DAPI2_thumb_w1Con-mcherry_s1.TIF - Stage20-1-1.tif]

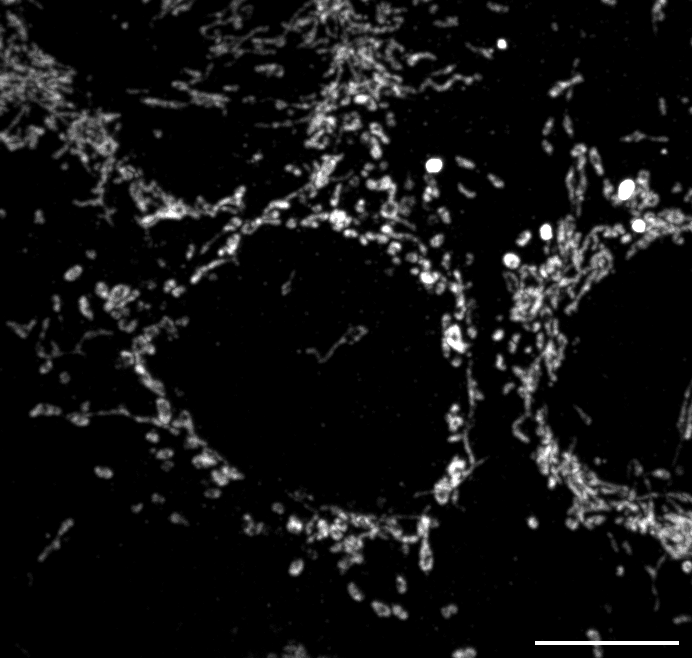

Supplement: Supplementary file 3 — Source Data Fig. 3 [file 44319_2023_9_MOESM3_ESM.zip › fig 2/b/mtch2 ko control/MAX_MEFs MTCH2 KO NT si CONTROL TOM40598 CYT633 DAPI2_thumb_w1Con-mcherry_s1.TIF - Stage20-1-2 scale bar.tif]

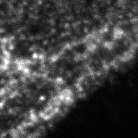

Supplement: Supplementary file 3 — Source Data Fig. 3 [file 44319_2023_9_MOESM3_ESM.zip › fig 2/c/ACTA/Composite-1.tif]

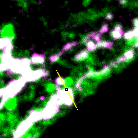

Supplement: Supplementary file 3 — Source Data Fig. 3 [file 44319_2023_9_MOESM3_ESM.zip › fig 2/c/ACTA/Composite-2 compo line plot.tif]

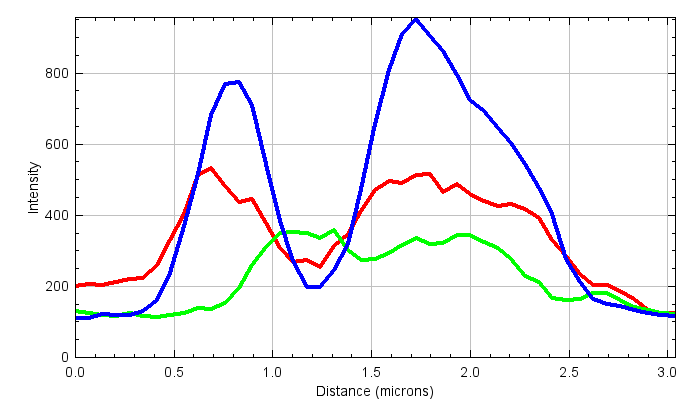

Supplement: Supplementary file 3 — Source Data Fig. 3 [file 44319_2023_9_MOESM3_ESM.zip › fig 2/c/ACTA/line plot acta.tif]

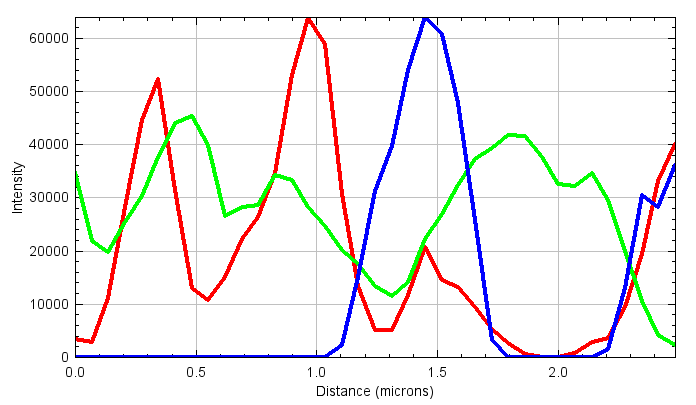

Supplement: Supplementary file 3 — Source Data Fig. 3 [file 44319_2023_9_MOESM3_ESM.zip › fig 2/c/IYFFT/line plot iyfft.tif]

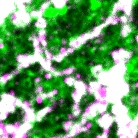

Supplement: Supplementary file 3 — Source Data Fig. 3 [file 44319_2023_9_MOESM3_ESM.zip › fig 2/c/IYFFT/MAX_MEFS MTCH2 KO MFN2 IYFFT FLAG 598 ER GFP TOM640 DAPI15_thumb_w1Con-mcherry-13-1-1-3.tif (RGB) comp.tif]

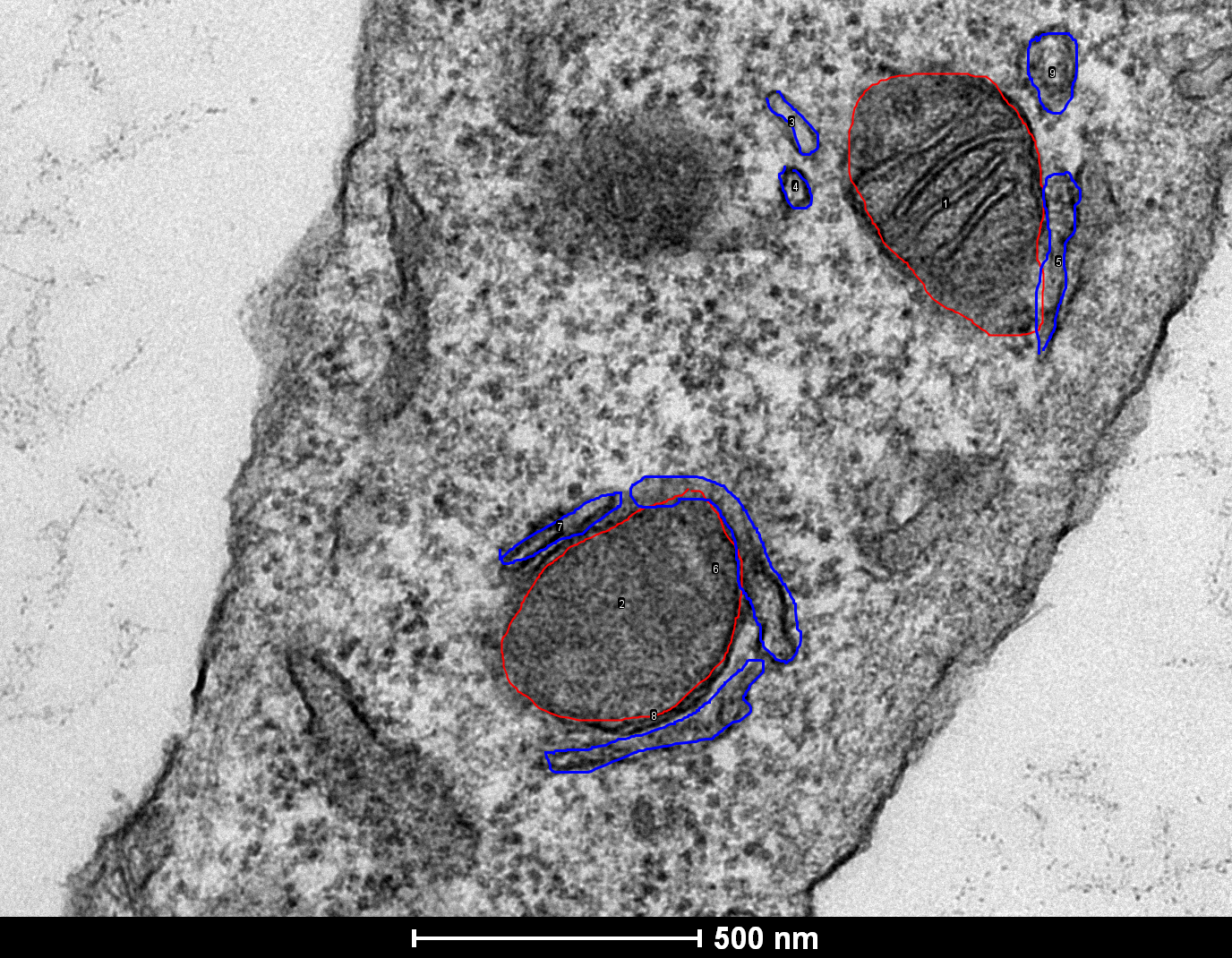

Supplement: Supplementary file 3 — Source Data Fig. 3 [file 44319_2023_9_MOESM3_ESM.zip › fig 2/f/images/Mtch2 KO/flaten_Q2 03b .tif]

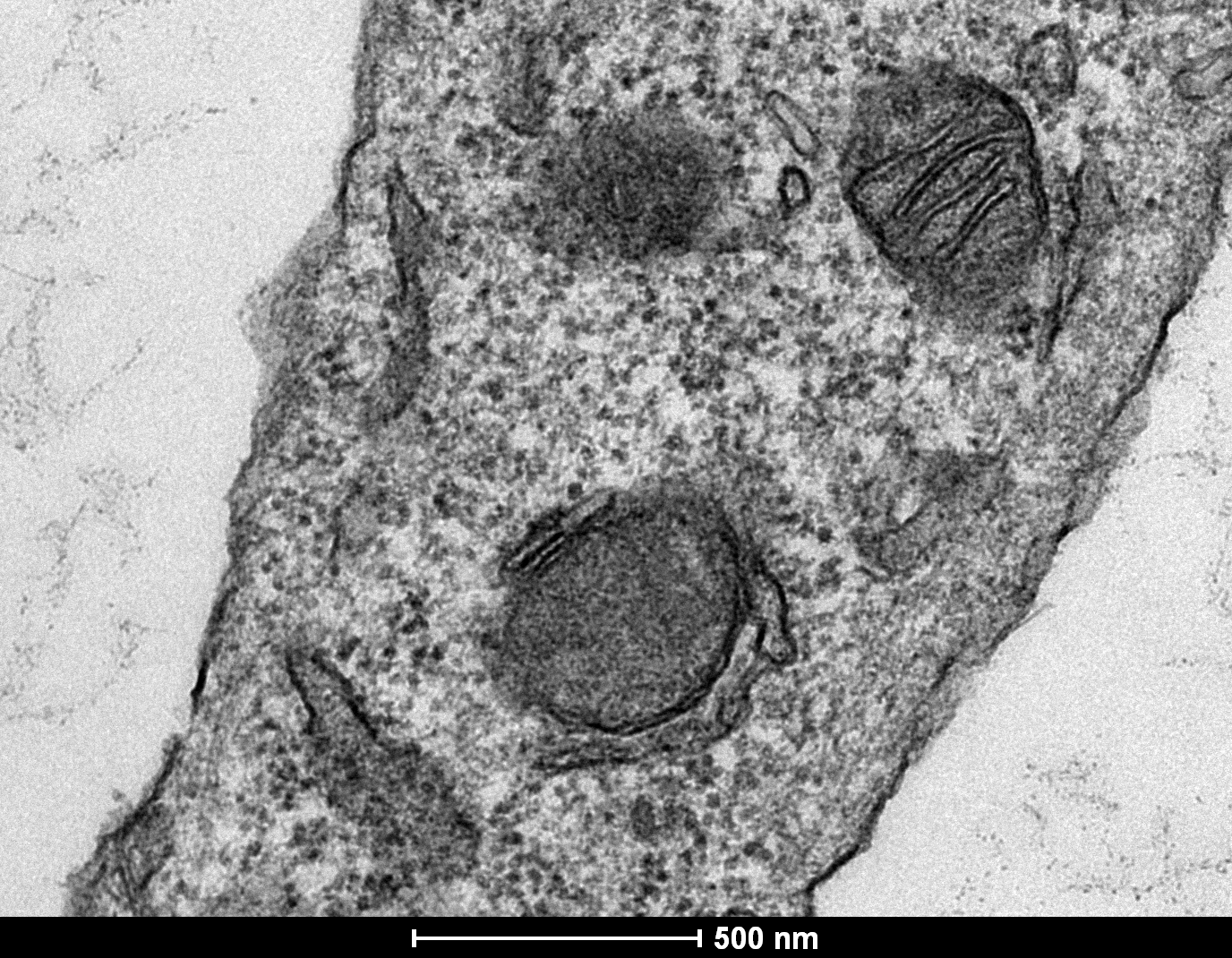

Supplement: Supplementary file 3 — Source Data Fig. 3 [file 44319_2023_9_MOESM3_ESM.zip › fig 2/f/images/Mtch2 KO/Q2 03b .tif]

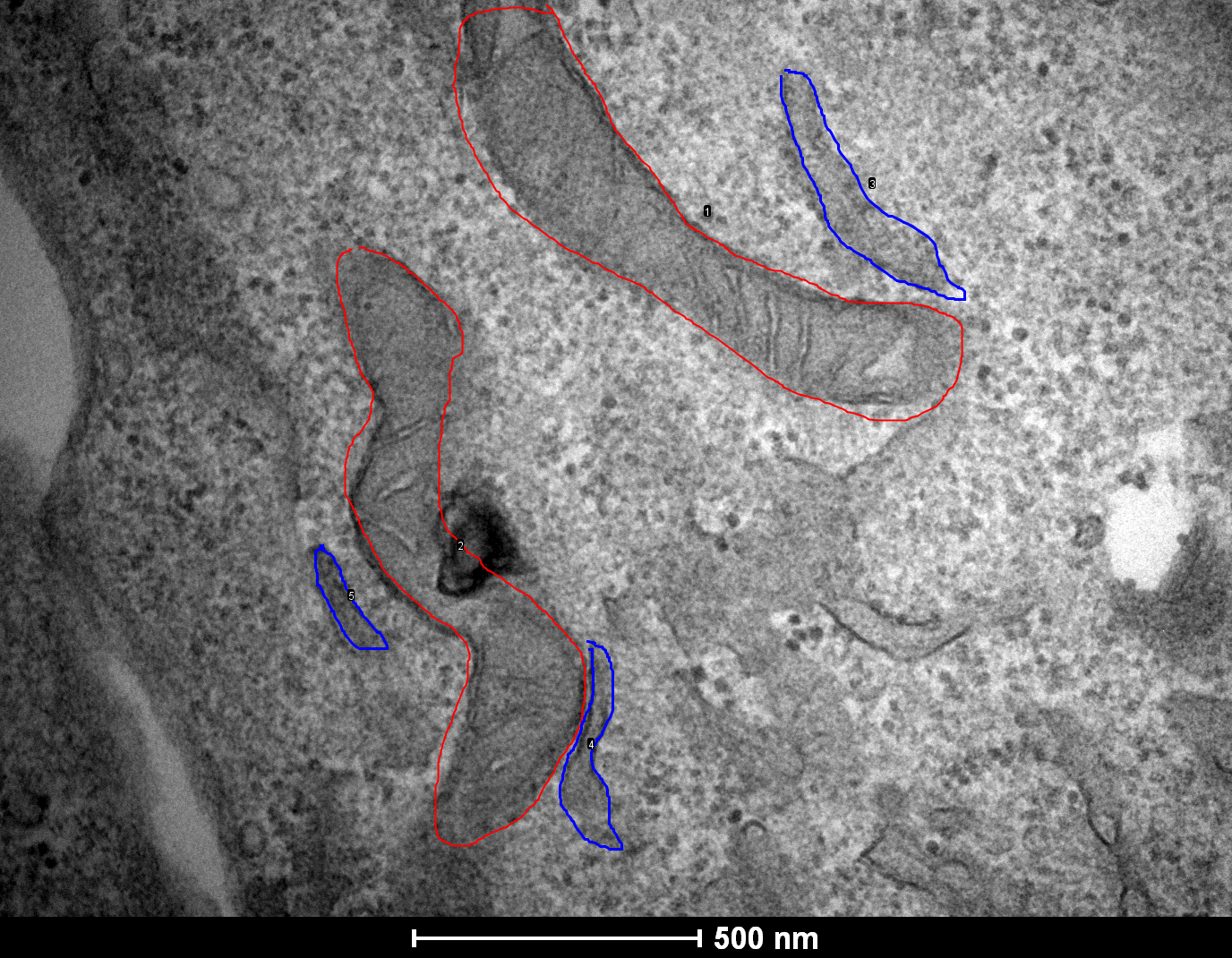

Supplement: Supplementary file 3 — Source Data Fig. 3 [file 44319_2023_9_MOESM3_ESM.zip › fig 2/f/images/WT/flaten_M2 06b .tif]

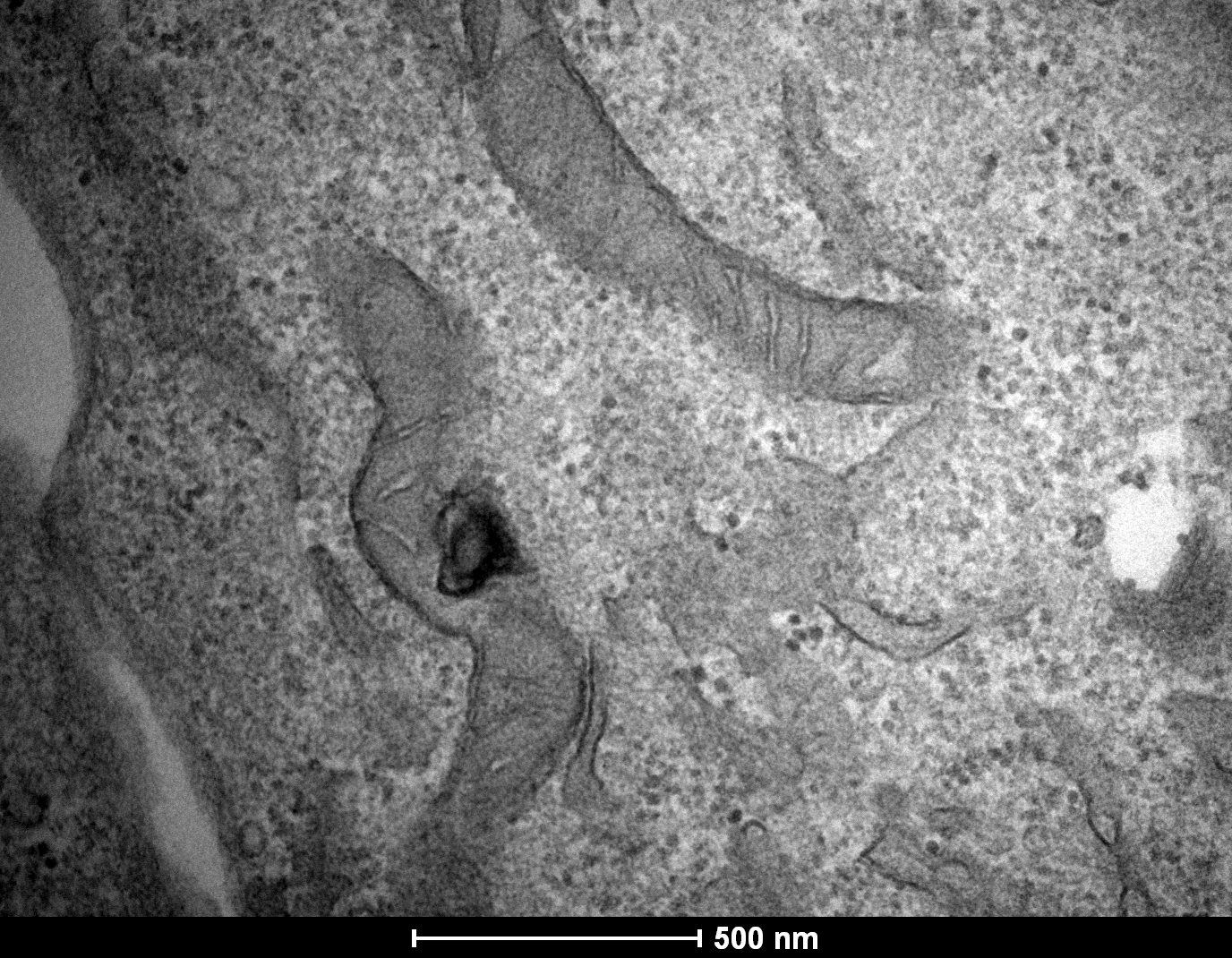

Supplement: Supplementary file 3 — Source Data Fig. 3 [file 44319_2023_9_MOESM3_ESM.zip › fig 2/f/images/WT/M2 06b .tif]

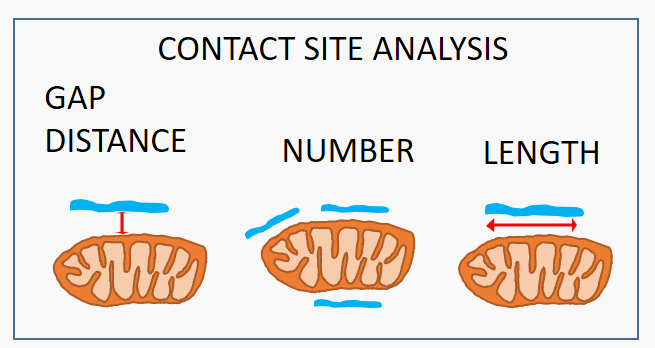

Supplement: Supplementary file 3 — Source Data Fig. 3 [file 44319_2023_9_MOESM3_ESM.zip › fig 2/g/contact site analysis.tif]

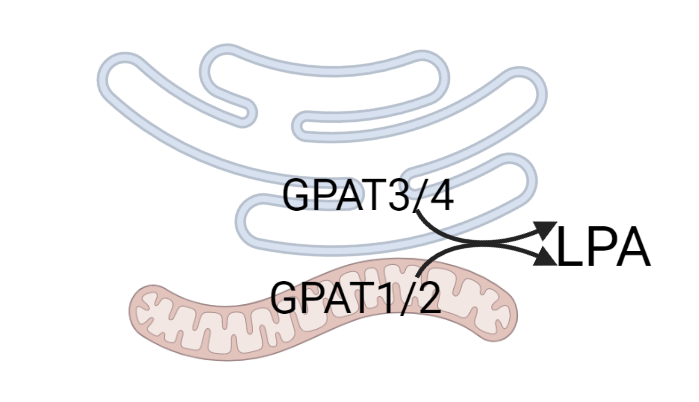

Supplement: Supplementary file 4 — Source Data Fig. 4 [file 44319_2023_9_MOESM4_ESM.zip › fig 3/a/gpats model.tif]

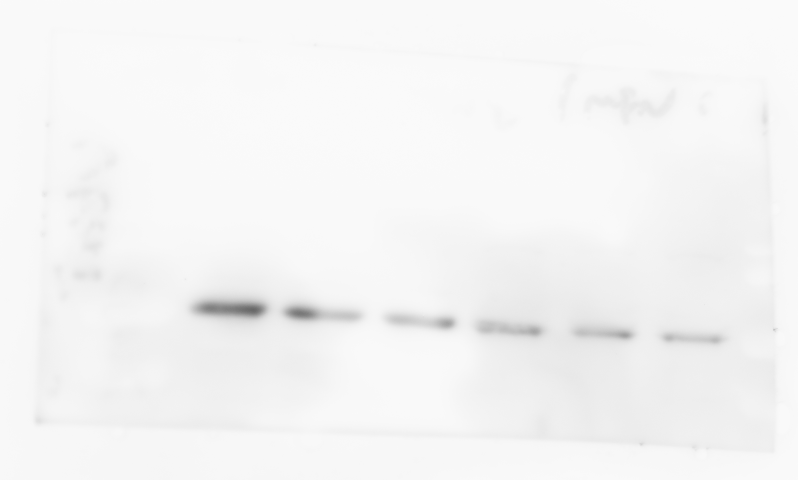

Supplement: Supplementary file 4 — Source Data Fig. 4 [file 44319_2023_9_MOESM4_ESM.zip › fig 3/b/BLOTS/CALNEXIN/CALNEXIN BLOT.tif]

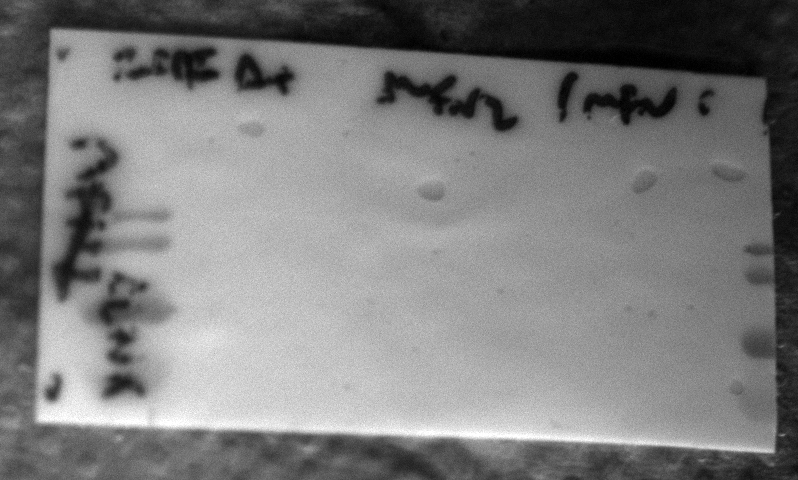

Supplement: Supplementary file 4 — Source Data Fig. 4 [file 44319_2023_9_MOESM4_ESM.zip › fig 3/b/BLOTS/CALNEXIN/CALNEXIN MEMBRANE.tif]

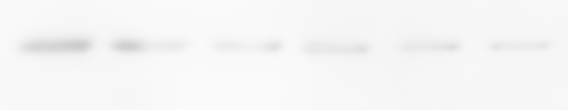

Supplement: Supplementary file 4 — Source Data Fig. 4 [file 44319_2023_9_MOESM4_ESM.zip › fig 3/b/BLOTS/CALNEXIN/clnx extra.tif]

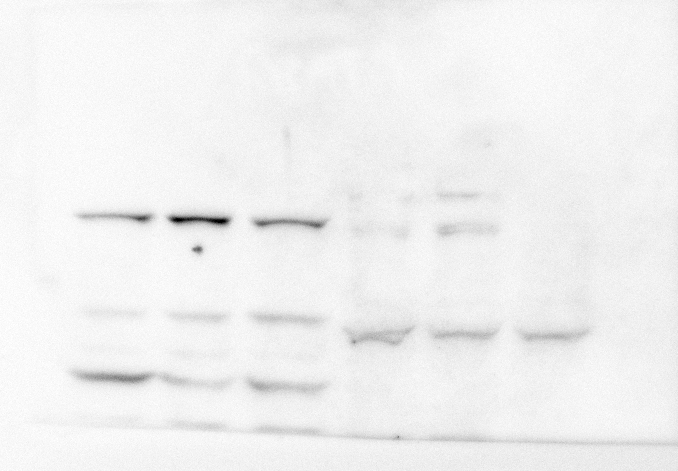

Supplement: Supplementary file 4 — Source Data Fig. 4 [file 44319_2023_9_MOESM4_ESM.zip › fig 3/b/BLOTS/GPAT1/GPAT1 BLOT.tif]

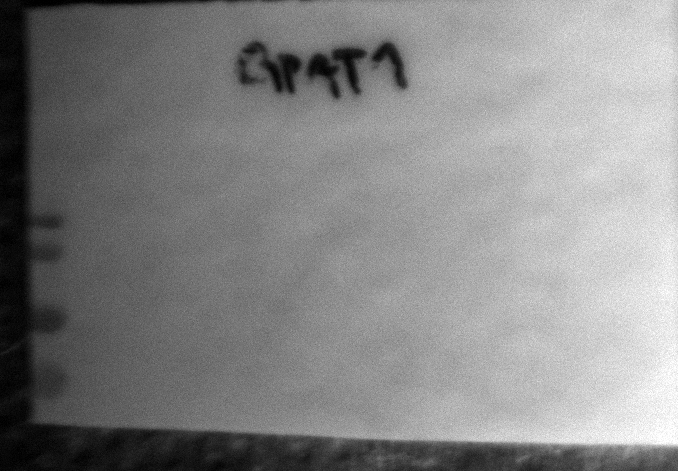

Supplement: Supplementary file 4 — Source Data Fig. 4 [file 44319_2023_9_MOESM4_ESM.zip › fig 3/b/BLOTS/GPAT1/GPAT1 MEMBRANE.tif]

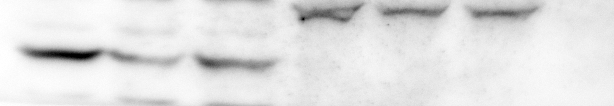

Supplement: Supplementary file 4 — Source Data Fig. 4 [file 44319_2023_9_MOESM4_ESM.zip › fig 3/b/BLOTS/GPAT1/GPAT1.tif]

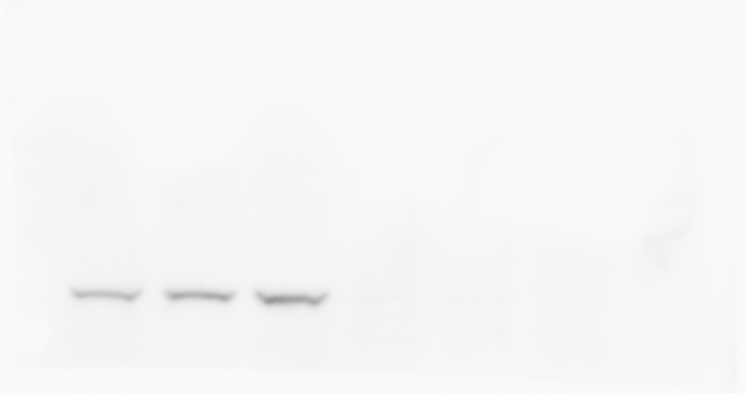

Supplement: Supplementary file 4 — Source Data Fig. 4 [file 44319_2023_9_MOESM4_ESM.zip › fig 3/b/BLOTS/GPAT2/GPAT2 BLOT.tif]

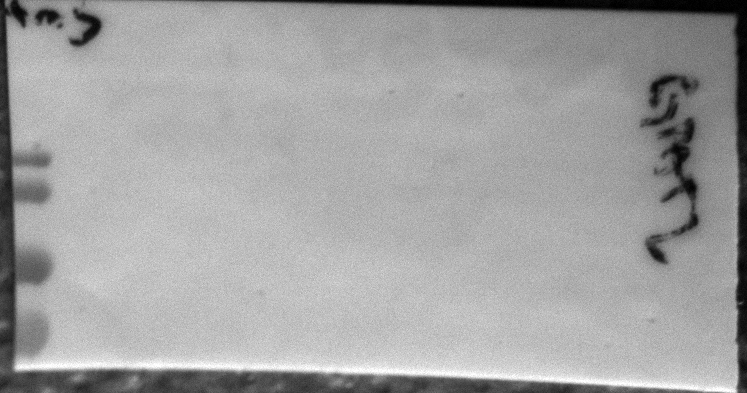

Supplement: Supplementary file 4 — Source Data Fig. 4 [file 44319_2023_9_MOESM4_ESM.zip › fig 3/b/BLOTS/GPAT2/GPAT2 MEMB.tif]

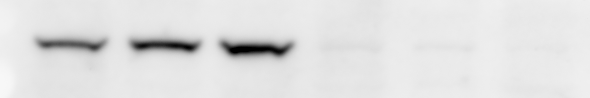

Supplement: Supplementary file 4 — Source Data Fig. 4 [file 44319_2023_9_MOESM4_ESM.zip › fig 3/b/BLOTS/GPAT2/gpat2.tif]

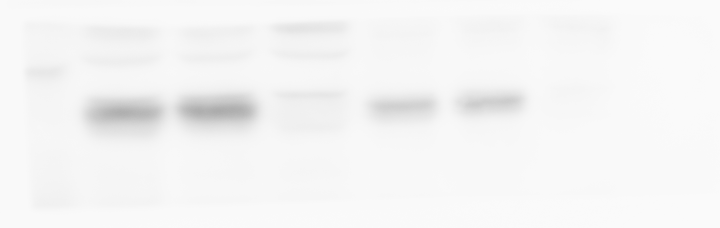

Supplement: Supplementary file 4 — Source Data Fig. 4 [file 44319_2023_9_MOESM4_ESM.zip › fig 3/b/BLOTS/GPAT3/GPAT3 BLOT.tif]

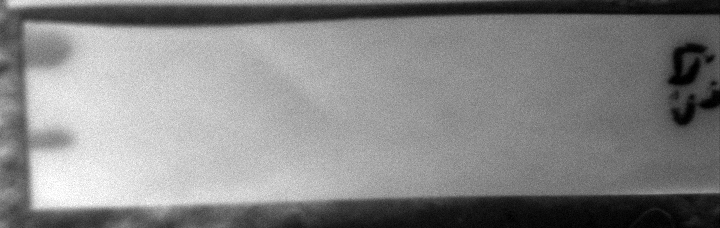

Supplement: Supplementary file 4 — Source Data Fig. 4 [file 44319_2023_9_MOESM4_ESM.zip › fig 3/b/BLOTS/GPAT3/GPAT3 MEMBRANE.tif]

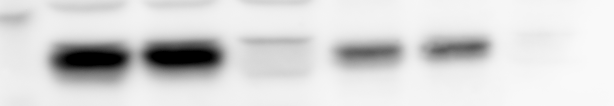

Supplement: Supplementary file 4 — Source Data Fig. 4 [file 44319_2023_9_MOESM4_ESM.zip › fig 3/b/BLOTS/GPAT3/GPAT3.tif]

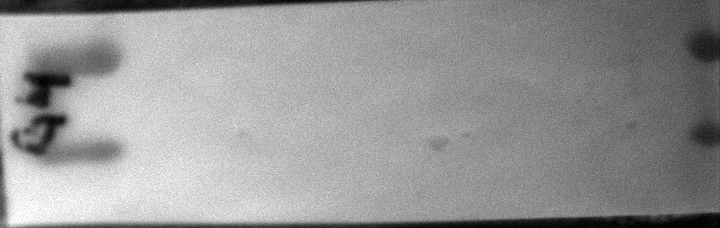

Supplement: Supplementary file 4 — Source Data Fig. 4 [file 44319_2023_9_MOESM4_ESM.zip › fig 3/b/BLOTS/GPAT4/GPAT4 MEMBRANE.tif]

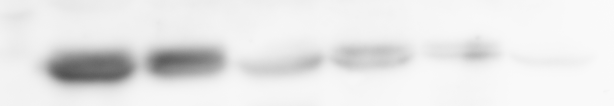

Supplement: Supplementary file 4 — Source Data Fig. 4 [file 44319_2023_9_MOESM4_ESM.zip › fig 3/b/BLOTS/GPAT4/GPAT4.tif]

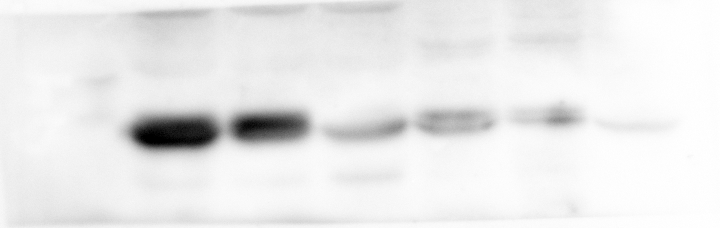

Supplement: Supplementary file 4 — Source Data Fig. 4 [file 44319_2023_9_MOESM4_ESM.zip › fig 3/b/BLOTS/GPAT4/GPAT4BLOT.tif]

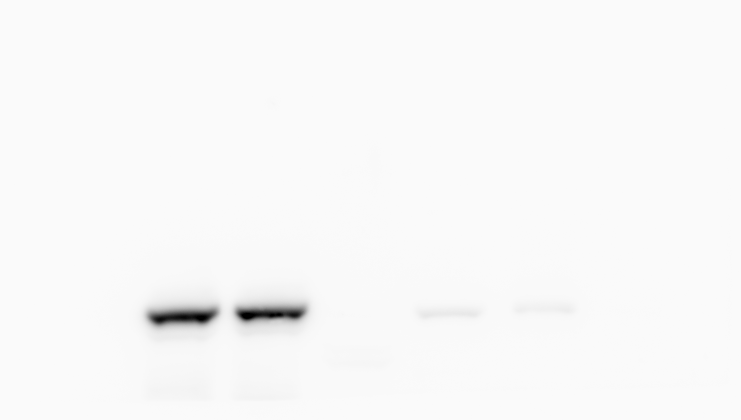

Supplement: Supplementary file 4 — Source Data Fig. 4 [file 44319_2023_9_MOESM4_ESM.zip › fig 3/b/BLOTS/MFN2/MFN2 BLOT.tif]

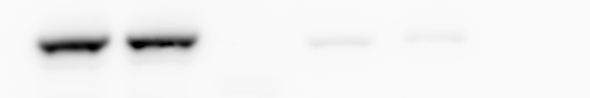

Supplement: Supplementary file 4 — Source Data Fig. 4 [file 44319_2023_9_MOESM4_ESM.zip › fig 3/b/BLOTS/MFN2/mfn2 ex1.tif]

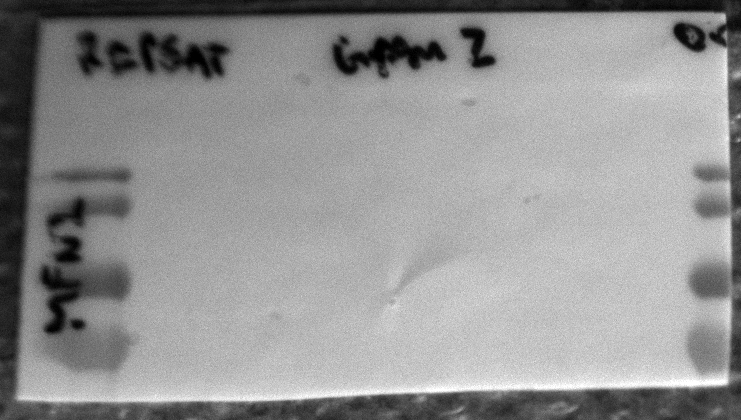

Supplement: Supplementary file 4 — Source Data Fig. 4 [file 44319_2023_9_MOESM4_ESM.zip › fig 3/b/BLOTS/MFN2/MFN2 MEMBRANE.tif]

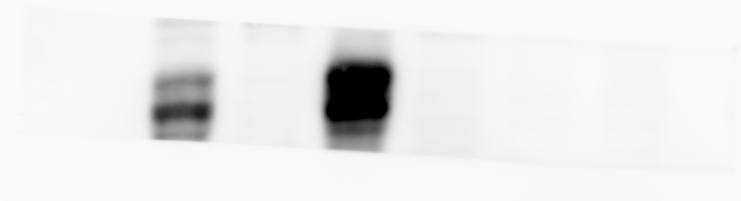

Supplement: Supplementary file 4 — Source Data Fig. 4 [file 44319_2023_9_MOESM4_ESM.zip › fig 3/b/BLOTS/MTCH2/MTCH2 BLOT.tif]

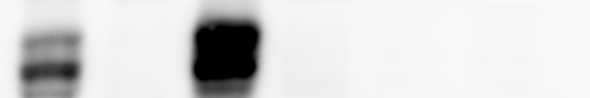

Supplement: Supplementary file 4 — Source Data Fig. 4 [file 44319_2023_9_MOESM4_ESM.zip › fig 3/b/BLOTS/MTCH2/mtch2 ex2.tif]

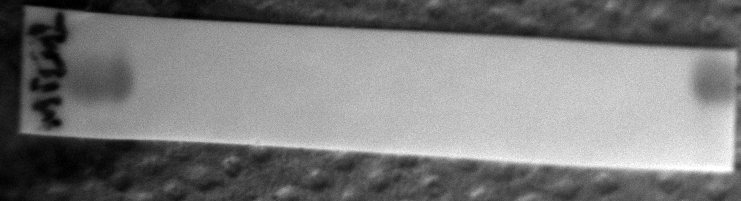

Supplement: Supplementary file 4 — Source Data Fig. 4 [file 44319_2023_9_MOESM4_ESM.zip › fig 3/b/BLOTS/MTCH2/MTCH2 MEMBRANE.tif]

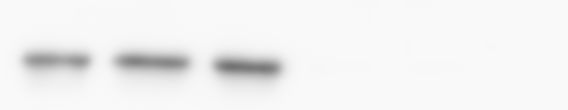

Supplement: Supplementary file 4 — Source Data Fig. 4 [file 44319_2023_9_MOESM4_ESM.zip › fig 3/b/BLOTS/TOMM40/TOM40.tif]

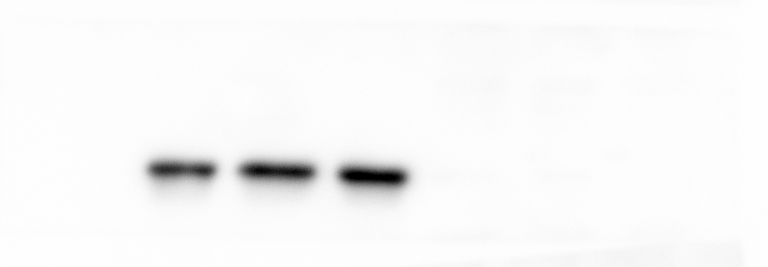

Supplement: Supplementary file 4 — Source Data Fig. 4 [file 44319_2023_9_MOESM4_ESM.zip › fig 3/b/BLOTS/TOMM40/TOMM40 BLOT.tif]

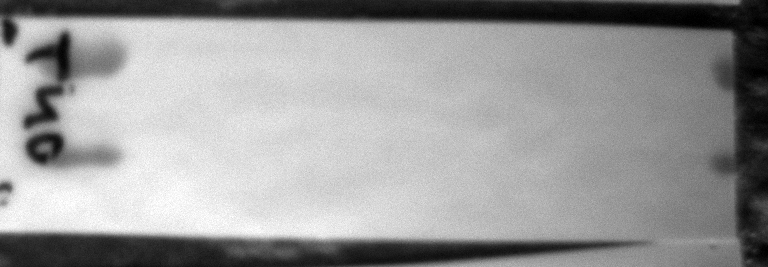

Supplement: Supplementary file 4 — Source Data Fig. 4 [file 44319_2023_9_MOESM4_ESM.zip › fig 3/b/BLOTS/TOMM40/TOMM40 MEMBRANE.tif]

FIG 3B GPATS/MTCH2 AND MFN2 EXPRESSION IN HM AND LM OF WT MTCH2 KO AND MFN2 KO MEFs

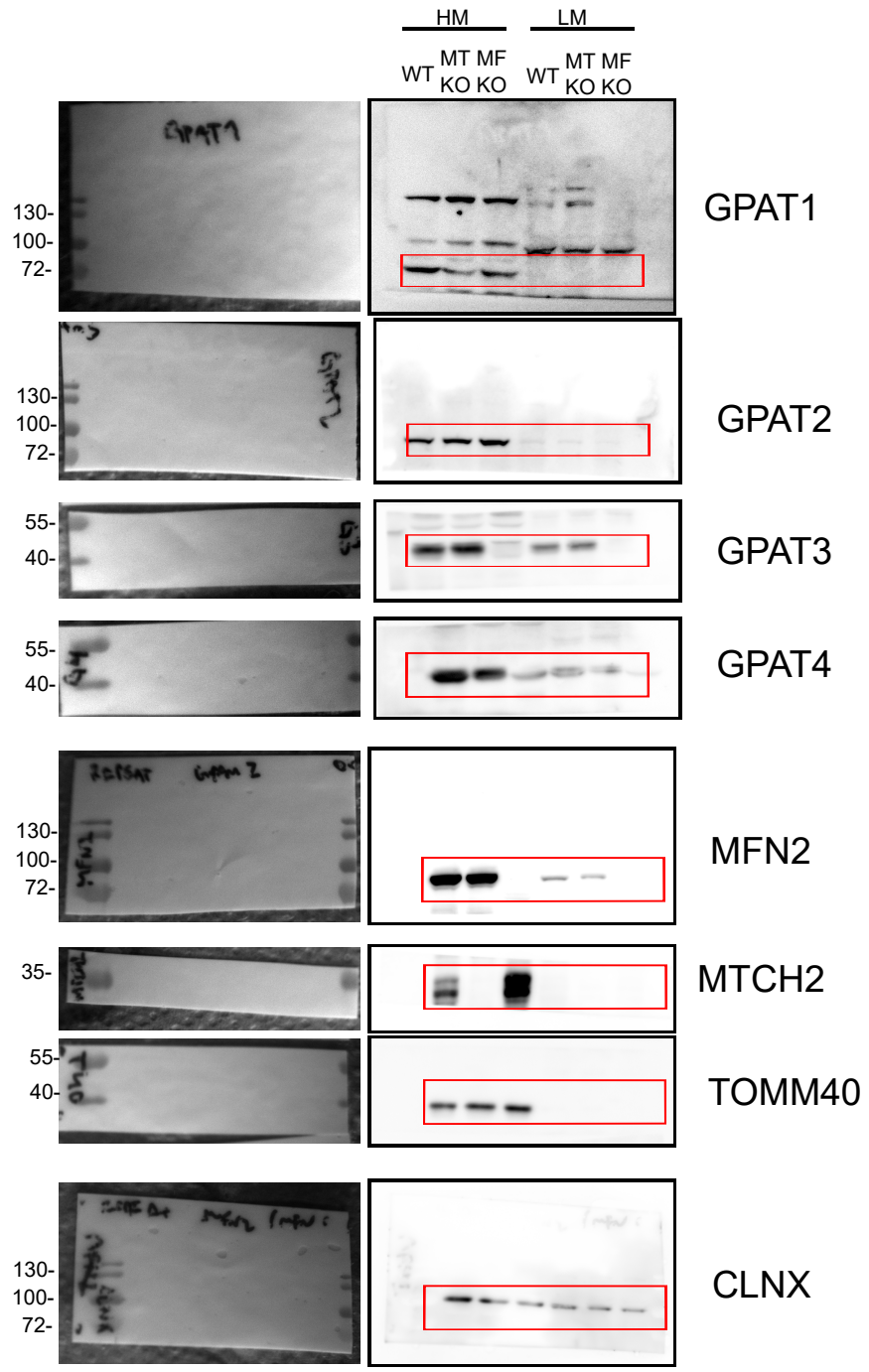

Supplement: Supplementary file 4 — Source Data Fig. 4 [file 44319_2023_9_MOESM4_ESM.zip › fig 3/b/UNCROPED BLOTS FIG3B.pdf]

## GPAT3/4

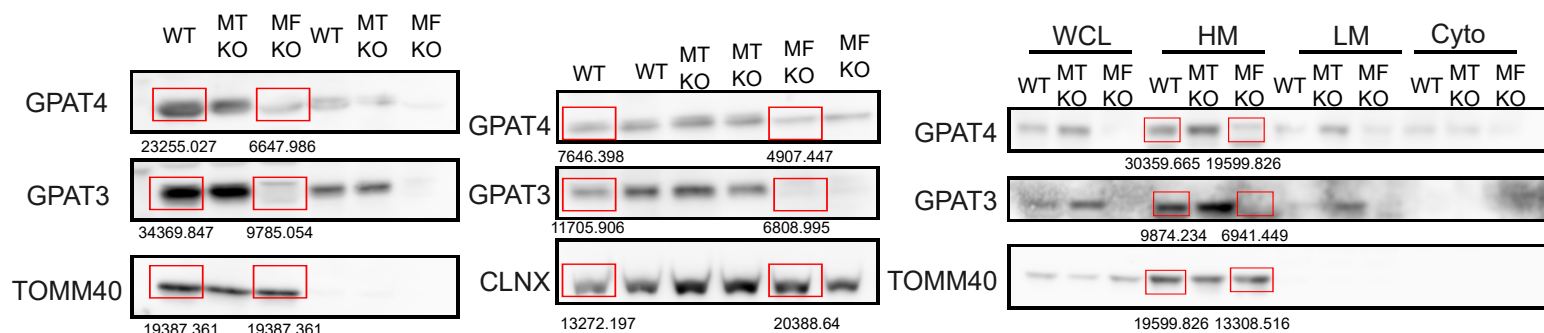

# MTCH2

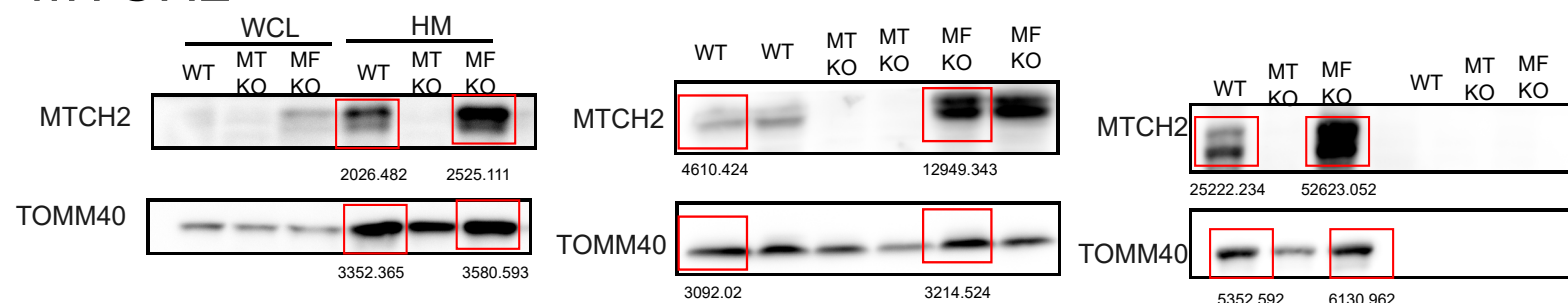

# MFN2

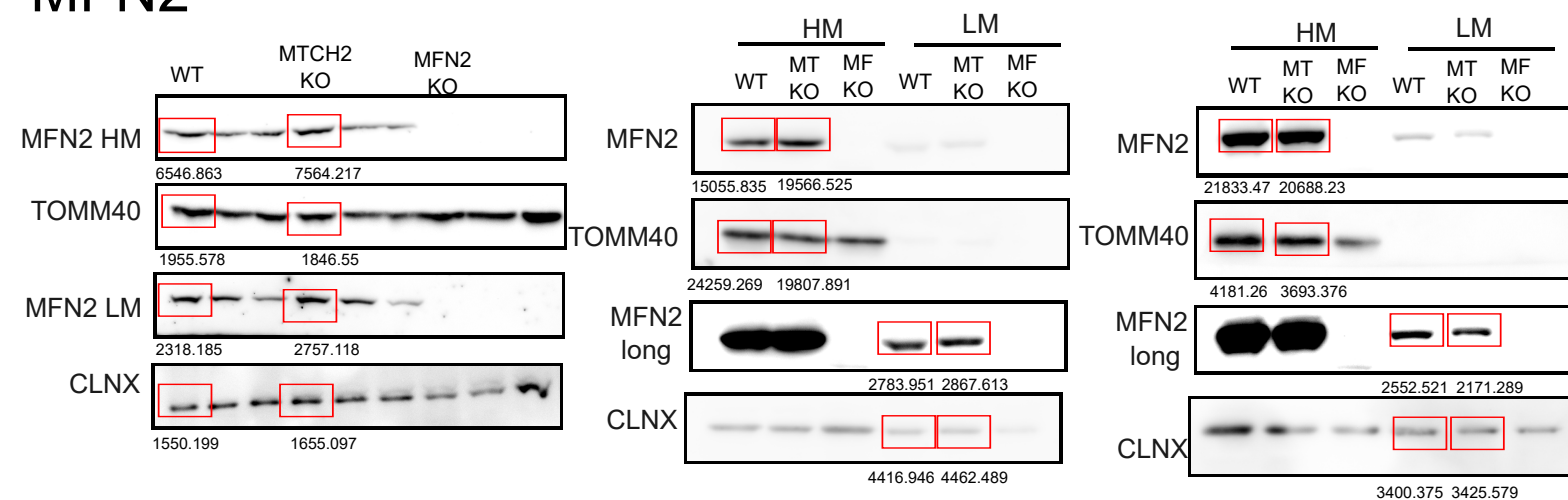

Supplement: Supplementary file 4 — Source Data Fig. 4 [file 44319_2023_9_MOESM4_ESM.zip › fig 3/c/densitometry/FIG 3C DENSITOMETRY.pdf]

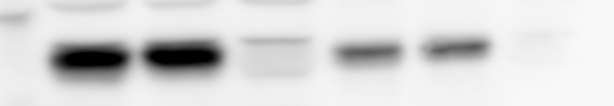

Supplement: Supplementary file 4 — Source Data Fig. 4 [file 44319_2023_9_MOESM4_ESM.zip › fig 3/c/densitometry/gpat3-4/1/GPAT3.tif]

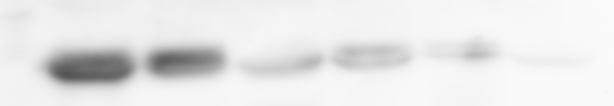

Supplement: Supplementary file 4 — Source Data Fig. 4 [file 44319_2023_9_MOESM4_ESM.zip › fig 3/c/densitometry/gpat3-4/1/GPAT4.tif]

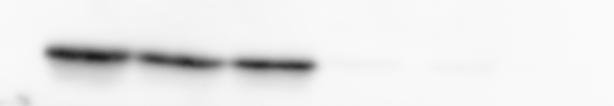

Supplement: Supplementary file 4 — Source Data Fig. 4 [file 44319_2023_9_MOESM4_ESM.zip › fig 3/c/densitometry/gpat3-4/1/TOM40.tif]

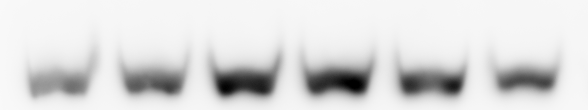

Supplement: Supplementary file 4 — Source Data Fig. 4 [file 44319_2023_9_MOESM4_ESM.zip › fig 3/c/densitometry/gpat3-4/2/CLNX.tif]

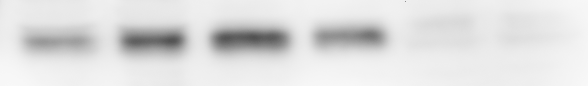

Supplement: Supplementary file 4 — Source Data Fig. 4 [file 44319_2023_9_MOESM4_ESM.zip › fig 3/c/densitometry/gpat3-4/2/GPAT3.tif]

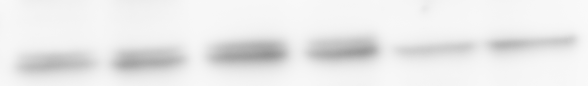

Supplement: Supplementary file 4 — Source Data Fig. 4 [file 44319_2023_9_MOESM4_ESM.zip › fig 3/c/densitometry/gpat3-4/2/GPAT4.tif]

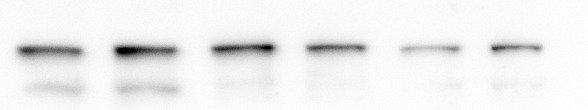

Supplement: Supplementary file 4 — Source Data Fig. 4 [file 44319_2023_9_MOESM4_ESM.zip › fig 3/c/densitometry/gpat3-4/2/IP3R3.tif]

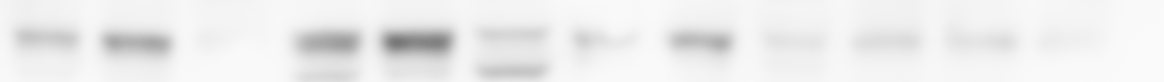

Supplement: Supplementary file 4 — Source Data Fig. 4 [file 44319_2023_9_MOESM4_ESM.zip › fig 3/c/densitometry/gpat3-4/3/gpat3.tif]

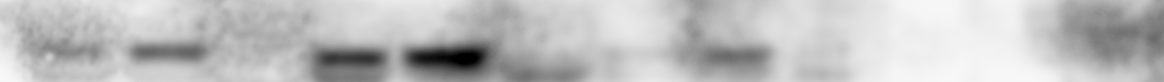

Supplement: Supplementary file 4 — Source Data Fig. 4 [file 44319_2023_9_MOESM4_ESM.zip › fig 3/c/densitometry/gpat3-4/3/gpat4.tif]

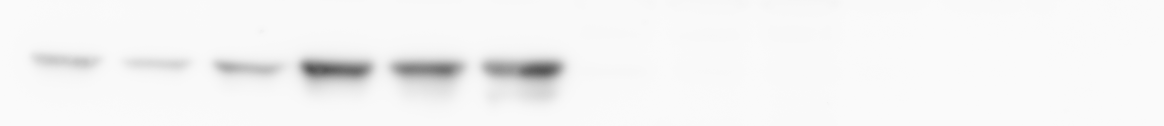

Supplement: Supplementary file 4 — Source Data Fig. 4 [file 44319_2023_9_MOESM4_ESM.zip › fig 3/c/densitometry/gpat3-4/3/tom40.tif]

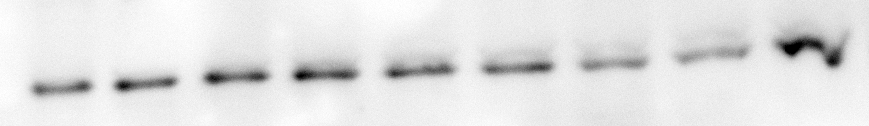

Supplement: Supplementary file 4 — Source Data Fig. 4 [file 44319_2023_9_MOESM4_ESM.zip › fig 3/c/densitometry/mfn2/1/calnexin.tif]

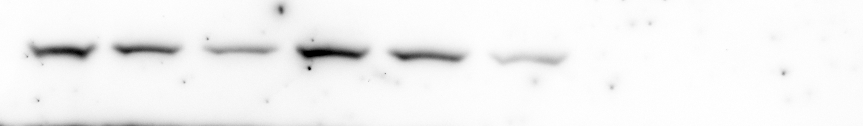

Supplement: Supplementary file 4 — Source Data Fig. 4 [file 44319_2023_9_MOESM4_ESM.zip › fig 3/c/densitometry/mfn2/1/mfn2 er.tif]

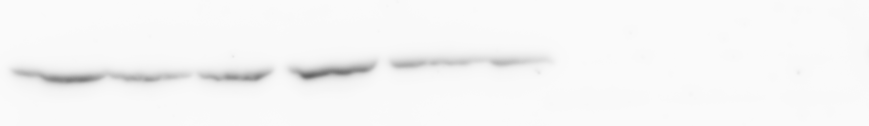

Supplement: Supplementary file 4 — Source Data Fig. 4 [file 44319_2023_9_MOESM4_ESM.zip › fig 3/c/densitometry/mfn2/1/mfn2.tif]

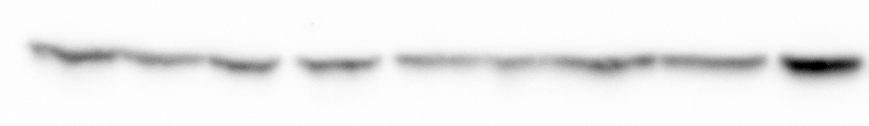

Supplement: Supplementary file 4 — Source Data Fig. 4 [file 44319_2023_9_MOESM4_ESM.zip › fig 3/c/densitometry/mfn2/1/tom40.tif]

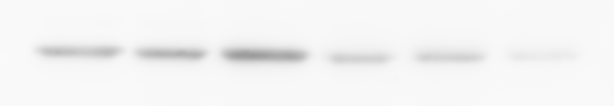

Supplement: Supplementary file 4 — Source Data Fig. 4 [file 44319_2023_9_MOESM4_ESM.zip › fig 3/c/densitometry/mfn2/2/CALNEXINB.tif]

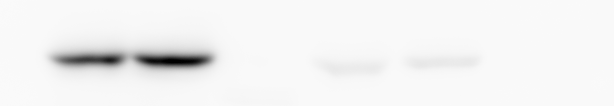

Supplement: Supplementary file 4 — Source Data Fig. 4 [file 44319_2023_9_MOESM4_ESM.zip › fig 3/c/densitometry/mfn2/2/MFN2.tif]

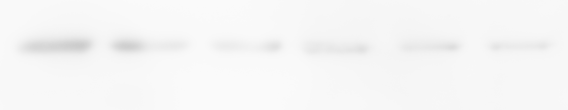

Supplement: Supplementary file 4 — Source Data Fig. 4 [file 44319_2023_9_MOESM4_ESM.zip › fig 3/c/densitometry/mfn2/3/clnx extra.tif]

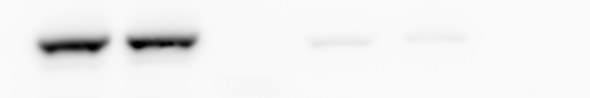

Supplement: Supplementary file 4 — Source Data Fig. 4 [file 44319_2023_9_MOESM4_ESM.zip › fig 3/c/densitometry/mfn2/3/mfn2 ex1.tif]

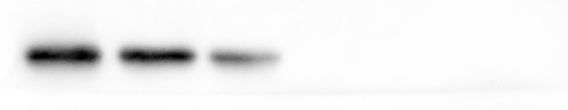

Supplement: Supplementary file 4 — Source Data Fig. 4 [file 44319_2023_9_MOESM4_ESM.zip › fig 3/c/densitometry/mfn2/3/TOM40 B.tif]

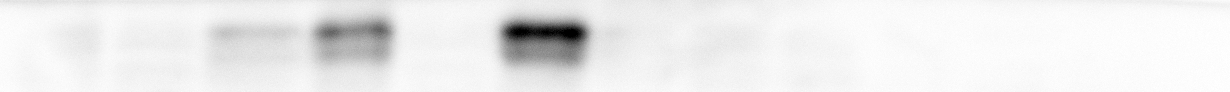

Supplement: Supplementary file 4 — Source Data Fig. 4 [file 44319_2023_9_MOESM4_ESM.zip › fig 3/c/densitometry/mtch2/1/MTCH2 396.tif]

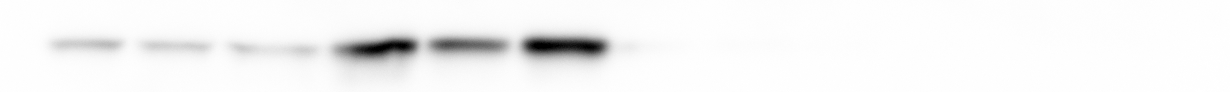

Supplement: Supplementary file 4 — Source Data Fig. 4 [file 44319_2023_9_MOESM4_ESM.zip › fig 3/c/densitometry/mtch2/1/TOM40.tif]

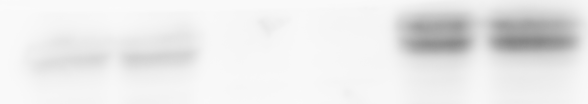

Supplement: Supplementary file 4 — Source Data Fig. 4 [file 44319_2023_9_MOESM4_ESM.zip › fig 3/c/densitometry/mtch2/2/MTCH2.tif]

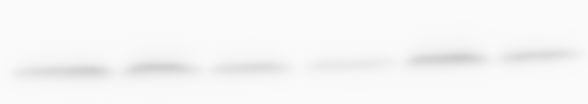

Supplement: Supplementary file 4 — Source Data Fig. 4 [file 44319_2023_9_MOESM4_ESM.zip › fig 3/c/densitometry/mtch2/2/TOM40.tif]

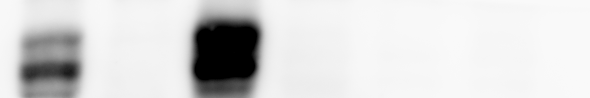

Supplement: Supplementary file 4 — Source Data Fig. 4 [file 44319_2023_9_MOESM4_ESM.zip › fig 3/c/densitometry/mtch2/3/mtch2 ex2.tif]

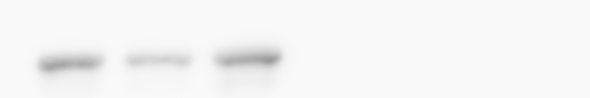

Supplement: Supplementary file 4 — Source Data Fig. 4 [file 44319_2023_9_MOESM4_ESM.zip › fig 3/c/densitometry/mtch2/3/tom40ex2.tif]

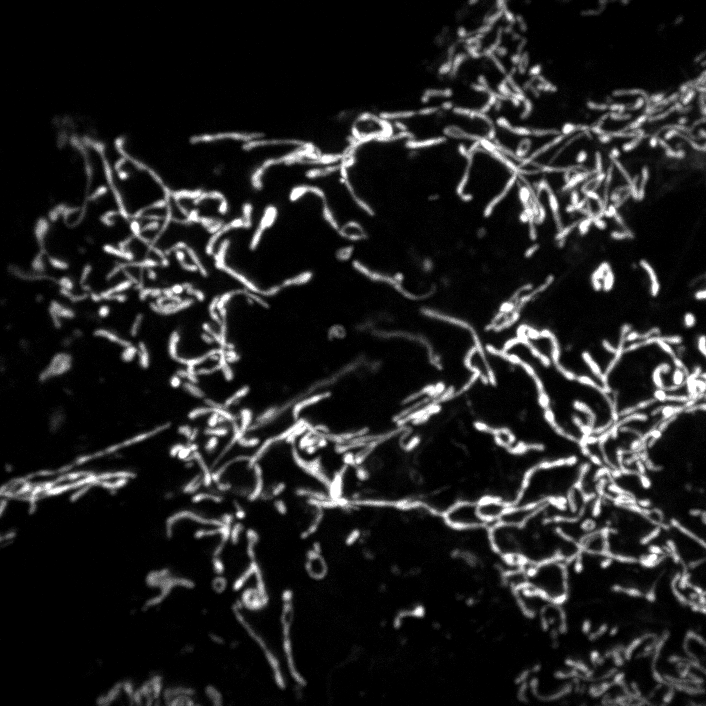

Supplement: Supplementary file 4 — Source Data Fig. 4 [file 44319_2023_9_MOESM4_ESM.zip › fig 3/d/IMAGES/CONTROL/MAX_ff mtdr44_thumb_w1Con-Cy5-1 8B.tif]

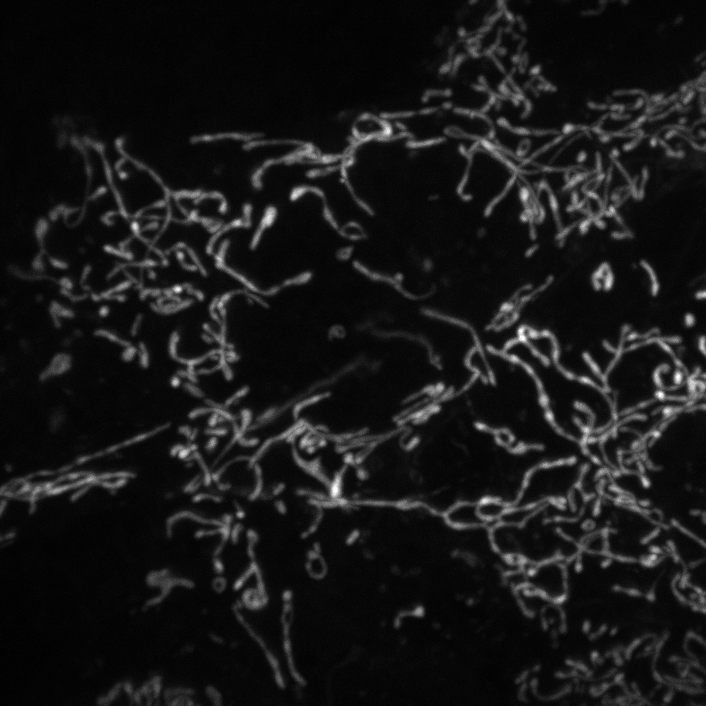

Supplement: Supplementary file 4 — Source Data Fig. 4 [file 44319_2023_9_MOESM4_ESM.zip › fig 3/d/IMAGES/CONTROL/MAX_ff mtdr44_thumb_w1Con-Cy5-1.tif]

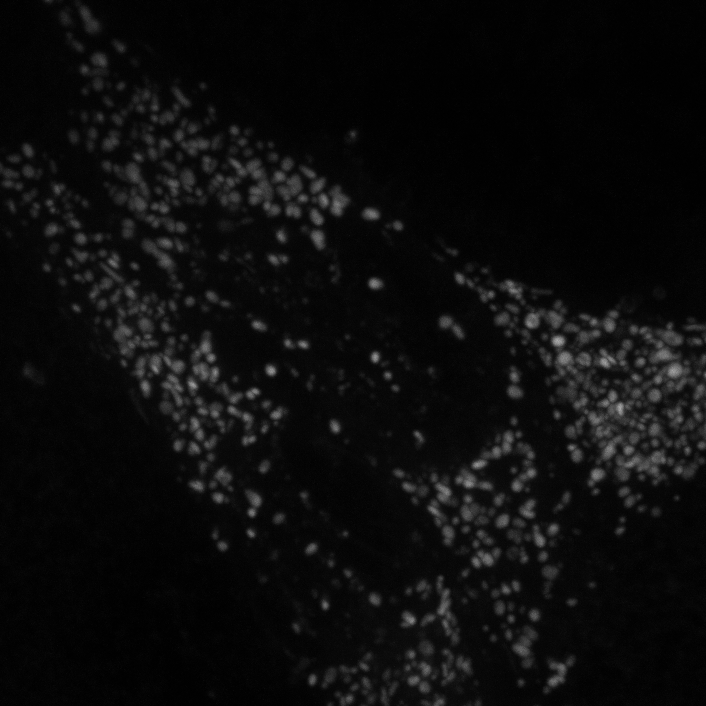

Supplement: Supplementary file 4 — Source Data Fig. 4 [file 44319_2023_9_MOESM4_ESM.zip › fig 3/d/IMAGES/GPATi/MAX_ff fsg 16hs mtdr6_thumb_w1Con-Cy5-1.tif]

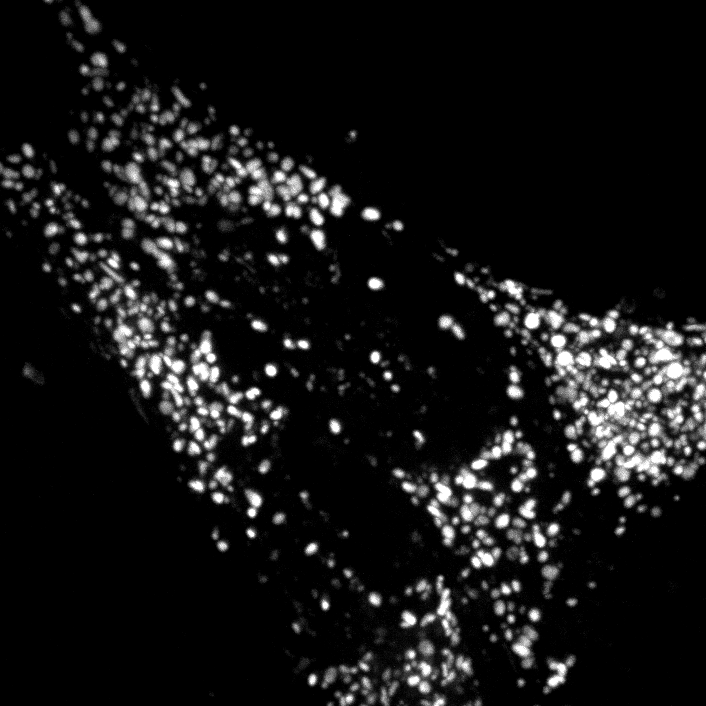

Supplement: Supplementary file 4 — Source Data Fig. 4 [file 44319_2023_9_MOESM4_ESM.zip › fig 3/d/IMAGES/GPATi/MAX_ff fsg 16hs mtdr6_thumb_w1Con-Cy5-1.TIF 8B.tif]

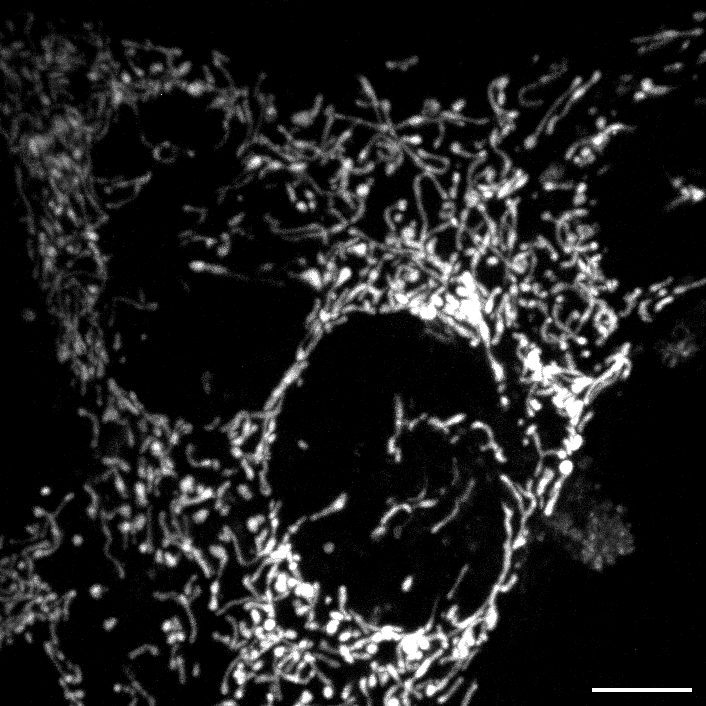

Supplement: Supplementary file 4 — Source Data Fig. 4 [file 44319_2023_9_MOESM4_ESM.zip › fig 3/d/IMAGES/WASH/MAX_ff 4hs wash mtdr25_thumb_w1Con-Cy5-1 8B-2 10um scale.tif]

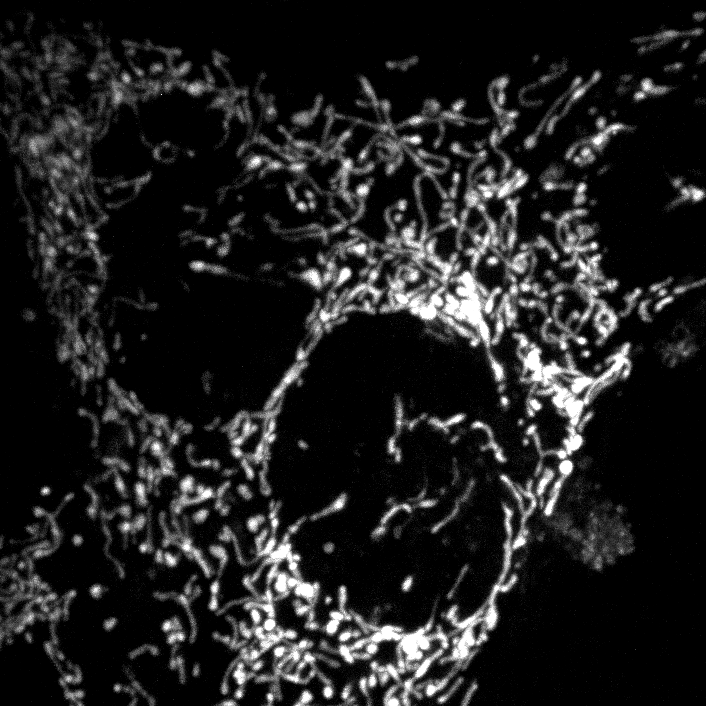

Supplement: Supplementary file 4 — Source Data Fig. 4 [file 44319_2023_9_MOESM4_ESM.zip › fig 3/d/IMAGES/WASH/MAX_ff 4hs wash mtdr25_thumb_w1Con-Cy5-1 8B.tif]

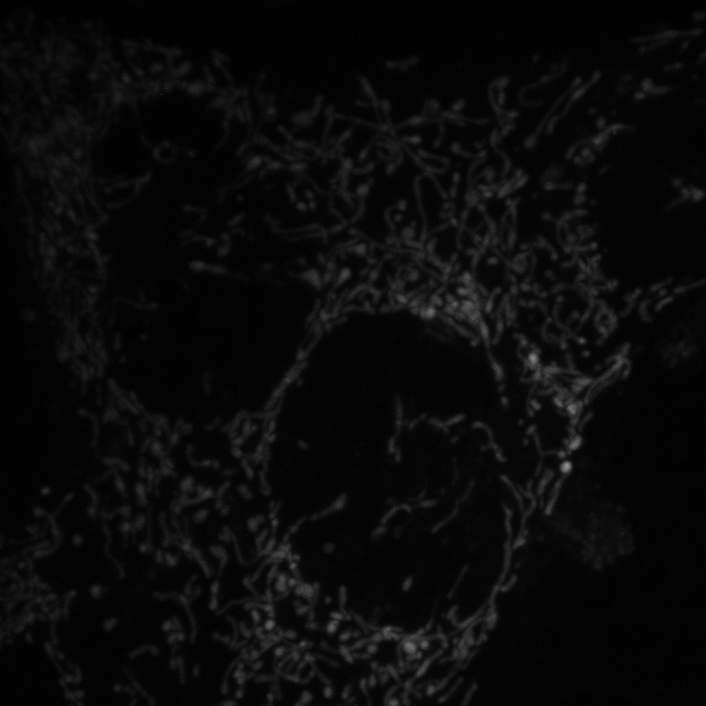

Supplement: Supplementary file 4 — Source Data Fig. 4 [file 44319_2023_9_MOESM4_ESM.zip › fig 3/d/IMAGES/WASH/MAX_ff 4hs wash mtdr25_thumb_w1Con-Cy5-1.tif]

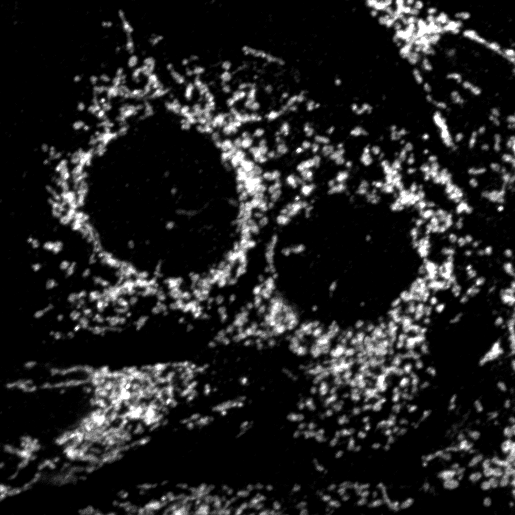

Supplement: Supplementary file 4 — Source Data Fig. 4 [file 44319_2023_9_MOESM4_ESM.zip › fig 3/f/IMAGES/MFN2 KO CTRL/C1-MAX_MEFS MFN2KO MTCH2 GFP TOM20CY5 DAPI060-1.tif]

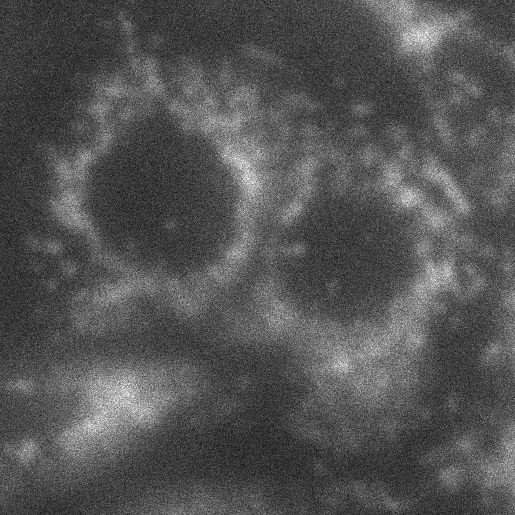

Supplement: Supplementary file 4 — Source Data Fig. 4 [file 44319_2023_9_MOESM4_ESM.zip › fig 3/f/IMAGES/MFN2 KO CTRL/MEFS MFN2KO MTCH2 GFP TOM20CY5 DAPI060-1.tif]

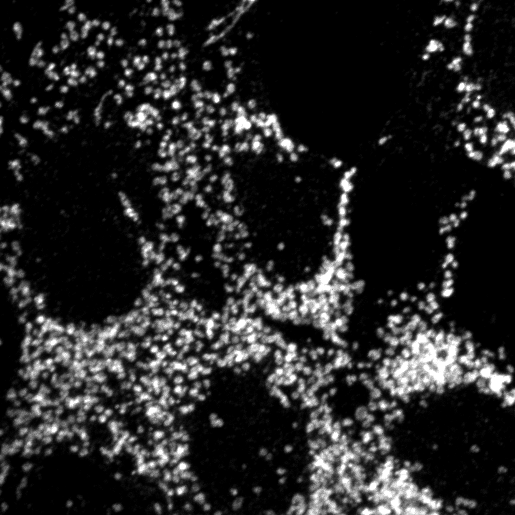

Supplement: Supplementary file 4 — Source Data Fig. 4 [file 44319_2023_9_MOESM4_ESM.zip › fig 3/f/IMAGES/MFN2 KO GPATi/MAX_MEFS MFN2KO FSG67 20H MTCH2 GFP TOM20CY5 DAPI090-1.tif (RGB).tif (red).tif]

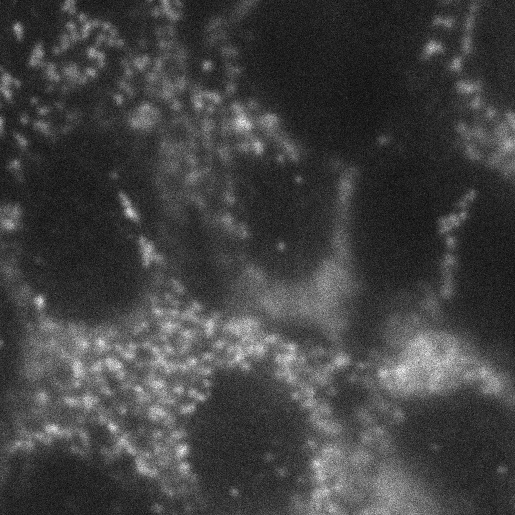

Supplement: Supplementary file 4 — Source Data Fig. 4 [file 44319_2023_9_MOESM4_ESM.zip › fig 3/f/IMAGES/MFN2 KO GPATi/MEFS MFN2KO FSG67 20H MTCH2 GFP TOM20CY5 DAPI090-1.tif]

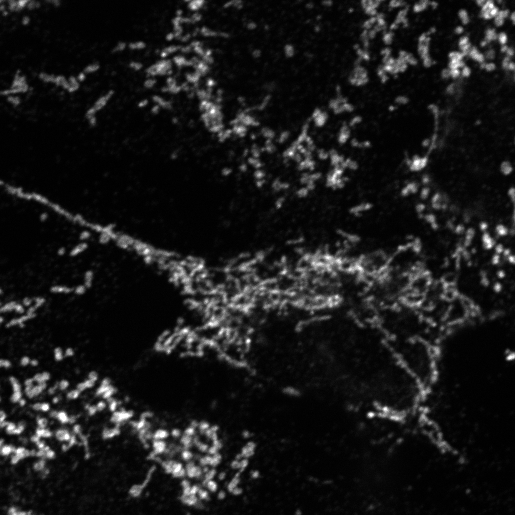

Supplement: Supplementary file 4 — Source Data Fig. 4 [file 44319_2023_9_MOESM4_ESM.zip › fig 3/f/IMAGES/MFN2 KO MTCH2 OE CTRL/MEFS MFN2KO MTCH2 GFP TOM20CY5 DAPI071-2.tif]

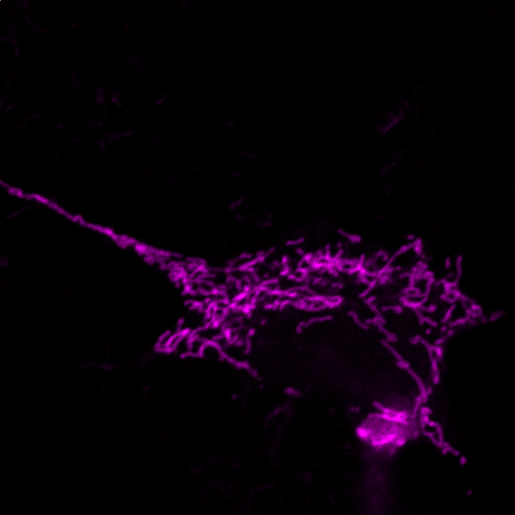

Supplement: Supplementary file 4 — Source Data Fig. 4 [file 44319_2023_9_MOESM4_ESM.zip › fig 3/f/IMAGES/MFN2 KO MTCH2 OE CTRL/MEFS MFN2KO MTCH2 GFP TOM20CY5 DAPI071-2.tif (RGB) mtch.tif]

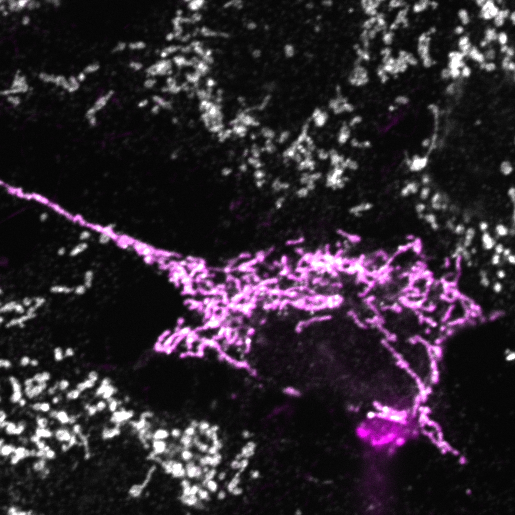

Supplement: Supplementary file 4 — Source Data Fig. 4 [file 44319_2023_9_MOESM4_ESM.zip › fig 3/f/IMAGES/MFN2 KO MTCH2 OE CTRL/MEFS MFN2KO MTCH2 GFP TOM20CY5 DAPI071-2.tif (RGB)comp.tif]

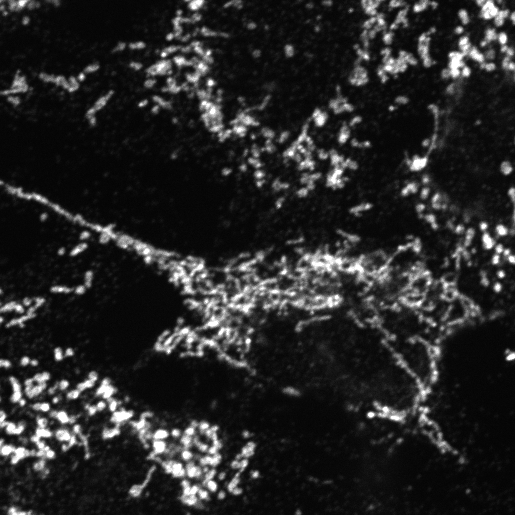

Supplement: Supplementary file 4 — Source Data Fig. 4 [file 44319_2023_9_MOESM4_ESM.zip › fig 3/f/IMAGES/MFN2 KO MTCH2 OE CTRL/MEFS MFN2KO MTCH2 GFP TOM20CY5 DAPI071-2.tif (RGB)mito.tif]

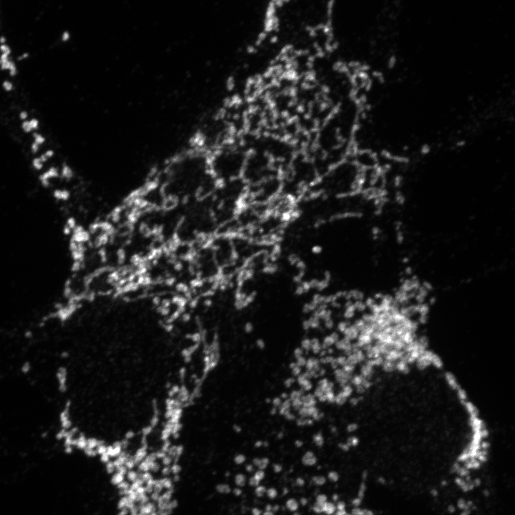

Supplement: Supplementary file 4 — Source Data Fig. 4 [file 44319_2023_9_MOESM4_ESM.zip › fig 3/f/IMAGES/MFN2 KO MTCH2 OE GPATi/MAX_MEFS MFN2KO FSG67 20H MTCH2 GFP TOM20CY5 DAPI091-1.tif]

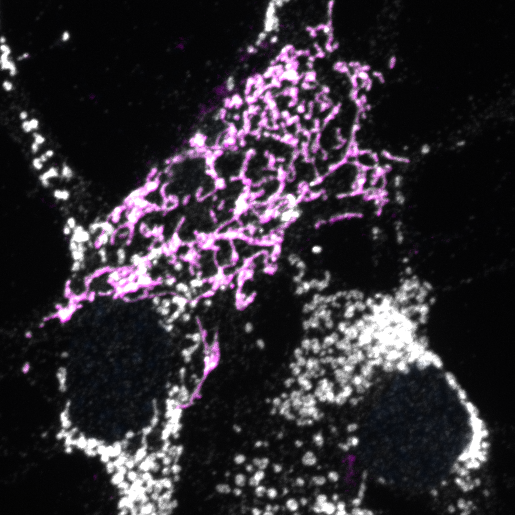

Supplement: Supplementary file 4 — Source Data Fig. 4 [file 44319_2023_9_MOESM4_ESM.zip › fig 3/f/IMAGES/MFN2 KO MTCH2 OE GPATi/MAX_MEFS MFN2KO FSG67 20H MTCH2 GFP TOM20CY5 DAPI091-1.tif (RGB) comp.tif]

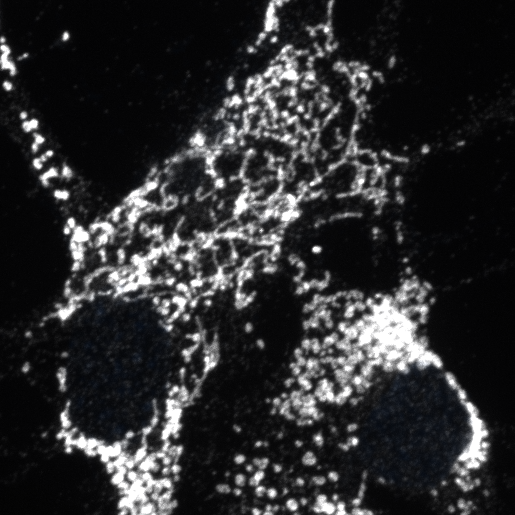

Supplement: Supplementary file 4 — Source Data Fig. 4 [file 44319_2023_9_MOESM4_ESM.zip › fig 3/f/IMAGES/MFN2 KO MTCH2 OE GPATi/MAX_MEFS MFN2KO FSG67 20H MTCH2 GFP TOM20CY5 DAPI091-1.tif (RGB) mito.tif]

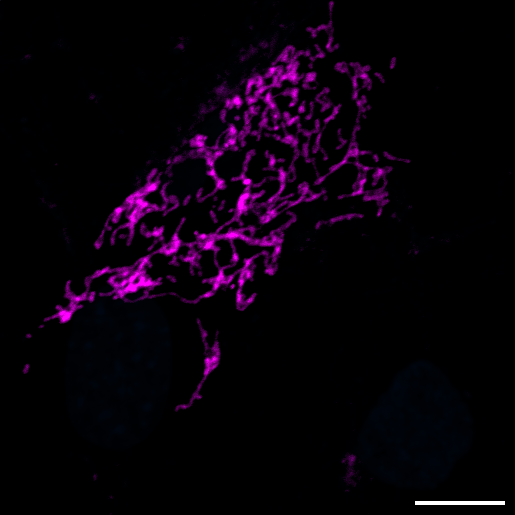

Supplement: Supplementary file 4 — Source Data Fig. 4 [file 44319_2023_9_MOESM4_ESM.zip › fig 3/f/IMAGES/MFN2 KO MTCH2 OE GPATi/MAX_MEFS MFN2KO FSG67 20H MTCH2 GFP TOM20CY5 DAPI091-1.tif (RGB) mtch2-1scal.tif]

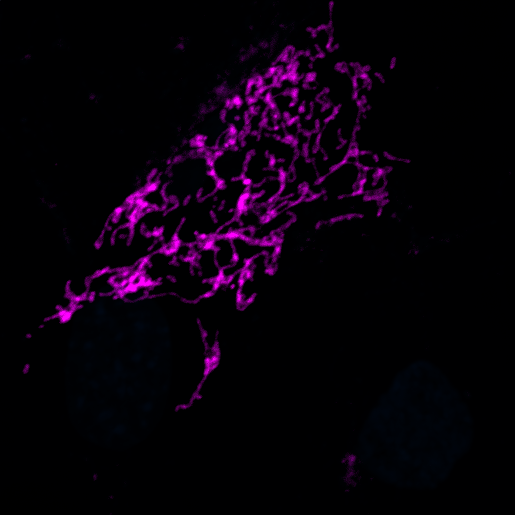

Supplement: Supplementary file 4 — Source Data Fig. 4 [file 44319_2023_9_MOESM4_ESM.zip › fig 3/f/IMAGES/MFN2 KO MTCH2 OE GPATi/MAX_MEFS MFN2KO FSG67 20H MTCH2 GFP TOM20CY5 DAPI091-1.tif (RGB) mtch2.tif]

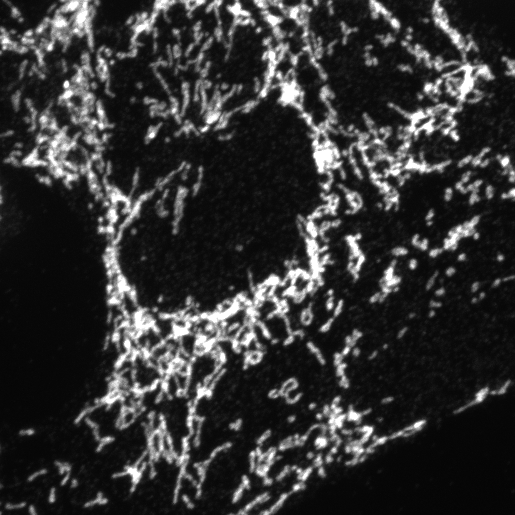

Supplement: Supplementary file 4 — Source Data Fig. 4 [file 44319_2023_9_MOESM4_ESM.zip › fig 3/f/IMAGES/WT CTRL/MAX_MEFS FF53 MTCH2 GFP TOM20CY5 DAPI030-1.tif (RGB).tif]

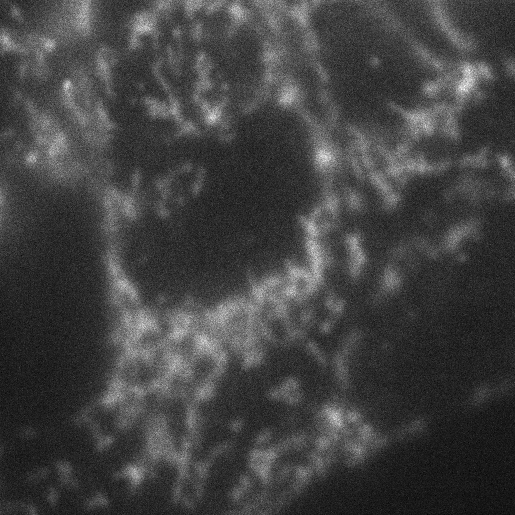

Supplement: Supplementary file 4 — Source Data Fig. 4 [file 44319_2023_9_MOESM4_ESM.zip › fig 3/f/IMAGES/WT CTRL/MEFS FF53 MTCH2 GFP TOM20CY5 DAPI030-1.tif]

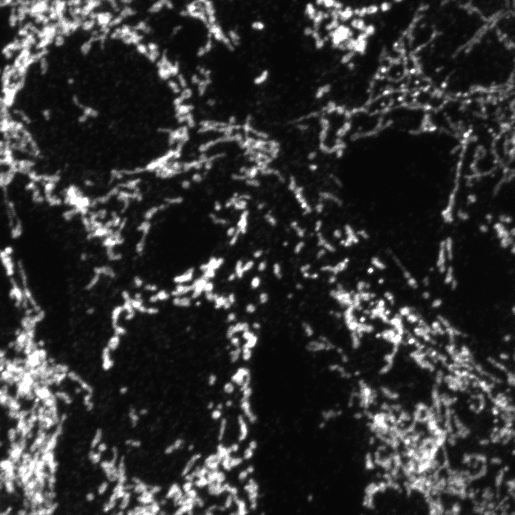

Supplement: Supplementary file 4 — Source Data Fig. 4 [file 44319_2023_9_MOESM4_ESM.zip › fig 3/f/IMAGES/WT GPATi/MAX_MEFS FF53 FSG67 20H MTCH2 GFP TOM20CY5 DAPI043-1.tif (RGB).tif]

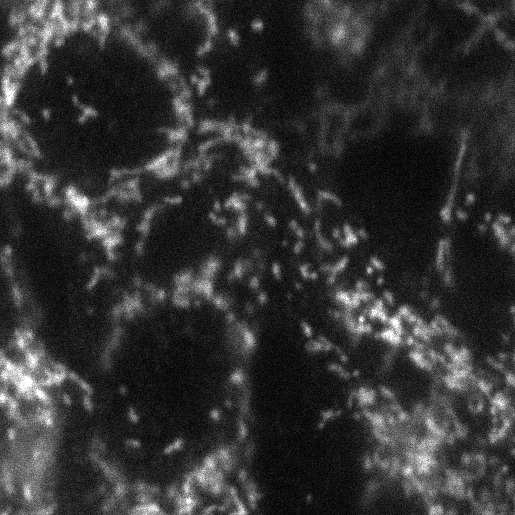

Supplement: Supplementary file 4 — Source Data Fig. 4 [file 44319_2023_9_MOESM4_ESM.zip › fig 3/f/IMAGES/WT GPATi/MEFS FF53 FSG67 20H MTCH2 GFP TOM20CY5 DAPI043-1.tif]

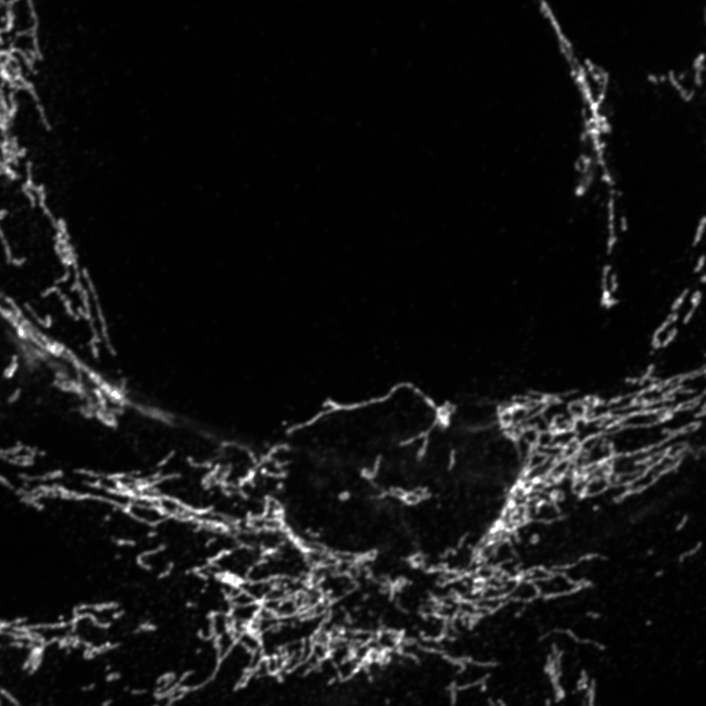

Supplement: Supplementary file 4 — Source Data Fig. 4 [file 44319_2023_9_MOESM4_ESM.zip › fig 3/f/IMAGES/WT MTCH2 OE CTRL/MEFS FF53 MTCH2 GFP TOM20CY5 DAPI001-1.tif]

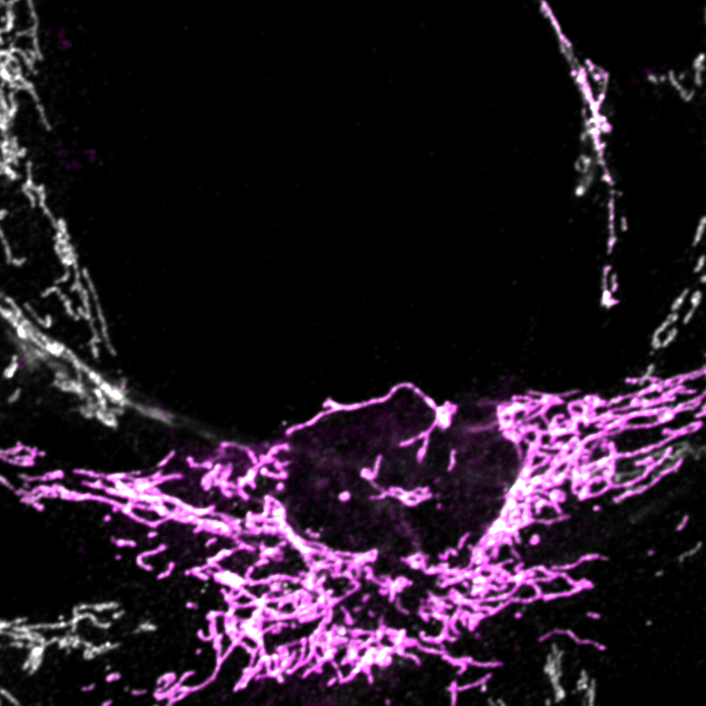

Supplement: Supplementary file 4 — Source Data Fig. 4 [file 44319_2023_9_MOESM4_ESM.zip › fig 3/f/IMAGES/WT MTCH2 OE CTRL/MEFS FF53 MTCH2 GFP TOM20CY5 DAPI001-1.tif (RGB)comp.tif]

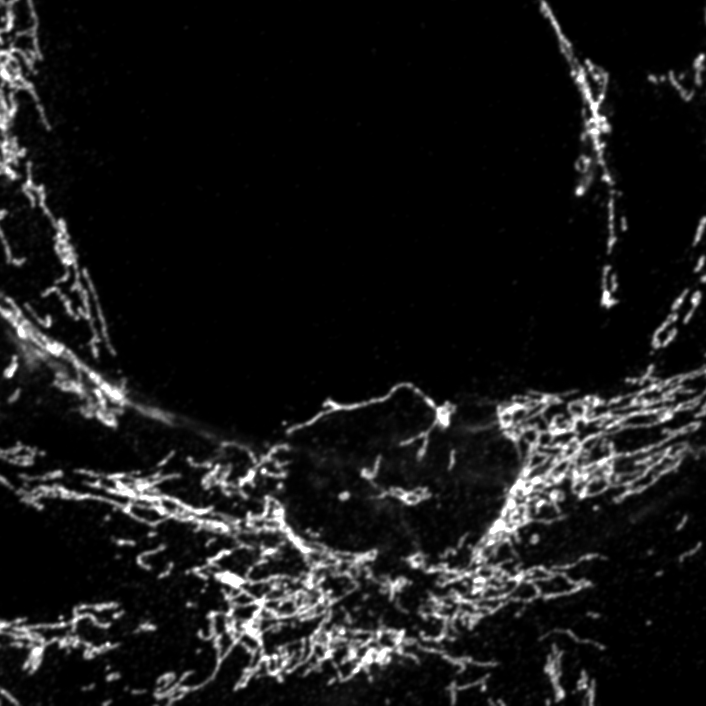

Supplement: Supplementary file 4 — Source Data Fig. 4 [file 44319_2023_9_MOESM4_ESM.zip › fig 3/f/IMAGES/WT MTCH2 OE CTRL/MEFS FF53 MTCH2 GFP TOM20CY5 DAPI001-1.tif (RGB)mito.tif]

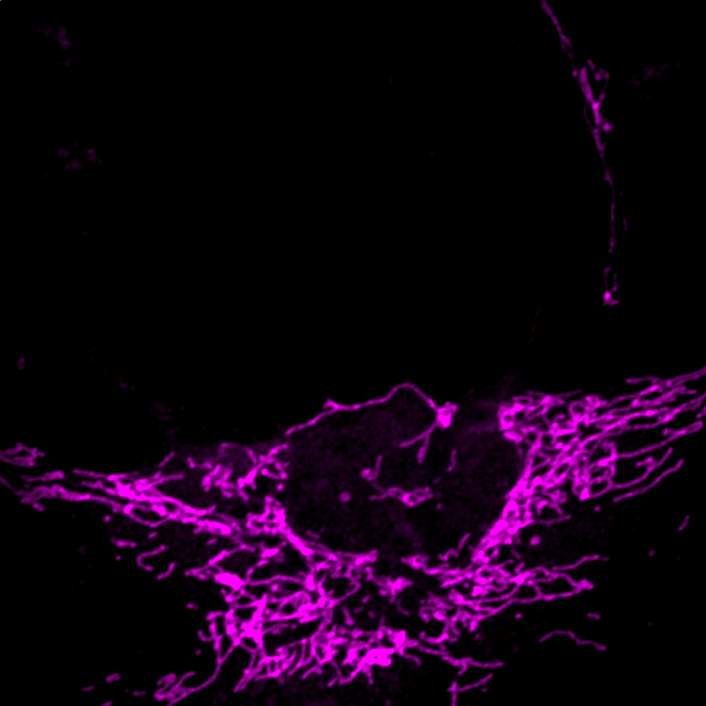

Supplement: Supplementary file 4 — Source Data Fig. 4 [file 44319_2023_9_MOESM4_ESM.zip › fig 3/f/IMAGES/WT MTCH2 OE CTRL/MEFS FF53 MTCH2 GFP TOM20CY5 DAPI001-1.tif (RGB)mtxh.tif]

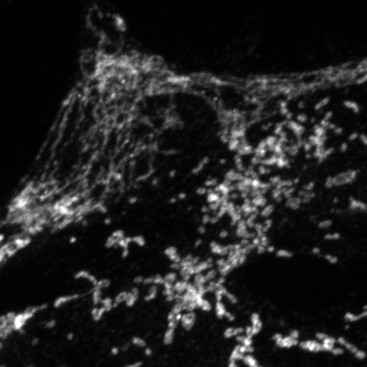

Supplement: Supplementary file 4 — Source Data Fig. 4 [file 44319_2023_9_MOESM4_ESM.zip › fig 3/f/IMAGES/WT MTCH2 OE GPATi/MAX_MEFS FF53 FSG67 20H MTCH2 GFP TOM20CY5 DAPI032-1-1.tif]

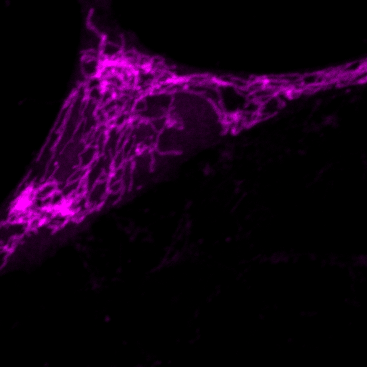

Supplement: Supplementary file 4 — Source Data Fig. 4 [file 44319_2023_9_MOESM4_ESM.zip › fig 3/f/IMAGES/WT MTCH2 OE GPATi/MAX_MEFS FF53 FSG67 20H MTCH2 GFP TOM20CY5 DAPI032-1-1.tif (RGB) mth2.tif]

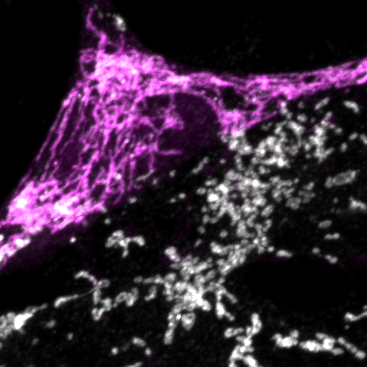

Supplement: Supplementary file 4 — Source Data Fig. 4 [file 44319_2023_9_MOESM4_ESM.zip › fig 3/f/IMAGES/WT MTCH2 OE GPATi/MAX_MEFS FF53 FSG67 20H MTCH2 GFP TOM20CY5 DAPI032-1-1.tif (RGB)comp.tif]

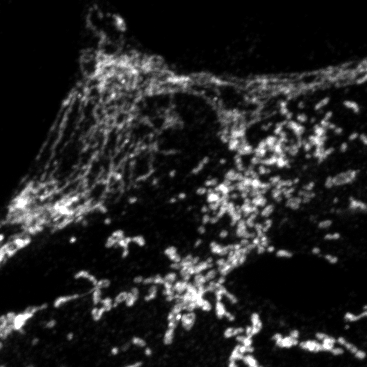

Supplement: Supplementary file 4 — Source Data Fig. 4 [file 44319_2023_9_MOESM4_ESM.zip › fig 3/f/IMAGES/WT MTCH2 OE GPATi/MAX_MEFS FF53 FSG67 20H MTCH2 GFP TOM20CY5 DAPI032-1-1.tif (RGB)tom.tif]

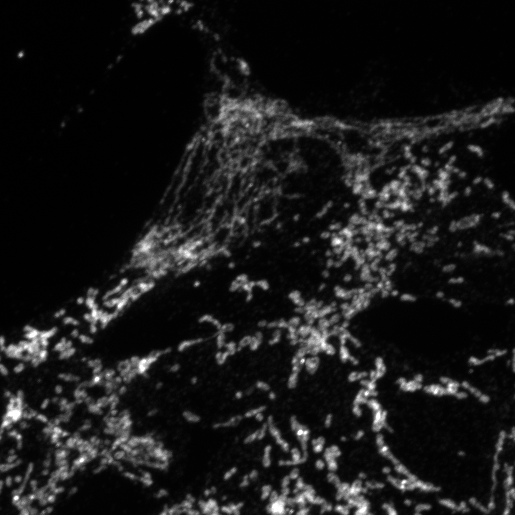

Supplement: Supplementary file 4 — Source Data Fig. 4 [file 44319_2023_9_MOESM4_ESM.zip › fig 3/f/IMAGES/WT MTCH2 OE GPATi/MAX_MEFS FF53 FSG67 20H MTCH2 GFP TOM20CY5 DAPI032-1.tif]

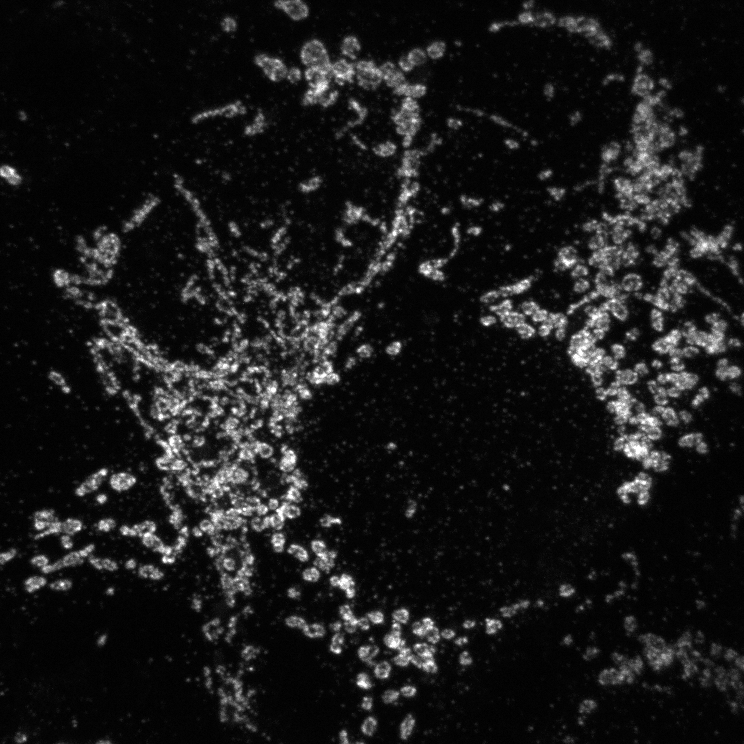

Supplement: Supplementary file 4 — Source Data Fig. 4 [file 44319_2023_9_MOESM4_ESM.zip › fig 3/i/IMAGES/control/mito MAX_mef 11c11ko control mfn2 myc 647 mitodsRED TOM40_511_thumb_w1Con-mcherry-1.tif]
