# Supplementary figures and images for: MTCH2 cooperates with MFN2 and lysophosphatidic acid synthesis to sustain mitochondrial fusion (part 3 of 6)
Source: EMBO Rep. 2023 Dec 14;25(1):8. doi: 10.1038/s44319-023-00009-1 (PMC10897490; doi:10.1038/s44319-023-00009-1)

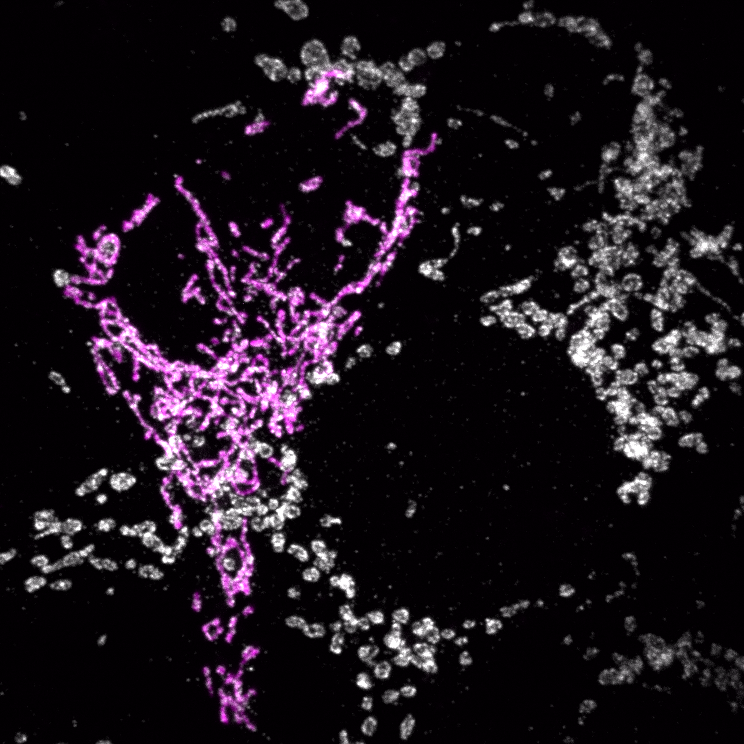

Supplement: Supplementary file 4 — Source Data Fig. 4 [file 44319_2023_9_MOESM4_ESM.zip › fig 3/i/IMAGES/control/mito MAX_mef 11c11ko control mfn2 myc 647 mitodsRED TOM40_511_thumb_w1Con-mcherry-1.tif (RGB)OPT.tif]

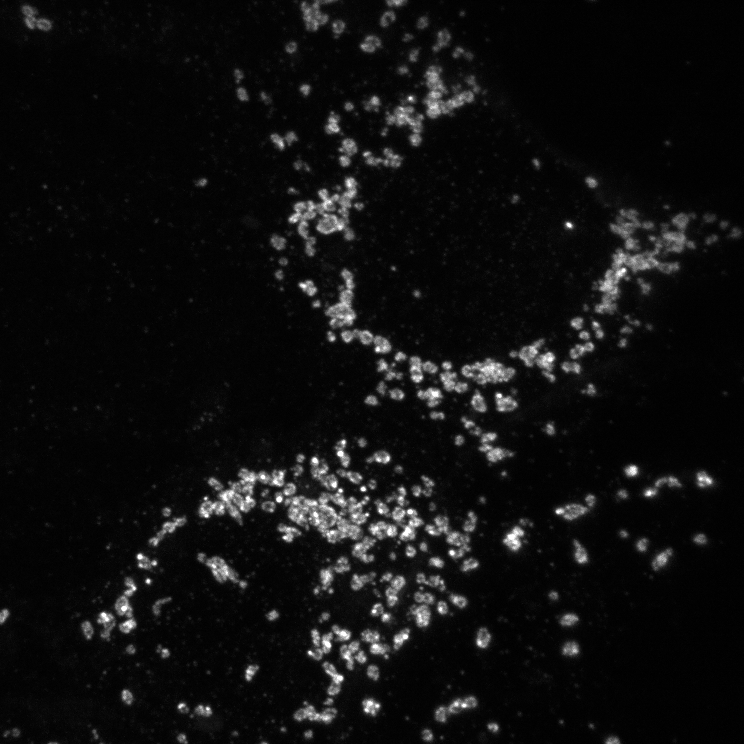

Supplement: Supplementary file 4 — Source Data Fig. 4 [file 44319_2023_9_MOESM4_ESM.zip › fig 3/i/IMAGES/fsg67/mito MAX_mef 11c11koFGS67 mfn2 myc 647 mitodsRED TOM40_542_thumb_w1Con-mcherry-1.tif]

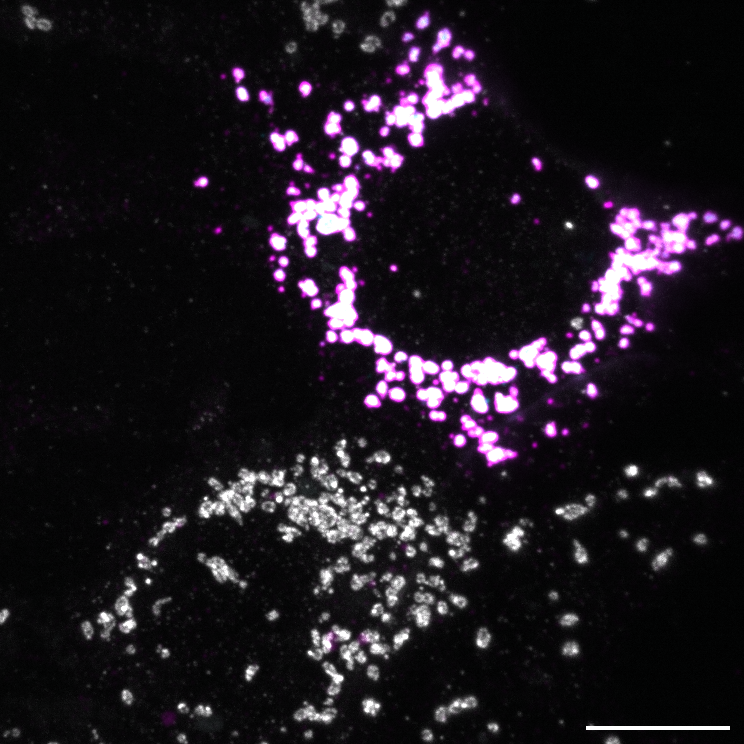

Supplement: Supplementary file 4 — Source Data Fig. 4 [file 44319_2023_9_MOESM4_ESM.zip › fig 3/i/IMAGES/fsg67/mito MAX_mef 11c11koFGS67 mfn2 myc 647 mitodsRED TOM40_542_thumb_w1Con-mcherry-1.tif (RGB)FDF-1 scal.tif]

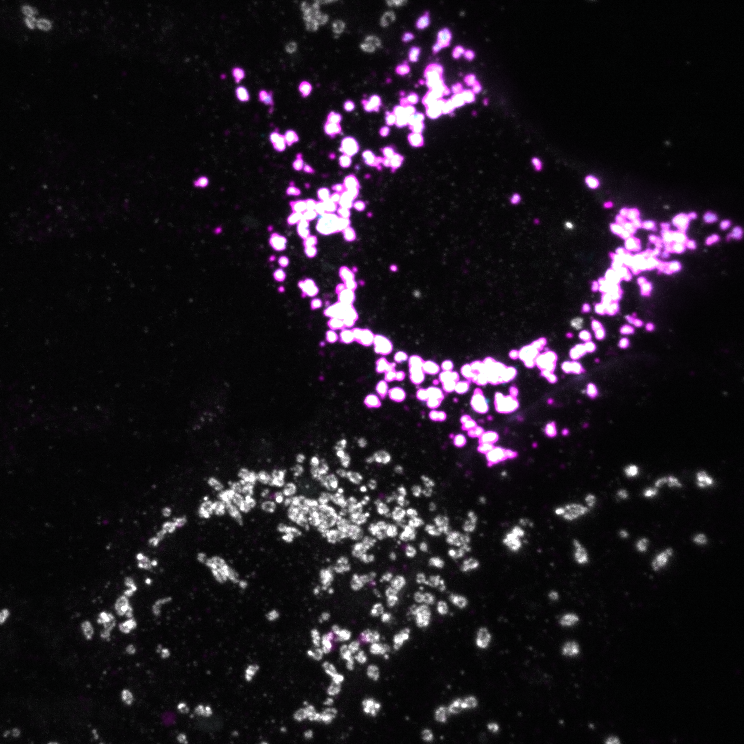

Supplement: Supplementary file 4 — Source Data Fig. 4 [file 44319_2023_9_MOESM4_ESM.zip › fig 3/i/IMAGES/fsg67/mito MAX_mef 11c11koFGS67 mfn2 myc 647 mitodsRED TOM40_542_thumb_w1Con-mcherry-1.tif (RGB)FDF.tif]

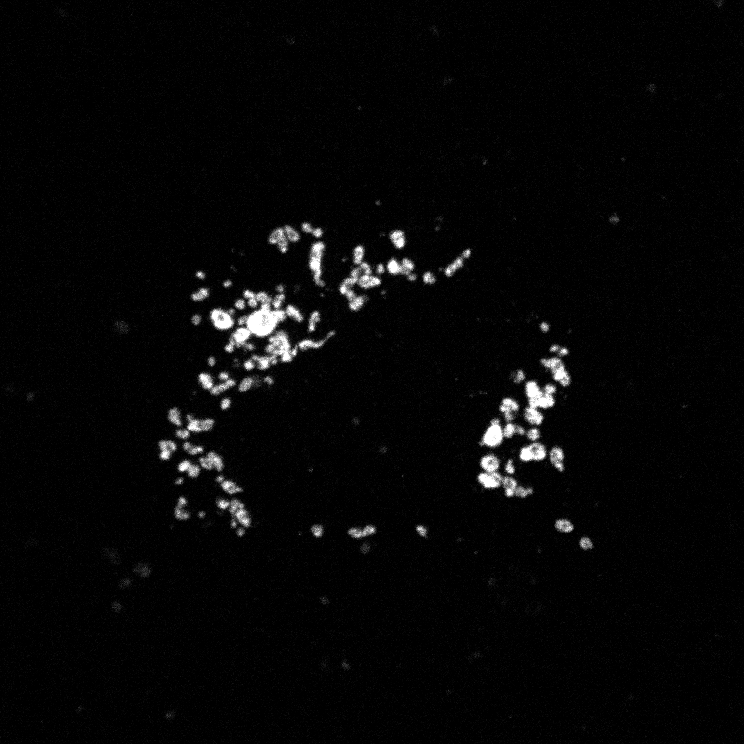

Supplement: Supplementary file 4 — Source Data Fig. 4 [file 44319_2023_9_MOESM4_ESM.zip › fig 3/i/IMAGES/NON TRANSFECTED/MAX_mef 11c11ko control mfn2 myc 647 mitodsRED TOM40_505_thumb_w1Con-mcherry-1FD.tif]

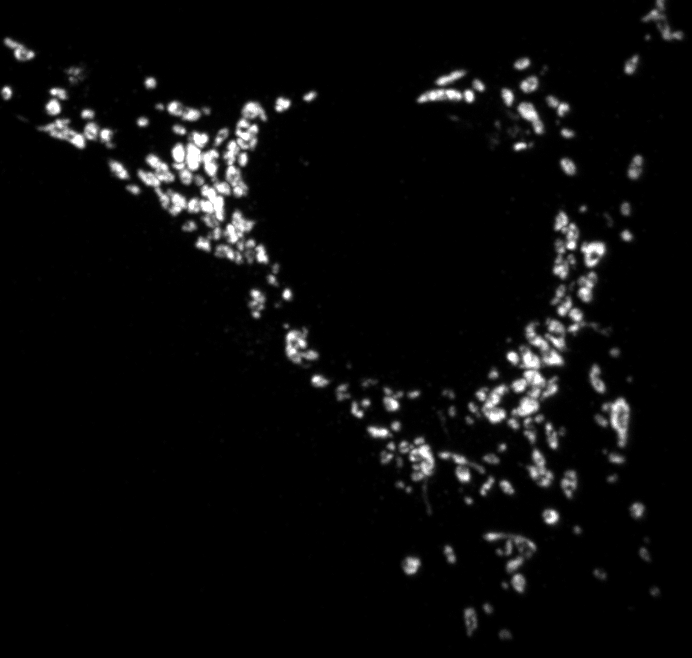

Supplement: Supplementary file 4 — Source Data Fig. 4 [file 44319_2023_9_MOESM4_ESM.zip › fig 3/l/IMAGES/MTCH2 GPAT34si/control/C2-MAX_MEFS MTCH2 KO NTsi T40 598 MRPL675_thumb_w1Con-mcherry_s1.TIF - Stage15-1.tif]

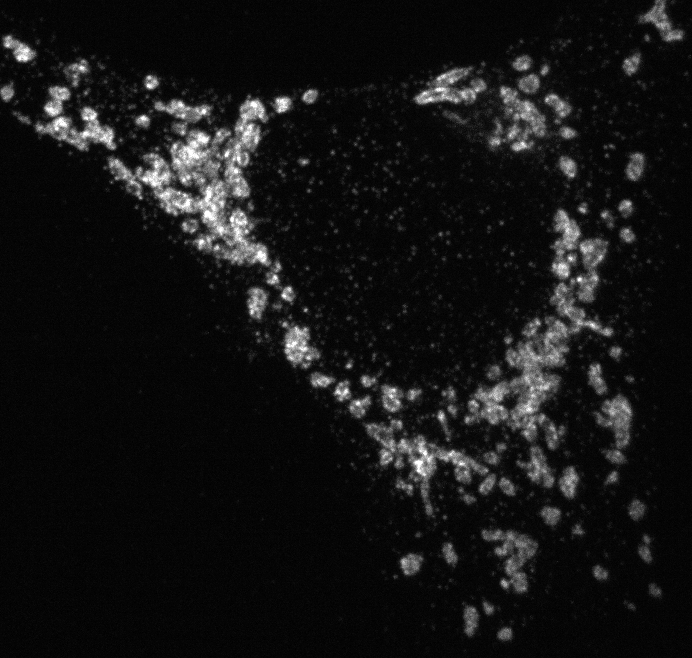

Supplement: Supplementary file 4 — Source Data Fig. 4 [file 44319_2023_9_MOESM4_ESM.zip › fig 3/l/IMAGES/MTCH2 GPAT34si/control/MAX_MEFS MTCH2 KO NTsi T40 598 MRPL675_thumb_w1Con-mcherry_s1.TIF - Stage15-1.tif]

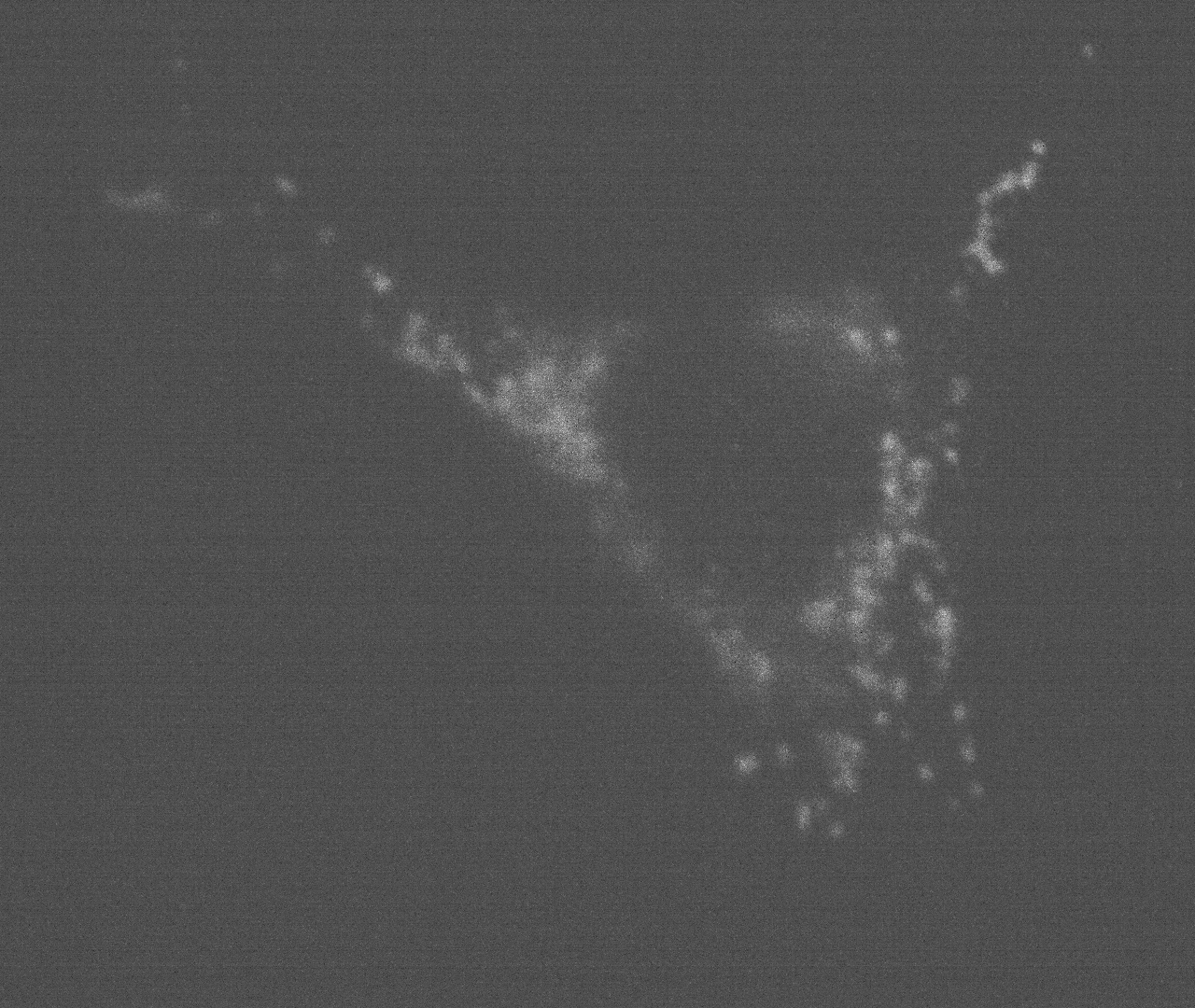

Supplement: Supplementary file 4 — Source Data Fig. 4 [file 44319_2023_9_MOESM4_ESM.zip › fig 3/l/IMAGES/MTCH2 GPAT34si/control/MEFS MTCH2 KO NTsi T40 598 MRPL675_thumb_w1Con-mcherry_s1.TIF - Stage15.tif]

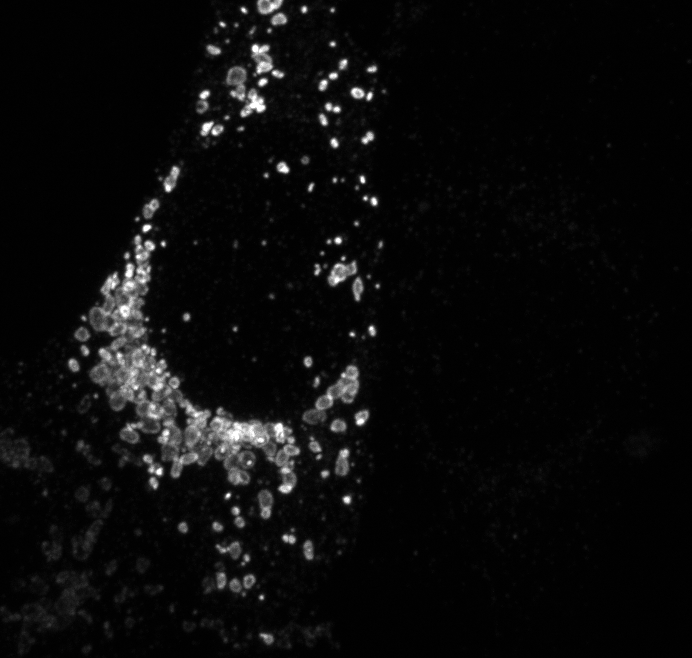

Supplement: Supplementary file 4 — Source Data Fig. 4 [file 44319_2023_9_MOESM4_ESM.zip › fig 3/l/IMAGES/MTCH2 GPAT34si/mfn2/MAX_MEFS MTCH2 KO GPAT3-4si MFN2 598 MRPL641_thumb_w1Con-mcherry-1.tif]

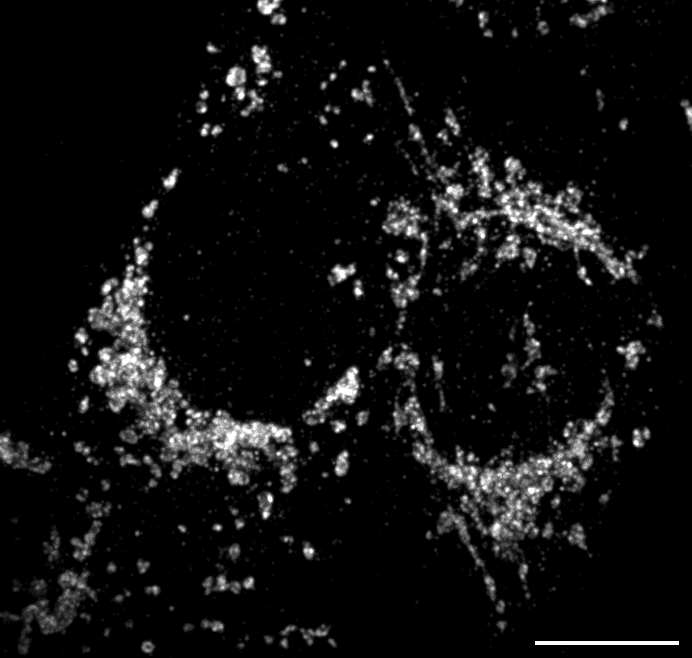

Supplement: Supplementary file 4 — Source Data Fig. 4 [file 44319_2023_9_MOESM4_ESM.zip › fig 3/l/IMAGES/MTCH2 GPAT34si/mfn2/MAX_MEFS MTCH2 KO GPAT3-4si MFN2 598 MRPL641_thumb_w1Con-mcherry-1.tif (RGB) ch1-1 scal.tif]

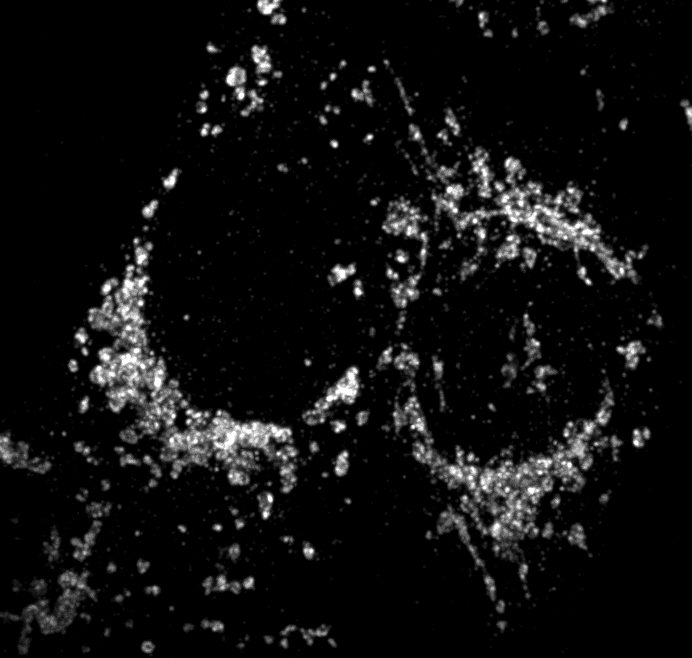

Supplement: Supplementary file 4 — Source Data Fig. 4 [file 44319_2023_9_MOESM4_ESM.zip › fig 3/l/IMAGES/MTCH2 GPAT34si/mfn2/MAX_MEFS MTCH2 KO GPAT3-4si MFN2 598 MRPL641_thumb_w1Con-mcherry-1.tif (RGB) ch1.tif]

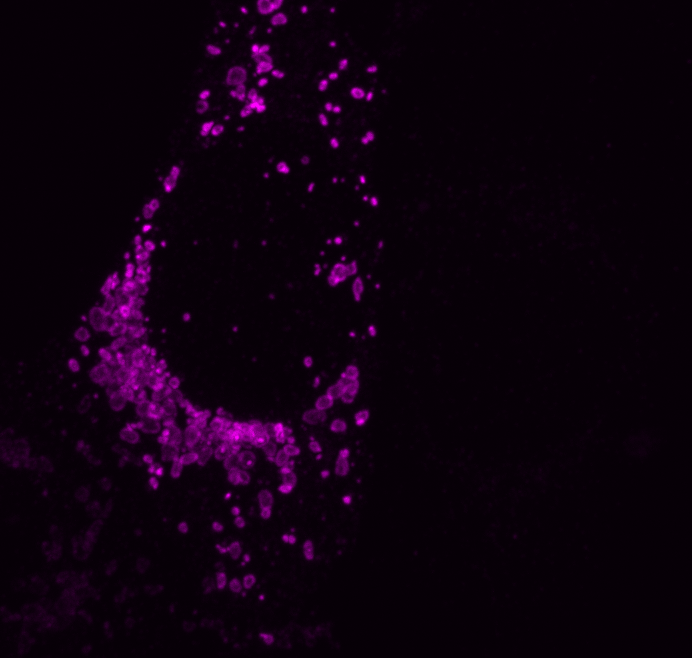

Supplement: Supplementary file 4 — Source Data Fig. 4 [file 44319_2023_9_MOESM4_ESM.zip › fig 3/l/IMAGES/MTCH2 GPAT34si/mfn2/MAX_MEFS MTCH2 KO GPAT3-4si MFN2 598 MRPL641_thumb_w1Con-mcherry-1.tif (RGB) ch2.tif]

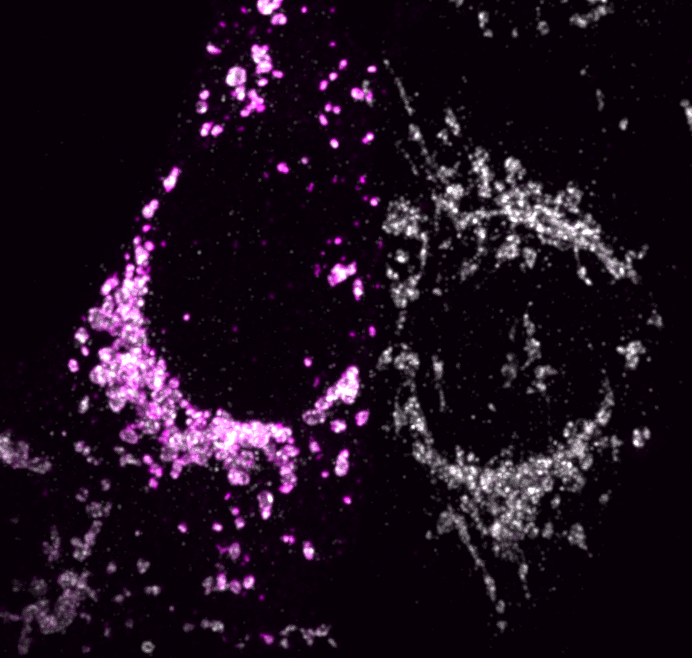

Supplement: Supplementary file 4 — Source Data Fig. 4 [file 44319_2023_9_MOESM4_ESM.zip › fig 3/l/IMAGES/MTCH2 GPAT34si/mfn2/MAX_MEFS MTCH2 KO GPAT3-4si MFN2 598 MRPL641_thumb_w1Con-mcherry-1.tif (RGB) comp.tif]

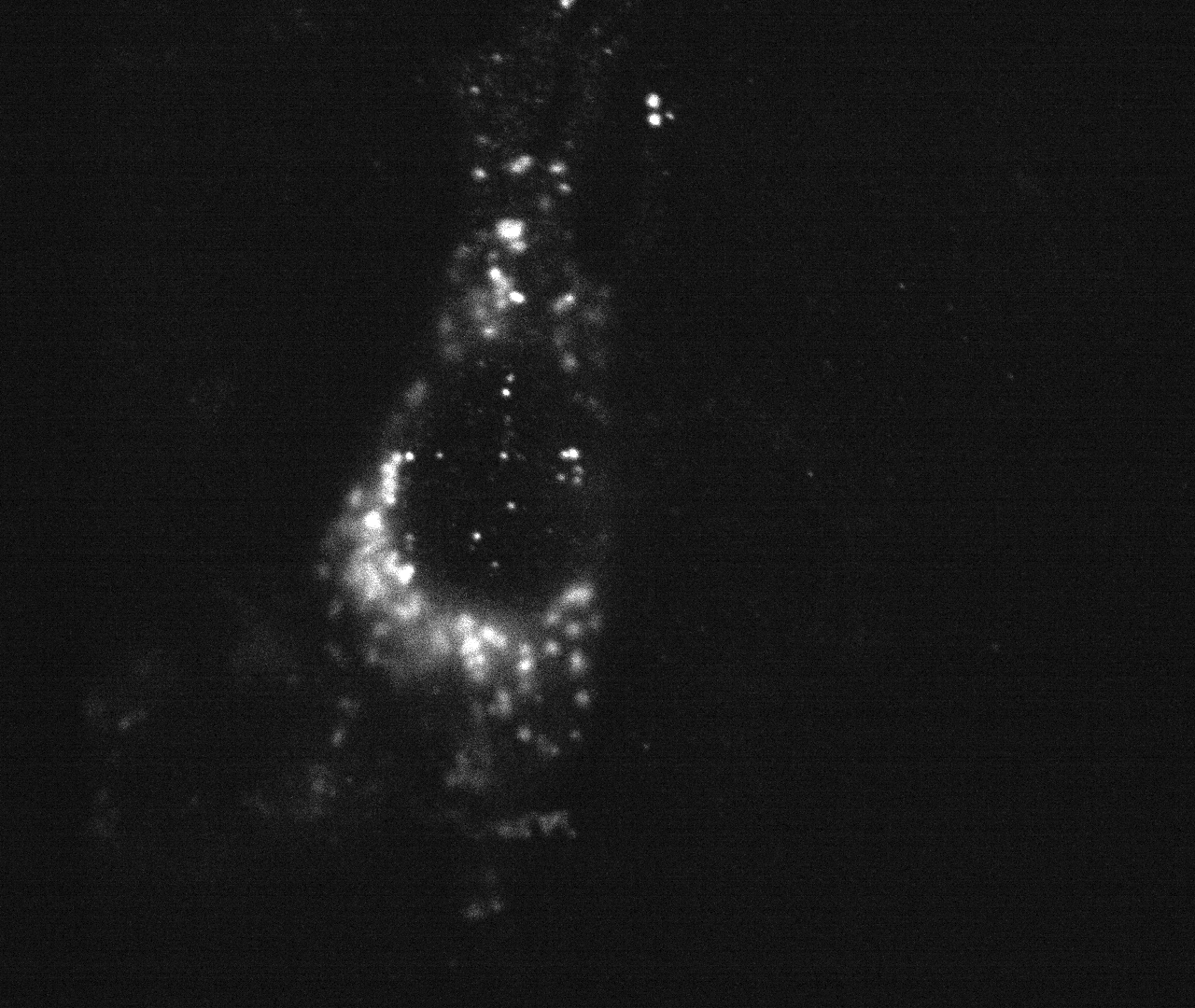

Supplement: Supplementary file 4 — Source Data Fig. 4 [file 44319_2023_9_MOESM4_ESM.zip › fig 3/l/IMAGES/MTCH2 GPAT34si/mfn2/MEFS MTCH2 KO GPAT3-4si MFN2 598 MRPL641_thumb_w1Con-mcherry.tif]

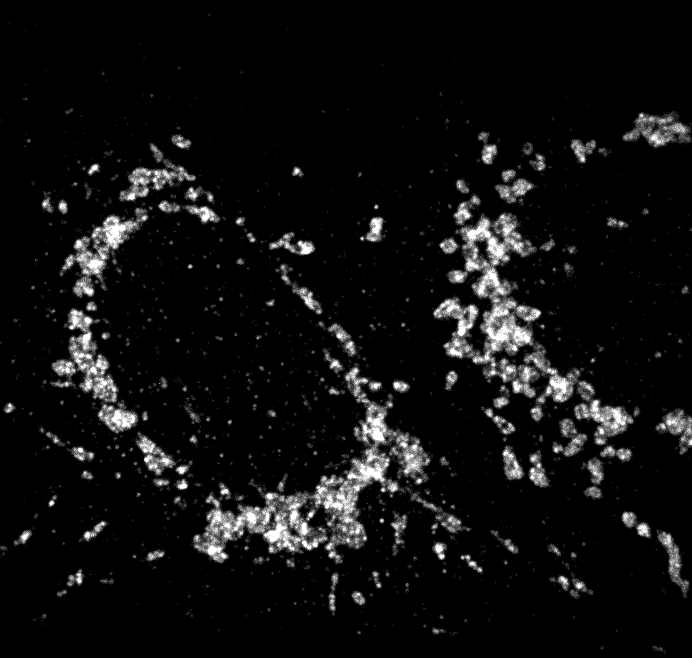

Supplement: Supplementary file 4 — Source Data Fig. 4 [file 44319_2023_9_MOESM4_ESM.zip › fig 3/l/IMAGES/MTCH2 NTsi/control/MAX_MEFS MTCH2 KO NTsi T40 598 MRPL674_thumb_w1Con-mcherry_s1.TIF - Stage6 -1.tif (RGB).tif]

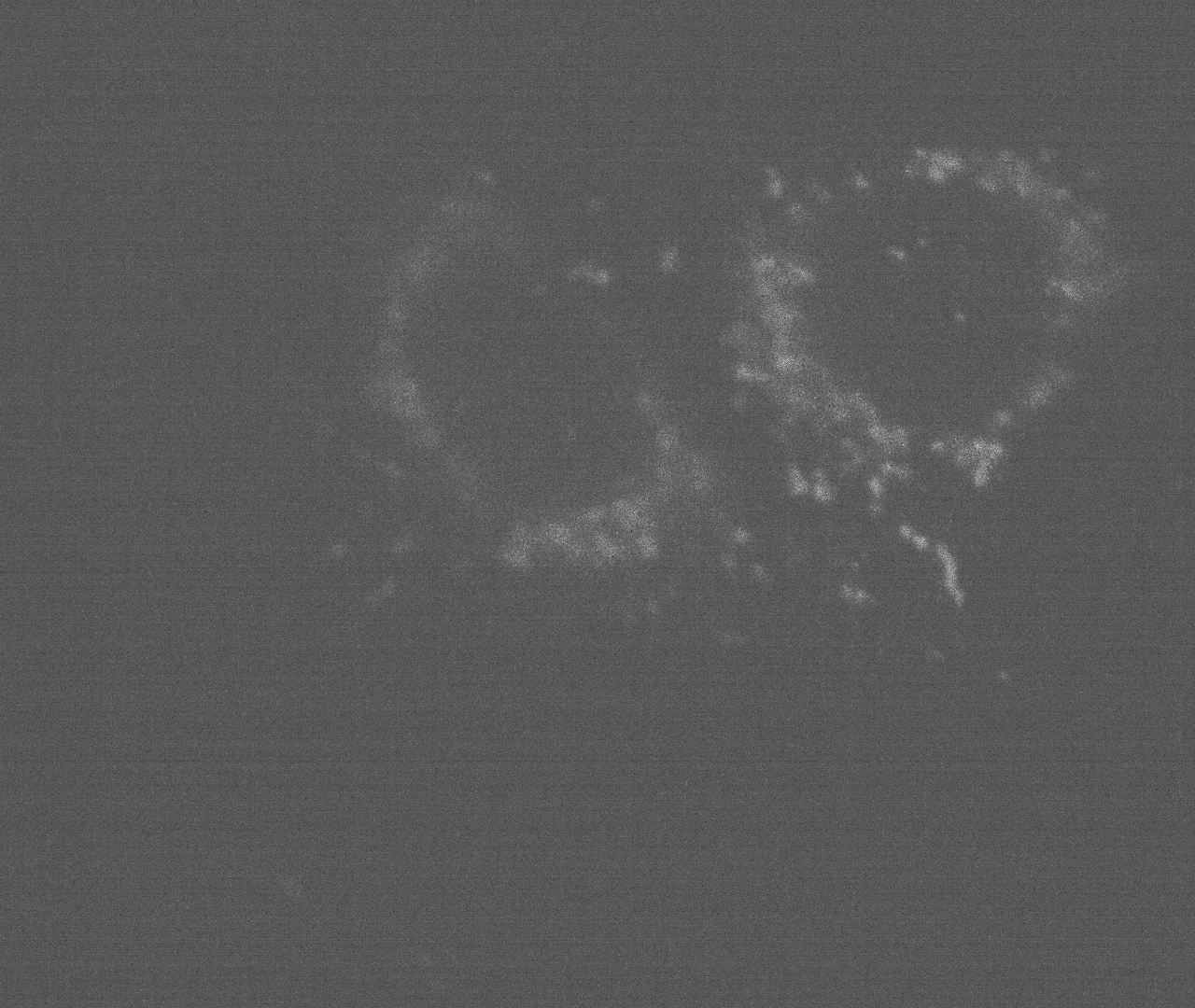

Supplement: Supplementary file 4 — Source Data Fig. 4 [file 44319_2023_9_MOESM4_ESM.zip › fig 3/l/IMAGES/MTCH2 NTsi/control/MEFS MTCH2 KO NTsi T40 598 MRPL674_thumb_w1Con-mcherry_s1.TIF - Stage6 .tif]

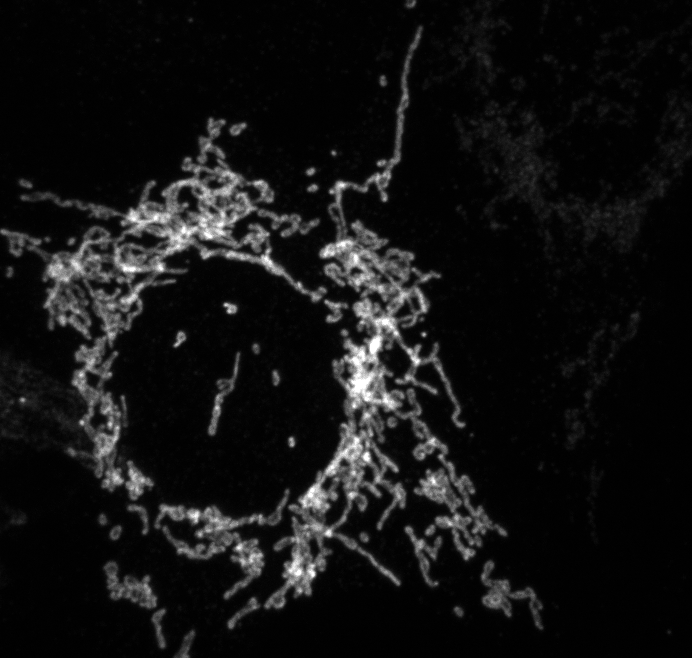

Supplement: Supplementary file 4 — Source Data Fig. 4 [file 44319_2023_9_MOESM4_ESM.zip › fig 3/l/IMAGES/MTCH2 NTsi/mfn2/MAX_MEFS MTCH2 KO NTsi MFN2 598 MRPL664_thumb_w1Con-mcherry-1.tif]

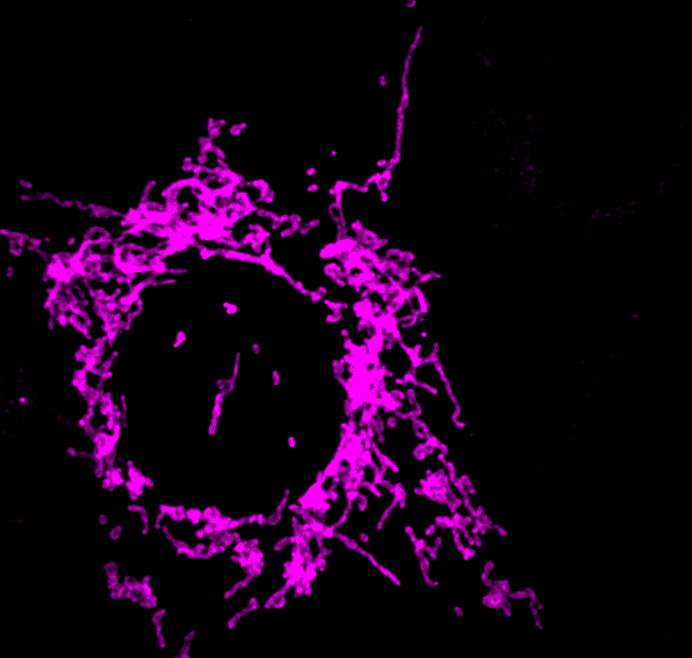

Supplement: Supplementary file 4 — Source Data Fig. 4 [file 44319_2023_9_MOESM4_ESM.zip › fig 3/l/IMAGES/MTCH2 NTsi/mfn2/MAX_MEFS MTCH2 KO NTsi MFN2 598 MRPL664_thumb_w1Con-mcherry-1.tif (RGB ch2.tif]

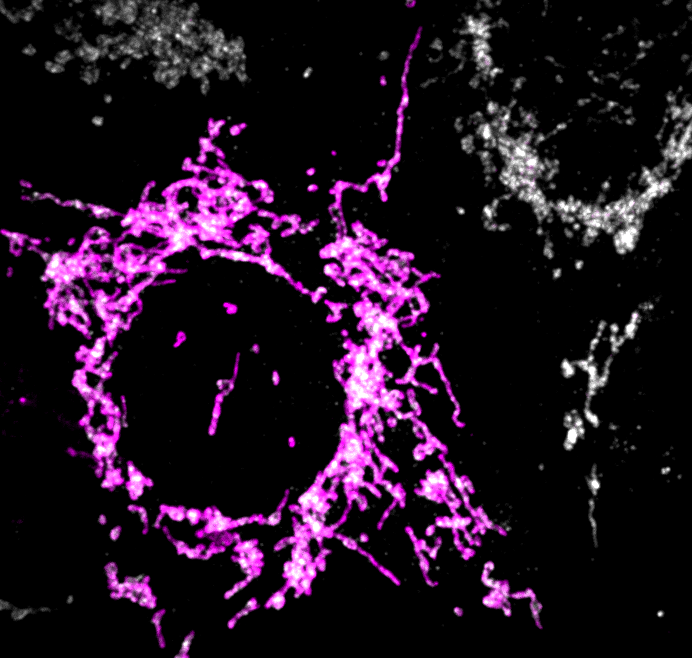

Supplement: Supplementary file 4 — Source Data Fig. 4 [file 44319_2023_9_MOESM4_ESM.zip › fig 3/l/IMAGES/MTCH2 NTsi/mfn2/MAX_MEFS MTCH2 KO NTsi MFN2 598 MRPL664_thumb_w1Con-mcherry-1.tif (RGB) comp.tif]

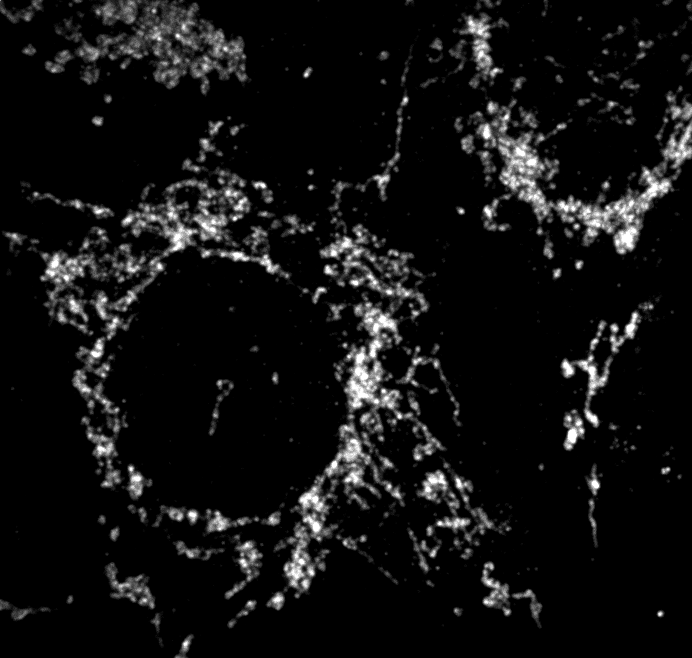

Supplement: Supplementary file 4 — Source Data Fig. 4 [file 44319_2023_9_MOESM4_ESM.zip › fig 3/l/IMAGES/MTCH2 NTsi/mfn2/MAX_MEFS MTCH2 KO NTsi MFN2 598 MRPL664_thumb_w1Con-mcherry-1.tif (RGB)ch1.tif]

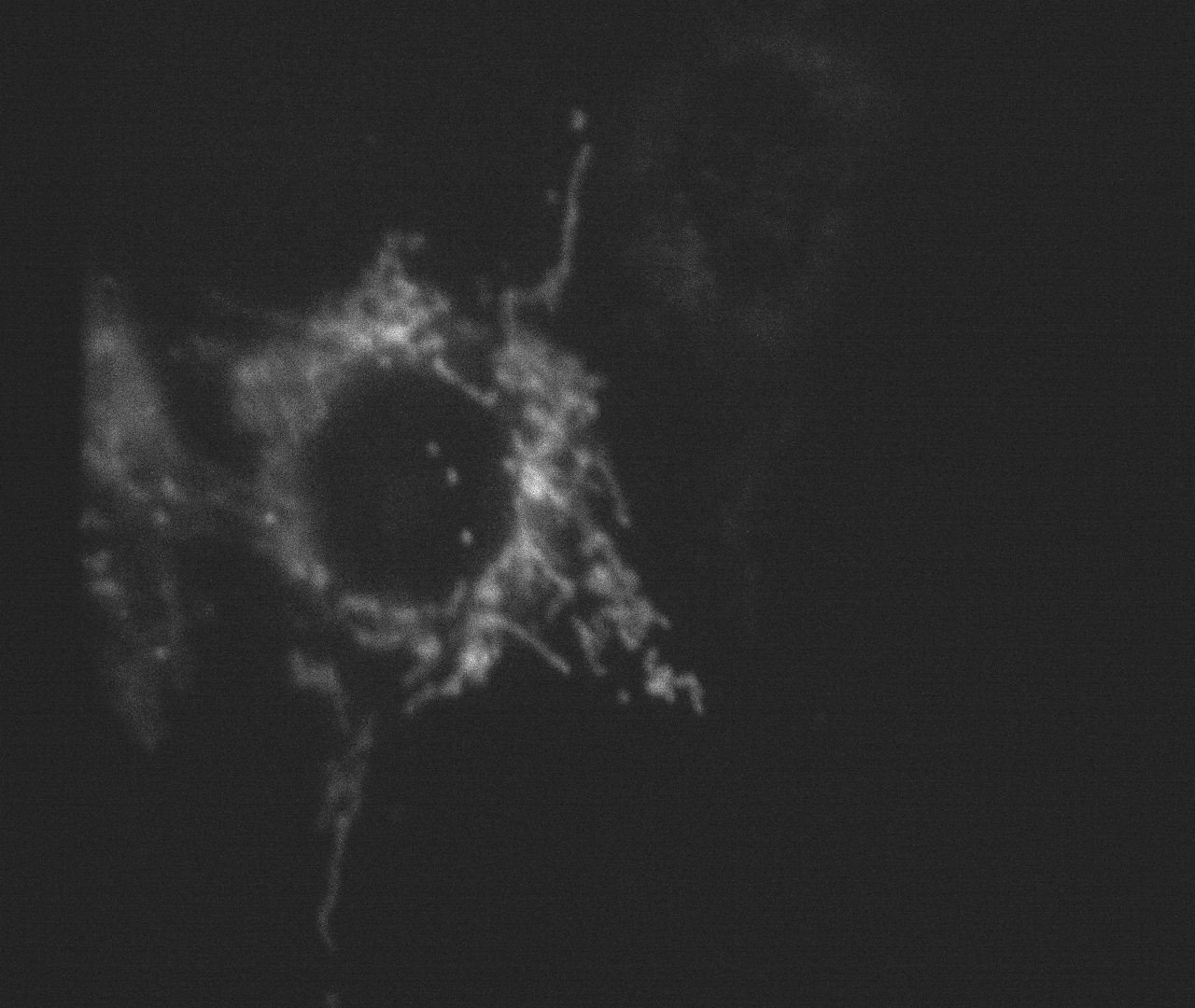

Supplement: Supplementary file 4 — Source Data Fig. 4 [file 44319_2023_9_MOESM4_ESM.zip › fig 3/l/IMAGES/MTCH2 NTsi/mfn2/MEFS MTCH2 KO NTsi MFN2 598 MRPL664_thumb_w1Con-mcherry.tif]

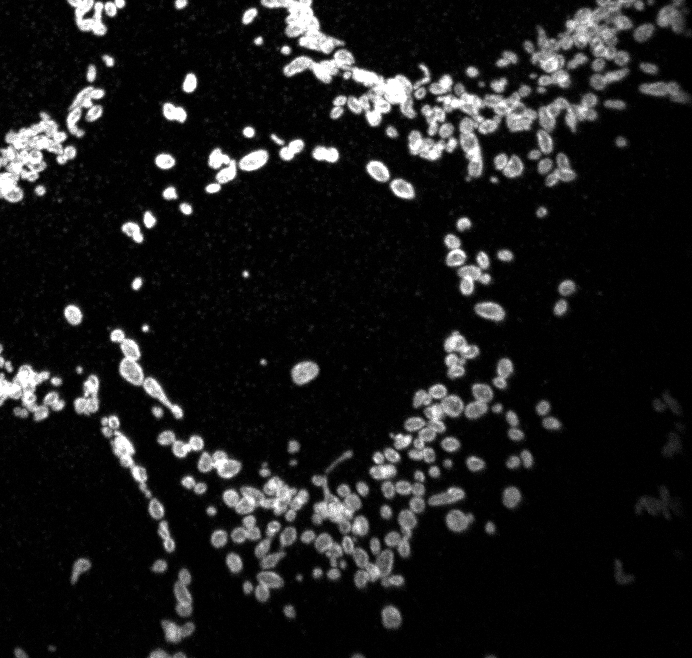

Supplement: Supplementary file 5 — Source Data EV Fig. 1 [file 44319_2023_9_MOESM5_ESM.zip › fig 4/a/IMAGES/CONTROL/MAX_MEFs MFN2 KO control tom598 cytc 633 dapi3_thumb_w1Con-mcherry_s1.TIF - Stage25-1.tif (RGB).tif]

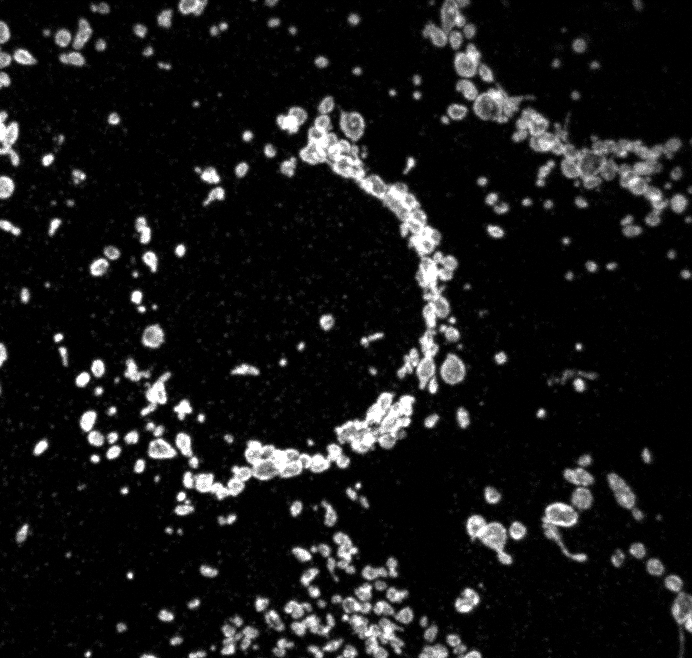

Supplement: Supplementary file 5 — Source Data EV Fig. 1 [file 44319_2023_9_MOESM5_ESM.zip › fig 4/a/IMAGES/CONTROL/MAX_MEFs MTCH2 KO control tom598 cytc 633 dapi3_thumb_w1Con-mcherry_s1.TIF - Stage26-1.tif (RGB).tif]

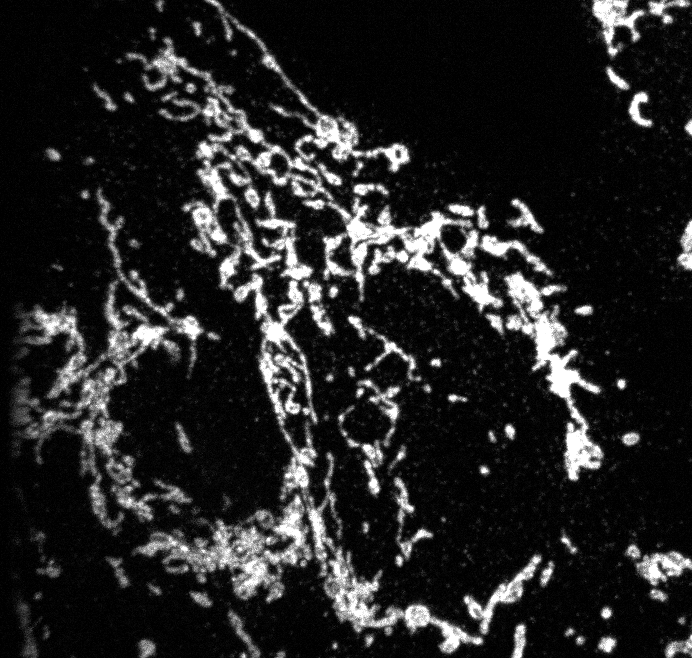

Supplement: Supplementary file 5 — Source Data EV Fig. 1 [file 44319_2023_9_MOESM5_ESM.zip › fig 4/a/IMAGES/CONTROL/MAX_MEFs WT control tom598 cytc 633 dapi3_thumb_w1Con-mcherry_s1.TIF - Stage5-1.tif (RGB).tif]

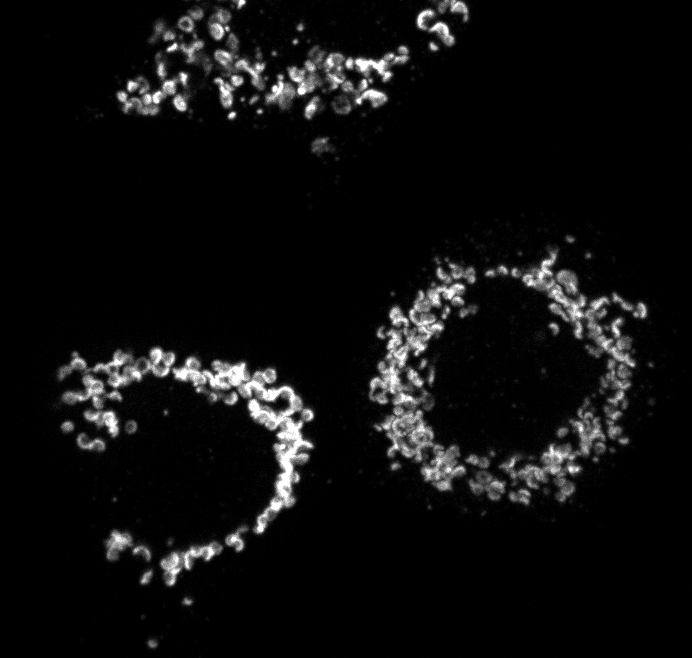

Supplement: Supplementary file 5 — Source Data EV Fig. 1 [file 44319_2023_9_MOESM5_ESM.zip › fig 4/a/IMAGES/GPATi+HBSS/MAX_mefs mfn2 ko FSG67 HBSS tom598 cytc 633 dapi3_thumb_w1Con-mcherry_s1.TIF - Stage15-1.tif (RGB).tif]

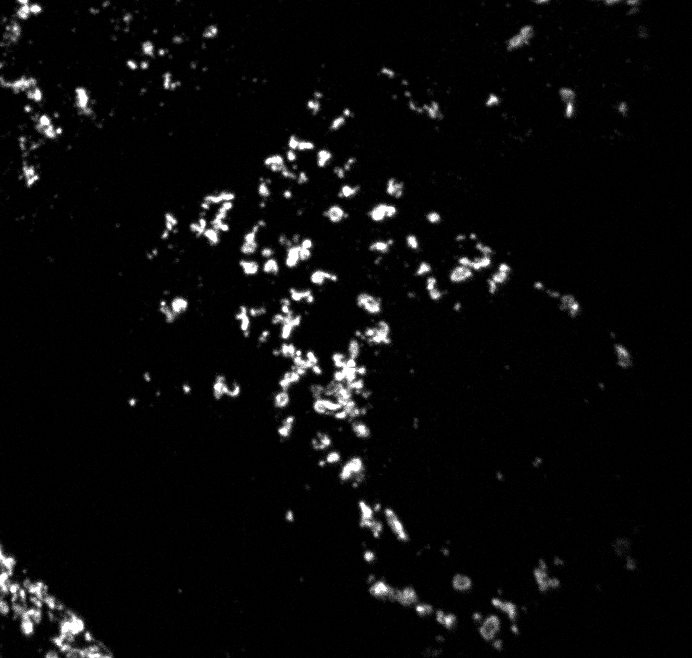

Supplement: Supplementary file 5 — Source Data EV Fig. 1 [file 44319_2023_9_MOESM5_ESM.zip › fig 4/a/IMAGES/GPATi+HBSS/MAX_mefs mtch2 ko FSG67 HBSS tom598 cytc 633 dapi5_thumb_w1Con-mcherry_s1.TIF - Stage10-1.tif (RGB).tif]

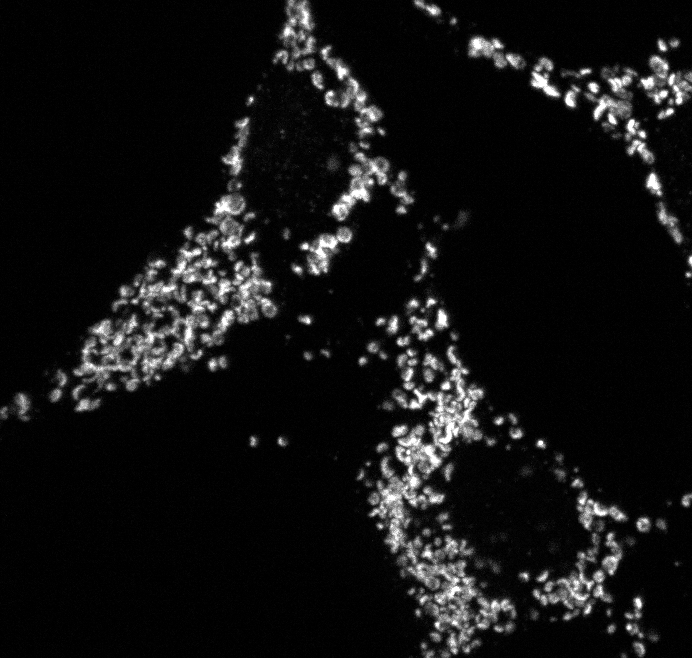

Supplement: Supplementary file 5 — Source Data EV Fig. 1 [file 44319_2023_9_MOESM5_ESM.zip › fig 4/a/IMAGES/GPATi+HBSS/MAX_mefs WT FSG67 HBSS tom598 cytc 633 dapi1_thumb_w1Con-mcherry_s1.TIF - Stage13-1.tif (RGB).tif]

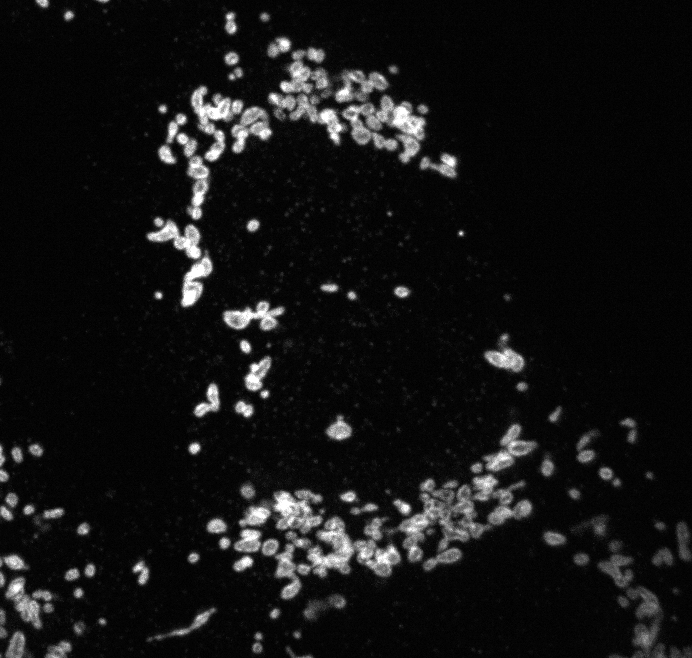

Supplement: Supplementary file 5 — Source Data EV Fig. 1 [file 44319_2023_9_MOESM5_ESM.zip › fig 4/a/IMAGES/GPATi/MAX_mefs mfn2 ko fsg64 tom 598 cytc633 dapi im6_thumb_w1Con-mcherry_s1.TIF - Stage18-1.tif (RGB).tif]

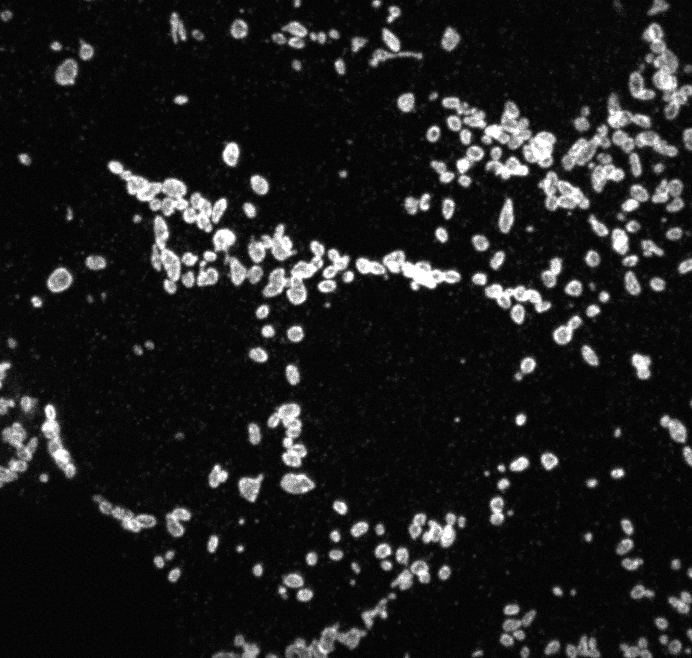

Supplement: Supplementary file 5 — Source Data EV Fig. 1 [file 44319_2023_9_MOESM5_ESM.zip › fig 4/a/IMAGES/GPATi/MAX_mefs mtch2 ko fsg64 tom 598 cytc633 dapi im6_thumb_w1Con-mcherry_s1.TIF - Stage29-1.tif (RGB).tif]

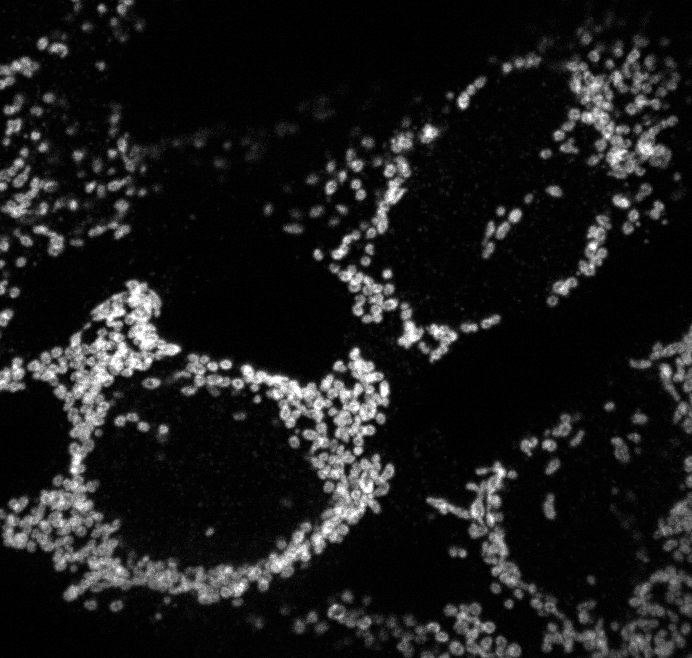

Supplement: Supplementary file 5 — Source Data EV Fig. 1 [file 44319_2023_9_MOESM5_ESM.zip › fig 4/a/IMAGES/GPATi/MAX_mefs wt fsg64 tom 598 cytc633 dapi im6_thumb_w1Con-mcherry_s1.TIF - Stage5 -1.tif (RGB).tif]

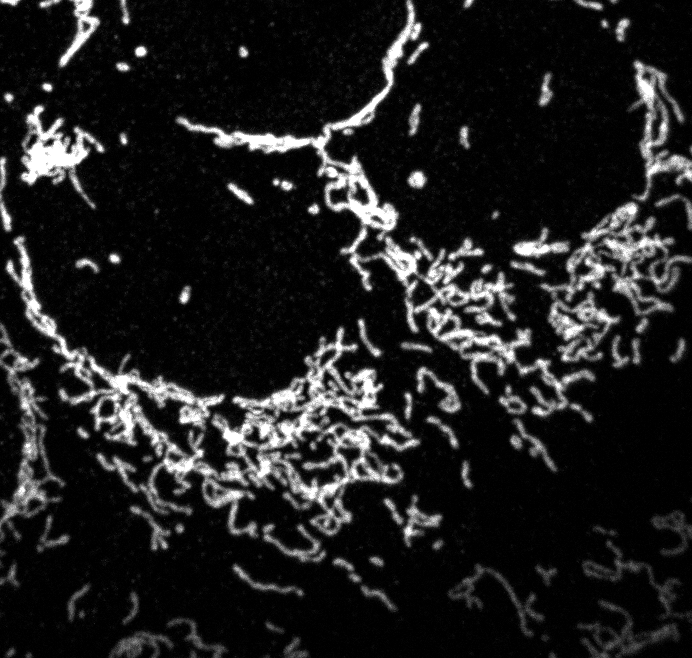

Supplement: Supplementary file 5 — Source Data EV Fig. 1 [file 44319_2023_9_MOESM5_ESM.zip › fig 4/a/IMAGES/HBSS/MAX_MEFs mfn2 ko HBSS tom598 cytc 633 dapi3_thumb_w1Con-mcherry_s1.TIF - Stage32 -1.tif (RGB).tif]

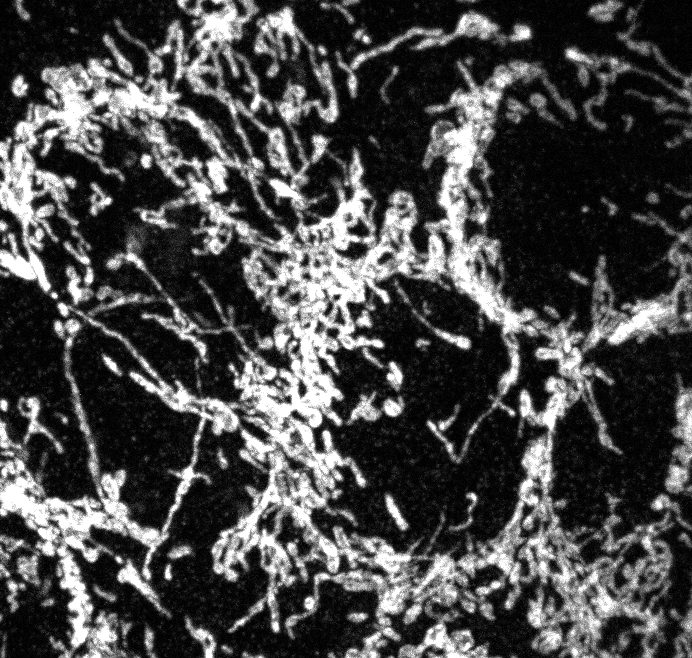

Supplement: Supplementary file 5 — Source Data EV Fig. 1 [file 44319_2023_9_MOESM5_ESM.zip › fig 4/a/IMAGES/HBSS/MAX_MEFs mtch2 ko HBSS tom598 cytc 633 dapi3_thumb_w1Con-mcherry_s1.TIF - Stage6 -1.tif (RGB).tif]

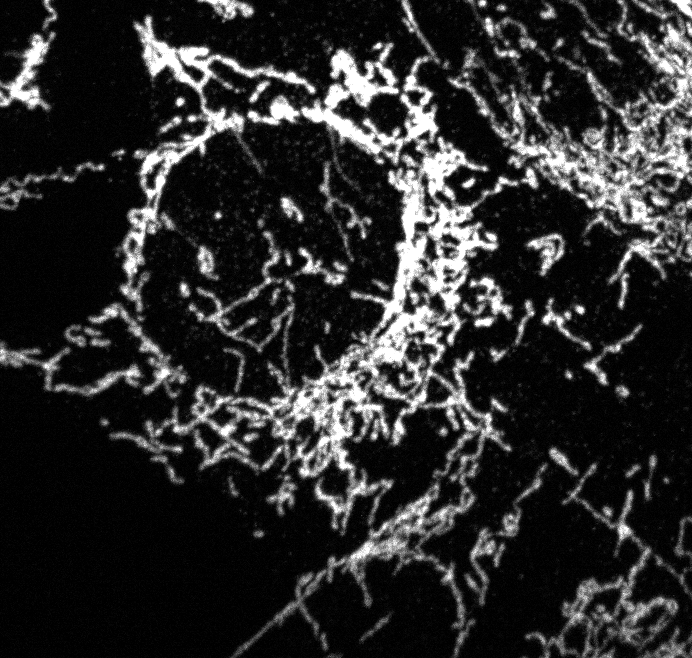

Supplement: Supplementary file 5 — Source Data EV Fig. 1 [file 44319_2023_9_MOESM5_ESM.zip › fig 4/a/IMAGES/HBSS/MAX_MEFs WT HBSS tom598 cytc 633 dapi_thumb_w1Con-mcherry_s1.TIF - Stage19-1.tif (RGB).tif]

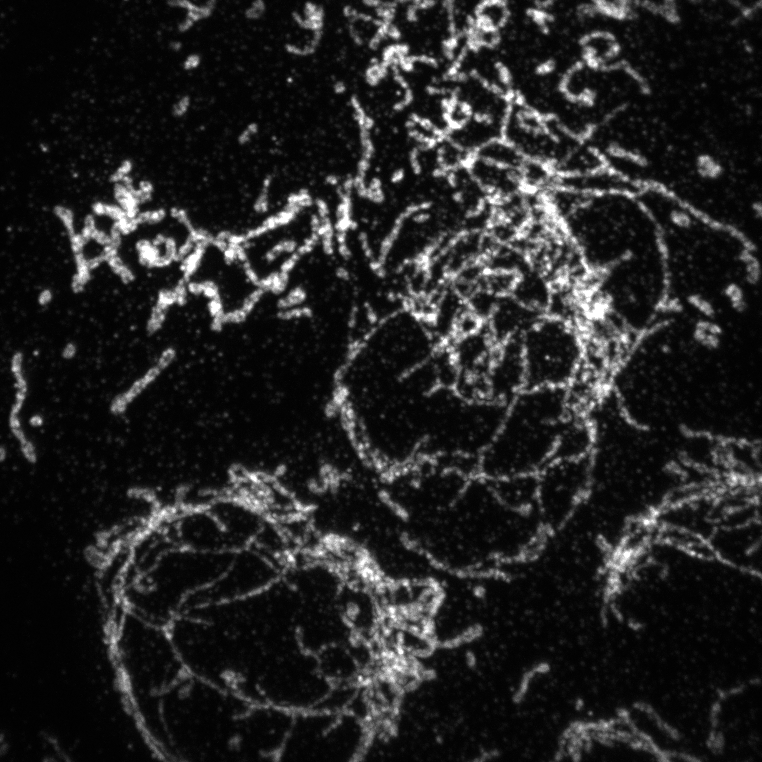

Supplement: Supplementary file 5 — Source Data EV Fig. 1 [file 44319_2023_9_MOESM5_ESM.zip › fig 4/d/IMAGES/CHX/MFN2 KO/MAX_mfn2 ko chx mito ds red tom40 cy123_thumb_w1Con-Cy5-13.tif]

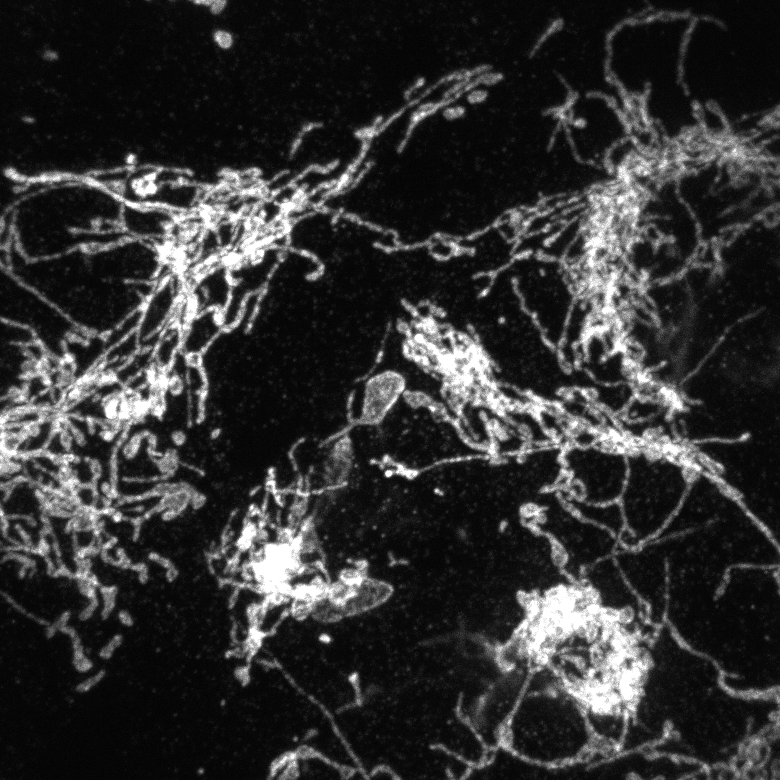

Supplement: Supplementary file 5 — Source Data EV Fig. 1 [file 44319_2023_9_MOESM5_ESM.zip › fig 4/d/IMAGES/CHX/MTCH2 KO/mitomef 11c11 ko chx3_thumb_w1Con-mcherry-1SS.tif]

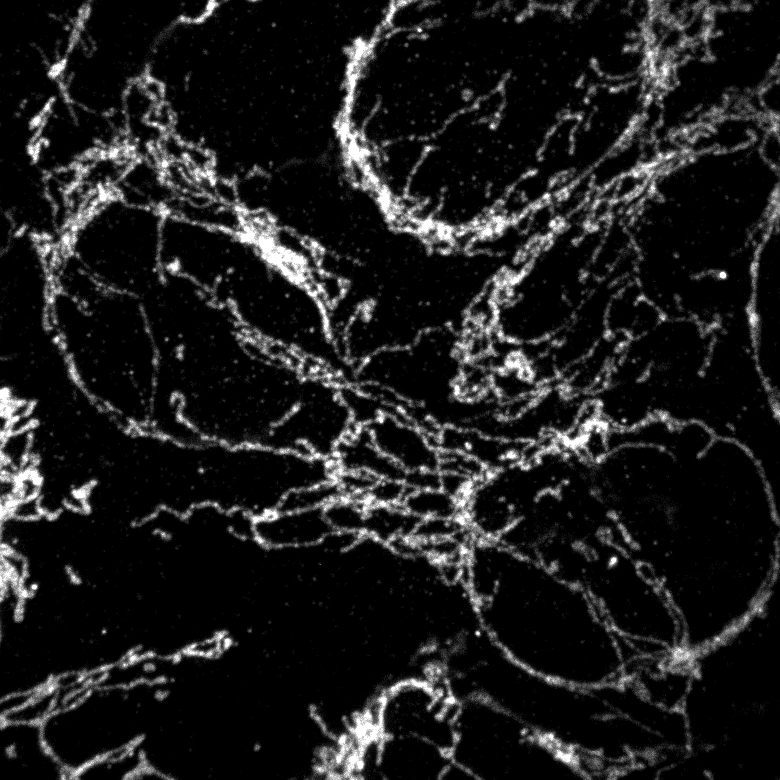

Supplement: Supplementary file 5 — Source Data EV Fig. 1 [file 44319_2023_9_MOESM5_ESM.zip › fig 4/d/IMAGES/CHX/WT/mitomef ff chx4_thumb_w1Con-mcherry-1SS.tif]

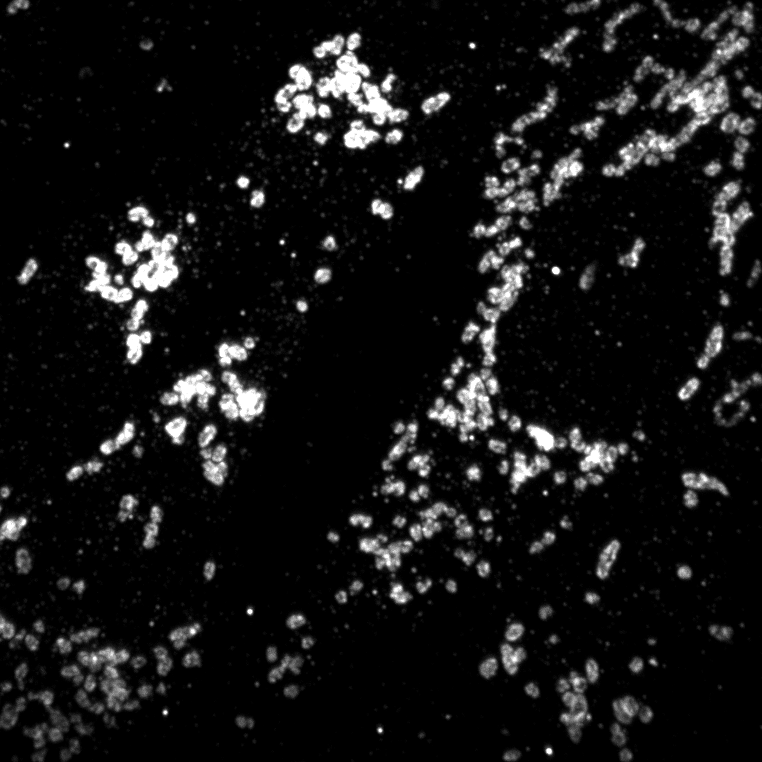

Supplement: Supplementary file 5 — Source Data EV Fig. 1 [file 44319_2023_9_MOESM5_ESM.zip › fig 4/d/IMAGES/CONTROL/MFN2 KO/MAX_mfn2 kocontrol mito ds red tom40 cy68_thumb_w1Con-Cy5-13.tif]

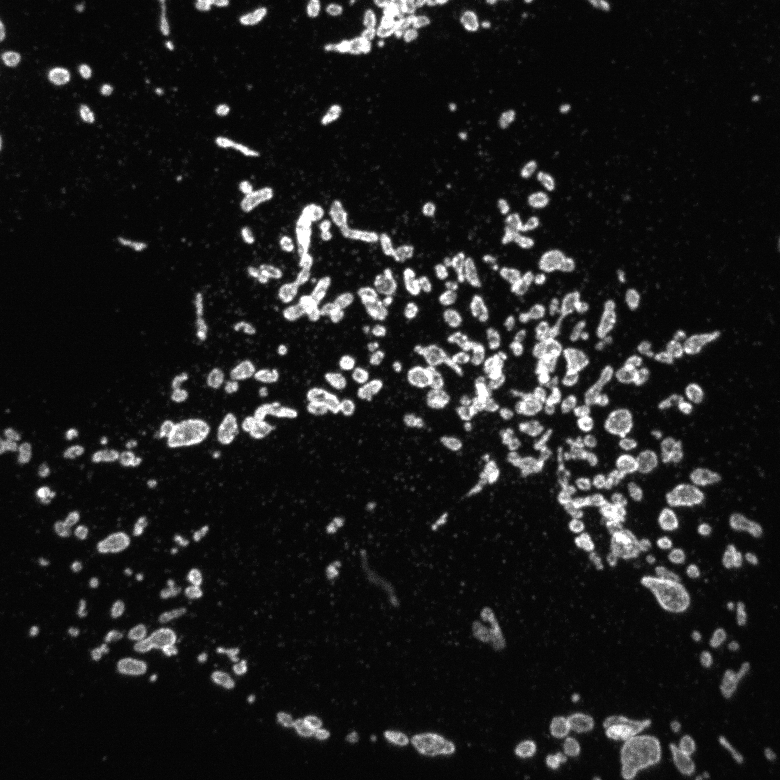

Supplement: Supplementary file 5 — Source Data EV Fig. 1 [file 44319_2023_9_MOESM5_ESM.zip › fig 4/d/IMAGES/CONTROL/MTCH2 KO/mitomef 11c11 ko control5_thumb_w1Con-mcherry-1SS.tif]

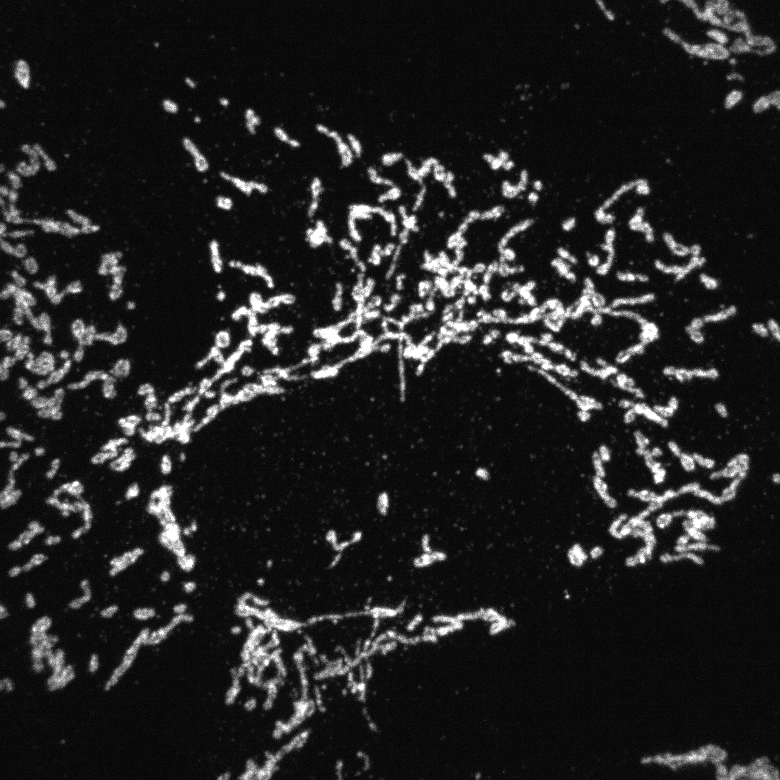

Supplement: Supplementary file 5 — Source Data EV Fig. 1 [file 44319_2023_9_MOESM5_ESM.zip › fig 4/d/IMAGES/CONTROL/WT/mitomef FF control5_thumb_w1Con-mcherrySS.tif]

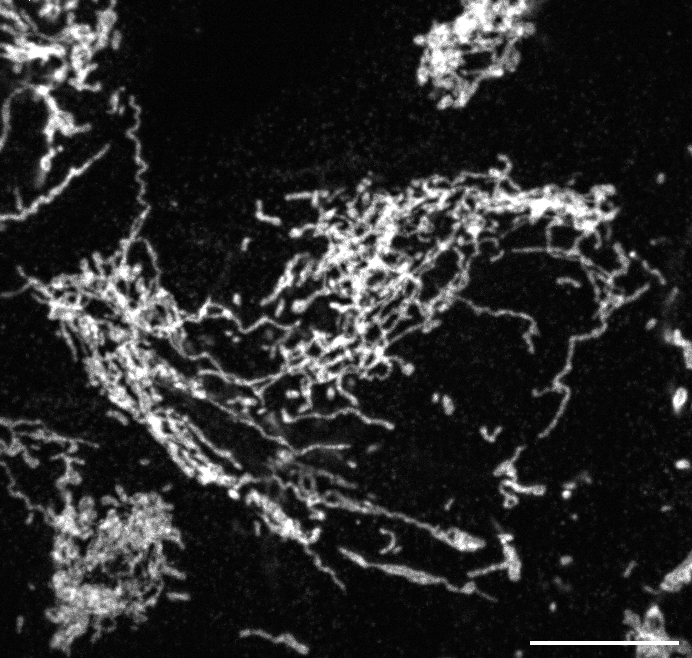

Supplement: Supplementary file 5 — Source Data EV Fig. 1 [file 44319_2023_9_MOESM5_ESM.zip › fig 4/d/IMAGES/GPATi + CHX/MFN2 KO/MAX_mefs mfn2 ko fsg64 chx tom 598 cytc633 dapi im2_thumb_w1Con-mcherry_s1.TIF - Stage5-1.tif (RGB)-3scale.tif]

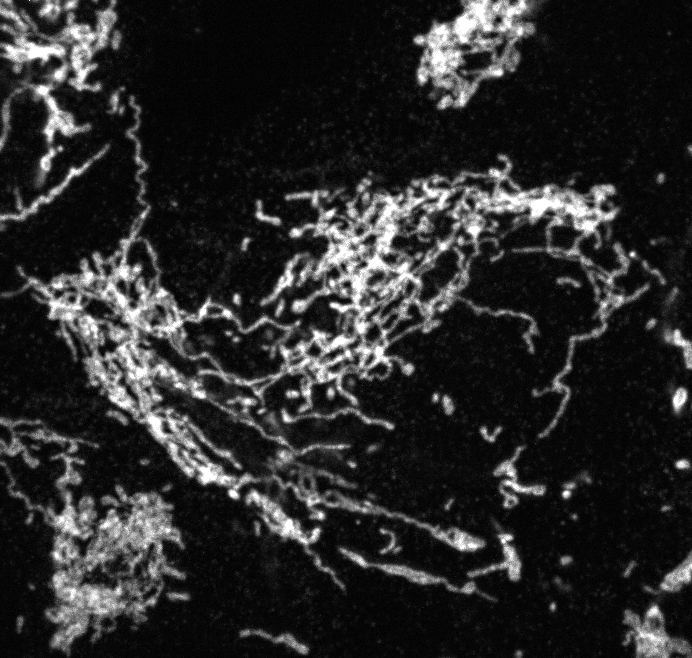

Supplement: Supplementary file 5 — Source Data EV Fig. 1 [file 44319_2023_9_MOESM5_ESM.zip › fig 4/d/IMAGES/GPATi + CHX/MFN2 KO/MAX_mefs mfn2 ko fsg64 chx tom 598 cytc633 dapi im2_thumb_w1Con-mcherry_s1.TIF - Stage5-1.tif (RGB).tif]

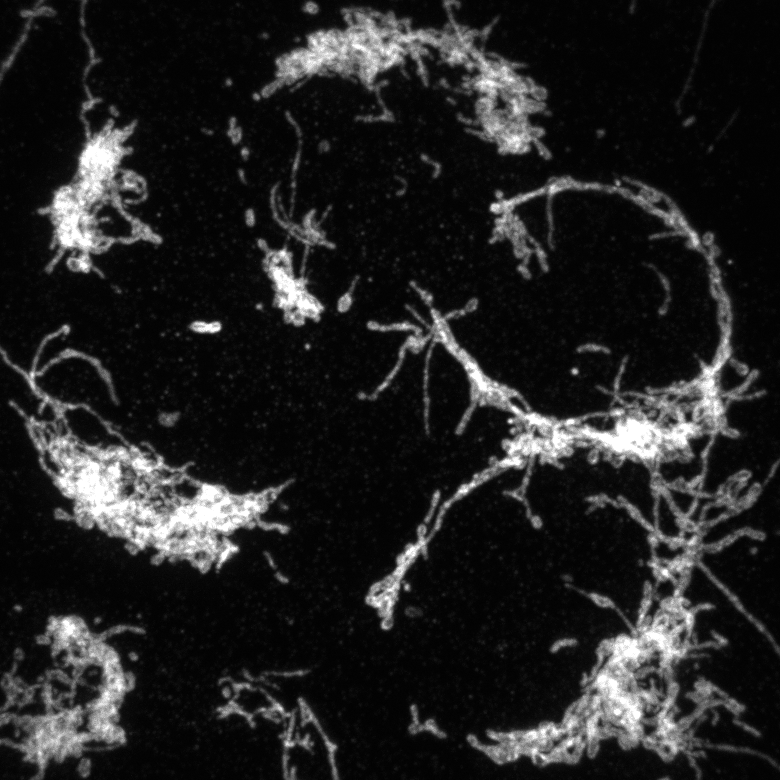

Supplement: Supplementary file 5 — Source Data EV Fig. 1 [file 44319_2023_9_MOESM5_ESM.zip › fig 4/d/IMAGES/GPATi + CHX/MTCH2 KO/mitomef 11c11 mtch2 ko fgs67---CHX 28_thumb_w1Con-mcherry-1SS.tif]

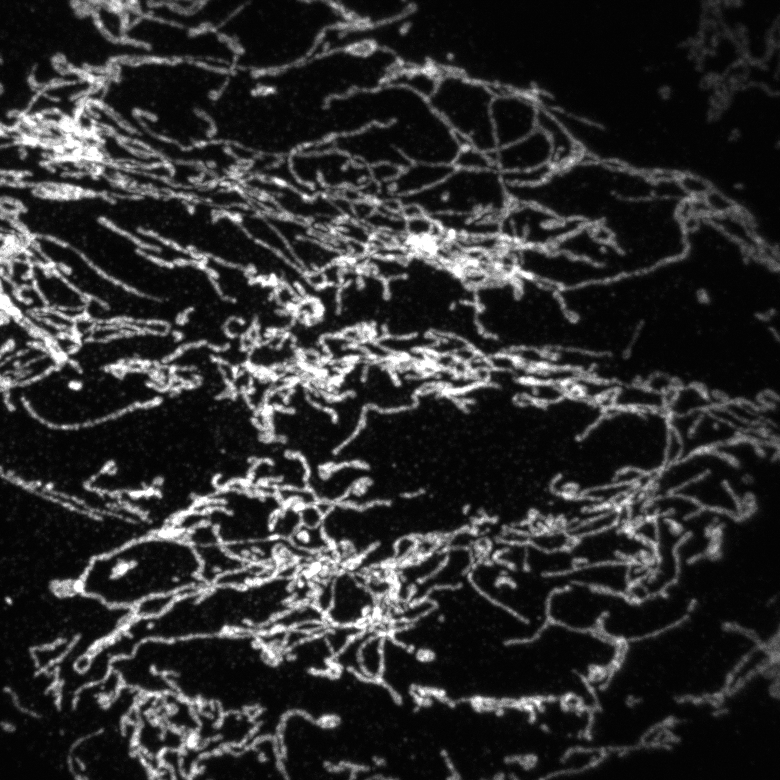

Supplement: Supplementary file 5 — Source Data EV Fig. 1 [file 44319_2023_9_MOESM5_ESM.zip › fig 4/d/IMAGES/GPATi + CHX/WT/mitomef FF fgs67---CHX 10_thumb_w1Con-mcherry-1SS.tif]

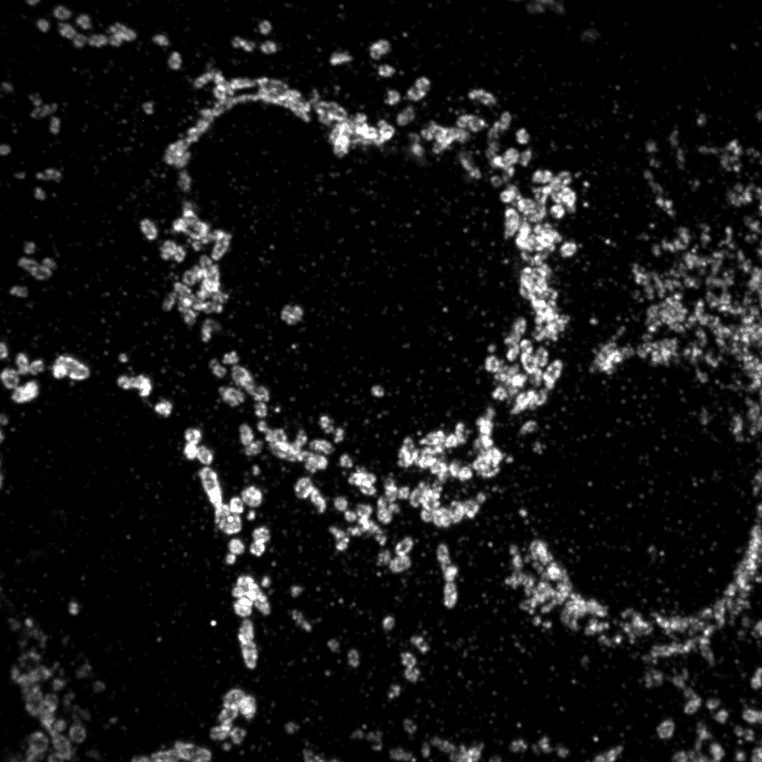

Supplement: Supplementary file 5 — Source Data EV Fig. 1 [file 44319_2023_9_MOESM5_ESM.zip › fig 4/d/IMAGES/GPATi/MFN2 KO/MAX_mfn2 ko fsg67 mito ds red tom40 cy86_thumb_w1Con-Cy5-13.tif]

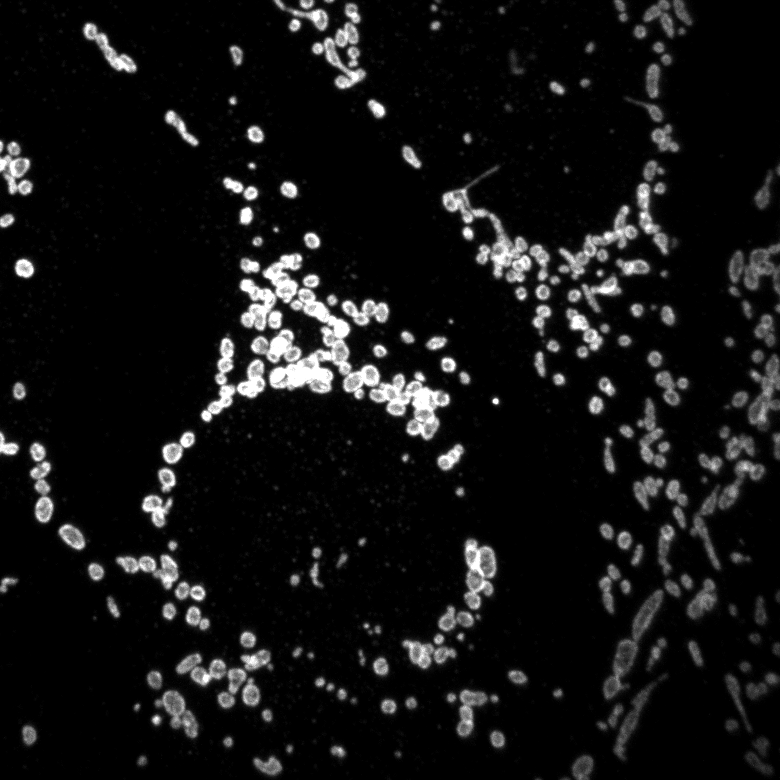

Supplement: Supplementary file 5 — Source Data EV Fig. 1 [file 44319_2023_9_MOESM5_ESM.zip › fig 4/d/IMAGES/GPATi/MTCH2 KO/mitomef 11c11 FGS8_thumb_w1Con-mcherry-1SS.tif]

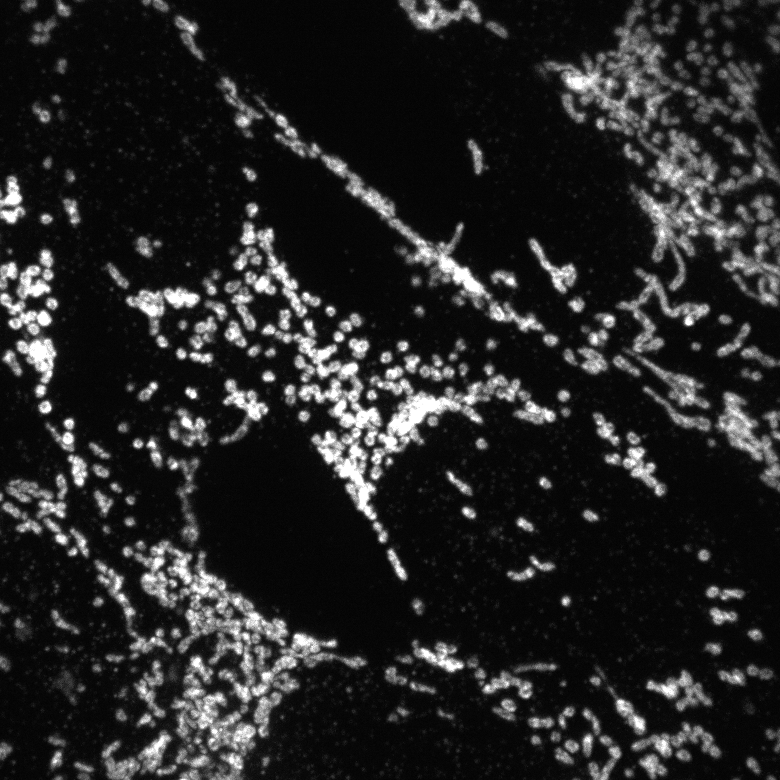

Supplement: Supplementary file 5 — Source Data EV Fig. 1 [file 44319_2023_9_MOESM5_ESM.zip › fig 4/d/IMAGES/GPATi/WT/mitomef ff FGS79_thumb_w1Con-mcherry-1SS.tif]

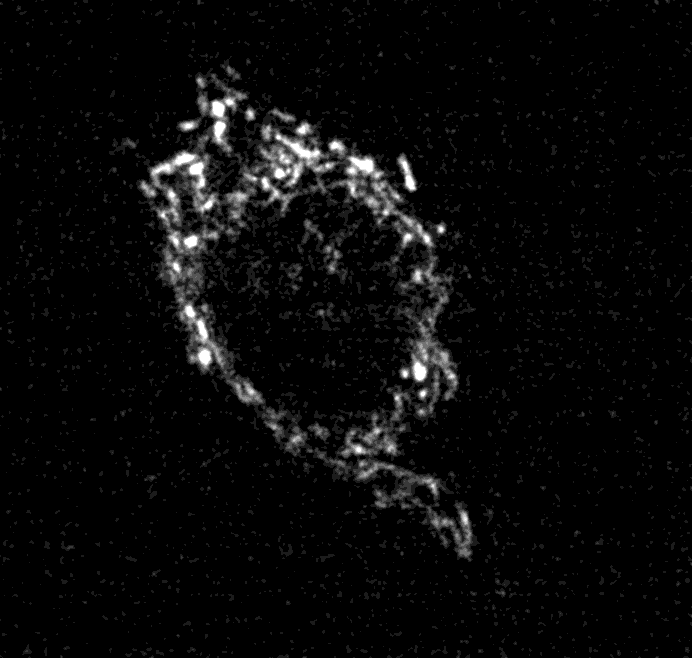

Supplement: Supplementary file 5 — Source Data EV Fig. 1 [file 44319_2023_9_MOESM5_ESM.zip › fig 4/g/IMAGES/chx/MAX_mefs MTCH2 KO CHX dsRED mito-PAGFP15_thumb_w1Con-mcherry_t1-1-1 OPT3.tif]

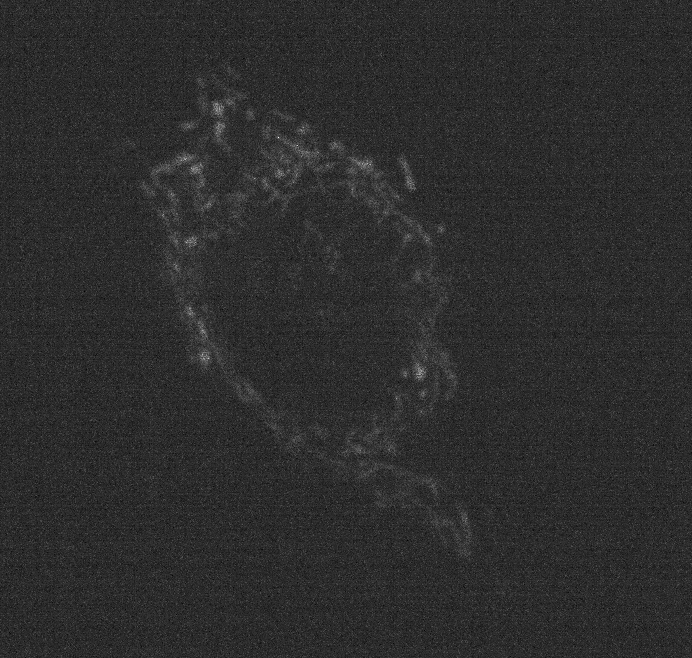

Supplement: Supplementary file 5 — Source Data EV Fig. 1 [file 44319_2023_9_MOESM5_ESM.zip › fig 4/g/IMAGES/chx/MAX_mefs MTCH2 KO CHX dsRED mito-PAGFP15_thumb_w1Con-mcherry_t1-1.tif]

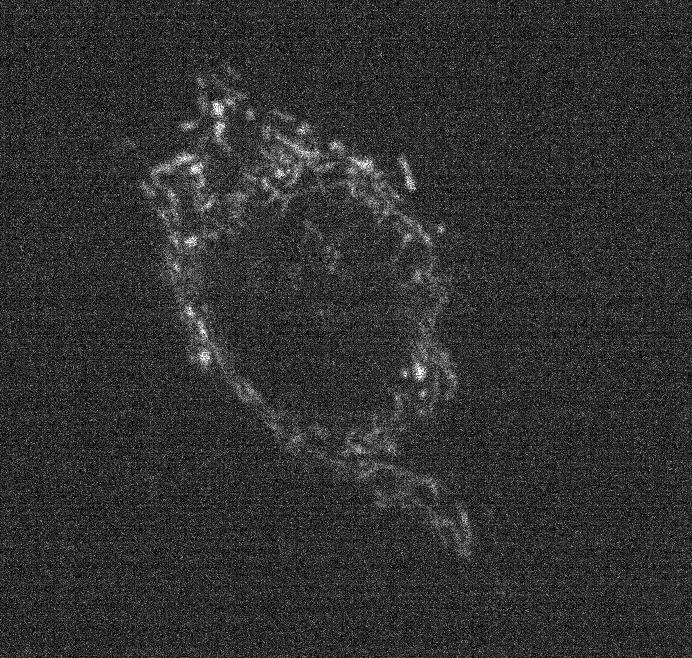

Supplement: Supplementary file 5 — Source Data EV Fig. 1 [file 44319_2023_9_MOESM5_ESM.zip › fig 4/g/IMAGES/chx/MAX_mefs MTCH2 KO CHX dsRED mito-PAGFP15_thumb_w1Con-mcherry_t1-2.tif]

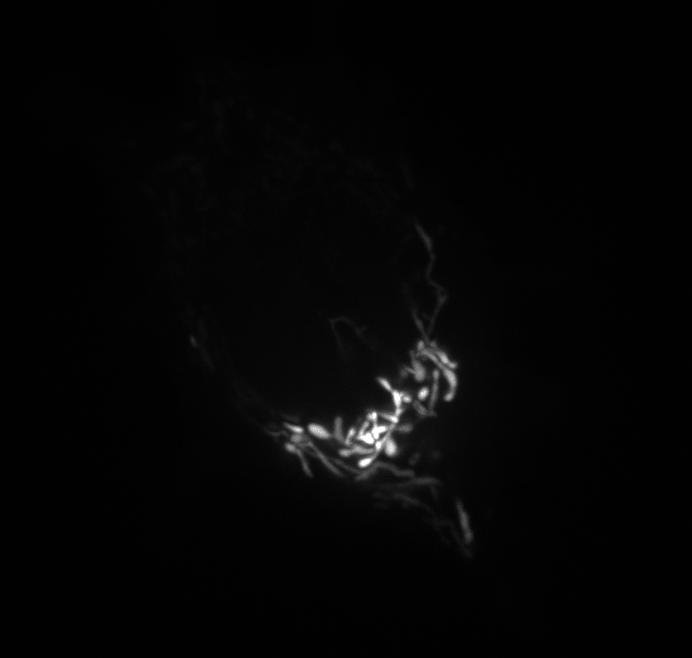

Supplement: Supplementary file 5 — Source Data EV Fig. 1 [file 44319_2023_9_MOESM5_ESM.zip › fig 4/g/IMAGES/chx/MAX_mefs MTCH2 KO CHX dsRED mito-PAGFP15_thumb_w1Con-mcherry_t1-3.tif]

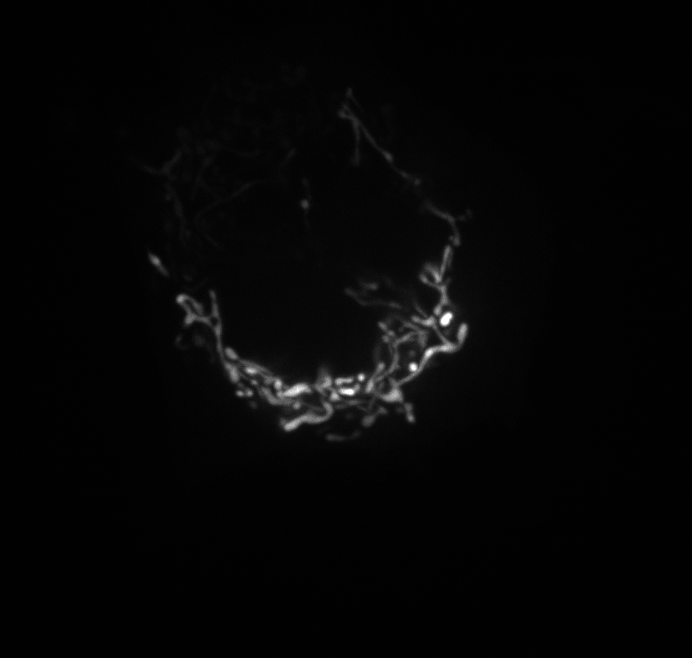

Supplement: Supplementary file 5 — Source Data EV Fig. 1 [file 44319_2023_9_MOESM5_ESM.zip › fig 4/g/IMAGES/chx/MAX_mefs MTCH2 KO CHX dsRED mito-PAGFP15_thumb_w1Con-mcherry_t1-4 t5.tif]

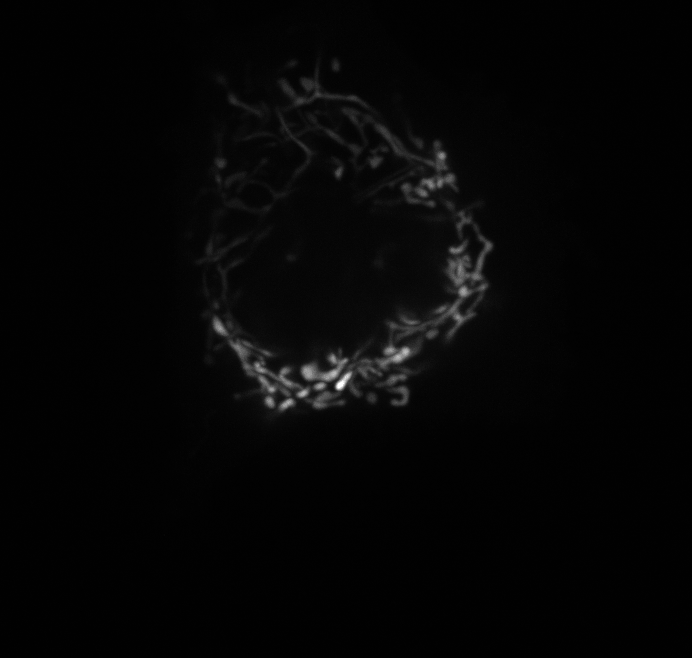

Supplement: Supplementary file 5 — Source Data EV Fig. 1 [file 44319_2023_9_MOESM5_ESM.zip › fig 4/g/IMAGES/chx/MAX_mefs MTCH2 KO CHX dsRED mito-PAGFP15_thumb_w1Con-mcherry_t1-5 t10.tif]

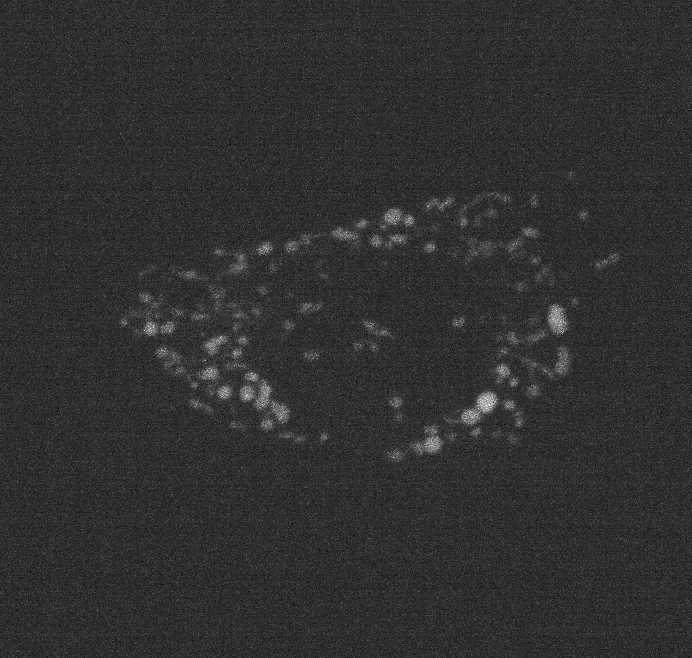

Supplement: Supplementary file 5 — Source Data EV Fig. 1 [file 44319_2023_9_MOESM5_ESM.zip › fig 4/g/IMAGES/ctrl/MAX_mefs MTCH2 KO control dsRED mito-PAGFP_thumb_w1Con-mcherry_t1-1.tif]

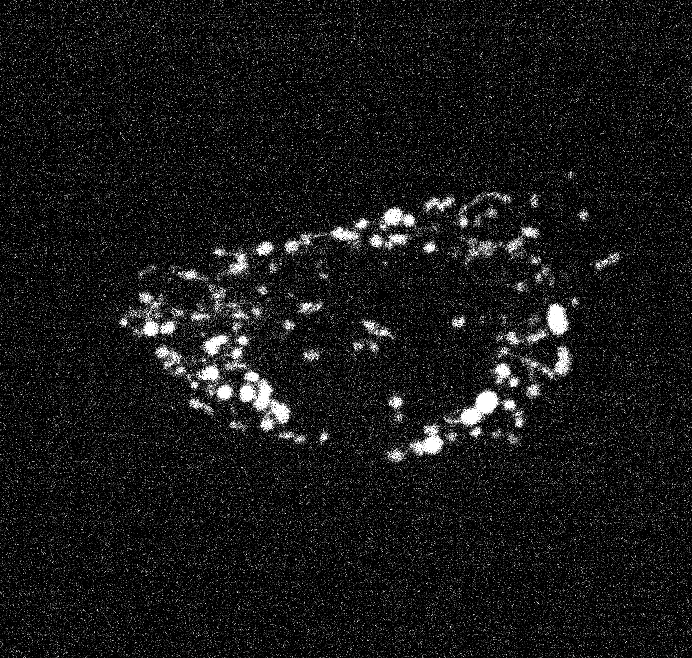

Supplement: Supplementary file 5 — Source Data EV Fig. 1 [file 44319_2023_9_MOESM5_ESM.zip › fig 4/g/IMAGES/ctrl/MAX_mefs MTCH2 KO control dsRED mito-PAGFP_thumb_w1Con-mcherry_t1-2.tif]

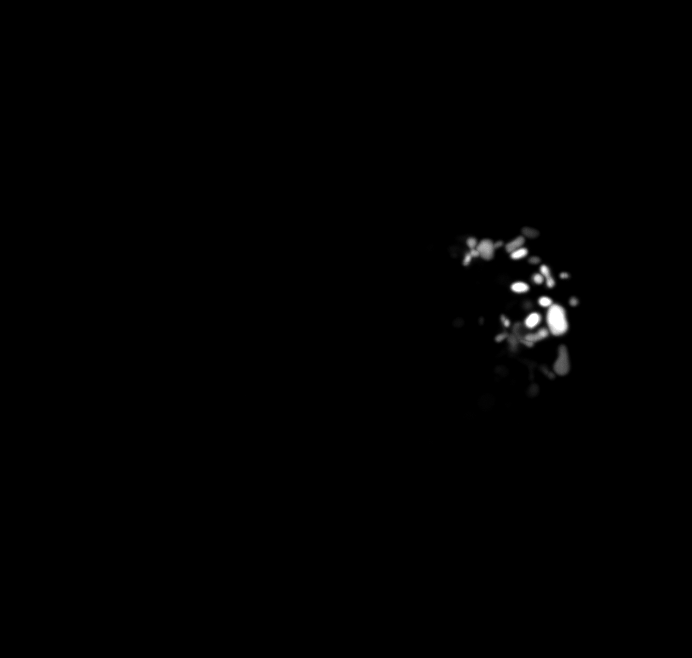

Supplement: Supplementary file 5 — Source Data EV Fig. 1 [file 44319_2023_9_MOESM5_ESM.zip › fig 4/g/IMAGES/ctrl/MAX_mefs MTCH2 KO control dsRED mito-PAGFP_thumb_w1Con-mcherry_t1-3 t0.tif]

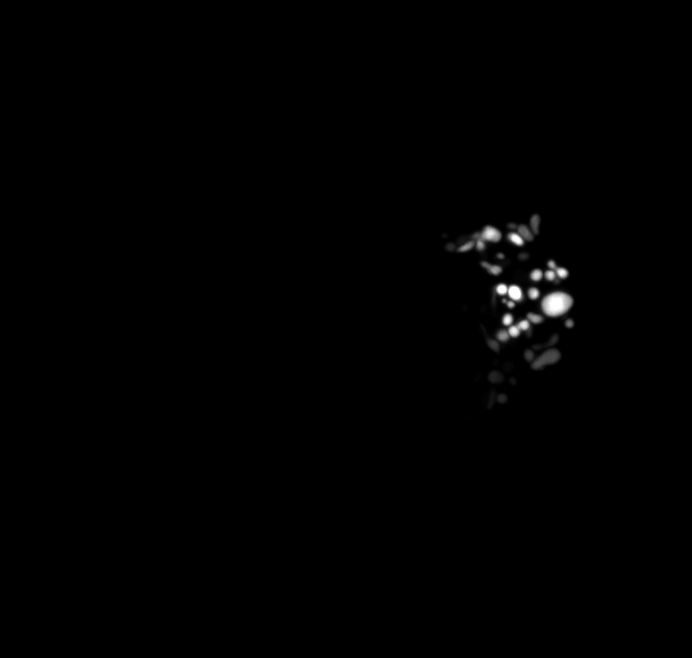

Supplement: Supplementary file 5 — Source Data EV Fig. 1 [file 44319_2023_9_MOESM5_ESM.zip › fig 4/g/IMAGES/ctrl/MAX_mefs MTCH2 KO control dsRED mito-PAGFP_thumb_w1Con-mcherry_t1-4 t5.tif]

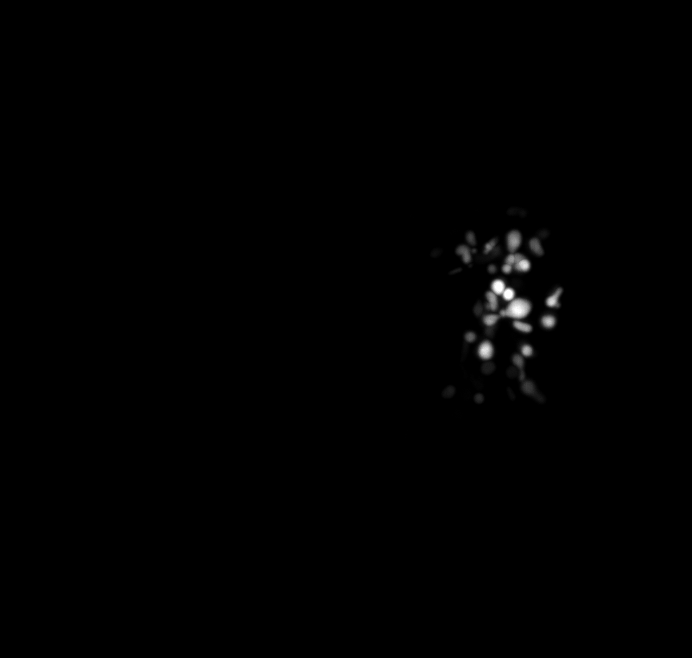

Supplement: Supplementary file 5 — Source Data EV Fig. 1 [file 44319_2023_9_MOESM5_ESM.zip › fig 4/g/IMAGES/ctrl/MAX_mefs MTCH2 KO control dsRED mito-PAGFP_thumb_w1Con-mcherry_t1-5 t10.tif]

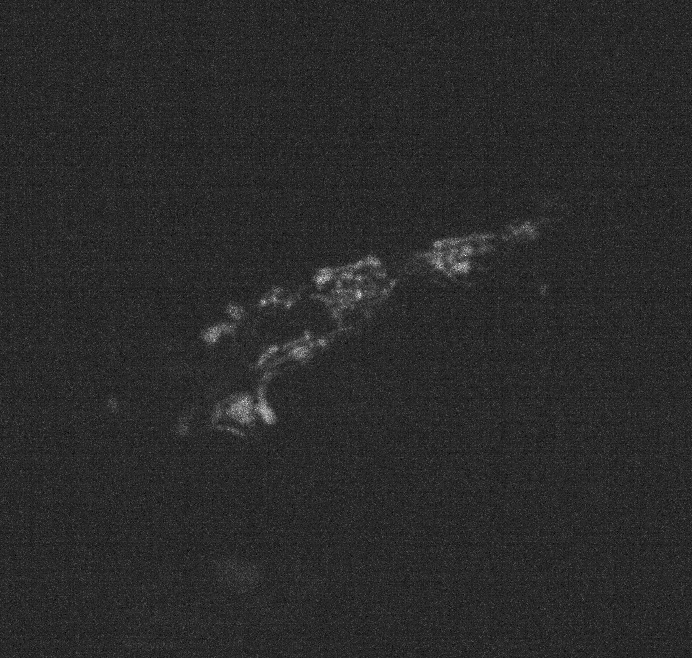

Supplement: Supplementary file 5 — Source Data EV Fig. 1 [file 44319_2023_9_MOESM5_ESM.zip › fig 4/g/IMAGES/fsg+chx/MAX_mtch2 ko fsg chx mito red pagfp1_thumb_w1Con-mcherry_t1-1.tif]

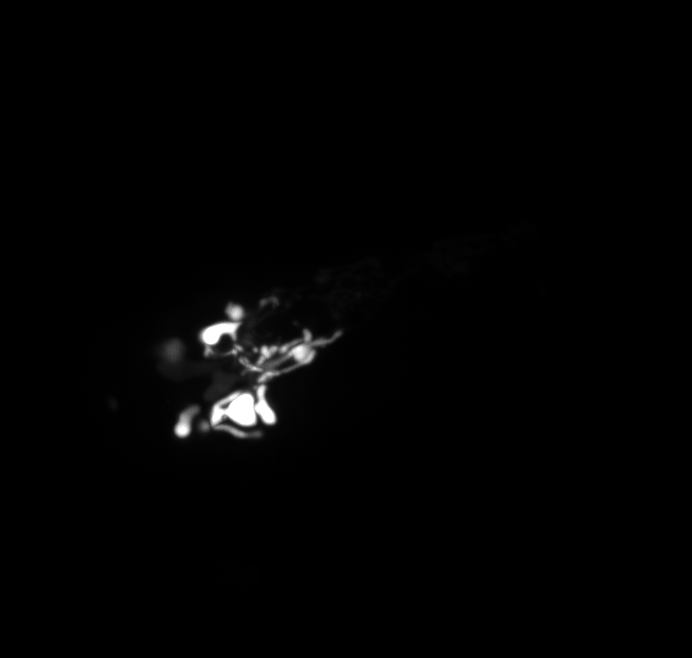

Supplement: Supplementary file 5 — Source Data EV Fig. 1 [file 44319_2023_9_MOESM5_ESM.zip › fig 4/g/IMAGES/fsg+chx/MAX_mtch2 ko fsg chx mito red pagfp1_thumb_w1Con-mcherry_t1-2 t0.tif]

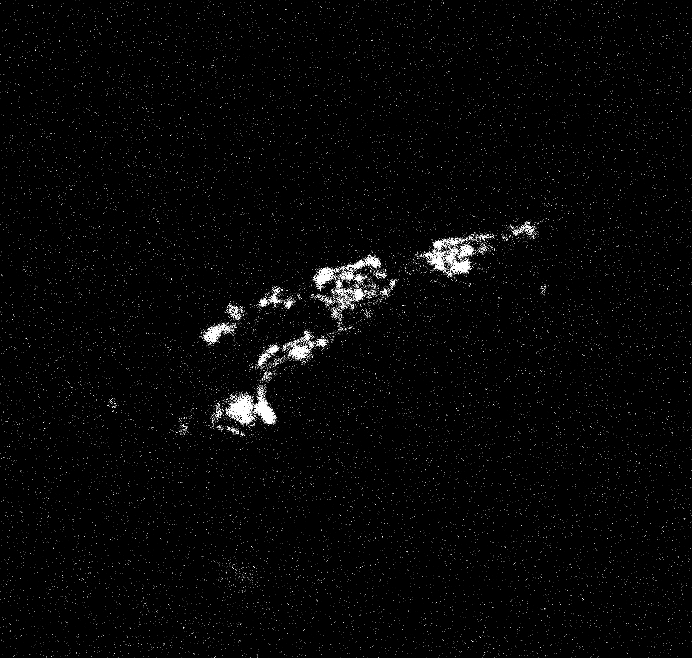

Supplement: Supplementary file 5 — Source Data EV Fig. 1 [file 44319_2023_9_MOESM5_ESM.zip › fig 4/g/IMAGES/fsg+chx/MAX_mtch2 ko fsg chx mito red pagfp1_thumb_w1Con-mcherry_t1-3.tif]

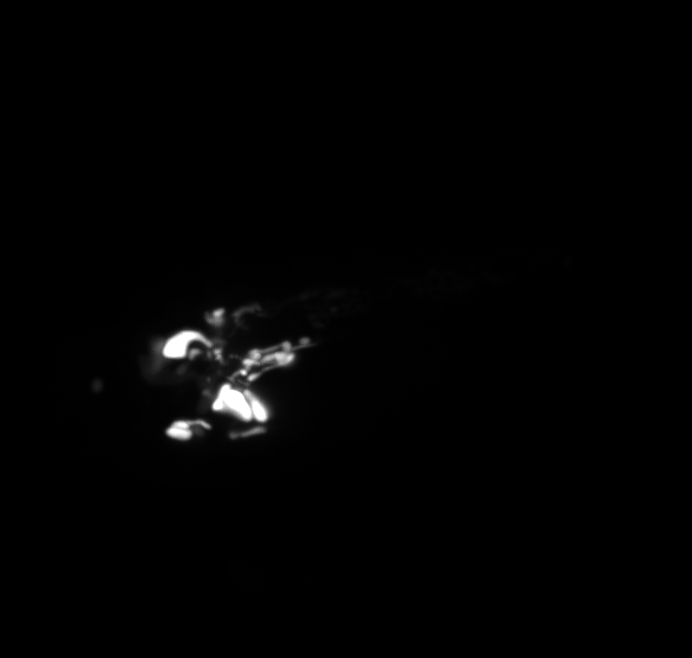

Supplement: Supplementary file 5 — Source Data EV Fig. 1 [file 44319_2023_9_MOESM5_ESM.zip › fig 4/g/IMAGES/fsg+chx/MAX_mtch2 ko fsg chx mito red pagfp1_thumb_w1Con-mcherry_t1-4t5.tif]

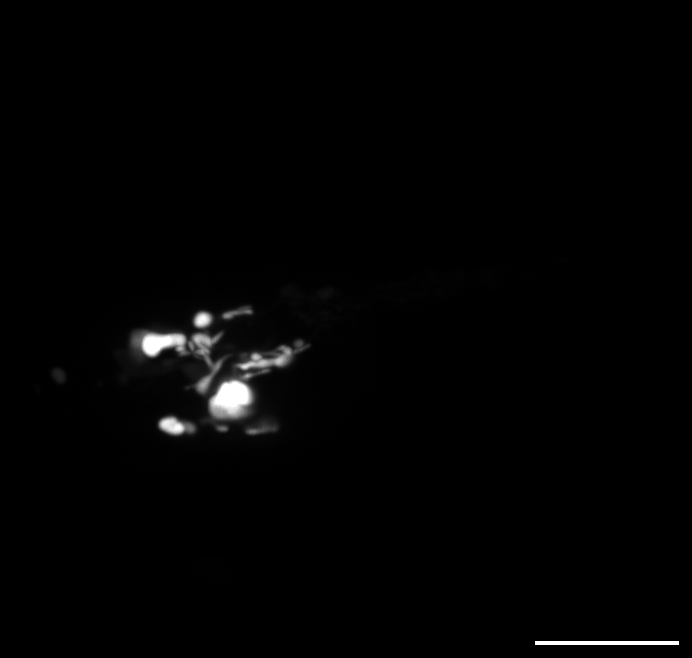

Supplement: Supplementary file 5 — Source Data EV Fig. 1 [file 44319_2023_9_MOESM5_ESM.zip › fig 4/g/IMAGES/fsg+chx/MAX_mtch2 ko fsg chx mito red pagfp1_thumb_w1Con-mcherry_t1-5t10-1 scale.tif]

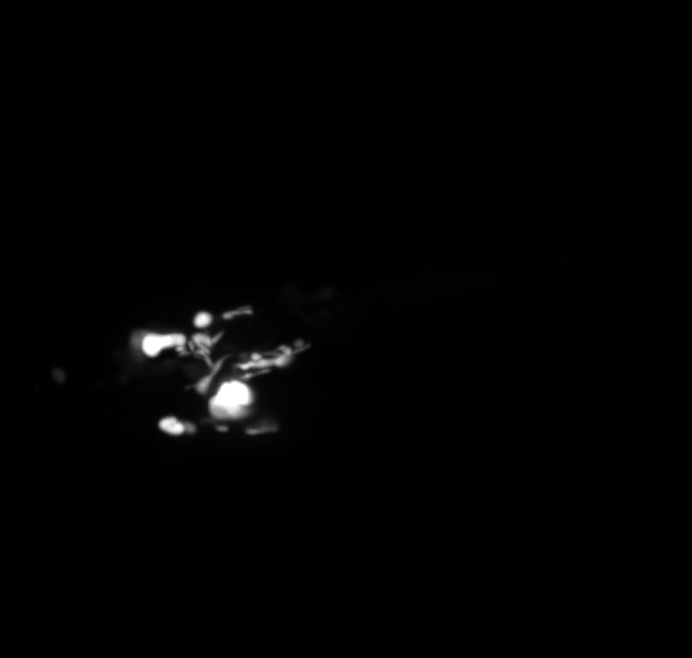

Supplement: Supplementary file 5 — Source Data EV Fig. 1 [file 44319_2023_9_MOESM5_ESM.zip › fig 4/g/IMAGES/fsg+chx/MAX_mtch2 ko fsg chx mito red pagfp1_thumb_w1Con-mcherry_t1-5t10.tif]

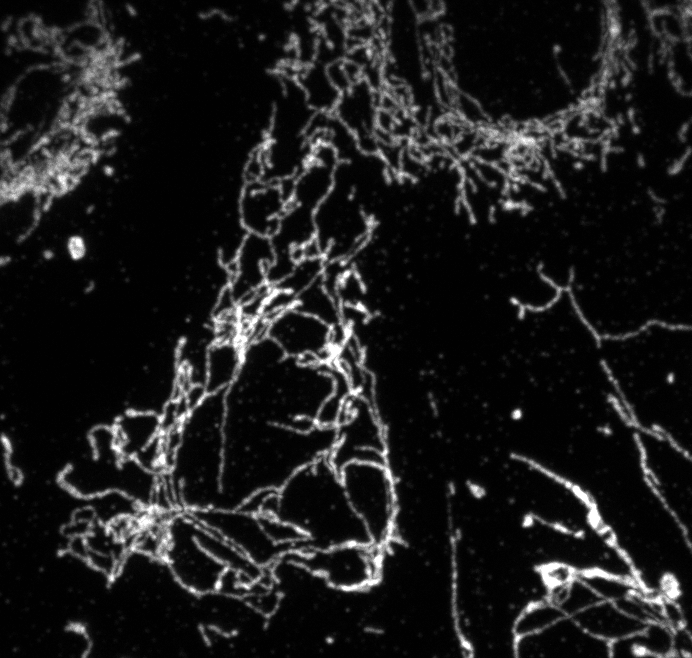

Supplement: Supplementary file 5 — Source Data EV Fig. 1 [file 44319_2023_9_MOESM5_ESM.zip › fig 4/i/IMAGES/MTCH2 KO GPAT3-4 si/CHX/MAX_MEFs MTCH2 KO GPAT3-4 si HBSS TOM40598 CYT633 DAPI1_thumb_w1Con-mcherry_s1.TIF - Stage4 -1.tif]

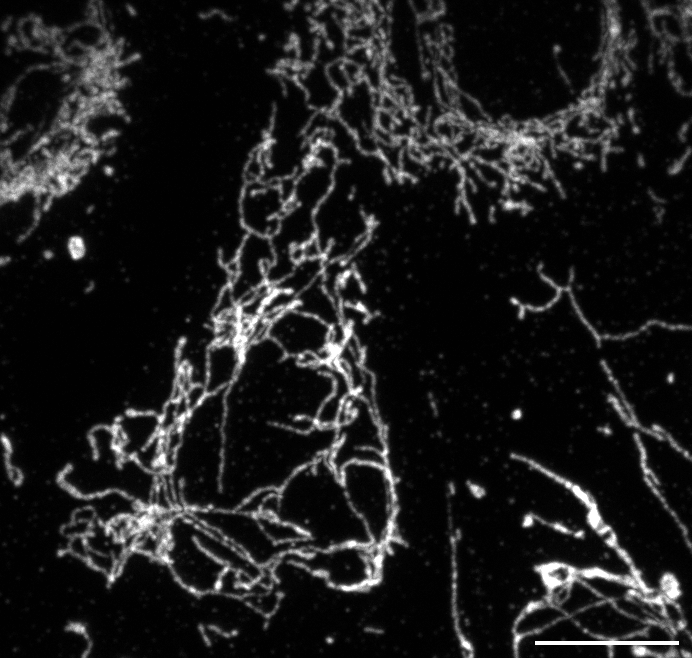

Supplement: Supplementary file 5 — Source Data EV Fig. 1 [file 44319_2023_9_MOESM5_ESM.zip › fig 4/i/IMAGES/MTCH2 KO GPAT3-4 si/CHX/MAX_MEFs MTCH2 KO GPAT3-4 si HBSS TOM40598 CYT633 DAPI1_thumb_w1Con-mcherry_s1.TIF - Stage4 -2scale.tif]

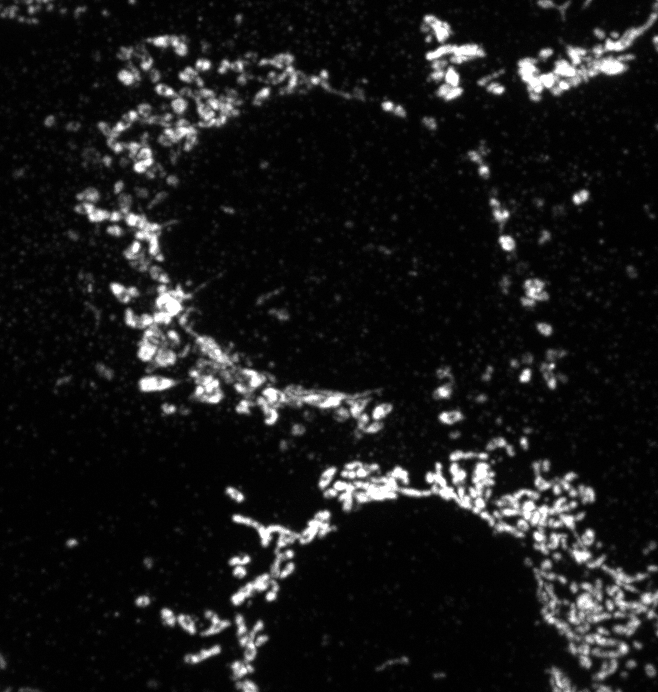

Supplement: Supplementary file 5 — Source Data EV Fig. 1 [file 44319_2023_9_MOESM5_ESM.zip › fig 4/i/IMAGES/MTCH2 KO GPAT3-4 si/CONTROL/MAX_MEFs MTCH2 KO GPAT3-4 si CONTROL TOM40598 CYT633 DAPI1_thumb_w1Con-mcherry_s1.TIF - Stage30-1.tif]

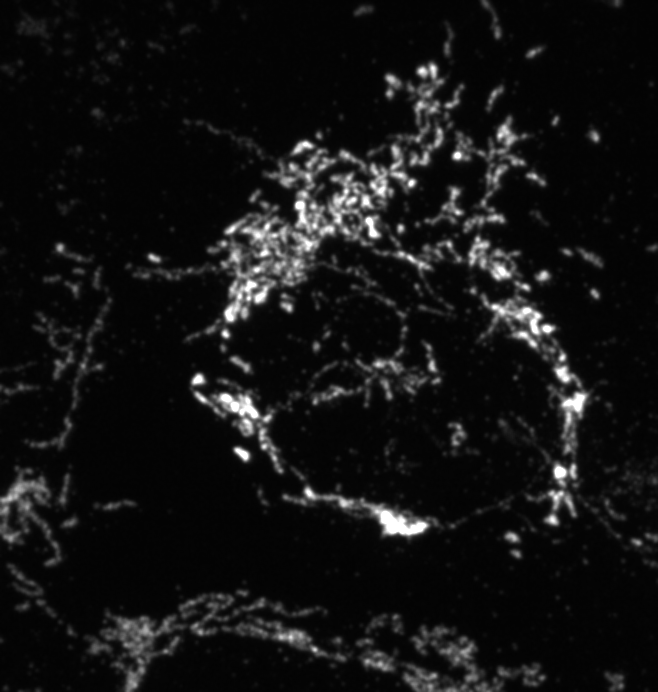

Supplement: Supplementary file 5 — Source Data EV Fig. 1 [file 44319_2023_9_MOESM5_ESM.zip › fig 4/i/IMAGES/MTCH2 KO GPAT3-4 si/HBSS/MAX_MEFs MTCH2 KO GPAT3-4 si HBSS TOM40598 CYT633 DAPI_thumb_w1Con-mcherry_s1.TIF - Stage46 -1-1.tif]

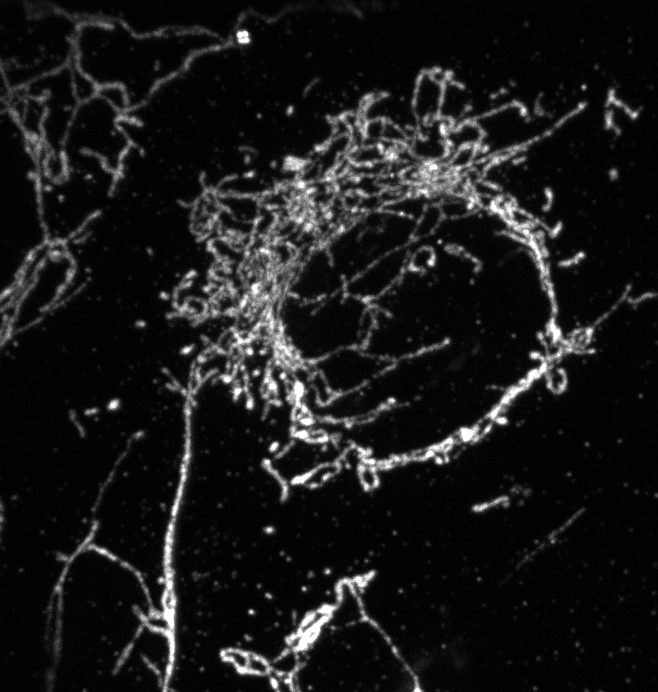

Supplement: Supplementary file 5 — Source Data EV Fig. 1 [file 44319_2023_9_MOESM5_ESM.zip › fig 4/i/IMAGES/MTCH2 KO NTsi/CHX/MAX_MEFs MTCH2 KO NT si CHX TOM40598 CYT633 DAPI2_thumb_w1Con-mcherry_s1.TIF - Stage15-1.tif]

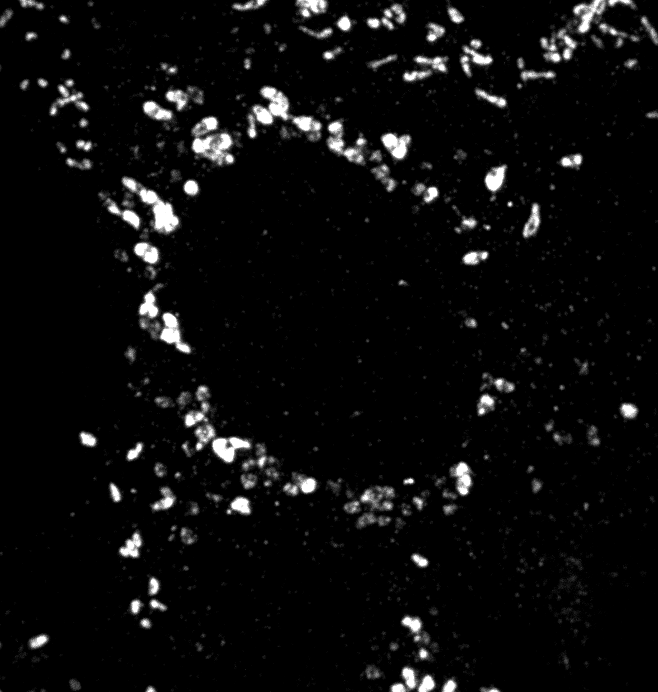

Supplement: Supplementary file 5 — Source Data EV Fig. 1 [file 44319_2023_9_MOESM5_ESM.zip › fig 4/i/IMAGES/MTCH2 KO NTsi/CONTROL/MAX_MEFs MTCH2 KO NT si CONTROL TOM40598 CYT633 DAPI2_thumb_w1Con-mcherry_s1.TIF - Stage7 -1-1.tif]

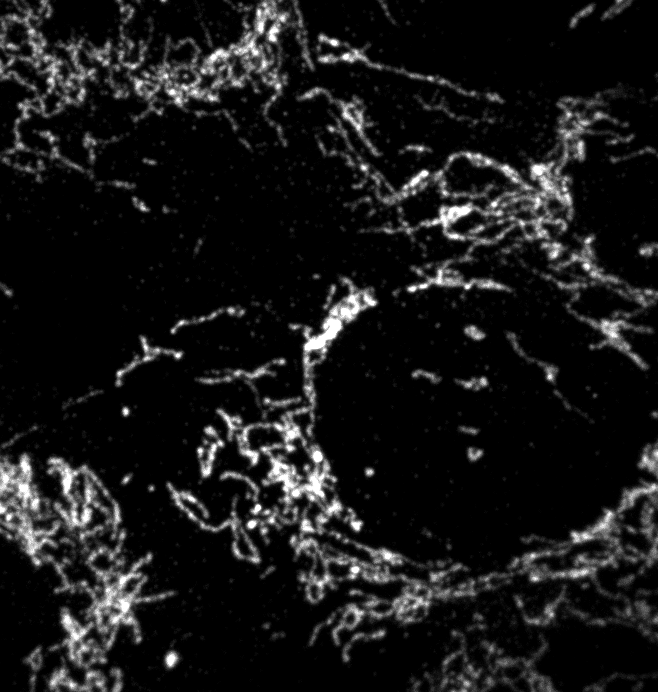

Supplement: Supplementary file 5 — Source Data EV Fig. 1 [file 44319_2023_9_MOESM5_ESM.zip › fig 4/i/IMAGES/MTCH2 KO NTsi/HBSS/C2-MAX_MEFs MTCH2 KO GPAT3-4 si CONTROL TOM40598 CYT633 DAPI2_thumb_w1Con-mcherry_s1.TIF - Stage9 -1-1.tif]

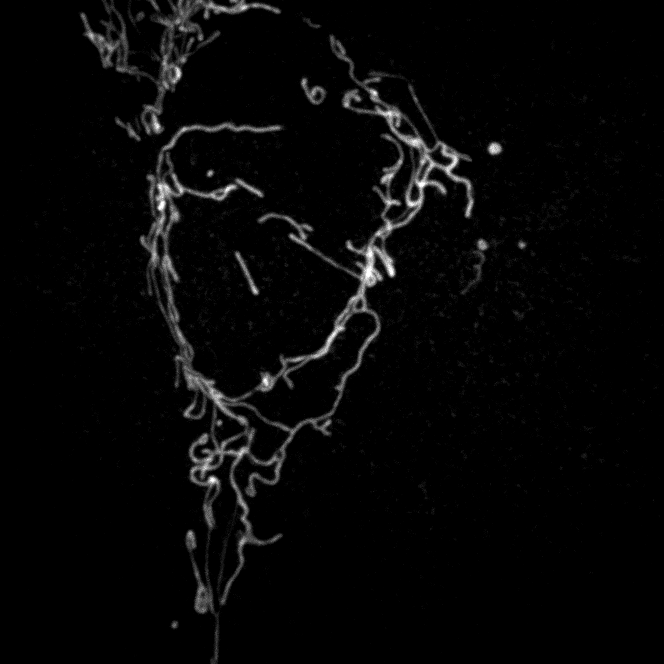

Supplement: Supplementary file 5 — Source Data EV Fig. 1 [file 44319_2023_9_MOESM5_ESM.zip › fig 4/l/IMAGES/CHX/MAX_ff CHX 44_thumb_w1Con-Cy5-1 8BIT.tif]

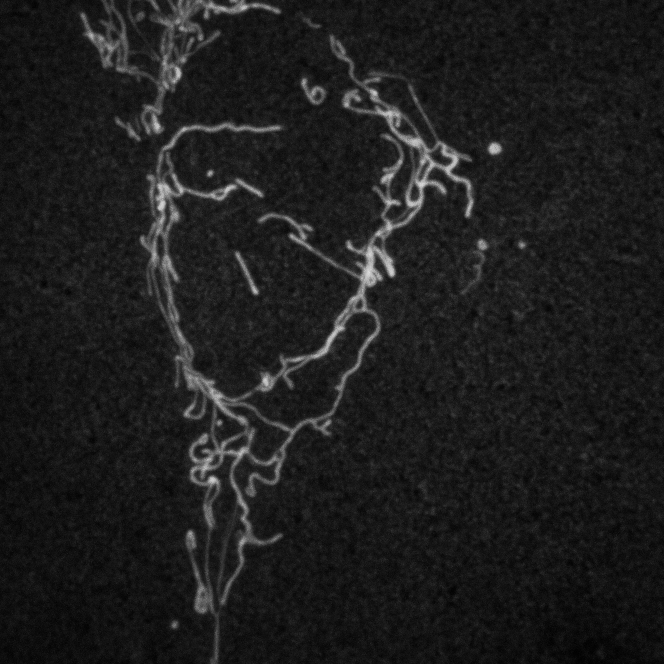

Supplement: Supplementary file 5 — Source Data EV Fig. 1 [file 44319_2023_9_MOESM5_ESM.zip › fig 4/l/IMAGES/CHX/MAX_ff CHX 44_thumb_w1Con-Cy5-1.tif]

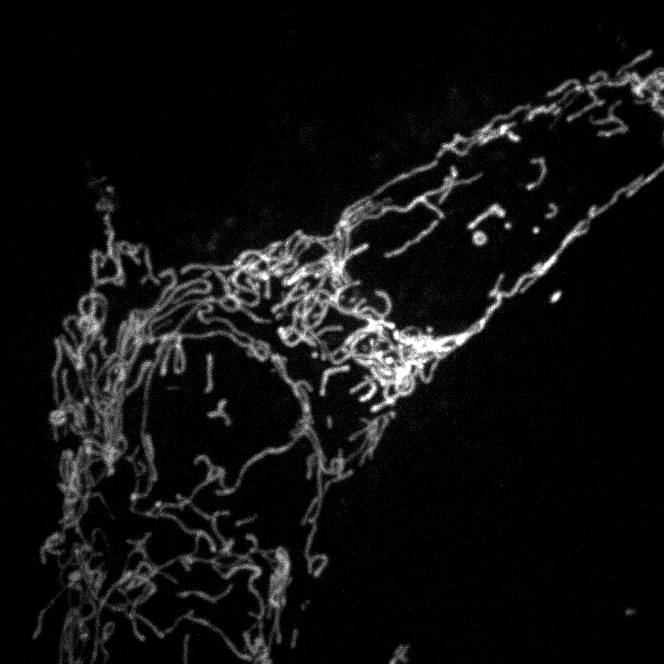

Supplement: Supplementary file 5 — Source Data EV Fig. 1 [file 44319_2023_9_MOESM5_ESM.zip › fig 4/l/IMAGES/CHX/MAX_mfn2 si CHX 60_thumb_w1Con-Cy5-1 8BIT.tif]

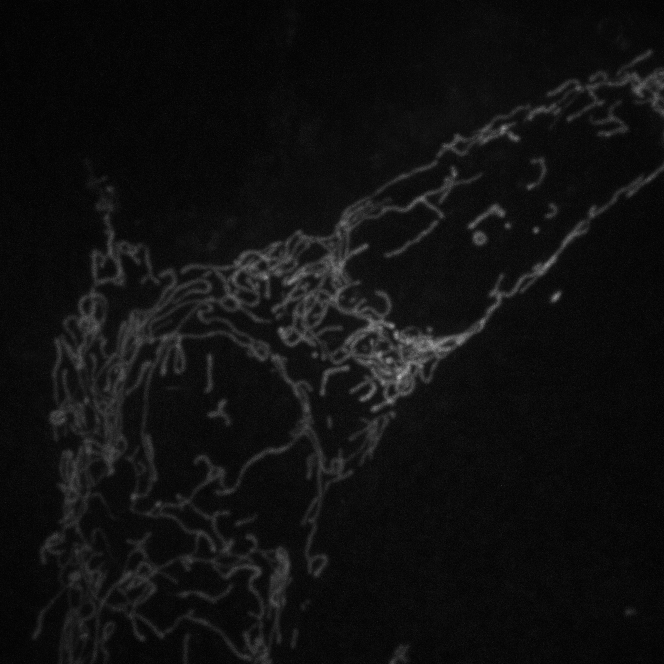

Supplement: Supplementary file 5 — Source Data EV Fig. 1 [file 44319_2023_9_MOESM5_ESM.zip › fig 4/l/IMAGES/CHX/MAX_mfn2 si CHX 60_thumb_w1Con-Cy5-1.tif]

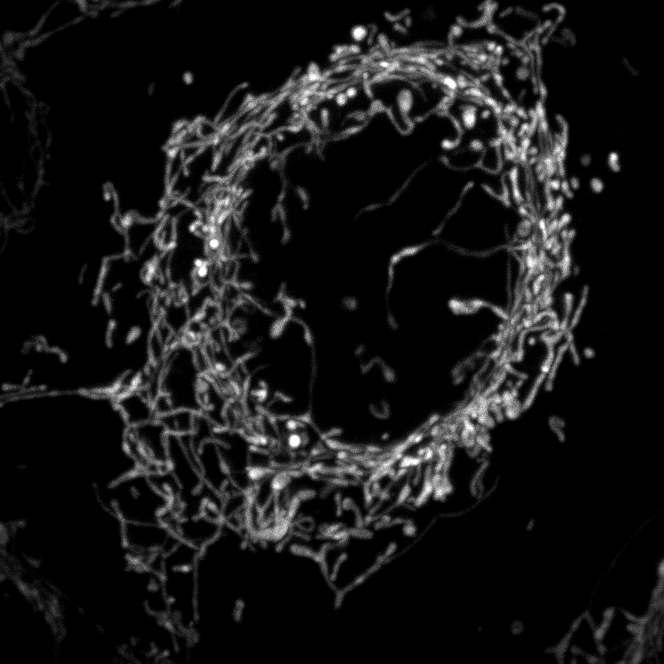

Supplement: Supplementary file 5 — Source Data EV Fig. 1 [file 44319_2023_9_MOESM5_ESM.zip › fig 4/l/IMAGES/CHX/MAX_mtch2 ko CHX 30_thumb_w1Con-Cy5-1 8BIT.tif]

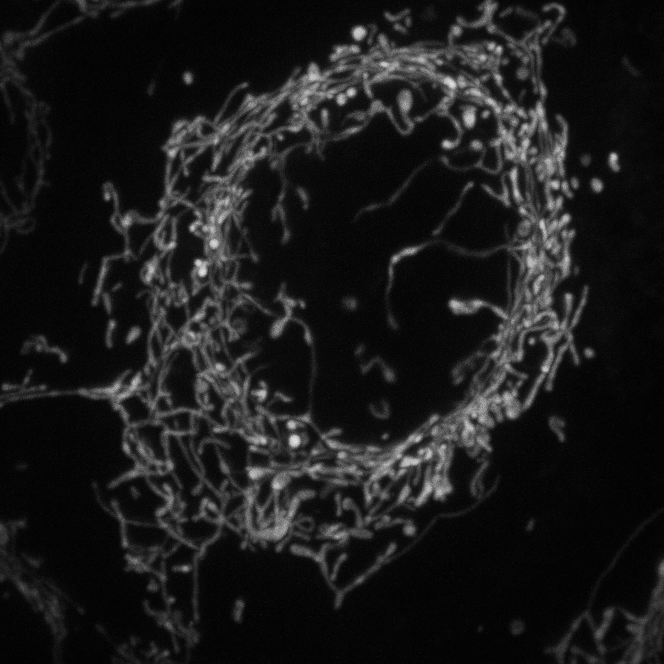

Supplement: Supplementary file 5 — Source Data EV Fig. 1 [file 44319_2023_9_MOESM5_ESM.zip › fig 4/l/IMAGES/CHX/MAX_mtch2 ko CHX 30_thumb_w1Con-Cy5-1.tif]

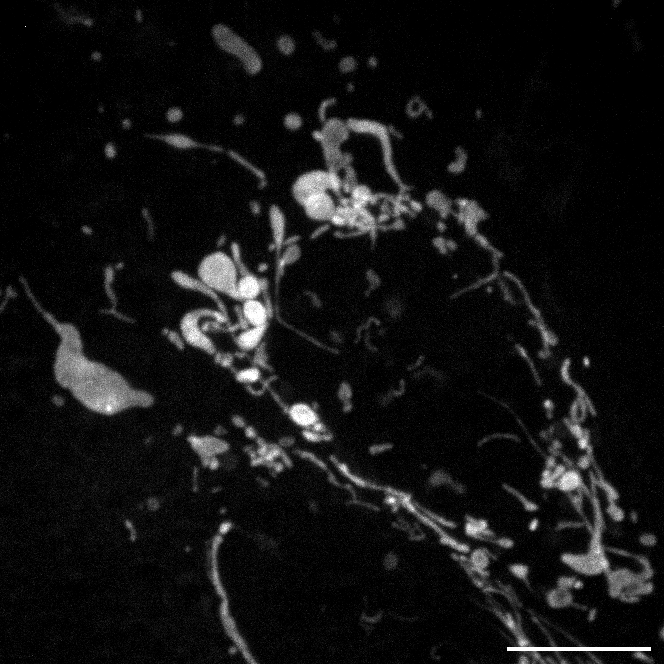

Supplement: Supplementary file 5 — Source Data EV Fig. 1 [file 44319_2023_9_MOESM5_ESM.zip › fig 4/l/IMAGES/CHX/MAX_mtch2 ko mfn2 si CHX 10_thumb_w1Con-Cy5-1 8BIT-1 10um scale bar.tif]

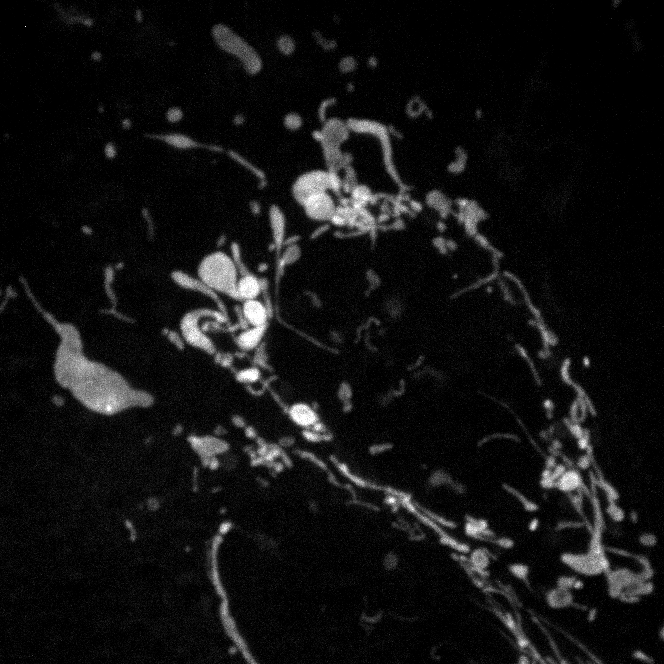

Supplement: Supplementary file 5 — Source Data EV Fig. 1 [file 44319_2023_9_MOESM5_ESM.zip › fig 4/l/IMAGES/CHX/MAX_mtch2 ko mfn2 si CHX 10_thumb_w1Con-Cy5-1 8BIT.tif]

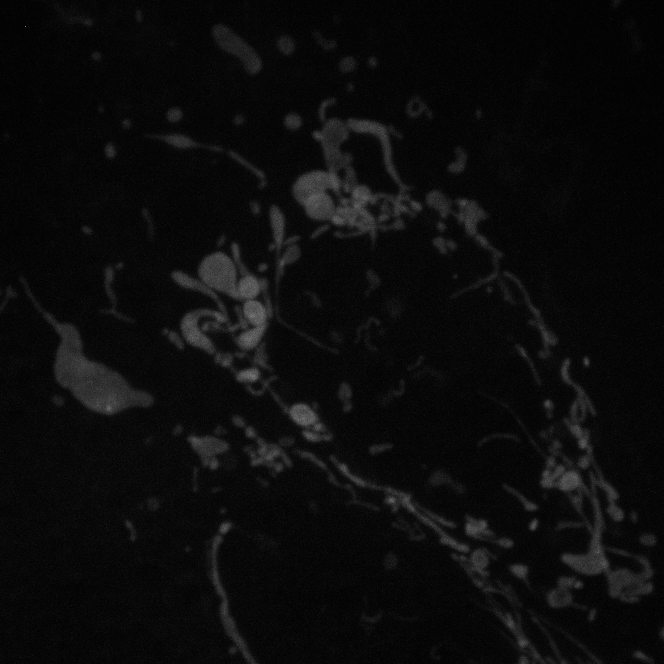

Supplement: Supplementary file 5 — Source Data EV Fig. 1 [file 44319_2023_9_MOESM5_ESM.zip › fig 4/l/IMAGES/CHX/MAX_mtch2 ko mfn2 si CHX 10_thumb_w1Con-Cy5-1.tif]

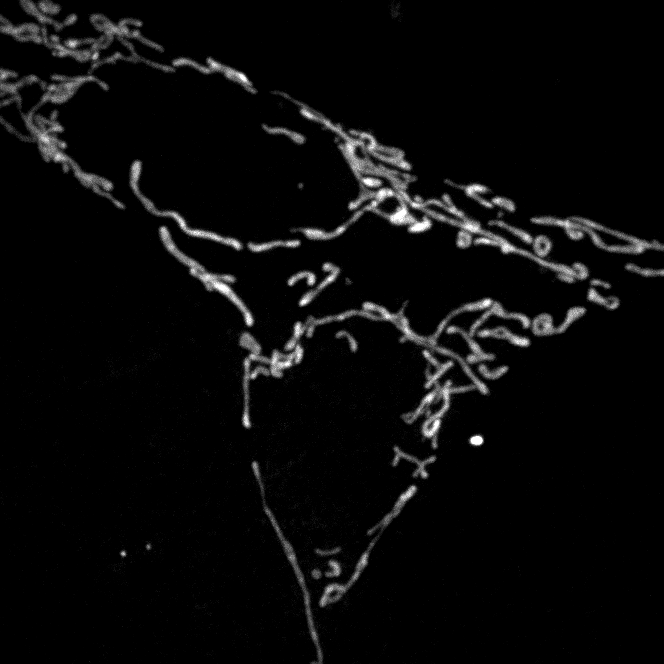

Supplement: Supplementary file 5 — Source Data EV Fig. 1 [file 44319_2023_9_MOESM5_ESM.zip › fig 4/l/IMAGES/CONTROL/MAX_mefs control3_thumb_w1Con-Cy5-1 8BIT.tif]

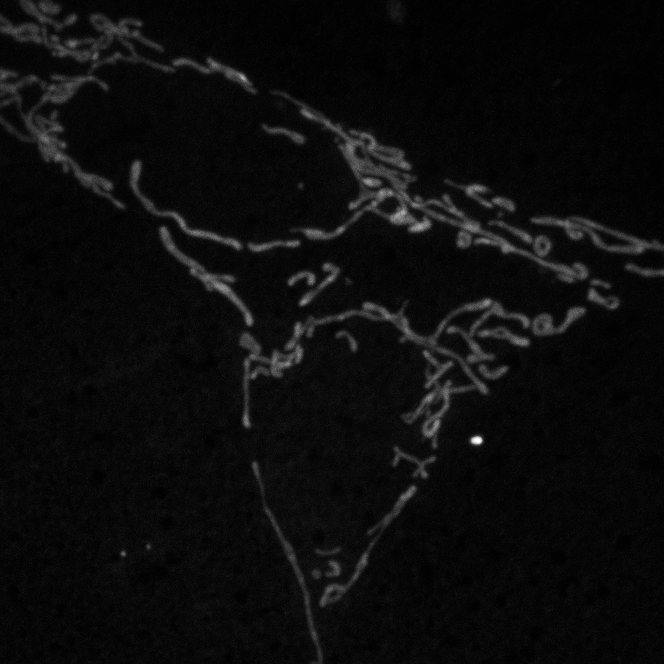

Supplement: Supplementary file 5 — Source Data EV Fig. 1 [file 44319_2023_9_MOESM5_ESM.zip › fig 4/l/IMAGES/CONTROL/MAX_mefs control3_thumb_w1Con-Cy5-1.tif]

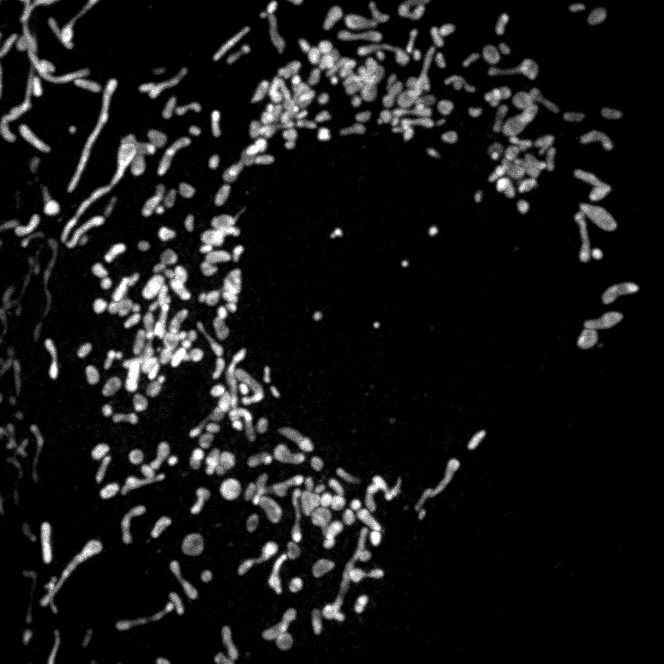

Supplement: Supplementary file 5 — Source Data EV Fig. 1 [file 44319_2023_9_MOESM5_ESM.zip › fig 4/l/IMAGES/CONTROL/MAX_mefsmfn2 si control23_thumb_w1Con-Cy5-1 8BIT.tif]

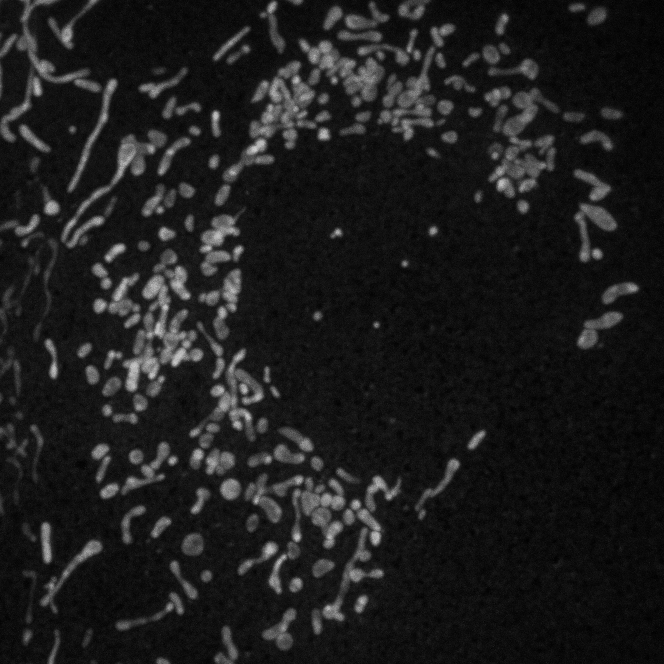

Supplement: Supplementary file 5 — Source Data EV Fig. 1 [file 44319_2023_9_MOESM5_ESM.zip › fig 4/l/IMAGES/CONTROL/MAX_mefsmfn2 si control23_thumb_w1Con-Cy5-1.tif]

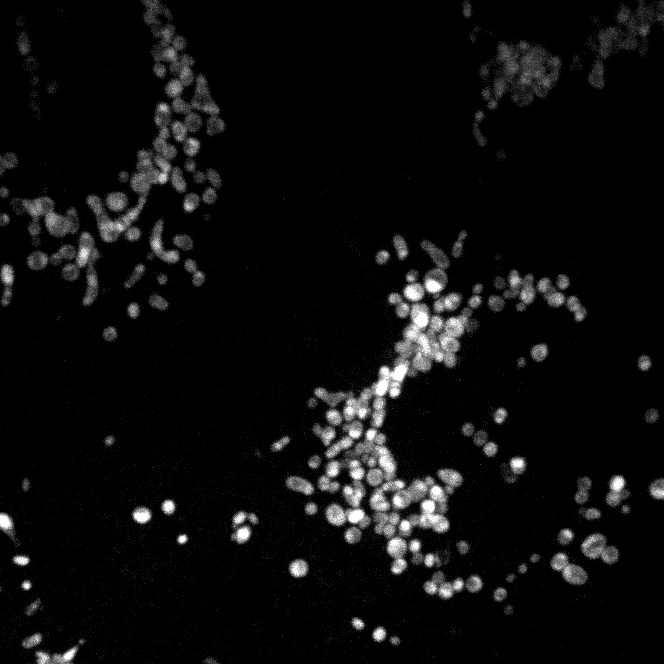

Supplement: Supplementary file 5 — Source Data EV Fig. 1 [file 44319_2023_9_MOESM5_ESM.zip › fig 4/l/IMAGES/CONTROL/MAX_mtch2 ko control37_thumb_w1Con-Cy5-1 8BUT.tif]

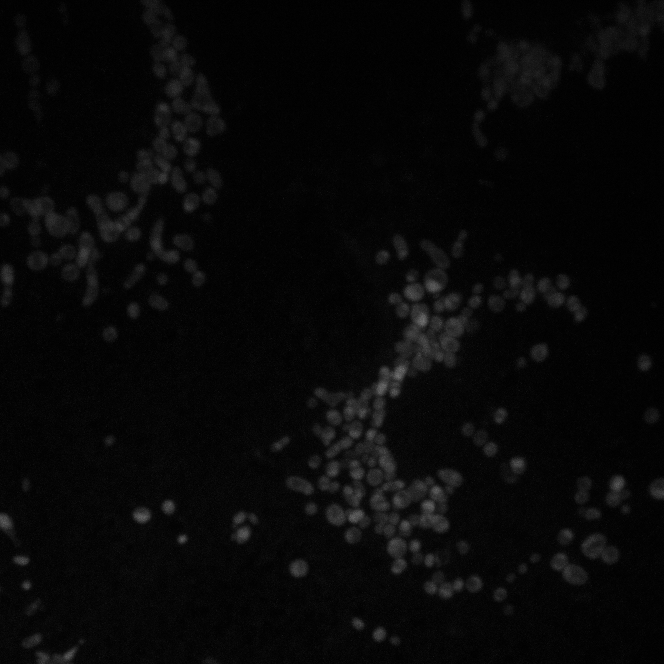

Supplement: Supplementary file 5 — Source Data EV Fig. 1 [file 44319_2023_9_MOESM5_ESM.zip › fig 4/l/IMAGES/CONTROL/MAX_mtch2 ko control37_thumb_w1Con-Cy5-1.tif]

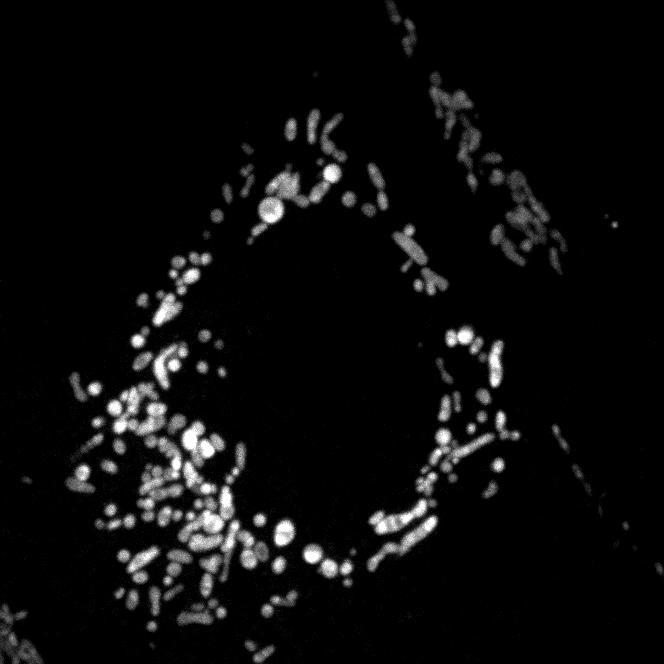

Supplement: Supplementary file 5 — Source Data EV Fig. 1 [file 44319_2023_9_MOESM5_ESM.zip › fig 4/l/IMAGES/CONTROL/MAX_mtch2 ko mfn2 si control55_thumb_w1Con-Cy5-1 8BIT.tif]

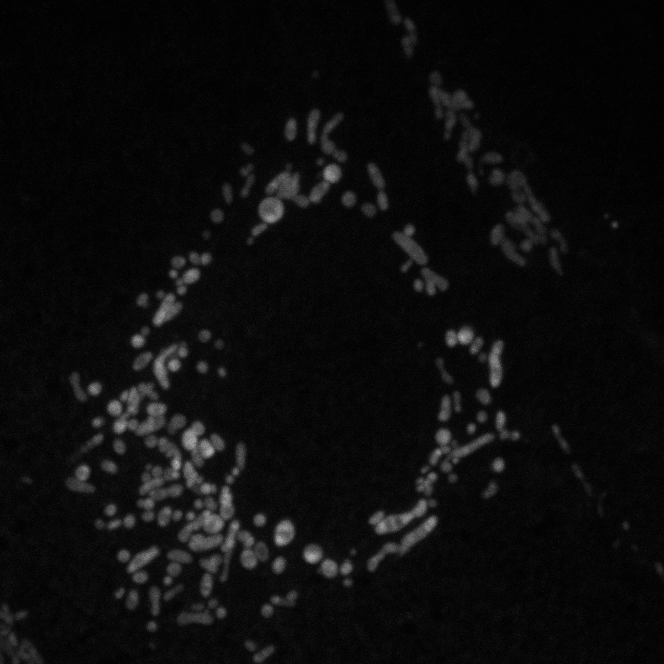

Supplement: Supplementary file 5 — Source Data EV Fig. 1 [file 44319_2023_9_MOESM5_ESM.zip › fig 4/l/IMAGES/CONTROL/MAX_mtch2 ko mfn2 si control55_thumb_w1Con-Cy5-1.tif]

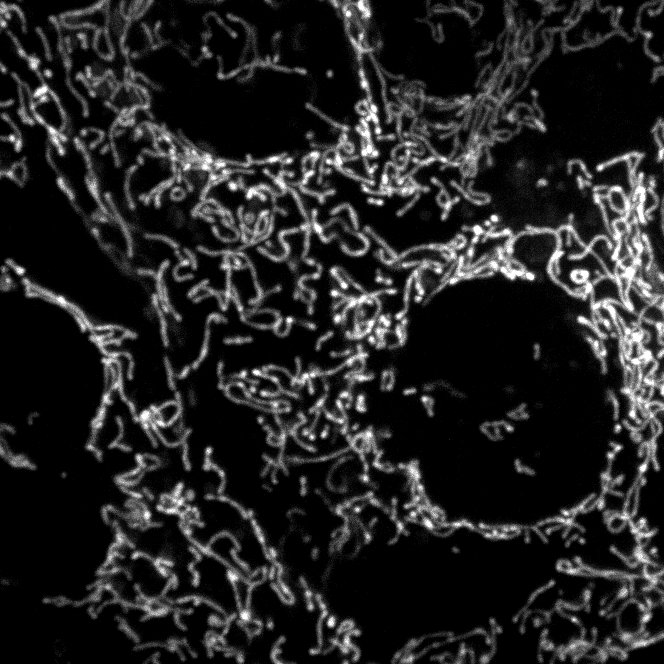

Supplement: Supplementary file 5 — Source Data EV Fig. 1 [file 44319_2023_9_MOESM5_ESM.zip › fig 4/l/IMAGES/HBSS/MAX_ff hbss56_thumb_w1Con-Cy5-1 8BIT.tif]

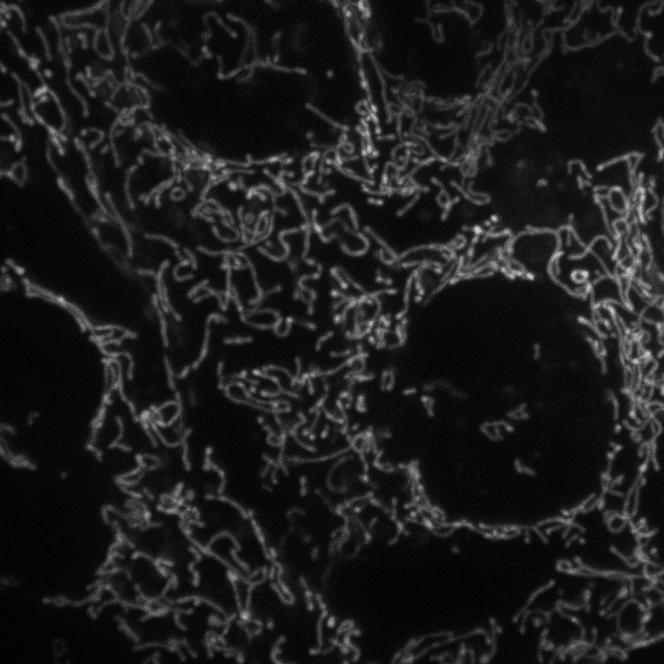

Supplement: Supplementary file 5 — Source Data EV Fig. 1 [file 44319_2023_9_MOESM5_ESM.zip › fig 4/l/IMAGES/HBSS/MAX_ff hbss56_thumb_w1Con-Cy5-1.tif]

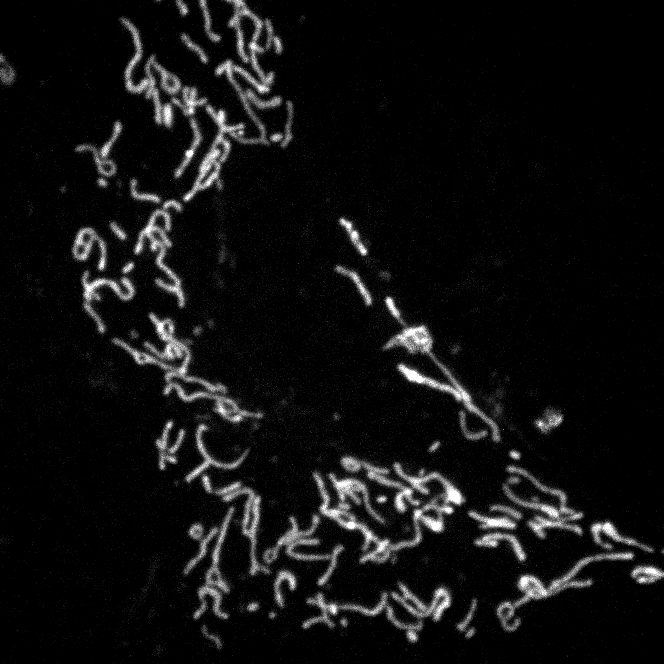

Supplement: Supplementary file 5 — Source Data EV Fig. 1 [file 44319_2023_9_MOESM5_ESM.zip › fig 4/l/IMAGES/HBSS/MAX_mfn2 si hbss64_thumb_w1Con-Cy5-1 8BIT.tif]

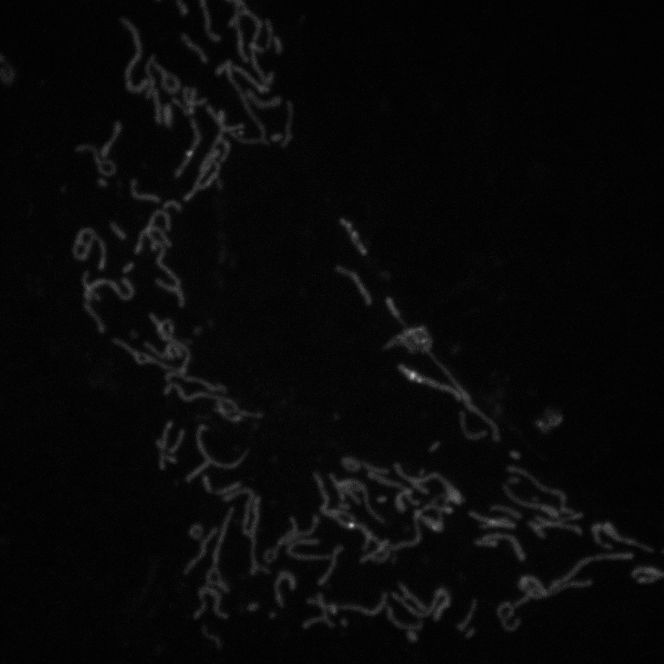

Supplement: Supplementary file 5 — Source Data EV Fig. 1 [file 44319_2023_9_MOESM5_ESM.zip › fig 4/l/IMAGES/HBSS/MAX_mfn2 si hbss64_thumb_w1Con-Cy5-1.tif]

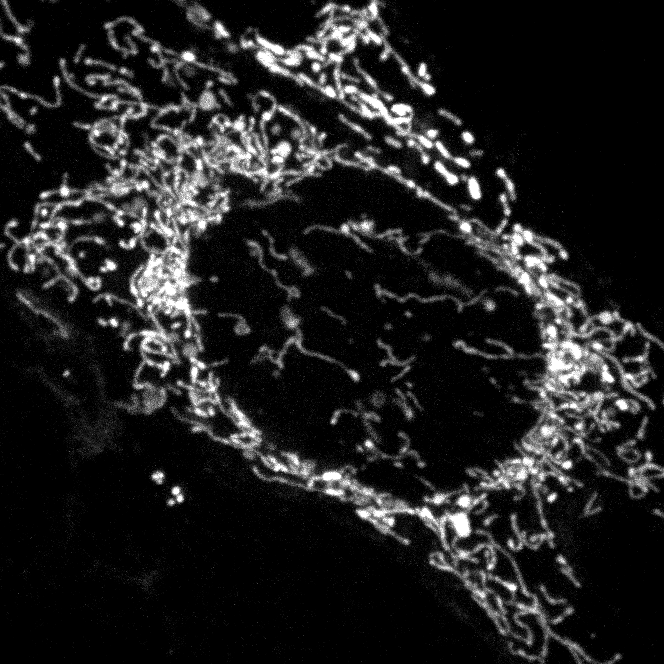

Supplement: Supplementary file 5 — Source Data EV Fig. 1 [file 44319_2023_9_MOESM5_ESM.zip › fig 4/l/IMAGES/HBSS/MAX_MTCH2 KO i hbss42_thumb_w1Con-Cy5-1 8BIT.tif]

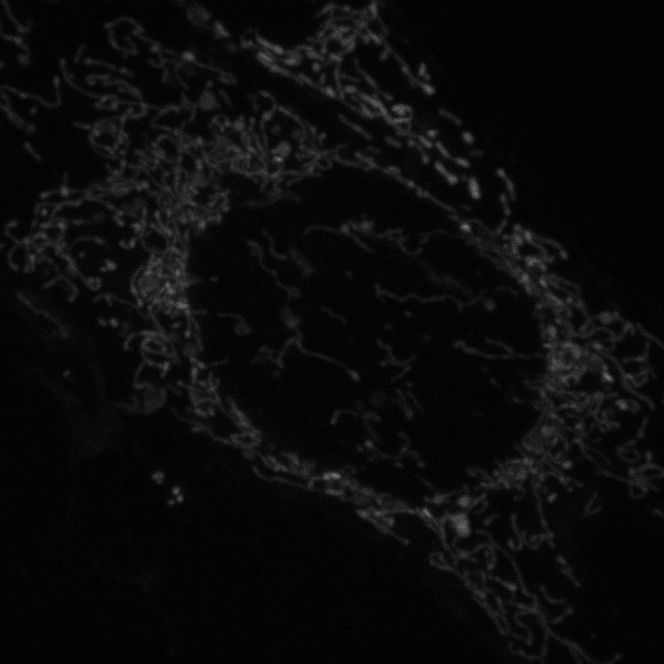

Supplement: Supplementary file 5 — Source Data EV Fig. 1 [file 44319_2023_9_MOESM5_ESM.zip › fig 4/l/IMAGES/HBSS/MAX_MTCH2 KO i hbss42_thumb_w1Con-Cy5-1.tif]

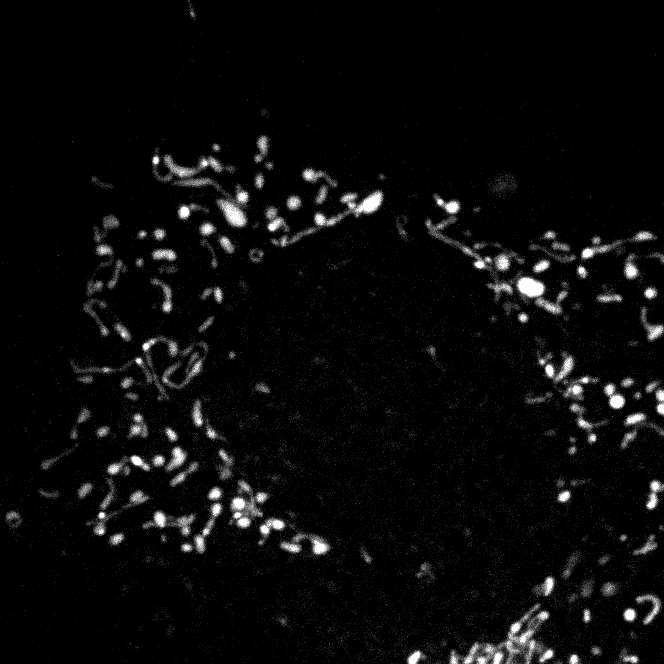

Supplement: Supplementary file 5 — Source Data EV Fig. 1 [file 44319_2023_9_MOESM5_ESM.zip › fig 4/l/IMAGES/HBSS/MAX_MTCH2 KOmfn2 si hbss14_thumb_w1Con-Cy5-1 8BIT.tif]

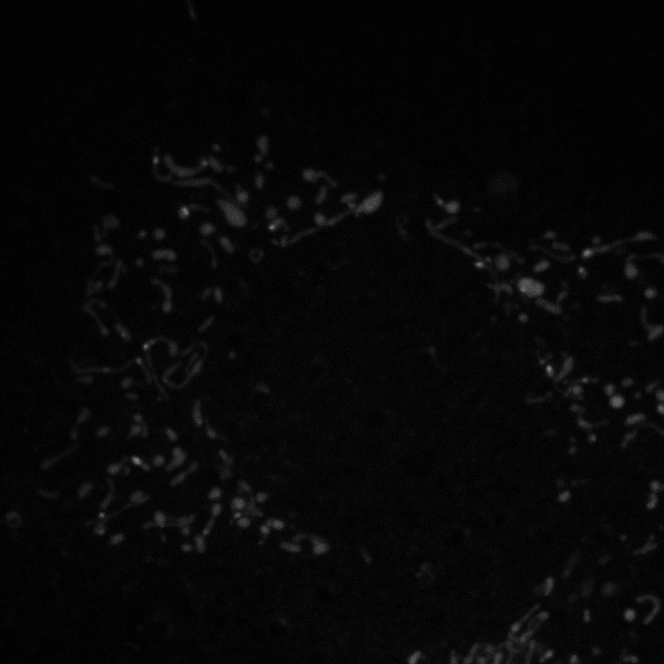

Supplement: Supplementary file 5 — Source Data EV Fig. 1 [file 44319_2023_9_MOESM5_ESM.zip › fig 4/l/IMAGES/HBSS/MAX_MTCH2 KOmfn2 si hbss14_thumb_w1Con-Cy5-1.tif]

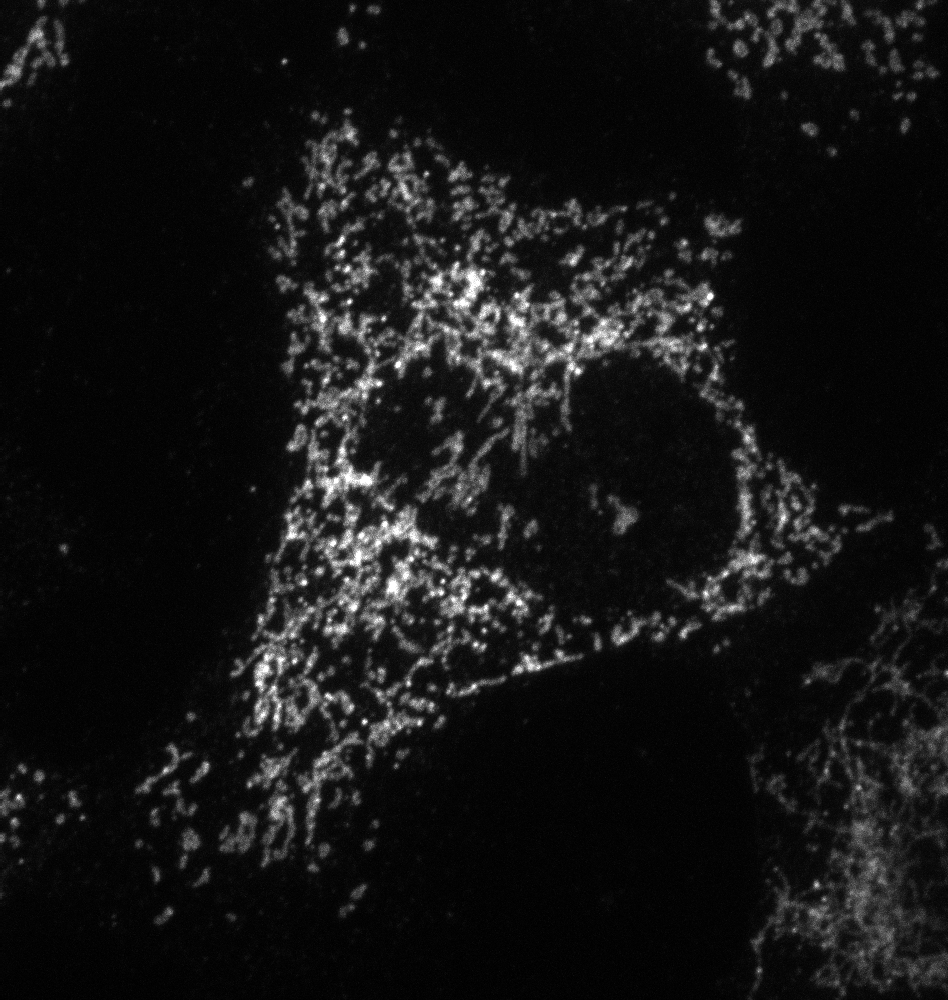

Supplement: Supplementary file 6 — Source Data EV Fig. 2 [file 44319_2023_9_MOESM6_ESM.zip › EV1/a/mfn1 ko/control/MAX_Experiment-1284 MFN1KO-1.tif]

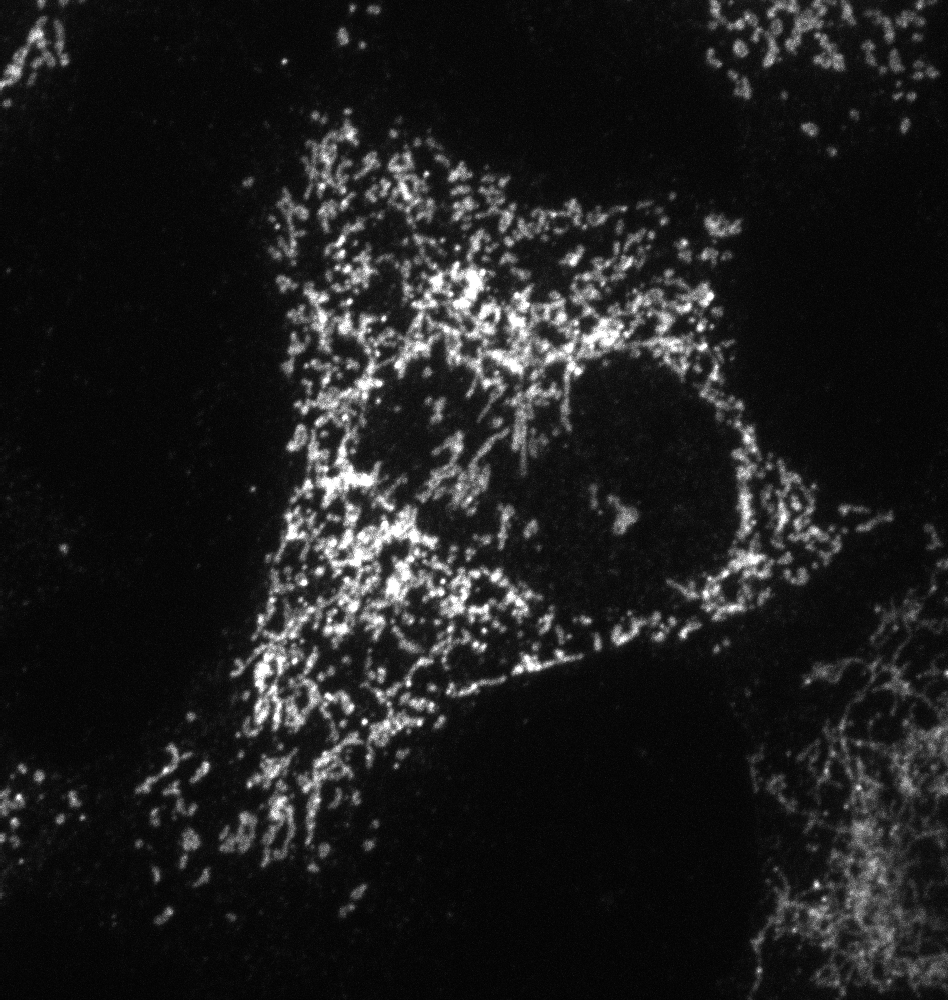

Supplement: Supplementary file 6 — Source Data EV Fig. 2 [file 44319_2023_9_MOESM6_ESM.zip › EV1/a/mfn1 ko/control/MAX_Experiment-1284 MFN1KO-1rgb.tif]

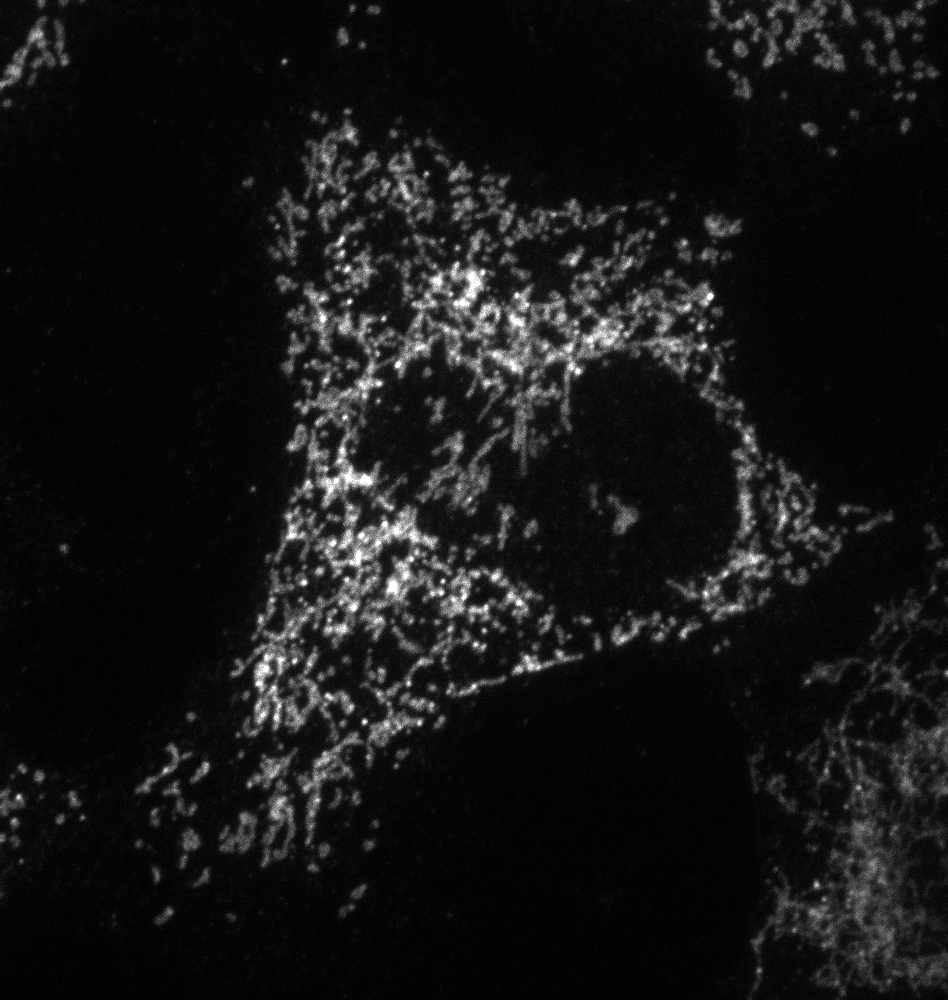

Supplement: Supplementary file 6 — Source Data EV Fig. 2 [file 44319_2023_9_MOESM6_ESM.zip › EV1/a/mfn1 ko/control/MAX_Experiment-1284 MFN1KO.tif]

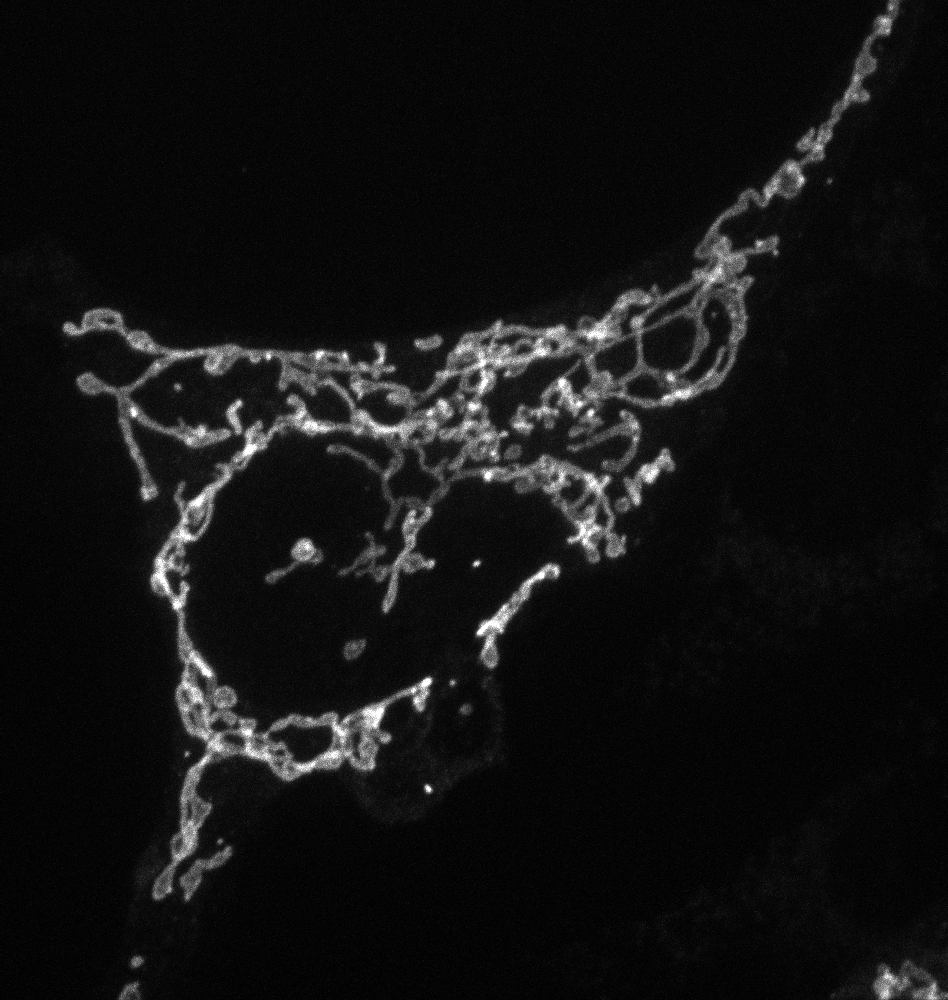

Supplement: Supplementary file 6 — Source Data EV Fig. 2 [file 44319_2023_9_MOESM6_ESM.zip › EV1/a/mfn1 ko/mfn1 oe/MAX_Experiment-2329 MFN1 KO MFN1 gfp.tif]

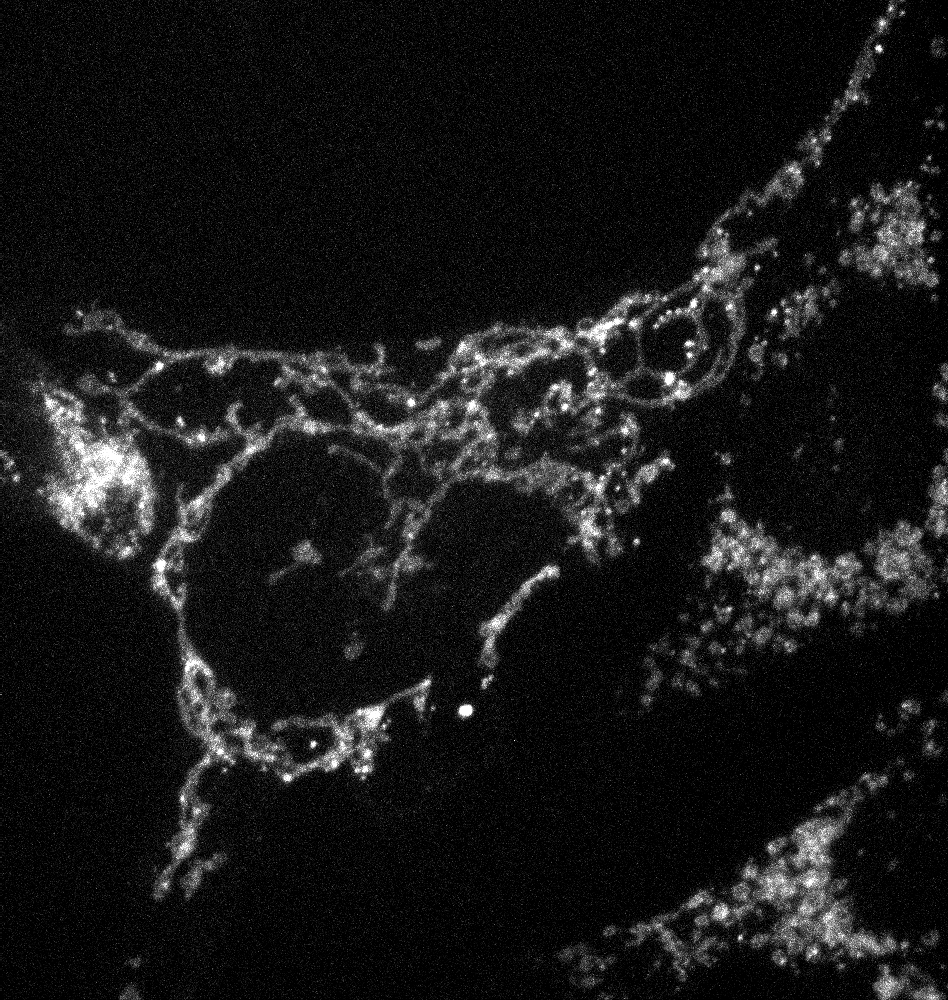

Supplement: Supplementary file 6 — Source Data EV Fig. 2 [file 44319_2023_9_MOESM6_ESM.zip › EV1/a/mfn1 ko/mfn1 oe/MAX_Experiment-2329 MFN1 KO MFN1 gfp.tif (RGB).tif]
