# Supplementary material for: MTCH2 cooperates with MFN2 and lysophosphatidic acid synthesis to sustain mitochondrial fusion
Source: EMBO Rep. 2023 Dec 14;25(1):8. doi: 10.1038/s44319-023-00009-1 (PMC10897490; doi:10.1038/s44319-023-00009-1)
Supplement: Supplementary file 6 — Source Data EV Fig. 2 [file 44319_2023_9_MOESM6_ESM.zip › EV1/d/UNCROPED BLOTS EV1D.pdf]

EV 1D FUSION FISSION PROTEINS IN MTCH2 KO

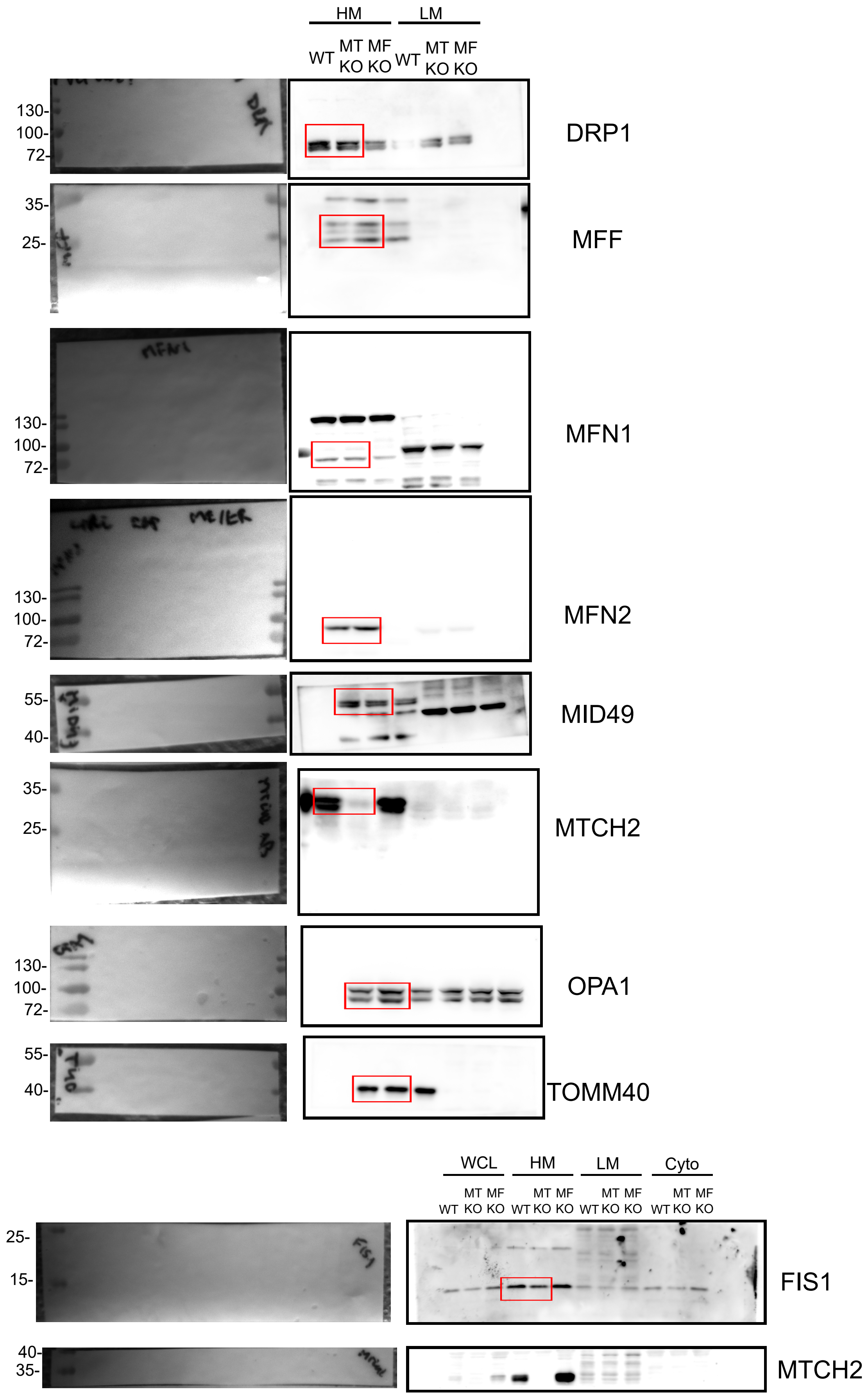

FIS 1 BLOT WAS OBTAINED WITH THE SAME SAMPLES USED FOR THE REST OF THE BLOTS
