# Supplementary figures and images for: MTCH2 cooperates with MFN2 and lysophosphatidic acid synthesis to sustain mitochondrial fusion (part 4 of 6)
Source: EMBO Rep. 2023 Dec 14;25(1):8. doi: 10.1038/s44319-023-00009-1 (PMC10897490; doi:10.1038/s44319-023-00009-1)

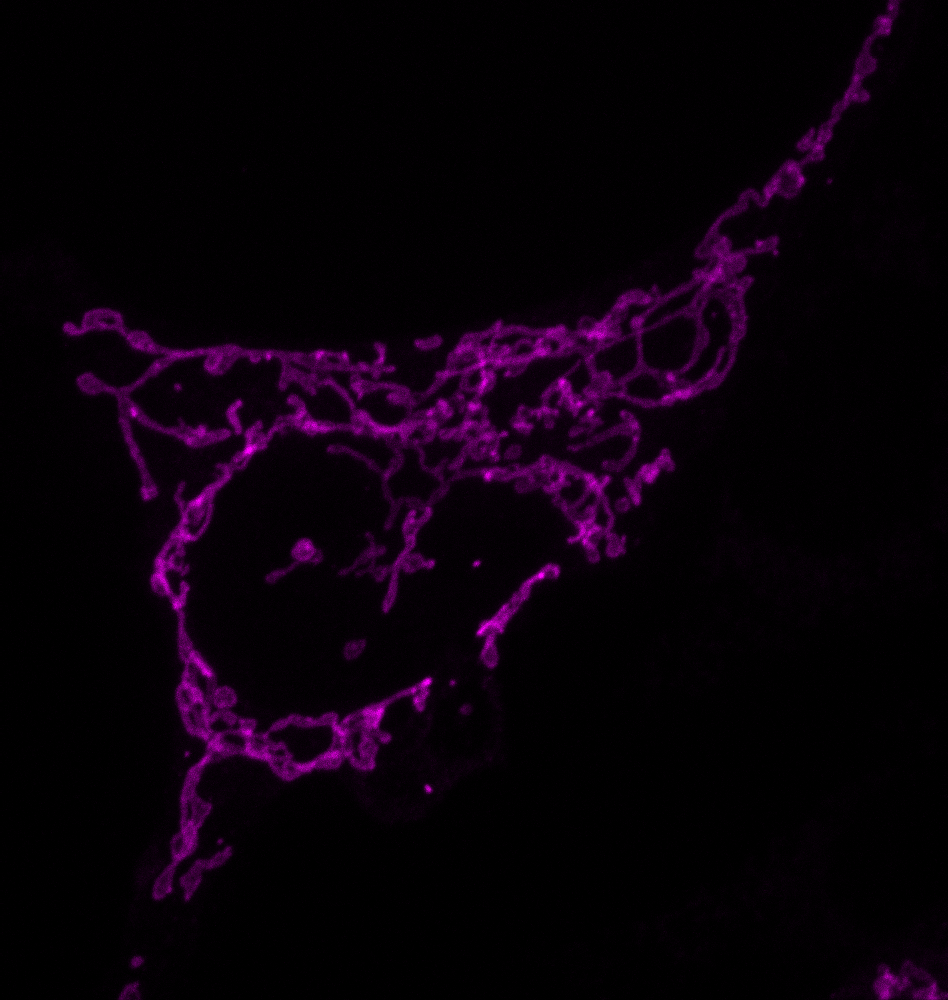

Supplement: Supplementary file 6 — Source Data EV Fig. 2 [file 44319_2023_9_MOESM6_ESM.zip › EV1/a/mfn1 ko/mfn1 oe/MAX_Experiment-2329 MFN1 KO MFN1 gfp.tif (RGB)ch1.tif]

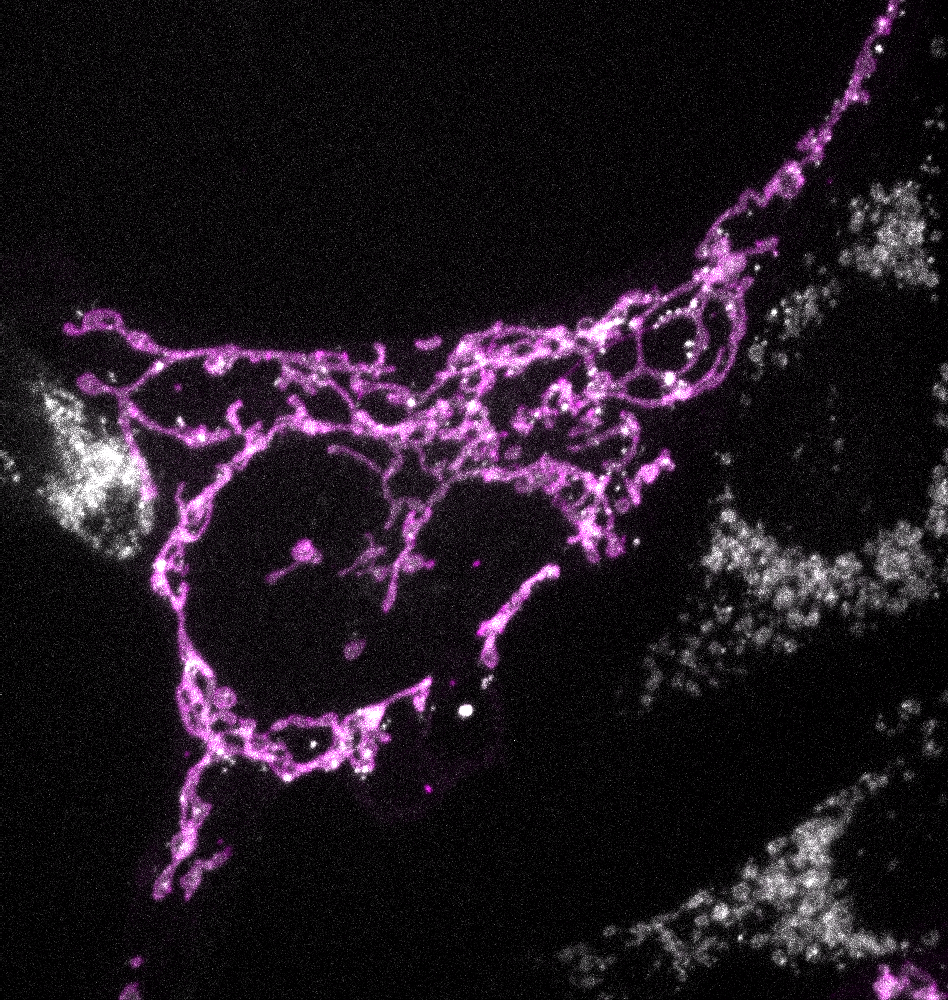

Supplement: Supplementary file 6 — Source Data EV Fig. 2 [file 44319_2023_9_MOESM6_ESM.zip › EV1/a/mfn1 ko/mfn1 oe/MAX_Experiment-2329 MFN1 KO MFN1 gfp.tif (RGB)comp.tif]

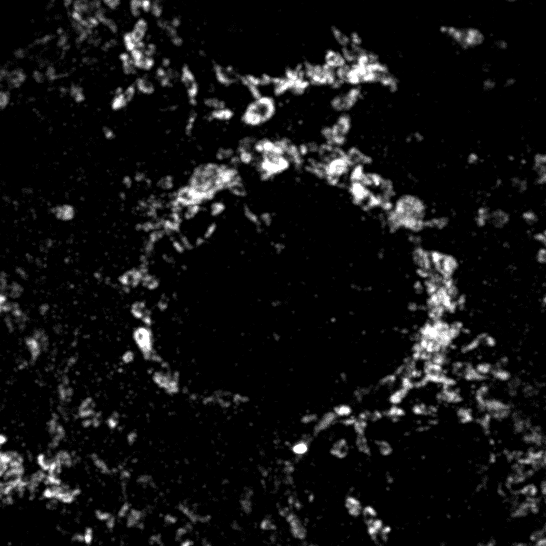

Supplement: Supplementary file 6 — Source Data EV Fig. 2 [file 44319_2023_9_MOESM6_ESM.zip › EV1/a/mtch2 ko/control/MAX_MEFs mtch2 ko control tom598 cytc633 dapi1_thumb_w1Con-mcherry_s1.TIF - Stage34 -1-1.tif]

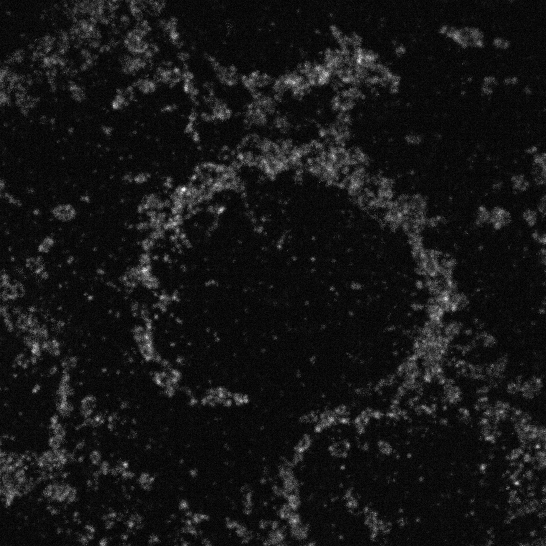

Supplement: Supplementary file 6 — Source Data EV Fig. 2 [file 44319_2023_9_MOESM6_ESM.zip › EV1/a/mtch2 ko/control/MAX_MEFs mtch2 ko control tom598 cytc633 dapi1_thumb_w1Con-mcherry_s1.TIF - Stage34 -1.tif]

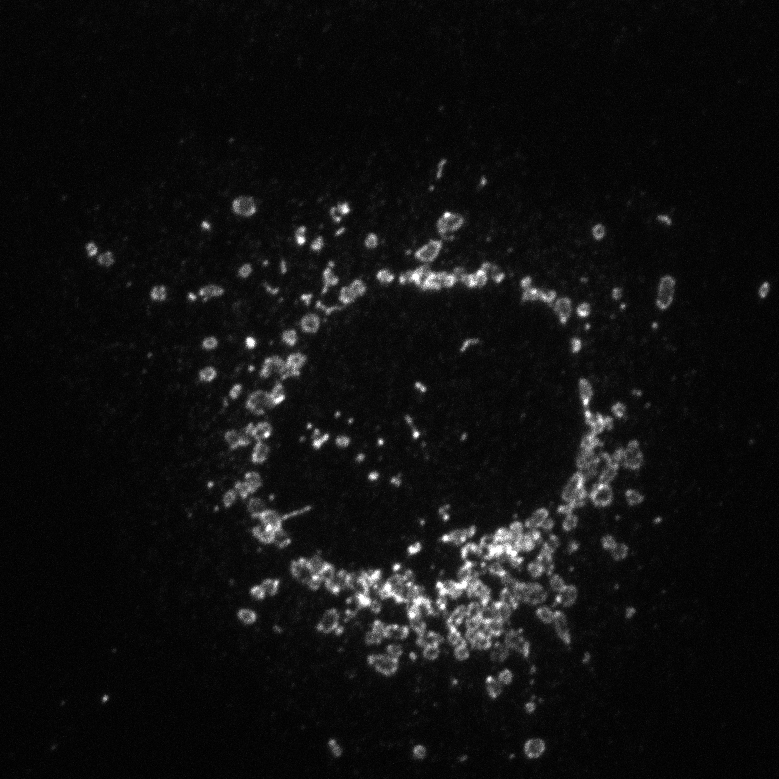

Supplement: Supplementary file 6 — Source Data EV Fig. 2 [file 44319_2023_9_MOESM6_ESM.zip › EV1/a/mtch2 ko/mfn1 oe/MAX_Experiment-1981-1-1 MTCH2 KO MFN1 GFP.tif]

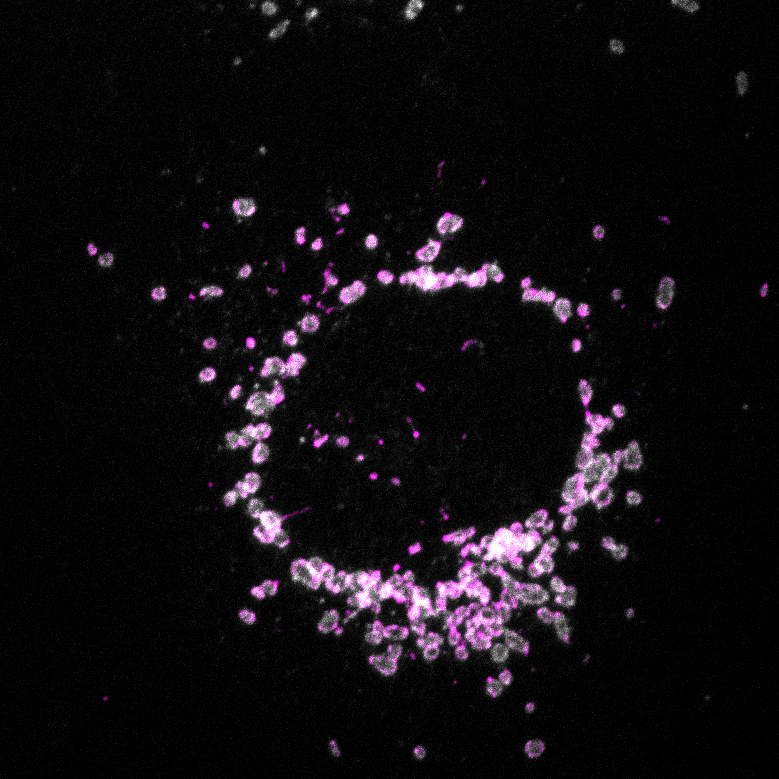

Supplement: Supplementary file 6 — Source Data EV Fig. 2 [file 44319_2023_9_MOESM6_ESM.zip › EV1/a/mtch2 ko/mfn1 oe/MAX_Experiment-1981-1-1 MTCH2 KO MFN1 GFP.tif (RGB) compo.tif]

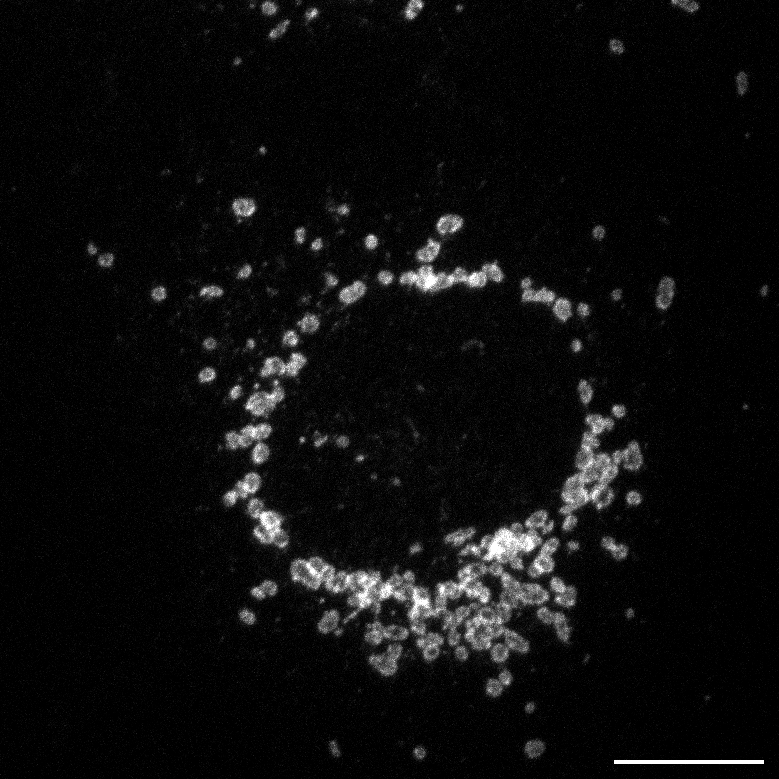

Supplement: Supplementary file 6 — Source Data EV Fig. 2 [file 44319_2023_9_MOESM6_ESM.zip › EV1/a/mtch2 ko/mfn1 oe/MAX_Experiment-1981-1-1 MTCH2 KO MFN1 GFP.tif (RGB)ch1-1scale bar.tif]

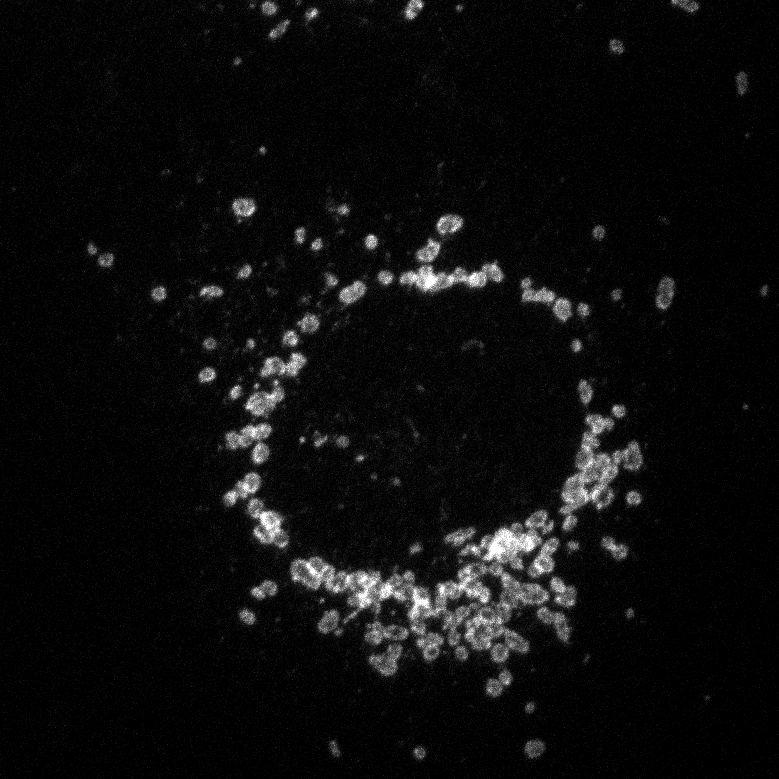

Supplement: Supplementary file 6 — Source Data EV Fig. 2 [file 44319_2023_9_MOESM6_ESM.zip › EV1/a/mtch2 ko/mfn1 oe/MAX_Experiment-1981-1-1 MTCH2 KO MFN1 GFP.tif (RGB)ch1.tif]

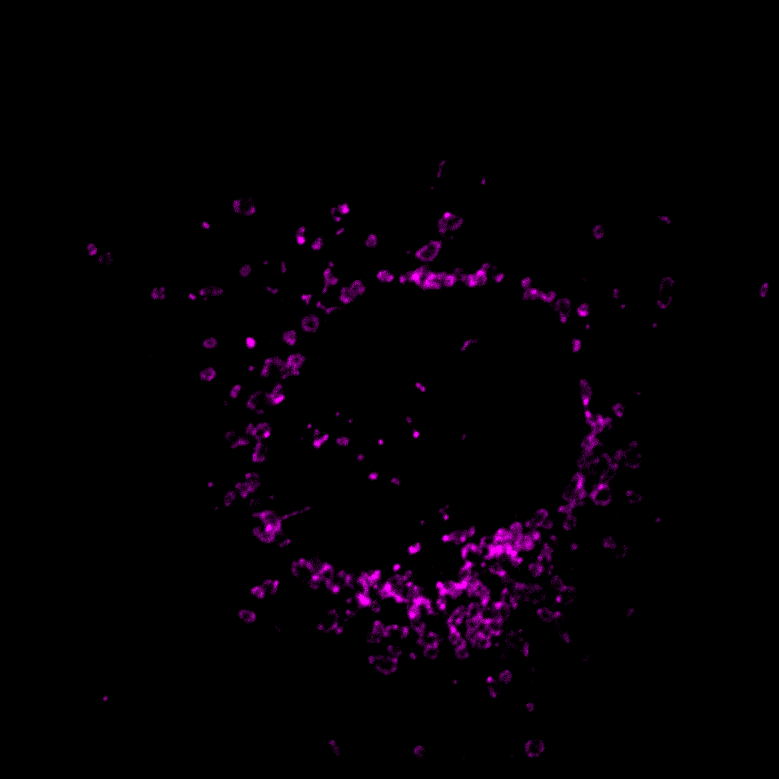

Supplement: Supplementary file 6 — Source Data EV Fig. 2 [file 44319_2023_9_MOESM6_ESM.zip › EV1/a/mtch2 ko/mfn1 oe/MAX_Experiment-1981-1-1 MTCH2 KO MFN1 GFP.tif (RGB)ch2.tif]

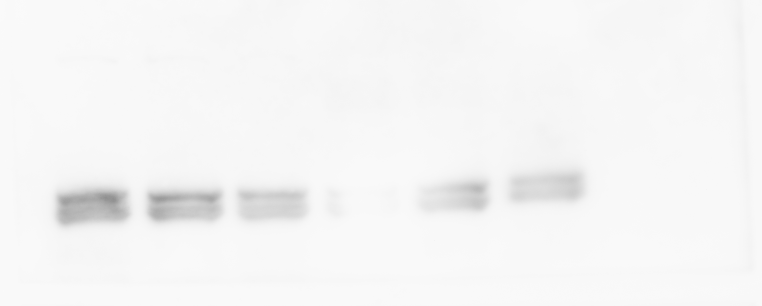

Supplement: Supplementary file 6 — Source Data EV Fig. 2 [file 44319_2023_9_MOESM6_ESM.zip › EV1/d/wb/drp1/drp1 blot.tif]

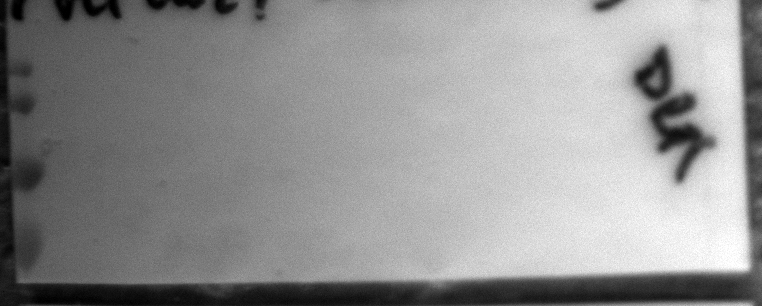

Supplement: Supplementary file 6 — Source Data EV Fig. 2 [file 44319_2023_9_MOESM6_ESM.zip › EV1/d/wb/drp1/drp1 memb.tif]

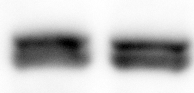

Supplement: Supplementary file 6 — Source Data EV Fig. 2 [file 44319_2023_9_MOESM6_ESM.zip › EV1/d/wb/drp1/DRP1-1.tif]

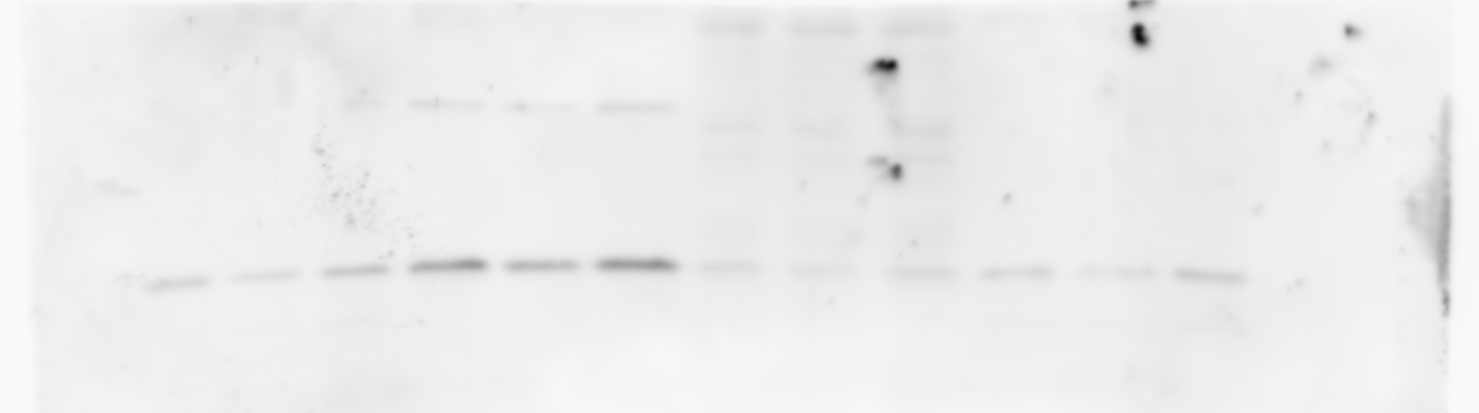

Supplement: Supplementary file 6 — Source Data EV Fig. 2 [file 44319_2023_9_MOESM6_ESM.zip › EV1/d/wb/fis1/fis1 blot.tif]

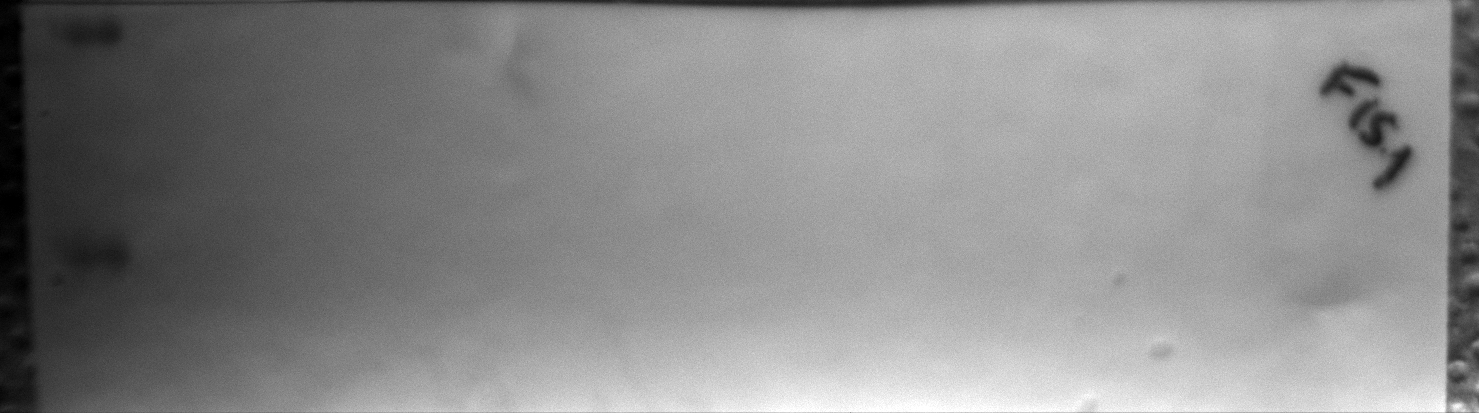

Supplement: Supplementary file 6 — Source Data EV Fig. 2 [file 44319_2023_9_MOESM6_ESM.zip › EV1/d/wb/fis1/fis1 memb.tif]

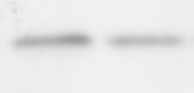

Supplement: Supplementary file 6 — Source Data EV Fig. 2 [file 44319_2023_9_MOESM6_ESM.zip › EV1/d/wb/fis1/fis1-1.tif]

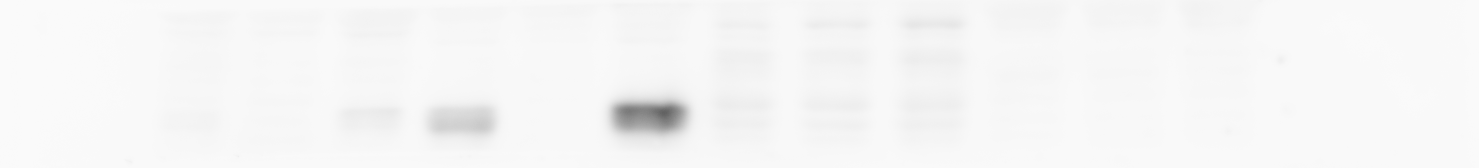

Supplement: Supplementary file 6 — Source Data EV Fig. 2 [file 44319_2023_9_MOESM6_ESM.zip › EV1/d/wb/fis1/mtch2 for fis1 blo.tif]

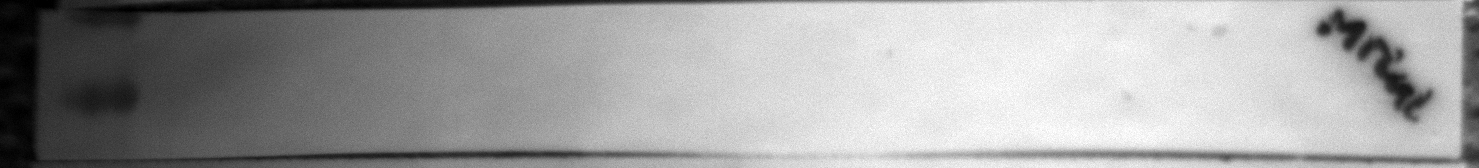

Supplement: Supplementary file 6 — Source Data EV Fig. 2 [file 44319_2023_9_MOESM6_ESM.zip › EV1/d/wb/fis1/mtch2 for fis1 memb.tif]

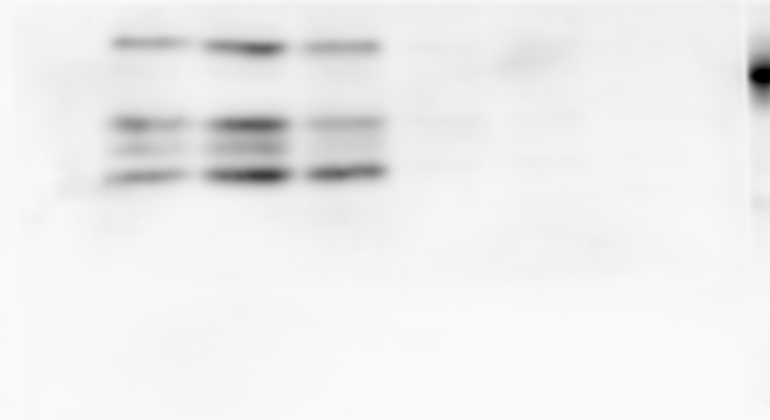

Supplement: Supplementary file 6 — Source Data EV Fig. 2 [file 44319_2023_9_MOESM6_ESM.zip › EV1/d/wb/mff/mff blot.tif]

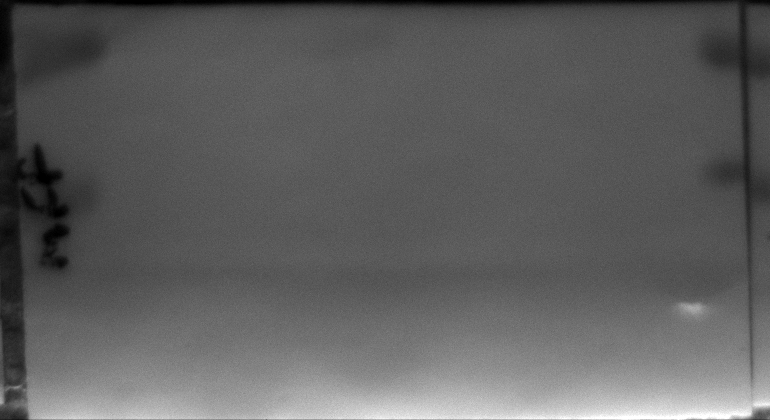

Supplement: Supplementary file 6 — Source Data EV Fig. 2 [file 44319_2023_9_MOESM6_ESM.zip › EV1/d/wb/mff/mff memb.tif]

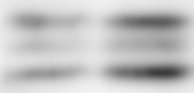

Supplement: Supplementary file 6 — Source Data EV Fig. 2 [file 44319_2023_9_MOESM6_ESM.zip › EV1/d/wb/mff/MFF-1.tif]

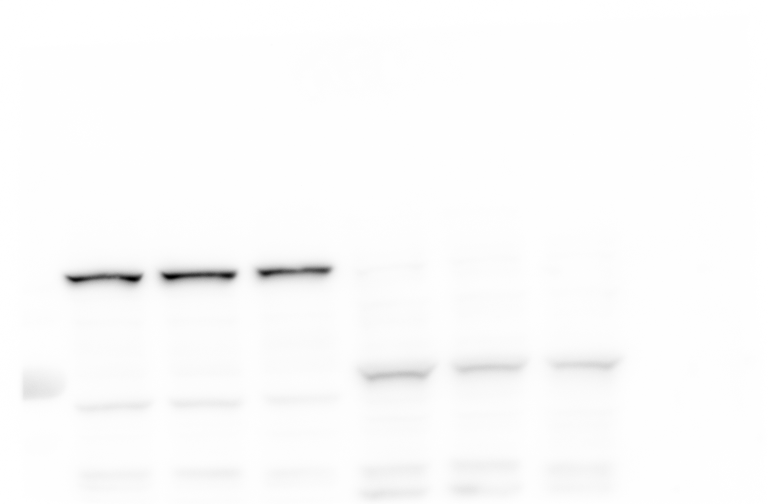

Supplement: Supplementary file 6 — Source Data EV Fig. 2 [file 44319_2023_9_MOESM6_ESM.zip › EV1/d/wb/mfn1/mfn1 blot.tif]

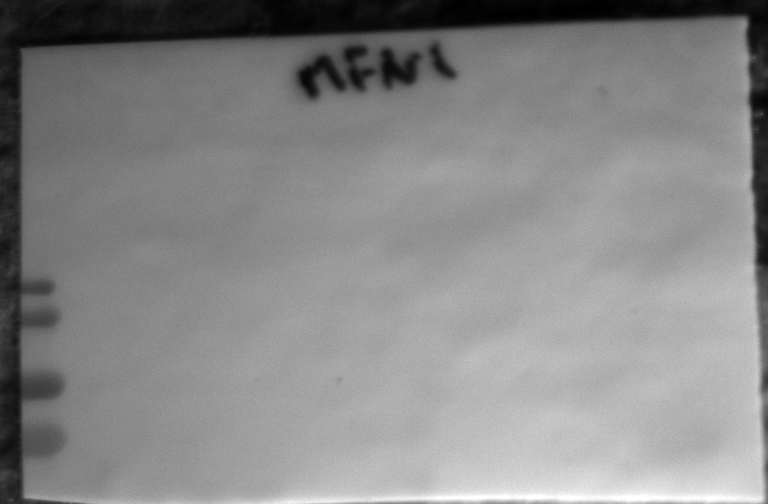

Supplement: Supplementary file 6 — Source Data EV Fig. 2 [file 44319_2023_9_MOESM6_ESM.zip › EV1/d/wb/mfn1/mfn1 membr.tif]

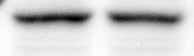

Supplement: Supplementary file 6 — Source Data EV Fig. 2 [file 44319_2023_9_MOESM6_ESM.zip › EV1/d/wb/mfn1/MFN1B-1.tif]

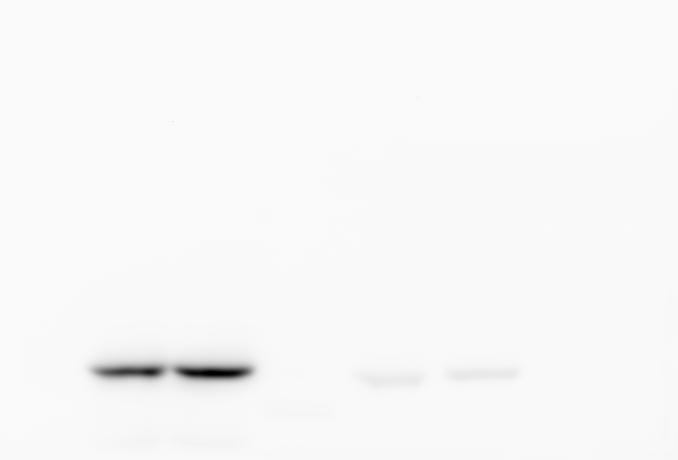

Supplement: Supplementary file 6 — Source Data EV Fig. 2 [file 44319_2023_9_MOESM6_ESM.zip › EV1/d/wb/mfn2/mfn2 blot.tif]

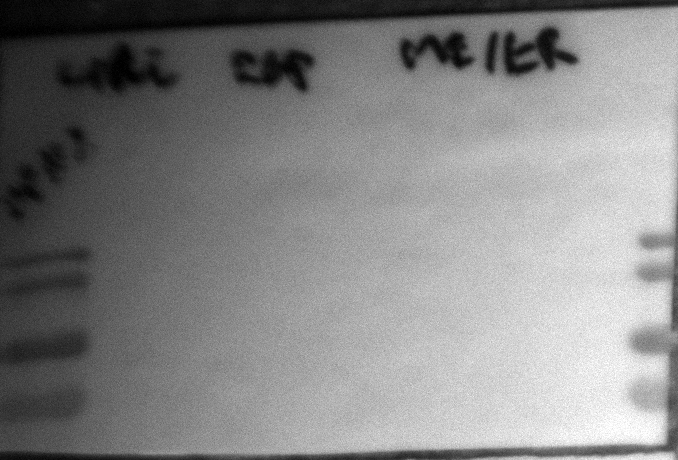

Supplement: Supplementary file 6 — Source Data EV Fig. 2 [file 44319_2023_9_MOESM6_ESM.zip › EV1/d/wb/mfn2/mfn2 memb.tif]

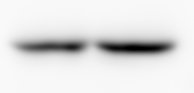

Supplement: Supplementary file 6 — Source Data EV Fig. 2 [file 44319_2023_9_MOESM6_ESM.zip › EV1/d/wb/mfn2/MFN2-1.tif]

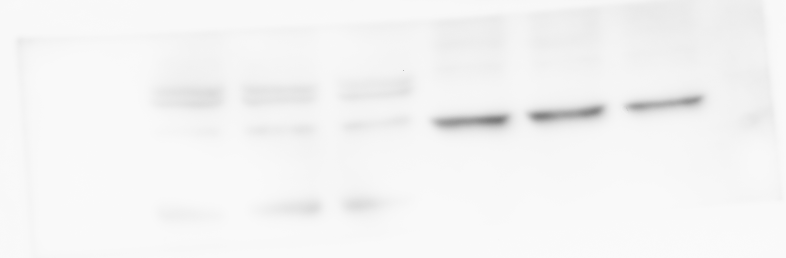

Supplement: Supplementary file 6 — Source Data EV Fig. 2 [file 44319_2023_9_MOESM6_ESM.zip › EV1/d/wb/mid49/mid49 blot.tif]

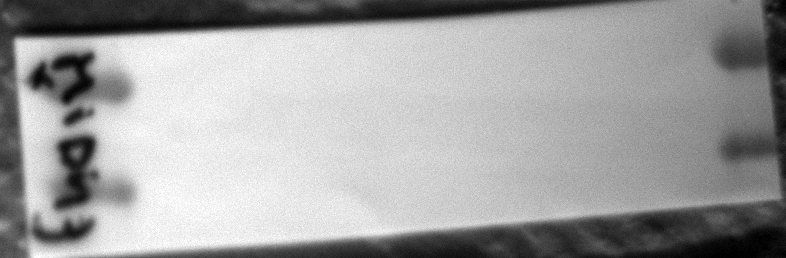

Supplement: Supplementary file 6 — Source Data EV Fig. 2 [file 44319_2023_9_MOESM6_ESM.zip › EV1/d/wb/mid49/mid49 memb.tif]

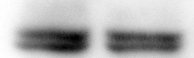

Supplement: Supplementary file 6 — Source Data EV Fig. 2 [file 44319_2023_9_MOESM6_ESM.zip › EV1/d/wb/mid49/MID49-1.tif]

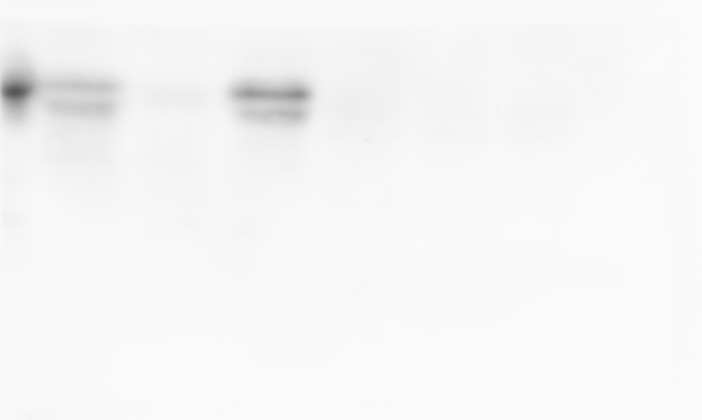

Supplement: Supplementary file 6 — Source Data EV Fig. 2 [file 44319_2023_9_MOESM6_ESM.zip › EV1/d/wb/mtch2/mtch2 blot.tif]

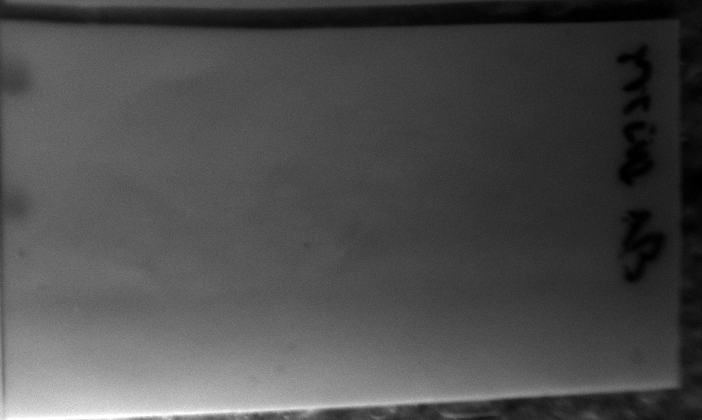

Supplement: Supplementary file 6 — Source Data EV Fig. 2 [file 44319_2023_9_MOESM6_ESM.zip › EV1/d/wb/mtch2/mtch2 memb.tif]

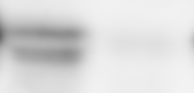

Supplement: Supplementary file 6 — Source Data EV Fig. 2 [file 44319_2023_9_MOESM6_ESM.zip › EV1/d/wb/mtch2/MTCH2-1.tif]

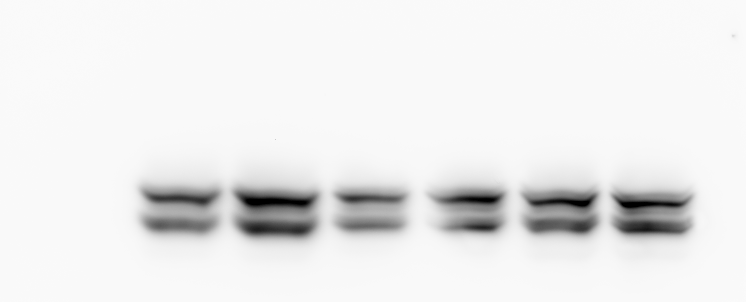

Supplement: Supplementary file 6 — Source Data EV Fig. 2 [file 44319_2023_9_MOESM6_ESM.zip › EV1/d/wb/opa1/opa all long.tif]

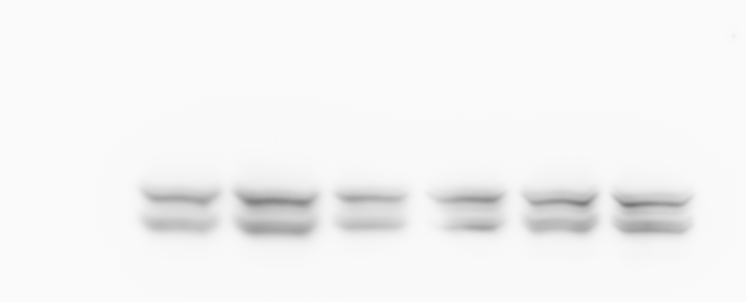

Supplement: Supplementary file 6 — Source Data EV Fig. 2 [file 44319_2023_9_MOESM6_ESM.zip › EV1/d/wb/opa1/opa1 all.tif]

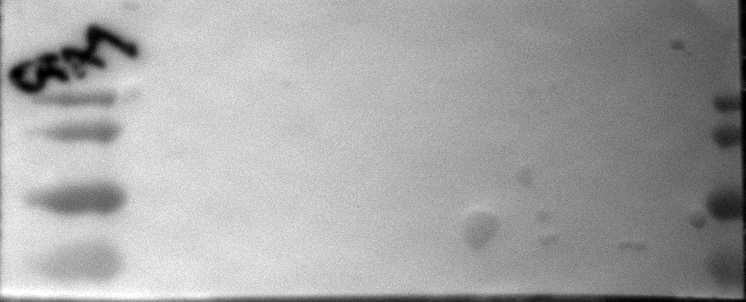

Supplement: Supplementary file 6 — Source Data EV Fig. 2 [file 44319_2023_9_MOESM6_ESM.zip › EV1/d/wb/opa1/opa1 memb.tif]

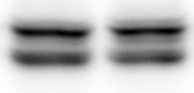

Supplement: Supplementary file 6 — Source Data EV Fig. 2 [file 44319_2023_9_MOESM6_ESM.zip › EV1/d/wb/opa1/OPA1-1.tif]

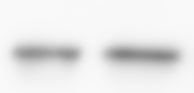

Supplement: Supplementary file 6 — Source Data EV Fig. 2 [file 44319_2023_9_MOESM6_ESM.zip › EV1/d/wb/tomm40/TOM40.tifjh-1.tif]

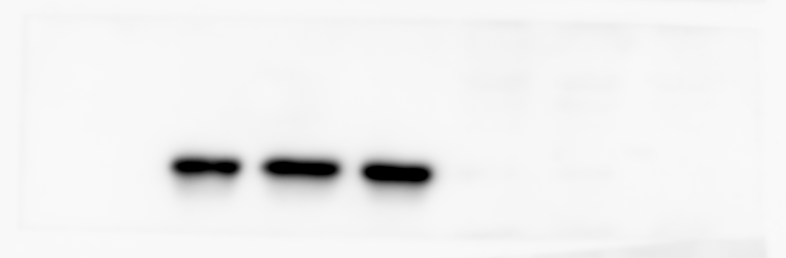

Supplement: Supplementary file 6 — Source Data EV Fig. 2 [file 44319_2023_9_MOESM6_ESM.zip › EV1/d/wb/tomm40/tomm40 blot.tif]

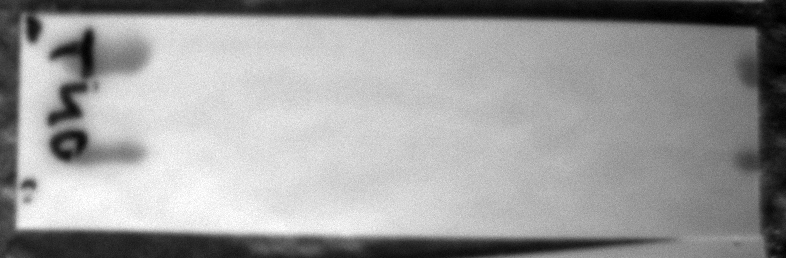

Supplement: Supplementary file 6 — Source Data EV Fig. 2 [file 44319_2023_9_MOESM6_ESM.zip › EV1/d/wb/tomm40/tomm40 memb.tif]

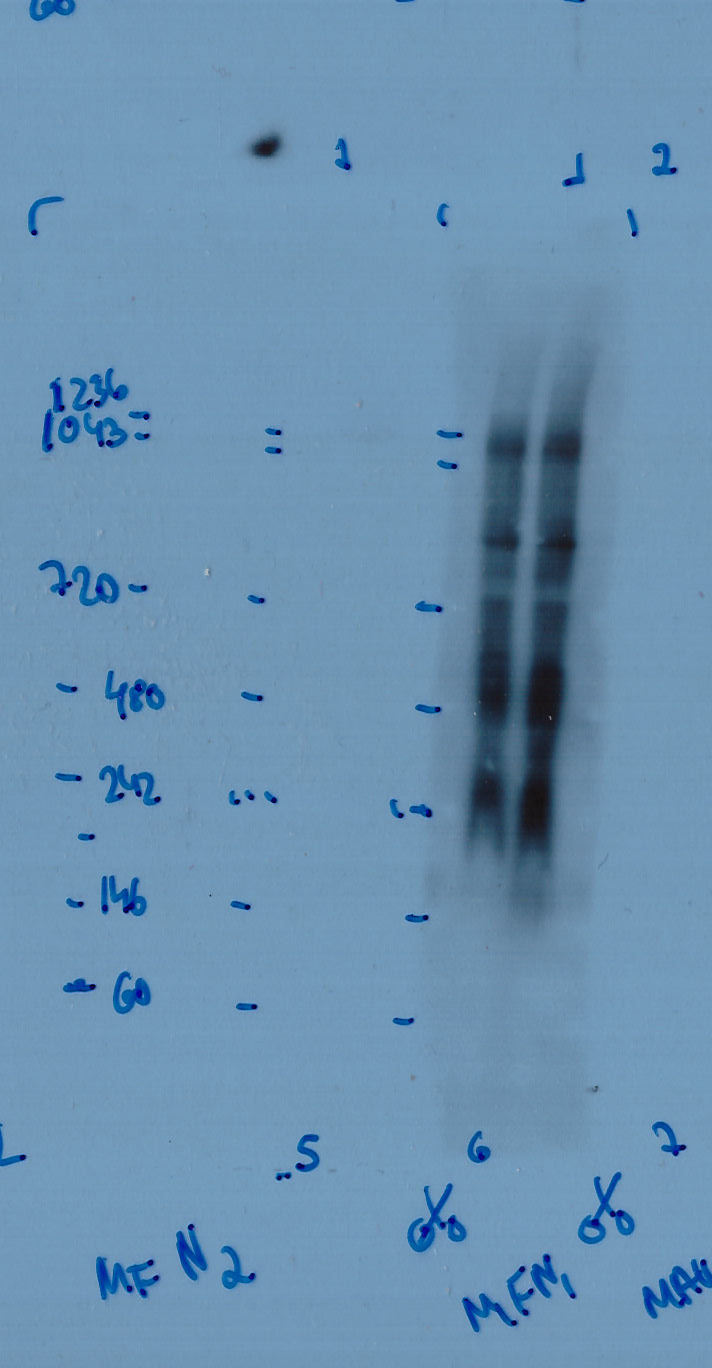

Supplement: Supplementary file 6 — Source Data EV Fig. 2 [file 44319_2023_9_MOESM6_ESM.zip › EV1/e/BLOTS/MFN1/MFN1 BLOT.tif]

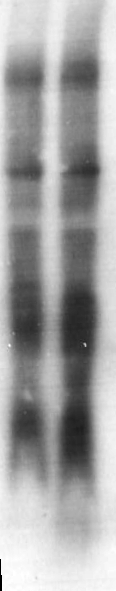

Supplement: Supplementary file 6 — Source Data EV Fig. 2 [file 44319_2023_9_MOESM6_ESM.zip › EV1/e/BLOTS/MFN1/mfn122.tif]

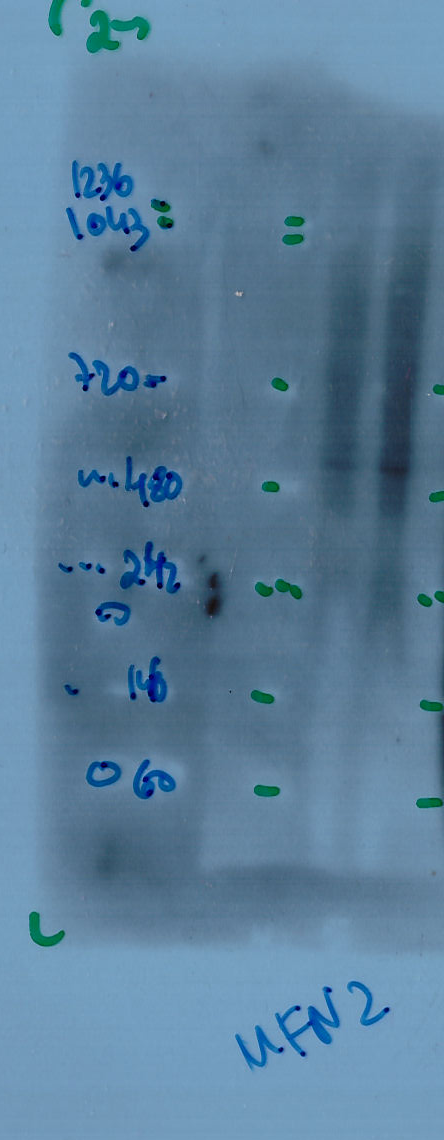

Supplement: Supplementary file 6 — Source Data EV Fig. 2 [file 44319_2023_9_MOESM6_ESM.zip › EV1/e/BLOTS/MFN2/MFN2 BLOT.tif]

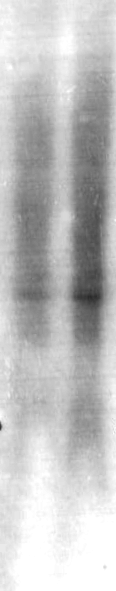

Supplement: Supplementary file 6 — Source Data EV Fig. 2 [file 44319_2023_9_MOESM6_ESM.zip › EV1/e/BLOTS/MFN2/mfn222.tif]

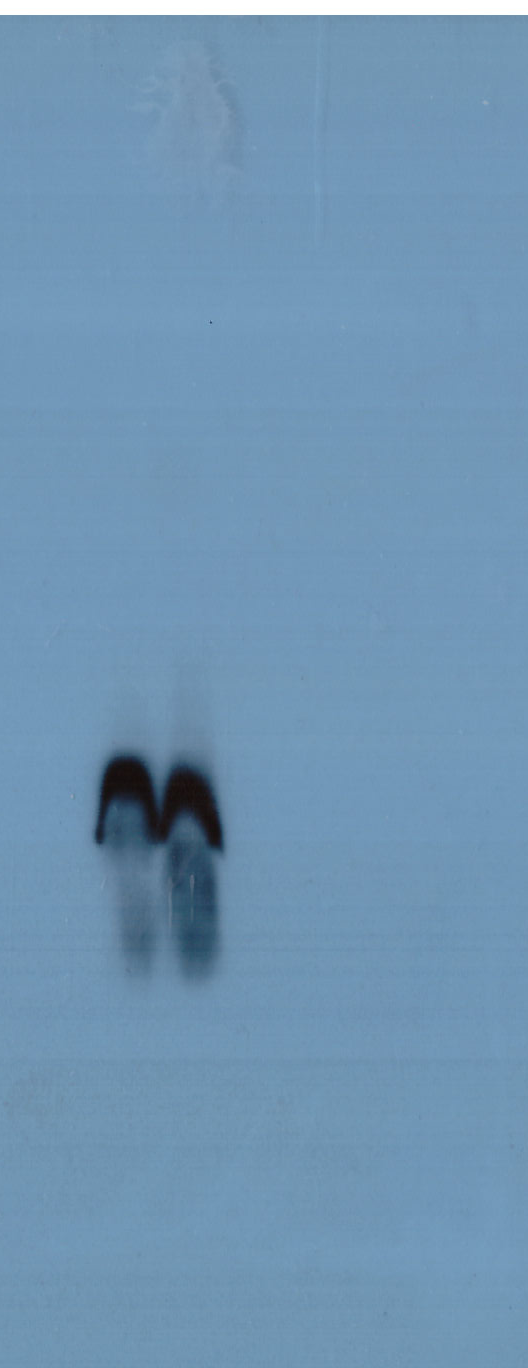

Supplement: Supplementary file 6 — Source Data EV Fig. 2 [file 44319_2023_9_MOESM6_ESM.zip › EV1/e/BLOTS/SDHA/COMPII BLOT USED.tif]

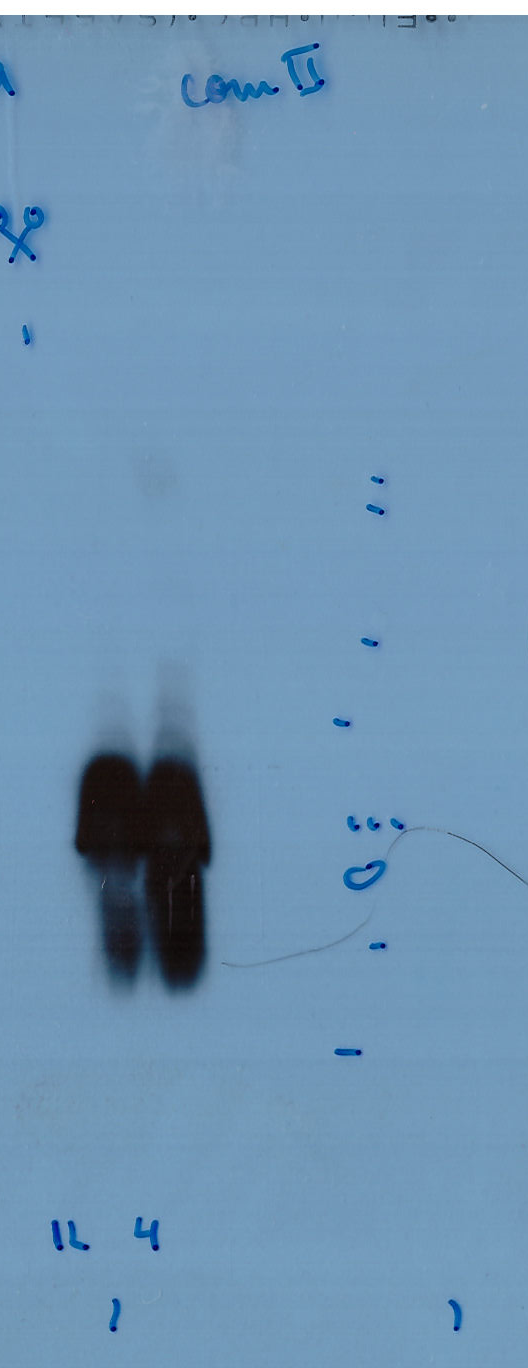

Supplement: Supplementary file 6 — Source Data EV Fig. 2 [file 44319_2023_9_MOESM6_ESM.zip › EV1/e/BLOTS/SDHA/COMPii BLOT.tif]

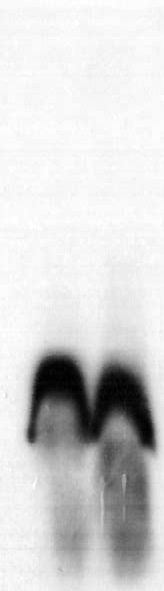

Supplement: Supplementary file 6 — Source Data EV Fig. 2 [file 44319_2023_9_MOESM6_ESM.zip › EV1/e/BLOTS/SDHA/sdha222.tif]

# EV 1D BNGE OF MFN1 AND MFN2

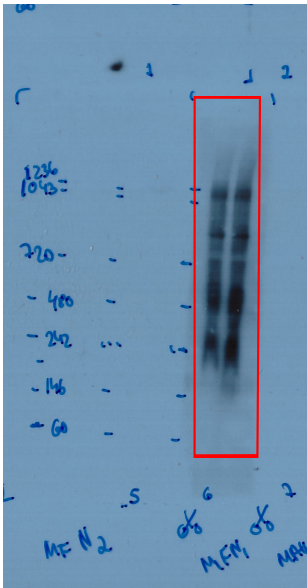

MFN1

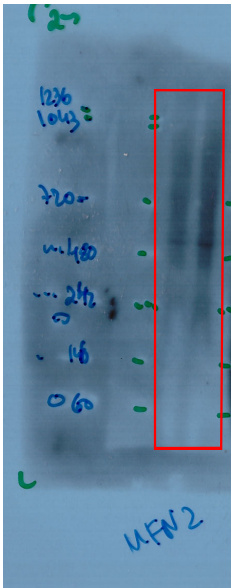

MFN2

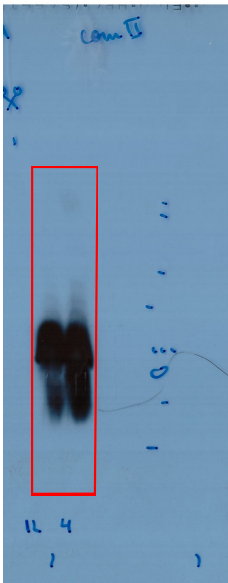

SDHA

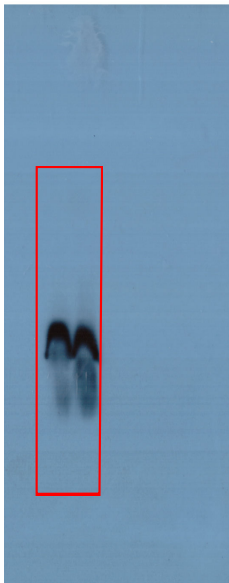

SDHA

Supplement: Supplementary file 6 — Source Data EV Fig. 2 [file 44319_2023_9_MOESM6_ESM.zip › EV1/e/BLOTS/UNCROPED BLOTS EV1E.pdf]

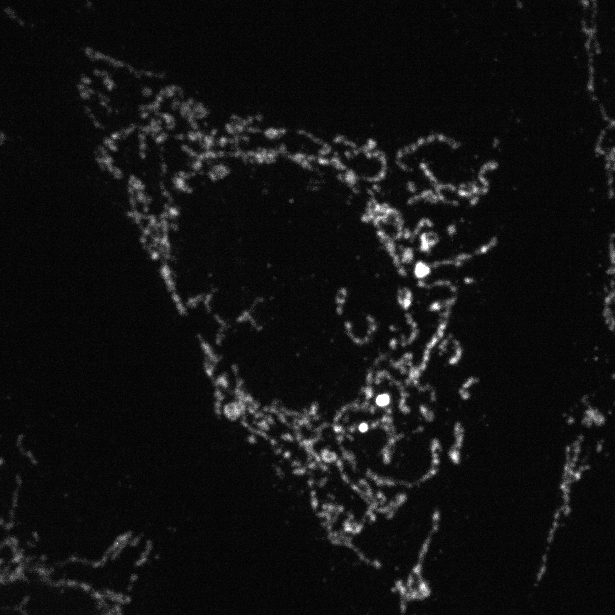

Supplement: Supplementary file 6 — Source Data EV Fig. 2 [file 44319_2023_9_MOESM6_ESM.zip › EV1/g/IMAGES/wt mtch1 si/MAX_mefs mtch1 siRNA 3d cytc real_thumb_w1Con-mcherry_s1.TIF - Stage24 -1.tif]

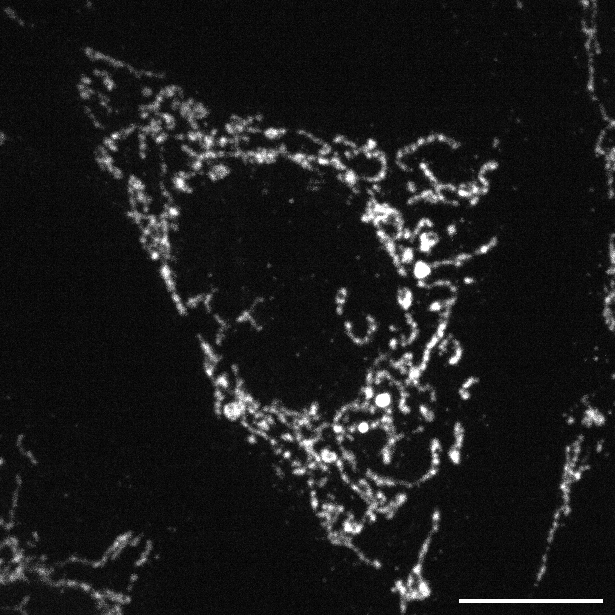

Supplement: Supplementary file 6 — Source Data EV Fig. 2 [file 44319_2023_9_MOESM6_ESM.zip › EV1/g/IMAGES/wt mtch1 si/MAX_mefs mtch1 siRNA 3d cytc real_thumb_w1Con-mcherry_s1.TIF - Stage24 -2 scale.tif]

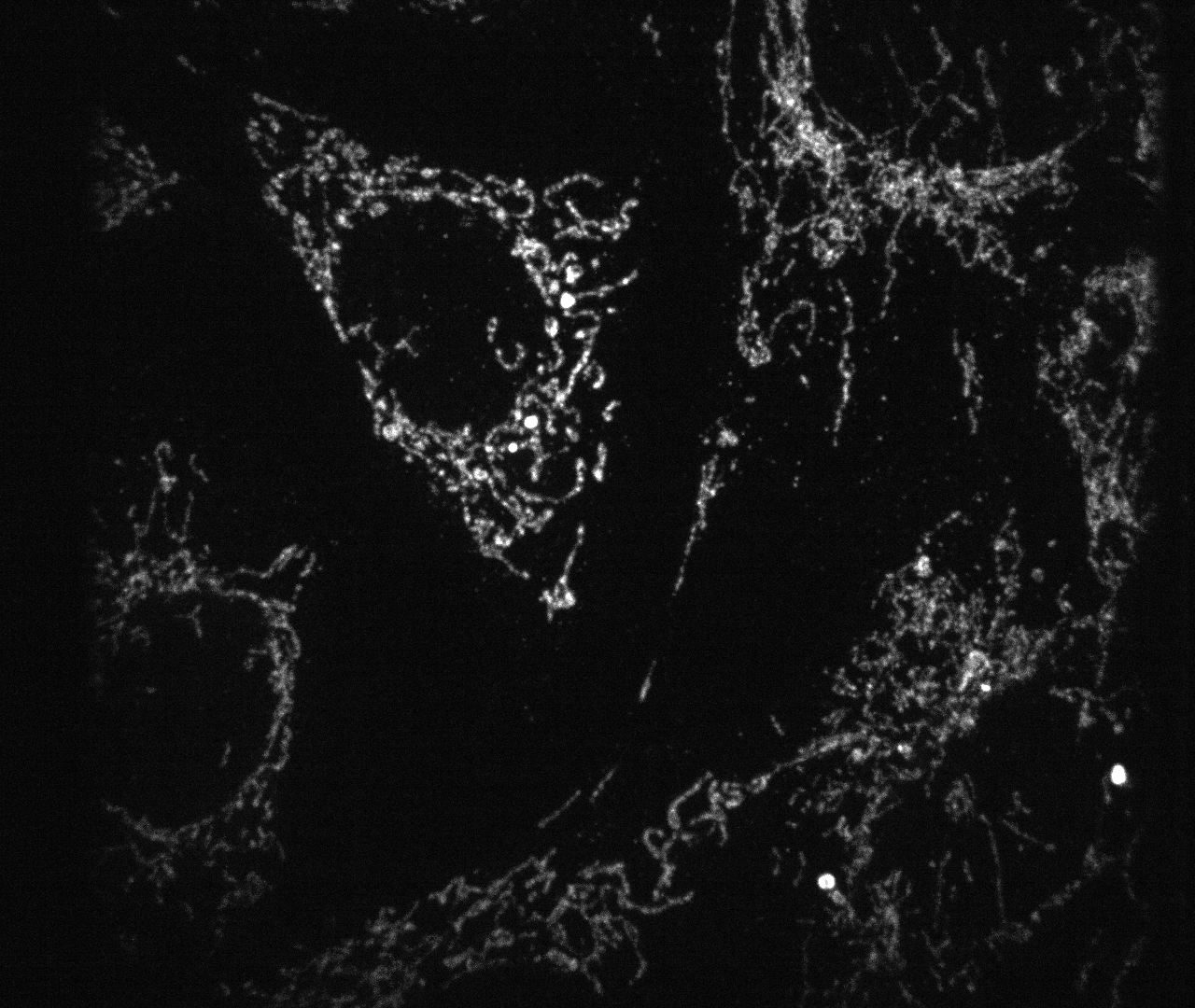

Supplement: Supplementary file 6 — Source Data EV Fig. 2 [file 44319_2023_9_MOESM6_ESM.zip › EV1/g/IMAGES/wt mtch1 si/MAX_mefs mtch1 siRNA 3d cytc real_thumb_w1Con-mcherry_s1.TIF - Stage24 .tif]

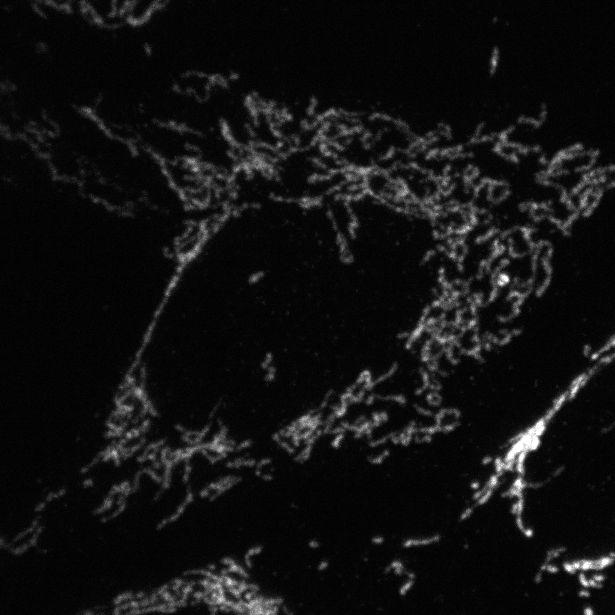

Supplement: Supplementary file 6 — Source Data EV Fig. 2 [file 44319_2023_9_MOESM6_ESM.zip › EV1/g/IMAGES/wt nt/MAX_mefs nt siRNA 3d cytc real_thumb_w1Con-mcherry_s1.TIF - Stage3.tif]

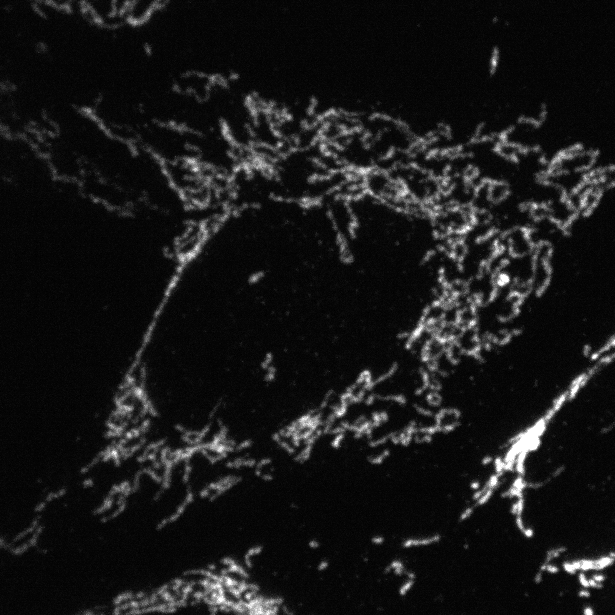

Supplement: Supplementary file 6 — Source Data EV Fig. 2 [file 44319_2023_9_MOESM6_ESM.zip › EV1/g/IMAGES/wt nt/MAX_mefs nt siRNA 3d cytc real_thumb_w1Con-mcherry_s1.TIF - Stage3rgb.tif]

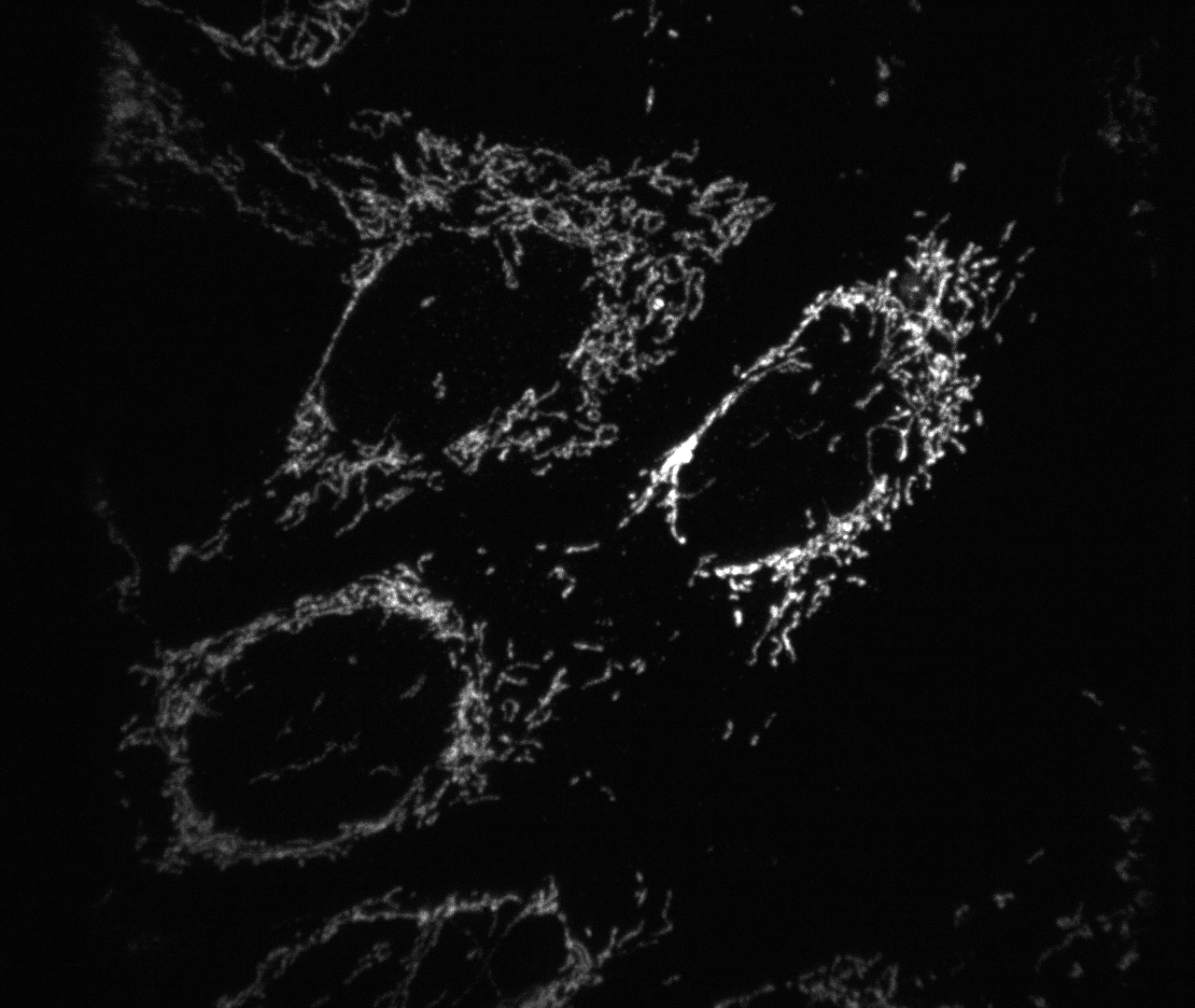

Supplement: Supplementary file 6 — Source Data EV Fig. 2 [file 44319_2023_9_MOESM6_ESM.zip › EV1/g/IMAGES/wt nt/mefs nt siRNA 3d cytc real_thumb_w1Con-mcherry_s1.TIF - Stage3.tif]

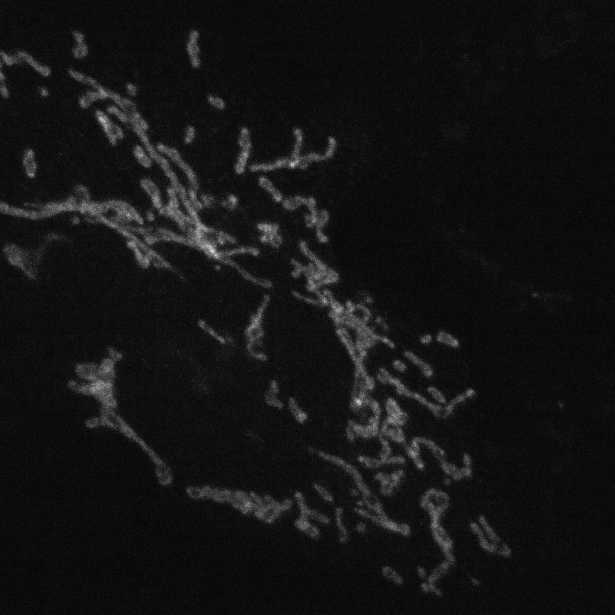

Supplement: Supplementary file 6 — Source Data EV Fig. 2 [file 44319_2023_9_MOESM6_ESM.zip › EV1/j/IMAGES/MTCH2 D189R/MAX_Mtch2 ko mtch2 d189r gfp pdh cy3 t40cy5 im35_thumb_w1Con-GFP-1-1.tif]

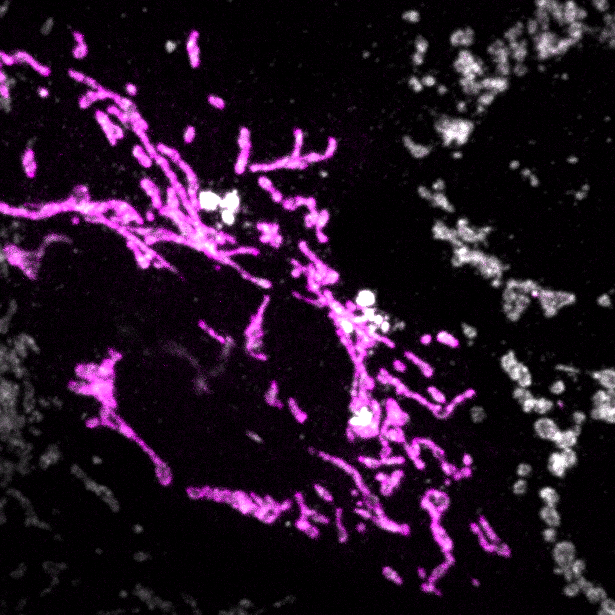

Supplement: Supplementary file 6 — Source Data EV Fig. 2 [file 44319_2023_9_MOESM6_ESM.zip › EV1/j/IMAGES/MTCH2 D189R/MAX_Mtch2 ko mtch2 d189r gfp pdh cy3 t40cy5 im35_thumb_w1Con-GFP-1-1.tif (RGB).tif]

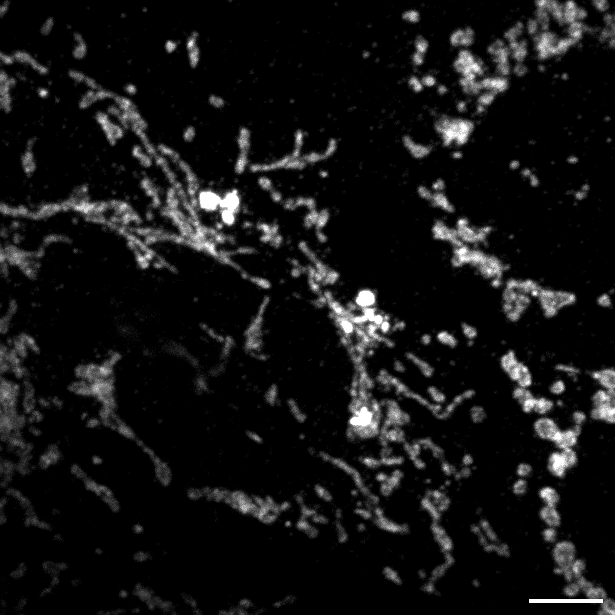

Supplement: Supplementary file 6 — Source Data EV Fig. 2 [file 44319_2023_9_MOESM6_ESM.zip › EV1/j/IMAGES/MTCH2 D189R/MAX_Mtch2 ko mtch2 d189r gfp pdh cy3 t40cy5 im35_thumb_w1Con-GFP-1-1.tif (RGB)F-1SCALE BAR.tif]

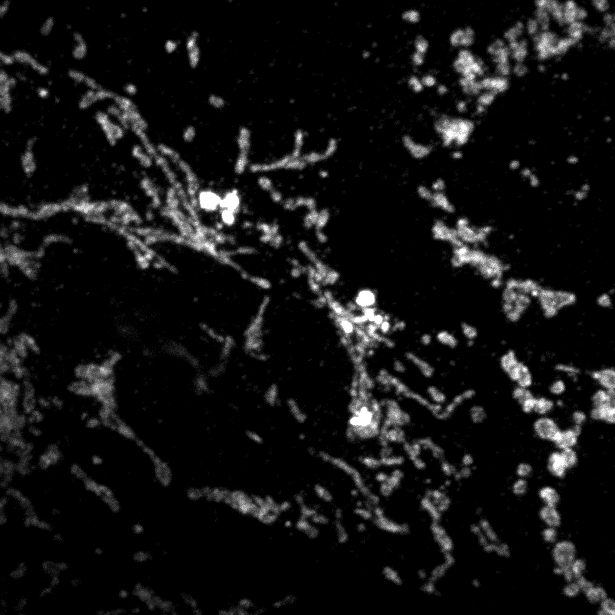

Supplement: Supplementary file 6 — Source Data EV Fig. 2 [file 44319_2023_9_MOESM6_ESM.zip › EV1/j/IMAGES/MTCH2 D189R/MAX_Mtch2 ko mtch2 d189r gfp pdh cy3 t40cy5 im35_thumb_w1Con-GFP-1-1.tif (RGB)F.tif]

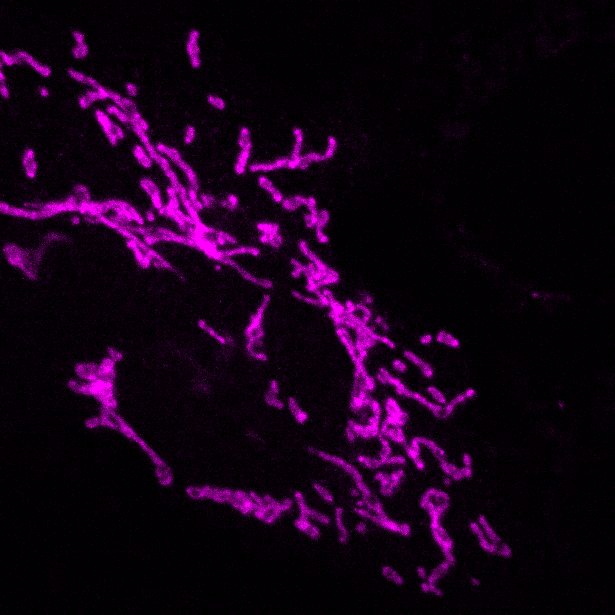

Supplement: Supplementary file 6 — Source Data EV Fig. 2 [file 44319_2023_9_MOESM6_ESM.zip › EV1/j/IMAGES/MTCH2 D189R/MAX_Mtch2 ko mtch2 d189r gfp pdh cy3 t40cy5 im35_thumb_w1Con-GFP-1-1.tif (RGB)FF.tif]

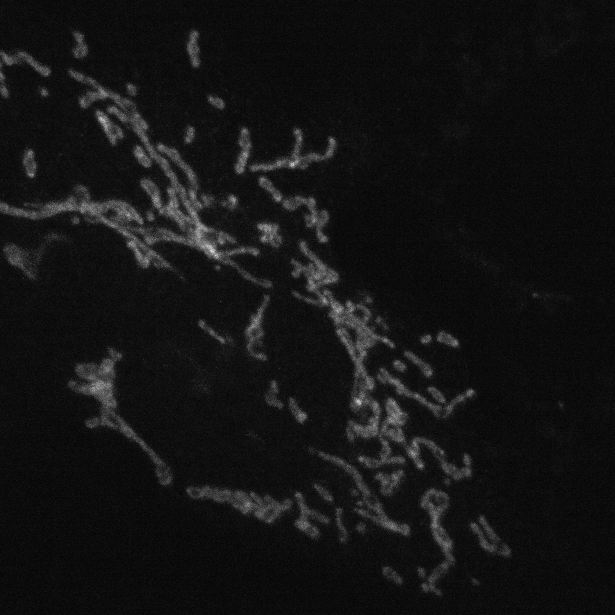

Supplement: Supplementary file 6 — Source Data EV Fig. 2 [file 44319_2023_9_MOESM6_ESM.zip › EV1/j/IMAGES/MTCH2 D189R/MAX_Mtch2 ko mtch2 d189r gfp pdh cy3 t40cy5 im35_thumb_w1Con-GFP-1.tif]

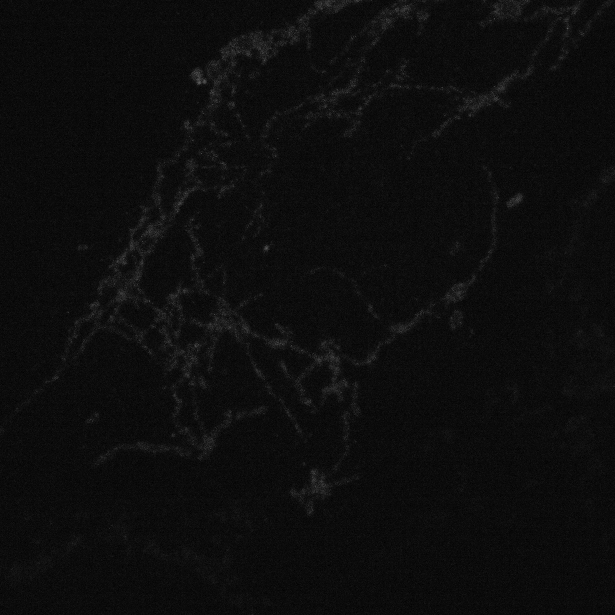

Supplement: Supplementary file 6 — Source Data EV Fig. 2 [file 44319_2023_9_MOESM6_ESM.zip › EV1/j/IMAGES/MTCH2 K25E/MAX_Mtch2 ko mtch2 k25e gfp pdh cy3 t40cy5 im17_thumb_w1Con-GFP-1.tif]

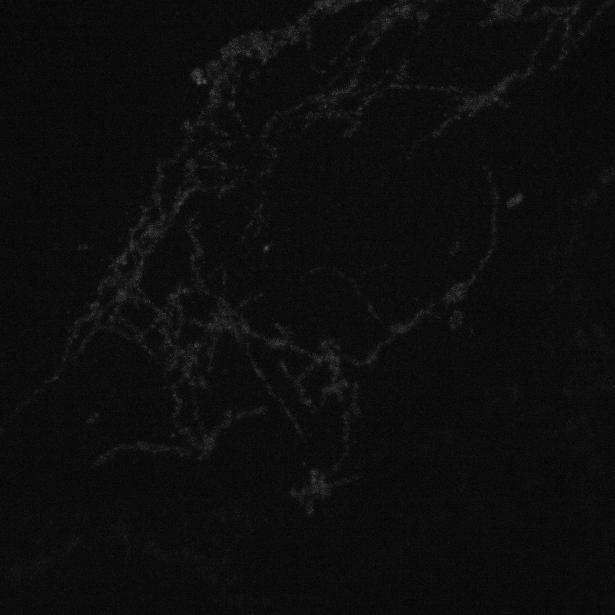

Supplement: Supplementary file 6 — Source Data EV Fig. 2 [file 44319_2023_9_MOESM6_ESM.zip › EV1/j/IMAGES/MTCH2 K25E/MAX_Mtch2 ko mtch2 k25e gfp pdh cy3 t40cy5 im17_thumb_w1Con-GFP-1B.tif]

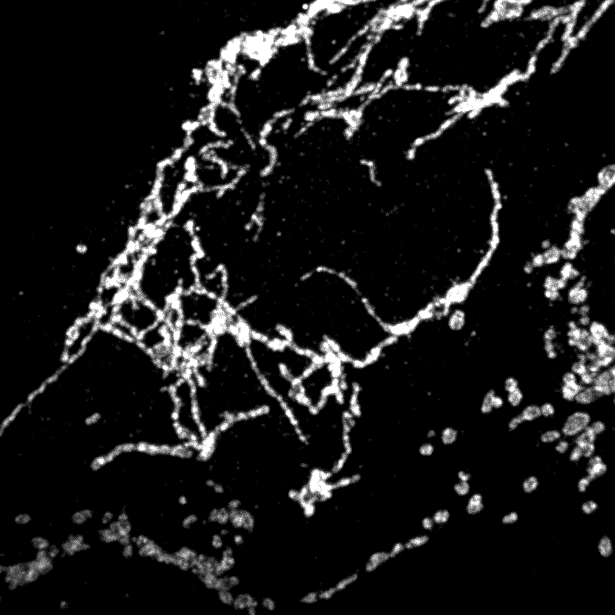

Supplement: Supplementary file 6 — Source Data EV Fig. 2 [file 44319_2023_9_MOESM6_ESM.zip › EV1/j/IMAGES/MTCH2 K25E/MAX_Mtch2 ko mtch2 k25e gfp pdh cy3 t40cy5 im17_thumb_w1Con-GFP-1B.tif (RGB).tif]

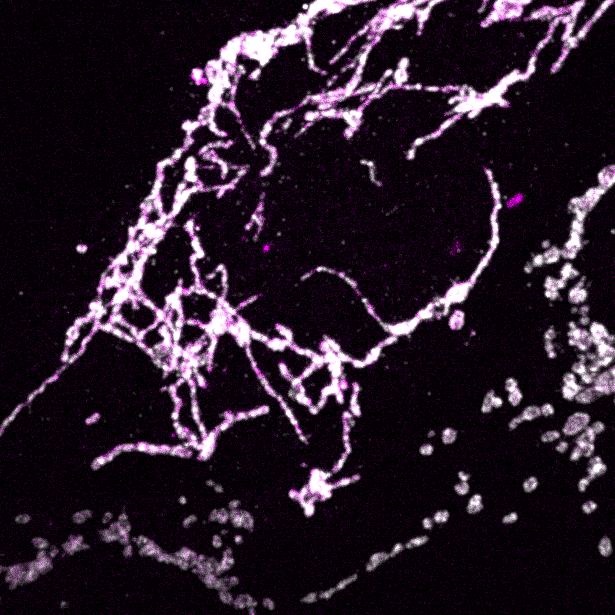

Supplement: Supplementary file 6 — Source Data EV Fig. 2 [file 44319_2023_9_MOESM6_ESM.zip › EV1/j/IMAGES/MTCH2 K25E/MAX_Mtch2 ko mtch2 k25e gfp pdh cy3 t40cy5 im17_thumb_w1Con-GFP-1B.tif (RGB)D.tif]

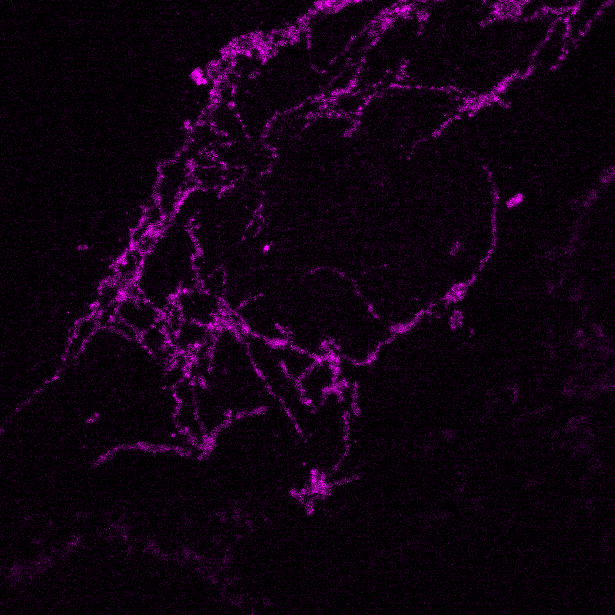

Supplement: Supplementary file 6 — Source Data EV Fig. 2 [file 44319_2023_9_MOESM6_ESM.zip › EV1/j/IMAGES/MTCH2 K25E/MAX_Mtch2 ko mtch2 k25e gfp pdh cy3 t40cy5 im17_thumb_w1Con-GFP-1B.tif (RGB)DD.tif]

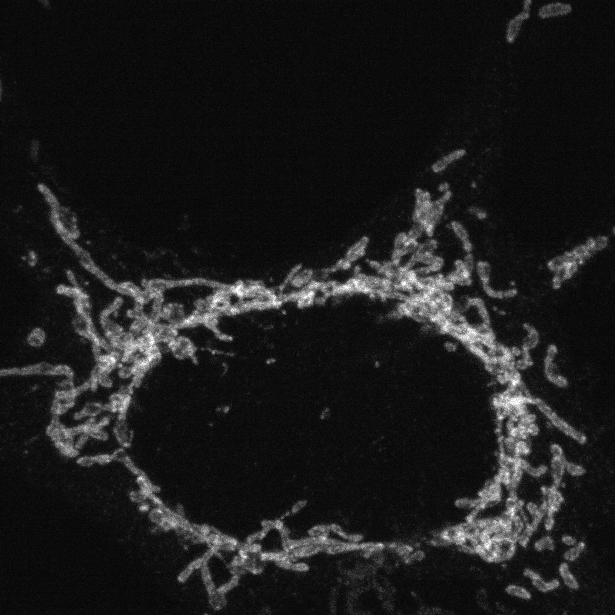

Supplement: Supplementary file 6 — Source Data EV Fig. 2 [file 44319_2023_9_MOESM6_ESM.zip › EV1/j/IMAGES/MTCH2 WT/MAX_Mtch2 ko mtch2 wt gfp pdh cy3 t40cy5 im26_thumb_w1Con-GFP-1.tif]

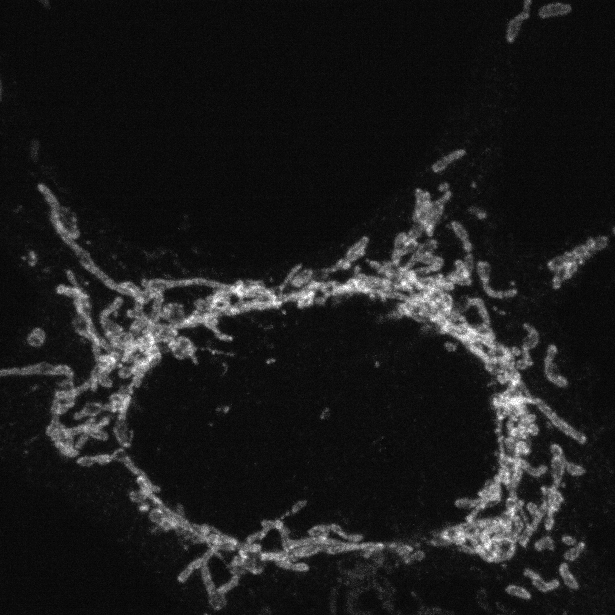

Supplement: Supplementary file 6 — Source Data EV Fig. 2 [file 44319_2023_9_MOESM6_ESM.zip › EV1/j/IMAGES/MTCH2 WT/MAX_Mtch2 ko mtch2 wt gfp pdh cy3 t40cy5 im26_thumb_w1Con-GFP-1D.tif]

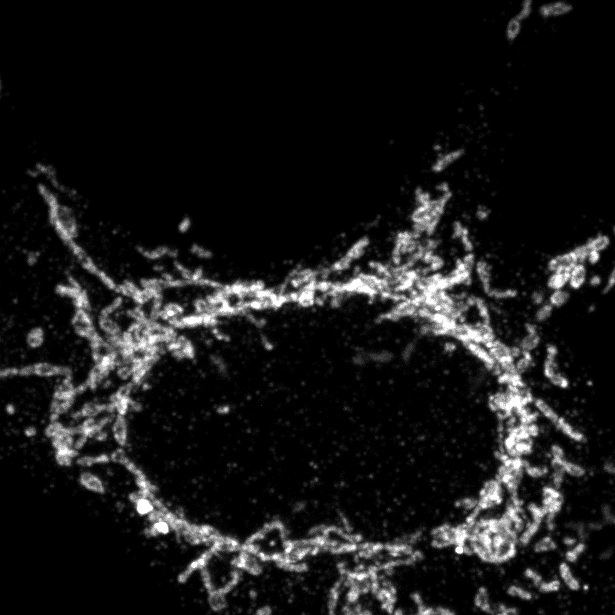

Supplement: Supplementary file 6 — Source Data EV Fig. 2 [file 44319_2023_9_MOESM6_ESM.zip › EV1/j/IMAGES/MTCH2 WT/MAX_Mtch2 ko mtch2 wt gfp pdh cy3 t40cy5 im26_thumb_w1Con-GFP-1D.tif (RGB).tif]

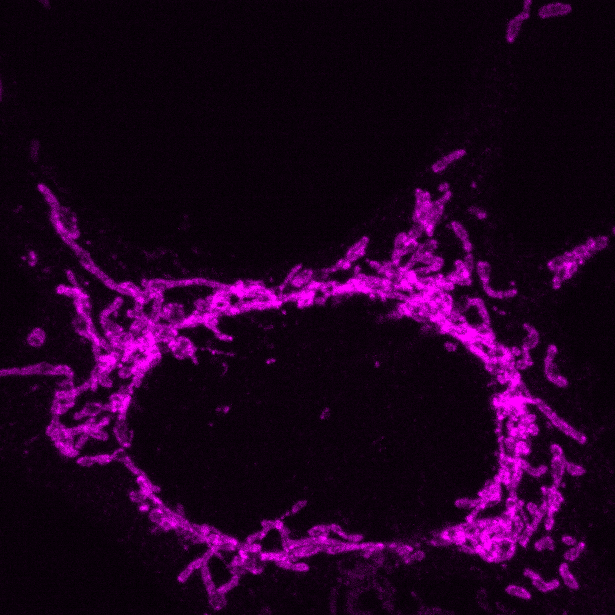

Supplement: Supplementary file 6 — Source Data EV Fig. 2 [file 44319_2023_9_MOESM6_ESM.zip › EV1/j/IMAGES/MTCH2 WT/MAX_Mtch2 ko mtch2 wt gfp pdh cy3 t40cy5 im26_thumb_w1Con-GFP-1D.tif (RGB)B.tif]

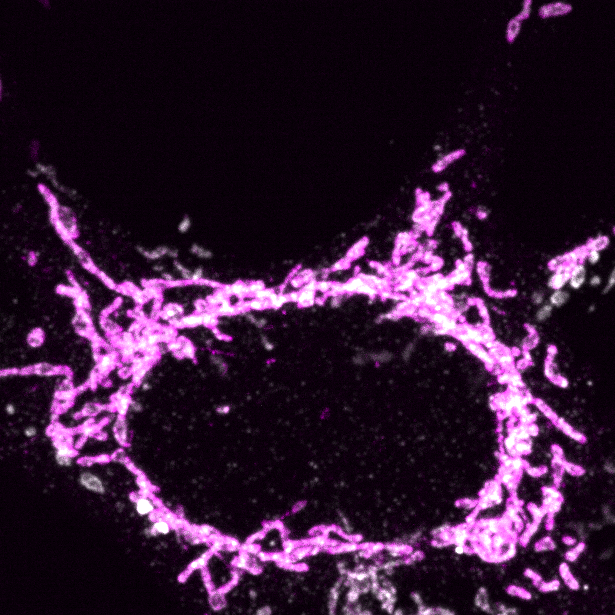

Supplement: Supplementary file 6 — Source Data EV Fig. 2 [file 44319_2023_9_MOESM6_ESM.zip › EV1/j/IMAGES/MTCH2 WT/MAX_Mtch2 ko mtch2 wt gfp pdh cy3 t40cy5 im26_thumb_w1Con-GFP-1D.tif (RGB)C.tif]

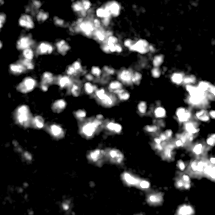

Supplement: Supplementary file 7 — Source Data EV Fig. 3 [file 44319_2023_9_MOESM7_ESM.zip › EV2/a/IMAGES/CONTROL/inset mfn2 ko control.tif]

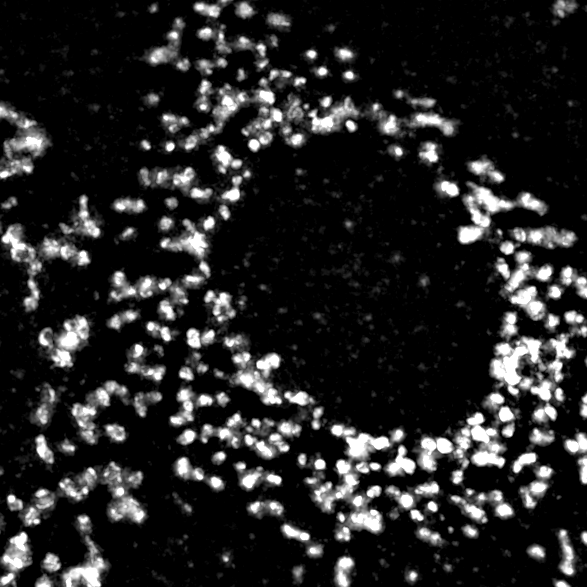

Supplement: Supplementary file 7 — Source Data EV Fig. 3 [file 44319_2023_9_MOESM7_ESM.zip › EV2/a/IMAGES/CONTROL/mfn2 ko control.tif]

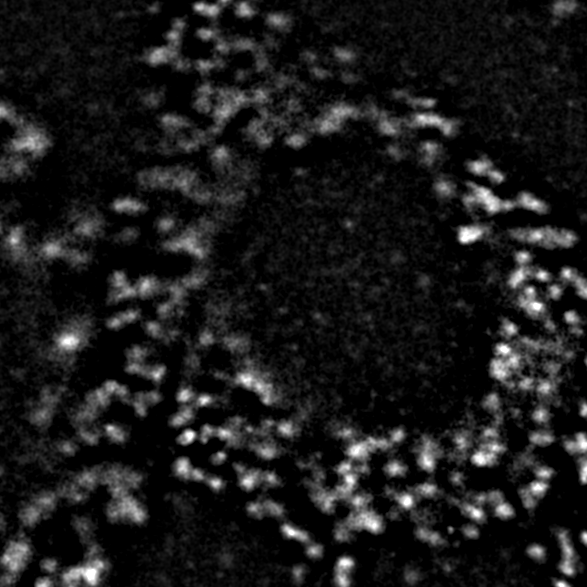

Supplement: Supplementary file 7 — Source Data EV Fig. 3 [file 44319_2023_9_MOESM7_ESM.zip › EV2/a/IMAGES/CONTROL/Process_16280.vsi - GFP-Quad, Cy5-Quad, mCherry-Quad-1.tif]

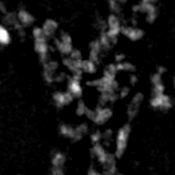

Supplement: Supplementary file 7 — Source Data EV Fig. 3 [file 44319_2023_9_MOESM7_ESM.zip › EV2/a/IMAGES/MFN2 acta/mfn2 ko Mfn2 ACTA colors-1.tif]

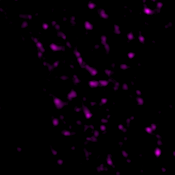

Supplement: Supplementary file 7 — Source Data EV Fig. 3 [file 44319_2023_9_MOESM7_ESM.zip › EV2/a/IMAGES/MFN2 acta/mfn2 ko Mfn2 ACTA colors-1.tif (RGB)acta.tif]

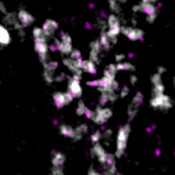

Supplement: Supplementary file 7 — Source Data EV Fig. 3 [file 44319_2023_9_MOESM7_ESM.zip › EV2/a/IMAGES/MFN2 acta/mfn2 ko Mfn2 ACTA colors-1.tif (RGB)merge.tif]

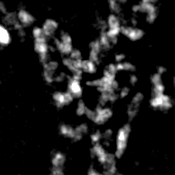

Supplement: Supplementary file 7 — Source Data EV Fig. 3 [file 44319_2023_9_MOESM7_ESM.zip › EV2/a/IMAGES/MFN2 acta/mfn2 ko Mfn2 ACTA colors-1.tif (RGB)mito.tif]

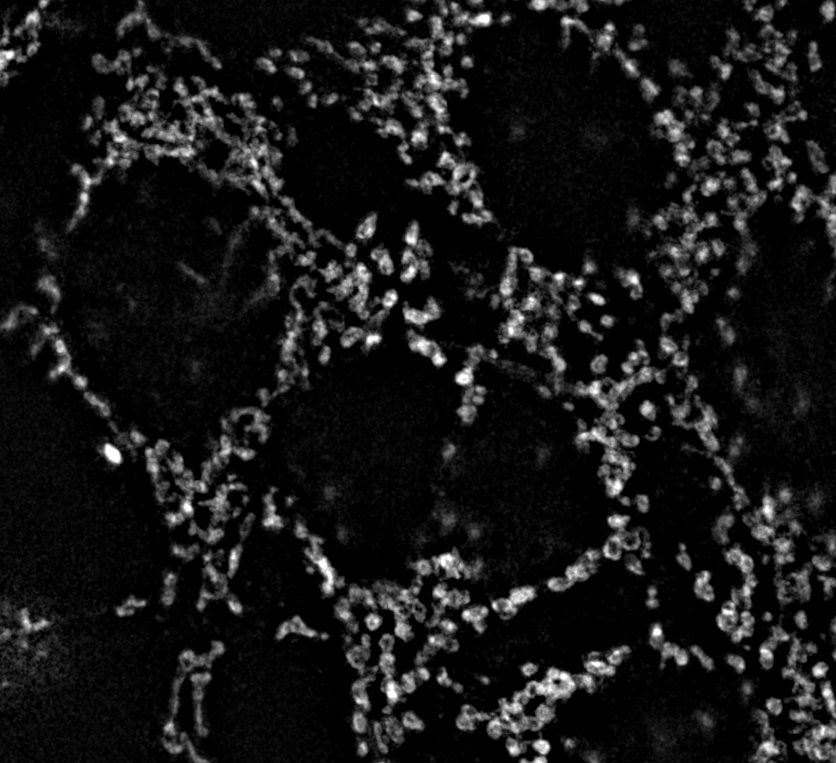

Supplement: Supplementary file 7 — Source Data EV Fig. 3 [file 44319_2023_9_MOESM7_ESM.zip › EV2/a/IMAGES/MFN2 acta/mfn2 ko Mfn2 ACTA colors.tif]

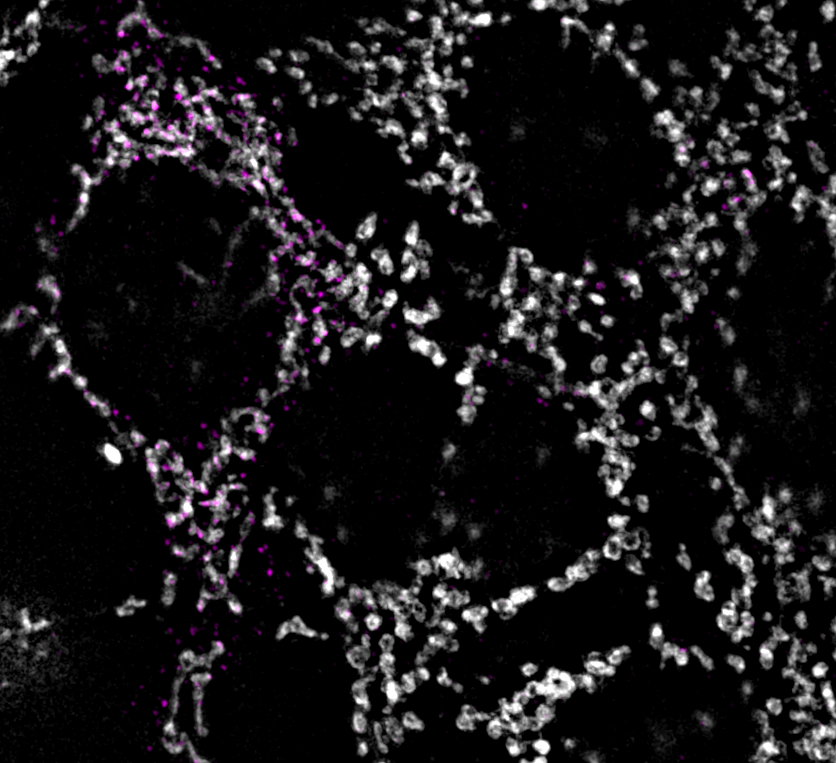

Supplement: Supplementary file 7 — Source Data EV Fig. 3 [file 44319_2023_9_MOESM7_ESM.zip › EV2/a/IMAGES/MFN2 acta/mfn2 ko Mfn2 ACTA colors.tif (RGB).tif]

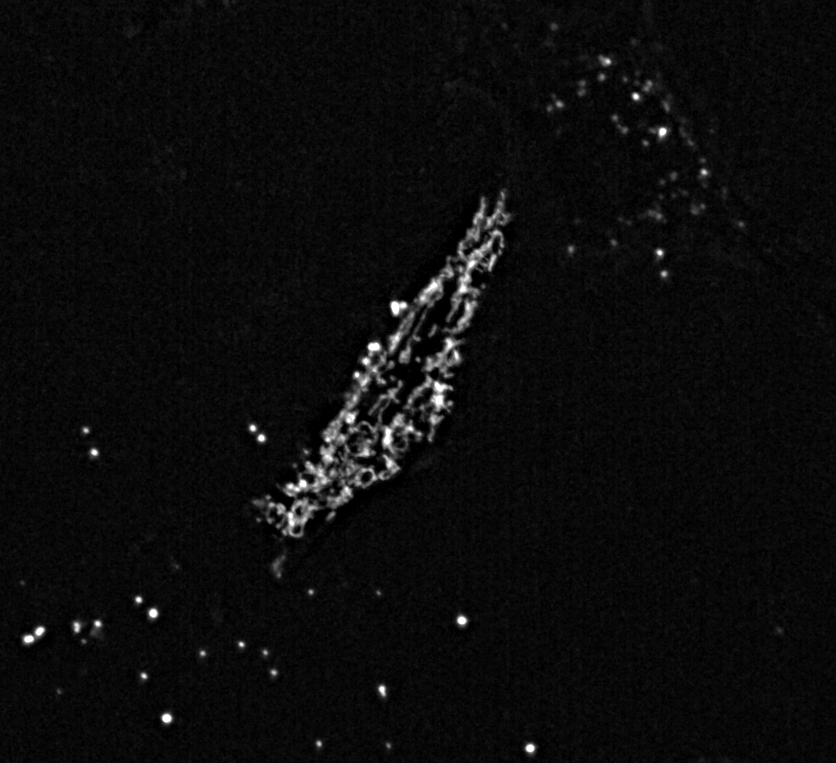

Supplement: Supplementary file 7 — Source Data EV Fig. 3 [file 44319_2023_9_MOESM7_ESM.zip › EV2/a/IMAGES/MFN2 IYFFT AND ACTA/MAX_Process_17229.vsi - Cy5-Quad, Current Settings, mCherry-Quad-1.tif]

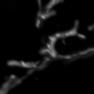

Supplement: Supplementary file 7 — Source Data EV Fig. 3 [file 44319_2023_9_MOESM7_ESM.zip › EV2/a/IMAGES/MFN2 IYFFT AND ACTA/mfn2 ko mfn2yfft and acta 2 colour-1-1.tif]

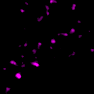

Supplement: Supplementary file 7 — Source Data EV Fig. 3 [file 44319_2023_9_MOESM7_ESM.zip › EV2/a/IMAGES/MFN2 IYFFT AND ACTA/mfn2 ko mfn2yfft and acta 2 colour-1-1.tif (RGB)acta.tif]

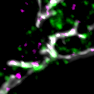

Supplement: Supplementary file 7 — Source Data EV Fig. 3 [file 44319_2023_9_MOESM7_ESM.zip › EV2/a/IMAGES/MFN2 IYFFT AND ACTA/mfn2 ko mfn2yfft and acta 2 colour-1-1.tif (RGB)comp.tif]

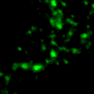

Supplement: Supplementary file 7 — Source Data EV Fig. 3 [file 44319_2023_9_MOESM7_ESM.zip › EV2/a/IMAGES/MFN2 IYFFT AND ACTA/mfn2 ko mfn2yfft and acta 2 colour-1-1.tif (RGB)iyfft.tif]

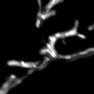

Supplement: Supplementary file 7 — Source Data EV Fig. 3 [file 44319_2023_9_MOESM7_ESM.zip › EV2/a/IMAGES/MFN2 IYFFT AND ACTA/mfn2 ko mfn2yfft and acta 2 colour-1-1.tif (RGB)tom.tif]

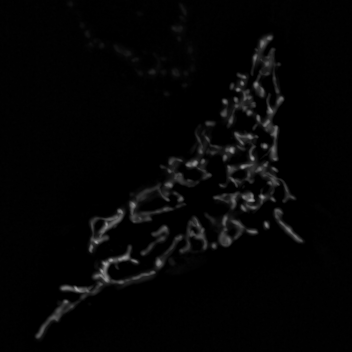

Supplement: Supplementary file 7 — Source Data EV Fig. 3 [file 44319_2023_9_MOESM7_ESM.zip › EV2/a/IMAGES/MFN2 IYFFT AND ACTA/mfn2 ko mfn2yfft and acta 2 colour-1.tif]

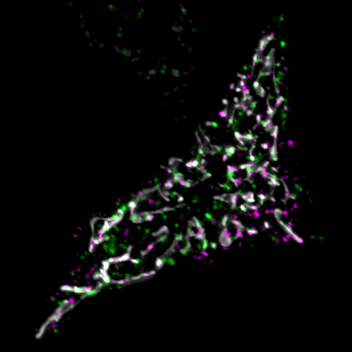

Supplement: Supplementary file 7 — Source Data EV Fig. 3 [file 44319_2023_9_MOESM7_ESM.zip › EV2/a/IMAGES/MFN2 IYFFT AND ACTA/mfn2 ko mfn2yfft and acta 2 colour-1.tif (RGB).tif]

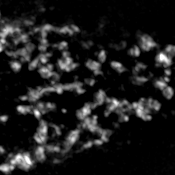

Supplement: Supplementary file 7 — Source Data EV Fig. 3 [file 44319_2023_9_MOESM7_ESM.zip › EV2/a/IMAGES/MFN2 IYFFT/MAX_Process_15731.vsi - GFP-Quad, mCherry-Quadcolr-2.tif]

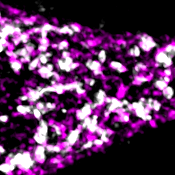

Supplement: Supplementary file 7 — Source Data EV Fig. 3 [file 44319_2023_9_MOESM7_ESM.zip › EV2/a/IMAGES/MFN2 IYFFT/MAX_Process_15731.vsi - GFP-Quad, mCherry-Quadcolr-2.tif (RGB) comp.tif]

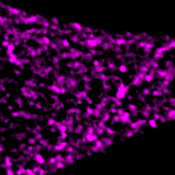

Supplement: Supplementary file 7 — Source Data EV Fig. 3 [file 44319_2023_9_MOESM7_ESM.zip › EV2/a/IMAGES/MFN2 IYFFT/MAX_Process_15731.vsi - GFP-Quad, mCherry-Quadcolr-2.tif (RGB) iyfft.tif]

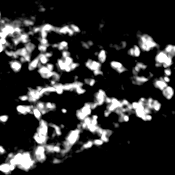

Supplement: Supplementary file 7 — Source Data EV Fig. 3 [file 44319_2023_9_MOESM7_ESM.zip › EV2/a/IMAGES/MFN2 IYFFT/MAX_Process_15731.vsi - GFP-Quad, mCherry-Quadcolr-2.tif (RGB) mito.tif]

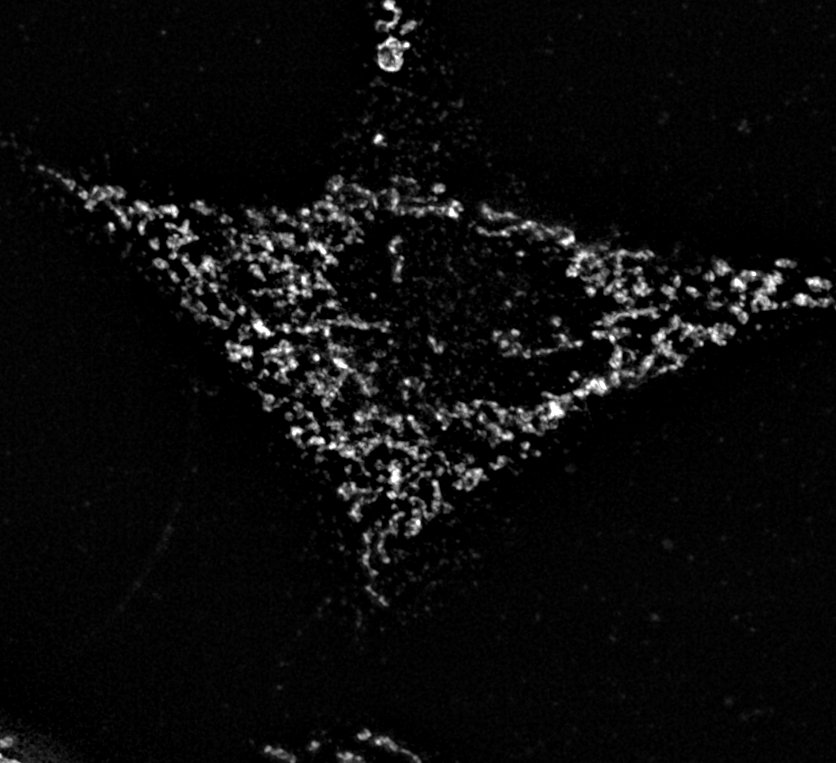

Supplement: Supplementary file 7 — Source Data EV Fig. 3 [file 44319_2023_9_MOESM7_ESM.zip › EV2/a/IMAGES/MFN2 IYFFT/MAX_Process_15731.vsi - GFP-Quad, mCherry-Quadcolr.tif]

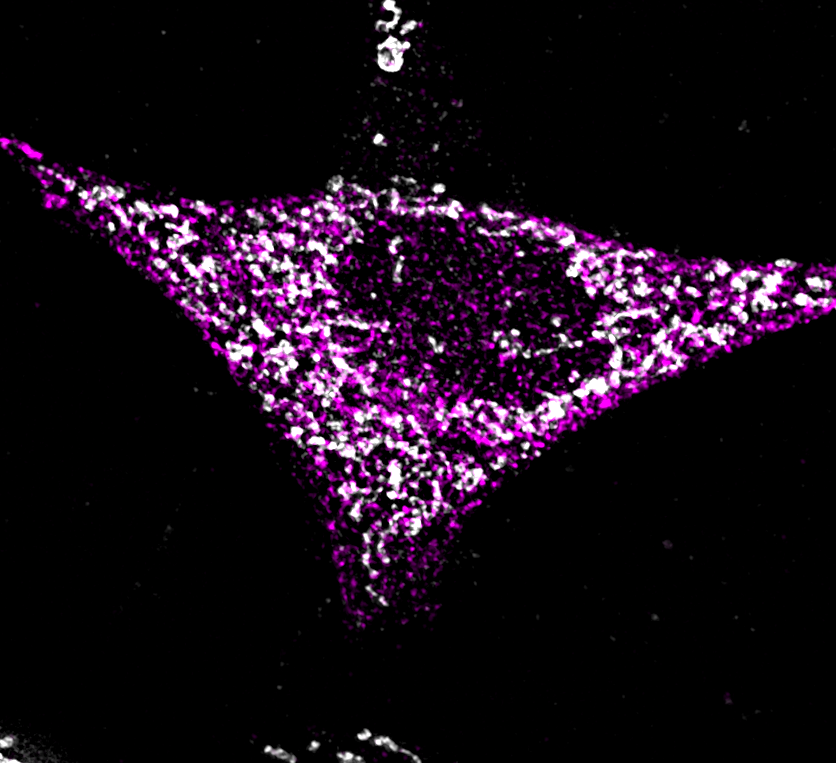

Supplement: Supplementary file 7 — Source Data EV Fig. 3 [file 44319_2023_9_MOESM7_ESM.zip › EV2/a/IMAGES/MFN2 IYFFT/MAX_Process_15731.vsi - GFP-Quad, mCherry-Quadcolr.tif (RGB).tif]

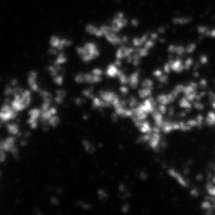

Supplement: Supplementary file 7 — Source Data EV Fig. 3 [file 44319_2023_9_MOESM7_ESM.zip › EV2/a/IMAGES/MFN2 K109A/MAX_MFN2 KO MEFS K109A 598 CYT633 IM4_thumb_w1Con-Cy5-1-1.tif]

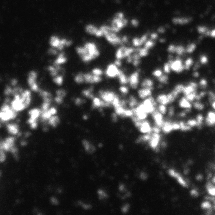

Supplement: Supplementary file 7 — Source Data EV Fig. 3 [file 44319_2023_9_MOESM7_ESM.zip › EV2/a/IMAGES/MFN2 K109A/MAX_MFN2 KO MEFS K109A 598 CYT633 IM4_thumb_w1Con-Cy5-1-1.tif (RGB) CH1.tif]

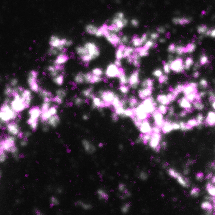

Supplement: Supplementary file 7 — Source Data EV Fig. 3 [file 44319_2023_9_MOESM7_ESM.zip › EV2/a/IMAGES/MFN2 K109A/MAX_MFN2 KO MEFS K109A 598 CYT633 IM4_thumb_w1Con-Cy5-1-1.tif (RGB) COMP.tif]

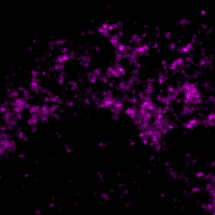

Supplement: Supplementary file 7 — Source Data EV Fig. 3 [file 44319_2023_9_MOESM7_ESM.zip › EV2/a/IMAGES/MFN2 K109A/MAX_MFN2 KO MEFS K109A 598 CYT633 IM4_thumb_w1Con-Cy5-1-1.tif (RGB)CH2.tif]

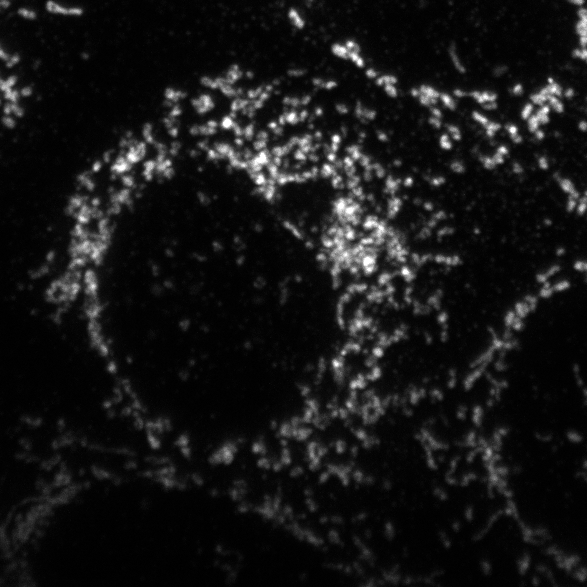

Supplement: Supplementary file 7 — Source Data EV Fig. 3 [file 44319_2023_9_MOESM7_ESM.zip › EV2/a/IMAGES/MFN2 K109A/MAX_MFN2 KO MEFS K109A 598 CYT633 IM4_thumb_w1Con-Cy5-1.tif]

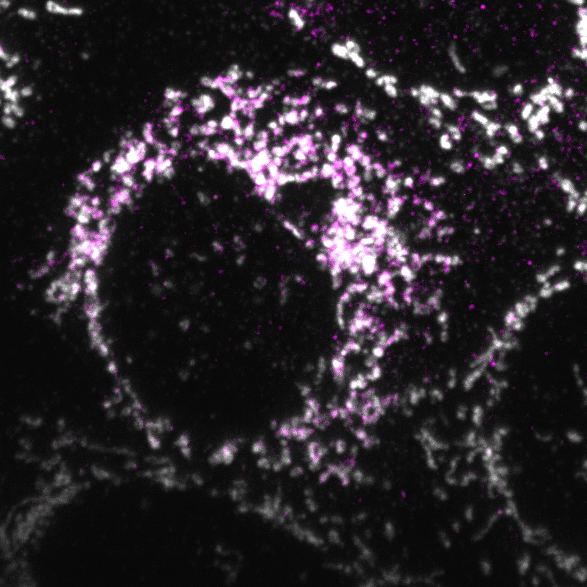

Supplement: Supplementary file 7 — Source Data EV Fig. 3 [file 44319_2023_9_MOESM7_ESM.zip › EV2/a/IMAGES/MFN2 K109A/MAX_MFN2 KO MEFS K109A 598 CYT633 IM4_thumb_w1Con-Cy5-1.tif (RGB).tif]

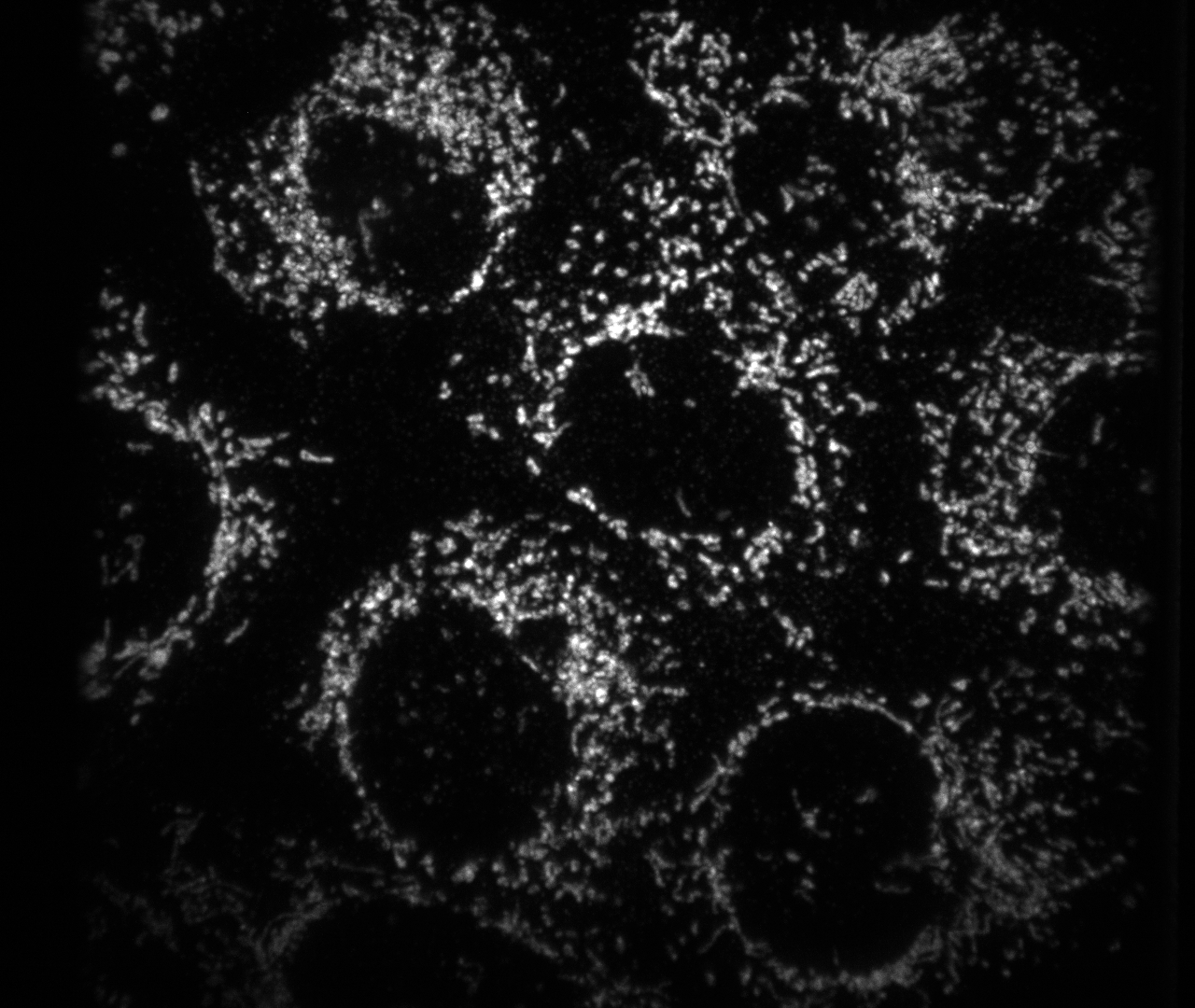

Supplement: Supplementary file 7 — Source Data EV Fig. 3 [file 44319_2023_9_MOESM7_ESM.zip › EV2/a/IMAGES/MFN2 K109A/MAX_MFN2 KO MEFS K109A 598 CYT633 IM4_thumb_w1Con-Cy5.tif]

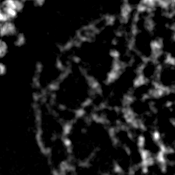

Supplement: Supplementary file 7 — Source Data EV Fig. 3 [file 44319_2023_9_MOESM7_ESM.zip › EV2/a/IMAGES/MFN2 WT/MAX_Process_15827.vsi - GFP-Quad, mCherry-Quad-1-1.tif]
