# Supplementary figures and images for: MTCH2 cooperates with MFN2 and lysophosphatidic acid synthesis to sustain mitochondrial fusion (part 5 of 6)
Source: EMBO Rep. 2023 Dec 14;25(1):8. doi: 10.1038/s44319-023-00009-1 (PMC10897490; doi:10.1038/s44319-023-00009-1)

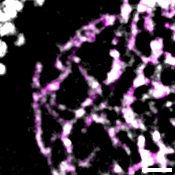

Supplement: Supplementary file 7 — Source Data EV Fig. 3 [file 44319_2023_9_MOESM7_ESM.zip › EV2/a/IMAGES/MFN2 WT/MAX_Process_15827.vsi - GFP-Quad, mCherry-Quad-1-1.tif (RGB) comp-1scale.tif]

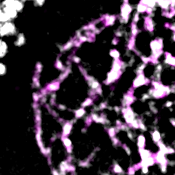

Supplement: Supplementary file 7 — Source Data EV Fig. 3 [file 44319_2023_9_MOESM7_ESM.zip › EV2/a/IMAGES/MFN2 WT/MAX_Process_15827.vsi - GFP-Quad, mCherry-Quad-1-1.tif (RGB) comp.tif]

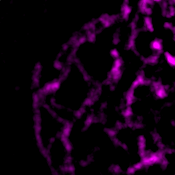

Supplement: Supplementary file 7 — Source Data EV Fig. 3 [file 44319_2023_9_MOESM7_ESM.zip › EV2/a/IMAGES/MFN2 WT/MAX_Process_15827.vsi - GFP-Quad, mCherry-Quad-1-1.tif (RGB)mfn2.tif]

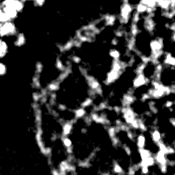

Supplement: Supplementary file 7 — Source Data EV Fig. 3 [file 44319_2023_9_MOESM7_ESM.zip › EV2/a/IMAGES/MFN2 WT/MAX_Process_15827.vsi - GFP-Quad, mCherry-Quad-1-1.tif (RGB)mito.tif]

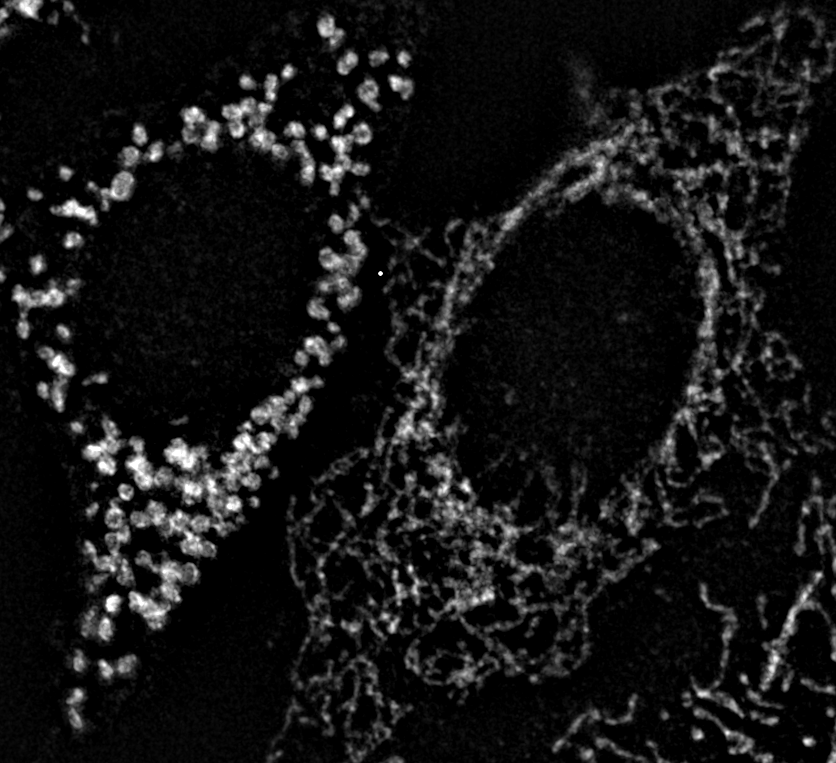

Supplement: Supplementary file 7 — Source Data EV Fig. 3 [file 44319_2023_9_MOESM7_ESM.zip › EV2/a/IMAGES/MFN2 WT/MAX_Process_15827.vsi - GFP-Quad, mCherry-Quad-1.tif]

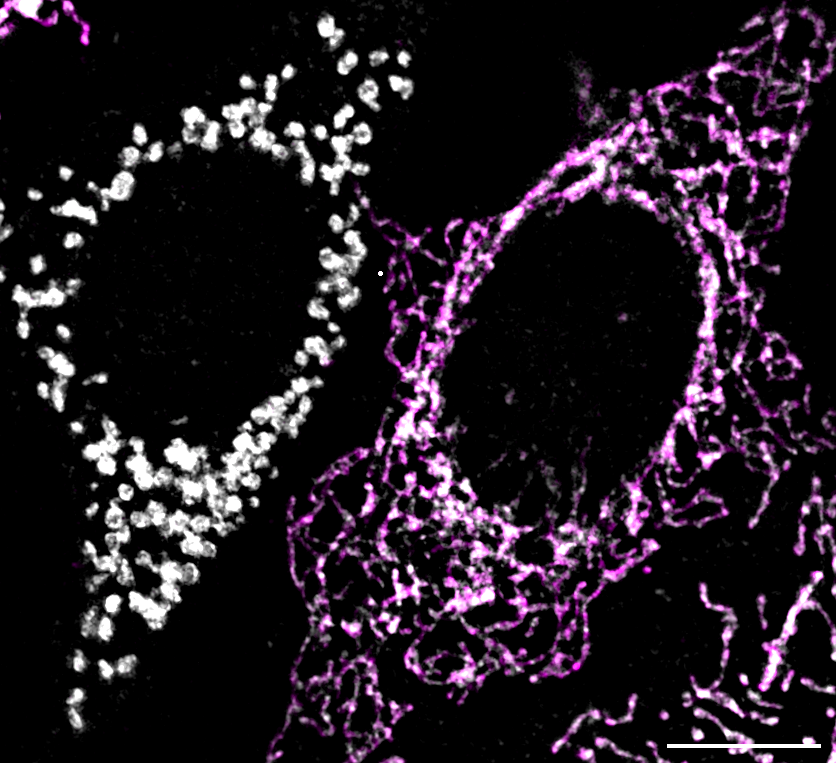

Supplement: Supplementary file 7 — Source Data EV Fig. 3 [file 44319_2023_9_MOESM7_ESM.zip › EV2/a/IMAGES/MFN2 WT/MAX_Process_15827.vsi - GFP-Quad, mCherry-Quad-1.tif (RGB)-1scale.tif]

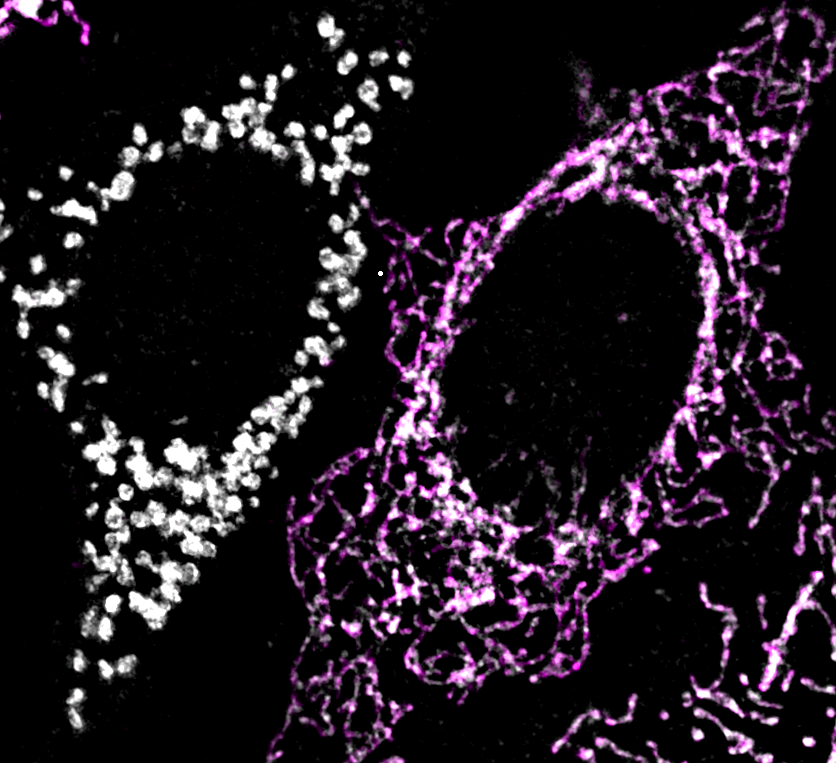

Supplement: Supplementary file 7 — Source Data EV Fig. 3 [file 44319_2023_9_MOESM7_ESM.zip › EV2/a/IMAGES/MFN2 WT/MAX_Process_15827.vsi - GFP-Quad, mCherry-Quad-1.tif (RGB).tif]

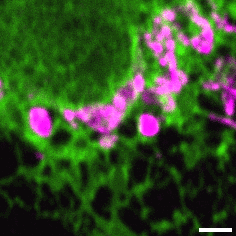

Supplement: Supplementary file 7 — Source Data EV Fig. 3 [file 44319_2023_9_MOESM7_ESM.zip › EV2/c/IMAGES/MTCH2 KO/2019-02-14_11.31.26_Mtch2KO ER Cy2 Tom Cy3_Andres_BFP_GFP_Cy3_...Cy2 Tom Cy3_Andres_BFP_GFP_Cy3_photoact.ims Resolution Level 1-1ss-1 2um scale bar.gif]

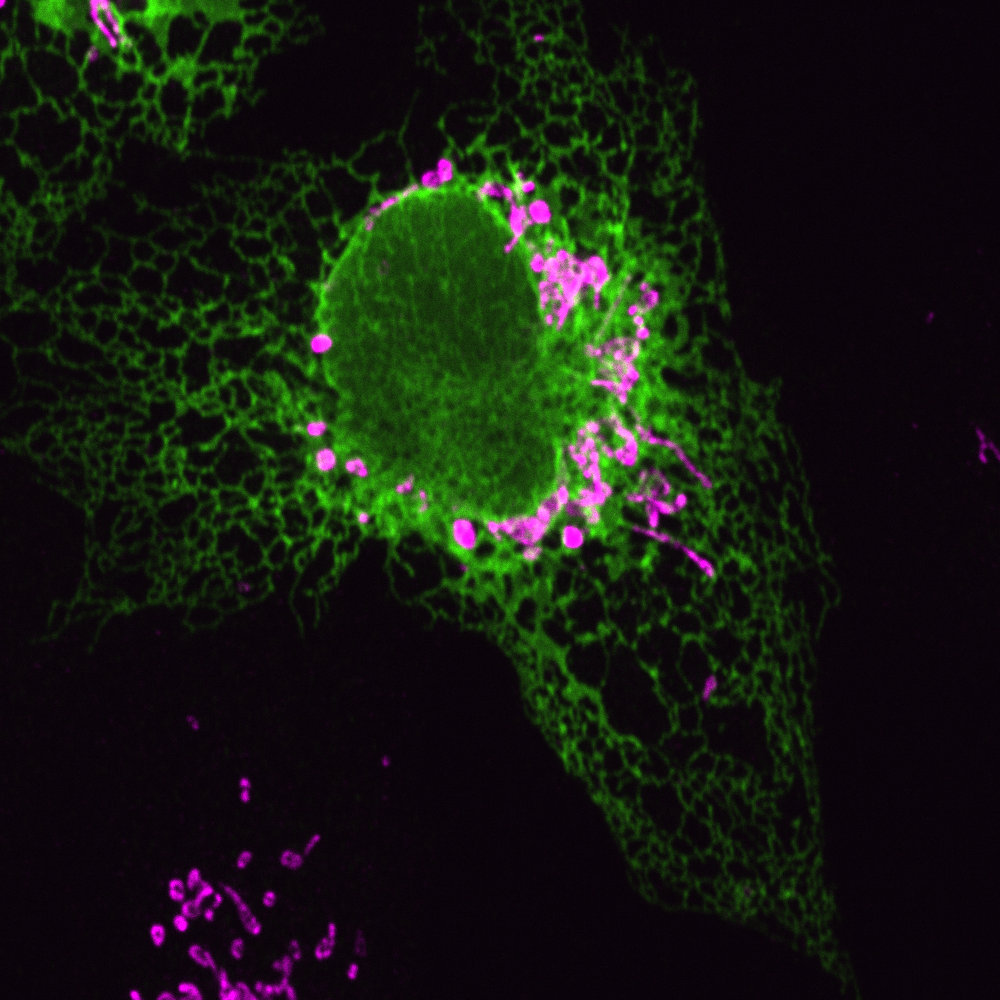

Supplement: Supplementary file 7 — Source Data EV Fig. 3 [file 44319_2023_9_MOESM7_ESM.zip › EV2/c/IMAGES/MTCH2 KO/2019-02-14_11.31.26_Mtch2KO ER Cy2 Tom Cy3_Andres_BFP_GFP_Cy3_...Cy2 Tom Cy3_Andres_BFP_GFP_Cy3_photoact.ims Resolution Level 1-1ss-1.tif]

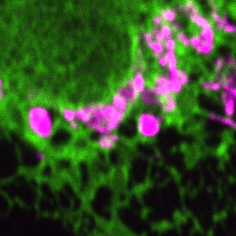

Supplement: Supplementary file 7 — Source Data EV Fig. 3 [file 44319_2023_9_MOESM7_ESM.zip › EV2/c/IMAGES/MTCH2 KO/2019-02-14_11.31.26_Mtch2KO ER Cy2 Tom Cy3_Andres_BFP_GFP_Cy3_...Cy2 Tom Cy3_Andres_BFP_GFP_Cy3_photoact.ims Resolution Level 1-1ss-2.tif]

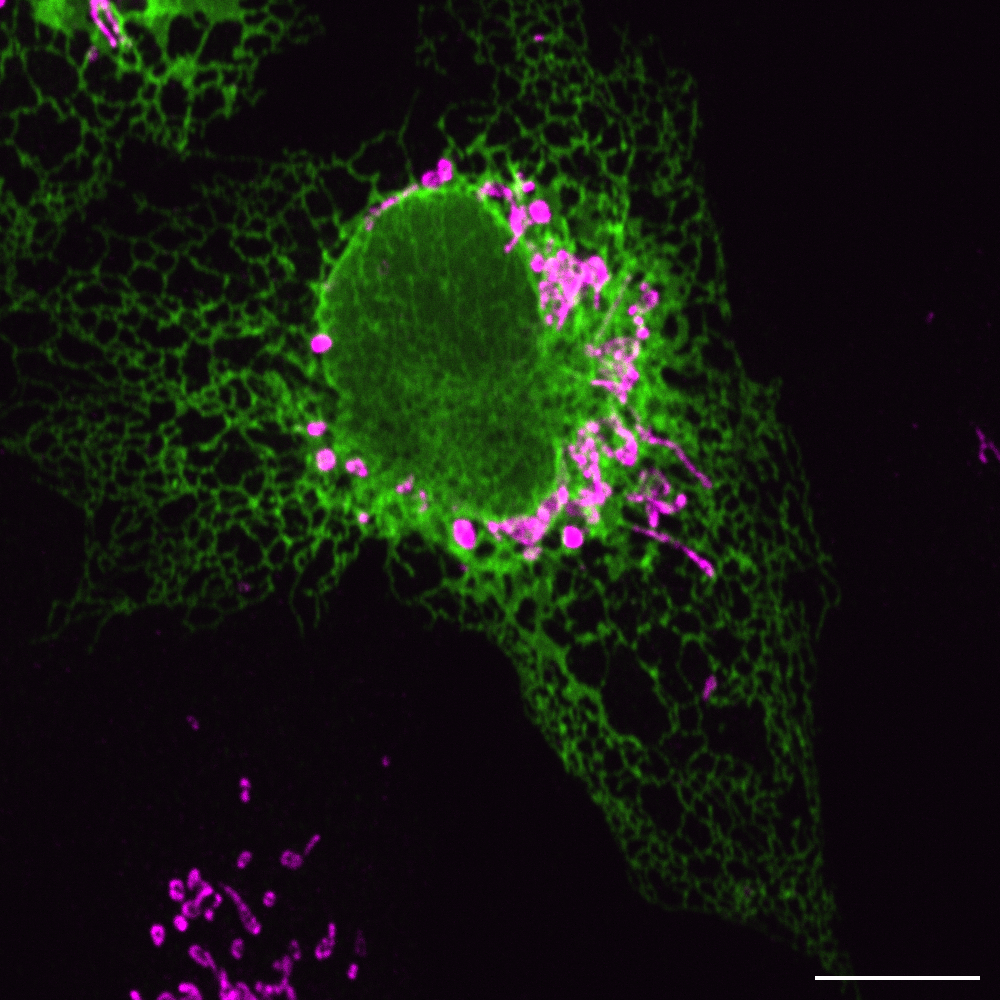

Supplement: Supplementary file 7 — Source Data EV Fig. 3 [file 44319_2023_9_MOESM7_ESM.zip › EV2/c/IMAGES/MTCH2 KO/2019-02-14_11.31.26_Mtch2KO ER Cy2 Tom Cy3_Andres_BFP_GFP_Cy3_...Cy2 Tom Cy3_Andres_BFP_GFP_Cy3_photoact.ims Resolution Level 1-1ss-3 10um scale bar.tif]

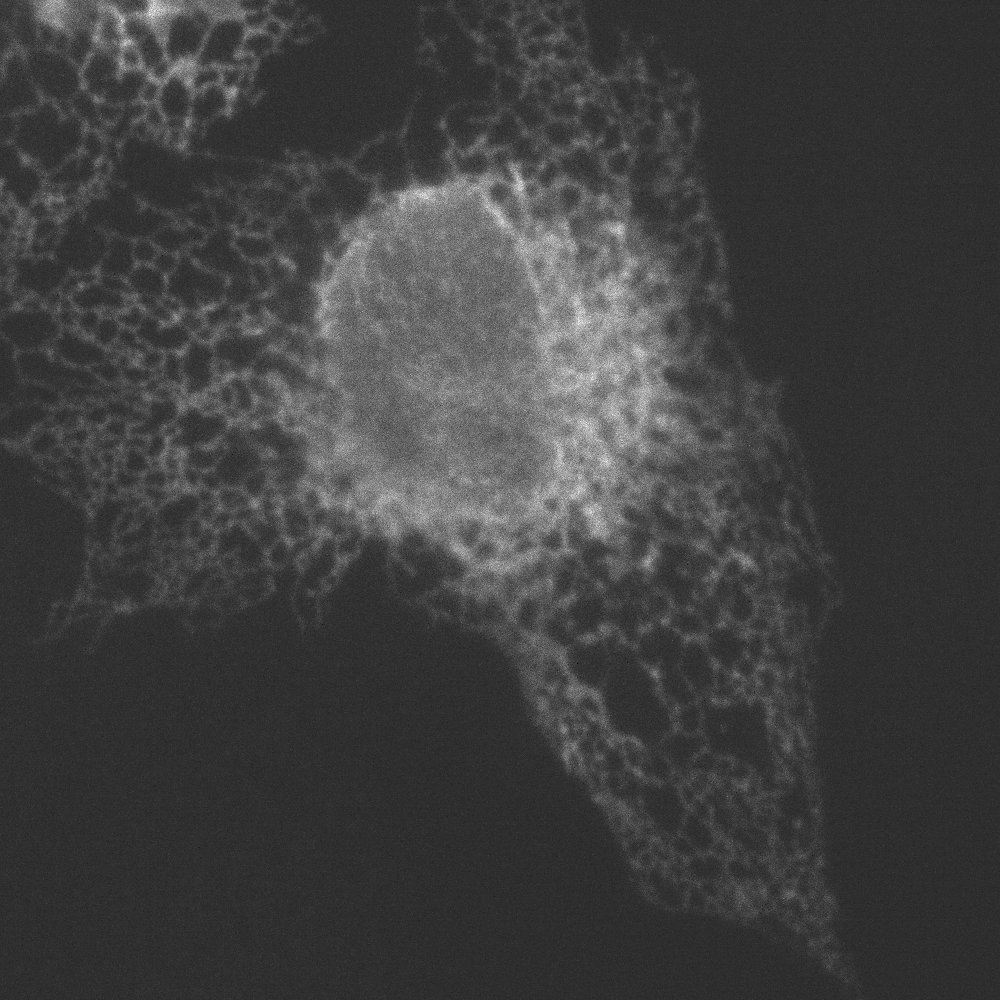

Supplement: Supplementary file 7 — Source Data EV Fig. 3 [file 44319_2023_9_MOESM7_ESM.zip › EV2/c/IMAGES/MTCH2 KO/2019-02-14_11.31.26_Mtch2KO ER Cy2 Tom Cy3_Andres_BFP_GFP_Cy3_...Cy2 Tom Cy3_Andres_BFP_GFP_Cy3_photoact.ims Resolution Level 1-1ss.tif]

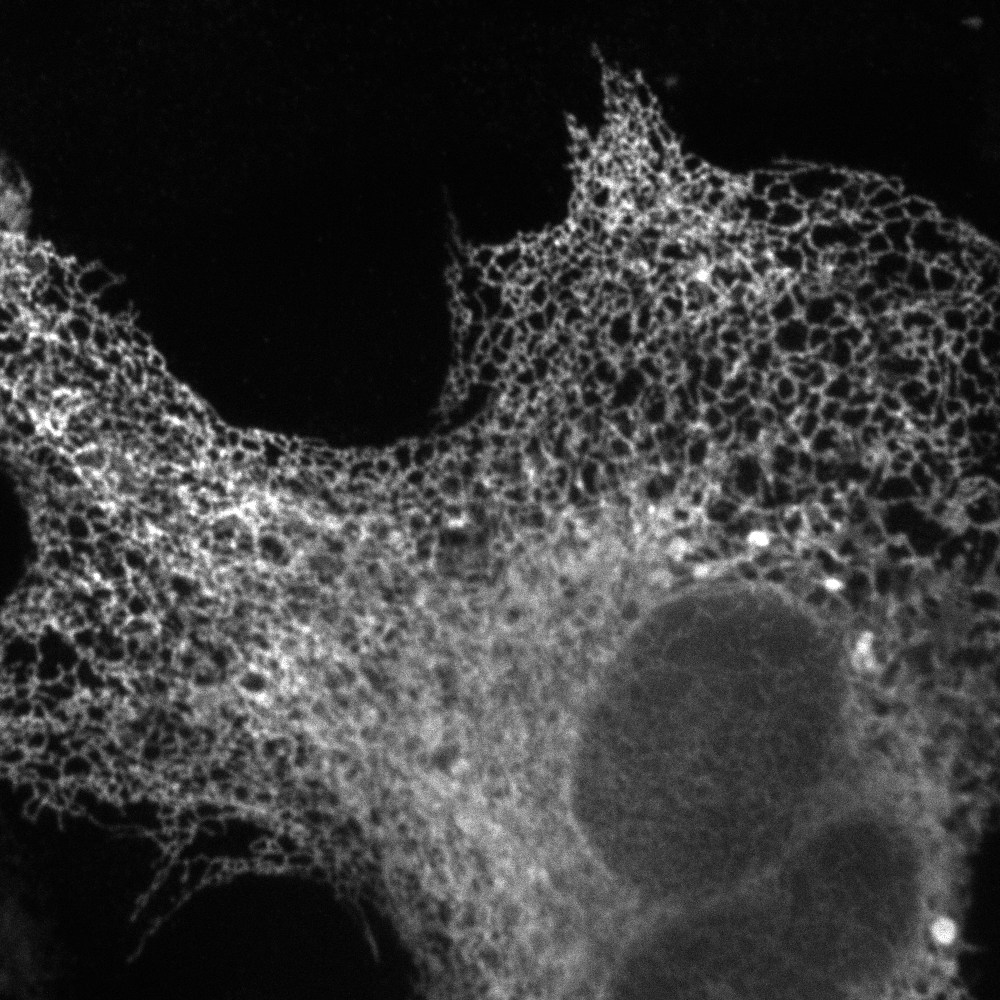

Supplement: Supplementary file 7 — Source Data EV Fig. 3 [file 44319_2023_9_MOESM7_ESM.zip › EV2/c/IMAGES/wt/2019-02-14_11.14.00_FF ER Cy2 Tom Cy3_Andres_BFP_GFP_Cy3_photo...Cy2 Tom Cy3_Andres_BFP_GFP_Cy3_photoact.ims Resolution Level 1-1.tif]

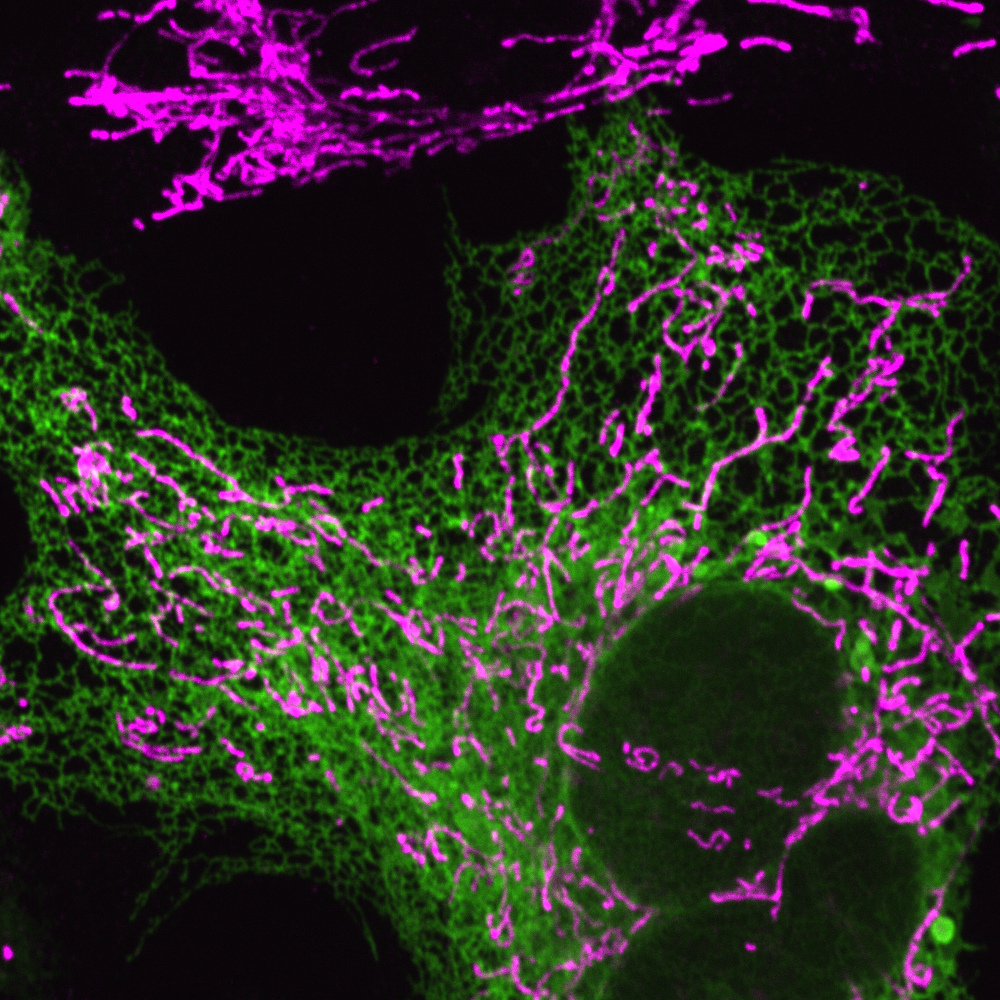

Supplement: Supplementary file 7 — Source Data EV Fig. 3 [file 44319_2023_9_MOESM7_ESM.zip › EV2/c/IMAGES/wt/2019-02-14_11.14.00_FF ER Cy2 Tom Cy3_Andres_BFP_GFP_Cy3_photo...Cy2 Tom Cy3_Andres_BFP_GFP_Cy3_photoact.ims Resolution Level 1-1.tifsss.tif]

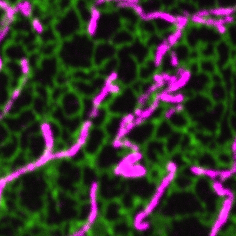

Supplement: Supplementary file 7 — Source Data EV Fig. 3 [file 44319_2023_9_MOESM7_ESM.zip › EV2/c/IMAGES/wt/2019-02-14_11.14.00_FF ER Cy2 Tom Cy3_Andres_BFP_GFP_Cy3_photo...Cy2 Tom Cy3_Andres_BFP_GFP_Cy3_photoact.ims Resolution Level 1-1ss.tif]

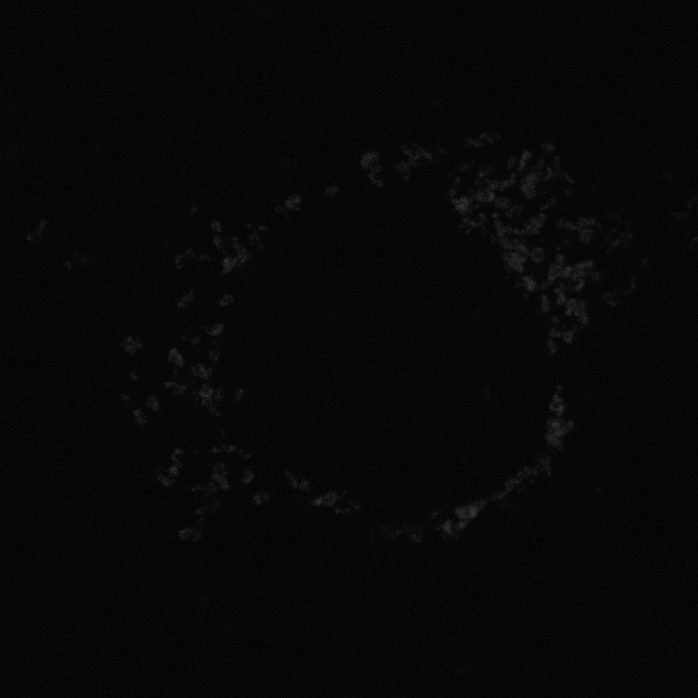

Supplement: Supplementary file 7 — Source Data EV Fig. 3 [file 44319_2023_9_MOESM7_ESM.zip › EV2/d/IMAGES/MTCH2 KO/mefs mtch2 ko mito-Er linker 3_thumb_w1Con-mcherry-1.TIF-1.tif]

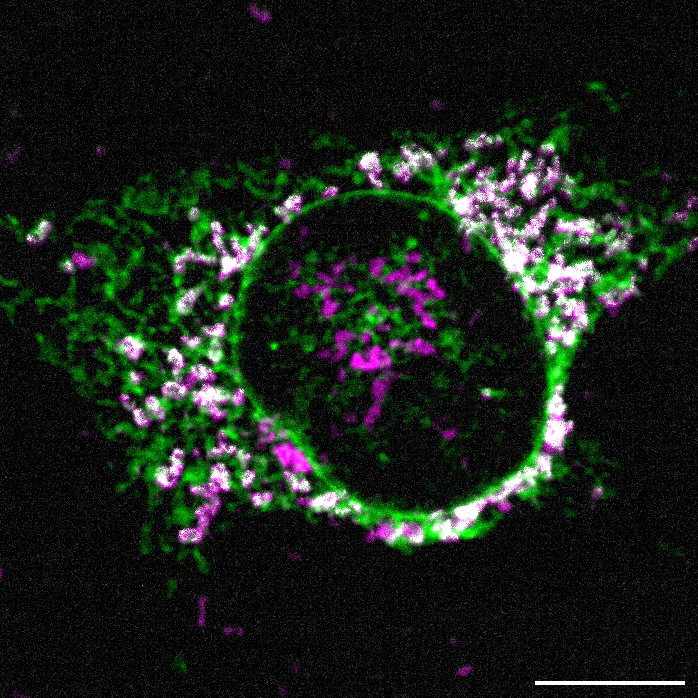

Supplement: Supplementary file 7 — Source Data EV Fig. 3 [file 44319_2023_9_MOESM7_ESM.zip › EV2/d/IMAGES/MTCH2 KO/mefs mtch2 ko mito-Er linker 3_thumb_w1Con-mcherry-1.TIF-1.tif (RGB)-1 10um scale bar.tif]

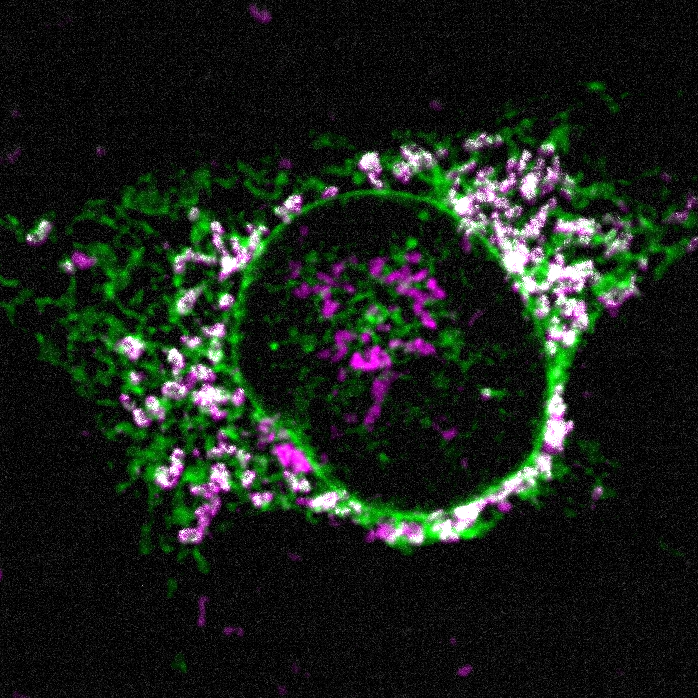

Supplement: Supplementary file 7 — Source Data EV Fig. 3 [file 44319_2023_9_MOESM7_ESM.zip › EV2/d/IMAGES/MTCH2 KO/mefs mtch2 ko mito-Er linker 3_thumb_w1Con-mcherry-1.TIF-1.tif (RGB).tif]

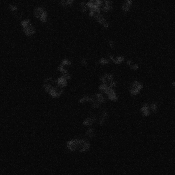

Supplement: Supplementary file 7 — Source Data EV Fig. 3 [file 44319_2023_9_MOESM7_ESM.zip › EV2/d/IMAGES/MTCH2 KO/mefs mtch2 ko mito-Er linker 3_thumb_w1Con-mcherry-1.TIF-2.tif]

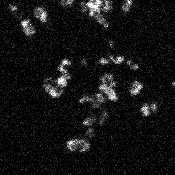

Supplement: Supplementary file 7 — Source Data EV Fig. 3 [file 44319_2023_9_MOESM7_ESM.zip › EV2/d/IMAGES/MTCH2 KO/mefs mtch2 ko mito-Er linker 3_thumb_w1Con-mcherry-1.TIF-2.tif (RGB) LINKER.tif]

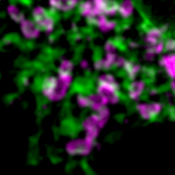

Supplement: Supplementary file 7 — Source Data EV Fig. 3 [file 44319_2023_9_MOESM7_ESM.zip › EV2/d/IMAGES/MTCH2 KO/mefs mtch2 ko mito-Er linker 3_thumb_w1Con-mcherry-1.TIF-2.tif (RGB) MITO ER.tif]

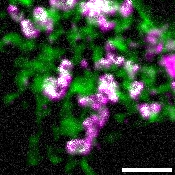

Supplement: Supplementary file 7 — Source Data EV Fig. 3 [file 44319_2023_9_MOESM7_ESM.zip › EV2/d/IMAGES/MTCH2 KO/mefs mtch2 ko mito-Er linker 3_thumb_w1Con-mcherry-1.TIF-2.tif (RGB) OVL-1 5um scale bar.tif]

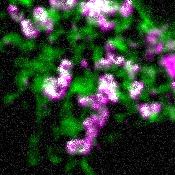

Supplement: Supplementary file 7 — Source Data EV Fig. 3 [file 44319_2023_9_MOESM7_ESM.zip › EV2/d/IMAGES/MTCH2 KO/mefs mtch2 ko mito-Er linker 3_thumb_w1Con-mcherry-1.TIF-2.tif (RGB) OVL.tif]

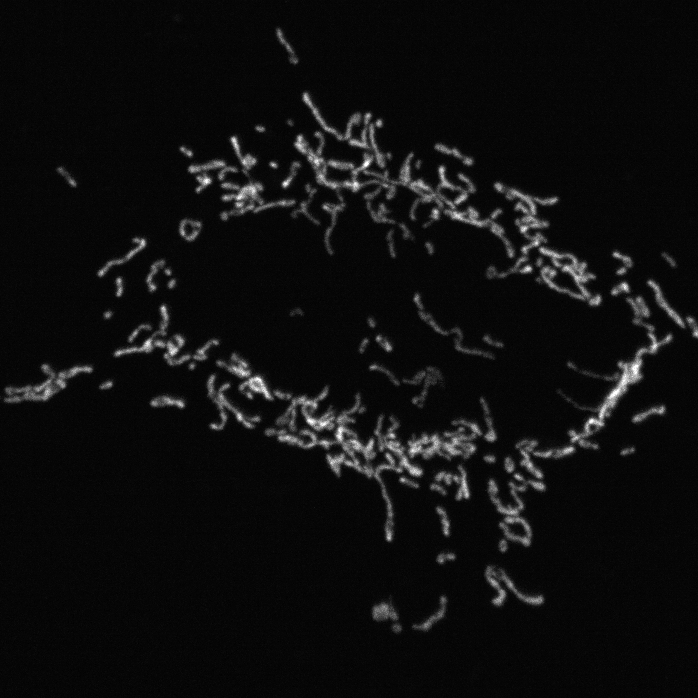

Supplement: Supplementary file 7 — Source Data EV Fig. 3 [file 44319_2023_9_MOESM7_ESM.zip › EV2/d/IMAGES/WT/mefs wt mito-Er linker 24_thumb_w1Con-mcherry-1.TIF-1.tif]

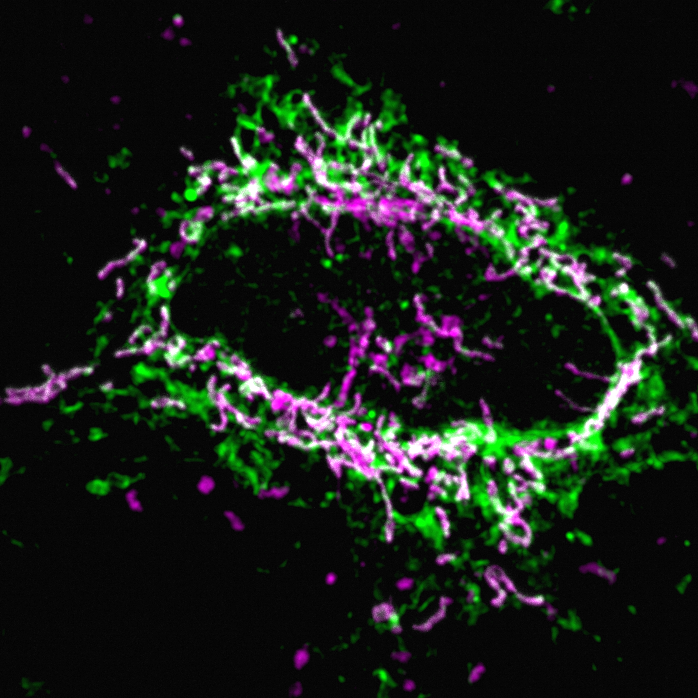

Supplement: Supplementary file 7 — Source Data EV Fig. 3 [file 44319_2023_9_MOESM7_ESM.zip › EV2/d/IMAGES/WT/mefs wt mito-Er linker 24_thumb_w1Con-mcherry-1.TIF-1.tif (RGB).tif]

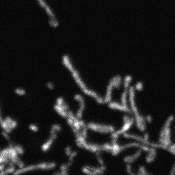

Supplement: Supplementary file 7 — Source Data EV Fig. 3 [file 44319_2023_9_MOESM7_ESM.zip › EV2/d/IMAGES/WT/mefs wt mito-Er linker 24_thumb_w1Con-mcherry-1.TIF-2.tif]

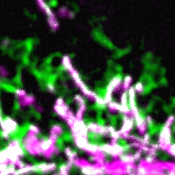

Supplement: Supplementary file 7 — Source Data EV Fig. 3 [file 44319_2023_9_MOESM7_ESM.zip › EV2/d/IMAGES/WT/mefs wt mito-Er linker 24_thumb_w1Con-mcherry-1.TIF-2.tif (RGB) COMPOSITE.tif]

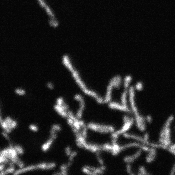

Supplement: Supplementary file 7 — Source Data EV Fig. 3 [file 44319_2023_9_MOESM7_ESM.zip › EV2/d/IMAGES/WT/mefs wt mito-Er linker 24_thumb_w1Con-mcherry-1.TIF-2.tif (RGB) LINKER.tif]

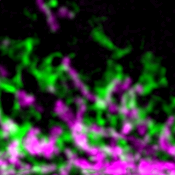

Supplement: Supplementary file 7 — Source Data EV Fig. 3 [file 44319_2023_9_MOESM7_ESM.zip › EV2/d/IMAGES/WT/mefs wt mito-Er linker 24_thumb_w1Con-mcherry-1.TIF-2.tif (RGB) MITO ER.tif]

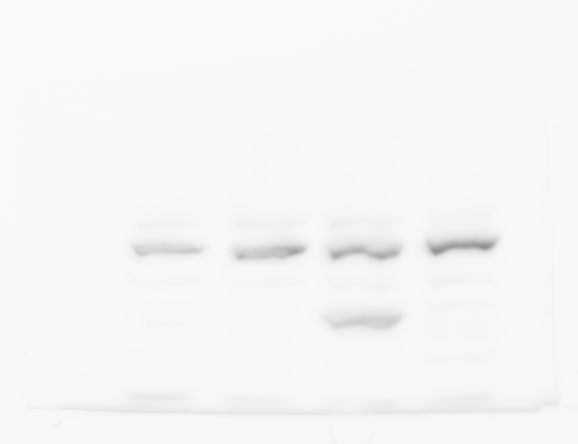

Supplement: Supplementary file 8 — Source Data EV Fig. 4 [file 44319_2023_9_MOESM8_ESM.zip › EV3/a/BLOTS/FLAG/FLAG BLOT.tif]

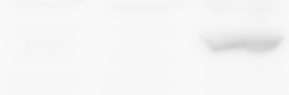

Supplement: Supplementary file 8 — Source Data EV Fig. 4 [file 44319_2023_9_MOESM8_ESM.zip › EV3/a/BLOTS/FLAG/flag cropped.tif]

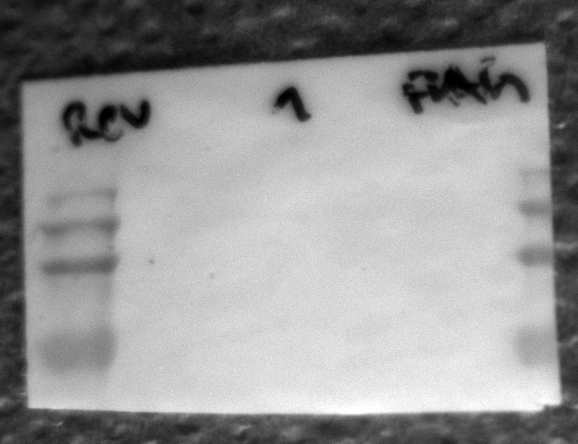

Supplement: Supplementary file 8 — Source Data EV Fig. 4 [file 44319_2023_9_MOESM8_ESM.zip › EV3/a/BLOTS/FLAG/FLAG MEMBRANE.tif]

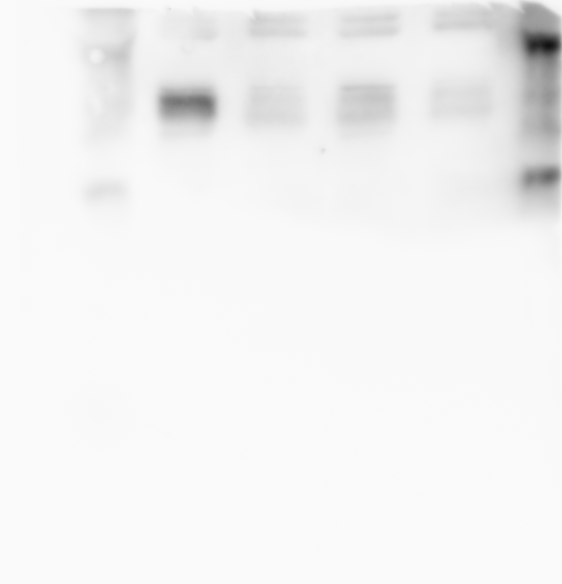

Supplement: Supplementary file 8 — Source Data EV Fig. 4 [file 44319_2023_9_MOESM8_ESM.zip › EV3/a/BLOTS/GPAT3/gpat3 blot.tif]

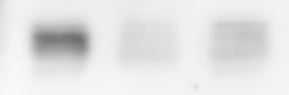

Supplement: Supplementary file 8 — Source Data EV Fig. 4 [file 44319_2023_9_MOESM8_ESM.zip › EV3/a/BLOTS/GPAT3/gpat3 cropped.tif]

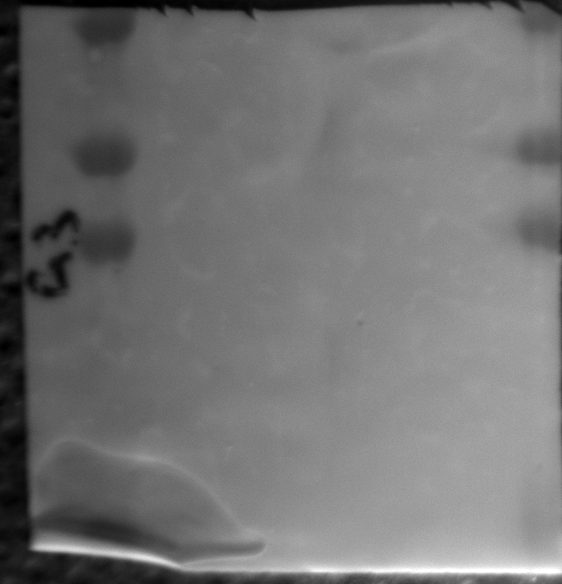

Supplement: Supplementary file 8 — Source Data EV Fig. 4 [file 44319_2023_9_MOESM8_ESM.zip › EV3/a/BLOTS/GPAT3/gpat3 membrane.tif]

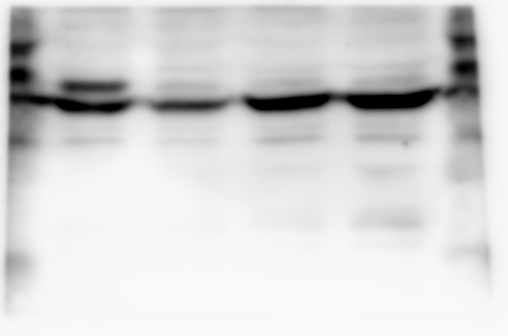

Supplement: Supplementary file 8 — Source Data EV Fig. 4 [file 44319_2023_9_MOESM8_ESM.zip › EV3/a/BLOTS/GPAT4/GPAT4 BLOT.tif]

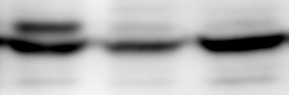

Supplement: Supplementary file 8 — Source Data EV Fig. 4 [file 44319_2023_9_MOESM8_ESM.zip › EV3/a/BLOTS/GPAT4/gpat4 cropped.tif]

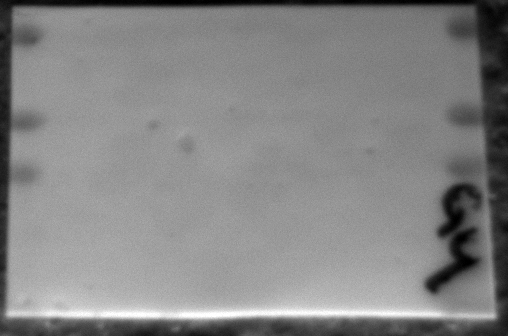

Supplement: Supplementary file 8 — Source Data EV Fig. 4 [file 44319_2023_9_MOESM8_ESM.zip › EV3/a/BLOTS/GPAT4/GPAT4 MEMBRANE.tif]

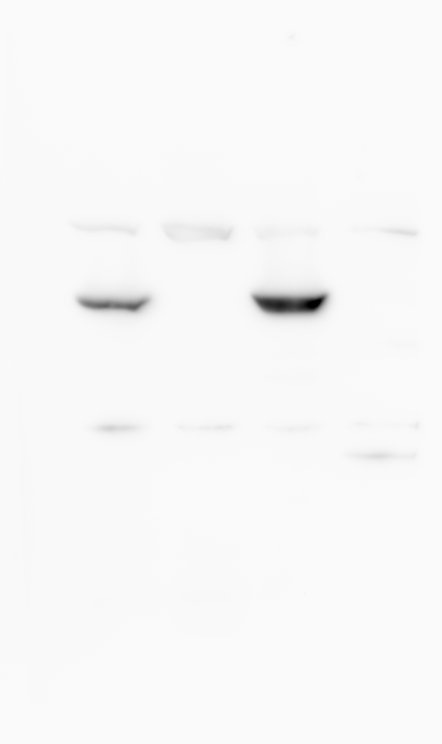

Supplement: Supplementary file 8 — Source Data EV Fig. 4 [file 44319_2023_9_MOESM8_ESM.zip › EV3/a/BLOTS/MFN2/MFN2 BLOT .tif]

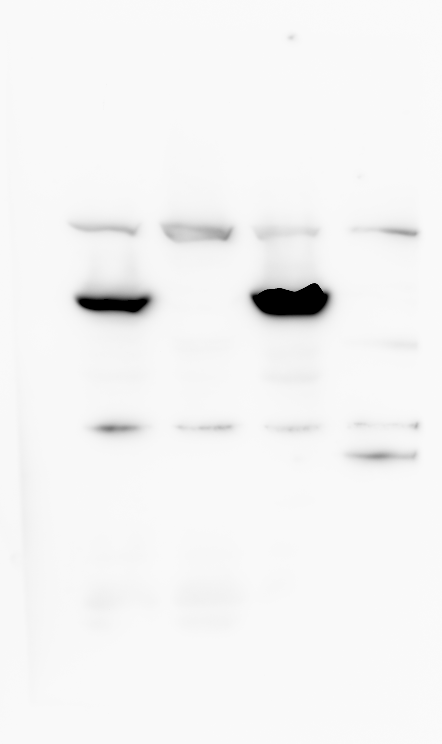

Supplement: Supplementary file 8 — Source Data EV Fig. 4 [file 44319_2023_9_MOESM8_ESM.zip › EV3/a/BLOTS/MFN2/MFN2 BLOT LONG.tif]

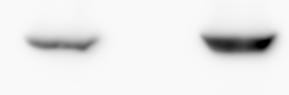

Supplement: Supplementary file 8 — Source Data EV Fig. 4 [file 44319_2023_9_MOESM8_ESM.zip › EV3/a/BLOTS/MFN2/mfn2 cropped.tif]

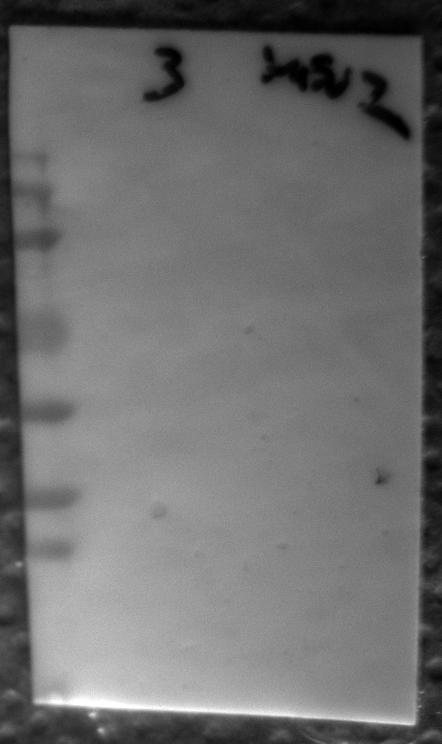

Supplement: Supplementary file 8 — Source Data EV Fig. 4 [file 44319_2023_9_MOESM8_ESM.zip › EV3/a/BLOTS/MFN2/MFN2 MEMBRANE.tif]

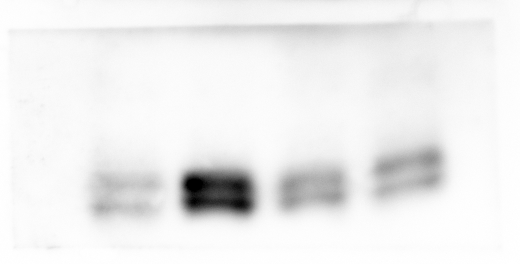

Supplement: Supplementary file 8 — Source Data EV Fig. 4 [file 44319_2023_9_MOESM8_ESM.zip › EV3/a/BLOTS/MTCH2/MTCH2 BLOT.tif]

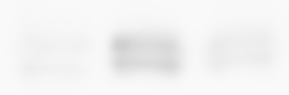

Supplement: Supplementary file 8 — Source Data EV Fig. 4 [file 44319_2023_9_MOESM8_ESM.zip › EV3/a/BLOTS/MTCH2/mtch2 cropped.tif]

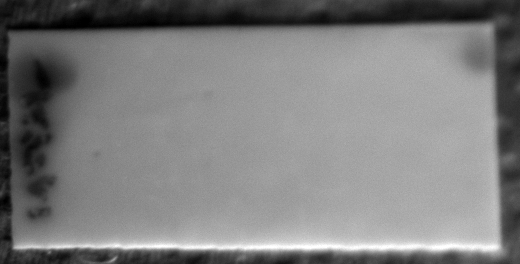

Supplement: Supplementary file 8 — Source Data EV Fig. 4 [file 44319_2023_9_MOESM8_ESM.zip › EV3/a/BLOTS/MTCH2/MTCH2 MEMBRANE.tif]

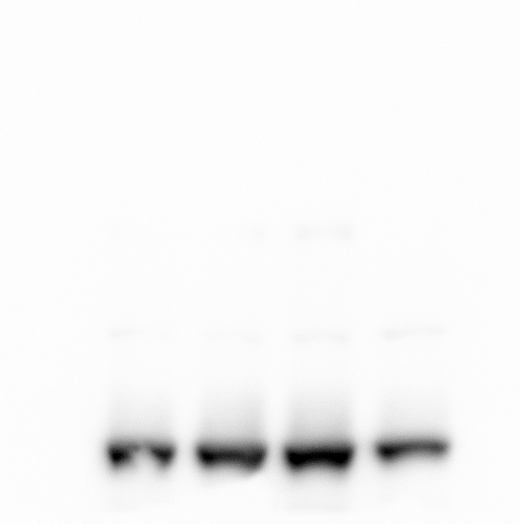

Supplement: Supplementary file 8 — Source Data EV Fig. 4 [file 44319_2023_9_MOESM8_ESM.zip › EV3/a/BLOTS/TOMM70/TOM70 BLOT.tif]

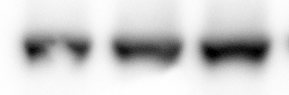

Supplement: Supplementary file 8 — Source Data EV Fig. 4 [file 44319_2023_9_MOESM8_ESM.zip › EV3/a/BLOTS/TOMM70/tom70 cropped.tif]

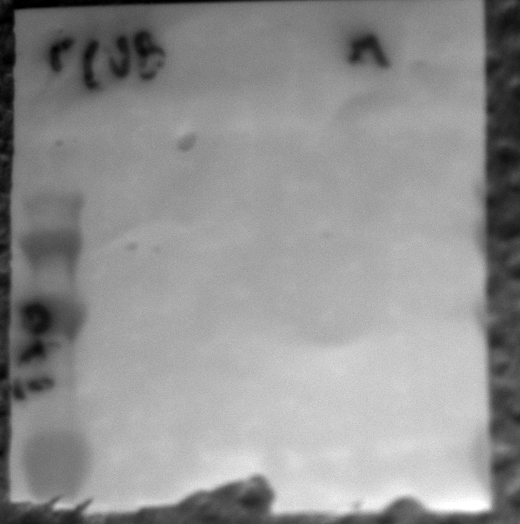

Supplement: Supplementary file 8 — Source Data EV Fig. 4 [file 44319_2023_9_MOESM8_ESM.zip › EV3/a/BLOTS/TOMM70/TOM70 MEMBRANE.tif]

# EV 3A GPAT3/4 AND MTCH2 EXPRESSION LEVELS IN MFN2 KO RESCUE WITH MFN2 FLAG

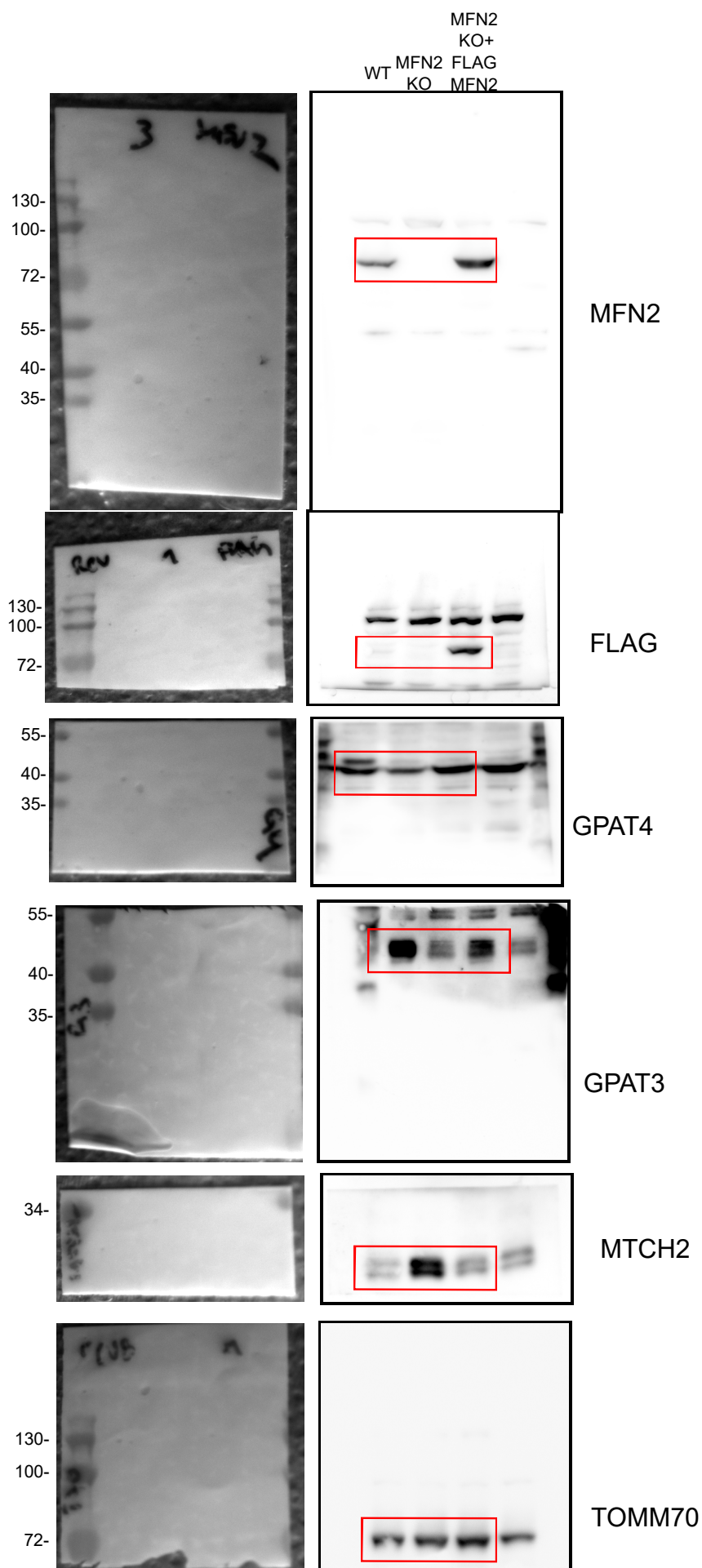

Supplement: Supplementary file 8 — Source Data EV Fig. 4 [file 44319_2023_9_MOESM8_ESM.zip › EV3/a/UNCROPED BLOTS EV3A.pdf]

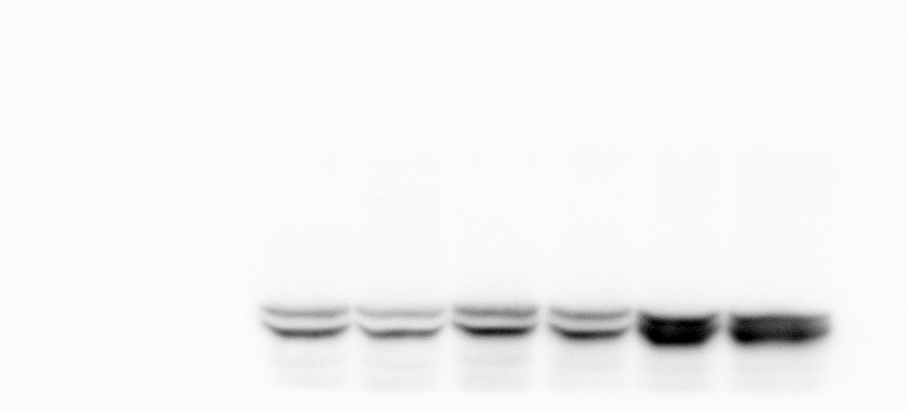

Supplement: Supplementary file 8 — Source Data EV Fig. 4 [file 44319_2023_9_MOESM8_ESM.zip › EV3/b/BLOTS/DRP1/drp1 blot.tif]

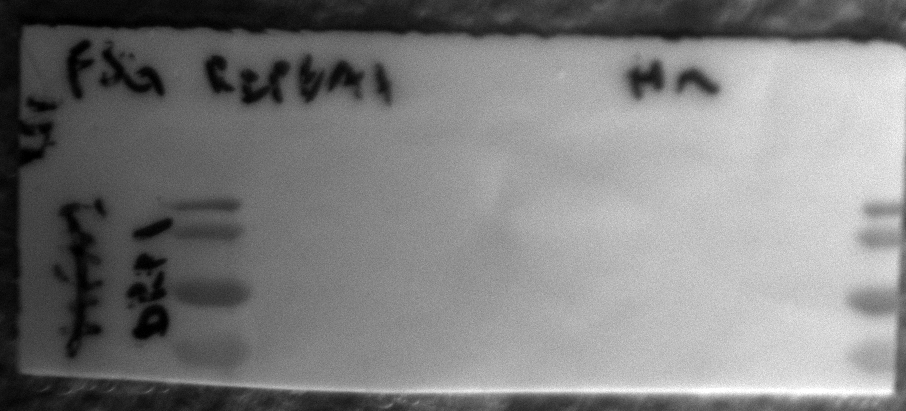

Supplement: Supplementary file 8 — Source Data EV Fig. 4 [file 44319_2023_9_MOESM8_ESM.zip › EV3/b/BLOTS/DRP1/drp1 membrane.tif]

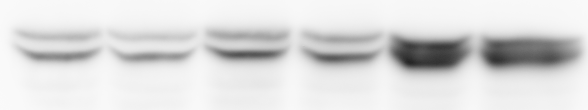

Supplement: Supplementary file 8 — Source Data EV Fig. 4 [file 44319_2023_9_MOESM8_ESM.zip › EV3/b/BLOTS/DRP1/DRP1.tif]

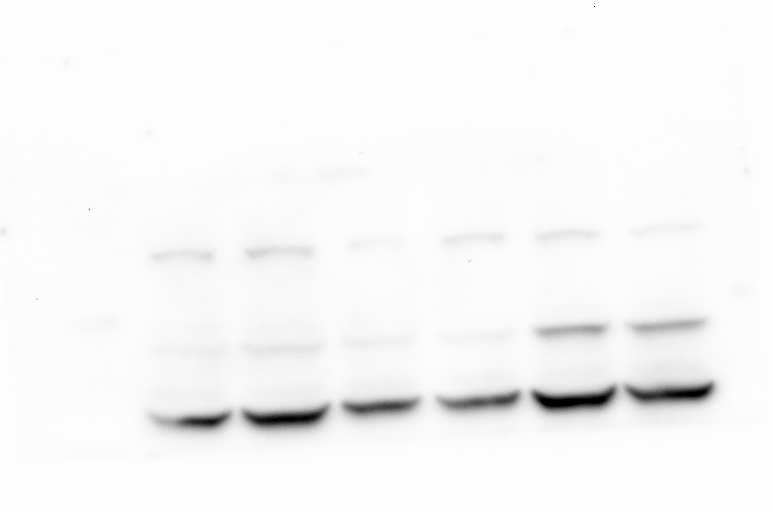

Supplement: Supplementary file 8 — Source Data EV Fig. 4 [file 44319_2023_9_MOESM8_ESM.zip › EV3/b/BLOTS/GPAT1/gpat1 mito blot.tif]

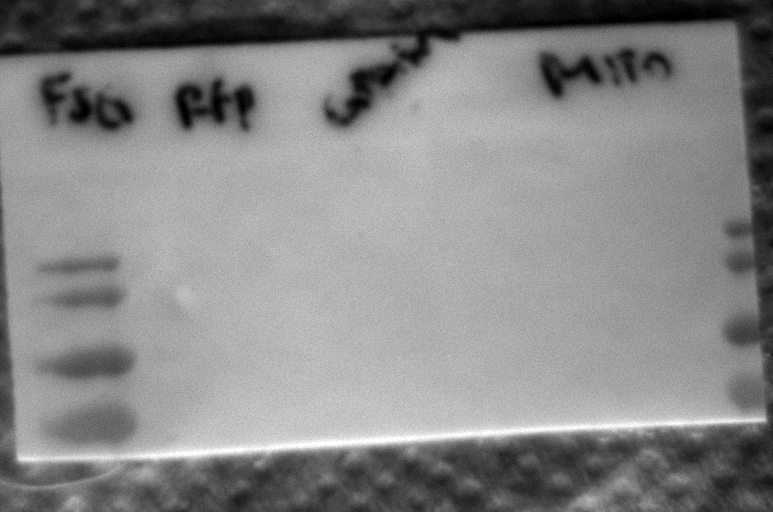

Supplement: Supplementary file 8 — Source Data EV Fig. 4 [file 44319_2023_9_MOESM8_ESM.zip › EV3/b/BLOTS/GPAT1/gpat1 mito membrane.tif]

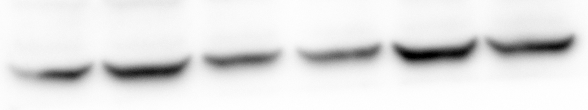

Supplement: Supplementary file 8 — Source Data EV Fig. 4 [file 44319_2023_9_MOESM8_ESM.zip › EV3/b/BLOTS/GPAT1/GPAT1.tif]

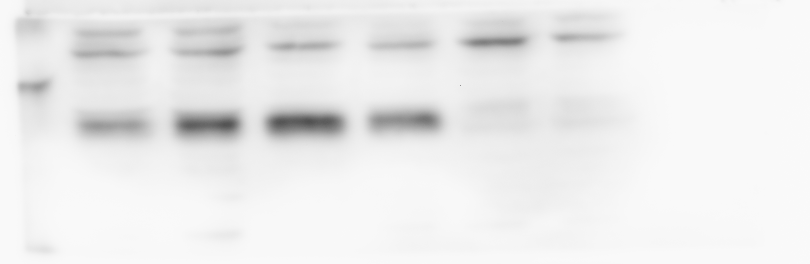

Supplement: Supplementary file 8 — Source Data EV Fig. 4 [file 44319_2023_9_MOESM8_ESM.zip › EV3/b/BLOTS/GPAT3/gpat3 er blot.tif]

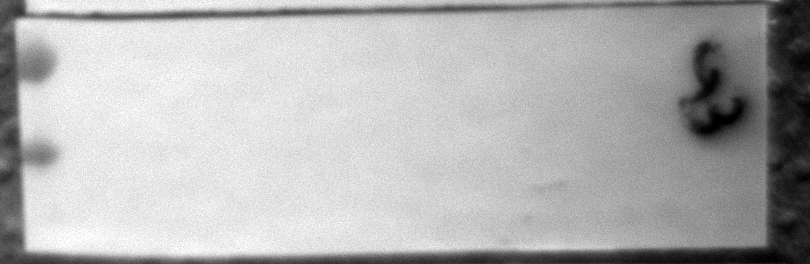

Supplement: Supplementary file 8 — Source Data EV Fig. 4 [file 44319_2023_9_MOESM8_ESM.zip › EV3/b/BLOTS/GPAT3/gpat3 er membrane.tif]

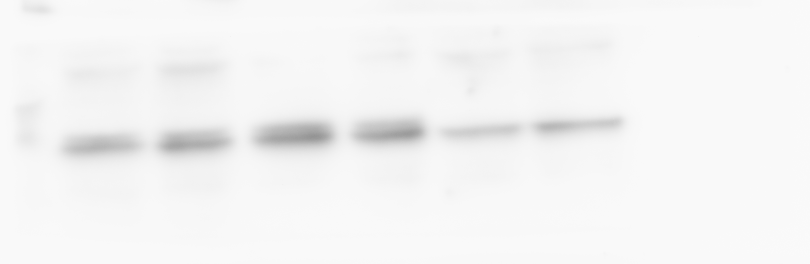

Supplement: Supplementary file 8 — Source Data EV Fig. 4 [file 44319_2023_9_MOESM8_ESM.zip › EV3/b/BLOTS/GPAT4/gpat4 er blot.tif]

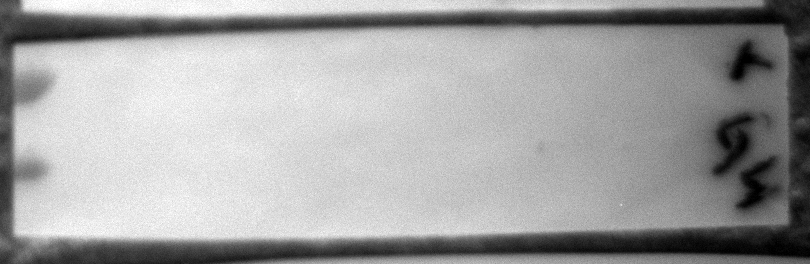

Supplement: Supplementary file 8 — Source Data EV Fig. 4 [file 44319_2023_9_MOESM8_ESM.zip › EV3/b/BLOTS/GPAT4/gpat4 er membrane.tif]

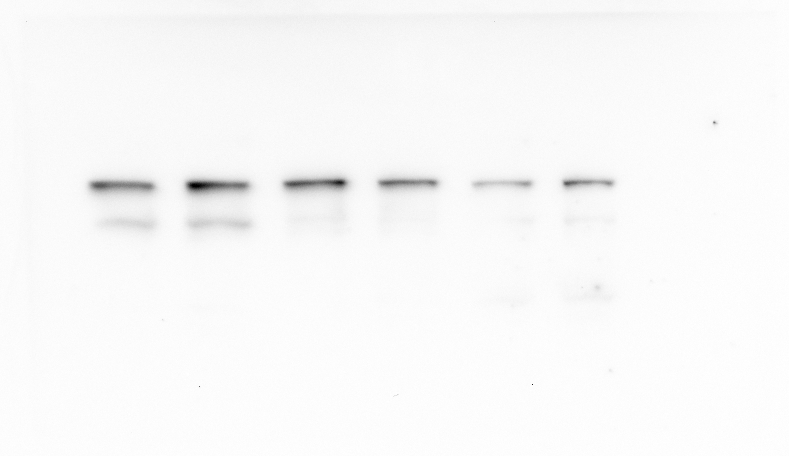

Supplement: Supplementary file 8 — Source Data EV Fig. 4 [file 44319_2023_9_MOESM8_ESM.zip › EV3/b/BLOTS/IP3R3/ip3r3 blot.tif]

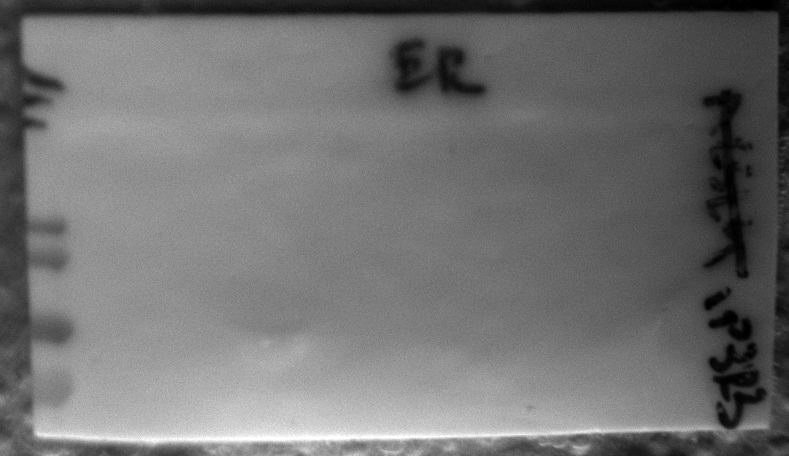

Supplement: Supplementary file 8 — Source Data EV Fig. 4 [file 44319_2023_9_MOESM8_ESM.zip › EV3/b/BLOTS/IP3R3/ip3r3 membrane.tif]

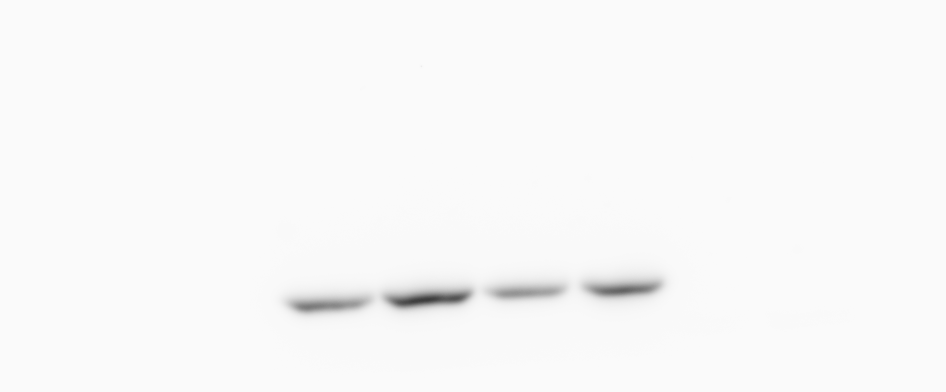

Supplement: Supplementary file 8 — Source Data EV Fig. 4 [file 44319_2023_9_MOESM8_ESM.zip › EV3/b/BLOTS/MFN2 HM/mfn2 mito blot.tif]

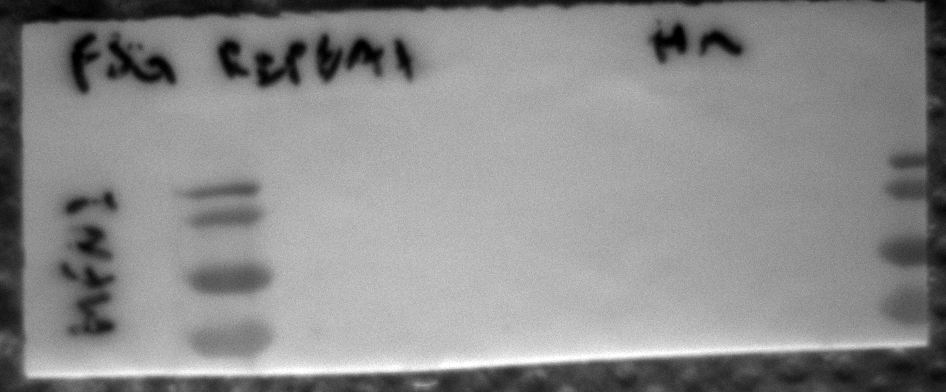

Supplement: Supplementary file 8 — Source Data EV Fig. 4 [file 44319_2023_9_MOESM8_ESM.zip › EV3/b/BLOTS/MFN2 HM/mfn2 mito membrane.tif]

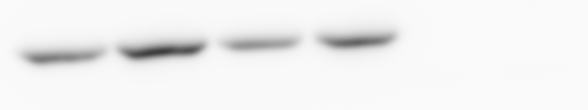

Supplement: Supplementary file 8 — Source Data EV Fig. 4 [file 44319_2023_9_MOESM8_ESM.zip › EV3/b/BLOTS/MFN2 HM/MFN2.tif]

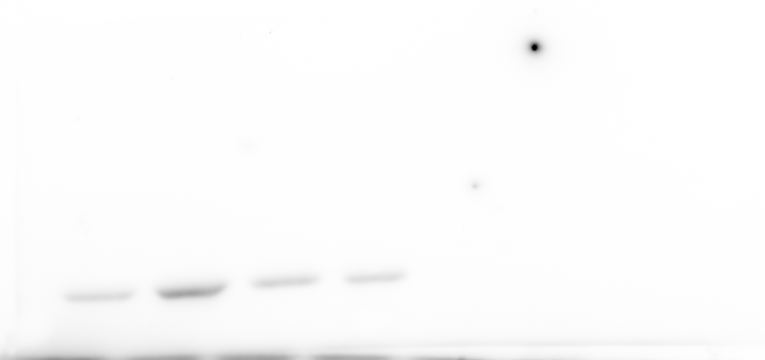

Supplement: Supplementary file 8 — Source Data EV Fig. 4 [file 44319_2023_9_MOESM8_ESM.zip › EV3/b/BLOTS/MFN2 LM/mfn2 er blot.tif]

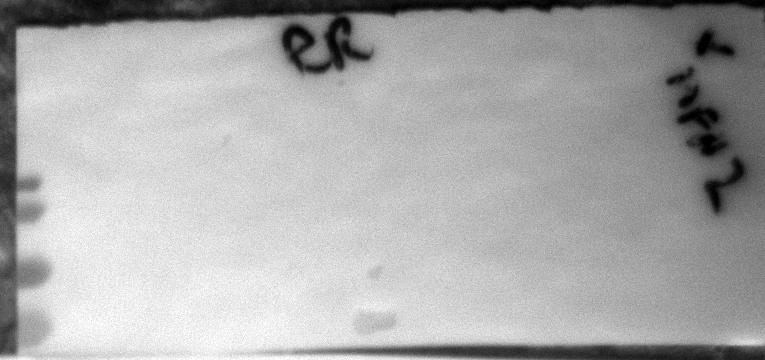

Supplement: Supplementary file 8 — Source Data EV Fig. 4 [file 44319_2023_9_MOESM8_ESM.zip › EV3/b/BLOTS/MFN2 LM/mfn2 er membrane.tif]

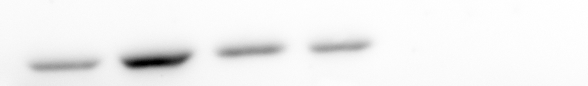

Supplement: Supplementary file 8 — Source Data EV Fig. 4 [file 44319_2023_9_MOESM8_ESM.zip › EV3/b/BLOTS/MFN2 LM/MFN2 ER.tif]

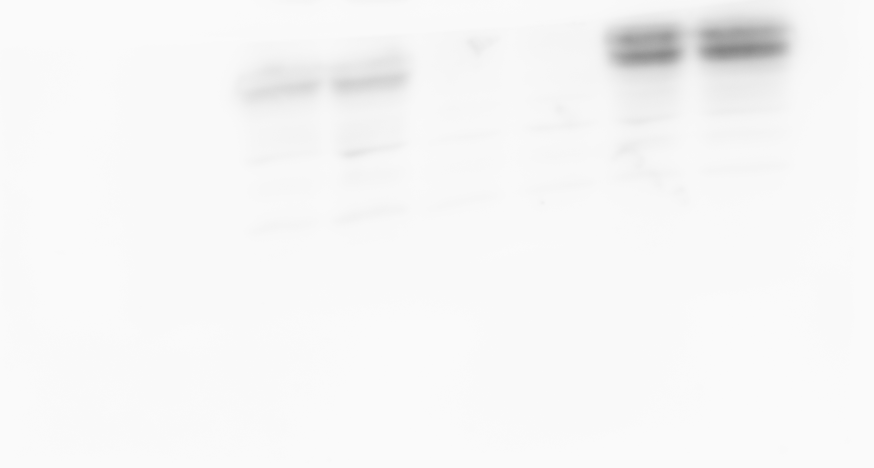

Supplement: Supplementary file 8 — Source Data EV Fig. 4 [file 44319_2023_9_MOESM8_ESM.zip › EV3/b/BLOTS/MTCH2/mtch2 blot.tif]

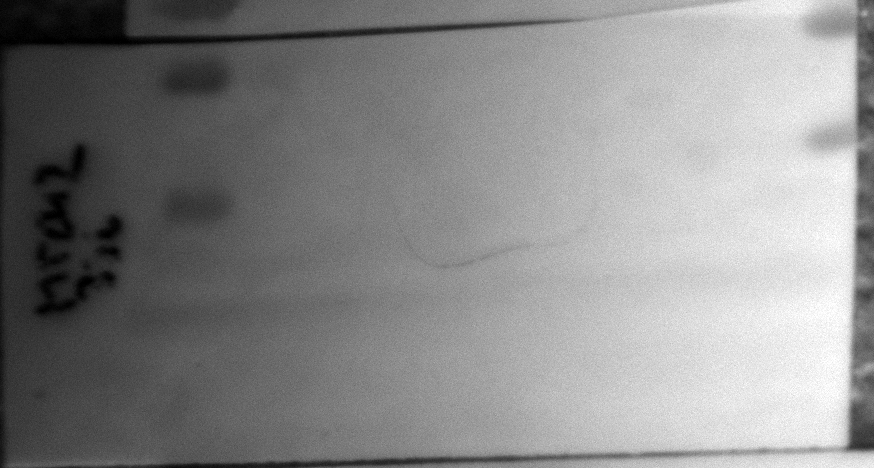

Supplement: Supplementary file 8 — Source Data EV Fig. 4 [file 44319_2023_9_MOESM8_ESM.zip › EV3/b/BLOTS/MTCH2/mtch2 membrane.tif]

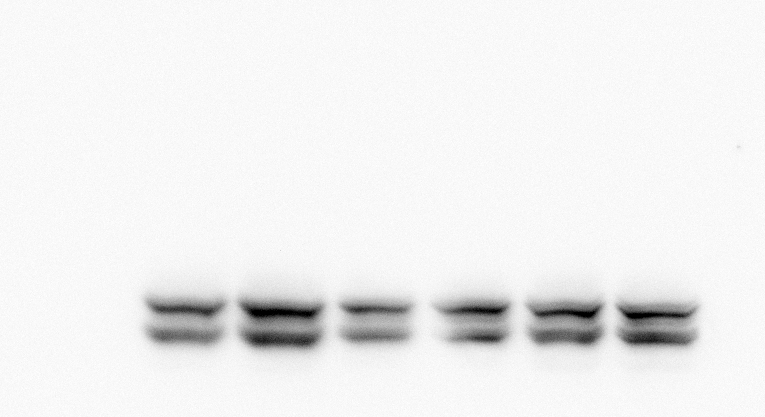

Supplement: Supplementary file 8 — Source Data EV Fig. 4 [file 44319_2023_9_MOESM8_ESM.zip › EV3/b/BLOTS/OPA1/opa1 blot.tif]

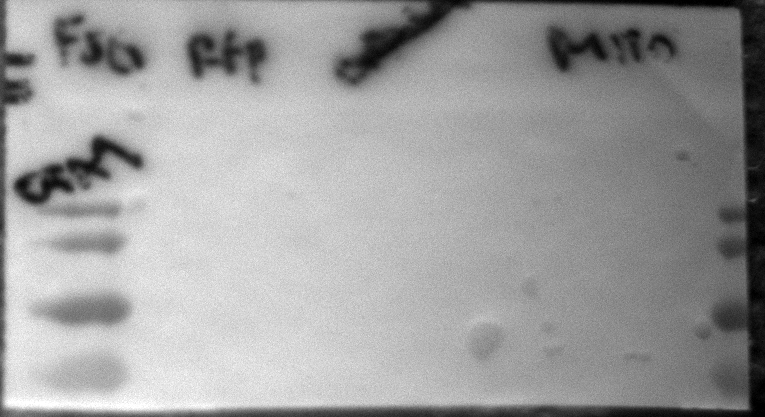

Supplement: Supplementary file 8 — Source Data EV Fig. 4 [file 44319_2023_9_MOESM8_ESM.zip › EV3/b/BLOTS/OPA1/opa1 membrane.tif]

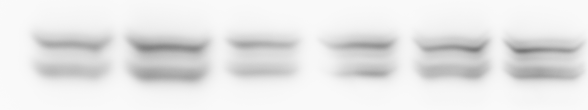

Supplement: Supplementary file 8 — Source Data EV Fig. 4 [file 44319_2023_9_MOESM8_ESM.zip › EV3/b/BLOTS/OPA1/OPA1.tif]

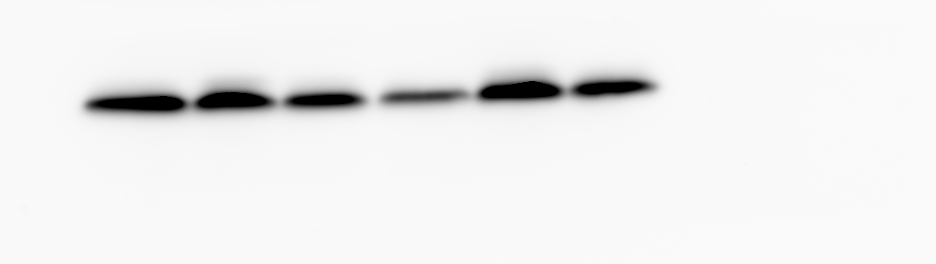

Supplement: Supplementary file 8 — Source Data EV Fig. 4 [file 44319_2023_9_MOESM8_ESM.zip › EV3/b/BLOTS/TOMM40/tomm40 blot.tif]

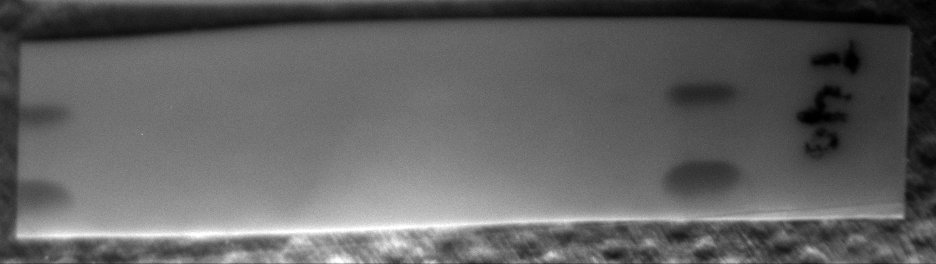

Supplement: Supplementary file 8 — Source Data EV Fig. 4 [file 44319_2023_9_MOESM8_ESM.zip › EV3/b/BLOTS/TOMM40/tomm40 membrane.tif]

# EV 3B FUSION FISSION PROTEINS AFTER GPATi

HM

LM

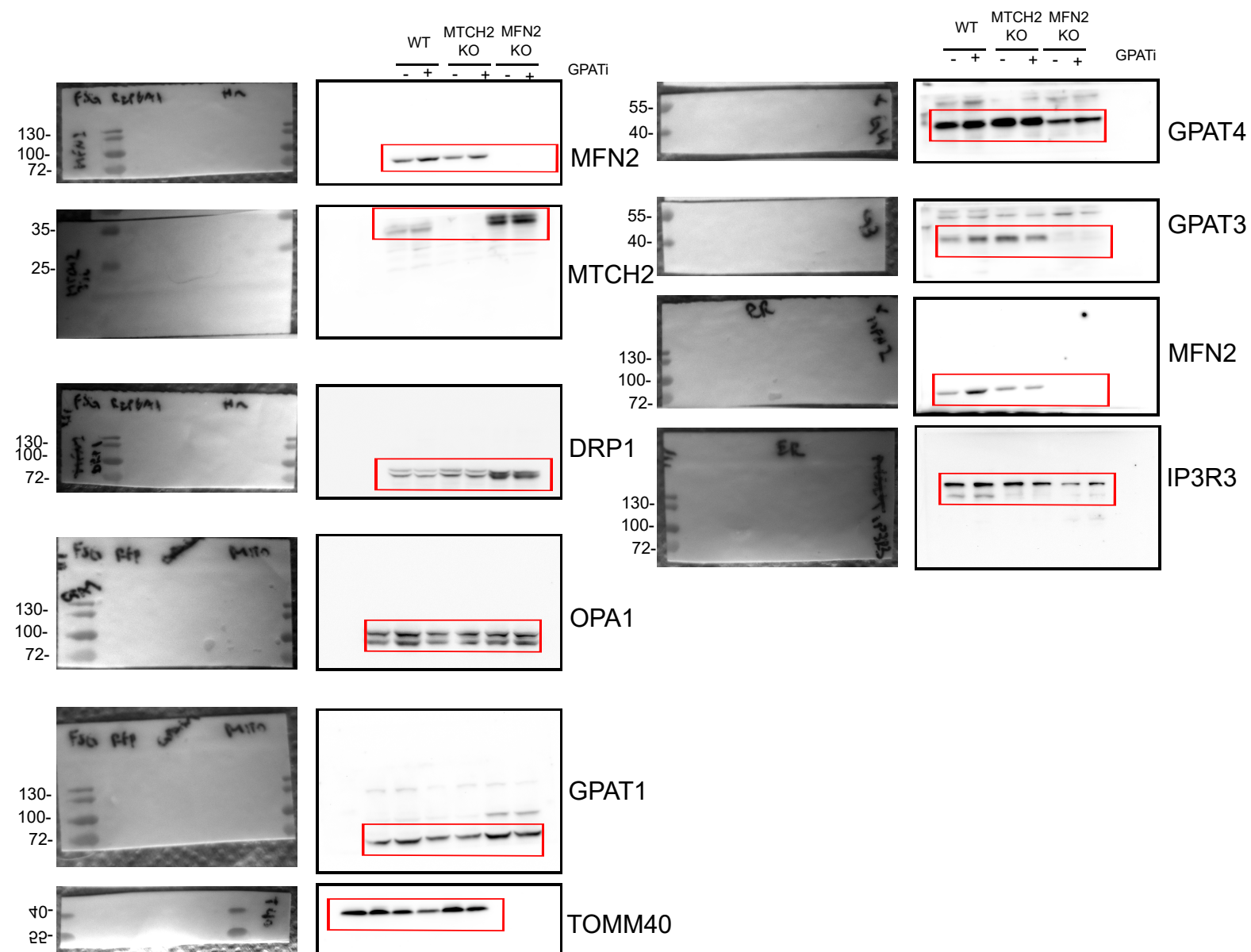

Supplement: Supplementary file 8 — Source Data EV Fig. 4 [file 44319_2023_9_MOESM8_ESM.zip › EV3/b/UNCROPED BLOTS EV3B.pdf]

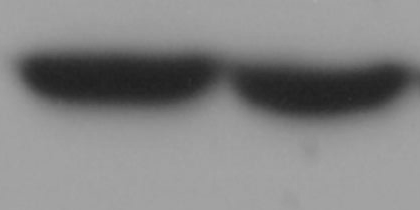

Supplement: Supplementary file 8 — Source Data EV Fig. 4 [file 44319_2023_9_MOESM8_ESM.zip › EV3/c/BLOTS/ATP5B/ATP5B NEW.tif]

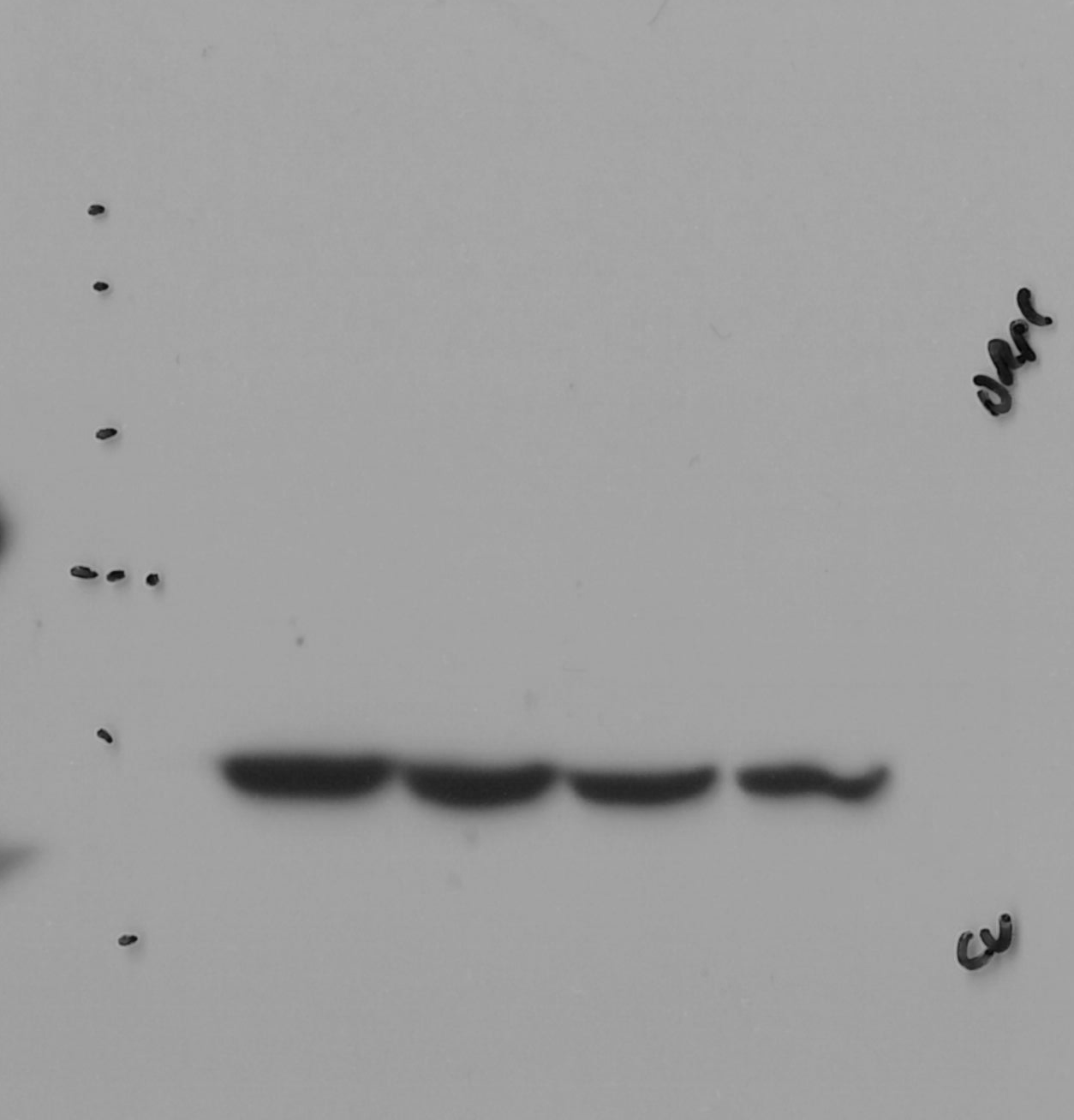

Supplement: Supplementary file 8 — Source Data EV Fig. 4 [file 44319_2023_9_MOESM8_ESM.zip › EV3/c/BLOTS/ATP5B/ATP5B WHOLE PLOT.tif]

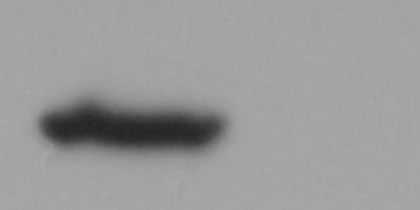

Supplement: Supplementary file 8 — Source Data EV Fig. 4 [file 44319_2023_9_MOESM8_ESM.zip › EV3/c/BLOTS/MTCH2/MTCH2 NEW.tif]

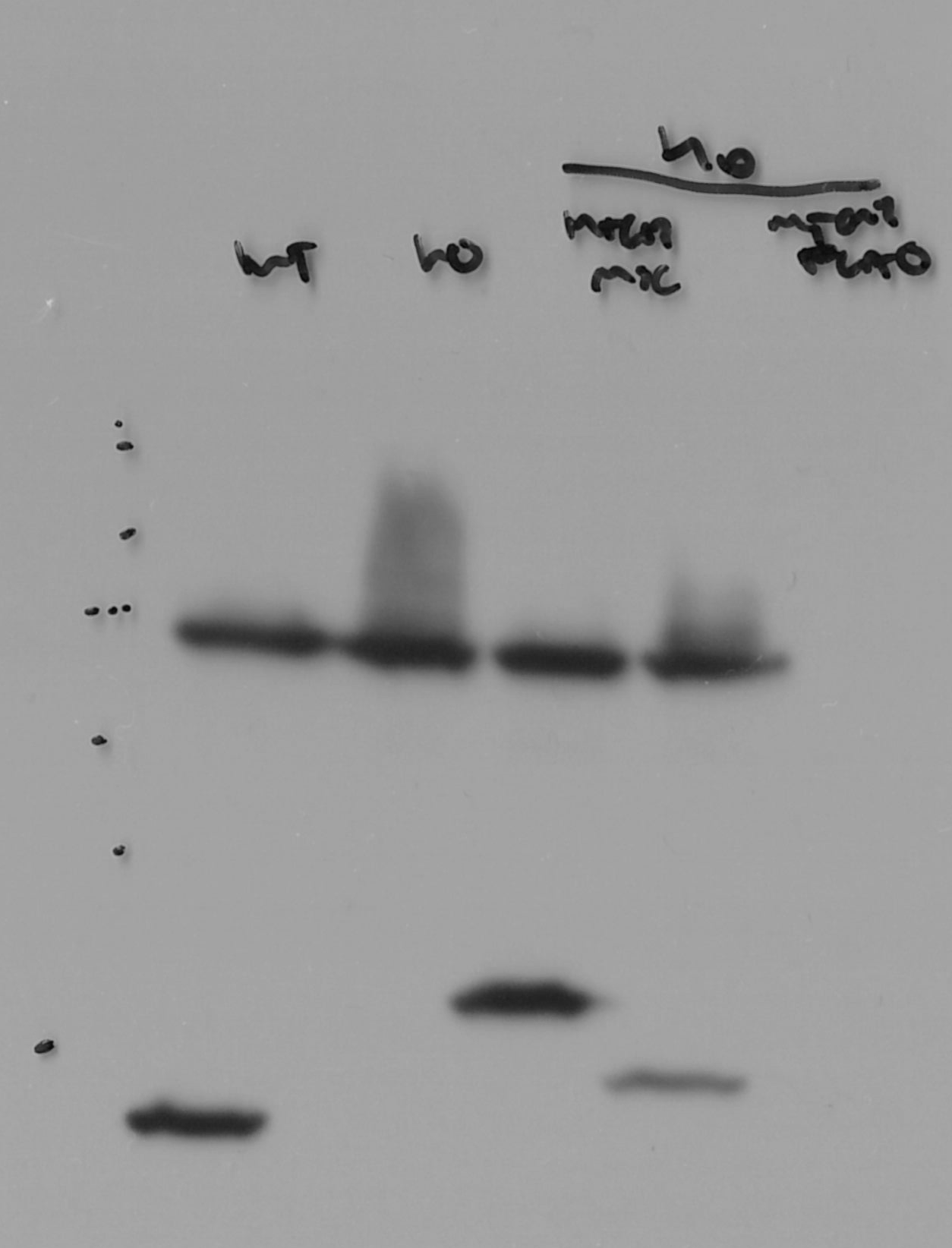

Supplement: Supplementary file 8 — Source Data EV Fig. 4 [file 44319_2023_9_MOESM8_ESM.zip › EV3/c/BLOTS/MTCH2/MTCH2 WHOLE PLOT.tif]

# EV 3C MTCH2 EXPRESSION IN HEK293T MTCH2 KO

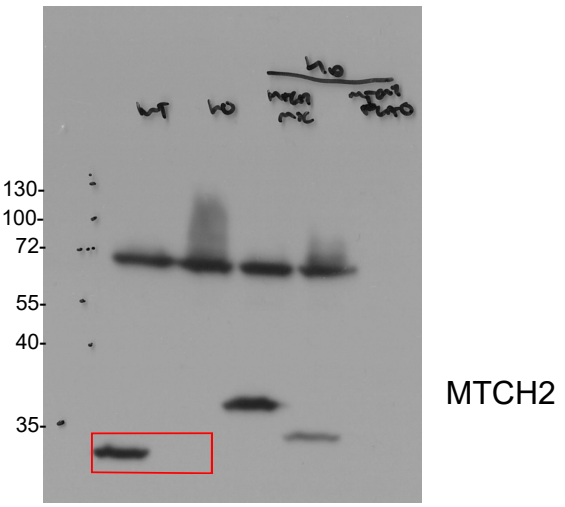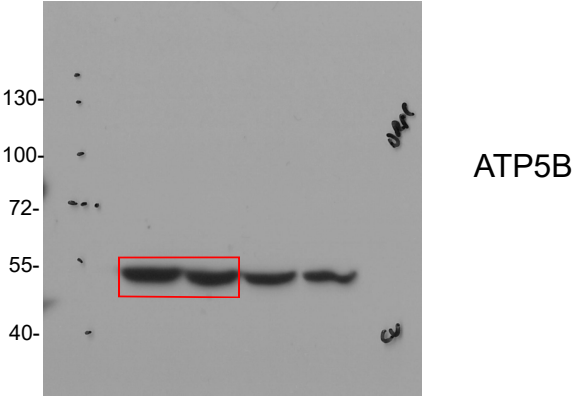

Supplement: Supplementary file 8 — Source Data EV Fig. 4 [file 44319_2023_9_MOESM8_ESM.zip › EV3/c/UNCROPED BLOTS EV3C.pdf]

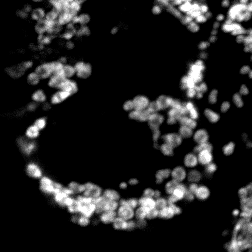

Supplement: Supplementary file 8 — Source Data EV Fig. 4 [file 44319_2023_9_MOESM8_ESM.zip › EV3/d/IMAGES/MTCH2 KO/MAX_293 Mtch2 KO006-1-1.tif]

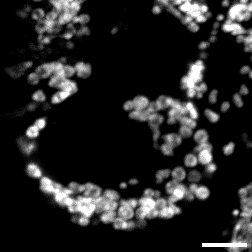

Supplement: Supplementary file 8 — Source Data EV Fig. 4 [file 44319_2023_9_MOESM8_ESM.zip › EV3/d/IMAGES/MTCH2 KO/MAX_293 Mtch2 KO006-1-2 5um scale bar.tif]

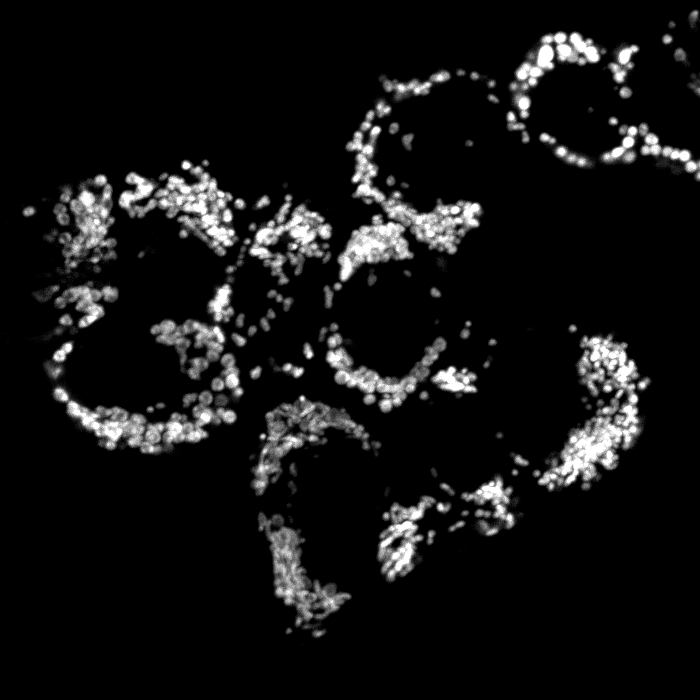

Supplement: Supplementary file 8 — Source Data EV Fig. 4 [file 44319_2023_9_MOESM8_ESM.zip › EV3/d/IMAGES/MTCH2 KO/MAX_293 Mtch2 KO006-1.tif]

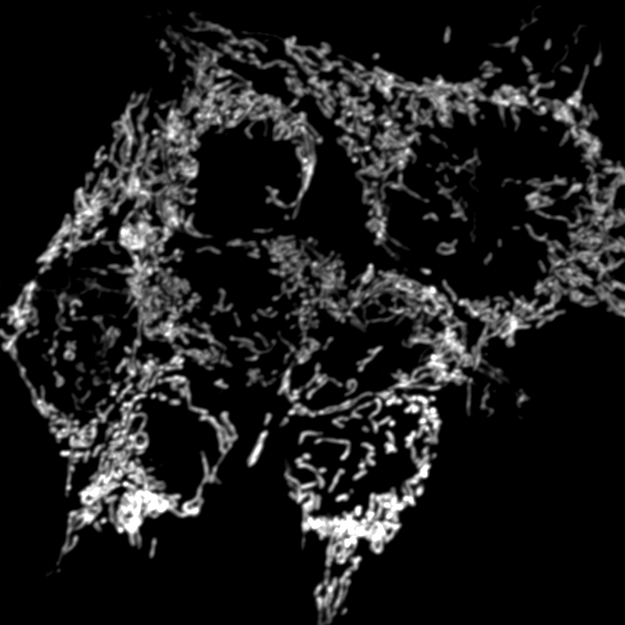

Supplement: Supplementary file 8 — Source Data EV Fig. 4 [file 44319_2023_9_MOESM8_ESM.zip › EV3/d/IMAGES/WT/MAX_293 WT013-5 greys-1.tif]

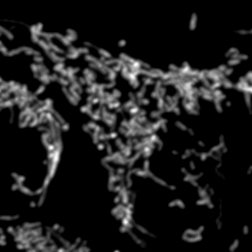

Supplement: Supplementary file 8 — Source Data EV Fig. 4 [file 44319_2023_9_MOESM8_ESM.zip › EV3/d/IMAGES/WT/MAX_293 WT013-5 greys-2.tif]

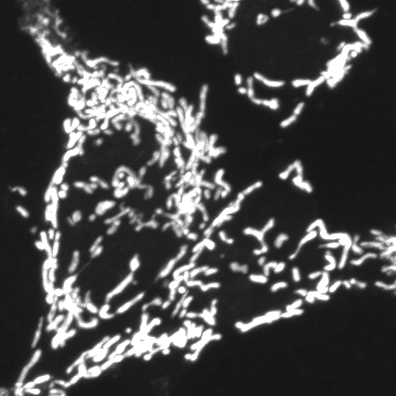

Supplement: Supplementary file 8 — Source Data EV Fig. 4 [file 44319_2023_9_MOESM8_ESM.zip › EV3/g/IMAGES/CONTROL/MAX_293tWT CTR1 MTDR007-1.tif]

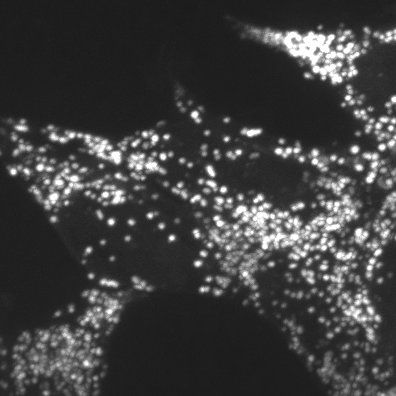

Supplement: Supplementary file 8 — Source Data EV Fig. 4 [file 44319_2023_9_MOESM8_ESM.zip › EV3/g/IMAGES/GPATi/MAX_293t WT FSG67 150uM 18H MTDR-1.tif]

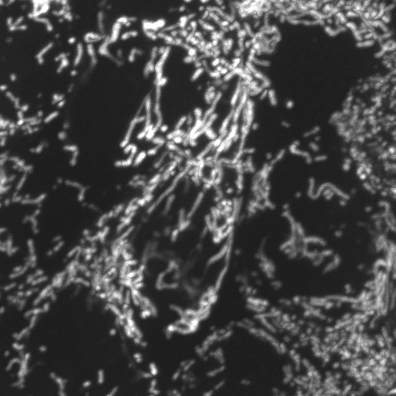

Supplement: Supplementary file 8 — Source Data EV Fig. 4 [file 44319_2023_9_MOESM8_ESM.zip › EV3/g/IMAGES/WASH/MAX_293t WT CTR1 WASHED 4H MTDR009-1.tif]

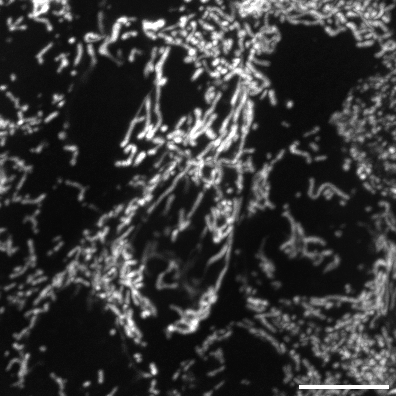

Supplement: Supplementary file 8 — Source Data EV Fig. 4 [file 44319_2023_9_MOESM8_ESM.zip › EV3/g/IMAGES/WASH/MAX_293t WT CTR1 WASHED 4H MTDR009-3 10um scale bar.tif]

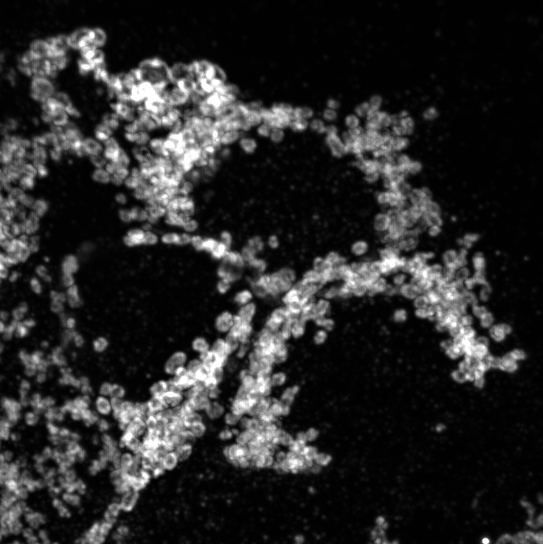

Supplement: Supplementary file 8 — Source Data EV Fig. 4 [file 44319_2023_9_MOESM8_ESM.zip › EV3/i/IMAGES/MTCH2 KO/MAX_hek293 mtch2 ko non transfected27_thumb_w1Con-Cy5-1-1 MTCH2 KO CONTROL.tif]

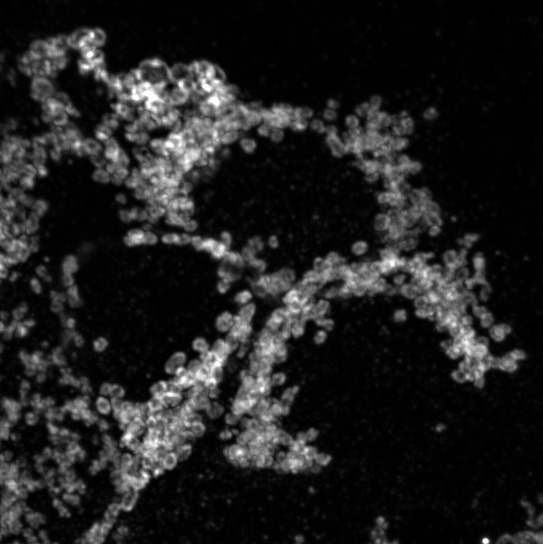

Supplement: Supplementary file 8 — Source Data EV Fig. 4 [file 44319_2023_9_MOESM8_ESM.zip › EV3/i/IMAGES/MTCH2 KO/MAX_hek293 mtch2 ko non transfected27_thumb_w1Con-Cy5-1-1.tif]

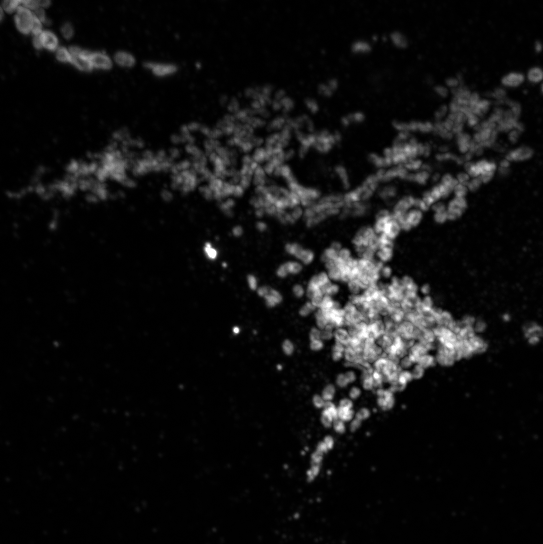

Supplement: Supplementary file 8 — Source Data EV Fig. 4 [file 44319_2023_9_MOESM8_ESM.zip › EV3/i/IMAGES/MTCH2 KO/MTCH2 OE MTCH2 KO.tif]

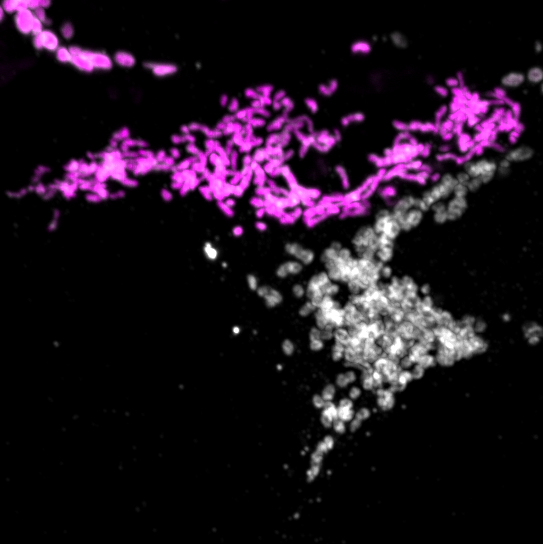

Supplement: Supplementary file 8 — Source Data EV Fig. 4 [file 44319_2023_9_MOESM8_ESM.zip › EV3/i/IMAGES/MTCH2 KO/MTCH2 OE MTCH2 KO.tif (RGB)COMP.tif]

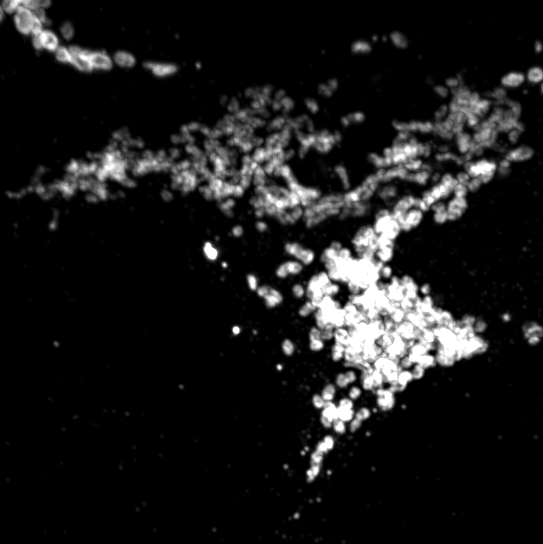

Supplement: Supplementary file 8 — Source Data EV Fig. 4 [file 44319_2023_9_MOESM8_ESM.zip › EV3/i/IMAGES/MTCH2 KO/MTCH2 OE MTCH2 KO.tif (RGB)MITO.tif]

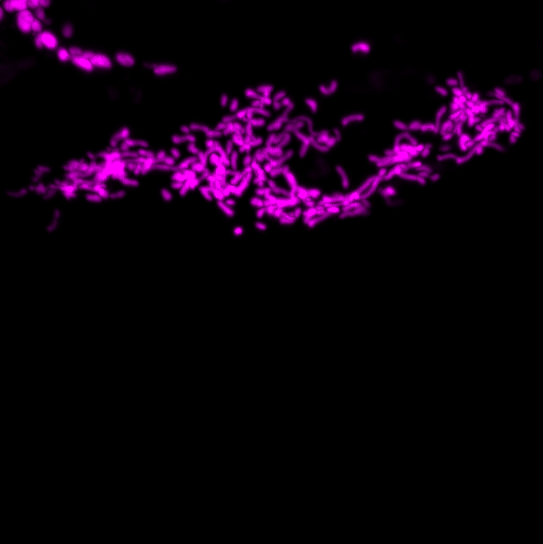

Supplement: Supplementary file 8 — Source Data EV Fig. 4 [file 44319_2023_9_MOESM8_ESM.zip › EV3/i/IMAGES/MTCH2 KO/MTCH2 OE MTCH2 KO.tif (RGB)MTCH.tif]

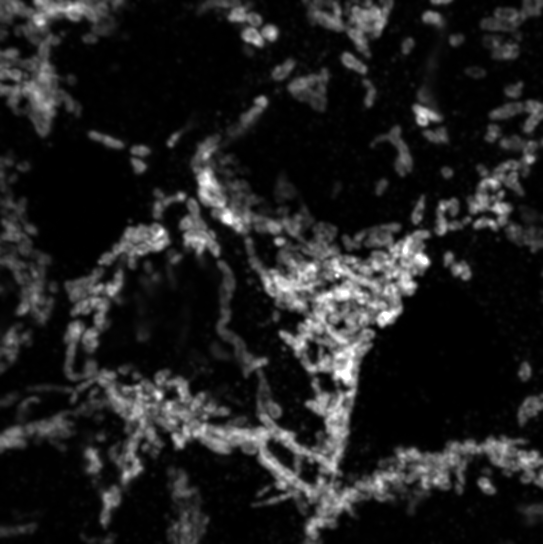

Supplement: Supplementary file 8 — Source Data EV Fig. 4 [file 44319_2023_9_MOESM8_ESM.zip › EV3/i/IMAGES/WT CTRL/MAX_hek293 wt control MTCH2 GFP mitods red 11_thumb_w1Con-mcherry-1.TIF-1.tif]

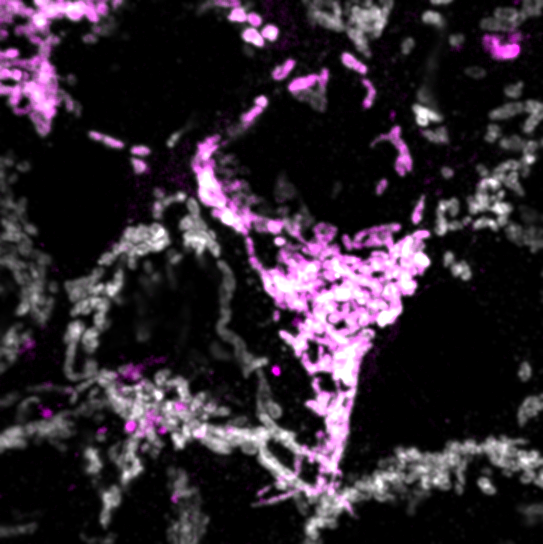

Supplement: Supplementary file 8 — Source Data EV Fig. 4 [file 44319_2023_9_MOESM8_ESM.zip › EV3/i/IMAGES/WT CTRL/MAX_hek293 wt control MTCH2 GFP mitods red 11_thumb_w1Con-mcherry-1.TIF-1.tif (RGB) COMPO.tif]

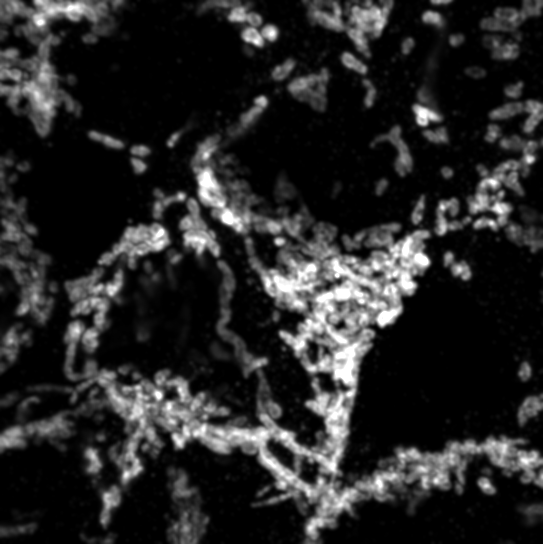

Supplement: Supplementary file 8 — Source Data EV Fig. 4 [file 44319_2023_9_MOESM8_ESM.zip › EV3/i/IMAGES/WT CTRL/MAX_hek293 wt control MTCH2 GFP mitods red 11_thumb_w1Con-mcherry-1.TIF-1.tif (RGB) MITO.tif]

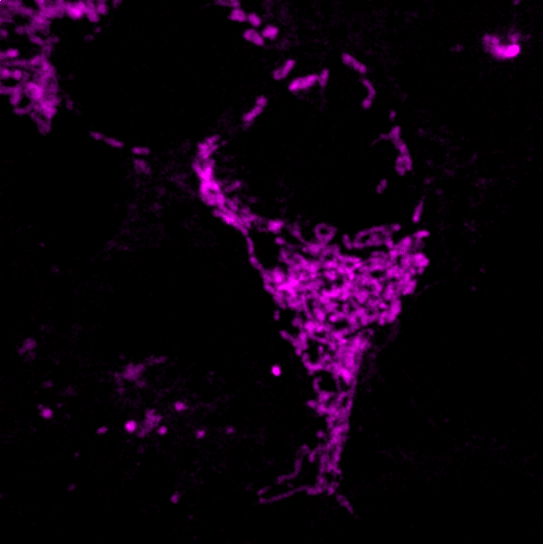

Supplement: Supplementary file 8 — Source Data EV Fig. 4 [file 44319_2023_9_MOESM8_ESM.zip › EV3/i/IMAGES/WT CTRL/MAX_hek293 wt control MTCH2 GFP mitods red 11_thumb_w1Con-mcherry-1.TIF-1.tif (RGB) MTCH2.tif]

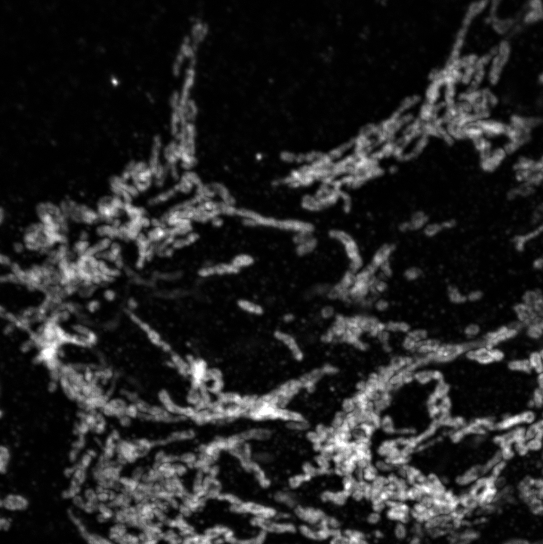

Supplement: Supplementary file 8 — Source Data EV Fig. 4 [file 44319_2023_9_MOESM8_ESM.zip › EV3/i/IMAGES/WT CTRL/WT CONTROLMAX_hek293 wt non transfected14_thumb_w1Con-Cy5-1-1.tif]
