# Supplementary figures and images for: MTCH2 cooperates with MFN2 and lysophosphatidic acid synthesis to sustain mitochondrial fusion (part 6 of 6)
Source: EMBO Rep. 2023 Dec 14;25(1):8. doi: 10.1038/s44319-023-00009-1 (PMC10897490; doi:10.1038/s44319-023-00009-1)

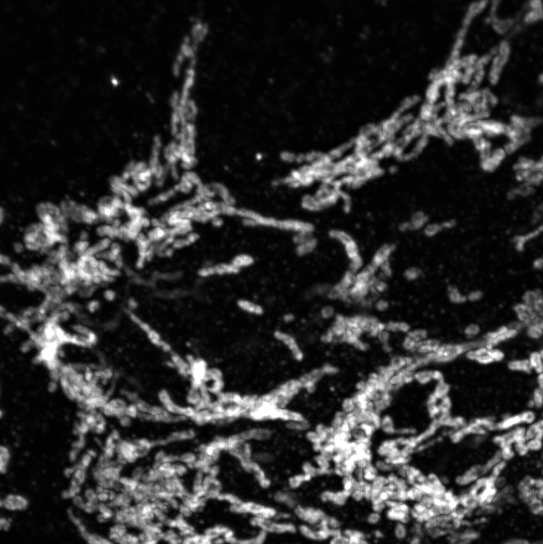

Supplement: Supplementary file 8 — Source Data EV Fig. 4 [file 44319_2023_9_MOESM8_ESM.zip › EV3/i/IMAGES/WT CTRL/WT CTRL2.tif]

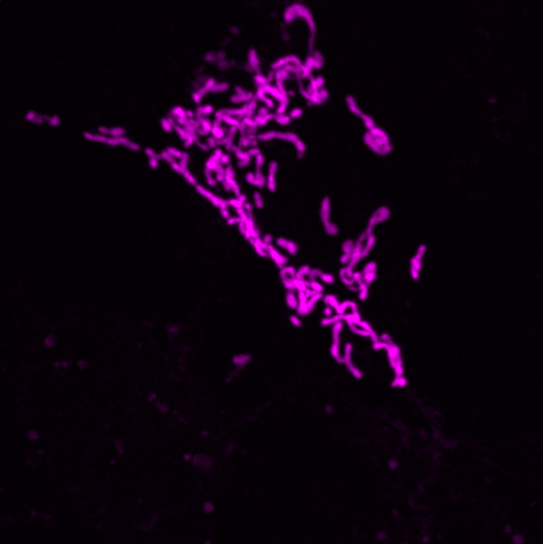

Supplement: Supplementary file 8 — Source Data EV Fig. 4 [file 44319_2023_9_MOESM8_ESM.zip › EV3/i/IMAGES/WT GPATi/mtch2 oe fsg MAX_hek293 MTCH2 GFP mitods red 20_thumb_w1Con-mcherry-1.TIF-1 MTCH2.tif]

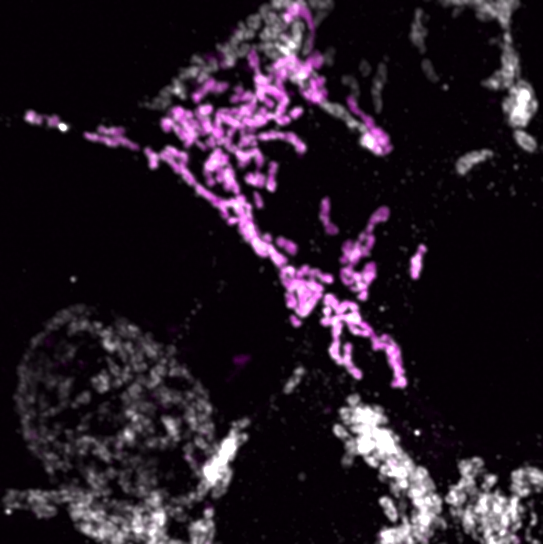

Supplement: Supplementary file 8 — Source Data EV Fig. 4 [file 44319_2023_9_MOESM8_ESM.zip › EV3/i/IMAGES/WT GPATi/mtch2 oe fsg MAX_hek293 MTCH2 GFP mitods red 20_thumb_w1Con-mcherry-1.TIF-1(RGB) COMPO.tif]

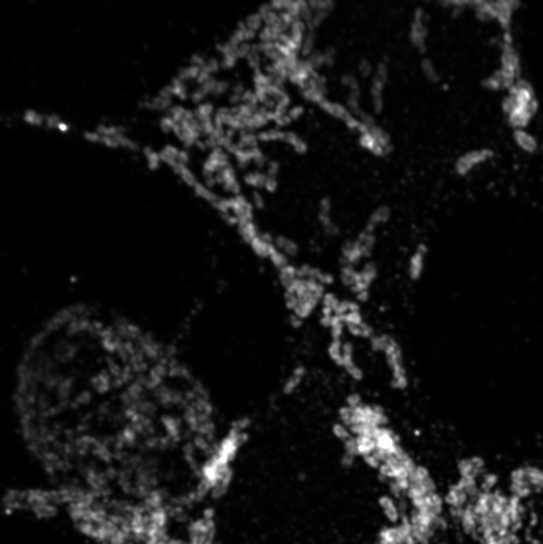

Supplement: Supplementary file 8 — Source Data EV Fig. 4 [file 44319_2023_9_MOESM8_ESM.zip › EV3/i/IMAGES/WT GPATi/mtch2 oe fsg MAX_hek293 MTCH2 GFP mitods red 20_thumb_w1Con-mcherry-1.TIF-1.tif]

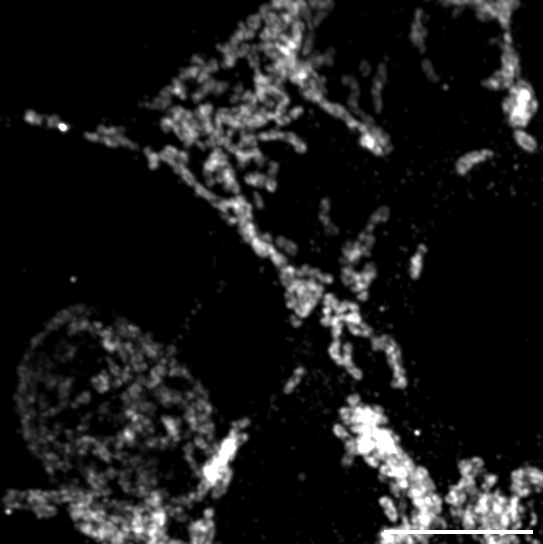

Supplement: Supplementary file 8 — Source Data EV Fig. 4 [file 44319_2023_9_MOESM8_ESM.zip › EV3/i/IMAGES/WT GPATi/mtch2 oe fsg MAX_hek293 MTCH2 GFP mitods red 20_thumb_w1Con-mcherry-1.TIF-1MITO-1 scale.tif]

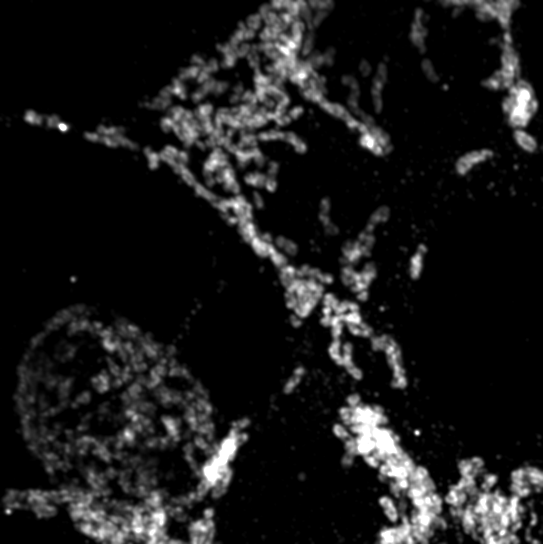

Supplement: Supplementary file 8 — Source Data EV Fig. 4 [file 44319_2023_9_MOESM8_ESM.zip › EV3/i/IMAGES/WT GPATi/mtch2 oe fsg MAX_hek293 MTCH2 GFP mitods red 20_thumb_w1Con-mcherry-1.TIF-1MITO.tif]

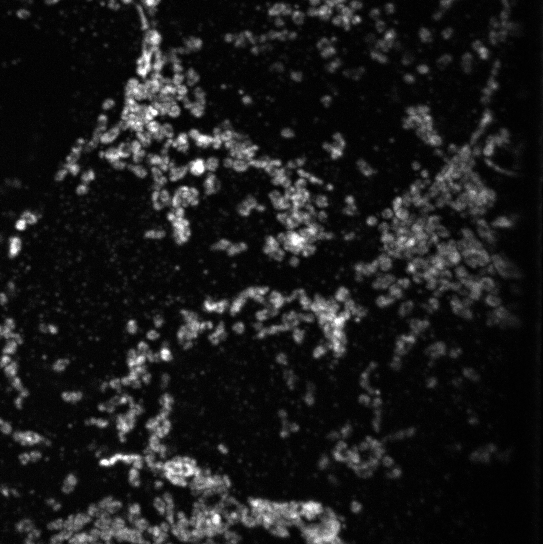

Supplement: Supplementary file 8 — Source Data EV Fig. 4 [file 44319_2023_9_MOESM8_ESM.zip › EV3/i/IMAGES/WT GPATi/WT FSG67 MAX_C1-hek wt drp1 dn fsg68_thumb_w1Con-Cy5-1-1.tif]

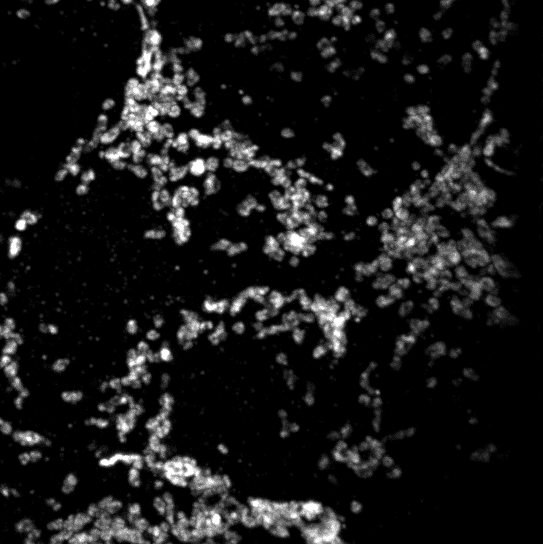

Supplement: Supplementary file 8 — Source Data EV Fig. 4 [file 44319_2023_9_MOESM8_ESM.zip › EV3/i/IMAGES/WT GPATi/WT FSG672.tif]

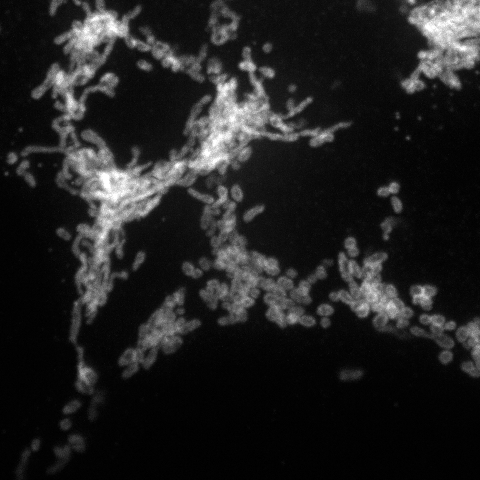

Supplement: Supplementary file 8 — Source Data EV Fig. 4 [file 44319_2023_9_MOESM8_ESM.zip › EV3/l/IMAGES/CONTROL/MAX_mtch2 ko control mfn2 myc cy5 tom40 cy11_thumb_w1Con-mcherry-1.TIF-1.tif]

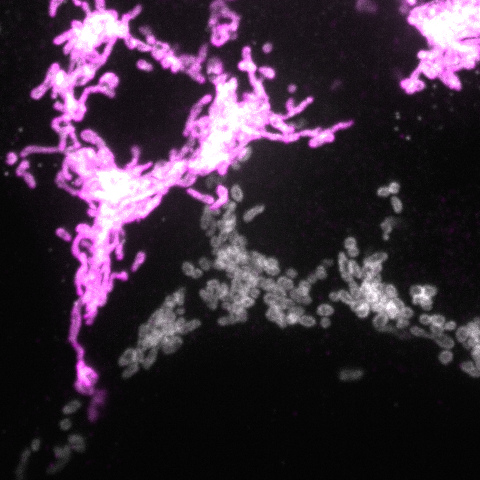

Supplement: Supplementary file 8 — Source Data EV Fig. 4 [file 44319_2023_9_MOESM8_ESM.zip › EV3/l/IMAGES/CONTROL/MAX_mtch2 ko control mfn2 myc cy5 tom40 cy11_thumb_w1Con-mcherry-1.TIF-1.tif (RGB) COMP.tif]

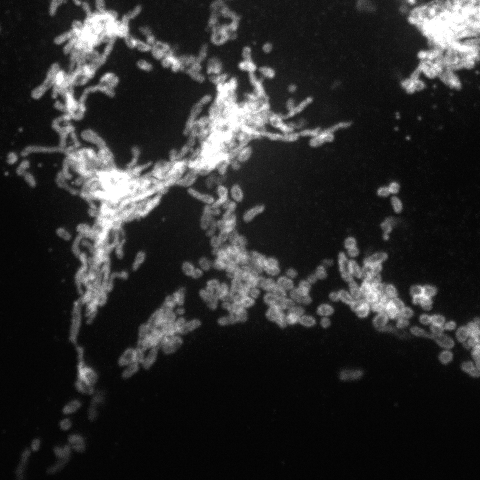

Supplement: Supplementary file 8 — Source Data EV Fig. 4 [file 44319_2023_9_MOESM8_ESM.zip › EV3/l/IMAGES/CONTROL/MAX_mtch2 ko control mfn2 myc cy5 tom40 cy11_thumb_w1Con-mcherry-1.TIF-1.tif (RGB) MITO.tif]

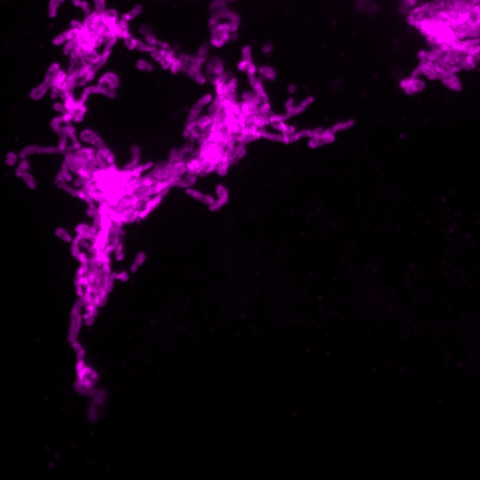

Supplement: Supplementary file 8 — Source Data EV Fig. 4 [file 44319_2023_9_MOESM8_ESM.zip › EV3/l/IMAGES/CONTROL/MAX_mtch2 ko control mfn2 myc cy5 tom40 cy11_thumb_w1Con-mcherry-1.TIF-1.tif (RGB) MTH.tif]

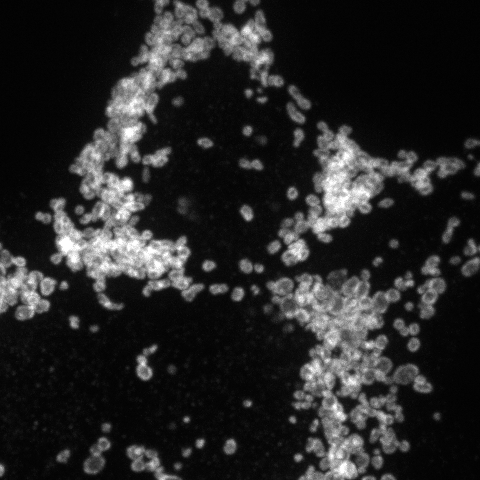

Supplement: Supplementary file 8 — Source Data EV Fig. 4 [file 44319_2023_9_MOESM8_ESM.zip › EV3/l/IMAGES/CONTROL/MAX_mtch2 ko control tom40 cy3_thumb_w1Con-mcherry-1.TIF-1.tif]

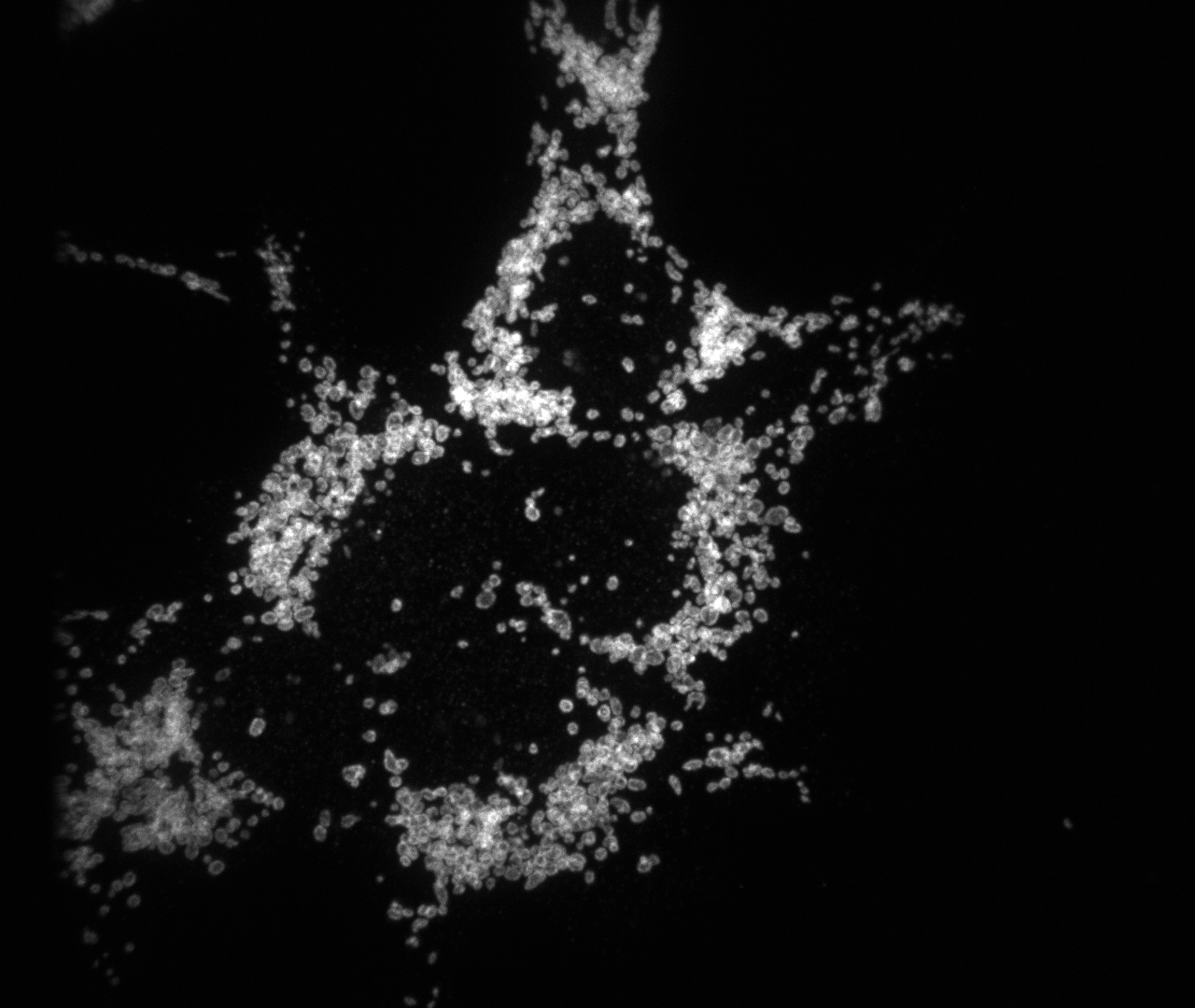

Supplement: Supplementary file 8 — Source Data EV Fig. 4 [file 44319_2023_9_MOESM8_ESM.zip › EV3/l/IMAGES/CONTROL/MAX_mtch2 ko control tom40 cy3_thumb_w1Con-mcherry-1.TIF.tif]

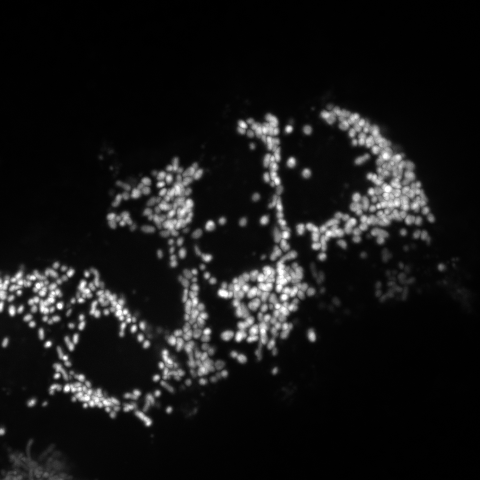

Supplement: Supplementary file 8 — Source Data EV Fig. 4 [file 44319_2023_9_MOESM8_ESM.zip › EV3/l/IMAGES/GPATi/MAX_293 mtch2 KO 3_thumb_w1Con-Cy5-1.tif]

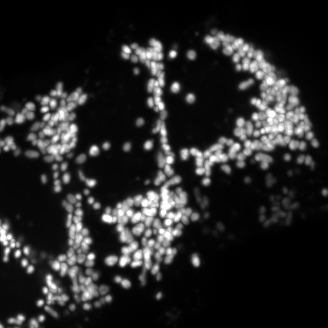

Supplement: Supplementary file 8 — Source Data EV Fig. 4 [file 44319_2023_9_MOESM8_ESM.zip › EV3/l/IMAGES/GPATi/MAX_293 mtch2 KO 3_thumb_w1Con-Cy5-1KO FSG-1.tif]

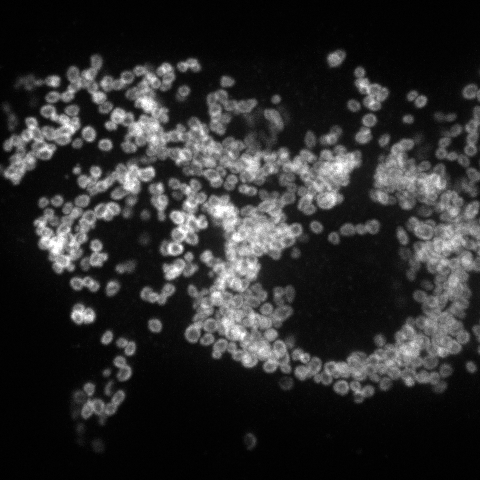

Supplement: Supplementary file 8 — Source Data EV Fig. 4 [file 44319_2023_9_MOESM8_ESM.zip › EV3/l/IMAGES/GPATi/MAX_mtch2 ko fgs57 150um on mfn2 myc cy5 tom40 cy23_thumb_w1Con-mcherry-1.TIF-1.tif]

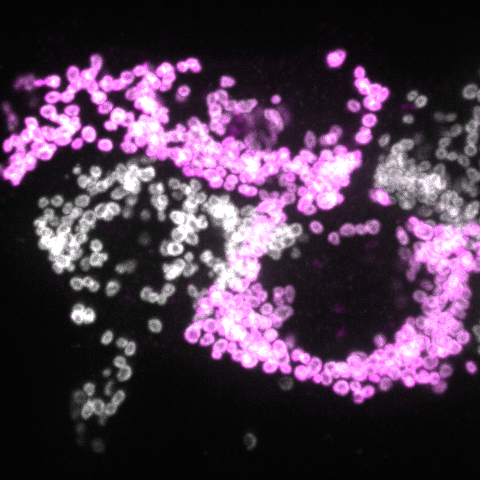

Supplement: Supplementary file 8 — Source Data EV Fig. 4 [file 44319_2023_9_MOESM8_ESM.zip › EV3/l/IMAGES/GPATi/MAX_mtch2 ko fgs57 150um on mfn2 myc cy5 tom40 cy23_thumb_w1Con-mcherry-1.TIF-1.tif (RGB) COMP.tif]

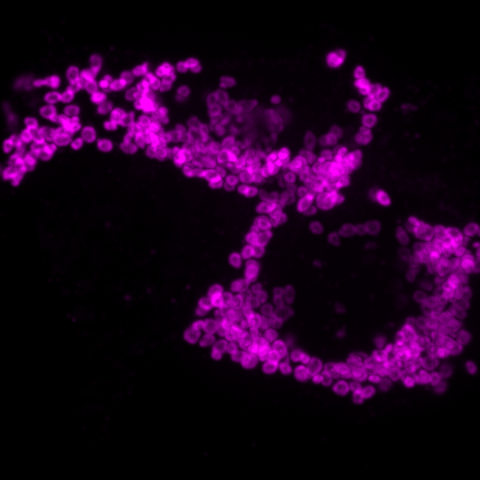

Supplement: Supplementary file 8 — Source Data EV Fig. 4 [file 44319_2023_9_MOESM8_ESM.zip › EV3/l/IMAGES/GPATi/MAX_mtch2 ko fgs57 150um on mfn2 myc cy5 tom40 cy23_thumb_w1Con-mcherry-1.TIF-1.tif (RGB).tif]

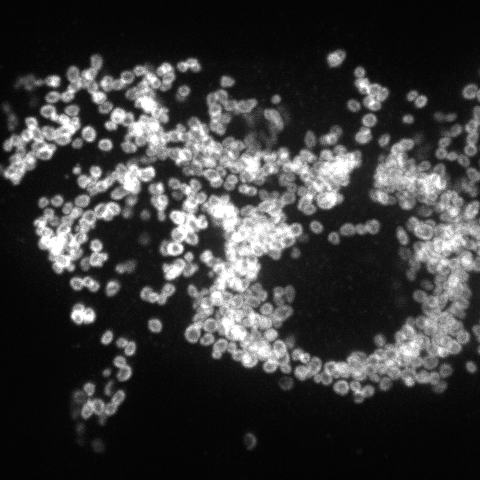

Supplement: Supplementary file 8 — Source Data EV Fig. 4 [file 44319_2023_9_MOESM8_ESM.zip › EV3/l/IMAGES/GPATi/MAX_mtch2 ko fgs57 150um on mfn2 myc cy5 tom40 cy23_thumb_w1Con-mcherry-1.TIF-1.tif (RGBMITO).tif]

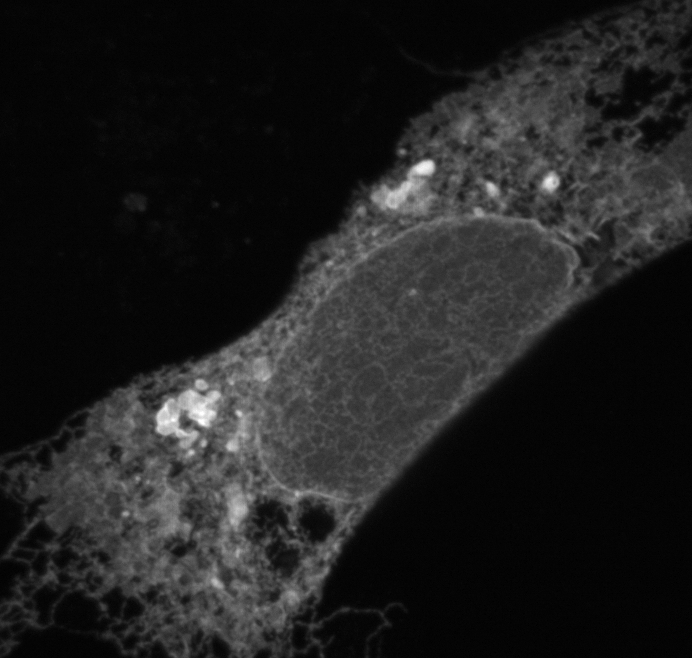

Supplement: Supplementary file 8 — Source Data EV Fig. 4 [file 44319_2023_9_MOESM8_ESM.zip › EV3/o/images/MFN2 ACTA/ctrl/MAX_MEFS MTCH2 ko acta flag598 er gfp mitobfp mrpl12 dapi60_thumb_w1Con-mcherry-1.tif]

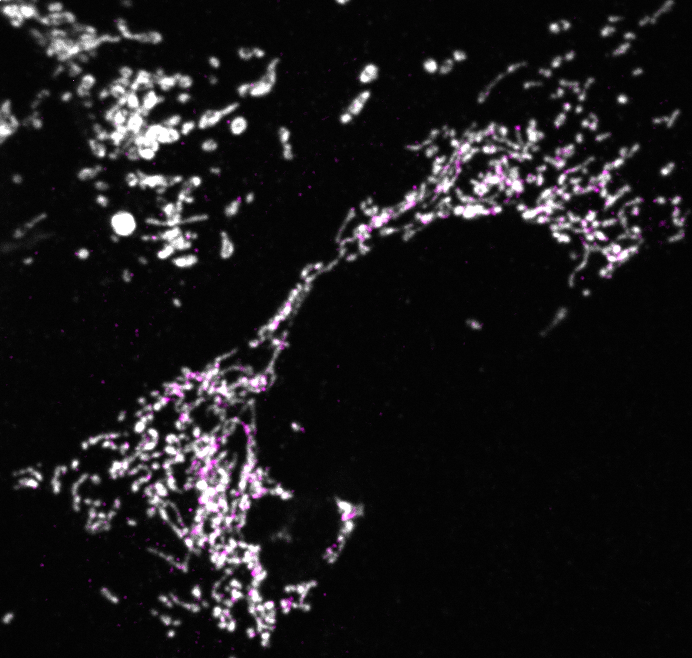

Supplement: Supplementary file 8 — Source Data EV Fig. 4 [file 44319_2023_9_MOESM8_ESM.zip › EV3/o/images/MFN2 ACTA/ctrl/MAX_MEFS MTCH2 ko acta flag598 er gfp mitobfp mrpl12 dapi60_thumb_w1Con-mcherry-1.tif (RGB) ch.tif]

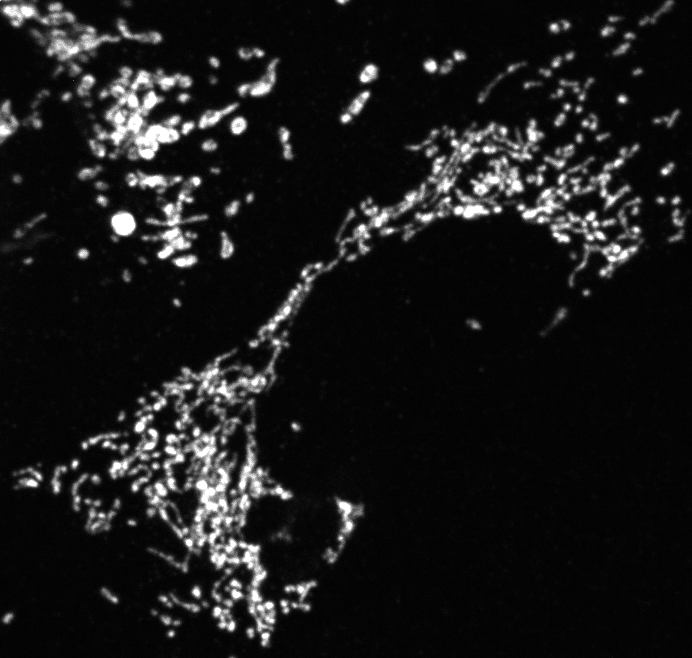

Supplement: Supplementary file 8 — Source Data EV Fig. 4 [file 44319_2023_9_MOESM8_ESM.zip › EV3/o/images/MFN2 ACTA/ctrl/MAX_MEFS MTCH2 ko acta flag598 er gfp mitobfp mrpl12 dapi60_thumb_w1Con-mcherry-1.tif (RGB) ch1.tif]

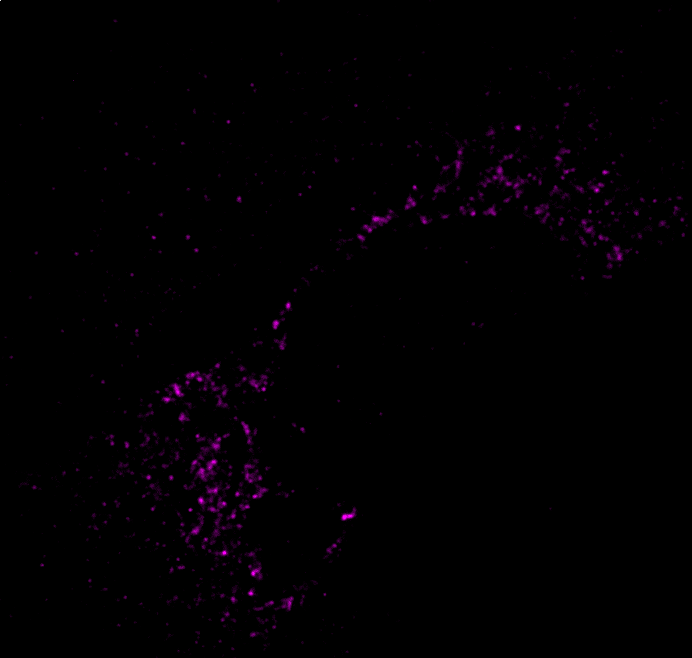

Supplement: Supplementary file 8 — Source Data EV Fig. 4 [file 44319_2023_9_MOESM8_ESM.zip › EV3/o/images/MFN2 ACTA/ctrl/MAX_MEFS MTCH2 ko acta flag598 er gfp mitobfp mrpl12 dapi60_thumb_w1Con-mcherry-1.tif (RGB).tif]

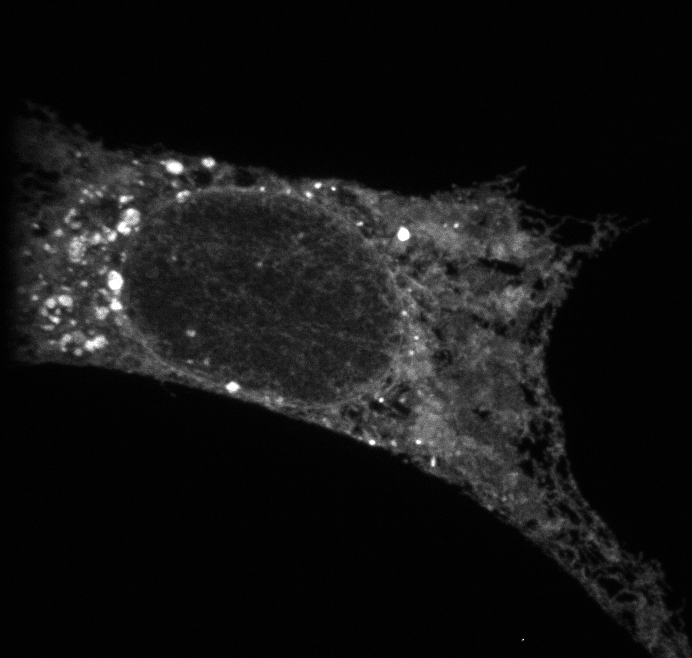

Supplement: Supplementary file 8 — Source Data EV Fig. 4 [file 44319_2023_9_MOESM8_ESM.zip › EV3/o/images/MFN2 ACTA/fsg67/MAX_mtch2 ko flag acta 598 ER GFP TOM633 dapi4_thumb_w1Con-mcherry-1.tif]

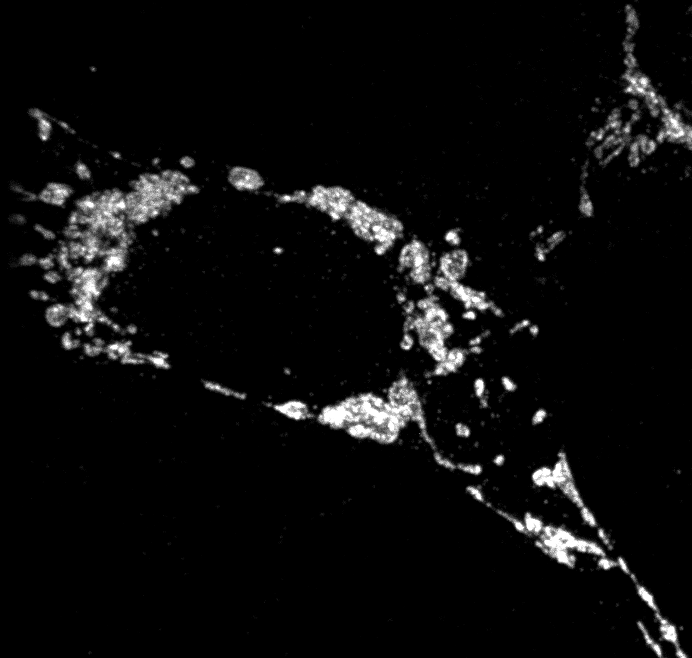

Supplement: Supplementary file 8 — Source Data EV Fig. 4 [file 44319_2023_9_MOESM8_ESM.zip › EV3/o/images/MFN2 ACTA/fsg67/MAX_mtch2 ko flag acta 598 ER GFP TOM633 dapi4_thumb_w1Con-mcherry-1.tif (RGB) ch1.tif]

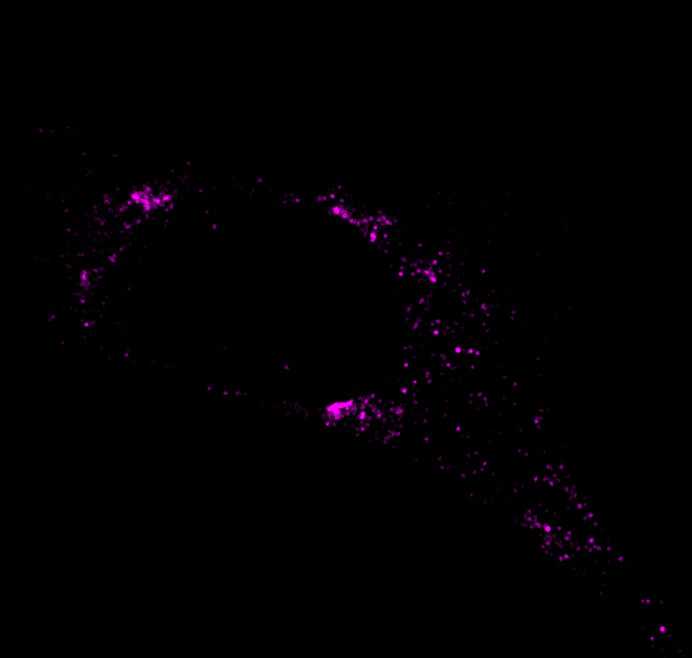

Supplement: Supplementary file 8 — Source Data EV Fig. 4 [file 44319_2023_9_MOESM8_ESM.zip › EV3/o/images/MFN2 ACTA/fsg67/MAX_mtch2 ko flag acta 598 ER GFP TOM633 dapi4_thumb_w1Con-mcherry-1.tif (RGB) ch3.tif]

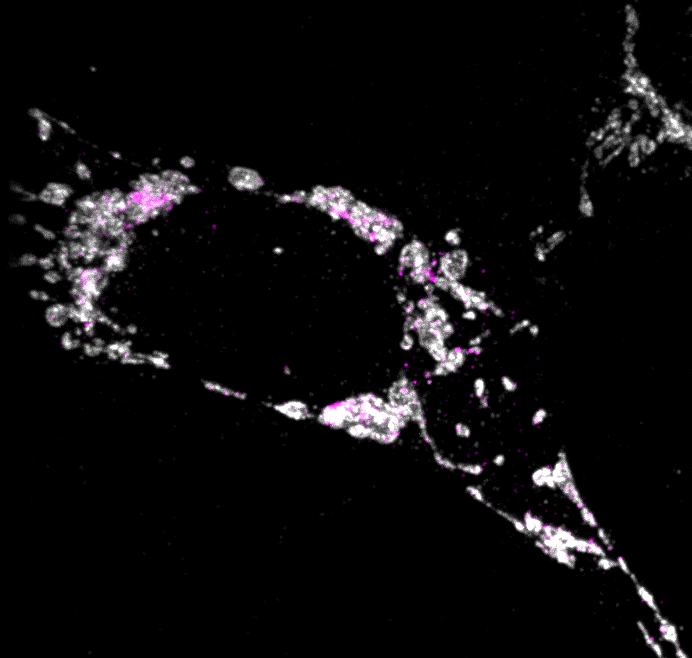

Supplement: Supplementary file 8 — Source Data EV Fig. 4 [file 44319_2023_9_MOESM8_ESM.zip › EV3/o/images/MFN2 ACTA/fsg67/MAX_mtch2 ko flag acta 598 ER GFP TOM633 dapi4_thumb_w1Con-mcherry-1.tif (RGB) comp.tif]

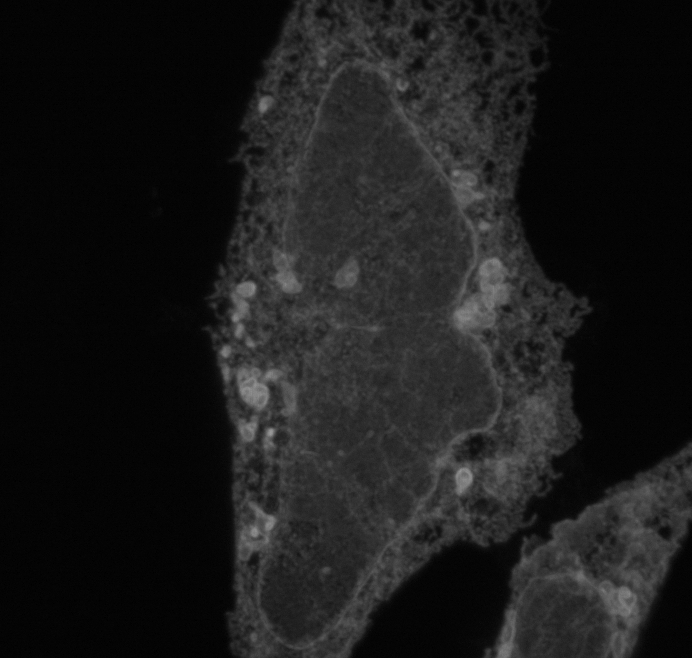

Supplement: Supplementary file 8 — Source Data EV Fig. 4 [file 44319_2023_9_MOESM8_ESM.zip › EV3/o/images/MFN2 IYFFT/control/MAX_MEFs MTCH2 KO MFN2 YIFFT FLAG 598 ER GFP mitoBFP pdh 634_thumb_w1Con-mcherry-1.tif]

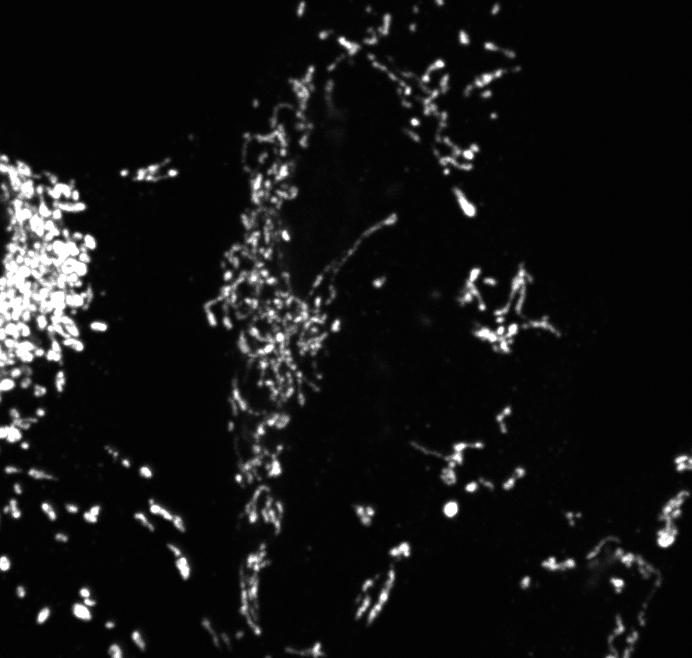

Supplement: Supplementary file 8 — Source Data EV Fig. 4 [file 44319_2023_9_MOESM8_ESM.zip › EV3/o/images/MFN2 IYFFT/control/MAX_MEFs MTCH2 KO MFN2 YIFFT FLAG 598 ER GFP mitoBFP pdh 634_thumb_w1Con-mcherry-1.tif (RGB) ch2.tif]

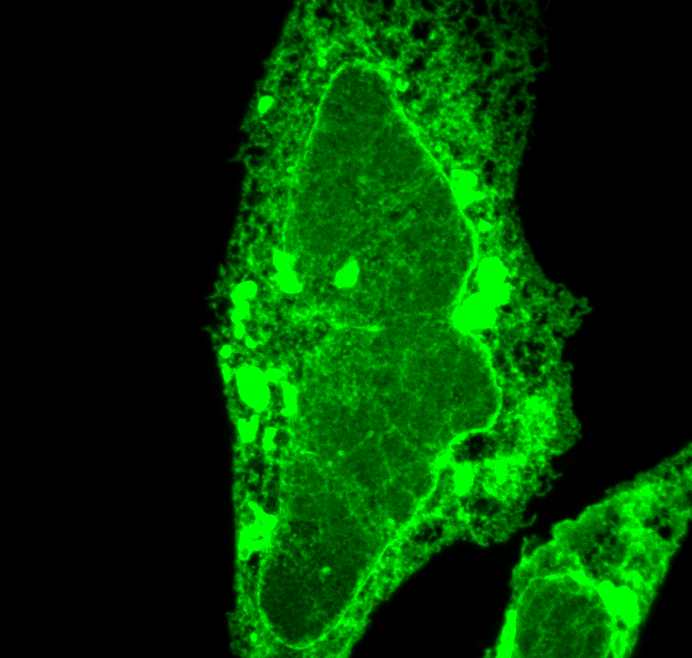

Supplement: Supplementary file 8 — Source Data EV Fig. 4 [file 44319_2023_9_MOESM8_ESM.zip › EV3/o/images/MFN2 IYFFT/control/MAX_MEFs MTCH2 KO MFN2 YIFFT FLAG 598 ER GFP mitoBFP pdh 634_thumb_w1Con-mcherry-1.tif (RGB) ch3.tif]

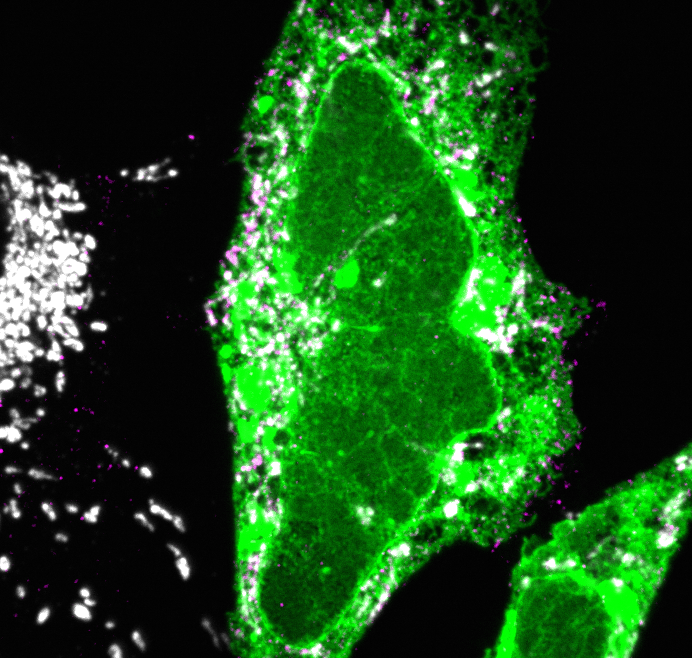

Supplement: Supplementary file 8 — Source Data EV Fig. 4 [file 44319_2023_9_MOESM8_ESM.zip › EV3/o/images/MFN2 IYFFT/control/MAX_MEFs MTCH2 KO MFN2 YIFFT FLAG 598 ER GFP mitoBFP pdh 634_thumb_w1Con-mcherry-1.tif (RGB) comp.tif]

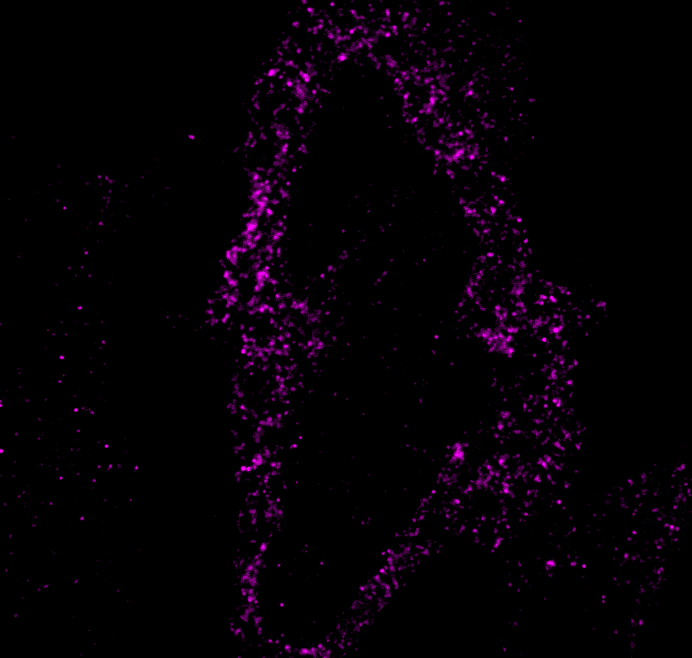

Supplement: Supplementary file 8 — Source Data EV Fig. 4 [file 44319_2023_9_MOESM8_ESM.zip › EV3/o/images/MFN2 IYFFT/control/MAX_MEFs MTCH2 KO MFN2 YIFFT FLAG 598 ER GFP mitoBFP pdh 634_thumb_w1Con-mcherry-1.tif (RGB)ch1.tif]

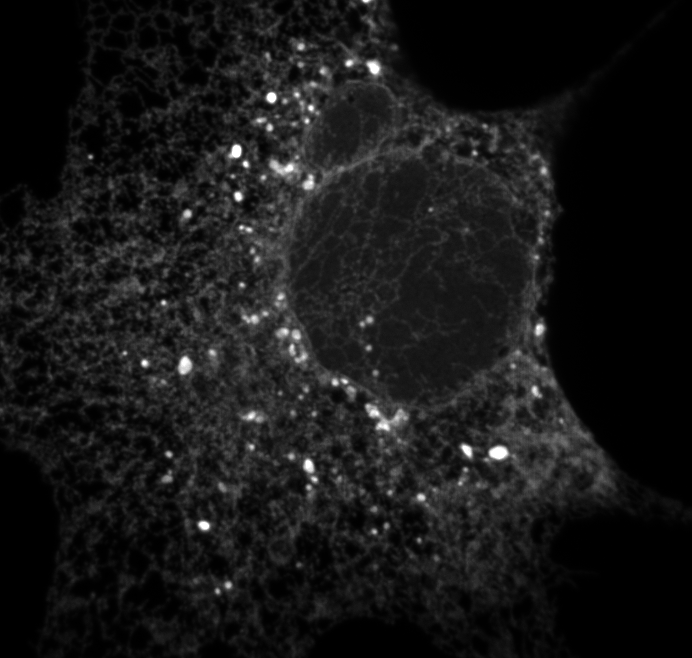

Supplement: Supplementary file 8 — Source Data EV Fig. 4 [file 44319_2023_9_MOESM8_ESM.zip › EV3/o/images/MFN2 IYFFT/FSG/MAX_MEFS MTCH2 KO IYFFT 598 ER GFP MITO BFP MRPL 652_thumb_w1Con-mcherry-1.tif]

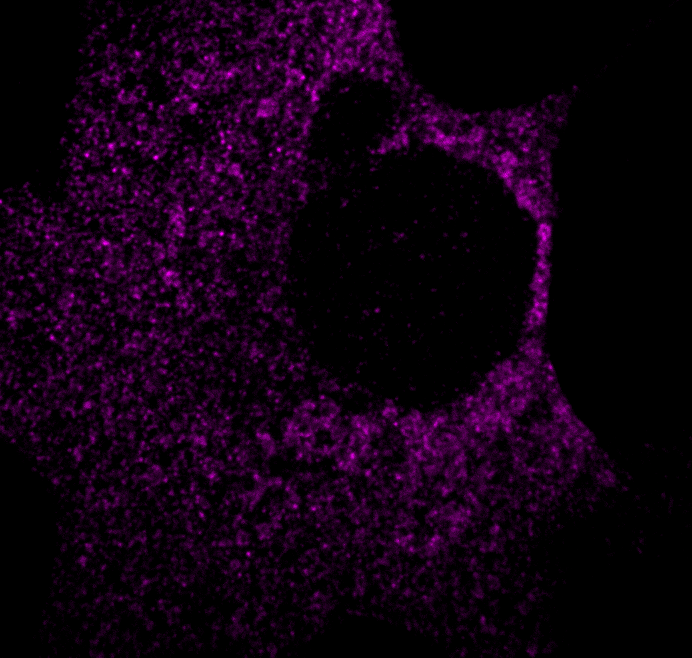

Supplement: Supplementary file 8 — Source Data EV Fig. 4 [file 44319_2023_9_MOESM8_ESM.zip › EV3/o/images/MFN2 IYFFT/FSG/MAX_MEFS MTCH2 KO IYFFT 598 ER GFP MITO BFP MRPL 652_thumb_w1Con-mcherry-1.tif (RGB) ch1.tif]

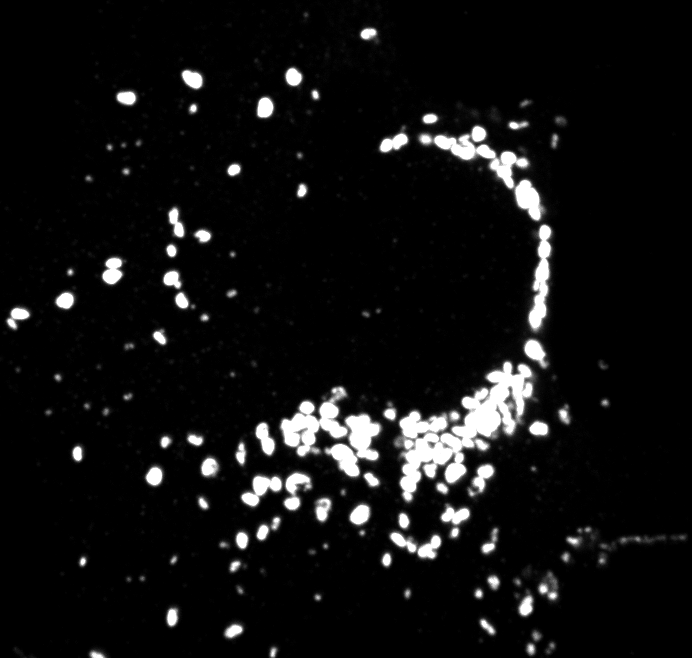

Supplement: Supplementary file 8 — Source Data EV Fig. 4 [file 44319_2023_9_MOESM8_ESM.zip › EV3/o/images/MFN2 IYFFT/FSG/MAX_MEFS MTCH2 KO IYFFT 598 ER GFP MITO BFP MRPL 652_thumb_w1Con-mcherry-1.tif (RGB) ch2.tif]

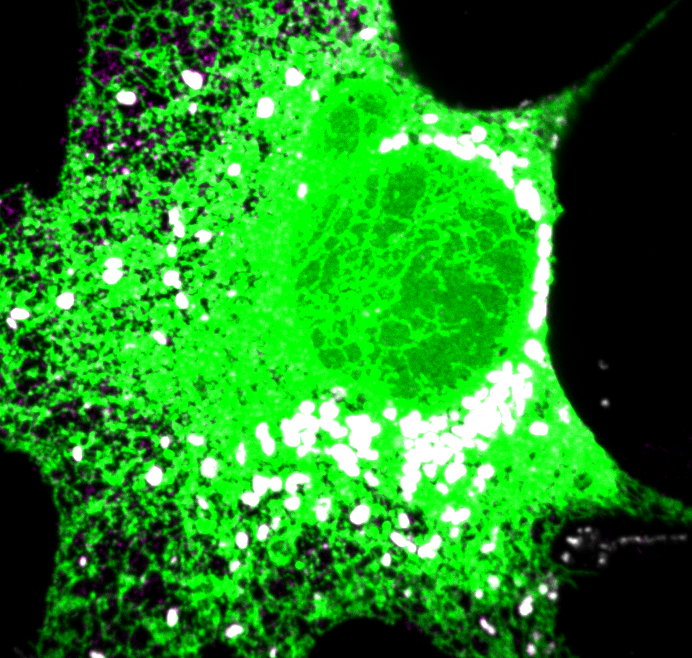

Supplement: Supplementary file 8 — Source Data EV Fig. 4 [file 44319_2023_9_MOESM8_ESM.zip › EV3/o/images/MFN2 IYFFT/FSG/MAX_MEFS MTCH2 KO IYFFT 598 ER GFP MITO BFP MRPL 652_thumb_w1Con-mcherry-1.tif (RGB) composite.tif]

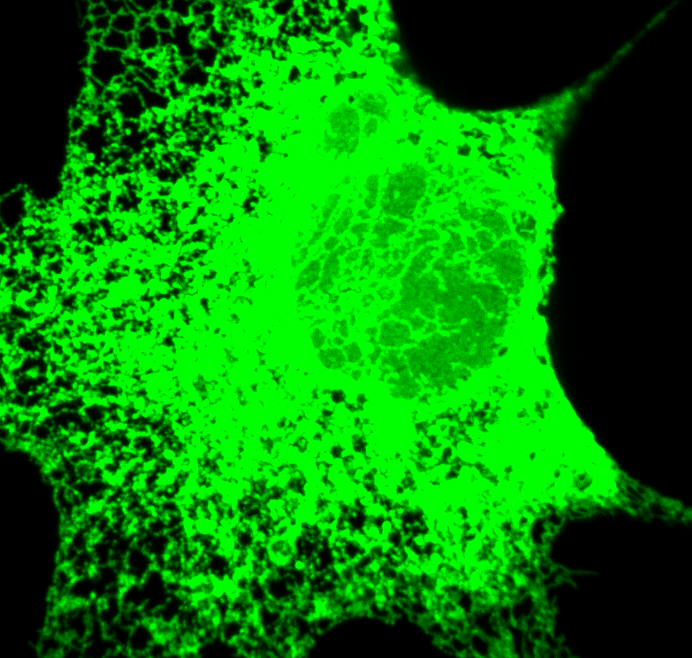

Supplement: Supplementary file 8 — Source Data EV Fig. 4 [file 44319_2023_9_MOESM8_ESM.zip › EV3/o/images/MFN2 IYFFT/FSG/MAX_MEFS MTCH2 KO IYFFT 598 ER GFP MITO BFP MRPL 652_thumb_w1Con-mcherry-1.tif (RGB)ch3.tif]

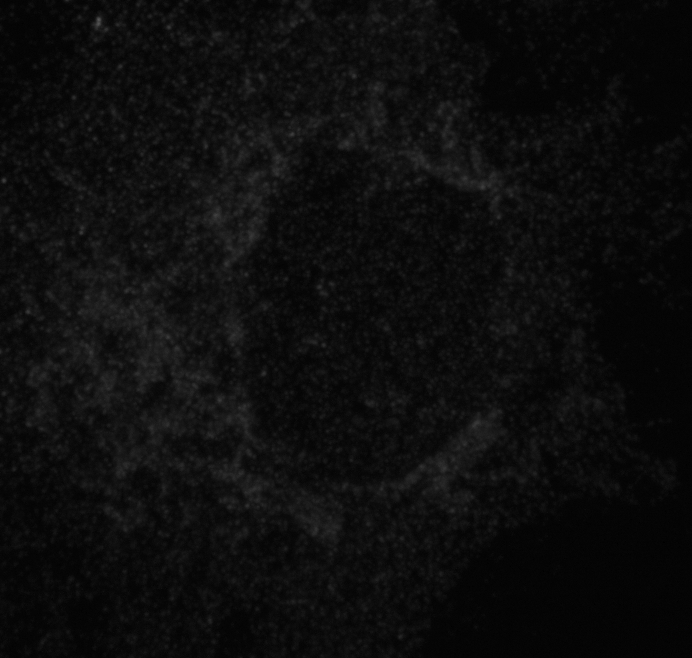

Supplement: Supplementary file 8 — Source Data EV Fig. 4 [file 44319_2023_9_MOESM8_ESM.zip › EV3/o/images/MFN2 k109a/control/MAX_MEFs MTCH2 KO MFN2 K109A 598 Citc648_thumb_w1Con-mcherry-1.tif]

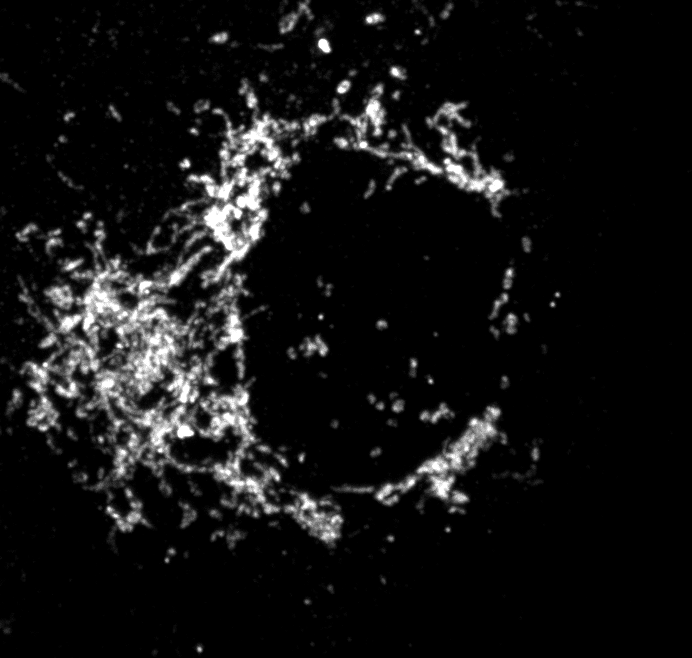

Supplement: Supplementary file 8 — Source Data EV Fig. 4 [file 44319_2023_9_MOESM8_ESM.zip › EV3/o/images/MFN2 k109a/control/MAX_MEFs MTCH2 KO MFN2 K109A 598 Citc648_thumb_w1Con-mcherry-1.tif (RGB) ch1.tif]

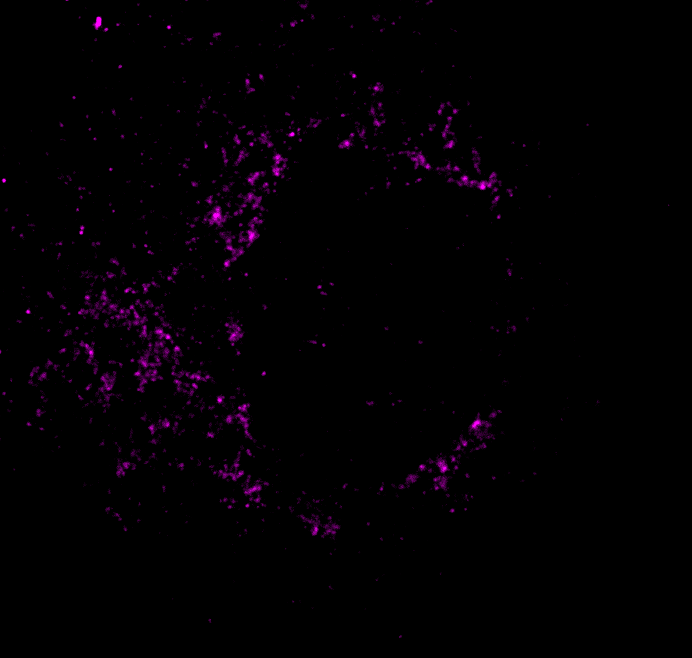

Supplement: Supplementary file 8 — Source Data EV Fig. 4 [file 44319_2023_9_MOESM8_ESM.zip › EV3/o/images/MFN2 k109a/control/MAX_MEFs MTCH2 KO MFN2 K109A 598 Citc648_thumb_w1Con-mcherry-1.tif (RGB) ch2.tif]

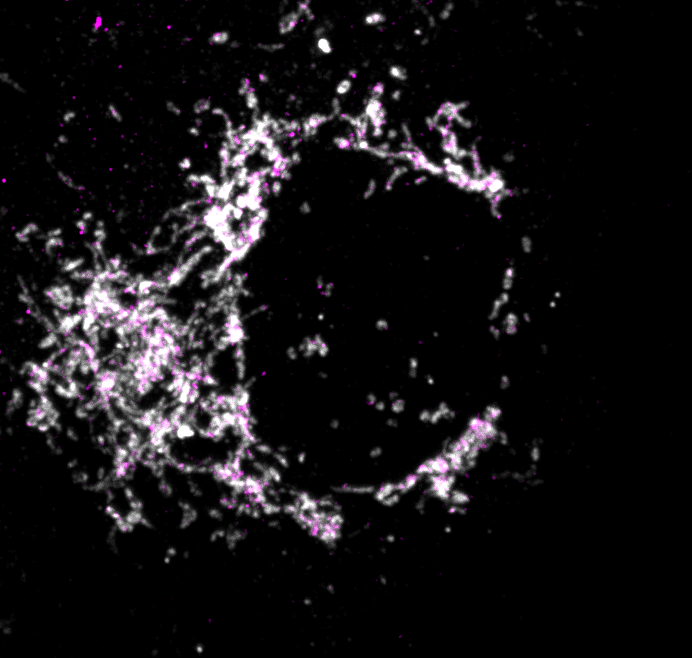

Supplement: Supplementary file 8 — Source Data EV Fig. 4 [file 44319_2023_9_MOESM8_ESM.zip › EV3/o/images/MFN2 k109a/control/MAX_MEFs MTCH2 KO MFN2 K109A 598 Citc648_thumb_w1Con-mcherry-1.tif (RGB) comp.tif]

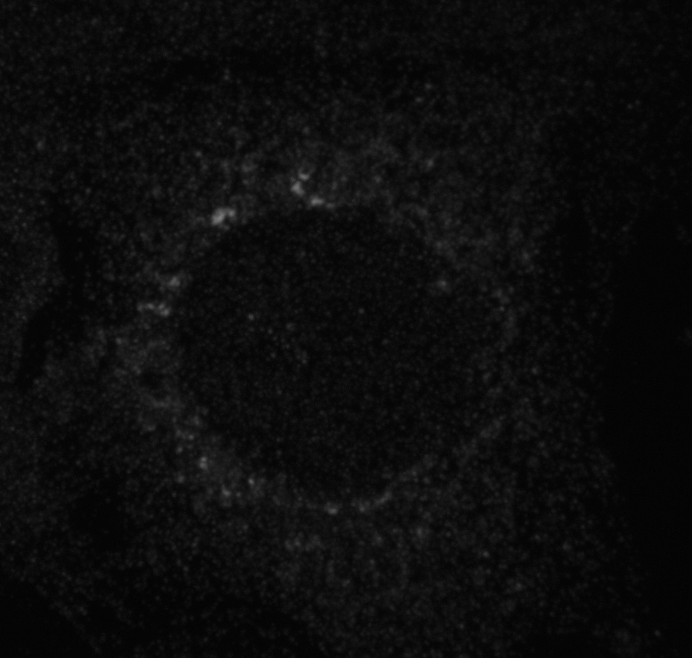

Supplement: Supplementary file 8 — Source Data EV Fig. 4 [file 44319_2023_9_MOESM8_ESM.zip › EV3/o/images/MFN2 k109a/fsg/MAX_MEFs MTCH2 KO MFN2 K109A 598 Citc634_thumb_w1Con-mcherry-1.tif]

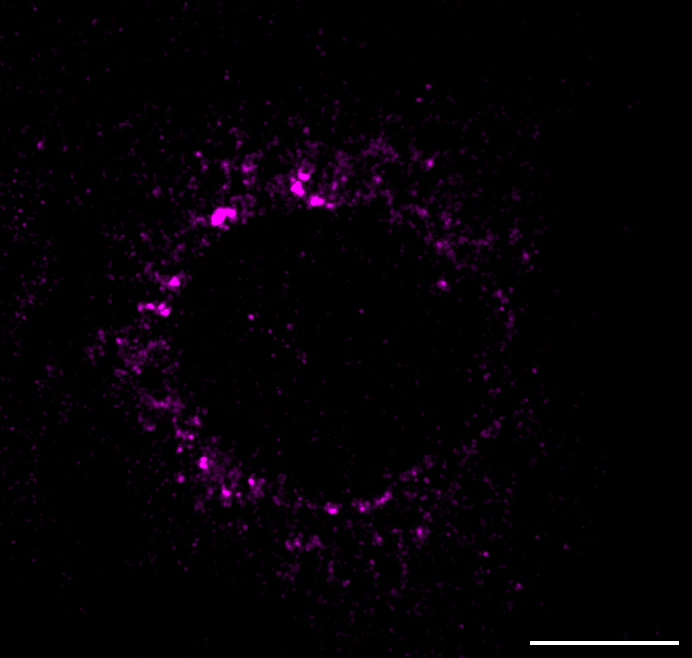

Supplement: Supplementary file 8 — Source Data EV Fig. 4 [file 44319_2023_9_MOESM8_ESM.zip › EV3/o/images/MFN2 k109a/fsg/MAX_MEFs MTCH2 KO MFN2 K109A 598 Citc634_thumb_w1Con-mcherry-1.tif (RGB) ch1-1 scale.tif]

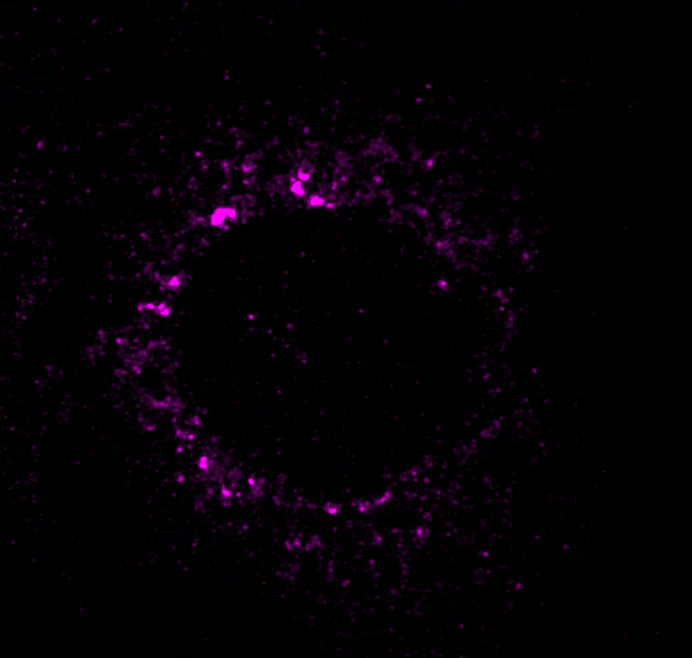

Supplement: Supplementary file 8 — Source Data EV Fig. 4 [file 44319_2023_9_MOESM8_ESM.zip › EV3/o/images/MFN2 k109a/fsg/MAX_MEFs MTCH2 KO MFN2 K109A 598 Citc634_thumb_w1Con-mcherry-1.tif (RGB) ch1.tif]

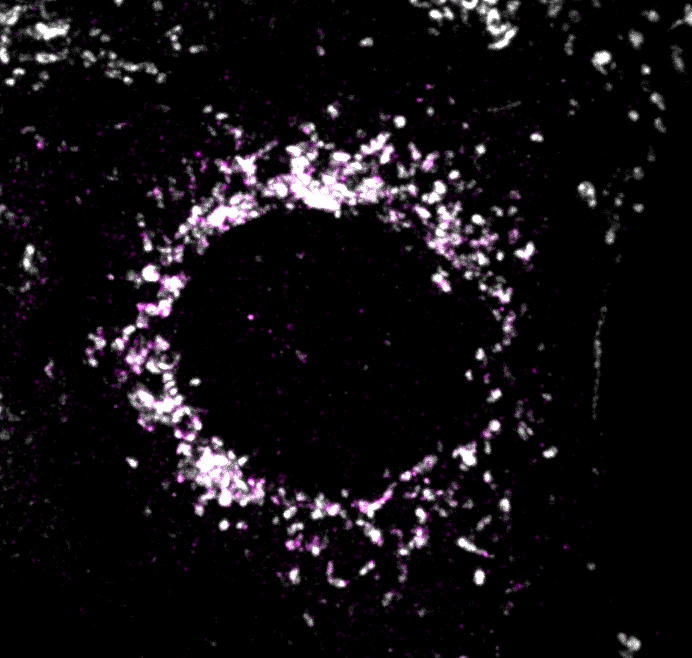

Supplement: Supplementary file 8 — Source Data EV Fig. 4 [file 44319_2023_9_MOESM8_ESM.zip › EV3/o/images/MFN2 k109a/fsg/MAX_MEFs MTCH2 KO MFN2 K109A 598 Citc634_thumb_w1Con-mcherry-1.tif (RGB) ch3.tif]

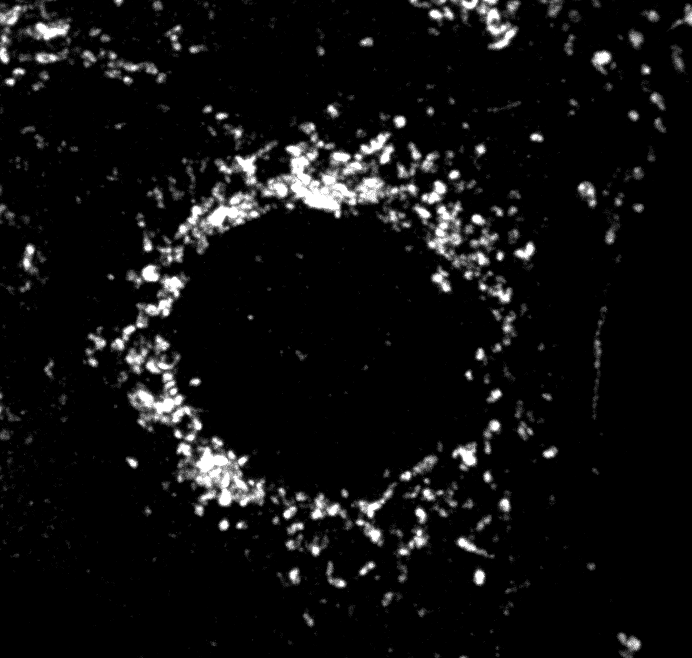

Supplement: Supplementary file 8 — Source Data EV Fig. 4 [file 44319_2023_9_MOESM8_ESM.zip › EV3/o/images/MFN2 k109a/fsg/MAX_MEFs MTCH2 KO MFN2 K109A 598 Citc634_thumb_w1Con-mcherry-1.tif (RGB)ch2.tif]

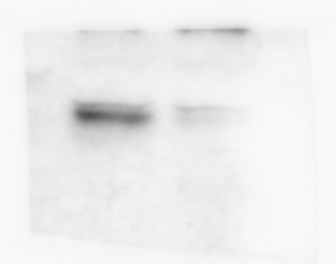

Supplement: Supplementary file 8 — Source Data EV Fig. 4 [file 44319_2023_9_MOESM8_ESM.zip › EV3/q/BLOTS/GPAT3 BLOT.tif]

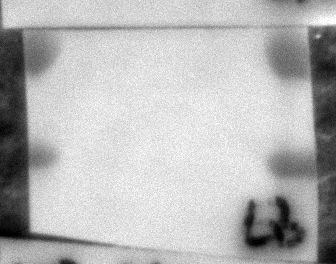

Supplement: Supplementary file 8 — Source Data EV Fig. 4 [file 44319_2023_9_MOESM8_ESM.zip › EV3/q/BLOTS/GPAT3 MEMBRANE.tif]

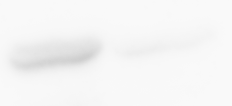

Supplement: Supplementary file 8 — Source Data EV Fig. 4 [file 44319_2023_9_MOESM8_ESM.zip › EV3/q/BLOTS/GPAT3.tif]

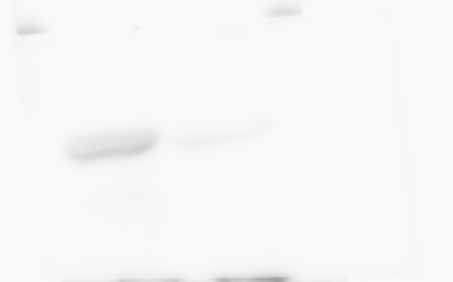

Supplement: Supplementary file 8 — Source Data EV Fig. 4 [file 44319_2023_9_MOESM8_ESM.zip › EV3/q/BLOTS/GPAT4 BLOT.tif]

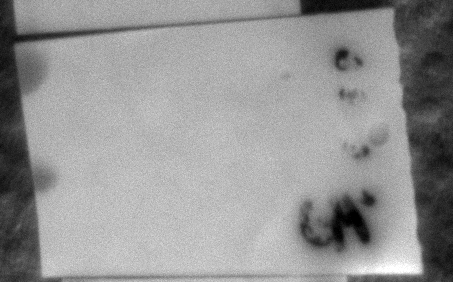

Supplement: Supplementary file 8 — Source Data EV Fig. 4 [file 44319_2023_9_MOESM8_ESM.zip › EV3/q/BLOTS/GPAT4 MEMBRANE.tif]

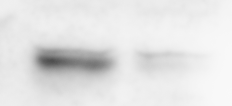

Supplement: Supplementary file 8 — Source Data EV Fig. 4 [file 44319_2023_9_MOESM8_ESM.zip › EV3/q/BLOTS/GPAT4.tif]

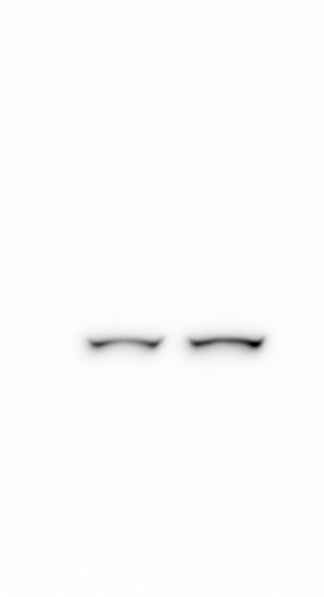

Supplement: Supplementary file 8 — Source Data EV Fig. 4 [file 44319_2023_9_MOESM8_ESM.zip › EV3/q/BLOTS/VINCULIN BLOT.tif]

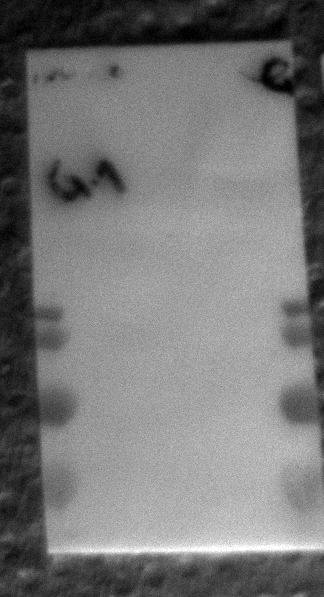

Supplement: Supplementary file 8 — Source Data EV Fig. 4 [file 44319_2023_9_MOESM8_ESM.zip › EV3/q/BLOTS/VINCULIN MEMBRANE.tif]

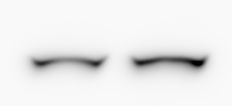

Supplement: Supplementary file 8 — Source Data EV Fig. 4 [file 44319_2023_9_MOESM8_ESM.zip › EV3/q/BLOTS/VINCULIN.tif]

# EV 3Q GPAT3/4 SILENCING IN MTCH2 KO MEFs

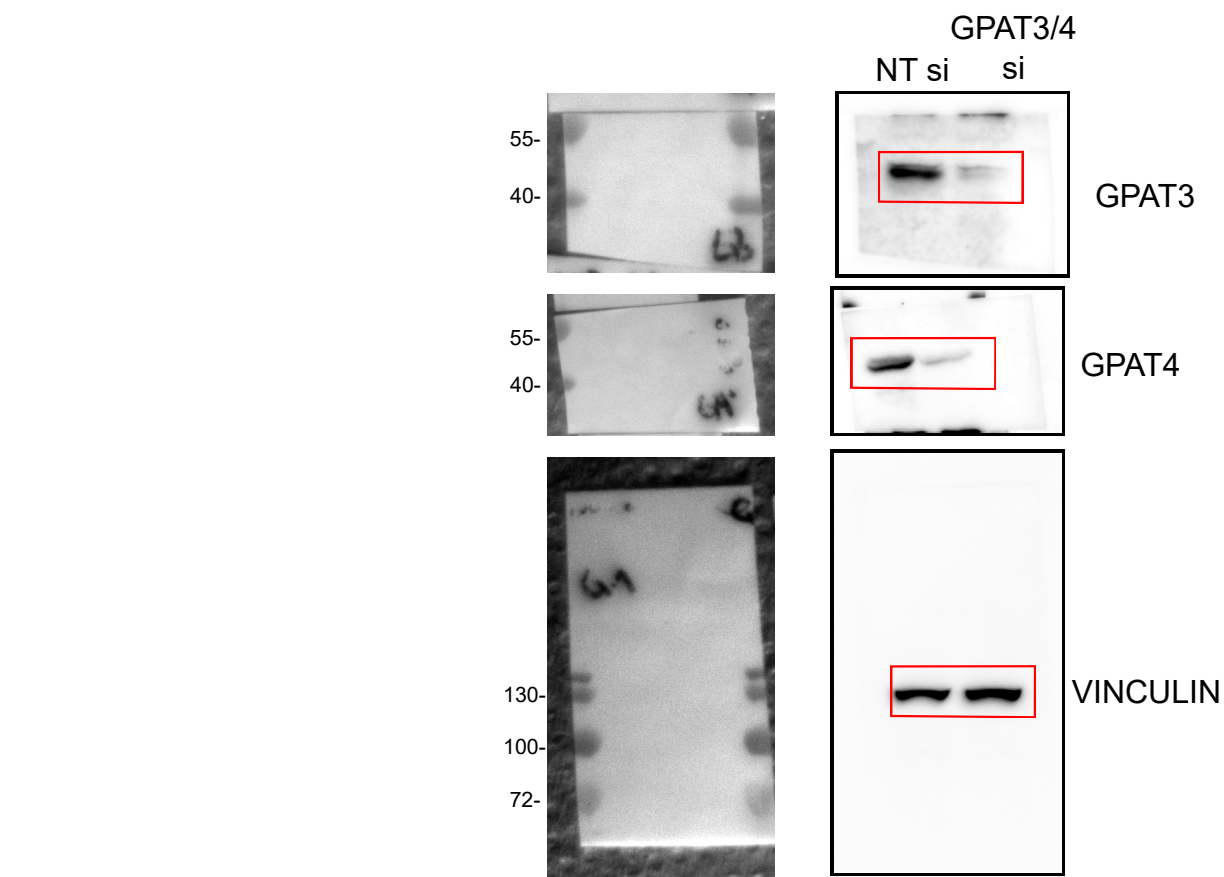

Supplement: Supplementary file 8 — Source Data EV Fig. 4 [file 44319_2023_9_MOESM8_ESM.zip › EV3/q/UNCROPED BLOTS FIG3B EV3Q.pdf]
