# Supplementary material for: MTCH2 cooperates with MFN2 and lysophosphatidic acid synthesis to sustain mitochondrial fusion
Source: EMBO Rep. 2023 Dec 14;25(1):8. doi: 10.1038/s44319-023-00009-1 (PMC10897490; doi:10.1038/s44319-023-00009-1)
Supplement: Supplementary file 10 — Expanded View Figures [file 44319_2023_9_MOESM10_ESM.pdf]

Expanded View Figures

Figure EV1. 1 MTCH2 and MFN1 form a mitochondrial fusion pathway independent of MFN2.

(**A**) Representative images of MFN1 KO and MTCH2 KO MEFs either not expressing (control) or expressing MFN1-GFP. Mitochondria were stained with αTOMM20 antibody. Scale bar 10 μm. (**B**) Classical classification of mitochondrial network morphology of cells, showing average and SEM of three separate experiments (at least 20 cells were analyzed per condition). (**C**) Mitochondrial aspect ratio analysis. At least 20 control and 20 transfected cells were analyzed per condition. One-way ANOVA statistical analysis and SEM are shown. (**D**) Western blot analysis of mitochondrial fusion/fission machinery in heavy membrane (HM) fractions of WT and MTCH2 KO MEFs. (**E**) BN-GE of WT and MTCH2 KO HM fractions blotted with anti-MFN1 and MFN2 antibodies. SDHA serves as control. (**F**) qRT-PCR of MFN1 expression levels in WT MEFs treated with NT-siRNA or with MTCH1-siRNA. (**G**) Representative images of WT MEFs treated with NT-siRNA or with MTCH1-siRNA for 3 days. Mitochondria were stained with αTOMM40 antibody. Scale bar 10 μm. (**H**) Classical classification of mitochondrial network morphology of cells, showing average and SEM of three separate experiments (at least 20 cells were analyzed per condition). (**I**) Mitochondrial aspect ratio analysis. At least 20 cells were analyzed per condition. T-test statistical analysis and SEM are shown. (**J**) Representative images of MTCH2 KO MEFs expressing WT MTCH2-GFP, MTCH2-K25E GFP, or MTCH2-D189R GFP. Mitochondria were stained with αTOMM40 antibody. Scale bar 10 μm. (**K**) Classical classification of mitochondrial network morphology of cells, showing average and SEM of three separate experiments (at least 20 cells were analyzed per condition). (**L**) Mitochondrial aspect ratio analysis. At least 20 cells were analyzed per condition. One-way ANOVA statistical analysis and SEM are shown. Data information: (**C**,**I**,**L**) ns, not significant, $^{***}P < 0.001$. Source data are available online for this figure.

▶

© The Author(s)

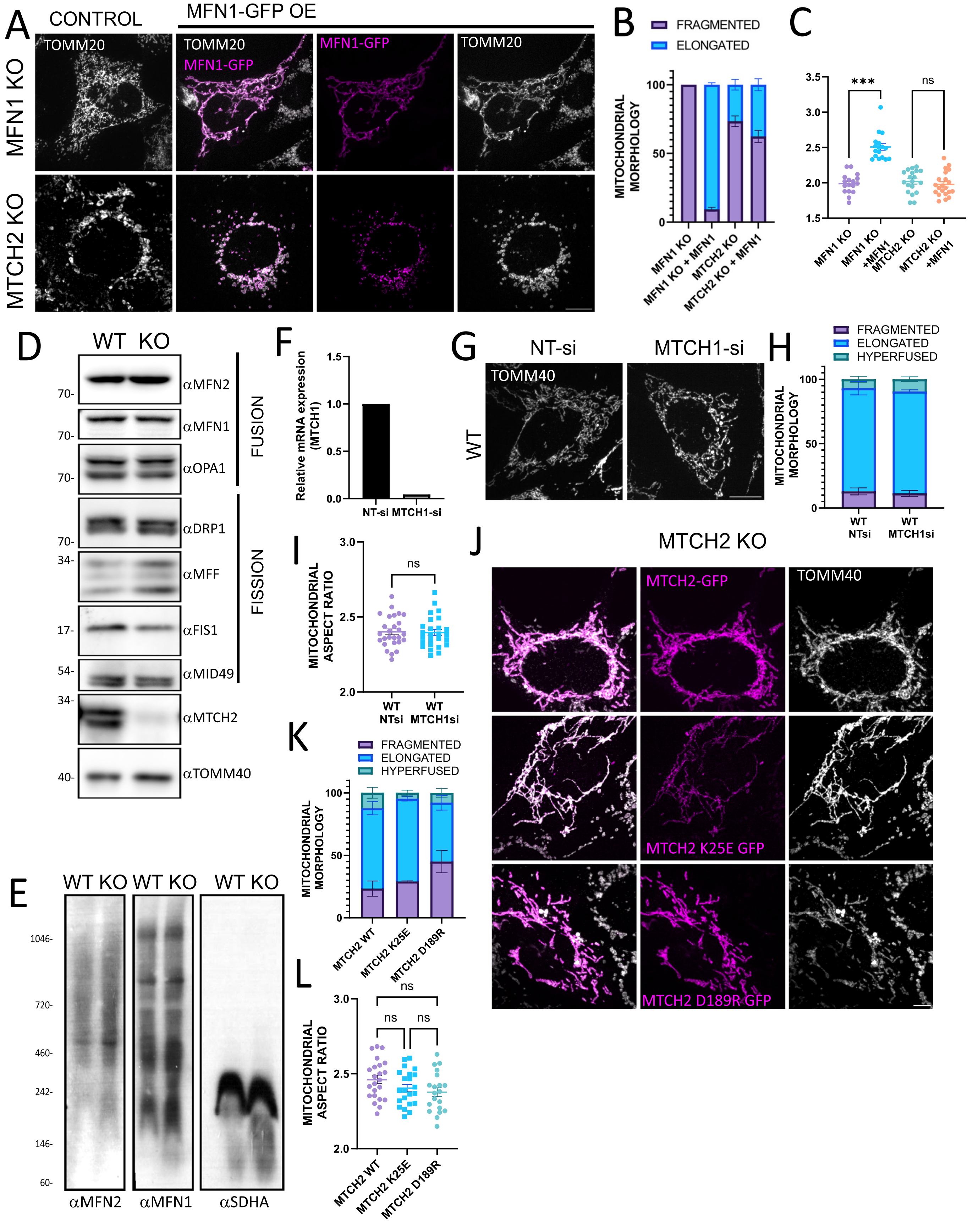

A
CONTROL
MFN1-GFP OE
MFN1 KO
MTCH2 KO
TOMM20
MFN1-GFP
B
FRAGMENTED
ELONGATED
MITOCHONDRIAL MORPHOLOGY
MFN1 KO
MFN1 KO + MFN1
MTCH2 KO
MTCH2 KO + MFN1
C
***
ns
MFN1 KO
MFN1 KO +MFN1
MTCH2 KO
MTCH2 KO +MFN1
D
WT KO
αMFN2
αMFN1
αOPA1
αDRP1
αMFF
αFIS1
αMID49
αMTCH2
αTOMM40
FUSION
FISSION
E
WT KO WT KO WT KO
αMFN2 αMFN1 αSDHA
F
Relative mRNA expression (MTCH1)
NT-si MTCH1-si
G
NT-si
MTCH1-si
WT
TOMM40
H
FRAGMENTED
ELONGATED
HYPERFUSED
MITOCHONDRIAL MORPHOLOGY
WT NTsi
WT MTCH1si
I
MITOCHONDRIAL ASPECT RATIO
ns
WT NTsi
WT MTCH1si
J
MTCH2 KO
MTCH2-GFP
TOMM40
MTCH2 K25E GFP
MTCH2 D189R GFP
K
FRAGMENTED
ELONGATED
HYPERFUSED
MITOCHONDRIAL MORPHOLOGY
MTCH2 WT
MTCH2 K25E
MTCH2 D189R
L
MITOCHONDRIAL ASPECT RATIO
ns
MTCH2 WT
MTCH2 K25E
MTCH2 D189R

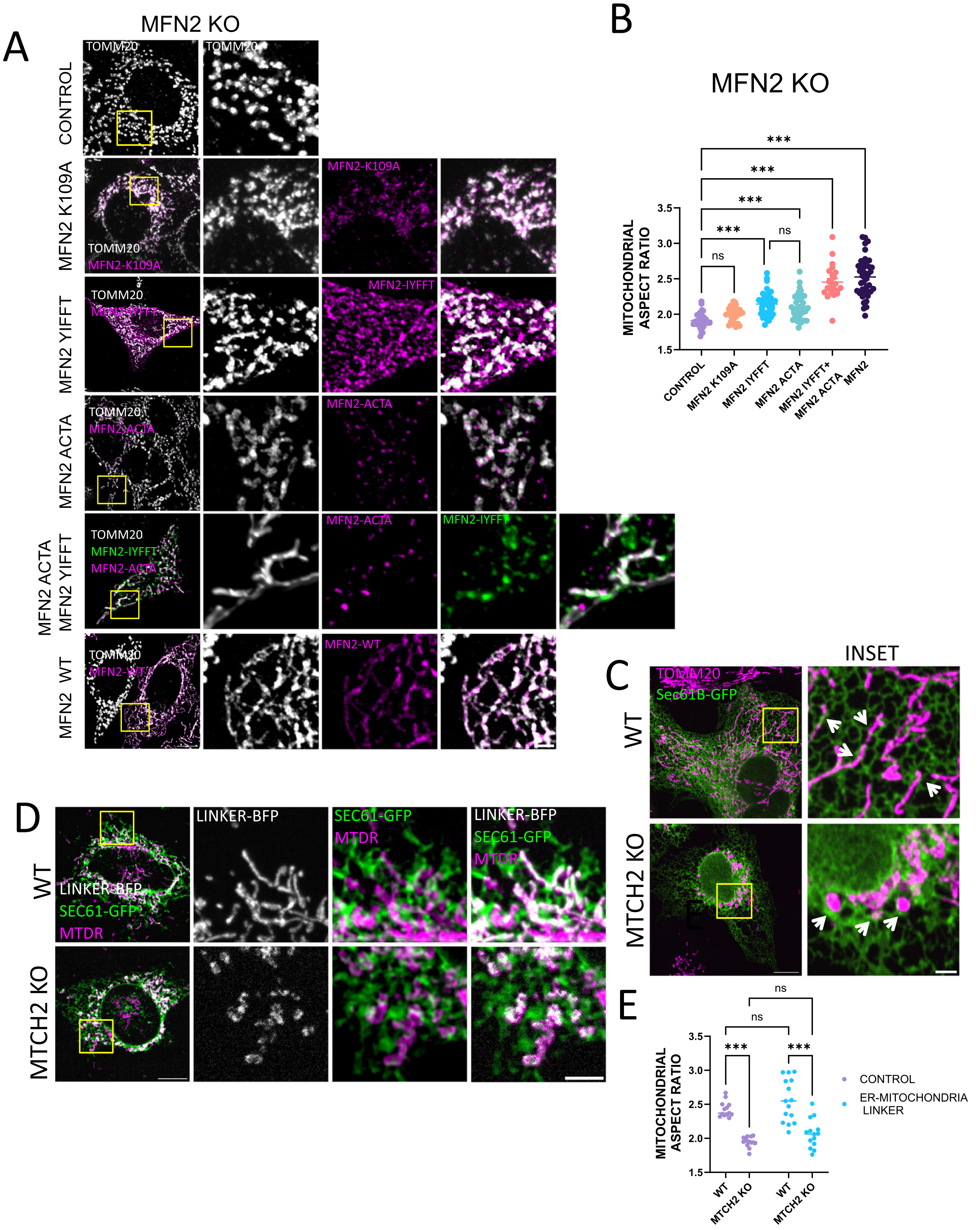
A
MFN2 KO
CONTROL
TOMM20
MFN2 K109A
MFN2-K109A
MFN2 YIFFT
MFN2-IYFFT
MFN2 ACTA
MFN2-ACTA
MFN2 ACTA
MFN2 YIFFT
MFN2 WT
MFN2-WT
B
MFN2 KO
MITOCHONDRIAL ASPECT RATIO
3.5
3.0
2.5
2.0
1.5
***
ns
CONTROL
MFN2 K109A
MFN2 IYFFT
MFN2 ACTA
MFN2 IYFFT+ MFN2 ACTA
MFN2
C
INSET
WT
TOMM20
Sec61B-GFP
MTCH2 KO
D
WT
LINKER-BFP
SEC61-GFP
MTDR
MTCH2 KO
E
MITOCHONDRIAL ASPECT RATIO
3.5
3.0
2.5
2.0
1.5
***
ns
WT
MTCH2 KO
CONTROL
ER-MITOCHONDRIA LINKER

Figure EV2. MFN2's ability to enforce mitochondrial fusion in MTCH2 KO cells is partially disconnected from its fusion activity.

(**A**) Representatives images of MFN2 KO MEFs control or transfected with MFN2-K109A-FLAG, MFN2-IYFFT-MYC, MFN2-ACTA-V5, WT MFN2-MYC, or co-transfected with MFN2-IYFFT-MYC and MFN2-ACTA-V5. Mitochondria were stained with αTOMM20 antibody. Scale bar 10 μm; inset scale bar 5 μm. (**B**) Mitochondrial aspect ratio analysis (at least 25 control and 25 transfected cells were analyzed per condition). One-way ANOVA statistical analysis and SEM are shown. (**C**) Representative images of WT and MTCH2 KO cells transfected with the ER marker Sec61b-GFP and stained with αTOMM20 antibody. Arrowheads indicate areas of close proximity between ER and mitochondria. Scale bar 10 μm; inset scale bar 2 μm. (**D**) Representatives images of WT and MTCH2 KO MEFs co-transfected with the ER-mitochondria linker construct AKAP-mtagBFP2-UBC6 and the ER marker Sec61b-GFP. Mitochondria were labeled with MTDR. Scale bar 10 μm; Scale bar inset 5 μm. (**E**) Mitochondrial aspect ratio analysis. At least 15 control and 15 transfected cells were analyzed per condition. One-way ANOVA statistical analysis and SEM are shown. Data information: (**B**,**E**) ns, not significant, ***$P < 0.001$. Source data are available online for this figure.

© The Author(s)

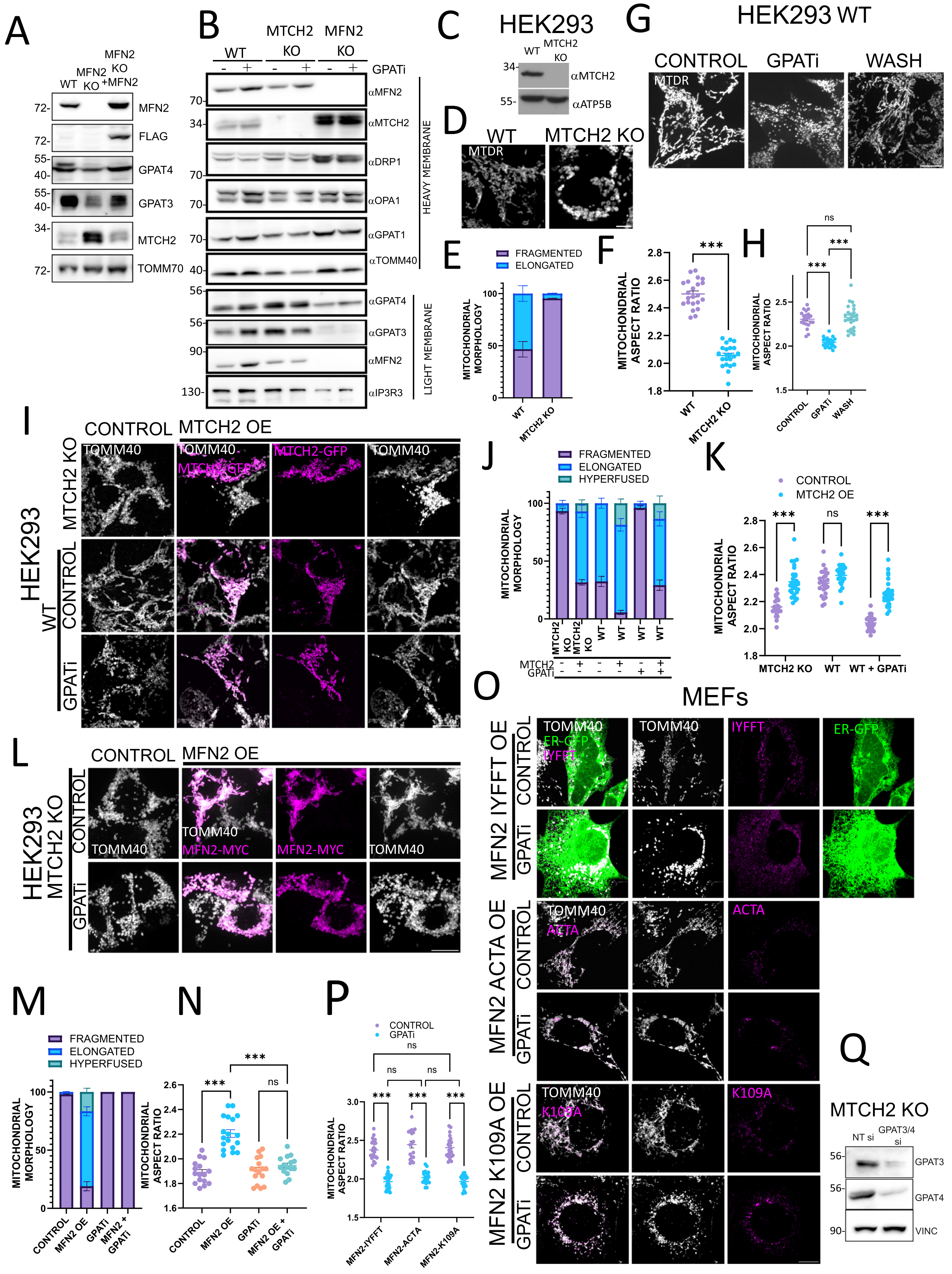
A
WT
MFN2 KO
MFN2 KO +MFN2
MFN2
FLAG
GPAT4
GPAT3
MTCH2
TOMM70
B
WT
MTCH2 KO
MFN2 KO
GPATi
αMFN2
αMTCH2
αDRP1
αOPA1
αGPAT1
αTOMM40
HEAVY MEMBRANE
αGPAT4
αGPAT3
αMFN2
αIP3R3
LIGHT MEMBRANE
C
HEK293
WT
MTCH2 KO
αMTCH2
αATP5B
D
WT
MTCH2 KO
MTDR
E
FRAGMENTED
ELONGATED
MITOCHONDRIAL MORPHOLOGY
F
MITOCHONDRIAL ASPECT RATIO
G
HEK293 WT
CONTROL
GPATi
WASH
H
I
CONTROL
MTCH2 OE
HEK293
MTCH2 KO
WT
CONTROL
GPATi
TOMM40
MTCH2-GFP
J
HYPERFUSED
K
MTCH2 KO
WT
WT + GPATi
L
MFN2 OE
MFN2-MYC
M
N
P
MFN2-IYFFT
MFN2-ACTA
MFN2-K109A
O
MEFs
MFN2 IYFFT OE
MFN2 ACTA OE
MFN2 K109A OE
ER-GFP
IYFFT
ACTA
K109A
Q
MTCH2 KO
NT si
GPAT3/4 si
GPAT3
GPAT4
VINC

 © The Author(s)

Figure EV3. MTCH2 deletion uncovers the requirement of LPA synthesis to sustain mitochondrial fusion enforced by MFN2 overexpression.

(**A**) Western blot analysis in whole-cell lysates of GPAT3/4 and MTCH2 in WT, MFN2 KO, and MFN2 KO MEFs rescued with FLAG-MFN2; TOMM70 serves as loading control. (**B**) Western blot analysis of mitochondrial fusion/fission machinery and GPATs in HM and LM fractions of WT, MTCH2 KO, and MFN2 KO MEFs control or treated for 16 h with GPATi. TOMM40 serves as a HM loading control, and IP3R3 serve as a LM loading control. (**C**) Western blot analysis of MTCH2 expression in WT and MTCH2 KO HEK293T cells; ATPVb (complex V) serves as a loading control. (**D**) Representatives images of mitochondria of WT and MTCH2 KO HEK293T cells. Mitochondria were labeled with MTDR. Scale bar 5 µm. (**E**) Classical classification of mitochondrial network morphology. Results are presented as average and SEM of three separate experiments; at least 20 fields were analyzed per condition. (**F**) Mitochondrial aspect ratio analysis. At least 25 fields were analyzed per condition. One-way ANOVA statistical analysis and SEM are shown. (**G**) Representatives images of WT HEK293T cells either non-treated (control) or treated for 16 h with GPATi $-/+$ wash and imaged after 4 h. Mitochondria were labeled with MTDR. Scale bar 10 µm. (**H**) Mitochondrial aspect ratio analysis. At least 25 fields were analyzed per condition. One-way ANOVA statistical analysis and SEM are shown. (**I**) Representatives images of WT and MTCH2 KO HEK293T cells overexpressing MTCH2-GFP in control conditions, and of WT cells treated for 16 h with GPATi and overexpressing MTCH2-GFP. Mitochondria were stained using αTOMM40 antibody. Scale bar 10 µm. (**J**) Classical classification of mitochondrial network morphology. Results are presented as average and SEM of three separate experiments; at least 20 cells were analyzed per condition. (**K**) Mitochondrial aspect ratio analysis. At least 25 control and 25 transfected cells were analyzed per condition. One-way ANOVA statistical analysis and SEM are shown. (**L**) Representatives images of MTCH2 KO HEK293T cells control or overexpressing MFN2-MYC non-treated or treated for 16 h with GPATi. Mitochondria were stained using αTOMM40 antibody. Scale bar 10 µm. (**M**) Classical classification of mitochondrial network morphology. Results are presented as average and SEM of three separate experiments; at least 20 cells were analyzed per condition. (**N**) Mitochondrial aspect ratio analysis of cells presented in L. At least 25 control and 25 transfected cells were analyzed per condition. One-way ANOVA statistical analysis and SEM are shown. (**O**) Representatives images of MTCH2 KO MEFs transfected with MFN2-IYFFT-FLAG, MFN2-ACTA-FLAG, or MFN2-K109A-FLAG non-treated or treated for 16 h with GPATi. MFN2-IYFFT and MFN2-ACTA mutants were co-transfected with the ER marker Sec61-GFP to ensure the correct subcellular localization. Mitochondria were stained with αTOMM40 antibody. Scale bar 10 µm. (**P**) Mitochondrial aspect ratio analysis of cells presented in (**O**). At least 20 transfected cells were analyzed per condition. One-way ANOVA statistical analysis and SEM are shown. (**Q**) Western blot analysis in whole-cell lysates of GPAT3/4 expression in MTCH2 KO MEFs cells treated with NT-siRNA or with a combination of GPAT3 and 4 siRNAs; vinculin (VINC) serves as loading control. Data information: (**F**,**H**,**K**,**N**,**P**) ns, not significant, ***$P < 0.001$. Source data are available online for this figure.

© The Author(s)

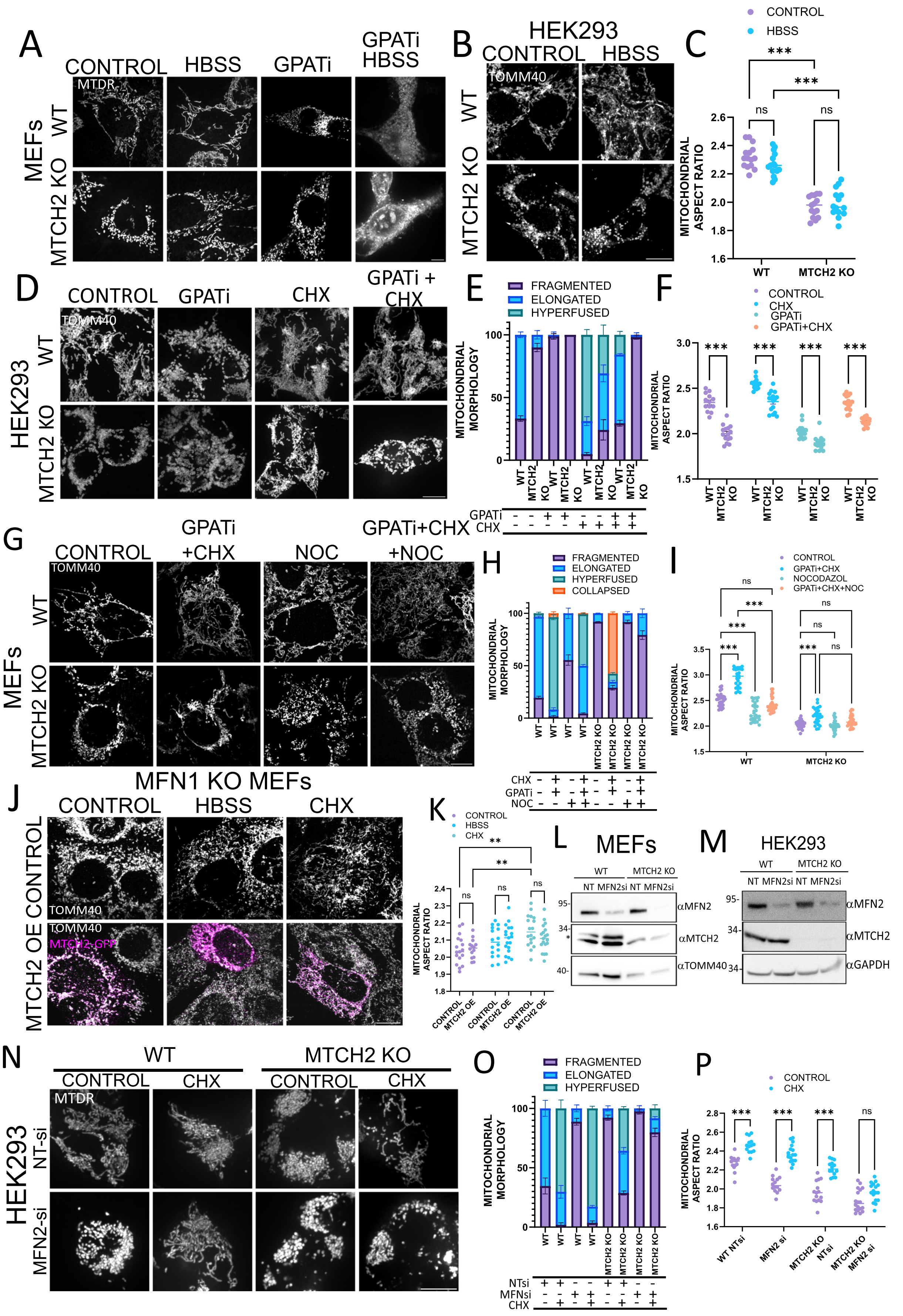
A
CONTROL
HBSS
GPATi
GPATi HBSS
MTDR
MEFs
WT
MTCH2 KO
B
HEK293
CONTROL
HBSS
TOMM40
C
CONTROL
HBSS
MITOCHONDRIAL ASPECT RATIO
WT
MTCH2 KO
D
GPATi + CHX
CHX
HEK293
E
FRAGMENTED
ELONGATED
HYPERFUSED
MITOCHONDRIAL MORPHOLOGY
F
G
GPATi +CHX
NOC
GPATi+CHX +NOC
H
COLLAPSED
I
NOCODAZOL
GPATi+CHX+NOC
J
MFN1 KO MEFs
MTCH2 OE
MTCH2-GFP
K
L
MEFs
NT
MFN2si
αMFN2
αMTCH2
αTOMM40
M
αGAPDH
N
NT-si
MFN2-si
O
NTsi
MFNsi
P
WT NTsi
MFN2 si
MTCH2 KO NTsi
MTCH2 KO MFN2 si

 © The Author(s)

Figure EV4. MTCH2 cooperates with MFN2 and LPA synthesis to sustain mitochondrial fusion under cellular stress conditions.

(**A**) Representatives images of WT and MTCH2 KO MEFs either non-treated (control), treated with HBSS for 4 h, treated with GPATi for 16 h, or pretreated for 16 h with GPATi and then treated with HBSS for 4 h in the presence of the inhibitor. Mitochondria were labeled with MTDR. Scale bar 10 μm. (**B**) Representatives images of WT and MTCH2 KO HEK293T cells non-treated (control) or treated with HBSS for 4 h. Mitochondria were stained with αTOMM40 antibody. Scale bar 10 μm. (**C**) Mitochondrial aspect ratio analysis. At least 20 fields were analyzed per condition. One-way ANOVA statistical analysis. (**D**) Representatives images of WT and MTCH2 KO HEK293T cells either non-treated (control), treated with GPATi for 16 h, treated with 10 μM CHX for 4 h or pretreated for 16 h with GPATi and then treated with 10 μM CHX for 4 h in the presence of the inhibitor. Scale bar 10 μm. (**E**) Classical classification of mitochondrial network morphology. Results are presented as average and SEM of three different experiments, and at least 30 cells analyzed per condition. (**F**) Mitochondrial aspect ratio analysis. At least 20 fields were analyzed per condition. One-way ANOVA statistical analysis and SEM are shown. (**G**) Representatives images of WT and MTCH2 KO MEFs either non-treated (control), pretreated for 16 h with GPATi and then treated with 10 μM CHX for 4 h in the presence of the inhibitor, treated with 5 μM nocodazole (NOC) for 4 h or pretreated for 16 h with GPATi and then treated with 5 μM NOC and 10 μM CHX for 4 h in the presence of the inhibitor. Mitochondria were labeled with αTOMM40 antibody. Scale bar 10 μm. (**H**) Classical classification of mitochondrial network morphology. Results are presented as average and SEM of three different experiments, and at least 30 cells analyzed per condition. (**I**) Mitochondrial aspect ratio analysis. At least 25 fields were analyzed per condition. One-way ANOVA statistical analysis and SEM are shown. (**J**) Representatives images of MFN1 KO MEFs control or overexpressing MTCH2-GFP in control conditions or treated for 4 h with HBSS or CHX. Mitochondria were stained with αTOMM40 antibody. Scale bar 10 μm. (**K**) Mitochondrial aspect ratio analysis. At least 20 cells were analyzed per condition. One-way ANOVA statistical analysis and SEM are shown. (**L**) Western blot analysis of MFN2 in WT and MTCH2 KO MEFs treated with NT-siRNA or with siRNA targeting MFN2 expression, and TOMM40 serves as loading control. *Depicts a non-specific band. (**M**) Western blot analysis of MFN2 in WT and MTCH2 KO HEK293T cells treated with non-targeting (NT) siRNA or with MFN2 siRNA. GAPDH serves as a loading control. (**N**) Representative images of WT and MTCH2 KO HEK293T cells treated with non-targeting (NT) siRNA or with MFN2 siRNA non-treated or treated with CHX for 4 h. Mitochondria were labeled with MTDR. Scale bar 10 μm. (**O**) Classical classification of mitochondrial network morphology. Results are presented as average and SEM of three different experiments, and at least 30 cells analyzed per condition. (**P**) Mitochondrial aspect ratio. At least 20 fields were analyzed per condition. One-way ANOVA statistical analysis and SEM are shown. Data information: (**C**,**F**,**I**,**K**,**P**) ns, not significant, $^{**}P < 0.01$, $^{***}P < 0.001$. Source data are available online for this figure.

© The Author(s)
