# Supplementary material for: MTCH2 cooperates with MFN2 and lysophosphatidic acid synthesis to sustain mitochondrial fusion
Source: EMBO Rep. 2023 Dec 14;25(1):8. doi: 10.1038/s44319-023-00009-1 (PMC10897490; doi:10.1038/s44319-023-00009-1)

EV 4L MFN2 SILENCING IN MTCH2 KO MEFS

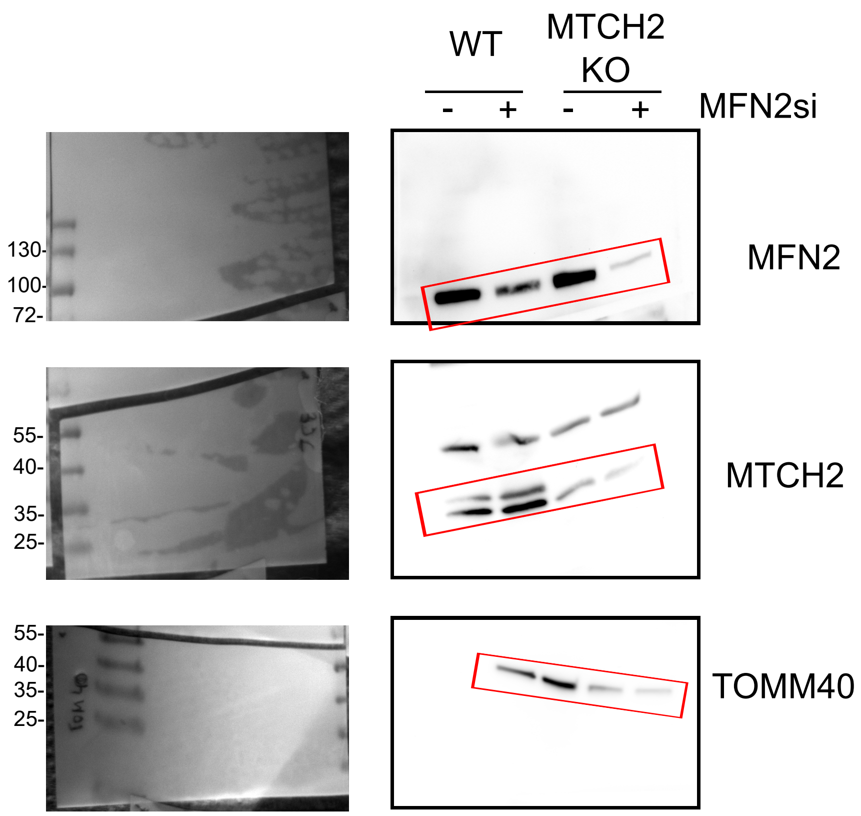

Supplement: Supplementary file 9 — Peer Review File [file 44319_2023_9_MOESM9_ESM.zip › EV4/l/UNCROPED BLOTS EV4L.pdf]
